# Generation and epitope mapping of two distinct neutralizing mAb subsets targeting BVDV E2: a pan-pestivirus cross-reactive linear epitope and a BVDV-specific conformational epitope

**DOI:** 10.1186/s13567-026-01852-5

**Published:** 2026-10-07

**Authors:** Fei Bao, Shijiang Mi, Zhongdi Liu, Shuchi Zhang, Meng Wu, Yixiao Zhang, Changchun Tu, Wenjie Gong

**Affiliations:** 1 State Key Laboratory for Diagnosis and Treatment of Severe Zoonotic Infectious Diseases, Key Laboratory for Zoonosis Research of the Ministry of Education, College of Veterinary Medicine, Jilin University, Changchun, 130062 China; 2 Key Laboratory of Jilin Province for Zoonoses Prevention and Control, State Key Laboratory of Pathogen and Biosecurity, Academy of Military Medical Sciences, Changchun, 130122 China; 3 Chongqing Academy of Animal Sciences, Chongqing, 402460 China; 4 Jiangsu Co-Innovation Center for Prevention and Control of Important Animal Infectious Diseases and Zoonoses, Yangzhou University, Yangzhou, 225009 China

**Keywords:** Bovine viral diarrhea virus, E2 protein, epitope mapping, cross-neutralizing mAb

## Abstract

Bovine viral diarrhea virus (BVDV) is an important infectious pathogen responsible for substantial economic losses in the global cattle industry. The structural protein E2 serves as a major protective antigen of BVDV and is the main target for host neutralizing immune responses. However, its antigenic structure and epitope distribution remain poorly understood. Here, five novel monoclonal antibodies (mAbs) against the E2 protein of BVDV-1 were generated using hybridoma technology, and their reactivity patterns, biological properties, and recognized epitopes were systematically characterized. As a result, one mAb, TBH033, specifically reacted with BVDV, while four mAbs, TBH002, TBH004, TBH008, and TBH009, exhibited broad-spectrum reactivity against different subgenotypes of classical swine fever virus (CSFV) and BVDV, as well as E2 proteins from eight pestivirus species (BVDV-1, BVDV-2, BVDV-3, CSFV, BDV, PPeV, GPeV, and ovIT PeV). All five mAbs exhibited high binding affinity to the E2 protein and potent cross-neutralizing activity against BVDV strains of different subgenotypes. Epitope mapping with chimeric recombinant and site-mutated E2 proteins revealed that TBH002, TBH004, TBH008, and TBH009 recognized a linear epitope in the DA domain, comprising ^70^H, ^72^R, ^74^L and ^76^T as critical residues; TBH033 targeted a BVDV-specific conformational epitope in the DB domain, defined by the conserved amino acid motifs ^103^LCP^105^, ^123^GP^124^. These findings enrich our understanding of the antigenic structure of the E2 protein and may facilitate the design of E2-based diagnostic tools and vaccines.

**Supplementary Information:**

The online version contains supplementary material available at https://doi.org/10.1186/s13567-026-01852-5.

## Introduction

Bovine viral diarrhea virus (BVDV) represents a group of pathogens in cattle that endanger the global livestock industry and inflict severe economic losses worldwide [1]. It includes three species: BVDV-1, BVDV-2, and BVDV-3 (HoBi-like pestivirus), belonging to the genus *Pestivirus* within the family *Flaviviridae*. The genus also contains the serologically related viruses, including classical swine fever virus (CSFV), border disease virus (BDV), and additional pestivirus species newly identified in various domestic and wild animals [2]. BVDV-1 usually causes relatively mild symptoms in cattle, such as depression, fever, and leukopenia, whereas BVDV-2 induces severe thrombocytopenia with hemorrhage and acute fatal disease [3, 4]. BVDV-3 was first detected in fetal bovine serum (FBS) collected in Brazil [5], which can cause respiratory diseases, reproductive failure, acute gastroenteritis, and mucosal disease-like syndrome in ruminants [6, 7]. BVDVs possess extensive genetic diversity; to date, 24 subgenotypes of BVDV-1 (BVDV-1a to BVDV-1x) and 5 subgenotypes of BVDV-2 (BVDV-2a to BVDV-2e) have been identified [8, 9].

The single-stranded positive-sense RNA genome of BVDV encodes a precursor polyprotein, which is processed into four structural and eight nonstructural proteins [10]. Among them, the envelope glycoprotein E2 is located on the surface of viral particles and plays a crucial role in the viral life cycle by mediating viral attachment to host cells and viral fusion [11, 12]. Additionally, as the major protective antigen of BVDV, E2 exhibits strong immunogenicity and can induce the production of neutralizing antibodies in the host [13, 14], making it a preferred target for the development of BVDV subunit vaccines and diagnostic reagents. However, the differences within and between BVDVs are highest in the E2 glycoprotein. For instance, the homology among BVDV-1 E2 proteins is 68–78%, and that between BVDV-1 and BVDV-2 is 61–66%, whereas the homology between BVDV-1 and BVDV-3 is only 57–63% [15]. This genetic divergence highlights the challenge of BVDV vaccine design. Further studies are needed to define neutralizing epitopes on the E2 protein of BVDVs, especially highly conserved ones, to provide a valuable reference for designing next-generation vaccines.

The crystal structure of BVDV E2 has been characterized into three (domains I, II, III) [16] or four domains (DA, DB, DC, and DD) [11], which share high structural similarity with the CSFV E2 protein [12, 17]. Specifically, domain I (DA) of BVDV corresponds to domains B/C of CSFV, while domain II (DB) of BVDV corresponds to domains D/A of CSFV. For CSFV, monoclonal antibodies (mAbs) against the E2 protein have been used to define its antigenic structure, and E2 epitopes have been extensively mapped to the B/C or D/A antigenic units in the N-terminal half of the protein [18, 19]. Furthermore, several new epitopes, including conformational epitopes [20] and linear epitopes, have been identified in the C-terminal region of E2 [21]. Common epitopes between CSFV and BDV have also been mapped in the B/C domain of CSFV E2 [22, 23]. However, the antigenic structure of BVDV E2 remains poorly defined.

Paton et al. reported two antigenic domains on the E2 protein of BVDV-1, one of which was described as immunodominant [24]. Subsequently, two neutralizing mAbs, 157 and 348, were reported to recognize a BVDV-1 specific epitope and a BVDV-1/BVDV-2 shared neutralizing epitope; three (positions 9, 32, and 72) and four (positions 71, 72, 74, and 77) critical amino acids affecting the epitopes recognized by mAbs 157 and 348 were identified, respectively [3]. For BVDV-2, nine neutralizing mAbs against the E2 protein were found to bind highly conserved epitopes in three antigenic domains, but their precise locations were not demonstrated [25]. In addition, several E2 mAbs, such as 4E1 and 19F7, mainly recognize BVDV-1; BZ25 mainly recognizes BVDV-2; and BZ81 and BZ82 recognize both BVDV-2 and BVDV-3. It is unclear whether these mAbs possess neutralizing activity, and the antigenic epitopes recognized by these mAbs have not been defined. To date, however, no epitope mapping has involved BVDV-1, BVDV-2, and BVDV-3.

Pestiviruses are generally not strictly host-specific, except that CSFV infects only pigs. BDV predominantly infects small ruminants and can also infect pigs and cattle [26, 27]. In addition to cattle, BVDV can also infect sheep, pigs, deer, and camels [27, 28]; the newly discovered ovine pestivirus (ovIT PeV) was reported to infect pigs and elicit robust protection against CSFV [29]. Due to the close antigenic relationships among these viruses, serological diagnosis of the associated diseases is frequently hampered by cross-reactive antibodies. It is necessary to develop virus-specific detection methods to accurately diagnose the type of viruses infecting animals.

In this study, we report the generation and systematic characterization of five novel neutralizing mAbs against BVDV-1 E2 glycoprotein. Epitope mapping revealed that these mAbs target two spatially and functionally distinct antigenic determinants on the E2 ectodomain: one BVDV-specific, and the other highly conserved among several pestiviruses. Our findings provide novel insights into the antigenic topography of BVDV E2 and the structural basis of antigenic cross-reactivity, while also delivering validated antibody reagents and epitope targets for the development of BVDV differential diagnostic assays and next-generation E2 subunit vaccines.

## Materials and methods

### Cells, viruses, and mAbs

Madin–Darby bovine kidney (MDBK), porcine kidney-15 (PK-15), SP2/0-Ag14, and spodoptera frugiperda (Sf9) cell lines were stored in our laboratory. MDBK, PK-15, and SP2/0-Ag14 cells were cultured in minimum essential medium (MEM) or Dulbecco’s Modified Eagle Medium (DMEM) (Corning, USA) supplemented with 100 μg/mL penicillin, 100 IU/mL streptomycin sulfate (Gibco, USA), and 10% fetal bovine serum (FBS) (Gibco, USA) at 37 °C and 5% CO_2_. Sf9 cells were cultured in Grace’s insect medium and Sf-900™ II SFM (Gibco, USA) supplemented with 100 μg/mL penicillin, 100 IU/mL streptomycin sulfate (Gibco, USA), and 5% FBS at 27 °C without CO_2_, and were cultured in FBS-free medium for protein expression. Two BVDV-1a strains (NADL and JLCC1-2022) were kindly provided by Prof. Liying Zhang from Jilin University and Prof. Yanling Yang from the Institute of Special Animal and Plant Sciences, Chinese Academy of Agricultural Sciences, respectively. Three BVDV-1b strains (XH32, HY-9, and AV69) were stored in our laboratory. One BVDV-1c strain (Oregon) was provided by Dr Xingyu Li from Shandong Normal University. Two BVDV-1m strains (GS and NM-HDNFY) were isolated from bovine serum samples in our laboratory. Three BVDV-2a strains (890, SH28, and BCM16) were kindly provided by Prof. Guoqiang Zhu from Yangzhou University. CSFV strains of different subgenotypes used in this study, including two subgenotype 1.1 strains (hog cholera lapinized virus (HCLV) and Shimen strain (SM)), seven subgenotype 2.1 strains (CN021, CN069, CN033, CN049, CN057, CN063, CN068), one subgenotype 2.2 strain (CN002), and one subgenotype 2.3 strain (CN018), were stored in our laboratory [19]. The broad-spectrum mAb TCH034 against pestivirus E2 protein [30] and mAb HCL-010 specifically against CSFV E2 protein [19] were generated in our laboratory and used as positive antibody controls in this study. The mouse anti-His tag mAb was purchased from GenScript (China).

### Preparation of recombinant BVDV E2 protein

A recombinant expression plasmid pFast Bac1-BVDV-XH32-E2 was constructed based on the E2 gene sequence of BVDV-1b strain XH32 (Additional file 1). To enhance translational efficiency and secretory expression, the coding sequence for the E2 ectodomain was selected, and a Kozak sequence was inserted immediately upstream of the initiation codon. A C-terminal 6 × His tag was added to facilitate protein detection and Ni-affinity purification. The recombinant plasmid was transformed into DH10Bac competent cells to generate recombinant bacmid DNA containing the BVDV E2 gene. The resulting bacmid was then transfected into Sf9 cells to express recombinant E2 protein. Culture supernatants from the third passage of recombinant baculoviruses were harvested, and the recombinant protein was purified using Ni-carboxymethyl ethylenediamine affinity chromatography (Vazyme, China). Protein purity was assessed by sodium dodecyl sulfate-polyacrylamide gel electrophoresis (SDS-PAGE), and recombinant protein expression was verified by western blot using mouse anti-His tag mAb and TCH034 as primary antibodies, and Alexa Fluor 680-conjugated donkey anti-mouse IgG(H + L) (Invitrogen, USA) as the secondary antibody.

### Generation and isotype determination of mAbs against the E2 protein

Six-week-old female BALB/c mice were immunized intraperitoneally and subcutaneously on days 0, 7, and 21 with 50 μg of purified E2 protein emulsified in an equal volume of ISA 206VG adjuvant (SEPPIC, France). All animal experiments were conducted in accordance with protocols approved by the Animal Welfare and Ethics Committee of the Academy of Military Medical Sciences (approval no.: IACUC of AMMS-11-2025-120). The mouse with the highest antibody titer received a booster immunization of 50 μg adjuvant-free E2 protein via intraperitoneal injection 3 days before fusion. Splenocytes were isolated and fused with SP2/0 myeloma cells at a 5:1 ratio. Fused cells were selected in hypoxanthine-aminopterin-thymidine (HAT) medium. Hybridoma clones secreting anti-E2 mAbs were screened by immunofluorescence assay (IFA) using BVDV-1b XH32-infected cells as described previously [31]. Positive clones were subcloned two times, and stable antibody-secreting hybridomas were injected into BALB/c mice to produce ascites. The mAb subtype was determined using Rapid enzyme-linked immunosorbent assay (ELISA) Mouse mAb Isotyping Kit (Biodragon, China).

### Identification of the reaction patterns of mAbs with pestiviruses

IFA was used to evaluate the reactivity and specificity of anti-E2 mAbs against BVDV and CSFV. MDBK cells were infected with different strains of BVDV-1 and BVDV-2, while PK-15 cells were inoculated with CSFV strains SM, HCLV, and field isolates at a multiplicity of infection (MOI) of 0.1. After incubation at 37 °C with 5% CO_2_ for 48 h, cells were fixed and analyzed using anti-E2 mAbs as primary antibody and Alexa Fluor 488-conjugated donkey anti-mouse IgG(H + L) (Invitrogen, USA) as secondary antibody. The mAbs TCH034 and WH303 were used as positive controls for BVDV and CSFV detection, respectively.

To further characterize the reactivity spectrum of these mAbs, genes encoding E2 proteins derived from representative strains of 18 different BVDV subgenotypes were synthesized and expressed using a baculovirus expression system [30]. Western blot was performed as previously described [31] to detect the reactivity of each mAb with E2 proteins of different BVDV subgenotypes. E2 proteins from 17 representative strains covering ten different CSFV subgenotypes and nine other pestivirus species, expressed using the same system and stored in our laboratory [30, 31], were used to verify their specificity. The mouse anti-His tag mAb was used as a positive control, and Alexa Fluor 680-conjugated donkey anti-mouse IgG(H + L) was used as the secondary antibody.

### Indirect ELISA

Indirect ELISA was performed to detect the binding patterns between mAbs and antigens. E2-specific mAbs in ascites were purified using HiTrap protein A/G resin (Cytiva, China). The 96-well ELISA plates were coated with 100 μL per well of BVDV XH32 E2 protein (1.0 μg/mL) and incubated overnight at 4 °C. The plates were then washed three times with PBST and blocked with 5% BSA at 37 °C for 1 h. Serial fourfold dilutions of mAbs were added to the plates at concentrations ranging from 0.1 ng/mL to 12 μg/mL and incubated at 37 °C for 1 h. Goat anti-mouse IgG(H + L) (HRP) (Abcam, UK) was used as the detection antibody. The absorbance at 450 nm (OD_450_) was measured using a Tecan Infinite 200 multifunctional microplate reader (TECAN, Switzerland). The half maximal effective concentration (EC_50_) values were analyzed using GraphPad Prism 9.5.

### Biolayer interferometry assays

Biolayer interferometry (BLI) experiments were performed using the Octet QKe system (ForteBio, USA). Briefly, purified mAbs and XH32 E2 protein were diluted in kinetic buffer (1% bovine serum albumin (BSA) in phosphate-buffered saline with Tween-20 (PBST)). Prior to detection, the anti-mouse IgG Fc biosensor (SARTORIUS, Germany) was hydrated in the kinetic buffer for 20 min. Subsequently, mAbs diluted to 10 μg/mL were immobilized onto the sensor surface. XH32 E2 protein was first diluted to an initial concentration of 266.7 nM in kinetic buffer, followed by threefold serial dilutions to generate seven concentration gradients ranging from 266.7 to 0.37 nM; kinetic buffer without E2 protein was used as a blank control. Diluted E2 protein was then employed as the analyte to determine the binding affinities between mAbs and E2 protein. All experimental data were analyzed using Octet Data Analysis HT 10.0 software.

### Virus neutralization test

A microneutralization assay was performed to evaluate the neutralizing activity of mAbs against BVDV and CSFV as previously described [32, 33]. BVDV (XH32) and CSFV (CN049) were propagated in MDBK and PK-15 cells, respectively, and viral titers were determined. Ascites mAbs were initially diluted fivefold, followed by serial twofold dilutions in 50 μL DMEM (for BVDV) or MEM (for CSFV). Each dilution was mixed with 50 μL (100 TCID_50_) BVDV or CSFV in 96-well plates and incubated at 37 °C for 1 h. Then, 1 × 10^4^ MDBK or PK-15 cells were added to each well, respectively. After incubation at 37 °C with 5% CO_2_ for 72 h, cells were fixed to test virus expression by IFA.

To further determine the neutralizing ability of mAbs against different subgenotypes of BVDV strains, five BVDV strains, belonging to BVDV-1a, BVDV-1b, BVDV-1c, BVDV-1m, and BVDV-2a, were diluted to a titer of 2000 TCID_50_/mL using DMEM. The purified mAbs were adjusted to a concentration of 1.5 mg/mL and subjected to an initial 1:8 dilution, followed by twofold serial dilutions in DMEM. Subsequently, 50 μL of viral working suspension was mixed with an equal volume of antibody diluent in a 96-well plate and incubated at 37 °C for 1 h. Then, 100 μL of MDBK cell suspension was added to all wells of the microplate and incubated at 37 °C with 5% CO_2_. After 3 days of incubation, cells were fixed and analyzed by IFA. The neutralization titer of each mAb was defined as the highest serum dilution that inhibited virus propagation in 50% of the wells tested in duplicates (ND_50_).

### Mapping of the epitopes on glycoprotein E2

To identify the type of epitopes recognized by the mAbs, purified E2 protein was treated with or without reducing SDS loading buffer containing β-mercaptoethanol and analyzed by western blot. Chimeric constructs were generated to map the antigenic regions by reciprocal domain swapping of the E2 extracellular regions from CSFV SM and BVDV XH32 [31]. Each E2 extracellular domain was divided into four segments: A/a: residues 1–90, B/b: residues 91–170, C/c: residues 171–250, and D/d: residues 251–331. After domain substitution, chimeric E2 proteins were expressed in a baculovirus system, and their reactivity with mAbs was analyzed by western blot under nonreducing conditions. The segment recognized by mAbs was further subdivided using similar strategies to narrow the target region.

Based on the identification of epitope region, E2 amino acid (aa) sequences covering this domain derived from different BVDV and CSFV strains were aligned using MEGA 7.0 and CLC Sequence Viewer 7.6.1 software. Given their divergent binding profiles (reactive or nonreactive) against the mAbs, putative key residues responsible for the epitope were screened out for site-directed mutagenesis (SDM). To precisely identify the epitopes recognized by TBH033, mutations of the candidate sites were introduced into XH32 E2 based on plasmid pFast Bac1-BVDV-XH32-E2 using In-Fusion HD Cloning Kit (TaKaRa Bio, China). In brief, the primers with mutation and 15-bp overlaps were designed for polymerase chain reaction (PCR) amplification (Additional file 2). The In-Fusion enzyme fused the purified DNA fragments and linearized plasmid vector by recognizing the 15-bp overlaps at 50 °C for 15 min. Then the competent cell transformed with reaction mixture was plated on selective media. The positive colonies were verified by PCR and Sanger sequencing. Then the mutant E2 proteins were expressed with the insect baculovirus expression system as described in Preparation of recombinant BVDV E2 protein section. Western blot analysis was conducted to assess the binding capacity of TBH033 to mutant E2 proteins.

To further simplify the experiment, the BVDV XH32 E2 gene was codon-optimized and synthesized based on mammalian cell expression system, followed by cloning of the E2 gene into the pcDNA3.1(+) vector (Additional file 3). Referring to the above description, the series of mutant-E2 vectors based pcDNA3.1-BVDV-XH32-E2 were conducted by In-Fusion Cloning reaction for epitopes identification of TBH002, TBH004, TBH008, and TBH009. Subsequently, the recombinant plasmids were transfected into HEK-293 cells using Lipofectamine 2000 (Thermo Fisher Scientific, USA). The expressed proteins were harvested at 72 h posttransfection and analyzed by western blot.

### Biological information analysis

To assess the conservation of epitopes recognized by mAbs, E2 protein sequences from 701 different strains of CSFV, BVDV, BDV, and other pestivirus members were subjected to multiple sequence alignment using MEGA 7.0 and CLC Sequence Viewer 7.6.1 software. The conservation of the mapped epitopes was evaluated using WebLogo diagrams [34]. The three-dimensional (3D) structure of the full-length E2 protein was simulated with the SWISS-MODEL online server [35], and the spatial distribution of epitopes on the predicted model of E2 protein was visualized using PyMOL 3.1 software.

## Results

### Preparation of mAbs against BVDV E2 protein

**Figure 1:**
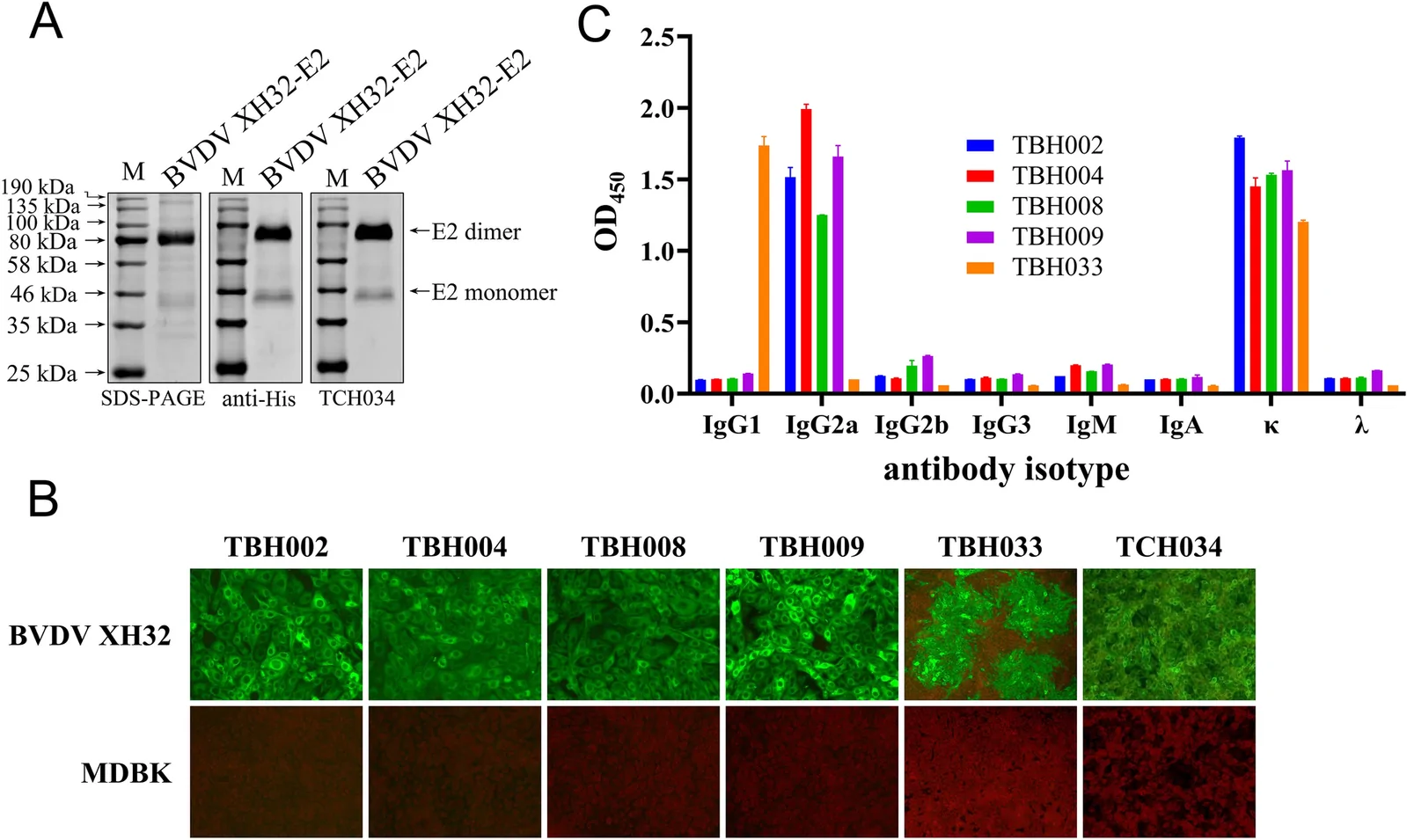
**Generation and identification of mAbs against E2 glycoprotein of BVDV.** **A** Expression and identification of recombinant E2 protein. SDS-PAGE and western blot were performed to verify the expression of E2 protein in sf9 cells. **B** IFA analysis of mAbs with BVDV XH32 infected and uninfected MDBK cells. The mAb TCH034 was used as positive control. **C** Subtype classification of mAbs was performed according to the manufacturer’s instructions. The result was read at OD_450._

The recombinant BVDV E2 protein was efficiently expressed in the insect baculovirus expression system. After affinity chromatography purification, the protein exhibited high purity and was specifically recognized by both the anti-His tag mAb and the anti-pestivirus E2 mAb TCH034 in western blot analysis (Figure 1A). Following immunization of BALB/c mice with the purified protein, the highest antibody titer in immunized mice reached 1:204 800 (data not shown). Mouse splenocytes were fused with SP2/0 cells, and five hybridoma cell lines secreting anti-BVDV E2 mAbs were successfully obtained after multiple rounds of subcloning and screening. IFA revealed that all five mAbs specifically reacted with XH32-infected MDBK cells (Figure 1B). Determination of their subtypes using a mouse mAb isotype identification kit showed that TBH002, TBH004, TBH008, and TBH009 were immunoglobulin (Ig)G2a, whereas TBH033 was IgG1. All five mAbs had a kappa light chain (Figure 1C).

### Characterization of mAb specificity

**Figure 2:**
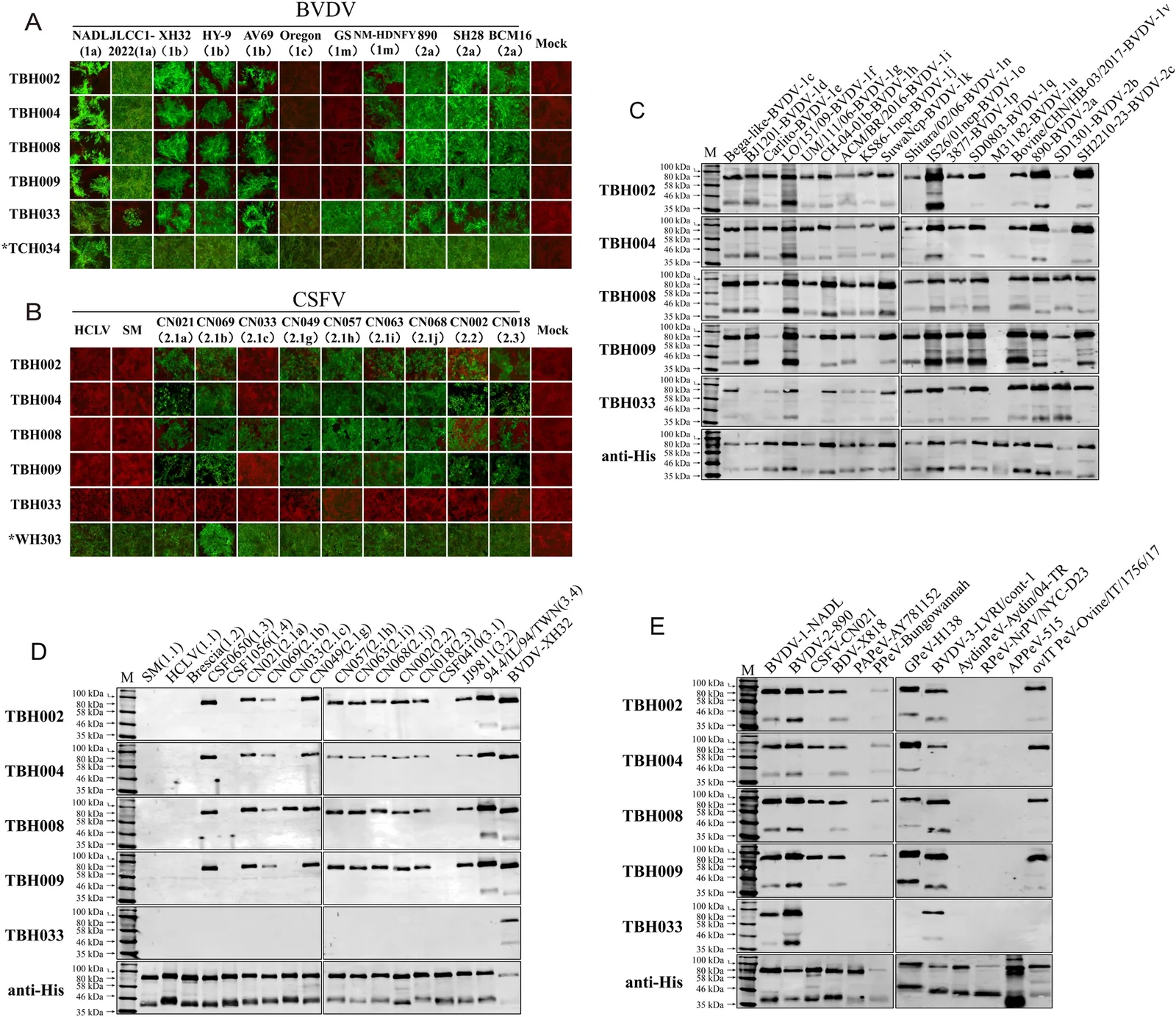
**Reaction patterns of mAbs with a panel of pestiviruses.** **A** IFA detection of BVDVs infected MDBK cells with mAbs. The mAb TCH034 was used as positive control. **B** IFA detection of CSFVs infected PK-15 cells with mAbs. The mAb WH303 was used as positive control. **C** Reactivity of mAbs with E2 proteins of different BVDV subgenotypes. E2 proteins of 18 subgenotypes (15 belonging to BVDV-1 and 3 to BVDV-2) were expressed and immunoblotted using the mAbs, with anti-His mAb serving as control. **D** Reactivity of mAbs with E2 proteins of different CSFV subgenotypes. E2 proteins of ten subgenotypes (three genotypes) were expressed and immunoblotted with the mAbs. BVDV XH32 E2 protein and anti-His mAb were used as controls. **E** Western blot analysis of mAbs with E2 proteins of different pestivirus members.

To characterize the reactivity patterns of the five mAbs, IFA was performed with five BVDV subgenotypes (BVDV-1a, 1b, 1c, 1m, and 2a) and ten CSFV subgenotypes. TBH002, TBH004, TBH008, and TBH009 reacted with all BVDV strains except Oregon (BVDV-1c) and GS (BVDV-1m), and cross-reacted with most prevalent CSFV strains but not the traditional subgenotype 1.1 strains HCLV and SM; TBH002, TBH004, and TBH009 additionally failed to react with CSFV subgenotype 2.1c. In contrast, TBH033 reacted with all BVDV strains but with no CSFV strains (Figure 2A, B).

Western blot analysis of binding to E2 proteins from 18 representative BVDV strains showed that the five mAbs reacted with all 17 E2 proteins except BVDV-1u; TBH033 additionally failed to react with BVDV-1d and BVDV-1g E2 proteins (Figure 2C). For the four CSFV cross-reactive mAbs identified by IFA, western blot verification was performed using CSFV E2 proteins from 17 representative strains of ten subgenotypes [31]. Consistent with the IFA results, TBH008, TBH002, TBH004, and TBH009 reacted with most CSFV E2 proteins but not HCLV, SM, or subtypes 1.2, 1.4, and 3.1; the latter three mAbs also failed to react with CSFV subgenotype 2.1c (Figure 2D).

Evaluation of cross-reactivity with other pestivirus members showed that TBH033 reacted only with BVDV-1, BVDV-2, and BVDV-3 E2 proteins, while the other four mAbs cross-reacted with CSFV, BDV, porcine pestivirus (PPeV), giraffe pestivirus (GPeV), and ovine/IT pestivirus (ovIT PeV) (Figure 2E).

### Determination of affinity and neutralizing activity of mAbs

**Figure 3:**
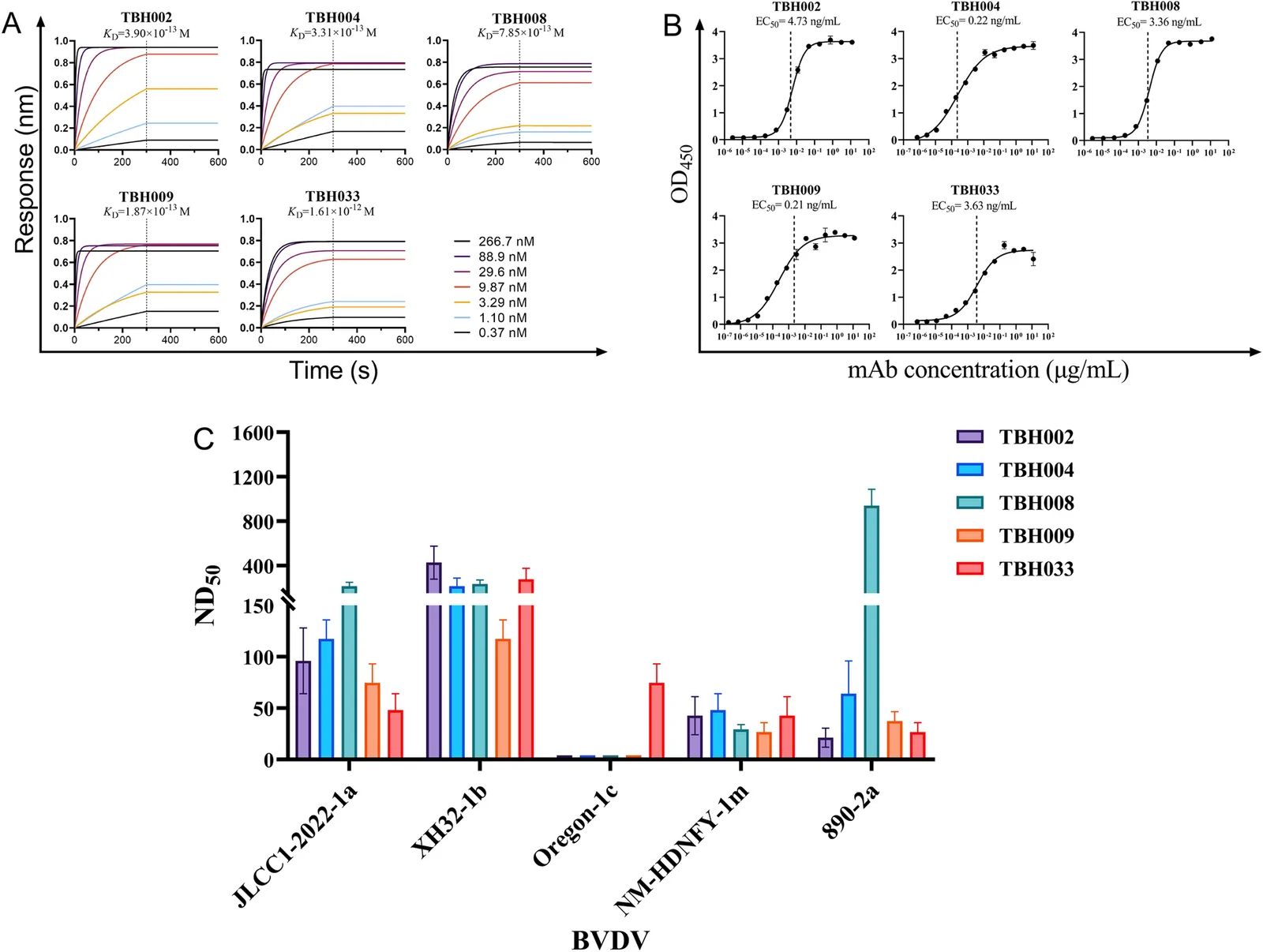
**In vitro activities of mAbs.** **A** Affinity determination of mAbs binding to BVDV XH32 E2 protein measured by BLI. Representative sensorgram traces are shown, derived from three independent biological replicates. *K*_D_ values were obtained by global fitting of binding-response data. **B** Binding of mAbs to BVDV XH32 E2 protein determined by indirect ELISA. Representative dose–response fitting curves are presented from three independent biological replicates, and corresponding EC_50_ values are annotated for each mAb. **C** Neutralizing efficacy of mAbs against different BVDV strains of various subgenotypes. Bar heights denote the mean ND_50_ values, and error bars represent the standard deviation (SD) calculated from three independent biological replicates.

The purified recombinant E2 protein of XH32 strain was used in BLI assay to assess the affinity of the five mAbs. As shown in Figure 3A, all mAbs exhibited picomolar affinities for the E2 protein of XH32 (*K*_D_ values 1.87–16.1 × 10^−13^ M). In addition, ELISA assays verified that all five mAbs exhibited robust binding to the E2 protein derived from strain XH32, with EC_50_ values below 5 ng/mL (Figure 3B).

To further assess the neutralizing activity of the mAbs, virus neutralization tests against BVDV and CSFV were conducted separately. The results showed that except for TBH009, the other four ascites mAbs could still neutralize BVDV at a dilution of 1:320. Notably, TBH002, TBH004, TBH008, and TBH009 reacted with both BVDV and CSFV, neutralized BVDV with high titers but showed no neutralizing activity against CSFV (Additional file 4). Furthermore, the five mAbs exhibited varying neutralizing potency against BVDV strains of different subgenotypes. As shown in Figure 3C, compared with NM-HDNFY (ND_50_ = 16–64), TBH002, TBH004, TBH008, and TBH009 exhibited stronger neutralization against JLCC1-2022 and XH32, with ND_50_ values ranging from 64 to 512; however, they showed little neutralizing activity against the Oregon strain (ND_50_ < 8), which was attributed to the lack of reactivity of these four mAbs with Oregon in IFA; TBH033 showed neutralizing activity against all five subgenotypes of strains, with the strongest neutralization against XH32 (ND_50_ = 192–384), followed by Oregon (ND_50_ = 64–96), and comparable neutralization against JLCC1-2022 and NM-HDNFY (ND_50_ = 32–64). For BVDV-2, all mAbs showed relatively weak neutralizing activity (ND_50_ = 16–96), except for TBH008, which showed an extremely high ND_50_ of 768–1024 against 890 strain.

Collectively, these results demonstrated robust binding and neutralizing activities of all tested mAbs against BVDV.

### Location of antigenic regions recognized by mAbs

**Figure 4:**
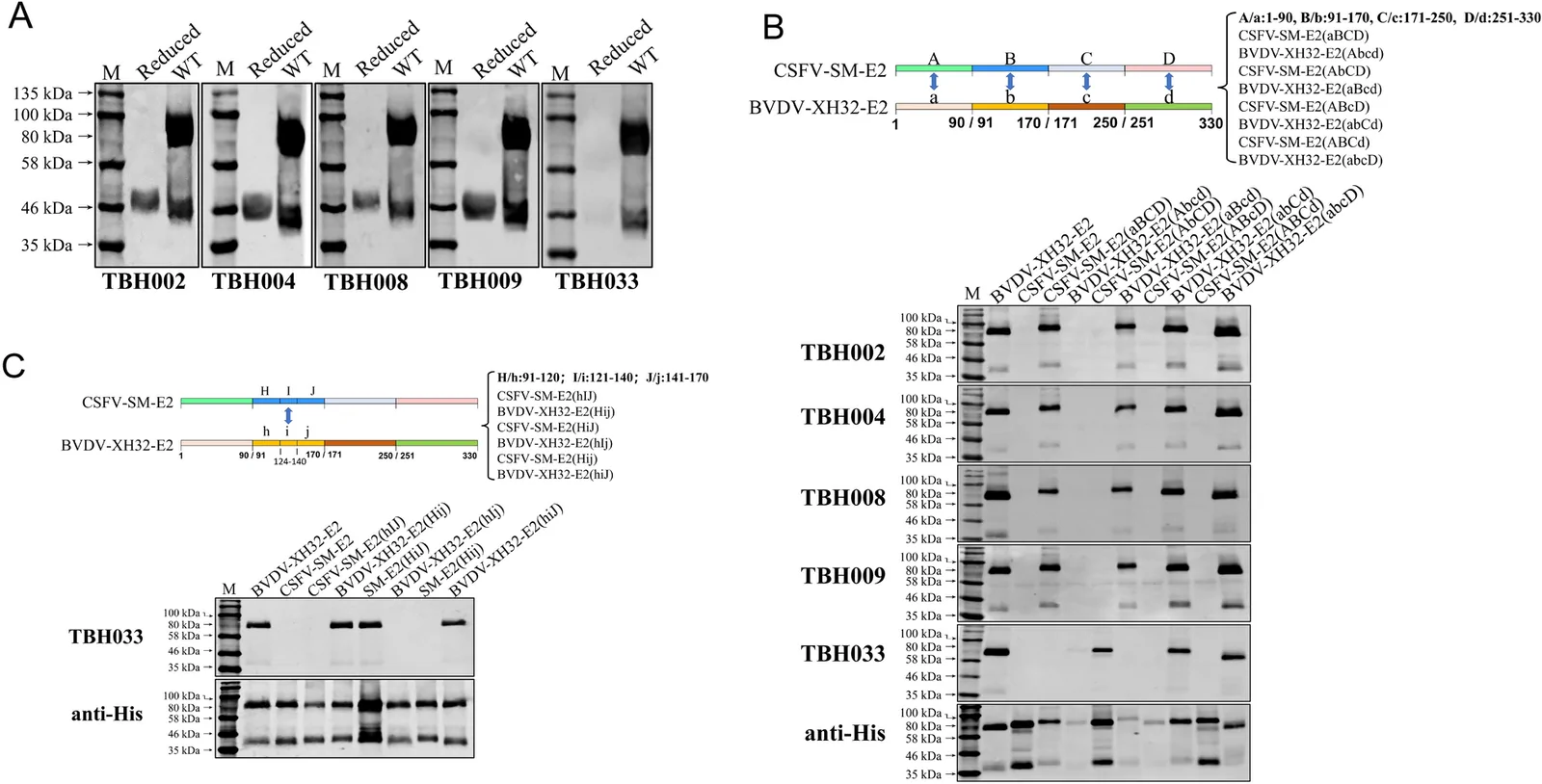
**Localization of antigenic epitope regions recognized by mAbs.** **A** Identification of epitope type recognized by mAbs under different buffer conditions, reduced: XH32 E2 protein following DTT treatment; WT: nonreduced XH32 E2 protein without DTT treatment. **B** E2 chimera with A (aa 1–90), B (aa 91–170), C (aa 171–250), and D (aa 251–330) regions respectively swapped between unreacted CSFV-SM-E2 and reacted BVDV-XH32-E2 were constructed to identify the reactive region by western blot. **C** E2 chimera with H (aa 91–120), I (aa 121–140), and J (aa 141–170) regions respectively swapped between unreacted CSFV-SM-E2 and reacted BVDV-XH32-E2 were constructed to further identify the TBH033 recognized region by western blot.

Western blot analysis was performed by incubating the mAbs with reduced and nonreduced E2 proteins to determine the types of antigenic epitopes they recognized. As shown in Figure 4A, TBH002, TBH004, TBH008, and TBH009 reacted with both reduced and nonreduced E2 proteins, while TBH033 reacted only with nonreduced E2 proteins, indicating that the former four mAbs recognized linear epitopes and TBH033 recognized a conformational epitope. Four segments of the ectodomains of CSFV SM and BVDV XH32 E2 proteins were reciprocally replaced (Figure 4B), and western blot was performed to test the reactivity of the mAbs with a series of chimeric E2 proteins. As expected, all mAbs reacted with BVDV-XH32-E2 but not with CSFV-SM-E2 protein. TBH002, TBH004, TBH008, and TBH009 reacted with chimeric CSFV-SM-E2 (aBCD), but not with chimeric BVDV-XH32-E2 (Abcd), suggesting that the regions recognized by these four mAbs are located within 1–90 aa of the BVDV E2 protein (Figure 4B). Similarly, TBH033 reacted with chimeric CSFV-SM-E2 (AbCD), but not with chimeric BVDV-XH32-E2 (aBcd), suggesting that TBH033 recognizes the 91–170 aa region of BVDV E2 protein (Figure 4B). To further define the binding regions, the aa 91–170 regions of SM and XH32 E2 proteins were divided into three parts (Figure 4C), and the results showed that TBH033 reacted exclusively with CSFV-SM-E2 (HiJ) but not with BVDV-XH32-E2 (hIj), indicating that the region recognized by TBH033 was located at aa 121–140 (Figure 4C).

### Mapping of antigenic epitopes recognized by mAbs

**Figure 5:**
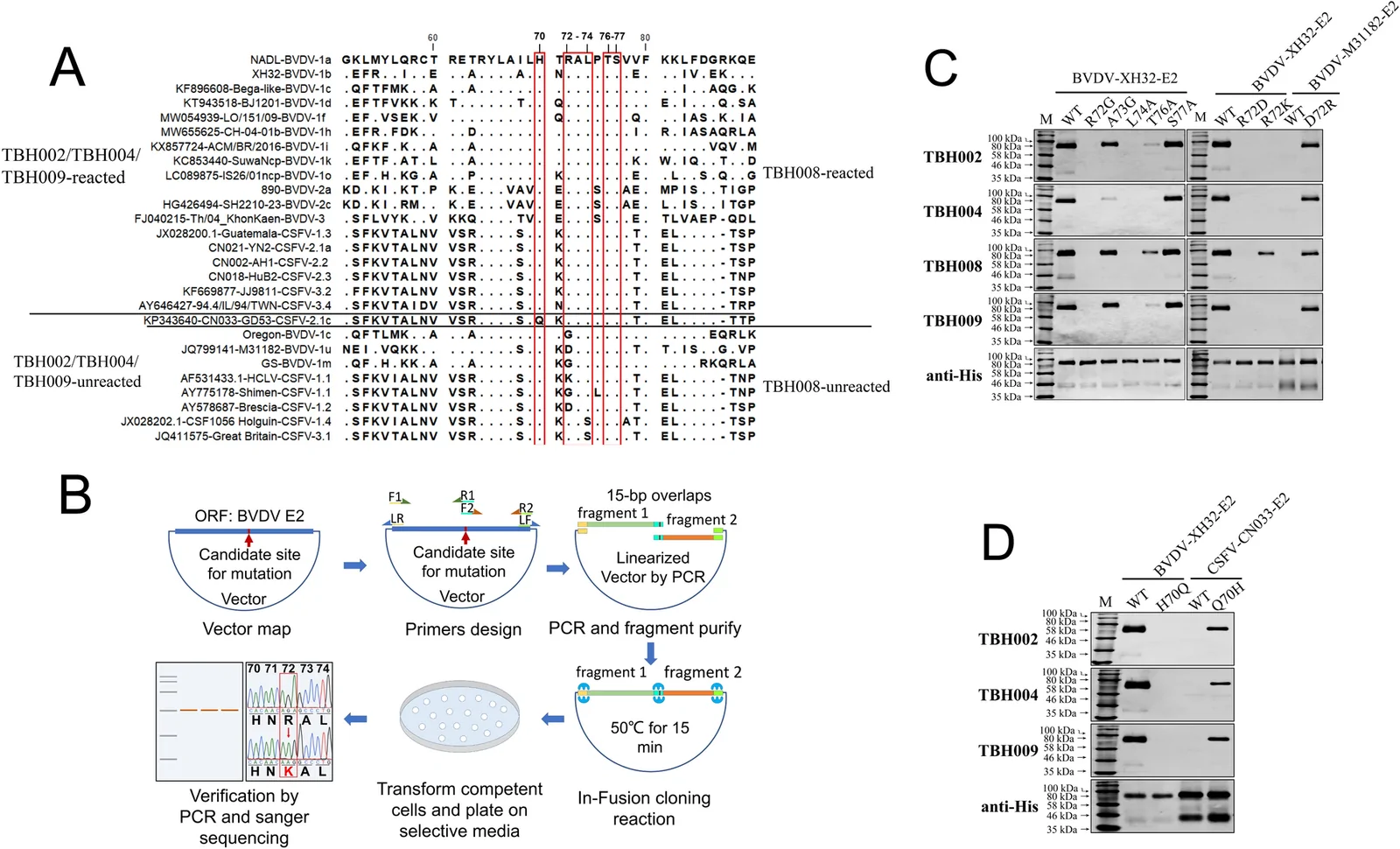
**Epitope mapping of the mAb TBH002, TBH004, TBH008, and TBH009.** **A** The amino acid sequence alignment of regions recognized by the four mAbs shows candidate sites for further site-directed mutagenesis (SDM). **B** Schematic diagram illustrating the construction of recombinant SDM plasmids using In-Fusion Cloning. **C**,**D** Reactivity of mAbs with a series of site-directed mutated E2 proteins of BVDV XH32 and unreacted strains, native E2 proteins (WT) of viruses were included as controls. The bottom panel is the normalization of E2 proteins for each lane as detected by anti-His mAb.

Binding sites of the mAbs were predicted by multiple sequence alignment of E2 aa sequences in their recognized regions. Distinct aa variations at position 72 were identified between mAb-reactive and nonreactive strains within the 1–90 aa region. Considering the flanking conserved residues, positions 72–74, 76, and 77 were selected for SDM (Figure 5A). The recombinant plasmids carrying the site-mutated E2 gene were constructed by In-Fusion Cloning technology (Figure 5B). Correct plasmids were verified through PCR and Sanger sequencing analysis (Additional file 5). The recombinant E2 was efficiently expressed in HEK-293 cells and analyzed by western blot. As shown in Figure 5C, R72G/D and L74A mutations abolished reactivity of all four mAbs (TBH002, TBH004, TBH008, and TBH009) with E2 protein, while A73G and S77A had no effect. The D72R mutation restored the reactivity of BVDV-1u strain M31182, confirming the critical role of ^72^R. The T76A mutation abolished the reactivity of TBH004 and reduced that of TBH002 and TBH009, but did not affect TBH008.

Additionally, given the lack of reactivity of TBH002, TBH004, and TBH009 with the CSFV CN033 strain, sequence alignment indicated that this lack of reactivity was attributable to the substitution at aa position 70. Western blot analysis verified this inference: the H70Q mutation abolished the reactivity of TBH002, TBH004, and TBH009 against BVDV XH32 E2, whereas the Q70H reverse mutation restored the reactivity of the three mAbs with CN033 E2 (Figure 5D). Thus, ^72^R and ^74^L were the key residues shared by the epitopes recognized by TBH008, TBH002, TBH004, and TBH009. Furthermore, ^70^H was critical for binding of the latter three mAbs, and ^76^T was also essential for binding of TBH004.

**Figure 6:**
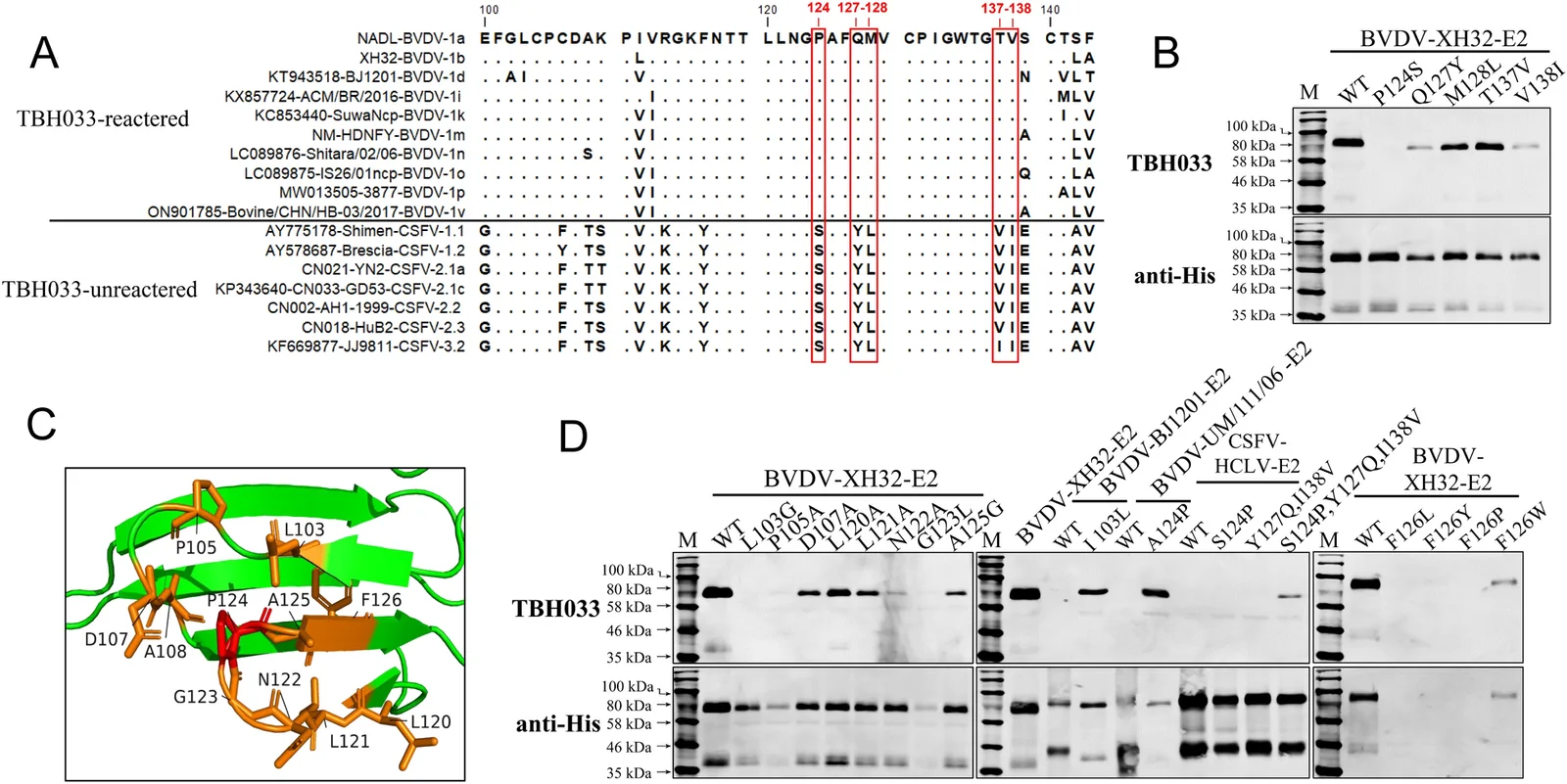
**Epitope mapping of the mAb TBH033.** **A** Multiple E2 sequence alignment of TBH033 reacted and unreacted BVDVs and CSFVs shows candidate sites for SDM within aa 121–140. **B** The critical amino acids of the epitope recognized by TBH033 were defined on BVDV XH32 E2 protein. SDM results showed that single mutation of P124S abolish the reactivity with TBH033. **C** Spatial structure analysis of amino acids surrounding the key site ^124^P. Combined with sequence alignment, nine conserved sites (103, 105, 107, 120–123, 125, 126) were selected for further mutagenesis verification. **D** Western blot analysis of the reactivity between TBH033 and E2 proteins containing mutation at selected sites.

Based on E2 sequence alignment of TBH033 reactive and nonreactive strains within the aa 121–140 region (Figure 6A), potentially differential aa at positions 124, 127, 128, 137, and 138 were initially selected for mutagenesis. Western blot analysis showed that the P124S mutation abolished TBH033 binding to the E2 protein, while Q127Y and V138I mutations reduced its reactivity, identifying ^124^P as a critical residue within the TBH033 epitope (Figure 6B). According to structural analysis and sequence alignment, nine conserved residues (103, 105, 107, 120–123, 125, and 126) were further mutagenized. Mutations L103G, P105A, and G123L abolished TBH033 reactivity, whereas reverse mutations I103L and A124P restored binding to E2 from BVDV-1d (BJ1201) and BVDV-1g (UM/111/06) strains (Figure 6C, D). Accordingly, the motifs ^103^LCP^105^, ^123^GP^124^ were identified as the core determinants of the TBH033 antigenic epitope. Notably, neither the single S124P mutation nor the double Y127Q/I138V mutations conferred TBH033 reactivity toward the originally nonreactive HCLV-E2; only simultaneous mutations at all three sites restored binding (Figure 6D).

### Structural and conservation analysis of the identified epitopes

**Figure 7:**
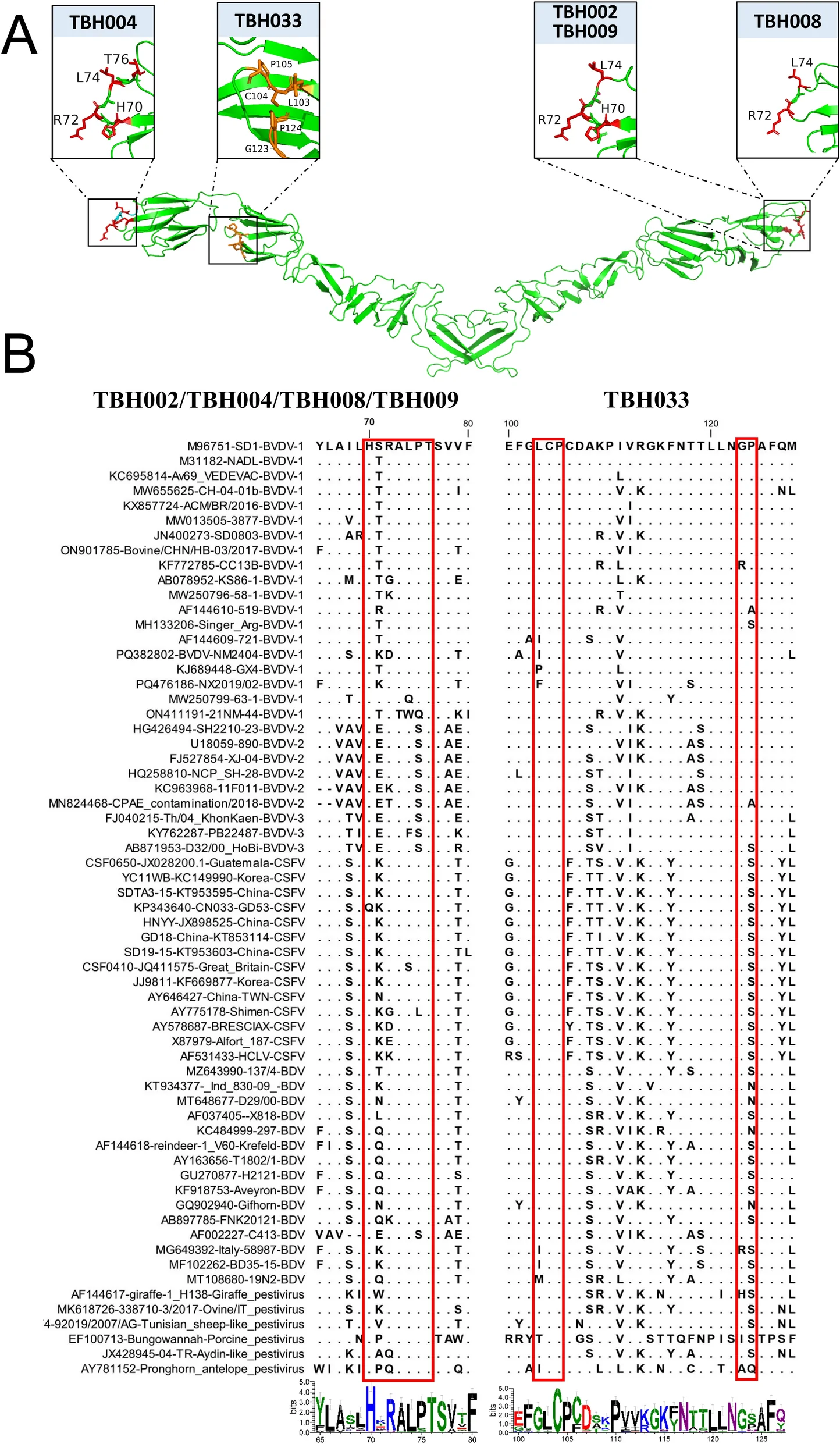
**Spatial location and conservation analysis of the epitope recognized by mAbs.** **A** The 3D structure of the BVDV XH32 E2 protein. Epitopes recognized by mAbs characterized in this study were mapped to the XH32 E2 protein. **B** Multiple sequence alignment between different pestiviruses with the identified epitopes highlighted by red frames. WebLogo presentation shows the epitope variability.

The 3D structure of the BVDV XH32 E2 protein was modeled and is shown in Figure 7A. PyMOL visualization revealed that the linear epitope recognized by TBH002, TBH004, TBH008, and TBH009 is exposed on the surface of domain DA of E2 protein, while the conformational epitope recognized by TBH033 is located on the surface of domain DB; each aa constituting the epitope is located within a random coil structure (Figure 7A). The spatial structures of these epitopes indicate that they can be recognized by humoral immunity and provide theoretical support for their future use in the development of antigenic or antibody diagnostic reagents.

To evaluate the conservation of the mapped epitopes, a total of 701 classical and field strains, including 251 BVDV-1, 162 BVDV-2, 30 BVDV-3, 43 BDV, and 215 CSFV strains, were collected, and their E2 proteins sequences were aligned using MEGA 7.0 software. Sequence alignment of representative strains is shown in Figure 7B. The critical aa ^70^H, ^72^R, ^74^L, and ^76^T, which constitute the epitopes recognized by TBH002, TBH004, TBH008, and TBH009, are highly conserved across most pestiviruses: 243/251 (96.81%) BVDV-1 strains, 160/162 (98.77%) BVDV-2 strains, 29/30 (96.67%) BVDV-3 strains, 42/43 (97.67%) BDV strains, and 202/215 (93.95%) CSFV strains retained the intact motif. The main substitutions were R72G/D/K and L74Q/W in BVDV-1; R72K/T in BVDV-2; L74F in BVDV-3; R72K in BDV; H70Q, R72G/D/E/K, and L74S in CSFV; and R72Q in other pestiviruses, which were identified by western blot as nonreactive with the mAbs (Figure 7B; Table 1). In contrast, the epitope ^103^LCP^105^, ^123^GP^124^, recognized by TBH033, exhibited a species-specific pattern and was highly conserved in BVDV-1 (226/251, 90.04%), BVDV-2 (161/162, 99.38%), and BVDV-3 (29/30, 96.67%), with only a few substitutions, such as L103I/P/F, G123R, and P124S/A in BVDV-1; P124A in BVDV-2; and P124S in BVDV-3. However, this epitope was significantly distinct from that of other pestivirus members. This divergence was primarily attributable to the mutation at residue ^124^P, whereas the remaining aa positions were relatively conserved (Figure 7B; Table 1).

**Table 1:** **Conservation analysis of the epitope recognized by mAbs**

Virus	Epitope 1: ^70^HXRXLXT^76^		Epitope 2: ^103^LCP^105^, ^123^GP^124^	
	Proportion of strains with conserved epitope^a^	Amino acid substitution sites^b^	Proportion of strains with conserved epitope^a^	Amino acid substitution sites^b^
BVDV-1	243/251	R72G/D/K; L74Q/W	226/251	L103I/P/F; G123R; P124S/A
BVDV-2	160/162	R72K/T	161/162	P124A
BVDV-3	29/30	L74F	29/30	P124S
BDV	42/43	R72K	2/43	L103I/M; G123R; P124S/N
CSFV	202/215	H70Q; R72G/D/E/K; L74S	0/215	P124S

^a^Epitope conservation across viruses was defined as the proportion of strains harboring a conserved epitope among all strains analyzed

^b^Amino acid substitution sites were denoted as the consensus amino acid residue at specific positions, as opposed to the differential residue(s)

## Discussion

BVDV remains a persistent, economically devastating pathogen of global cattle industries, and integrated strategies combining vaccination and precise antigen/antibody screening are the cornerstone of herd-level BVDV eradication programs. The present study generated a panel of five high-affinity neutralizing mAbs targeting the immunodominant envelope glycoprotein E2 of BVDV-1b strain XH32, with two distinct reactivity phenotypes: four pan-pestivirus cross-reactive mAbs (TBH002, TBH004, TBH008, TBH009) and one strictly BVDV-specific mAb (TBH033). Beyond providing dual-purpose antibody reagents for differential pestivirus diagnosis, systematic epitope mapping of these mAbs further dissects the molecular and structural basis of E2 antigenicity, delivers two well-defined neutralizing epitopes, and offers rational targets for next-generation broad-spectrum or marker differentiating infected from vaccinated animals (DIVA) subunit vaccines.

The pan-pestivirus cross-reactivity of TBH002, TBH004, TBH008, and TBH009 was confirmed by comprehensive reaction profiling across 22 BVDV subgenotypes, 10 CSFV subgenotypes, and eight heterologous pestivirus species. Their shared epitope localized within residues 1–90 of the E2 extracellular domain (DA domain), a region corresponding to the B/C antigenic region of CSFV E2. Subsequent SDM pinpointed four functionally critical residues spanning positions 70–76: ^70^H, ^72^R, ^74^L, and ^76^T. Among these, ^72^R and ^74^L represent absolutely conserved core residues shared by all four mAbs; substitutions R72G/D/K and L74A completely abolished mAb binding to E2 protein, explaining the loss of reactivity against HCLV (R72K) and SM (R72G), as well as two BVDV strains Oregon (BVDV-1c) and GS (BVDV-1m) strains carrying R72G mutations. Interestingly, R72K mutation has no effect on recognition of TBH008 (Figure 5C); even though HCLV retains ^72^K and ^74^L, it remains unreactive to TBH008, indicating that interspecies structural divergence of E2 and additional uncharacterized amino acid polymorphisms jointly interfere with mAb docking. This hypothesis is supported by mutational reverse validation: D72R substitution restored mAb reactivity to the naturally nonreactive BVDV-1u strain M31182, while G72R mutation in CSFV SM E2 failed to rescue binding (Additional file 6), confirming that single-site restoration of ^72^R cannot overcome broader interspecies structural barriers.

Early research by Paton et al. described residues 71–74 of BVDV-1 E2 as an immunodominant region [24]. Deregt et al. identified two neutralizing anti-E2 mAbs (157 and 348) targeting this epitope region. mAb 157 proved to be BVDV-1-specific, targeting a discontinuous epitope at amino acid position 9, 32, and 72; mAb 348 cross-neutralized BVDV-1 and BVDV-2, with substitutions at ^71^E, ^72^R, ^74^L, or ^77^S impairing both binding and neutralization [3]. Here, four of five mAbs obtained in our study also target this conserved DA domain, yet define an extended epitope landscape with the novel regulatory residue ^70^H. This represents a key novel finding expanding the known functional boundaries of this immunodominant epitope.

Residue ^70^H represents a previously unreported determinant governing mAb recognition and serves as the differential residue between TBH008 and TBH002/TBH004/TBH009. This histidine is highly conserved across all pestiviruses and acts as a pH sensor that mediates E2 conformational rearrangement during viral membrane fusion via protonation-induced postfusion stabilization [16, 36]. Given that mAbs TBH002, TBH004, TBH008, and TBH009 confer neutralizing activity against diverse BVDV strains, a plausible neutralization mechanism is that these broad-spectrum mAbs bind to this conserved linear motif and interfere with pH-dependent structural transitions of E2, thereby inhibiting viral membrane fusion. Notably, TBH008 retains potent neutralizing capacity, particularly against BVDV-2 strain 890, despite its failure to recognize residue ^70^H. This observation indicates that neutralization can also be mediated by other residues within the epitope; therefore, an alternative mechanism whereby these mAbs block virus-receptor engagement cannot be excluded. Further experiments, including receptor-binding inhibition assays and conformational probing under acidic conditions, are required to precisely define their mode of neutralization.

It is worth noting that Deregt et al. demonstrated that mAbs 157 and 348 did not react with CSFV, leading to the speculation that the 71–74 immunodominant region and position 72 are less critical for CSFV recognition than for BVDV [3, 37]. Our results refine this model: while TBH002, TBH004, TBH008, and TBH009 also failed to react with CSFV 1.1 strains, they cross-recognize nearly all other circulating CSFV subgenotypes, all of which retain conserved ^72^R. Similarly, our prior work characterized one mAb, HCL-018, recognizing epitope ^69^LDXGXLLT^76^ on CSFV E2 [19]; HCL-018 potently neutralizes CSFV SM but shows no cross-reactivity with BVDV. Mutation G72R abrogated its binding to SM, while K72G restored reactivity to HCLV, indicating that ^72^G is critical for SM recognition. These results highlight antigenic divergence among BVDV and CSFV and the significance of ^72^R/G for recognition of CSFV. Furthermore, beyond BVDV and CSFV, these four mAbs cross-react with BDV, PPeV, GPeV, and ovIT PeV—genetically related pestiviruses with overlapping host ranges in ruminants and swine [38, 39]. This finding confirms that the linear 70–76 motif targeted by TBH002, TBH004, TBH008, and TBH009 constitutes a genus-conserved epitope and elucidates the molecular basis for broad antigenic cross-reactivity within the genus *Pestivirus*.

To date, only limited number of broad-spectrum epitopes across pestiviruses have been identified. The antigenic epitope ^307^YYEP^310^ recognized by mAb 4-9D4 was found to be highly conserved in the E2 protein of pestiviruses, including CSFV, BVDV-1, and BDV [40]. However, this epitope became accessible only after antigen treatment with 1% NP-40, limiting the application of mAb 4-9D4 in antigen and antibody detection. Our group previously identified two broad-spectrum neutralizing mAbs (TCH034, TCH052) targeting a separate conserved E2 motif (^113^G, ^114^K, ^133^W, ^135^G) across eight pestivirus species [30]. Whether this epitope is immunodominant remains to be further analyzed to verify its applicability in developing a blocking ELISA for serological antibody detection. The epitopes targeted by mAbs TBH002, TBH004, TBH008, and TBH009 are exposed on the surface of the E2 protein and is highly conserved among all major pestivirus members, making it a superior candidate for universal pan-pestivirus antigen capture and serological screening reagents.

TBH033 is the only mAb within our panel with exclusive BVDV reactivity, a trait driven by three functionally significant properties: (1) high binding affinity (K_D_ = 1.61 × 10^−^^12^ M, EC_50_ = 3.63 ng/mL) and potent neutralizing activity (ND_50_ = 32–384) against BVDV; (2) high specificity for BVDV, with no cross-reactivity with CSFV or other pestiviruses; and (3) broad cross-reactivity with BVDV-1 (15 subgenotypes), BVDV-2 (3 subgenotypes), and BVDV-3. Epitope mapping via reduced/nonreduced western blot confirmed that TBH033 recognizes a disulfide-dependent conformational epitope localized to residues 91–170 within E2 domain DB; to our knowledge, this is the first neutralizing conformational epitope ever mapped to the DB antigenic region of BVDV E2. Chimeric domain swapping and SDM resolved two core structural motifs governing mAb binding: ^103^LCP^105^ and ^123^GP^124^. Single substitution P124S fully abolished TBH033 binding, while Q127Y and V138I only partially reduced reactivity, identifying ^124^P as the primary species-discriminant residue.

Large-scale sequence alignment of 701 global pestivirus strains (251 BVDV-1, 162 BVDV-2, 30 BVDV-3, 43 BDV, 215 CSFV) revealed extremely high conservation of the TBH033 epitope across all BVDV species: 90.04% of BVDV-1, 99.38% of BVDV-2, and 96.67% of BVDV-3 isolates carry intact ^103^LCP^105^, ^123^GP^124^ motifs. The residue ^124^P represents the most prominent molecular difference, with nearly all non-BVDV pestiviruses encoding serine at this position, providing a definitive molecular marker to serologically distinguish BVDV from other pestiviruses. Notably, single P124S reversion was insufficient to restore TBH033 binding to the nonreactive HCLV; simultaneous reverse mutations at ^124^P, ^127^Q, and ^138^V were required to recover reactivity. One plausible mechanism is that the Q127Y and V138I mutations attenuate TBH033 reactivity with BVDV E2 (Figure 6B), thus, the combined effect of these three mutations in HCLV may switch reactivity from reduced binding to complete nonreactivity, indicating that residues ^127^Q and ^138^V do not form the core epitope recognized by TBH033 but instead modulate mAb binding.

The mAb panel and mapped epitopes deliver two primary veterinary applications. First, dual-antibody differential diagnostic platforms: TBH002, TBH004, TBH008, and TBH009 can be incorporated into pan-pestivirus antigen capture ELISAs or IFA kits for broad screening of aborted fetuses, persistently infected calves, and contaminated fetal bovine serum. Subsequent testing with TBH033 enables definitive confirmation of BVDV infection to eliminate cross-reactive false positives caused by CSFV or BDV co-circulation. Second, epitope-targeted vaccine design: the highly conserved DA linear epitope provides a minimal neutralizing antigen motif for broad-spectrum pan-pestivirus subunit vaccines, while the BVDV-specific DB conformational epitope recognized by TBH033 can be engineered as a negative marker to construct DIVA vaccines, allowing for serological discrimination between naturally infected and E2-vaccinated cattle.

## Supplementary Information

**Additional file 1. Experimental proofs of pFast Bac1-BVDV-XH32-E2 construction.**

**Additional file 2. Full information of all recombinant plasmids expressing site-mutated E2, with specific mutagenesis primers and primers for mutagenesis verification listed in the table.**

**Additional file 3. Plasmid map and Sanger sequencing chromatograms for pcDNA3.1-BVDV-XH32-E2.**

**Additional file 4. Evaluation of neutralizing activity of ascitic mAbs against BVDV and CSFV. **Ascitic mAbs (TBH002, TBH004, TBH008, TBH009, TBH033) were initially 5-fold diluted, followed by serial 2-fold dilutions. Diluted mAbs were incubated with an equal volume of 100 TCID₅₀ BVDV XH32 or CSFV CN049 at 37°C for 1 h. The antibody-virus mixtures were then added to MDBK cells, and cultured for 72 h. Cells were fixed with precooled 80% acetone and subjected to IFA. As a result, except for TBH009, the remaining four ascitic mAbs retained neutralizing capacity against BVDV XH32 at a dilution of 1:320. For CSFV CN049, TBH002, TBH004, TBH008 and TBH009 failed to neutralize viral infection even at the 1:5 dilution.

**Additional file 5. Representative colony PCR validation results for recombinant plasmids expressing site-mutated E2, as well as corresponding Sanger sequencing chromatograms.**

**Additional file 6. Reactivity of mAb TBH008 with E2 proteins bearing mutation at aa position 72 of BVDV XH32 and CSFV SM.** The residue ^72^R of BVDV-XH32-E2 was mutated to D and K, respectively, while residue ^72^G of CSFV-SM-E2 was mutated to R. Position-72 substituted E2 variants were transiently expressed in HEK-293 cells, and recombinant proteins harvested from culture supernatants were analyzed by western blot. As a result, mAb TBH008 reacted with wild-type BVDV-XH32-E2 but not wild-type CSFV-SM-E2. The R72D mutation in BVDV-XH32-E2 completely abrogated TBH008 binding, whereas the R72K mutation had no effect on TBH008 recognition. In parallel, the G72R mutation in CSFV-SM-E2 failed to restore binding reactivity to TBH008.

## Data Availability

The corresponding data are all presented in this article, and the remaining materials or reagents are available from the authors upon reasonable request.
