# Supplementary material for: Generation and epitope mapping of two distinct neutralizing mAb subsets targeting BVDV E2: a pan-pestivirus cross-reactive linear epitope and a BVDV-specific conformational epitope
Source: Vet Res. 2026 Oct 7;57:176. doi: 10.1186/s13567-026-01852-5 (PMC13644284; doi:10.1186/s13567-026-01852-5)

**pFast Bac1-BVDV-XH32-E2**

The BVDV-XH32-E2 gene (1137 bp) was amplified from the cDNA of BVDV-XH32 strain. The E2 fragment with 6×His Tag at the C-terminal was inserted into the *Bam*H I/*Eco*R I site of pFast Bac1 vector by In-Fusion cloning technology. And the insert was verified using sequencing primers: poly-F-AAATGATAACCATCTCGC, SV40pA-R-GAAATTTGTGATGCTATTGC.

The following are the electrophoresis, plasmid map, complete sequence and Sanger sequencing chromatograms of the pFast Bac1-BVDV-XH32-E2.

**1.** Construction of pFast Bac1-BVDV-XH32-E2 ........................................ 1

**2.** Plasmid map of pFast Bac1-BVDV-XH32-E2 vector. ............................ 2

**3.** Complete sequence of pFast Bac1-BVDV-XH32-E2 vector. .................. 3

**4.** Sanger sequencing chromatograms of pFast Bac1-BVDV-XH32-E2 vector for inserts.

(1) pFast Bac1-BVDV-XH32-E2_Poly-F.............................................. 9

(2) pFast Bac1-BVDV-XH32-E2_SV40-pA-R....................................... 11

1. Construction of pFast Bac1-BVDV-XH32-E2

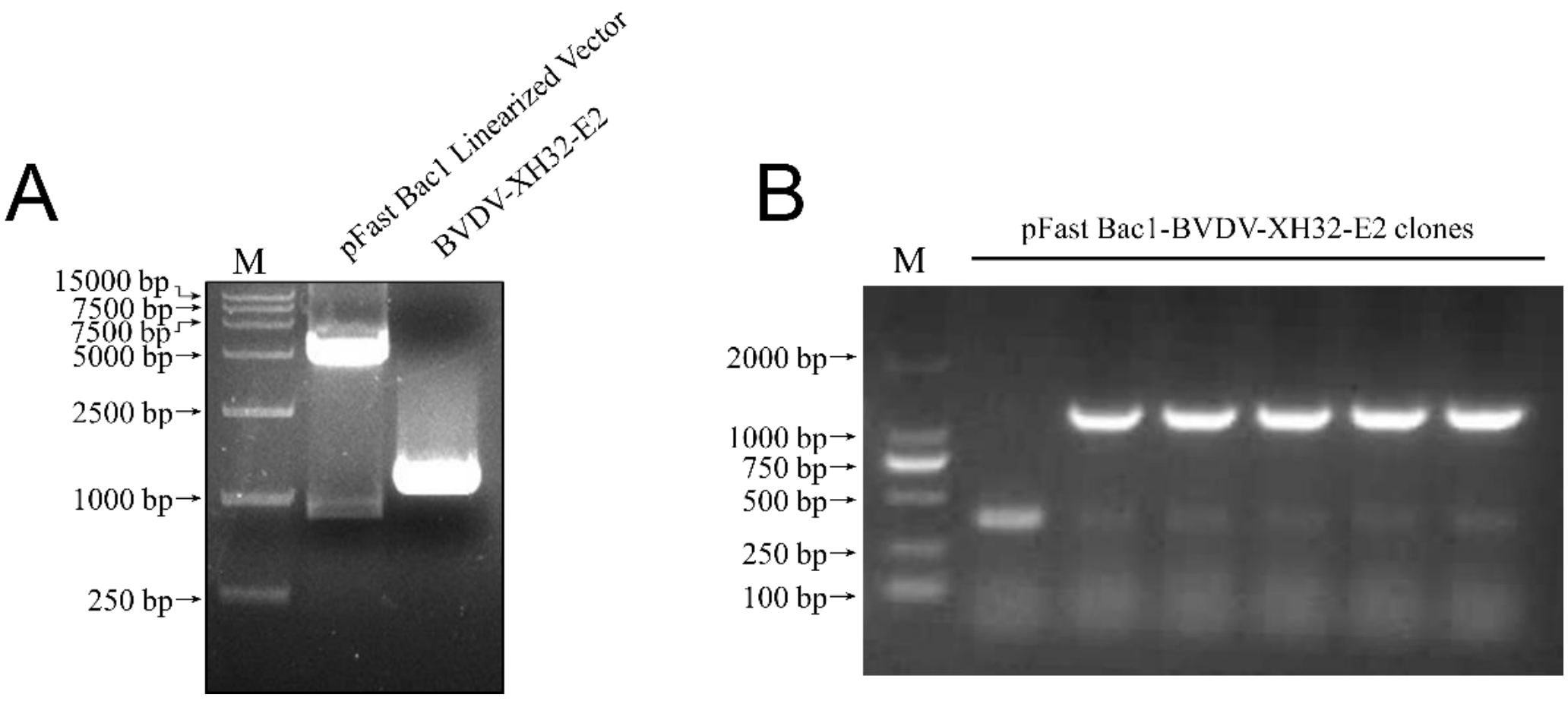


A. Linearization of pFast Bac1 vector and amplification of BVDV-XH32-E2 gene by PCR.

B. PCR Verification of pFast Bac1-XH32-E2 clones.

Sequence: pFastBac1-BVDV-XH32-E2 .dna (Circular / 5879 bp)
Enzymes: < Unsaved Enzyme Set > (7 of 678 total)
Features: 14 total
Primers: 6 total

# 2. Plasmid map of pFast Bac1-BVDV-XH32-E2

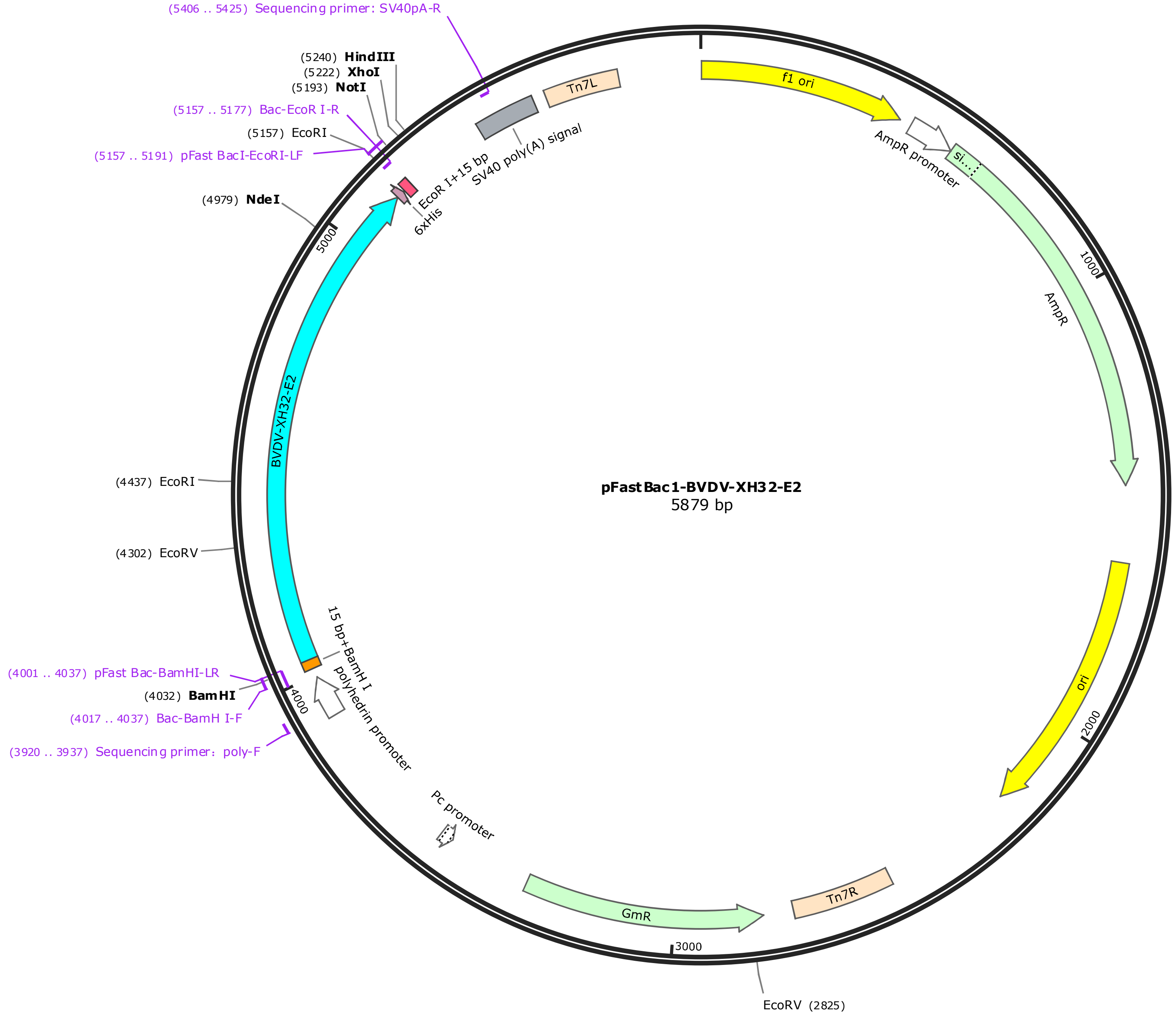

# 3. Complete sequence of pFast Bac1-BVDV-XH32-E2

```
5' GACGCGCCCTGTAGCGGCGCATTAAGCGCGGCGGGTGTGGTGGTTACGCGCAGCGTGACCGCTACACTTGCCAGCGCCCTAGCGCCCGCTCCTTT 95
3' CTGCGCGGGACATCGCCGCGTAATTCGCGCCGCCCACACCACCAATGCGCGTCGCACTGGCGATGTGAACGGTCGCGGGATCGCGGGCGAGGAAA
f1 ori

CGCTTTCTTCCCTTCCTTTCTCGCCACGTTCGCCGGCTTTCCCCGTCAAGCTCTAAATCGGGGGCTCCCTTTAGGGTTCCGATTTAGTGCTTTAC 190
GCGAAAGAAGGGAAGGAAAGAGCGGTGCAAGCGGCCGAAAGGGGCAGTTCGAGATTTAGCCCCCGAGGGAAATCCCAAGGCTAAATCACGAAATG
f1 ori

GGCACCTCGACCCCAAAAAACTTGATTAGGGTGATGGTTCACGTAGTGGGCCATCGCCCTGATAGACGGTTTTTCGCCCTTTGACGTTGGAGTCC 285
CCGTGGAGCTGGGGTTTTTTGAACTAATCCCACTACCAAGTGCATCACCCGGTAGCGGGACTATCTGCCAAAAAGCGGGAAACTGCAACCTCAGG
f1 ori

ACGTTCTTTAATAGTGGACTCTTGTTCCAAACTGGAACAACACTCAACCCTATCTCGGTCTATTCTTTTGATTTATAAGGGATTTTGCCGATTTC 380
TGCAAGAAATTATCACCTGAGAACAAGGTTTGACCTTGTTGTGAGTTGGGATAGAGCCAGATAAGAAAACTAAATATTCCCTAAAACGGCTAAAG
f1 ori

GGCCTATTGGTTAAAAAATGAGCTGATTTAACAAAAATTTAACGCGAATTTTAACAAAATATTAACGTTTACAATTTCAGGTGGCACTTTTCGGG 475
CCGGATAACCAATTTTTTACTCGACTAAATTGTTTTTAAATTGCGCTTAAAATTGTTTTATAATTGCAAATGTTAAAGTCCACCGTGAAAAGCCC
f1 ori

GAAATGTGCGCGGAACCCCTATTTGTTTATTTTTCTAAATACATTCAAATATGTATCCGCTCATGAGACAATAACCCTGATAAATGCTTCAATAA 570
CTTTACACGCGCCTTGGGGATAAACAAATAAAAAGATTTATGTAAGTTTATACATAGGCGAGTACTCTGTTATTGGGACTATTTACGAAGTTATT
AmpR promoter

TATTGAAAAAGGAAGAGTATGAGTATTCAACATTTCCGTGTCGCCCTTATTCCCTTTTTTGCGGCATTTTGCCTTCCTGTTTTTGCTCACCCAGA 665
ATAACTTTTTCCTTCTCATACTCATAAGTTGTAAAGGCACAGCGGGAATAAGGGAAAAAACGCCGTAAAACGGAAGGACAAAAACGAGTGGGTCT
M S I Q H F R V A L I P F F A A F C L P V F A H P E
AmpR promoter   signal sequence
AmpR

AACGCTGGTGAAAGTAAAAGATGCTGAAGATCAGTTGGGTGCACGAGTGGGTTACATCGAACTGGATCTCAACAGCGGTAAGATCCTTGAGAGTT 760
TTGCGACCACTTTCATTTTCTACGACTTCTAGTCAACCCACGTGCTCACCCAATGTAGCTTGACCTAGAGTTGTCGCCATTCTAGGAACTCTCAA
T L V K V K D A E D Q L G A R V G Y I E L D L N S G K I L E S
AmpR

TTCGCCCCGAAGAACGTTTTCCAATGATGAGCACTTTTAAAGTTCTGCTATGTGGCGCGGTATTATCCCGTATTGACGCCGGGCAAGAGCAACTC 855
AAGCGGGGCTTCTTGCAAAAGGTTACTACTCGTGAAAATTTCAAGACGATACACCGCGCCATAATAGGGCATAACTGCGGCCCGTTCTCGTTGAG
F R P E E R F P M M S T F K V L L C G A V L S R I D A G Q E Q L
AmpR

GGTCGCCGCATACACTATTCTCAGAATGACTTGGTTGAGTACTCACCAGTCACAGAAAAGCATCTTACGGATGGCATGACAGTAAGAGAATTATG 950
CCAGCGGCGTATGTGATAAGAGTCTTACTGAACCAACTCATGAGTGGTCAGTGTCTTTTCGTAGAATGCCTACCGTACTGTCATTCTCTTAATAC
G R R I H Y S Q N D L V E Y S P V T E K H L T D G M T V R E L C
AmpR

CAGTGCTGCCATAACCATGAGTGATAACACTGCGGCCAACTTACTTCTGACAACGATCGGAGGACCGAAGGAGCTAACCGCTTTTTTGCACAACA 1045
GTCACGACGGTATTGGTACTCACTATTGTGACGCCGGTTGAATGAAGACTGTTGCTAGCCTCCTGGCTTCCTCGATTGGCGAAAAAACGTGTTGT
S A A I T M S D N T A A N L L L T T I G G P K E L T A F L H N
AmpR
```

TGGGGGATCATGTAACTCGCCTTGATCGTTGGGAACCGGAGCTGAATGAAGCCATACCAAACGACGAGCGTGACACCACGATGCCTGTAGCAATG 1140
ACCCCCTAGTACATTGAGCGGAACTAGCAACCCTTGGCCTCGACTTACTTCGGTATGGTTTGCTGCTCGCACTGTGGTGCTACGGACATCGTTAC
155 160 165 170 175 180
M G D H V T R L D R W E P E L N E A I P N D E R D T T M P V A M
AmpR

GCAACAACGTTGCGCAAACTATTAACTGGCGAACTACTTACTCTAGCTTCCCGGCAACAATTAATAGACTGGATGGAGGCGGATAAAGTTGCAGG 1235
CGTTGTTGCAACGCGTTTGATAATTGACCGCTTGATGAATGAGATCGAAGGGCCGTTGTTAATTATCTGACCTACCTCCGCCTATTTCAACGTCC
185 190 195 200 205 210 215
A T T L R K L L T G E L L T L A S R Q Q L I D W M E A D K V A G
AmpR

ACCACTTCTGCGCTCGGCCCTTCCGGCTGGCTGGTTTATTGCTGATAAATCTGGAGCCGGTGAGCGTGGGTCTCGCGGTATCATTGCAGCACTGG 1330
TGGTGAAGACGCGAGCCGGGAAGGCCGACCGACCAAATAACGACTATTTAGACCTCGGCCACTCGCACCCAGAGCGCCATAGTAACGTCGTGACC
220 225 230 235 240 245
P L L R S A L P A G W F I A D K S G A G E R G S R G I I A A L
AmpR

GGCCAGATGGTAAGCCCTCCCGTATCGTAGTTATCTACACGACGGGGAGTCAGGCAACTATGGATGAACGAAATAGACAGATCGCTGAGATAGGT 1425
CCGGTCTACCATTCGGGAGGGCATAGCATCAATAGATGTGCTGCCCCTCAGTCCGTTGATACCTACTTGCTTTATCTGTCTAGCGACTCTATCCA
250 255 260 265 270 275
G P D G K P S R I V V I Y T T G S Q A T M D E R N R Q I A E I G
AmpR

GCCTCACTGATTAAGCATTGGTAACTGTCAGACCAAGTTTACTCATATATACTTTAGATTGATTTAAAACTTCATTTTTAATTTAAAAGGATCTA 1520
CGGAGTGACTAATTCGTAACCATTGACAGTCTGGTTCAAATGAGTATATATGAAATCTAACTAAATTTTGAAGTAAAAATTAAATTTTCCTAGAT
280 285
A S L I K H W *
AmpR

GGTGAAGATCCTTTTTGATAATCTCATGACCAAAATCCCTTAACGTGAGTTTTCGTTCCACTGAGCGTCAGACCCCGTAGAAAAGATCAAAGGAT 1615
CCACTTCTAGGAAAAACTATTAGAGTACTGGTTTTAGGGAATTGCACTCAAAAGCAAGGTGACTCGCAGTCTGGGGCATCTTTTCTAGTTTCCTA

CTTCTTGAGATCCTTTTTTTCTGCGCGTAATCTGCTGCTTGCAAACAAAAAAACCACCGCTACCAGCGGTGGTTTGTTTGCCGGATCAAGAGCTA 1710
GAAGAACTCTAGGAAAAAAAGACGCGCATTAGACGACGAACGTTTGTTTTTTTGGTGGCGATGGTCGCCACCAAACAAACGGCCTAGTTCTCGAT
ori

CCAACTCTTTTTCCGAAGGTAACTGGCTTCAGCAGAGCGCAGATACCAAATACTGTCCTTCTAGTGTAGCCGTAGTTAGGCCACCACTTCAAGAA 1805
GGTTGAGAAAAAGGCTTCCATTGACCGAAGTCGTCTCGCGTCTATGGTTTATGACAGGAAGATCACATCGGCATCAATCCGGTGGTGAAGTTCTT
ori

CTCTGTAGCACCGCCTACATACCTCGCTCTGCTAATCCTGTTACCAGTGGCTGCTGCCAGTGGCGATAAGTCGTGTCTTACCGGGTTGGACTCAA 1900
GAGACATCGTGGCGGATGTATGGAGCGAGACGATTAGGACAATGGTCACCGACGACGGTCACCGCTATTCAGCACAGAATGGCCCAACCTGAGTT
ori

GACGATAGTTACCGGATAAGGCGCAGCGGTCGGGCTGAACGGGGGGTTCGTGCACACAGCCCAGCTTGGAGCGAACGACCTACACCGAACTGAGA 1995
CTGCTATCAATGGCCTATTCCGCGTCGCCAGCCCGACTTGCCCCCCAAGCACGTGTGTCGGGTCGAACCTCGCTTGCTGGATGTGGCTTGACTCT
ori

TACCTACAGCGTGAGCATTGAGAAAGCGCCACGCTTCCCGAAGGGAGAAAGGCGGACAGGTATCCGGTAAGCGGCAGGGTCGGAACAGGAGAGCG 2090
ATGGATGTCGCACTCGTAACTCTTTCGCGGTGCGAAGGGCTTCCCTCTTTCCGCCTGTCCATAGGCCATTCGCCGTCCCAGCCTTGTCCTCTCGC
ori

CACGAGGGAGCTTCCAGGGGGAAACGCCTGGTATCTTTATAGTCCTGTCGGGTTTCGCCACCTCTGACTTGAGCGTCGATTTTTGTGATGCTCGT 2185
GTGCTCCCTCGAAGGTCCCCCTTTGCGGACCATAGAAATATCAGGACAGCCCAAAGCGGTGGAGACTGAACTCGCAGCTAAAAACACTACGAGCA
ori

```
CAGGGGGGCGGAGCCTATGGAAAAACGCCAGCAACGCGGCCTTTTTACGGTTCCTGGCCTTTTGCTGGCCTTTTGCTCACATGTTCTTTCCTGCG   2280
GTCCCCCCGCCTCGGATACCTTTTTGCGGTCGTTGCGCCGGAAAAATGCCAAGGACCGGAAAACGACCGGAAAACGAGTGTACAAGAAAGGACGC
ori

TTATCCCCTGATTCTGTGGATAACCGTATTACCGCCTTTGAGTGAGCTGATACCGCTCGCCGCAGCCGAACGACCGAGCGCAGCGAGTCAGTGAG   2375
AATAGGGGACTAAGACACCTATTGGCATAATGGCGGAAACTCACTCGACTATGGCGAGCGGCGTCGGCTTGCTGGCTCGCGTCGCTCAGTCACTC

CGAGGAAGCGGAAGAGCGCCTGATGCGGTATTTTCTCCTTACGCATCTGTGCGGTATTTCACACCGCAGACCAGCCGCGTAACCTGGCAAAATCG   2470
GCTCCTTCGCCTTCTCGCGGACTACGCCATAAAAGAGGAATGCGTAGACACGCCATAAAGTGTGGCGTCTGGTCGGCGCATTGGACCGTTTTAGC

GTTACGGTTGAGTAATAAATGGATGCCCTGCGTAAGCGGGTGTGGGCGGACAATAAAGTCTTAAACTGAACAAAATAGATCTAAACTATGACAAT   2565
CAATGCCAACTCATTATTTACCTACGGGACGCATTCGCCCACACCCGCCTGTTATTTCAGAATTTGACTTGTTTTATCTAGATTTGATACTGTTA
Tn7R

AAAGTCTTAAACTAGACAGAATAGTTGTAAACTGAAATCAGTCCAGTTATGCTGTGAAAAAGCATACTGGACTTTTGTTATGGCTAAAGCAAACT   2660
TTTCAGAATTTGATCTGTCTTATCAACATTTGACTTTAGTCAGGTCAATACGACACTTTTTCGTATGACCTGAAAACAATACCGATTTCGTTTGA
Tn7R

CTTCATTTTCTGAAGTGCAAATTGCCCGTCGTATTAAAGAGGGGCGTGGCCAAGGGCATGGTAAAGACTATATTCGCGGCGTTGTGACAATTTAC   2755
GAAGTAAAAGACTTCACGTTTAACGGGCAGCATAATTTCTCCCCGCACCGGTTCCCGTACCATTTCTGATATAAGCGCCGCAACACTGTTAAATG
Tn7R

EcoRV
CGAACAACTCCGCGGCCGGGAAGCCGATCTCGGCTTGAACGAATTGTTAGGTGGCGGTACTTGGGTCGATATCAAAGTGCATCACTTCTTCCCGT   2850
GCTTGTTGAGGCGCCGGCCCTTCGGCTAGAGCCGAACTTGCTTAACAATCCACCGCCATGAACCCAGCTATAGTTTCACGTAGTGAAGAAGGGCA
175 170 165
* T A T S P D I D F H M V E E R
GmR

ATGCCCAACTTTGTATAGAGAGCCACTGCGGGATCGTCACCGTAATCTGCTTGCACGTAGATCACATAAGCACCAAGCGCGTTGGCCTCATGCTT   2945
TACGGGTTGAAACATATCTCTCGGTGACGCCCTAGCAGTGGCATTAGACGAACGTGCATCTAGTGTATTCGTGGTTCGCGCAACCGGAGTACGAA
160 155 150 145 140 135
I G L K T Y L A V A P D D G Y D A Q V Y I V Y A G L A N A E H K
GmR

GAGGAGATTGATGAGCGCGGTGGCAATGCCCTGCCTCCGGTGCTCGCCGGAGACTGCGAGATCATAGATATAGATCTCACTACGCGGCTGCTCAA   3040
CTCCTCTAACTACTCGCGCCACCGTTACGGGACGGAGGCCACGAGCGGCCTCTGACGCTCTAGTATCTATATCTAGAGTGATGCGCCGACGAGTT
130 125 120 115 110 105 100
L L N I L A T A I G Q R R H E G S V A L D Y I Y I E S R P Q E F
GmR

ACCTGGGCAGAACGTAAGCCGCGAGAGCGCCAACAACCGCTTCTTGGTCGAAGGCAGCAAGCGCGATGAATGTCTTACTACGGAGCAAGTTCCCG   3135
TGGACCCGTCTTGCATTCGGCGCTCTCGCGGTTGTTGGCGAAGAACCAGCTTCCGTCGTTCGCGCTACTTACAGAATGATGCCTCGTTCAAGGGC
95 90 85 80 75 70
R P L V Y A A L A G V V A E Q D F A A L A I F T K S R L L N G
GmR

AGGTAATCGGAGTCCGGCTGATGTTGGGAGTAGGTGGCTACGTCTCCGAACTCACGACCGAAAAGATCAAGAGCAGCCCGCATGGATTTGACTTG   3230
TCCATTAGCCTCAGGCCGACTACAACCCTCATCCACCGATGCAGAGGCTTGAGTGCTGGCTTTTCTAGTTCTCGTCGGGCGTACCTAAACTGAAC
65 60 55 50 45 40
L Y D S D P Q H Q S Y T A V D G F E R G F L D L A A R M S K V Q
GmR

GTCAGGGCCGAGCCTACATGTGCGAATGATGCCCATACTTGAGCCACCTAACTTTGTTTTAGGGCGACTGCCCTGCTGCGTAACATCGTTGCTGC   3325
CAGTCCCGGCTCGGATGTACACGCTTACTACGGGTATGAACTCGGTGGATTGAAACAAAATCCCGCTGACGGGACGACGCATTGTAGCAACGACG
35 30 25 20 15 10 5
D P G L R C T R I I G M S S G G L K T K P R S G Q Q T V D N S S
GmR
```

TGCGTAACATCGTTGCTGCTCCATAACATCAAACATCGACCCACGGCGTAACGCGCTTGCTGCTTGGATGCCCGAGGCATAGACTGTACAAAAAA 3420
ACGCATTGTAGCAACGACGAGGTATTGTAGTTTGTAGCTGGGTGCCGCATTGCGCGAACGACGAACCTACGGGCTCCGTATCTGACATGTTTTTT
1
R L M
GmR

ACAGTCATAACAAGCCATGAAAACCGCCACTGCGCCGTTACCACCGCTGCGTTCGGTCAAGGTTCTGGACCAGTTGCGTGAGCGCATACGCTACT 3515
TGTCAGTATTGTTCGGTACTTTTGGCGGTGACGCGGCAATGGTGGCGACGCAAGCCAGTTCCAAGACCTGGTCAACGCACTCGCGTATGCGATGA

TGCATTACAGTTTACGAACCGAACAGGCTTATGTCAACTGGGTTCGTGCCTTCATCCGTTTCCACGGTGTGCGTCACCCGGCAACCTTGGGCAGC 3610
ACGTAATGTCAAATGCTTGGCTTGTCCGAATACAGTTGACCCAAGCACGGAAGTAGGCAAAGGTGCCACACGCAGTGGGCCGTTGGAACCCGTCG
-10 -35
Pc promoter

AGCGAAGTCGAGGCATTTCTGTCCTGGCTGGCGAACGAGCGCAAGGTTTCGGTCTCCACGCATCGTCAGGCATTGGCGGCCTTGCTGTTCTTCTA 3705
TCGCTTCAGCTCCGTAAAGACAGGACCGACCGCTTGCTCGCGTTCCAAAGCCAGAGGTGCGTAGCAGTCCGTAACCGCCGGAACGACAAGAAGAT

CGGCAAGGTGCTGTGCACGGATCTGCCCTGGCTTCAGGAGATCGGAAGACCTCGGCCGTCGCGGCGCTTGCCGGTGGTGCTGACCCCGGATGAAG 3800
GCCGTTCCACGACACGTGCCTAGACGGGACCGAAGTCCTCTAGCCTTCTGGAGCCGGCAGCGCCGCGAACGGCCACCACGACTGGGGCCTACTTC

TGGTTCGCATCCTCGGTTTTCTGGAAGGCGAGCATCGTTTGTTCGCCCAGGACTCTAGCTATAGTTCTAGTGGTTGGCTACGTATACTCCGGAAT 3895
ACCAAGCGTAGGAGCCAAAAGACCTTCCGCTCGTAGCAAACAAGCGGGTCCTGAGATCGATATCAAGATCACCAACCGATGCATATGAGGCCTTA

Sequencing primer：poly-F
AAATGATAACCATCTCGC
ATTAATAGATCATGGAGATAATTAAAATGATAACCATCTCGCAAATAAATAAGTATTTTACTGTTTTCGTAACAGTTTTGTAATAAAAAAACCTA 3990
TAATTATCTAGTACCTCTATTAATTTTACTATTGGTAGAGCGTTTATTTATTCATAAAATGACAAAAGCATTGTCAAAACATTATTTTTTTGGAT
polyhedrin promoter

**BamHI**
Bac-BamH I-F
CCCACCATCGGGCGCGGATCC
TAAATATTCCGGATTATTCATACCGTCCCACCATCGGGCGCGGATCCACCATGACAACCACTGCATGCTTGGTTTGTCTAGTAAAGGTGGTGAGG 4085
ATTTATAAGGCCTAATAAGTATGGCAGGGTGGTAGCCCGCGCCTAGGTGGTACTGTTGGTGACGTACGAACCAAACAGATCATTTCCACCACTCC
5 10 15
T M T T T A C L V C L V K V V R
polyhedrin promoter
15 bp+BamH I
BVDV-XH32-E2
CCTAATAAGTATGGCAGGGTGGTAGCCCGCGCCTAGG
pFast Bac-BamHI-LR

GGCCAAGTGTTGCAAGGTATACTGTGGCTGATGCTCATAACAGGGACGCAAGGGTACCCAGAATGCAAACCCGACTTTTCATACGCCATAGCCAA 4180
CCGGTTCACAACGTTCCATATGACACCGACTACGAGTATTGTCCCTGCGTTCCCATGGGTCTTACGTTTGGGCTGAAAAGTATGCGGTATCGGTT
20 25 30 35 40 45 50
G Q V L Q G I L W L M L I T G T Q G Y P E C K P D F S Y A I A K
BVDV-XH32-E2

AAATGATGAGATTGGCCTACTTGGAGCTACAGGCCTTGTCACTCGGTGGTACAAATACTCGGATGGGGTTCGGCTGAAGGACTCAACTGTTGAGG 4275
TTTACTACTCTAACCGGATGAACCTCGATGTCCGGAACAGTGAGCCACCATGTTTATGAGCCTACCCCAAGCCGACTTCCTGAGTTGACAACTCC
55 60 65 70 75 80
N D E I G L L G A T G L V T R W Y K Y S D G V R L K D S T V E
BVDV-XH32-E2

EcoRV
TTTGGTGTGAGGGTGGAGAGTTCAGATATCTAATTAGATGTGAGAGGGAAGCTAGGTATCTGGCTGCTCTACACAACAGAGCTTTGCCGACATCT 4370
AAACCACACTCCCACCTCTCAAGTCTATAGATTAATCTACACTCTCCCTTCGATCCATAGACCGACGAGATGTGTTGTCTCGAAACGGCTGTAGA
85 90 95 100 105 110
V W C E G G E F R Y L I R C E R E A R Y L A A L H N R A L P T S
BVDV-XH32-E2

EcoRI

GTAGAATTTAAAAAAATTGTAGATGAAAAAAAACAAGAGGACATAGTAGAAATGGATGACAATTTTGAATTCGGTCTTTGCCCGTGTGATGCTAA 4465

CATCTTAAATTTTTTTAACATCTACTTTTTTTTGTTCTCCTGTATCATCTTTACCTACTGTTAAAACTTAAGCCAGAAACGGGCACACTACGATT

115 120 125 130 135 140 145

V E F K K I V D E K K Q E D I V E M D D N F E F G L C P C D A K

BVDV-XH32-E2

ACCCTTGGTAAGGGGAAAATTTAATACAACACTTCTAAATGGACCAGCCTTCCAGATGGTTTGCCCTATAGGATGGACAGGGACTGTAAGCTGTA 4560

TGGGAACCATTCCCCTTTTAAATTATGTTGTGAAGATTTACCTGGTCGGAAGGTCTACCAAACGGGATATCCTACCTGTCCCTGACATTCGACAT

150 155 160 165 170 175

P L V R G K F N T T L L N G P A F Q M V C P I G W T G T V S C

BVDV-XH32-E2

CACTGGCCAATGAAGATACGTTAGCCACAACCGTTGTGCGAACATATAAGAGGGTCAGGCCTTTTCCATATAGGCAGGACTGTGTCACACAGAAA 4655

GTGACCGGTTACTTCTATGCAATCGGTGTTGGCAACACGCTTGTATATTCTCCCAGTCCGGAAAAGGTATATCCGTCCTGACACAGTGTGTCTTT

180 185 190 195 200 205

T L A N E D T L A T T V V R T Y K R V R P F P Y R Q D C V T Q K

BVDV-XH32-E2

ACCATTGGGGAAGACCTCTATGACTGTGCCCTAGGAGGGAATTGGACCTGTGTGTTGGGGGATATACTACGATATGTAGCTGGGCCTGTCGAGTC 4750

TGGTAACCCCTTCTGGAGATACTGACACGGGATCCTCCCTTAACCTGGACACACAACCCCCTATATGATGCTATACATCGACCCGGACAGCTCAG

210 215 220 225 230 235 240

T I G E D L Y D C A L G G N W T C V L G D I L R Y V A G P V E S

BVDV-XH32-E2

TTGTAAGTGGTGTGGTCACAAATTTCTTAAAAGTGAGGGTCTGCCGCATTTCCCAATTGGCAAATGCAGGCTGAAGAATGAGAGTGGCTATAGAC 4845

AACATTCACCACACCAGTGTTTAAAGAATTTTCACTCCCAGACGGCGTAAAGGGTTAACCGTTTACGTCCGACTTCTTACTCTCACCGATATCTG

245 250 255 260 265 270

C K W C G H K F L K S E G L P H F P I G K C R L K N E S G Y R

BVDV-XH32-E2

TAGTGGATGGGACTTCTTGCAACAGAGACGGCGTGGCTATAGTGCCATCTGGCCGGGTCAAATGCAAAATAGGAGACACAGTGGTGCAGGTCATT 4940

ATCACCTACCCTGAAGAACGTTGTCTCTGCCGCACCGATATCACGGTAGACCGGCCCAGTTTACGTTTTATCCTCTGTGTCACCACGTCCAGTAA

275 280 285 290 295 300

L V D G T S C N R D G V A I V P S G R V K C K I G D T V V Q V I

BVDV-XH32-E2

**NdeI**

GCAACGGATGATAAACTAGGGCCCATGCCTTGCAAACCATATGAAATCATTTCCAGTGAGGGGCCGGTAAAAAAGACGGCATGCACCTTCAACTA 5035

CGTTGCCTACTATTTGATCCCGGGTACGGAACGTTTGGTATACTTTAGTAAAGGTCACTCCCCGGCCATTTTTTCTGCCGTACGTGGAAGTTGAT

305 310 315 320 325 330 335

A T D D K L G P M P C K P Y E I I S S E G P V K K T A C T F N Y

BVDV-XH32-E2

CACAAGAACATTAAAAAACAAGTACTTTGAGCCTAGGGACAACTATTTCCAACAATACATGTTAAAGGGGGAGTACCAATACTGGTTTGACCTAG 5130

GTGTTCTTGTAATTTTTTGTTCATGAAACTCGGATCCCTGTTGATAAAGGTTGTTATGTACAATTTCCCCCTCATGGTTATGACCAAACTGGATC

340 345 350 355 360 365

T R T L K N K Y F E P R D N Y F Q Q Y M L K G E Y Q Y W F D L

BVDV-XH32-E2

EcoRI

pFast BacI-EcoRI-LF

GAATTCAAAGGCCTACGTCGACGAGCTCACTAGTC

**NotI** **XhoI**

AGATCCATCATCACCATCACCATTAAGAATTCAAAGGCCTACGTCGACGAGCTCACTAGTCGCGGCCGCTTTCGAATCTAGAGCCTGCAGTCTCG 5225

TCTAGGTAGTAGTGGTAGTGGTAATTCTTAAGTTTCCGGATGCAGCTGCTCGAGTGATCAGCGCCGGCGAAAGCTTAGATCTCGGACGTCAGAGC

1 5

E I H H H H H H *

BVDV-XH32-E2 6xHis EcoR I+15 bp

CTTAAGTTTCCGGATGCAGCT

Bac-EcoR I-R

**HindIII**

AGGCATGCGGTACCAAGCTTGTCGAGAAGTACTAGAGGATCATAATCAGCCATACCACATTTGTAGAGGTTTTACTTGCTTTAAAAAACCTCCCA 5320

TCCGTACGCCATGGTTCGAACAGCTCTTCATGATCTCCTAGTATTAGTCGGTATGGTGTAAACATCTCCAAAATGAACGAAATTTTTTGGAGGGT

```
CACCTCCCCCTGAACCTGAAACATAAAATGAATGCAATTGTTGTTGTTAACTTGTTTATTGCAGCTTATAATGGTTACAAATAAAGCAATAGCAT
                                                                                                   5415
GTGGAGGGGGACTTGGACTTTGTATTTTACTTACGTTAACAACAACAATTGAACAAATAACGTCGAATATTACCAATGTTTATTTCGTTATCGTA
```

SV40 poly(A) signal

CGTTATCGTA
Sequencing primer: SV40pA-R

```
CACAAATTTCACAAATAAAGCATTTTTTTCACTGCATTCTAGTTGTGGTTTGTCCAAACTCATCAATGTATCTTATCATGTCTGGATCTGATCAC
                                                                                                   5510
GTGTTTAAAGTGTTTATTTCGTAAAAAAAGTGACGTAAGATCAACACCAAACAGGTTTGAGTAGTTACATAGAATAGTACAGACCTAGACTAGTG
```

SV40 poly(A) signal

GTGTTTAAAG
Sequencing primer: SV40pA-R

```
TGCTTGAGCCTAGGAGATCCGAACCAGATAAGTGAAATCTAGTTCCAAACTATTTTGTCATTTTTAATTTTCGTATTAGCTTACGACGCTACACC
                                                                                                   5605
ACGAACTCGGATCCTCTAGGCTTGGTCTATTCACTTTAGATCAAGGTTTGATAAAACAGTAAAAATTAAAAGCATAATCGAATGCTGCGATGTGG
```

Tn7L

```
CAGTTCCCATCTATTTTGTCACTCTTCCCTAAATAATCCTTAAAAACTCCATTTCCACCCCTCCCAGTTCCCAACTATTTTGTCCGCCCACAGCG
                                                                                                   5700
GTCAAGGGTAGATAAAACAGTGAGAAGGGATTTATTAGGAATTTTTGAGGTAAAGGTGGGGAGGGTCAAGGGTTGATAAAACAGGCGGGTGTCGC
```

Tn7L

```
GGGCATTTTTCTTCCTGTTATGTTTTTAATCAAACATCCTGCCAACTCCATGTGACAAACCGTCATCTTCGGCTACTTTTTCTCTGTCACAGAAT
                                                                                                   5795
CCCGTAAAAAGAAGGACAATACAAAAATTAGTTTGTAGGACGGTTGAGGTACACTGTTTGGCAGTAGAAGCCGATGAAAAAGAGACAGTGTCTTA
```

```
GAAAATTTTTCTGTCATCTCTTCGTTATTAATGTTTGTAATTGACTGAATATCAACGCTTATTTGCAGCCTGAATGGCGAATGG     3'
                                                                                    ...  5879
CTTTTAAAAAGACAGTAGAGAAGCAATAATTACAAACATTAACTGACTTATAGTTGCGAATAAACGTCGGACTTACCGCTTACC     5'
```

# 4. Sanger sequencing chromatograms of pFast Bac1-BVDV-XH32-E2 vector for inserts.

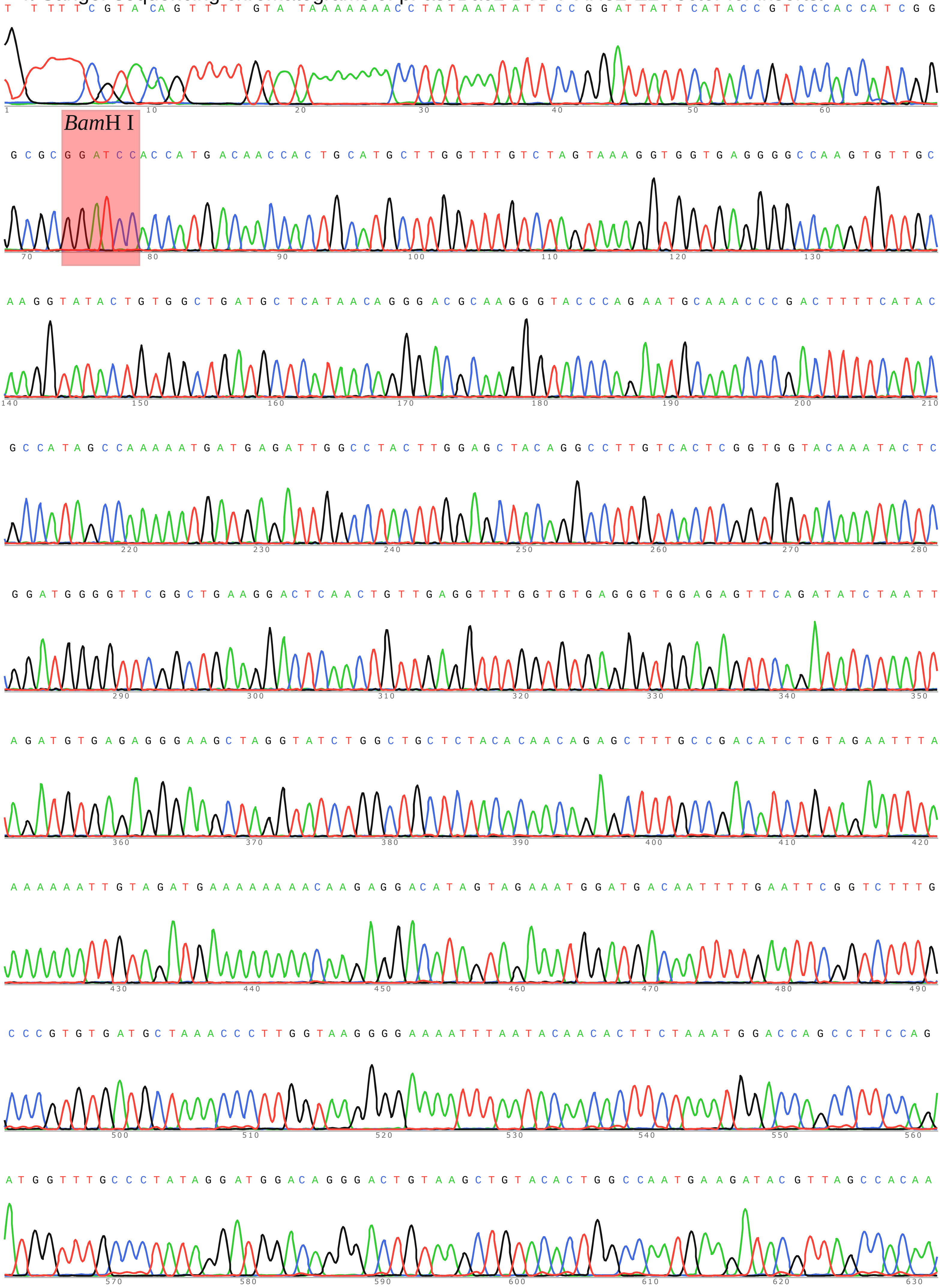

CCGTTGTGCGAACATATAAGAGGGTCAGGCCTTTTCCATATAGGCAGGACTGTGTCACACAGAAAACCATT

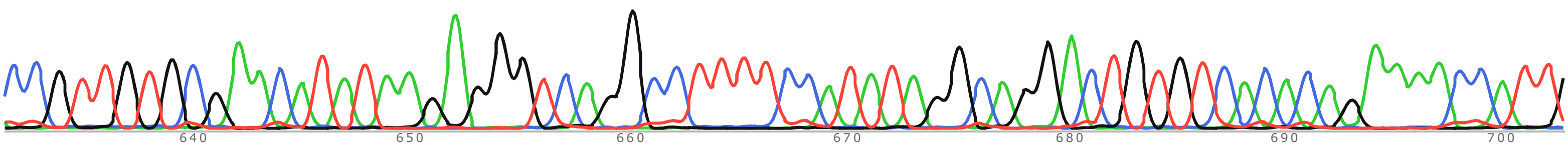


GGGGAAGACCTCTATGACTGTGCCCTAGGAGGGAATTGGACCTGTGTGTTGGGGATATACTACGATATGT

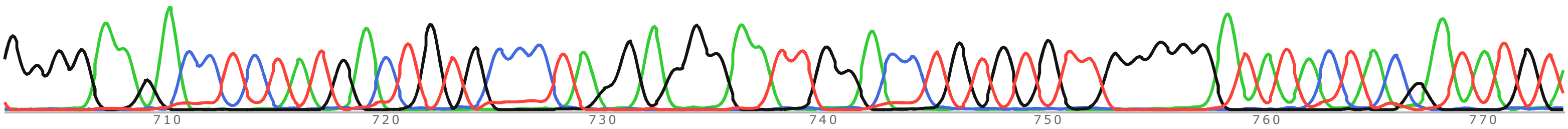


AGCTGGGCCTGTCGAGTCTTGTAAGTGGTGTGGTCACAAATTTCTTAAAAGTGAGGGTCTGCCGCATTTCCC

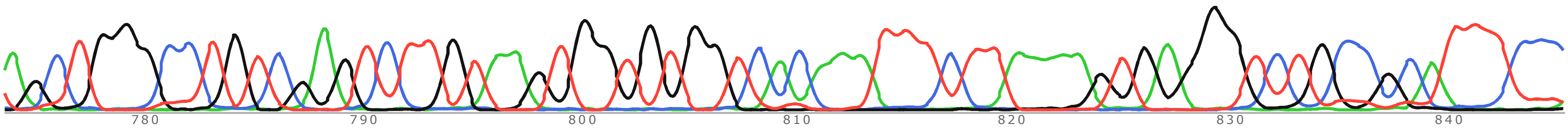


AATTGGCAAATGCACGCTGAAGAATGAGAGTGGCTATAGACTAGTGGATGGGACTTCTTGCAACAGAGAC

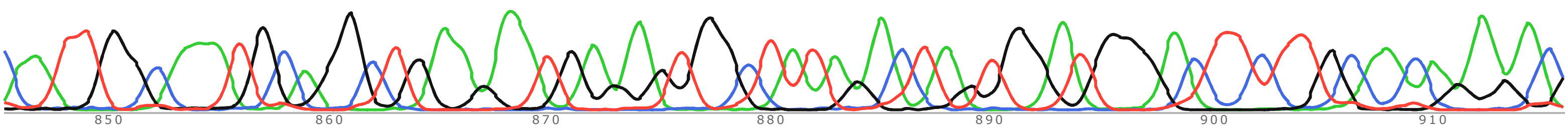


GGCGTGGCTATAGTGCCATCTGGCCGGGTCAAATGCAAAATAGGAGACACAGTGGTGCAGGTCATTGCA

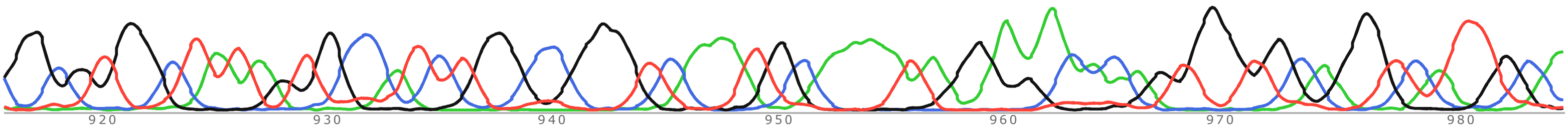


ACGGATGATAAACTAG

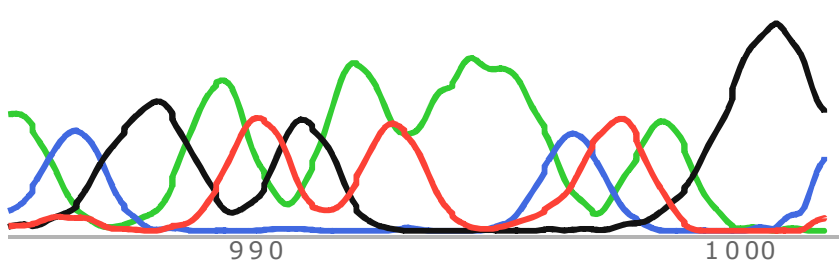

AAA TTGTAGATG AAAAAAA C AA GAGG A CATAGTAG AAAT GG ATGA C AA TTTT G AA TT C GG T C TTT G

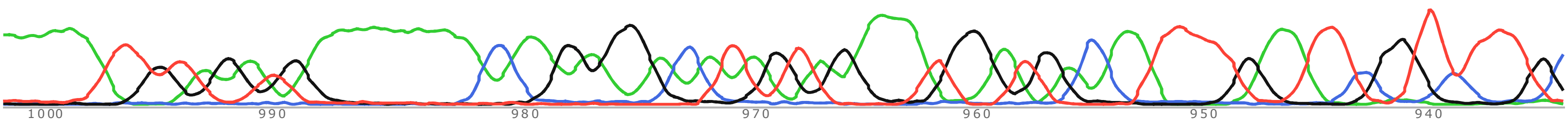


CCC GTGTGATGCTAAA CCC TT GG T AA GGGG AAAATTTAATAC AACAC TT C TAAAT GG ACCA G CC TTT C CA G

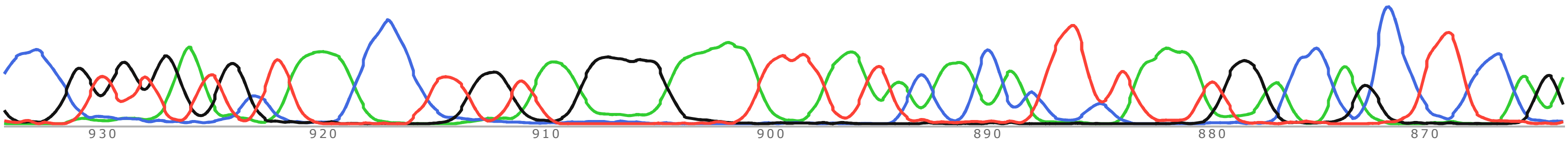


ATGGTTTGCCCTATAGGATGGACAGGGACTGTAAGCTGTACACTGGCCAATGAAGATACGTTAGCCACAAC

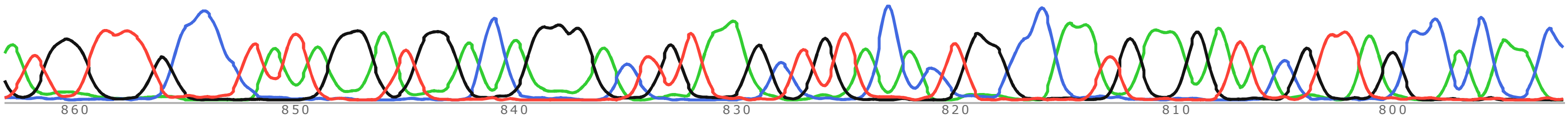


CGTTGTGCGAACATATAAGAGGGTCAGGCCTTTTCCATATAGGCAGGACTGTGTCACACAGAAAACCATTG

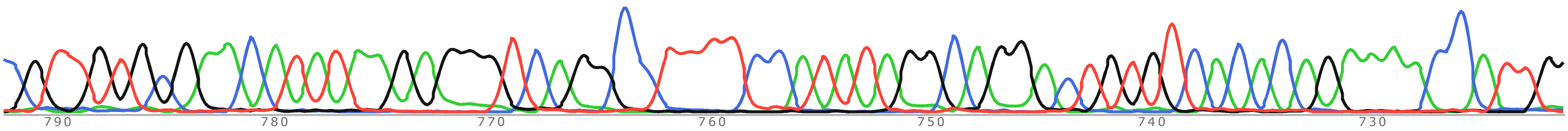


GG GAAGACCTCTATGACTGTGCCCTAGGAGGGAATTGGACCTGTGTGTTGGGGATATACTACGATATGTA

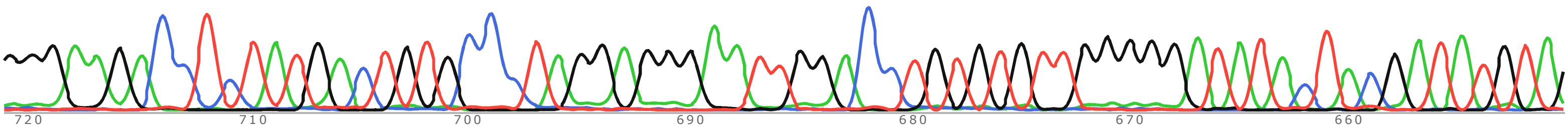


GCTGGGCCTGTCGAGTCTTGTAAGTGGTGTGGTCACAAATTTCTTAAAAGTGAGGGTCTGCCGCATTTCCC

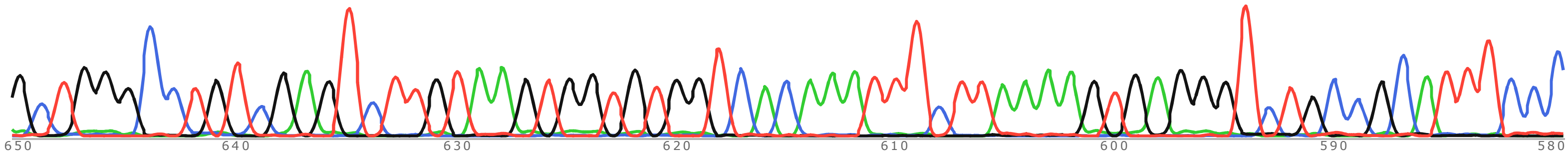


AATTGGCAAATGCAGGCTGAAGAATGAGAGTGGCTATAGACTAGTGGATGGGACTTCTTGCAACAGAGAC

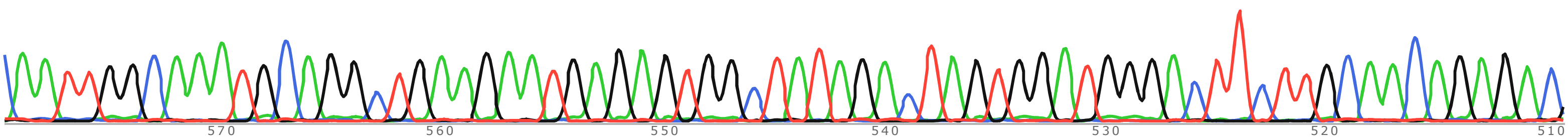


GGCGTGGCTATAGTGCCATCTGGCCGGGTCAAATGCAAAATAGGAGACACAGTGGTGCAGGTCATTGCAA

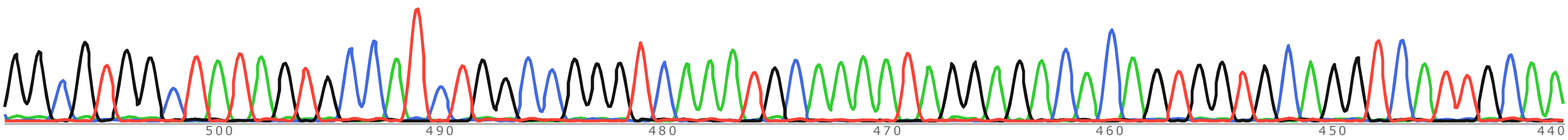


CGGATGATAAACTAGGGCCCATGCCTTGCAAACCATATGAAATCATTTCCAGTGAGGGGCCGGTAAAAAA

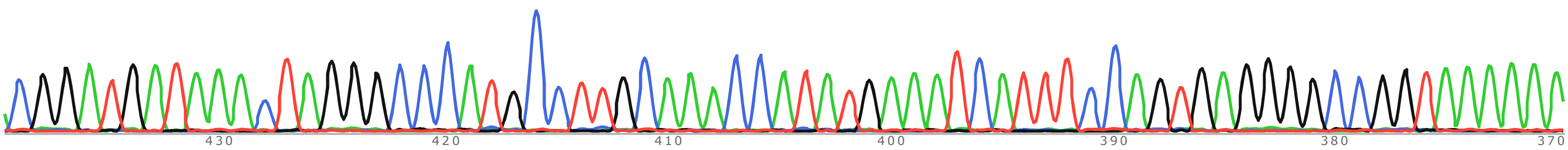

G A C G G C A T G C A C C T T C A A C T A C A C A A G A A C A T T A A A A A A C A A G T A C T T T G A G C C T A G G G A C A A C T A T T T C

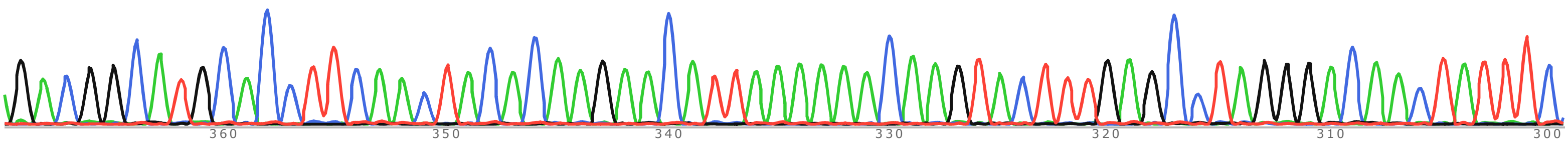

C A A C A A T A C A T G T T A A A G G G G G A G T A C C A A T A C T G G T T T G A C C T A G A G A T C C A T C A T C A C C A T C A C C A T T A

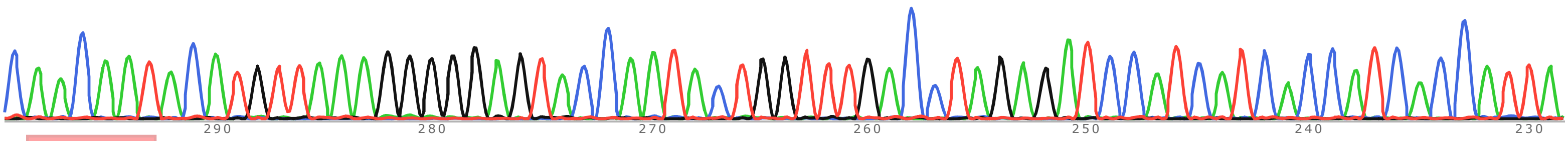

*Eco*R I

A G A A T T C A A A G G C C T A C G T C G A C G A G C T C A C T A G T C G C G G C C G C T T T C G A A T C T A G A G C C T G C A G T C T C G A

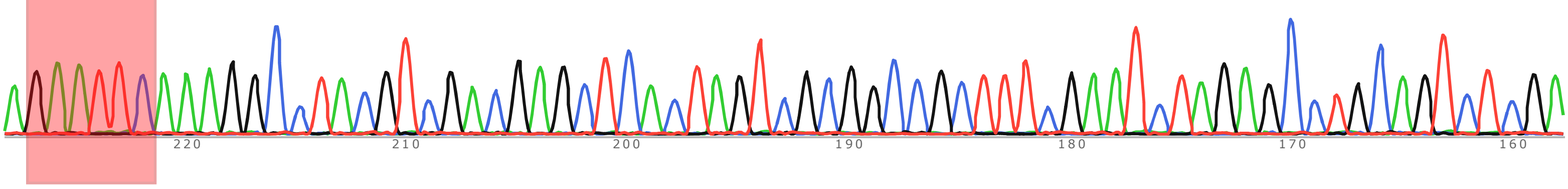

G G C A T G C G G T A C C A A G C T T G T C G A G A A G T A C T A G A G G A T C A T A A T C A G C C A T A C C A C A T T T G T A G A G G T T T

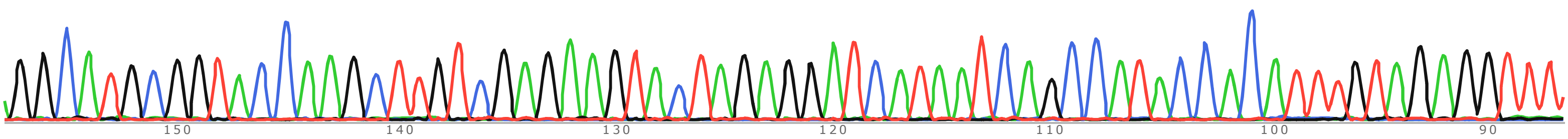

T A C T T G C T T T A A A A A A C C T C C C A C A C C T C C C C C T G A A C C T G A A A C A T A A A A T G A A T G C A A T T G T T G T T G T T

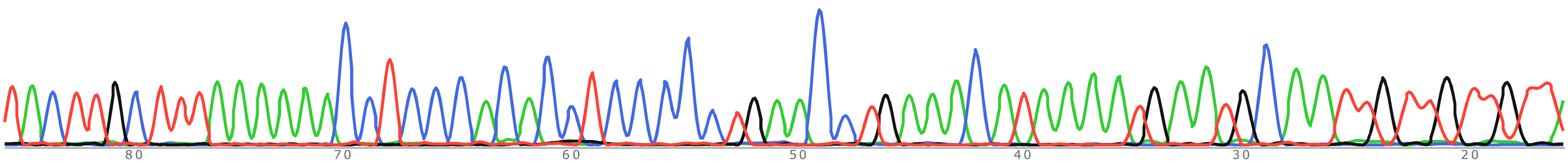

A A C T T G T T A T G C A G T

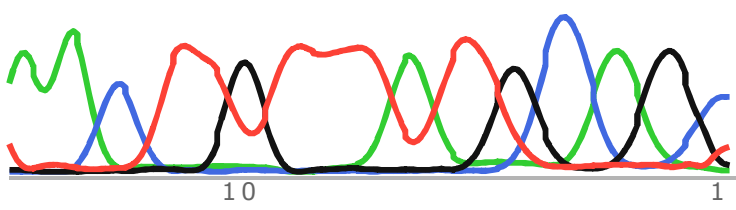

Supplement: Supplementary file 1 [file 13567_2026_1852_MOESM1_ESM.pdf]
