# Supplementary material for: Generation and epitope mapping of two distinct neutralizing mAb subsets targeting BVDV E2: a pan-pestivirus cross-reactive linear epitope and a BVDV-specific conformational epitope
Source: Vet Res. 2026 Oct 7;57:176. doi: 10.1186/s13567-026-01852-5 (PMC13644284; doi:10.1186/s13567-026-01852-5)
Supplement: Supplementary file 2 [file 13567_2026_1852_MOESM3_ESM.pdf]

pcDNA3.1-BVDV-XH32-E2

The BVDV-XH32-E2 gene (1137 bp) was codon-optimized and synthesized based on mammalian cell expression system, followed by cloning of the E2 gene with 6×His Tag into the pcDNA3.1(+) vector (*Bam*HI/*Eco*R I site) by Sangon Biotech(China). And the insert was verified using sequencing primers:

pRLuc-MCS-F-GGGCGGTAGGCGTGTACGGTGG,

BGH-r-TAGAAGGCACAGTCGAGG.

There are the plasmid map, complete sequence and Sanger sequencing chromatograms of the pcDNA3.1-BVDV-XH32-E2.

1. Plasmid map of pcDNA3.1-BVDV-XH32-E2 vector 1

2. Complete sequence of pcDNA3.1-BVDV-XH32-E2 vector 2

3. Sanger sequencing chromatograms of pcDNA3.1-BVDV-XH32-E2 vector for inserts:

(1) pcDNA3.1-BVDV1-32-E2_pRLuc-MCS-F 9

(2) pcDNA3.1-BVDV1-32-E2_BGH-r 11

1. Plasmid map of pcDNA3.1-BVDV-XH32-E2 vector

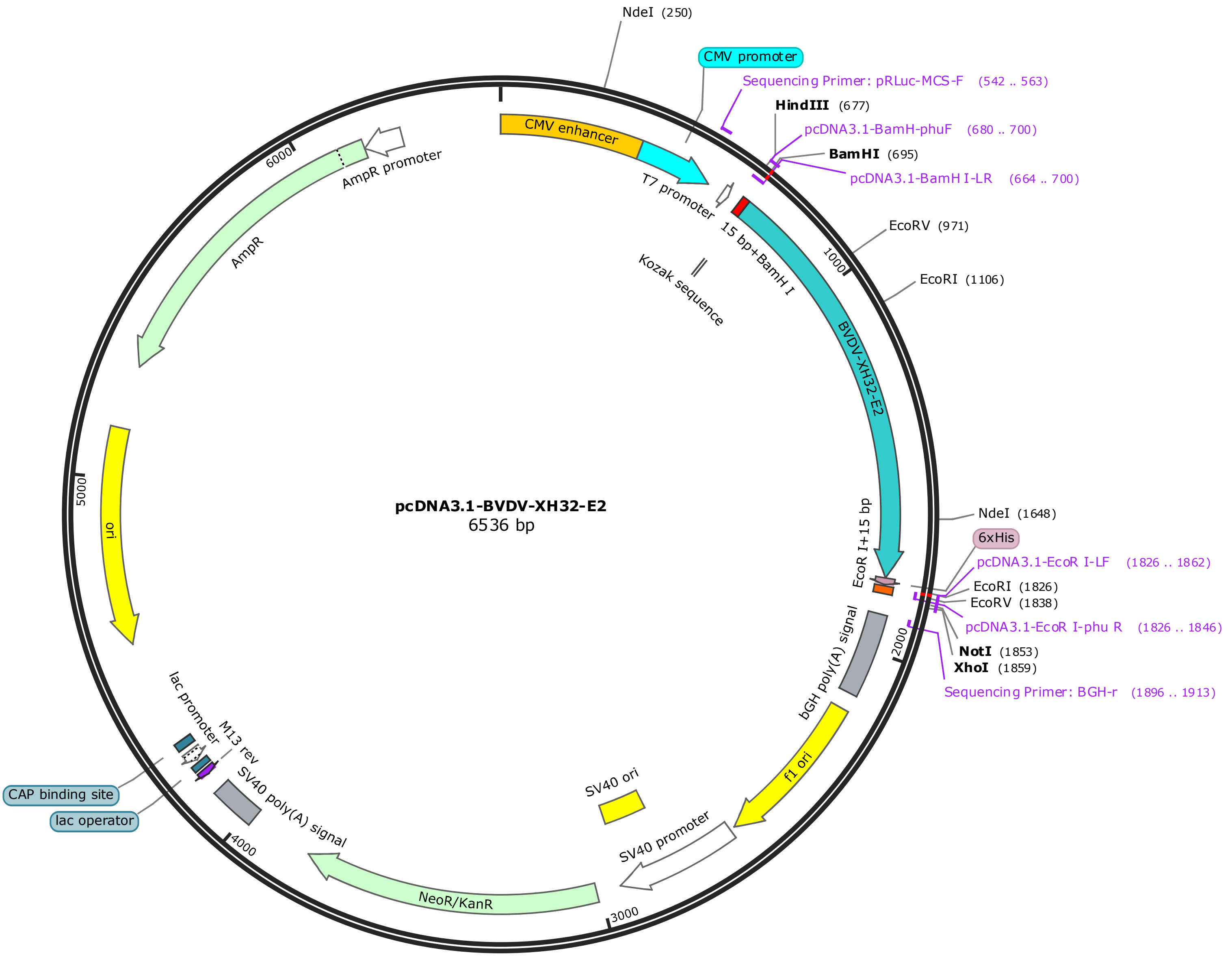

2. Complete sequence of pcDNA3.1-BVDV-XH32-E2 vector

```
5' GACATTGATTATTGACTAGTTATTAATAGTAATCAATTACGGGGTCATTAGTTCATAGCCCATATATGGAGTTCCGCGTTACATAACTTACGGTA
                                                                                                      95
3' CTGTAACTAATAACTGATCAATAATTATCATTAGTTAATGCCCCAGTAATCAAGTATCGGGTATATACCTCAAGGCGCAATGTATTGAATGCCAT
   CMV enhancer

   AATGGCCCGCCTGGCTGACCGCCCAACGACCCCCGCCCATTGACGTCAATAATGACGTATGTTCCCATAGTAACGCCAATAGGGACTTTCCATTG
                                                                                                      190
   TTACCGGGCGGACCGACTGGCGGGTTGCTGGGGGCGGGTAACTGCAGTTATTACTGCATACAAGGGTATCATTGCGGTTATCCCTGAAAGGTAAC
   CMV enhancer

   NdeI
   ACGTCAATGGGTGGAGTATTTACGGTAAACTGCCCACTTGGCAGTACATCAAGTGTATCATATGCCAAGTACGCCCCCTATTGACGTCAATGACG
                                                                                                      285
   TGCAGTTACCCACCTCATAAATGCCATTTGACGGGTGAACCGTCATGTAGTTCACATAGTATACGGTTCATGCGGGGGATAACTGCAGTTACTGC
   CMV enhancer

   GTAAATGGCCCGCCTGGCATTATGCCCAGTACATGACCTTATGGGACTTTCCTACTTGGCAGTACATCTACGTATTAGTCATCGCTATTACCATG
                                                                                                      380
   CATTTACCGGGCGGACCGTAATACGGGTCATGTACTGGAATACCCTGAAAGGATGAACCGTCATGTAGATGCATAATCAGTAGCGATAATGGTAC
   CMV enhancer

   GTGATGCGGTTTTGGCAGTACATCAATGGGCGTGGATAGCGGTTTGACTCACGGGGATTTCCAAGTCTCCACCCCATTGACGTCAATGGGAGTTT
                                                                                                      475
   CACTACGCCAAAACCGTCATGTAGTTACCCGCACCTATCGCCAAACTGAGTGCCCCTAAAGGTTCAGAGGTGGGGTAACTGCAGTTACCCTCAAA
   CMV promoter

   Sequencing Primer: pRLuc-MCS-F
   GGGCGGTAGGCGTGTACGGTGG
   GTTTTGGCACCAAAATCAACGGGACTTTCCAAAATGTCGTAACAACTCCGCCCCATTGACGCAAATGGGCGGTAGGCGTGTACGGTGGGAGGTCT
                                                                                                      570
   CAAAACCGTGGTTTTAGTTGCCCTGAAAGGTTTTACAGCATTGTTGAGGCGGGGTAACTGCGTTTACCCGCCATCCGCACATGCCACCCTCCAGA
   CMV promoter

   ATATAAGCAGAGCTCTCTGGCTAACTAGAGAACCCACTGCTTACTGGCTTATCGAAATTAATACGACTCACTATAGGGAGACCCAAGCTGGCTAG
                                                                                                      665
   TATATTCGTCTCGAGAGACCGATTGATCTCTTGGGTGACGAATGACCGAATAGCTTTAATTATGCTGAGTGATATCCCTCTGGGTTCGACCGATC
   CMV promoter                                                        T7 promoter
   TC
   pcDNA3.1-BamH I-LR

   HindIII BamHI
   pcDNA3.1-BamH-phuF
   CTTGGTACCGAGCTCGGATCC
   CGTTTAAACTTAAGCTTGGTACCGAGCTCGGATCCGCCACCATGGCAACAACCACTGCATGCTTGGTTTGTCTAGTAAAGGTGGTGAGGGGCCAA
                                                                                                      760
   GCAAATTTGAATTCGAACCATGGCTCGAGCCTAGGCGGTGGTACCGTTGTTGGTGACGTACGAACCAAACAGATCATTTCCACCACTCCCCGGTT
   1 A T M A 5 T T T A C 10 L V C L V 15 K V V R G 20 Q
   15 bp+BamH I    BVDV-XH32-E2
   Kozak sequence
   GCAAATTTGAATTCGAACCATGGCTCGAGCCTAGG
   pcDNA3.1-BamH I-LR

   GTGTTGCAAGGTATACTGTGGCTGATGCTCATAACAGGGACGCAAGGGTACCCAGAATGCAAACCCGACTTTTCATACGCCATAGCCAAAAATGA
                                                                                                      855
   CACAACGTTCCATATGACACCGACTACGAGTATTGTCCCTGCGTTCCCATGGGTCTTACGTTTGGGCTGAAAAGTATGCGGTATCGGTTTTTACT
   V L Q G 25 I L W L M 30 L I T G T 35 Q G Y P E 40 C K P D F 45 S Y A I A 50 K N D
   BVDV-XH32-E2

   TGAGATTGGCCTACTTGGAGCTACAGGCCTTGTCACTCGGTGGTACAAATACTCGGATGGGGTTCGGCTGAAGGACTCAACTGTTGAGGTTTGGT
                                                                                                      950
   ACTCTAACCGGATGAACCTCGATGTCCGGAACAGTGAGCCACCATGTTTATGAGCCTACCCCAAGCCGACTTCCTGAGTTGACAACTCCAAACCA
   E I 55 G L L G A 60 T G L V T 65 R W Y K Y 70 S D G V R 75 L K D S T 80 V E V W
   BVDV-XH32-E2
```

```
                                 EcoRV
GTGAGGGTGGAGAGTTCAGATATCTAATTAGATGTGAGAGGGAAGCTAGGTATCTGGCTGCTCTACACAACAGAGCTTTGCCGACATCTGTAGAA   1045
CACTCCCACCTCTCAAGTCTATAGATTAATCTACACTCTCCCTTCGATCCATAGACCGACGAGATGTGTTGTCTCGAAACGGCTGTAGACATCTT
   85         90          95          100         105         110         115
C E G G E F R Y L I R C E R E A R Y L A A L H N R A L P T S V E
BVDV-XH32-E2

                                                                   EcoRI
TTTAAAAAAATTGTAGATGAAAAAAAACAAGAGGACATAGTAGAAATGGATGACAATTTTGAATTCGGTCTTTGCCCGTGTGATGCTAAACCCTT   1140
AAATTTTTTTAACATCTACTTTTTTTTGTTCTCCTGTATCATCTTTACCTACTGTTAAAACTTAAGCCAGAAACGGGCACACTACGATTTGGGAA
         120         125         130         135         140         145
F K K I V D E K K Q E D I V E M D D N F E F G L C P C D A K P L
BVDV-XH32-E2

GGTAAGGGGAAAATTTAATACAACACTTCTAAATGGACCAGCCTTCCAGATGGTTTGCCCTATAGGATGGACAGGGACTGTAAGCTGTACACTGG   1235
CCATTCCCCTTTTAAATTATGTTGTGAAGATTTACCTGGTCGGAAGGTCTACCAAACGGGATATCCTACCTGTCCCTGACATTCGACATGTGACC
    150         155         160         165         170         175
V R G K F N T T L L N G P A F Q M V C P I G W T G T V S C T L
BVDV-XH32-E2

CCAATGAAGATACGTTAGCCACAACCGTTGTGCGAACATATAAGAGGGTCAGGCCTTTTCCATATAGGCAGGACTGTGTCACACAGAAAACCATT   1330
GGTTACTTCTATGCAATCGGTGTTGGCAACACGCTTGTATATTCTCCCAGTCCGGAAAAGGTATATCCGTCCTGACACAGTGTGTCTTTTGGTAA
  180         185         190         195         200         205         210
A N E D T L A T T V V R T Y K R V R P F P Y R Q D C V T Q K T I
BVDV-XH32-E2

GGGGAAGACCTCTATGACTGTGCCCTAGGAGGGAATTGGACCTGTGTGTTGGGGGATATACTACGATATGTAGCTGGGCCTGTCGAGTCTTGTAA   1425
CCCCTTCTGGAGATACTGACACGGGATCCTCCCTTAACCTGGACACACAACCCCCTATATGATGCTATACATCGACCCGGACAGCTCAGAACATT
         215         220         225         230         235         240
G E D L Y D C A L G G N W T C V L G D I L R Y V A G P V E S C K
BVDV-XH32-E2

GTGGTGTGGTCACAAATTTCTTAAAAGTGAGGGTCTGCCGCATTTCCCAATTGGCAAATGCAGGCTGAAGAATGAGAGTGGCTATAGACTAGTGG   1520
CACCACACCAGTGTTTAAAGAATTTTCACTCCCAGACGGCGTAAAGGGTTAACCGTTTACGTCCGACTTCTTACTCTCACCGATATCTGATCACC
    245         250         255         260         265         270
W C G H K F L K S E G L P H F P I G K C R L K N E S G Y R L V
BVDV-XH32-E2

ATGGGACTTCTTGCAACAGAGACGGCGTGGCTATAGTGCCATCTGGCCGGGTCAAATGCAAAATAGGAGACACAGTGGTGCAGGTCATTGCAACG   1615
TACCCTGAAGAACGTTGTCTCTGCCGCACCGATATCACGGTAGACCGGCCCAGTTTACGTTTTATCCTCTGTGTCACCACGTCCAGTAACGTTGC
  275         280         285         290         295         300         305
D G T S C N R D G V A I V P S G R V K C K I G D T V V Q V I A T
BVDV-XH32-E2

                                            NdeI
GATGATAAACTAGGGCCCATGCCTTGCAAACCATATGAAATCATTTCCAGTGAGGGGCCGGTAAAAAAGACGGCATGCACCTTCAACTACACAAG   1710
CTACTATTTGATCCCGGGTACGGAACGTTTGGTATACTTTAGTAAAGGTCACTCCCCGGCCATTTTTTCTGCCGTACGTGGAAGTTGATGTGTTC
         310         315         320         325         330         335
D D K L G P M P C K P Y E I I S S E G P V K K T A C T F N Y T R
BVDV-XH32-E2

AACATTAAAAAACAAGTACTTTGAGCCTAGGGACAACTATTTCCAACAATACATGTTAAAGGGGGAGTACCAATACTGGTTTGACCTAGAGATCC   1805
TTGTAATTTTTTGTTCATGAAACTCGGATCCCTGTTGATAAAGGTTGTTATGTACAATTTCCCCCTCATGGTTATGACCAAACTGGATCTCTAGG
    340         345         350         355         360         365
T L K N K Y F E P R D N Y F Q Q Y M L K G E Y Q Y W F D L E I
BVDV-XH32-E2                                                       6xHis
```

EcoRI EcoRV **NotI** **XhoI**

pcDNA3.1-EcoR I-LF

GAATTCTGCAGATATCCAGCACAGTGGCGGCCGCTCG

ATCATCACCATCACCATTAAGAATTCTGCAGATATCCAGCACAGTGGCGGCCGCTCGAGTCTAGAGGGCCCGTTTAAACCCGCTGATCAGCCTCG 1900

TAGTAGTGGTAGTGGTAATTCTTAAGACGTCTATAGGTCGTGTCACCGCCGGCGAGCTCAGATCTCCCGGGCAAATTTGGGCGACTAGTCGGAGC

1 5

H H H H H H

6xHis

EcoR I+15 bp

CTTAAGACGTCTATAGGTCGT

pcDNA3.1-EcoR I-phu R

GGAGC

Sequencing Primer: BGH-r

ACTGTGCCTTCTAGTTGCCAGCCATCTGTTGTTTGCCCCTCCCCCGTGCCTTCCTTGACCCTGGAAGGTGCCACTCCCACTGTCCTTTCCTAATA 1995

TGACACGGAAGATCAACGGTCGGTAGACAACAAACGGGGAGGGGGCACGGAAGGAACTGGGACCTTCCACGGTGAGGGTGACAGGAAAGGATTAT

bGH poly(A) signal

TGACACGGAAGAT

Sequencing Primer: BGH-r

AAATGAGGAAATTGCATCGCATTGTCTGAGTAGGTGTCATTCTATTCTGGGGGGTGGGGTGGGGCAGGACAGCAAGGGGGAGGATTGGGAAGACA 2090

TTTACTCCTTTAACGTAGCGTAACAGACTCATCCACAGTAAGATAAGACCCCCCACCCCACCCCGTCCTGTCGTTCCCCCTCCTAACCCTTCTGT

bGH poly(A) signal

ATAGCAGGCATGCTGGGGATGCGGTGGGCTCTATGGCTTCTGAGGCGGAAAGAACCAGCTGGGGCTCTAGGGGGTATCCCCACGCGCCCTGTAGC 2185

TATCGTCCGTACGACCCCTACGCCACCCGAGATACCGAAGACTCCGCCTTTCTTGGTCGACCCCGAGATCCCCCATAGGGGTGCGCGGGACATCG

bGH poly(A) signal

f1 ori

GGCGCATTAAGCGCGGCGGGTGTGGTGGTTACGCGCAGCGTGACCGCTACACTTGCCAGCGCCCTAGCGCCCGCTCCTTTCGCTTTCTTCCCTTC 2280

CCGCGTAATTCGCGCCGCCCACACCACCAATGCGCGTCGCACTGGCGATGTGAACGGTCGCGGGATCGCGGGCGAGGAAAGCGAAAGAAGGGAAG

f1 ori

CTTTCTCGCCACGTTCGCCGGCTTTCCCCGTCAAGCTCTAAATCGGGGGCTCCCTTTAGGGTTCCGATTTAGTGCTTTACGGCACCTCGACCCCA 2375

GAAAGAGCGGTGCAAGCGGCCGAAAGGGGCAGTTCGAGATTTAGCCCCCGAGGGAAATCCCAAGGCTAAATCACGAAATGCCGTGGAGCTGGGGT

f1 ori

AAAAACTTGATTAGGGTGATGGTTCACGTAGTGGGCCATCGCCCTGATAGACGGTTTTTCGCCCTTTGACGTTGGAGTCCACGTTCTTTAATAGT 2470

TTTTTGAACTAATCCCACTACCAAGTGCATCACCCGGTAGCGGGACTATCTGCCAAAAAGCGGGAAACTGCAACCTCAGGTGCAAGAAATTATCA

f1 ori

GGACTCTTGTTCCAAACTGGAACAACACTCAACCCTATCTCGGTCTATTCTTTTGATTTATAAGGGATTTTGCCGATTTCGGCCTATTGGTTAAA 2565

CCTGAGAACAAGGTTTGACCTTGTTGTGAGTTGGGATAGAGCCAGATAAGAAAACTAAATATTCCCTAAAACGGCTAAAGCCGGATAACCAATTT

f1 ori

AAATGAGCTGATTTAACAAAAATTTAACGCGAATTAATTCTGTGGAATGTGTGTCAGTTAGGGTGTGGAAAGTCCCCAGGCTCCCCAGCAGGCAG 2660

TTTACTCGACTAAATTGTTTTTAAATTGCGCTTAATTAAGACACCTTACACACAGTCAATCCCACACCTTTCAGGGGTCCGAGGGGTCGTCCGTC

f1 ori

SV40 promoter

AAGTATGCAAAGCATGCATCTCAATTAGTCAGCAACCAGGTGTGGAAAGTCCCCAGGCTCCCCAGCAGGCAGAAGTATGCAAAGCATGCATCTCA 2755

TTCATACGTTTCGTACGTAGAGTTAATCAGTCGTTGGTCCACACCTTTCAGGGGTCCGAGGGGTCGTCCGTCTTCATACGTTTCGTACGTAGAGT

SV40 promoter

ATTAGTCAGCAACCATAGTCCCGCCCCTAACTCCGCCCATCCCGCCCCTAACTCCGCCCAGTTCCGCCCATTCTCCGCCCCATGGCTGACTAATT 2850

TAATCAGTCGTTGGTATCAGGGCGGGGATTGAGGCGGGTAGGGCGGGGATTGAGGCGGGTCAAGGCGGGTAAGAGGCGGGGTACCGACTGATTAA

SV40 promoter

SV40 ori

TTTTTTATTTATGCAGAGGCCGAGGCCGCCTCTGCCTCTGAGCTATTCCAGAAGTAGTGAGGAGGCTTTTTTGGAGGCCTAGGCTTTTGCAAAAA 2945
AAAAAATAAATACGTCTCCGGCTCCGGCGGAGACGGAGACTCGATAAGGTCTTCATCACTCCTCCGAAAAAACCTCCGGATCCGAAAACGTTTTT
SV40 promoter
SV40 ori

GCTCCCGGGAGCTTGTATATCCATTTTCGGATCTGATCAAGAGACAGGATGAGGATCGTTTCGCATGATTGAACAAGATGGATTGCACGCAGGTT 3040
CGAGGGCCCTCGAACATATAGGTAAAAGCCTAGACTAGTTCTCTGTCCTACTCCTAGCAAAGCGTACTAACTTGTTCTACCTAACGTGCGTCCAA
1 5 10
M I E Q D G L H A G
NeoR/KanR

CTCCGGCCGCTTGGGTGGAGAGGCTATTCGGCTATGACTGGGCACAACAGACAATCGGCTGCTCTGATGCCGCCGTGTTCCGGCTGTCAGCGCAG 3135
GAGGCCGGCGAACCCACCTCTCCGATAAGCCGATACTGACCCGTGTTGTCTGTTAGCCGACGAGACTACGGCGGCACAAGGCCGACAGTCGCGTC
15 20 25 30 35 40
S P A A W V E R L F G Y D W A Q Q T I G C S D A A V F R L S A Q
NeoR/KanR

GGGCGCCCGGTTCTTTTTGTCAAGACCGACCTGTCCGGTGCCCTGAATGAACTGCAGGACGAGGCAGCGCGGCTATCGTGGCTGGCCACGACGGG 3230
CCCGCGGGCCAAGAAAAACAGTTCTGGCTGGACAGGCCACGGGACTTACTTGACGTCCTGCTCCGTCGCGCCGATAGCACCGACCGGTGCTGCCC
45 50 55 60 65 70
G R P V L F V K T D L S G A L N E L Q D E A A R L S W L A T T G
NeoR/KanR

CGTTCCTTGCGCAGCTGTGCTCGACGTTGTCACTGAAGCGGGAAGGGACTGGCTGCTATTGGGCGAAGTGCCGGGGCAGGATCTCCTGTCATCTC 3325
GCAAGGAACGCGTCGACACGAGCTGCAACAGTGACTTCGCCCTTCCCTGACCGACGATAACCCGCTTCACGGCCCCGTCCTAGAGGACAGTAGAG
75 80 85 90 95 100 105
V P C A A V L D V V T E A G R D W L L L G E V P G Q D L L S S
NeoR/KanR

ACCTTGCTCCTGCCGAGAAAGTATCCATCATGGCTGATGCAATGCGGCGGCTGCATACGCTTGATCCGGCTACCTGCCCATTCGACCACCAAGCG 3420
TGGAACGAGGACGGCTCTTTCATAGGTAGTACCGACTACGTTACGCCGCCGACGTATGCGAACTAGGCCGATGGACGGGTAAGCTGGTGGTTCGC
110 115 120 125 130 135
H L A P A E K V S I M A D A M R R L H T L D P A T C P F D H Q A
NeoR/KanR

AAACATCGCATCGAGCGAGCACGTACTCGGATGGAAGCCGGTCTTGTCGATCAGGATGATCTGGACGAAGAGCATCAGGGGCTCGCGCCAGCCGA 3515
TTTGTAGCGTAGCTCGCTCGTGCATGAGCCTACCTTCGGCCAGAACAGCTAGTCCTACTAGACCTGCTTCTCGTAGTCCCCGAGCGCGGTCGGCT
140 145 150 155 160 165
K H R I E R A R T R M E A G L V D Q D D L D E E H Q G L A P A E
NeoR/KanR

ACTGTTCGCCAGGCTCAAGGCGCGCATGCCCGACGGCGAGGATCTCGTCGTGACCCATGGCGATGCCTGCTTGCCGAATATCATGGTGGAAAATG 3610
TGACAAGCGGTCCGAGTTCCGCGCGTACGGGCTGCCGCTCCTAGAGCAGCACTGGGTACCGCTACGGACGAACGGCTTATAGTACCACCTTTTAC
170 175 180 185 190 195 200
L F A R L K A R M P D G E D L V V T H G D A C L P N I M V E N
NeoR/KanR

GCCGCTTTTCTGGATTCATCGACTGTGGCCGGCTGGGTGTGGCGGACCGCTATCAGGACATAGCGTTGGCTACCCGTGATATTGCTGAAGAGCTT 3705
CGGCGAAAAGACCTAAGTAGCTGACACCGGCCGACCCACACCGCCTGGCGATAGTCCTGTATCGCAACCGATGGGCACTATAACGACTTCTCGAA
205 210 215 220 225 230
G R F S G F I D C G R L G V A D R Y Q D I A L A T R D I A E E L
NeoR/KanR

GGCGGCGAATGGGCTGACCGCTTCCTCGTGCTTTACGGTATCGCCGCTCCCGATTCGCAGCGCATCGCCTTCTATCGCCTTCTTGACGAGTTCTT 3800
CCGCCGCTTACCCGACTGGCGAAGGAGCACGAAATGCCATAGCGGCGAGGGCTAAGCGTCGCGTAGCGGAAGATAGCGGAAGAACTGCTCAAGAA
235 240 245 250 255 260
G G E W A D R F L V L Y G I A A P D S Q R I A F Y R L L D E F F
NeoR/KanR

CTGAGCGGGACTCTGGGGTTCGAAATGACCGACCAAGCGACGCCCAACCTGCCATCACGAGATTTCGATTCCACCGCCGCCTTCTATGAAAGGTT 3895
GACTCGCCCTGAGACCCCAAGCTTTACTGGCTGGTTCGCTGCGGGTTGGACGGTAGTGCTCTAAAGCTAAGGTGGCGGCGGAAGATACTTTCCAA
265
*
NeoR/KanR

GGGCTTCGGAATCGTTTTCCGGGACGCCGGCTGGATGATCCTCCAGCGCGGGGATCTCATGCTGGAGTTCTTCGCCCACCCCAACTTGTTTATTG 3990
CCCGAAGCCTTAGCAAAAGGCCCTGCGGCCGACCTACTAGGAGGTCGCGCCCCTAGAGTACGACCTCAAGAAGCGGGTGGGGTTGAACAAATAAC
SV40 poly(A) signal

CAGCTTATAATGGTTACAAATAAAGCAATAGCATCACAAATTTCACAAATAAAGCATTTTTTTCACTGCATTCTAGTTGTGGTTTGTCCAAACTC 4085
GTCGAATATTACCAATGTTTATTTCGTTATCGTAGTGTTTAAAGTGTTTATTTCGTAAAAAAAGTGACGTAAGATCAACACCAAACAGGTTTGAG
SV40 poly(A) signal

ATCAATGTATCTTATCATGTCTGTATACCGTCGACCTCTAGCTAGAGCTTGGCGTAATCATGGTCATAGCTGTTTCCTGTGTGAAATTGTTATCC 4180
TAGTTACATAGAATAGTACAGACATATGGCAGCTGGAGATCGATCTCGAACCGCATTAGTACCAGTATCGACAAAGGACACACTTTAACAATAGG
SV40 poly(A) signal | M13 rev | lac operator

GCTCACAATTCCACACAACATACGAGCCGGAAGCATAAAGTGTAAAGCCTGGGGTGCCTAATGAGTGAGCTAACTCACATTAATTGCGTTGCGCT 4275
CGAGTGTTAAGGTGTGTTGTATGCTCGGCCTTCGTATTTCACATTTCGGACCCCACGGATTACTCACTCGATTGAGTGTAATTAACGCAACGCGA
lac operator | -10 | lac promoter | -35 | CAP binding site

CACTGCCCGCTTTCCAGTCGGGAAACCTGTCGTGCCAGCTGCATTAATGAATCGGCCAACGCGCGGGGAGAGGCGGTTTGCGTATTGGGCGCTCT 4370
GTGACGGGCGAAAGGTCAGCCCTTTGGACAGCACGGTCGACGTAATTACTTAGCCGGTTGCGCGCCCCTCTCCGCCAAACGCATAACCCGCGAGA

TCCGCTTCCTCGCTCACTGACTCGCTGCGCTCGGTCGTTCGGCTGCGGCGAGCGGTATCAGCTCACTCAAAGGCGGTAATACGGTTATCCACAGA 4465
AGGCGAAGGAGCGAGTGACTGAGCGACGCGAGCCAGCAAGCCGACGCCGCTCGCCATAGTCGAGTGAGTTTCCGCCATTATGCCAATAGGTGTCT

ATCAGGGGATAACGCAGGAAAGAACATGTGAGCAAAAGGCCAGCAAAAGGCCAGGAACCGTAAAAAGGCCGCGTTGCTGGCGTTTTTCCATAGGC 4560
TAGTCCCCTATTGCGTCCTTTCTTGTACACTCGTTTTCCGGTCGTTTTCCGGTCCTTGGCATTTTTCCGGCGCAACGACCGCAAAAAGGTATCCG
ori

TCCGCCCCCCTGACGAGCATCACAAAAATCGACGCTCAAGTCAGAGGTGGCGAAACCCGACAGGACTATAAAGATACCAGGCGTTTCCCCCTGGA 4655
AGGCGGGGGGACTGCTCGTAGTGTTTTTAGCTGCGAGTTCAGTCTCCACCGCTTTGGGCTGTCCTGATATTTCTATGGTCCGCAAAGGGGGACCT
ori

AGCTCCCTCGTGCGCTCTCCTGTTCCGACCCTGCCGCTTACCGGATACCTGTCCGCCTTTCTCCCTTCGGGAAGCGTGGCGCTTTCTCATAGCTC 4750
TCGAGGGAGCACGCGAGAGGACAAGGCTGGGACGGCGAATGGCCTATGGACAGGCGGAAAGAGGGAAGCCCTTCGCACCGCGAAAGAGTATCGAG
ori

ACGCTGTAGGTATCTCAGTTCGGTGTAGGTCGTTCGCTCCAAGCTGGGCTGTGTGCACGAACCCCCCGTTCAGCCCGACCGCTGCGCCTTATCCG 4845
TGCGACATCCATAGAGTCAAGCCACATCCAGCAAGCGAGGTTCGACCCGACACACGTGCTTGGGGGGCAAGTCGGGCTGGCGACGCGGAATAGGC
ori

GTAACTATCGTCTTGAGTCCAACCCGGTAAGACACGACTTATCGCCACTGGCAGCAGCCACTGGTAACAGGATTAGCAGAGCGAGGTATGTAGGC 4940
CATTGATAGCAGAACTCAGGTTGGGCCATTCTGTGCTGAATAGCGGTGACCGTCGTCGGTGACCATTGTCCTAATCGTCTCGCTCCATACATCCG
ori

GGTGCTACAGAGTTCTTGAAGTGGTGGCCTAACTACGGCTACACTAGAAGAACAGTATTTGGTATCTGCGCTCTGCTGAAGCCAGTTACCTTCGG 5035
CCACGATGTCTCAAGAACTTCACCACCGGATTGATGCCGATGTGATCTTCTTGTCATAAACCATAGACGCGAGACGACTTCGGTCAATGGAAGCC
ori

AAAAAGAGTTGGTAGCTCTTGATCCGGCAAACAAACCACCGCTGGTAGCGGTTTTTTTGTTTGCAAGCAGCAGATTACGCGCAGAAAAAAAGGAT 5130
TTTTTCTCAACCATCGAGAACTAGGCCGTTTGTTTGGTGGCGACCATCGCCAAAAAAACAAACGTTCGTCGTCTAATGCGCGTCTTTTTTTCCTA
ori

CTCAAGAAGATCCTTTGATCTTTTCTACGGGGTCTGACGCTCAGTGGAACGAAAACTCACGTTAAGGGATTTTGGTCATGAGATTATCAAAAAGG 5225
GAGTTCTTCTAGGAAACTAGAAAAGATGCCCCAGACTGCGAGTCACCTTGCTTTTGAGTGCAATTCCCTAAAACCAGTACTCTAATAGTTTTTCC
ori

ATCTTCACCTAGATCCTTTTAAATTAAAAATGAAGTTTTAAATCAATCTAAAGTATATATGAGTAAACTTGGTCTGACAGTTACCAATGCTTAAT 5320
TAGAAGTGGATCTAGGAAAATTTAATTTTTACTTCAAAATTTAGTTAGATTTCATATATACTCATTTGAACCAGACTGTCAATGGTTACGAATTA

285
* W H K I
AmpR

CAGTGAGGCACCTATCTCAGCGATCTGTCTATTTCGTTCATCCATAGTTGCCTGACTCCCCGTCGTGTAGATAACTACGATACGGGAGGGCTTAC 5415
GTCACTCCGTGGATAGAGTCGCTAGACAGATAAAGCAAGTAGGTATCAACGGACTGAGGGGCAGCACATCTATTGATGCTATGCCCTCCCGAATG

280 275 270 265 260 255
L S A G I E A I Q R N R E D M T A Q S G T T Y I V V I R S P K G
AmpR

CATCTGGCCCCAGTGCTGCAATGATACCGCGAGACCCACGCTCACCGGCTCCAGATTTATCAGCAATAAACCAGCCAGCCGGAAGGGCCGAGCGC 5510
GTAGACCGGGGTCACGACGTTACTATGGCGCTCTGGGTGCGAGTGGCCGAGGTCTAAATAGTCGTTATTTGGTCGGTCGGCCTTCCCGGCTCGCG

250 245 240 235 230 225 220
D P G L A A I I G R S G R E G A G S K D A I F W G A P L A S R
AmpR

AGAAGTGGTCCTGCAACTTTATCCGCCTCCATCCAGTCTATTAATTGTTGCCGGGAAGCTAGAGTAAGTAGTTCGCCAGTTAATAGTTTGCGCAA 5605
TCTTCACCAGGACGTTGAAATAGGCGGAGGTAGGTCAGATAATTAACAACGGCCCTTCGATCTCATTCATCAAGCGGTCAATTATCAAACGCGTT

215 210 205 200 195 190
L L P G A V K D A E M W D I L Q Q R S A L T L L E G T L L K R L
AmpR

CGTTGTTGCCATTGCTACAGGCATCGTGGTGTCACGCTCGTCGTTTGGTATGGCTTCATTCAGCTCCGGTTCCCAACGATCAAGGCGAGTTACAT 5700
GCAACAACGGTAACGATGTCCGTAGCACCACAGTGCGAGCAGCAAACCATACCGAAGTAAGTCGAGGCCAAGGGTTGCTAGTTCCGCTCAATGTA

185 180 175 170 165 160
T T A M A V P M T T D R E D N P I A E N L E P E W R D L R T V H
AmpR

GATCCCCCATGTTGTGCAAAAAAGCGGTTAGCTCCTTCGGTCCTCCGATCGTTGTCAGAAGTAAGTTGGCCGCAGTGTTATCACTCATGGTTATG 5795
CTAGGGGGTACAACACGTTTTTTCGCCAATCGAGGAAGCCAGGAGGCTAGCAACAGTCTTCATTCAACCGGCGTCACAATAGTGAGTACCAATAC

155 150 145 140 135 130 125
D G M N H L F A T L E K P G G I T T L L L N A A T N D S M T I
AmpR

GCAGCACTGCATAATTCTCTTACTGTCATGCCATCCGTAAGATGCTTTTCTGTGACTGGTGAGTACTCAACCAAGTCATTCTGAGAATAGTGTAT 5890
CGTCGTGACGTATTAAGAGAATGACAGTACGGTAGGCATTCTACGAAAAGACACTGACCACTCATGAGTTGGTTCAGTAAGACTCTTATCACATA

120 115 110 105 100 95
A A S C L E R V T M G D T L H K E T V P S Y E V L D N Q S Y H I
AmpR

GCGGCGACCGAGTTGCTCTTGCCCGGCGTCAATACGGGATAATACCGCGCCACATAGCAGAACTTTAAAAGTGCTCATCATTGGAAAACGTTCTT 5985
CGCCGCTGGCTCAACGAGAACGGGCCGCAGTTATGCCCTATTATGGCGCGGTGTATCGTCTTGAAATTTTCACGAGTAGTAACCTTTTGCAAGAA

90 85 80 75 70 65
R R G L Q E Q G A D I R S L V A G C L L V K F T S M M P F R E E
AmpR

CGGGGCGAAAACTCTCAAGGATCTTACCGCTGTTGAGATCCAGTTCGATGTAACCCACTCGTGCACCCAACTGATCTTCAGCATCTTTTACTTTC 6080
GCCCCGCTTTTGAGAGTTCCTAGAATGGCGACAACTCTAGGTCAAGCTACATTGGGTGAGCACGTGGGTTGACTAGAAGTCGTAGAAAATGAAAG

60 55 50 45 40 35 30
P R F S E L I K G S N L D L E I Y G V R A G L Q D E A D K V K
AmpR

ACCAGCGTTTCTGGGTGAGCAAAAACAGGAAGGCAAAATGCCGCAAAAAAGGGAATAAGGGCGACACGGAAATGTTGAATACTCATACTCTTCCT 6175
TGGTCGCAAAGACCCACTCGTTTTTGTCCTTCCGTTTTACGGCGTTTTTTCCCTTATTCCCGCTGTGCCTTTACAACTTATGAGTATGAGAAGGA

25 20 15 10 5 1
V L T E P H A F V P L C F A A F F P I L A V R F H Q I S M
signal sequence
AmpR
AmpR promoter

```
TTTTCAATATTATTGAAGCATTTATCAGGGTTATTGTCTCATGAGCGGATACATATTTGAATGTATTTAGAAAAATAAACAAATAGGGGTTCCGC   6270
AAAAGTTATAATAACTTCGTAAATAGTCCCAATAACAGAGTACTCGCCTATGTATAAACTTACATAAATCTTTTTATTTGTTTATCCCCAAGGCG
                                    AmpR promoter

GCACATTTCCCCGAAAAGTGCCACCTGACGTCGACGGATCGGGAGATCTCCCGATCCCCTATGGTGCACTCTCAGTACAATCTGCTCTGATGCCG   6365
CGTGTAAAGGGGCTTTTCACGGTGGACTGCAGCTGCCTAGCCCTCTAGAGGGCTAGGGGATACCACGTGAGAGTCATGTTAGACGAGACTACGGC
AmpR promoter

CATAGTTAAGCCAGTATCTGCTCCCTGCTTGTGTGTTGGAGGTCGCTGAGTAGTGCGCGAGCAAAATTTAAGCTACAACAAGGCAAGGCTTGACC   6460
GTATCAATTCGGTCATAGACGAGGGACGAACACACAACCTCCAGCGACTCATCACGCGCTCGTTTTAAATTCGATGTTGTTCCGTTCCGAACTGG

GACAATTGCATGAAGAATCTGCTTAGGGTTAGGCGTTTTGCGCTGCTTCGCGATGTACGGGCCAGATATACGCGTT   3′
                                                                           ••• 6536
CTGTTAACGTACTTCTTAGACGAATCCCAATCCGCAAAACGCGACGAAGCGCTACATGCCCGGTCTATATGCGCAA   5′
```

3. Sanger sequencing chromatograms of pcDNA3.1-BVDV-XH32-E2

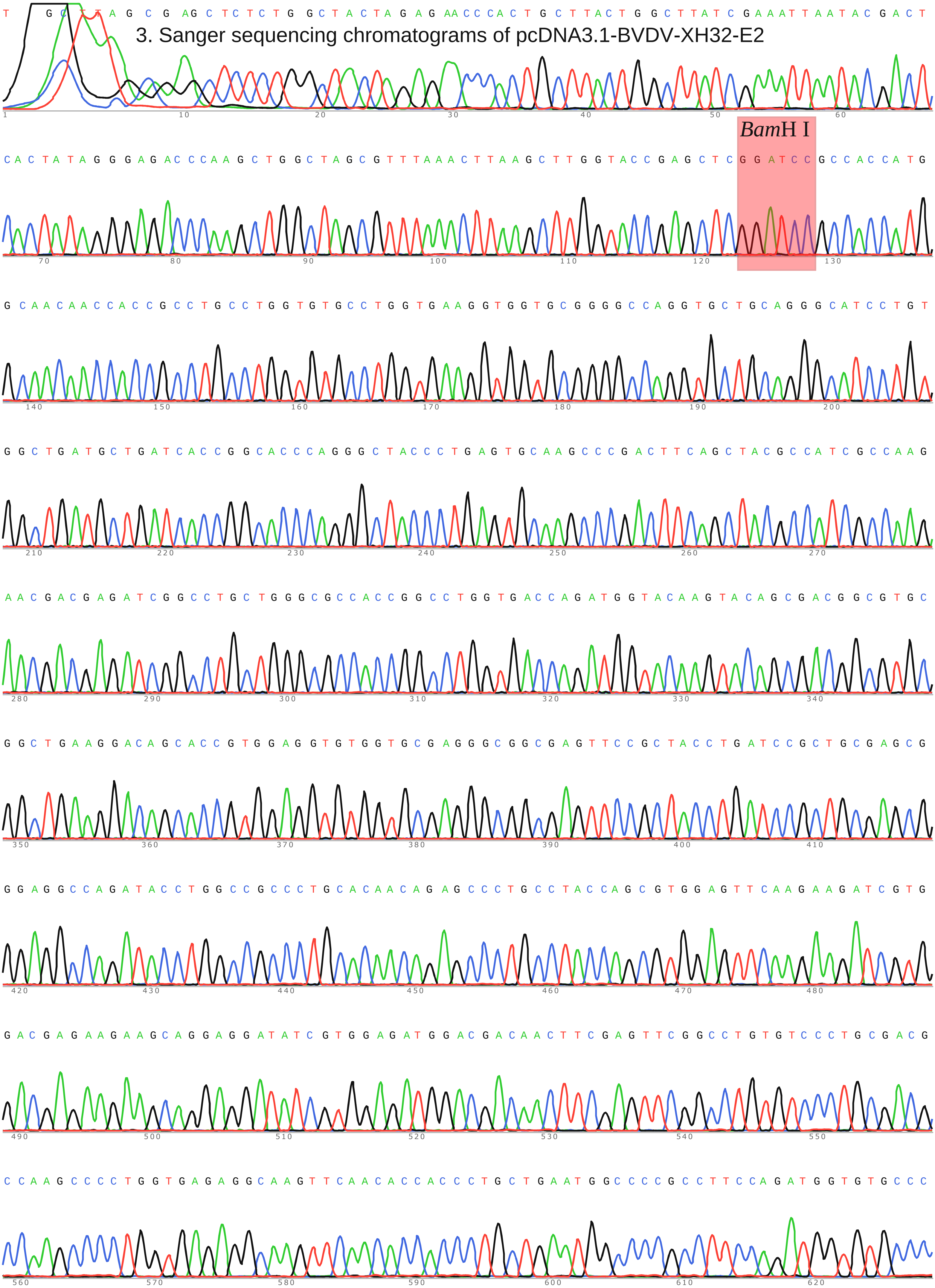

C A T C G G C T G G A C C G G C A C C G T G A G C T G C A C C C T G G C C A A C G A G G A T A C C C T G G C C A C C A C C G T G G T G C G C

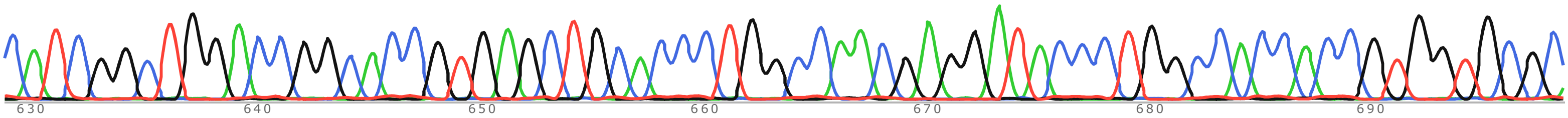

A C C T A C A A G A G G G T G A G A C C C T T C C C C T A C C G G C A G G A C T G C G T G A C C C A G A A A A C C A T C G G C G A G G A C C

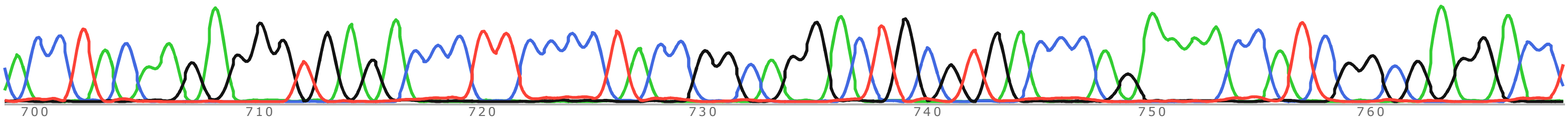

T G T A C G A T T G C G C C C T G G G C G G C A A C T G G A C C T G C G T G C T G G G C G A C A T C C T G A G G T A C G T G G C C G G C C C

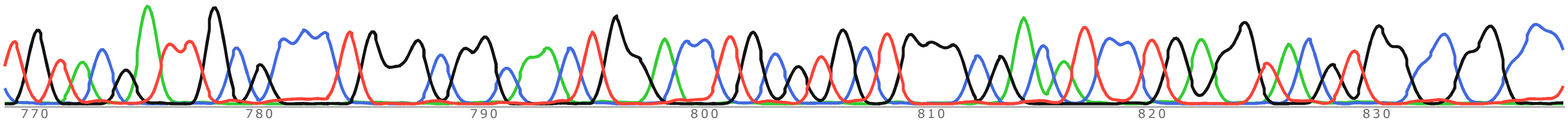

T G T G G A G T C C T G C A A G T G G T G C G G C C A C A A G T T C C T G A A G T C C G A G G G C C T G C C C C A C T T C C C C A T C G G

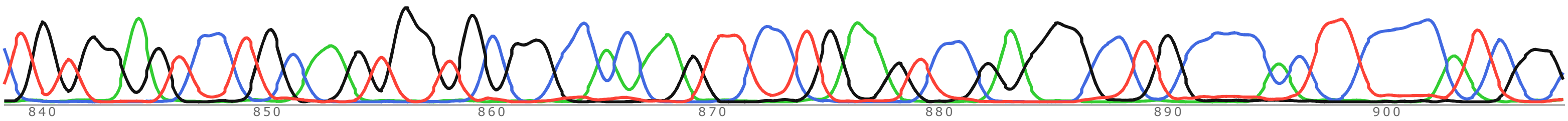

C A A G T G C C G C C T G A A G A A C G A G A G C G G C T A C A G A C T G G T G G A C G G C A C C T C C T G C A A C C G G G A C G G C G T G

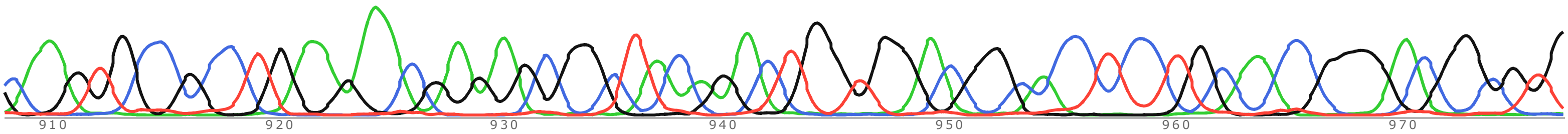

G C C A T C G T G C C C T C C G G C C G G G T G A A G T G C A G A

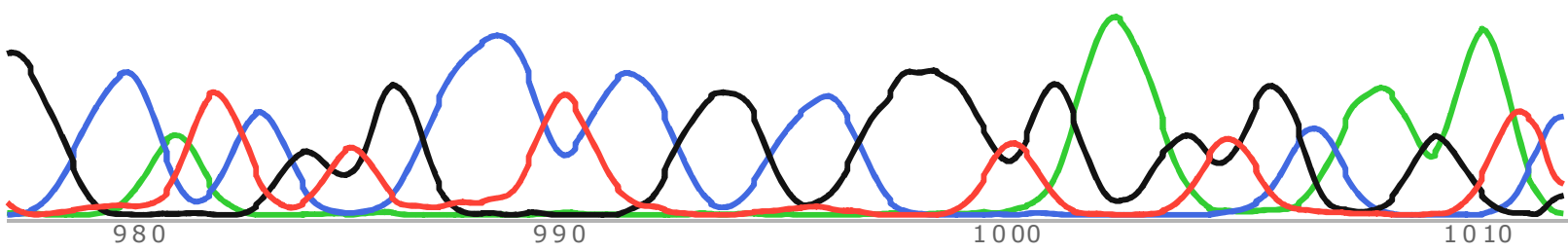

CGCCACCGGCCTGGTGACCAGATGGTACAAGTACAGCGACGGCGTGCGGCTGAAGGACAGCACCGTG

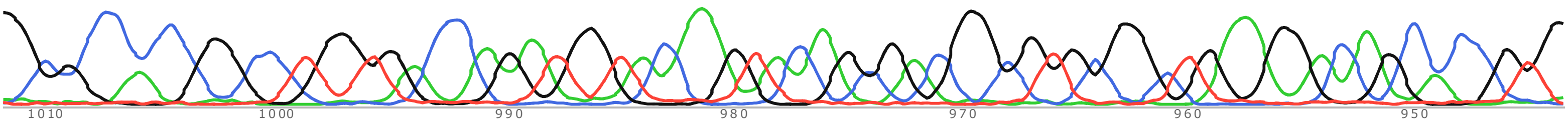

GAGGTGTGGTGCGAGGGCGGCGAGTTCCGCTACCTGATCCGCTGCGAGCGGGAGGCCAGATACCTGGCC

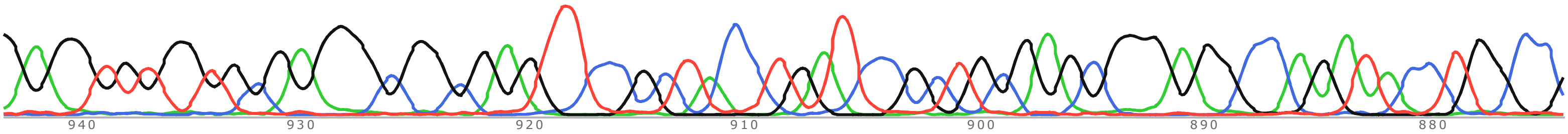

GCCCTGCACAACAGAGCCCTGCCTACCAGCGTGGAGTTCAAGAAGATCGTGGACGAGAAGAAGCAGGAGG

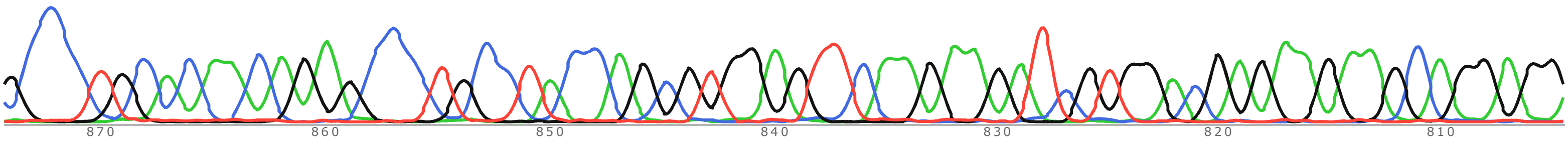

ATATCGTGGAGATGGACGACAACTTCGAGTTCGGCCTGTGTCCCTGCGACGCCAAGCCCCTGGTGAGAGG

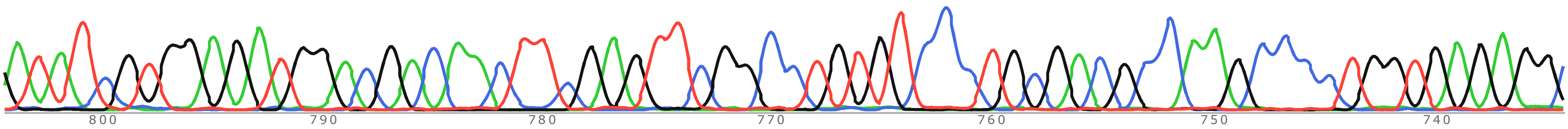

CAAGTTCAACACCACCCTGCTGAATGGCCCCGCCTTCCAGATGGTGTGCCCCATCGGCTGGACCGGCACC

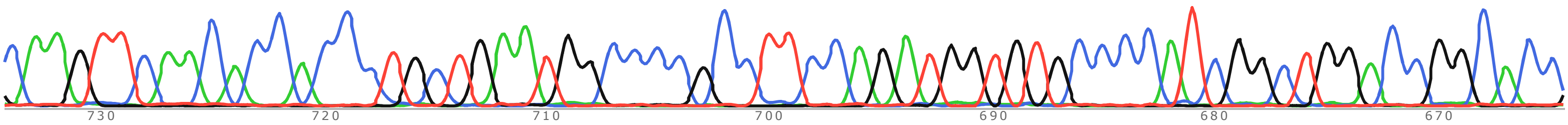

GTGAGCTGCACCCTGGCCAACGAGGATACCCTGGCCACCACCGTGGTGCGCACCTACAAGAGGGTGAGAC

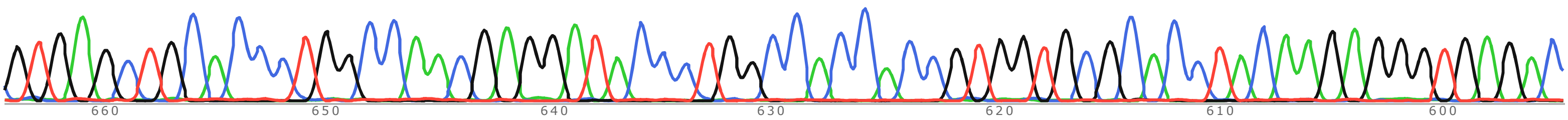

CCTTCCCCTACCGGCAGGACTGCGTGACCCAGAAAACCATCGGCGAGGACCTGTACGATTGCGCCCTGGG

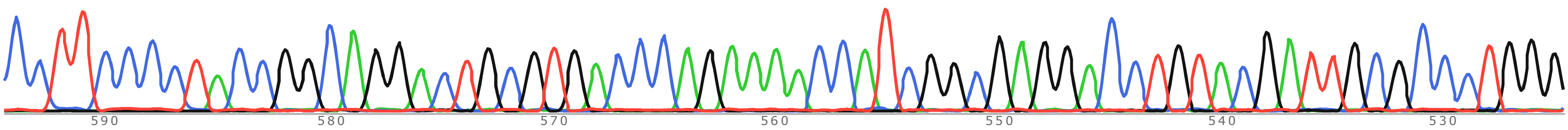

CGGCAACTGGACCTGCGTGCTGGGCGACATCCTGAGGTACGTGGCCGGCCCCTGTGGAGTCCTGCAAGTGG

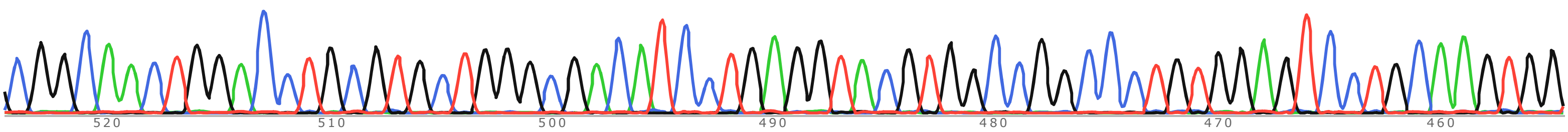

TGCGGCCACAAGTTCCTGAAGTCCGAGGGCCTGCCCCACTTCCCCATCGGCAAGTGCCGCCTGAAGAACG

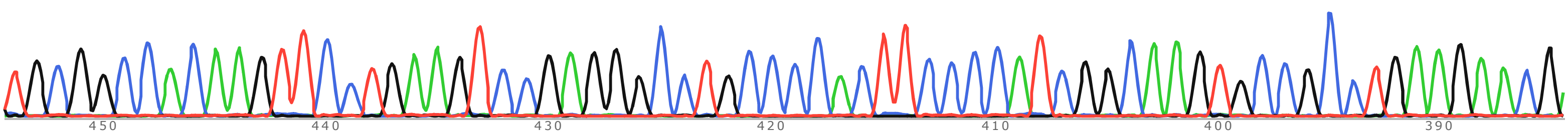

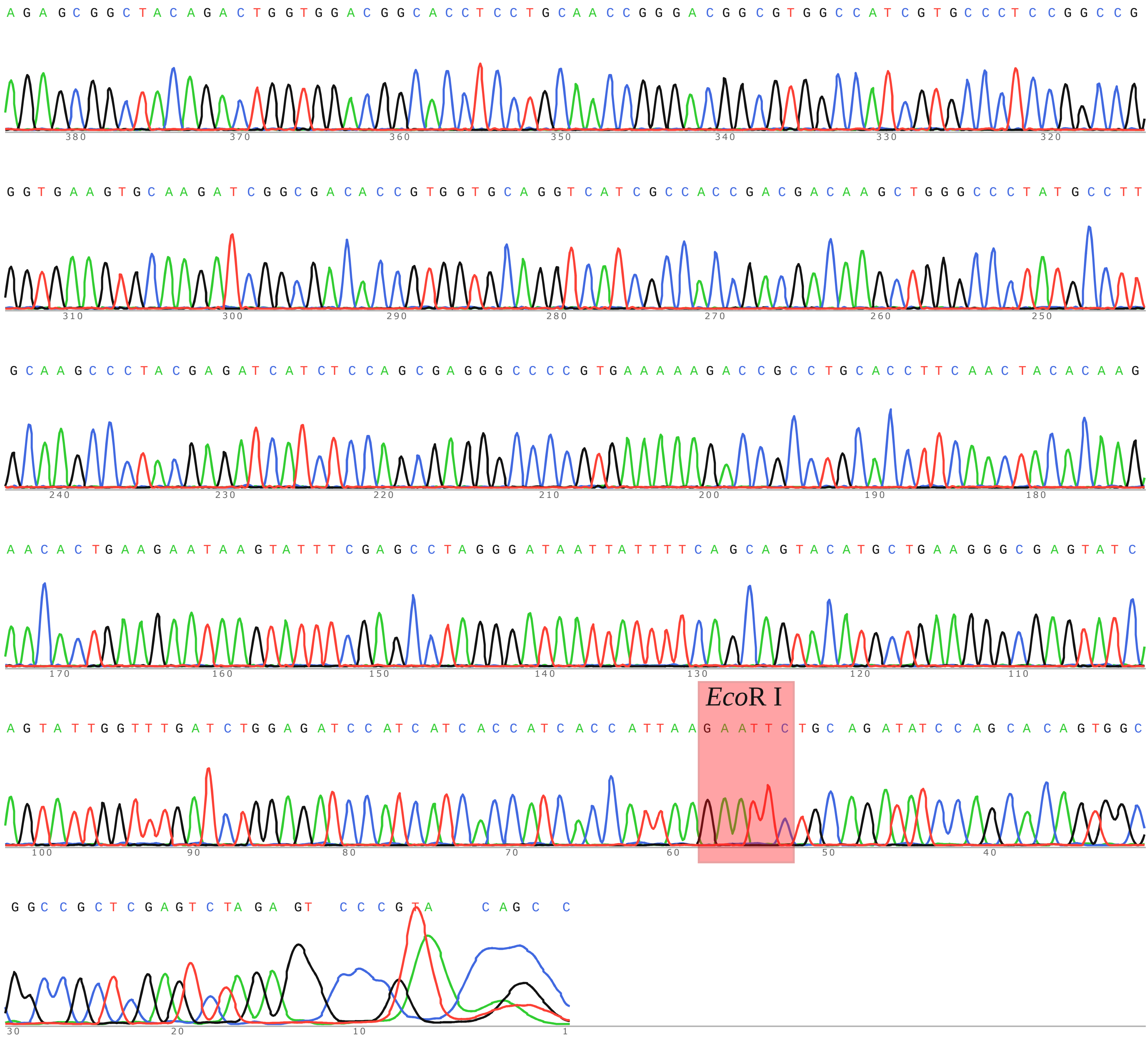
A G A G C G G C T A C A G A C T G G T G G A C G G C A C C T C C T G C A A C C G G G A C G G C G T G G C C A T C G T G C C C T C C G G C C G
G G T G A A G T G C A A G A T C G G C G A C A C C G T G G T G C A G G T C A T C G C C A C C G A C G A C A A G C T G G G C C C T A T G C C T T
G C A A G C C C T A C G A G A T C A T C T C C A G C G A G G G C C C C G T G A A A A A G A C C G C C T G C A C C T T C A A C T A C A C A A G
A A C A C T G A A G A A T A A G T A T T T C G A G C C T A G G G A T A A T T A T T T T C A G C A G T A C A T G C T G A A G G G C G A G T A T C
EcoR I
A G T A T T G G T T T G A T C T G G A G A T C C A T C A T C A C C A T C A C C A T T A A G A A T T C T G C A G A T A T C C A G C A C A G T G G C
G G C C G C T C G A G T C T A G A G T C C C G T A C A G C C
