# Supplementary material for: Generation and epitope mapping of two distinct neutralizing mAb subsets targeting BVDV E2: a pan-pestivirus cross-reactive linear epitope and a BVDV-specific conformational epitope
Source: Vet Res. 2026 Oct 7;57:176. doi: 10.1186/s13567-026-01852-5 (PMC13644284; doi:10.1186/s13567-026-01852-5)
Supplement: Supplementary file 3 [file 13567_2026_1852_MOESM4_ESM.pdf]

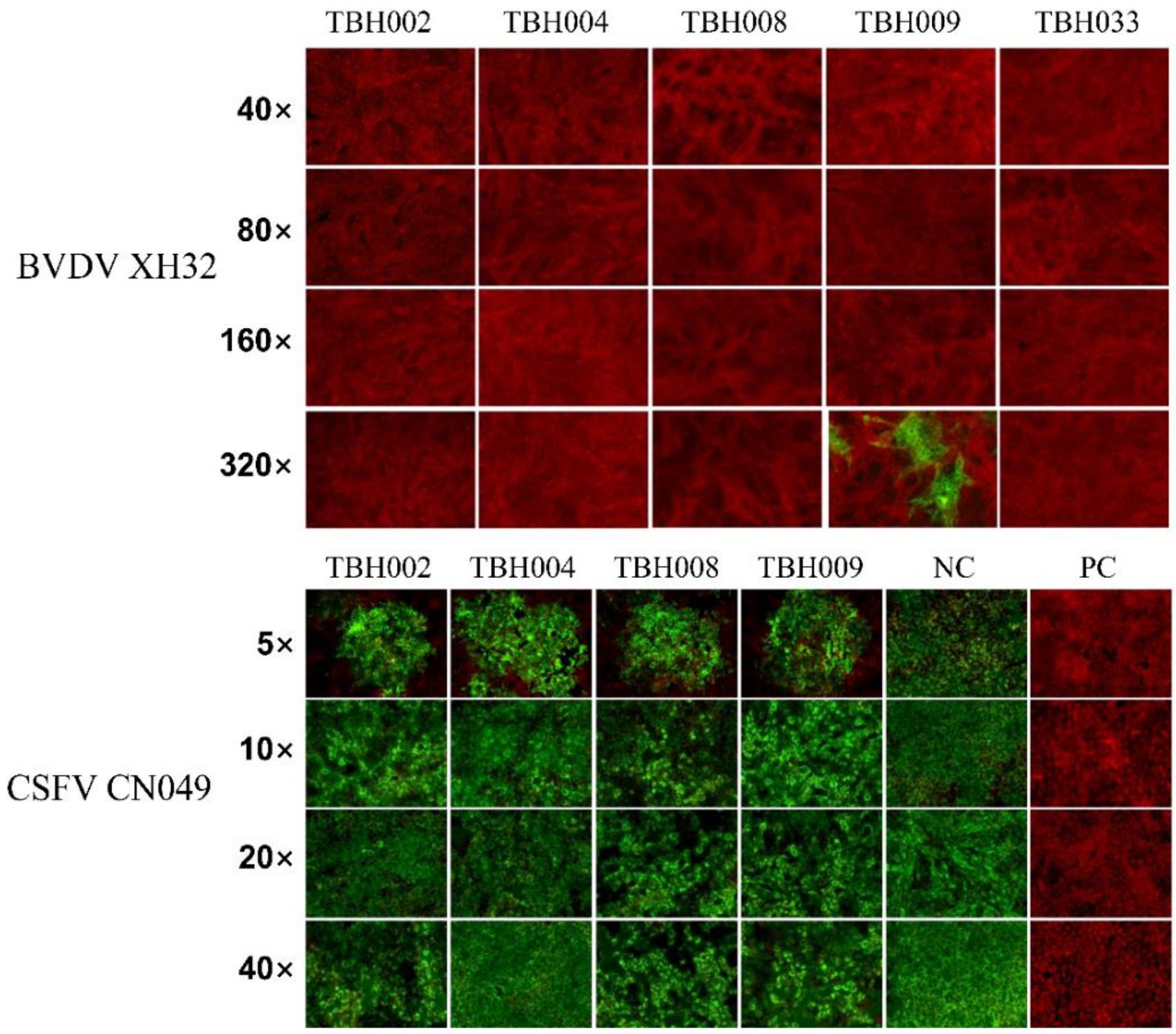

Evaluation of neutralizing activity of ascitic mAbs against BVDV and CSFV. Ascitic mAbs (TBH002, TBH004, TBH008, TBH009, TBH033) were initially 5-fold diluted, followed by serial 2-fold dilutions. Diluted mAbs were incubated with an equal volume of 100 $TCID_{50}$ BVDV XH32 or CSFV CN049 at 37°C for 1 h. The antibody-virus mixtures were then added to MDBK cells, and cultured for 72 h. Cells were fixed with pre-cooled 80% acetone and subjected to IFA. As a result, except for TBH009, the remaining four ascitic mAbs retained neutralizing capacity against BVDV XH32 at a dilution of 1:320. For CSFV CN049, TBH002, TBH004, TBH008 and TBH009 failed to neutralize viral infection even at the 1:5 dilution.
