# Supplementary material for: Generation and epitope mapping of two distinct neutralizing mAb subsets targeting BVDV E2: a pan-pestivirus cross-reactive linear epitope and a BVDV-specific conformational epitope
Source: Vet Res. 2026 Oct 7;57:176. doi: 10.1186/s13567-026-01852-5 (PMC13644284; doi:10.1186/s13567-026-01852-5)
Supplement: Supplementary file 4 [file 13567_2026_1852_MOESM5_ESM.pdf]

Contents

Data of partial colony PCR electrophoresis for specific mutagenesis verification

Series of mutant-E2 vectors were constructed based on pFast Bac1-BVDV-XH32-E2 or pcDNA3.1-BVDV-XH32-E2 using In-Fusion cloning technology. Firstly, the clones were verified by PCR and then the positive clones were characterized by sanger sequencing.

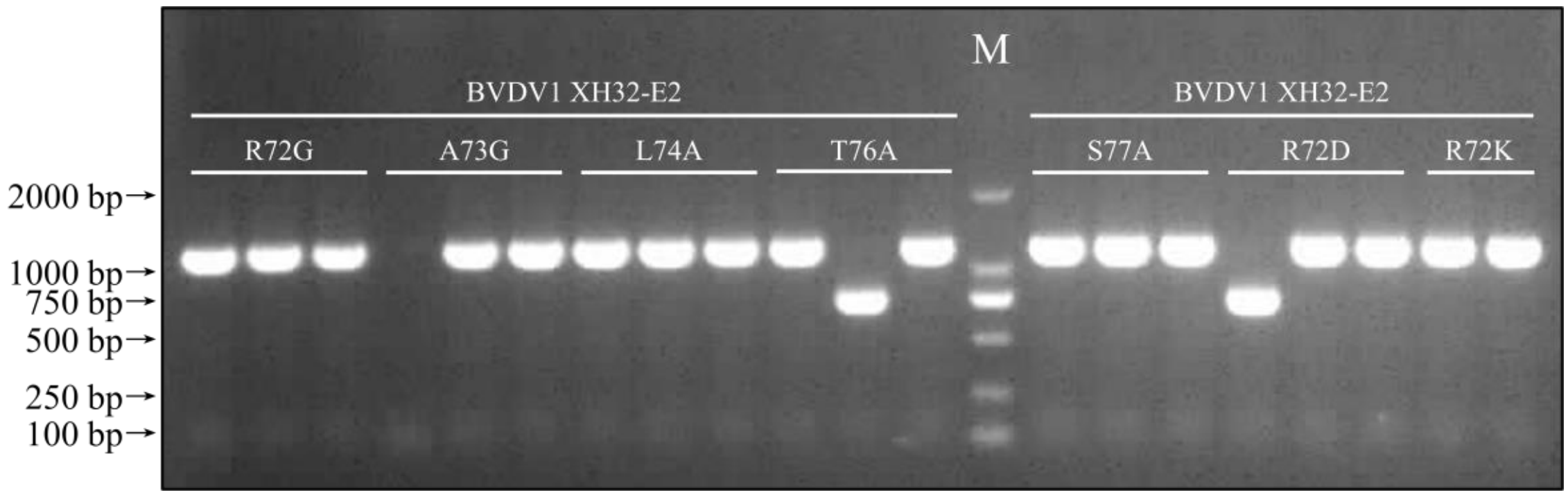

Representative colony PCR electrophoresis validation results of recombinant plasmids expressing site-mutated E2

Contents

Data of Sanger sequencing chromatograms for specific mutagenesis verification

(The following items are attached with page number links. Please click to view. Cloning sites and specific mutation sites are marked with red boxes in the chromatograms)

Sanger sequencing chromatograms data of all relevant mutant plasmids in this research.

Related to **Fig 5-C**

MEP-017-pcDNA3.1-**BVDV-XH32-E2 (R72G)**_pRLuc-MCS-F 1
MEP-017-pcDNA3.1-**BVDV-XH32-E2 (R72G)**_BGH-r 3
MEP-018-pcDNA3.1-**BVDV-XH32-E2 (A73G)**_pRLuc-MCS-F 5
MEP-018-pcDNA3.1-**BVDV-XH32-E2 (A73G)**_BGH-r 7
MEP-019-pcDNA3.1-**BVDV-XH32-E2 (L74A)**_pRLuc-MCS-F 9
MEP-019-pcDNA3.1-**BVDV-XH32-E2 (L74A)**_BGH-r 11
MEP-020-pcDNA3.1-**BVDV-XH32-E2 (T76A)**_pRLuc-MCS-F 13
MEP-020-pcDNA3.1-**BVDV-XH32-E2 (T76A)**_BGH-r 15
MEP-021-pcDNA3.1-**BVDV-XH32-E2 (S77A)**_pRLuc-MCS-F 17
MEP-021-pcDNA3.1-**BVDV-XH32-E2 (S77A)**_BGH-r 19
MEP-028-pcDNA3.1-**BVDV-XH32-E2 (R72D)**_pRluc-MCS-F 21
MEP-028-pcDNA3.1-**BVDV-XH32-E2 (R72D)**_BGH-r 23
MEP-029-pcDNA3.1-**BVDV-XH32-E2 (R72K)**_pRluc-MCS-F 25
MEP-029-pcDNA3.1-**BVDV-XH32-E2 (R72K)**_BGH-r 27
BEP-505-pFsat Bac1-**BVDV-M31182-E2 (D72R)**_poly-F 29
BEP-505-pFsat Bac1-**BVDV-M31182-E2 (D72R)**_SV40pA-R 31

Related to **Fig 5-D**

MEP-048-pcDNA3.1-**BVDV-XH32-E2 (H70Q)**_pRLuc-MCS-F 33
MEP-048-pcDNA3.1-**BVDV-XH32-E2 (H70Q)**_BGH-r 35
MEP-049-pcDNA3.1-**CSFV-CN033-E2**_pRLuc-MCS-F 37
MEP-049-pcDNA3.1-**CSFV-CN033-E2**_BGH-r 39
MEP-050-pcDNA3.1-**CSFV-CN033-E2 (Q70H)**_pRLuc-MCS-F 41
MEP-050-pcDNA3.1-**CSFV-CN033-E2 (Q70H)**_BGH-r 43

Related to **Fig 6-B**

BEP-485-pFsat Bac1-**BVDV-XH32-E2 (P124S)**_poly-F 45
BEP-485-pFsat Bac1-**BVDV-XH32-E2 (P124S)**_SV40pA-R 47
BEP-486-pFsat Bac1-**BVDV-XH32-E2 (Q127Y)**_poly-F 49
BEP-486-pFsat Bac1-**BVDV-XH32-E2 (Q127Y)**_SV40pA-R 51
BEP-487-pFsat Bac1-**BVDV-XH32-E2 (M128L)**_poly-F 53
BEP-487-pFsat Bac1-**BVDV-XH32-E2 (M128L)**_SV40pA-R 55
BEP-488-pFsat Bac1-**BVDV-XH32-E2 (T138V)**_poly-F 57
BEP-488-pFsat Bac1-**BVDV-XH32-E2 (T138V)**_SV40pA-R 59
BEP-489-pFsat Bac1-**BVDV-XH32-E2 (V138 I)**_poly-F 61
BEP-489-pFsat Bac1-**BVDV-XH32-E2 (V138 I)**_SV40pA-R 63

Related to **Fig 6-D**

BEP-490-pFsat Bac1-**BVDV-XH32-E2 (L103G)**_poly-F 65
BEP-490-pFsat Bac1-**BVDV-XH32-E2 (L103G)**_SV40pA-R 67
BEP-491-pFsat Bac1-**BVDV-XH32-E2 (L105A)**_poly-F 69

Contents

Data of Sanger sequencing chromatograms for specific mutagenesis verification

(The following items are attached with page number links. Please click to view. Cloning sites and specific mutation sites are marked with red boxes in the chromatograms)

BEP-491-pFsat Bac1-**BVDV-XH32-E2 (L105A)**_SV40pA-R 71
BEP-492-pFsat Bac1-**BVDV-XH32-E2 (L107A)**_poly-F 73
BEP-492-pFsat Bac1-**BVDV-XH32-E2 (L107A)**_SV40pA-R 75
BEP-493-pFsat Bac1-**BVDV-XH32-E2 (L120A)**_poly-F 77
BEP-493-pFsat Bac1-**BVDV-XH32-E2 (L120A)**_SV40pA-R 79
BEP-494-pFsat Bac1-**BVDV-XH32-E2 (L121A)**_poly-F 81
BEP-494-pFsat Bac1-**BVDV-XH32-E2 (L121A)**_SV40pA-R 83
BEP-495-pFsat Bac1-**BVDV-XH32-E2 (N122A)**_poly-F 85
BEP-495-pFsat Bac1-**BVDV-XH32-E2 (N122A)**_SV40pA-R 87
BEP-496-pFsat Bac1-**BVDV-XH32-E2 (G123L)**_poly-F 89
BEP-496-pFsat Bac1-**BVDV-XH32-E2 (G123L)**_SV40pA-R 91
BEP-497-pFsat Bac1-**BVDV-XH32-E2 (A125G)**_poly-F 93
BEP-497-pFsat Bac1-**BVDV-XH32-E2 (A125G)**_SV40pA-R 95
BEP-499-pFsat Bac1-**CSFV-HCLV-E2 (S124P)**_poly-F 97
BEP-499-pFsat Bac1-**CSFV-HCLV-E2 (S124P)**_SV40pA-R 99
BEP-500-pFsat Bac1-**BVDV-BJ1201-E2 (I103L)**_poly-F 101
BEP-500-pFsat Bac1-**BVDV-BJ1201-E2 (I103L)**_SV40-pA-R 103
BEP-501-pFsat Bac1-**BVDV-UM-111-06-E2 (A124P)**_poly-F 105
BEP-501-pFsat Bac1-**BVDV-UM-111-06-E2 (A124P)**_SV40-pA-R 107
BEP-502-pFsat Bac1-**CSFV-HCLV-E2 (Y127Q, I138V)**_poly-F 109
BEP-502-pFsat Bac1-**CSFV-HCLV-E2 (Y127Q, I138V)**_SV40-pA-R 111
BEP-503-pFsat Bac1-**CSFV-HCLV-E2 (S124P, Y127Q, I138V)**_poly-F 113
BEP-503-pFsat Bac1-**CSFV-HCLV-E2 (S124P, Y127Q, I138V)**_SV40-pA-R 115
MEP-044-pcDNA3.1-**BVDV-XH32-E2 (F126L)**_pRLuc-MCS-F 117
MEP-044-pcDNA3.1-**BVDV-XH32-E2 (F126L)**_BGH-r 119
MEP-045-pcDNA3.1-**BVDV-XH32-E2 (F126Y)**_pRLuc-MCS-F 121
MEP-045-pcDNA3.1-**BVDV-XH32-E2 (F126Y)**_BGH-r 123
MEP-046-pcDNA3.1-**BVDV-XH32-E2 (F126P)**_pRLuc-MCS-F 125
MEP-046-pcDNA3.1-**BVDV-XH32-E2 (F126P)**_BGH-r 127
MEP-047-pcDNA3.1-**BVDV-XH32-E2 (F126W)**_pRLuc-MCS-F 129
MEP-047-pcDNA3.1-**BVDV-XH32-E2 (F126W)**_BGH-r 131

MEP-017-pcDNA3.1-BVDV-XH32-E2 (R72G)_pRLuc-MCS-F_2024-01-19_A07.ab1 (1000 bases)

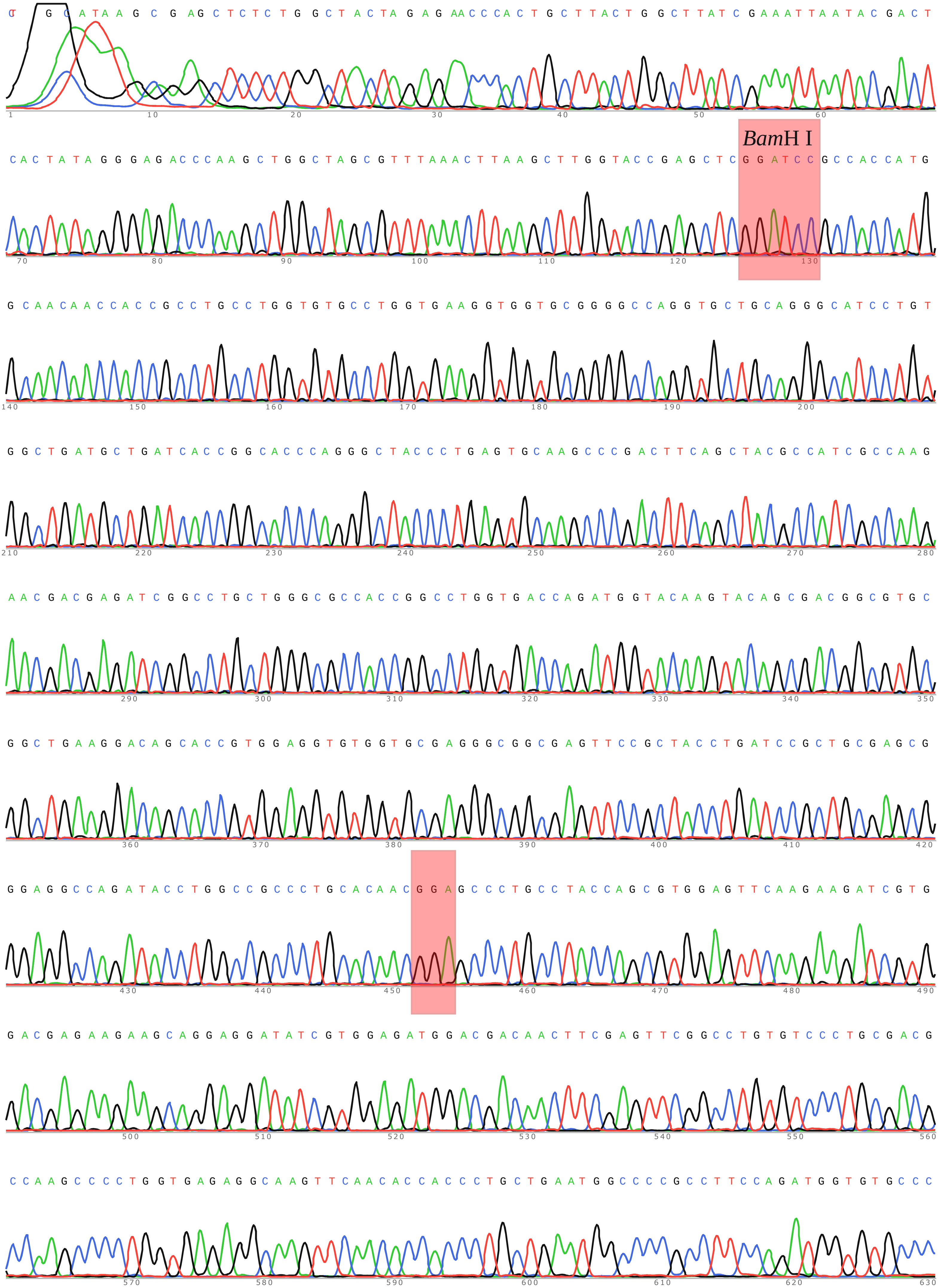

C A T C G G C T G G A C C G G C A C C G T G A G C T G C A C C C T G G C C A A C G A G G A T A C C C T G G C C A C C A C C G T G G T G C G C

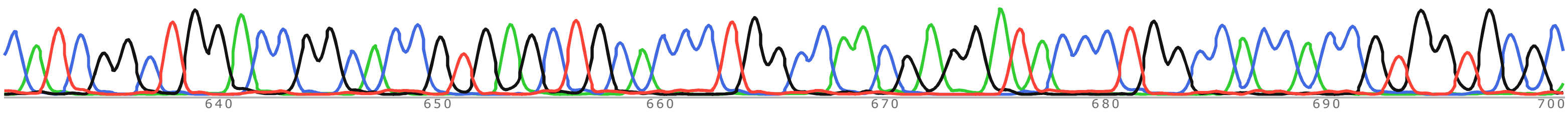

A C C T A C A A G A G G G T G A G A C C C T T C C C C T A C C G G C A G G A C T G C G T G A C C C A G A A A A C C A T C G G C G A G G A C C

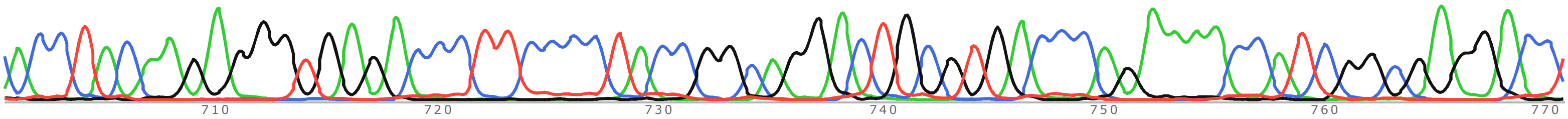

T G T A C G A T T G C G C C C T G G G C G G C A A C T G G A C C T G C G T G C T G G G C G A C A T C C T G A G G T A C G T G G C C G G C C C

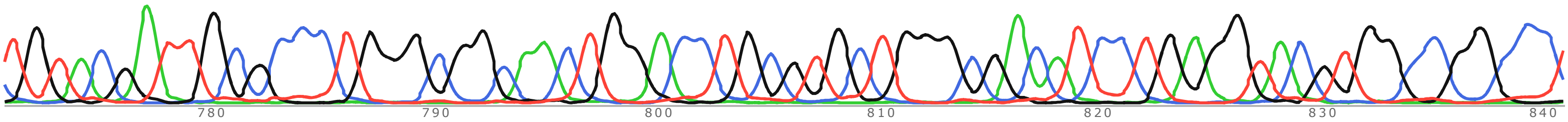

T G T G G A G T C C T G C A A G T G G T G C G G C C A C A A G T T C C T G A A G T C C G A G G G C C T G C C C C A C T T C C C C A T C G G C A

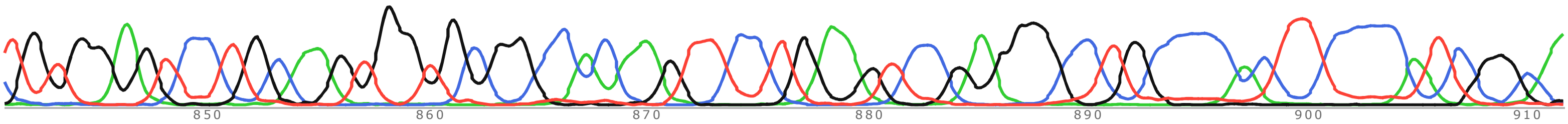

A G T G C C G C C T G A A G A A C G A G A G C G G C T A C A G A C T G G T G G A C G G C A C C T C C T G C A A C C G G G A C G G C G T G G C

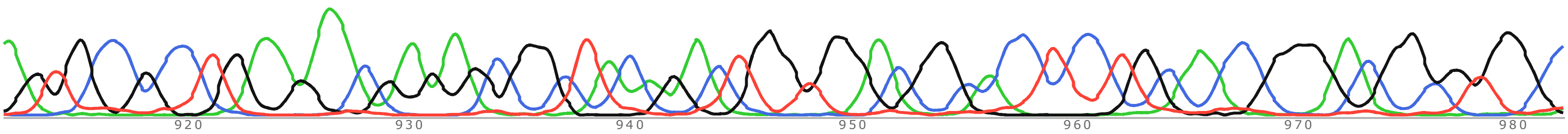

C A T C G T G C C C T C C G G C C G G

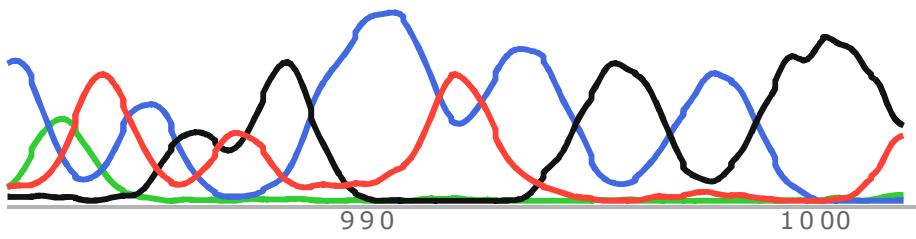

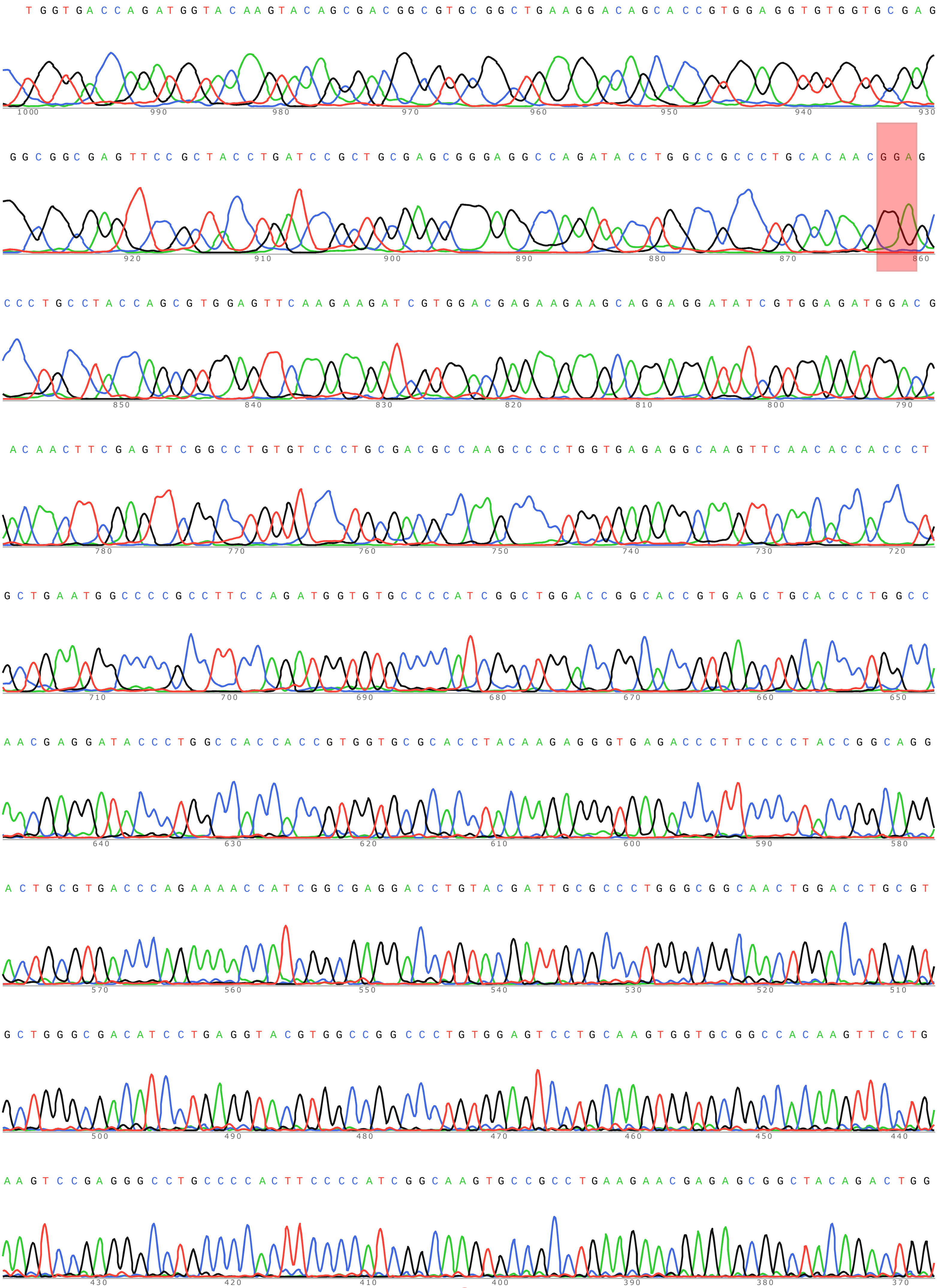
T G G T G A C C A G A T G G T A C A A G T A C A G C G A C G G C G T G C G G C T G A A G G A C A G C A C C G T G G A G G T G T G G T G C G A G
1000 990 980 970 960 950 940 930
G G C G G C G A G T T C C G C T A C C T G A T C C G C T G C G A G C G G G A G G C C A G A T A C C T G G C C G C C C T G C A C A A C G G A G
920 910 900 890 880 870 860
C C C T G C C T A C C A G C G T G G A G T T C A A G A A G A T C G T G G A C G A G A A G A A G C A G G A G G A T A T C G T G G A G A T G G A C G
850 840 830 820 810 800 790
A C A A C T T C G A G T T C G G C C T G T G T C C C T G C G A C G C C A A G C C C C T G G T G A G A G G C A A G T T C A A C A C C A C C C T
780 770 760 750 740 730 720
G C T G A A T G G C C C C G C C T T C C A G A T G G T G T G C C C C A T C G G C T G G A C C G G C A C C G T G A G C T G C A C C C T G G C C
710 700 690 680 670 660 650
A A C G A G G A T A C C C T G G C C A C C A C C G T G G T G C G C A C C T A C A A G A G G G T G A G A C C C T T C C C C T A C C G G C A G G
640 630 620 610 600 590 580
A C T G C G T G A C C C A G A A A A C C A T C G G C G A G G A C C T G T A C G A T T G C G C C C T G G G C G G C A A C T G G A C C T G C G T
570 560 550 540 530 520 510
G C T G G G C G A C A T C C T G A G G T A C G T G G C C G G C C C T G T G G A G T C C T G C A A G T G G T G C G G C C A C A A G T T C C T G
500 490 480 470 460 450 440
A A G T C C G A G G G C C T G C C C C A C T T C C C C A T C G G C A A G T G C C G C C T G A A G A A C G A G A G C G G C T A C A G A C T G G
430 420 410 400 390 380 370

MEP-017-pcDNA3.1-BVDV-XH32-E2 (R72G)_BGH-r_2024-01-19_A08.ab1 (1000 bases)

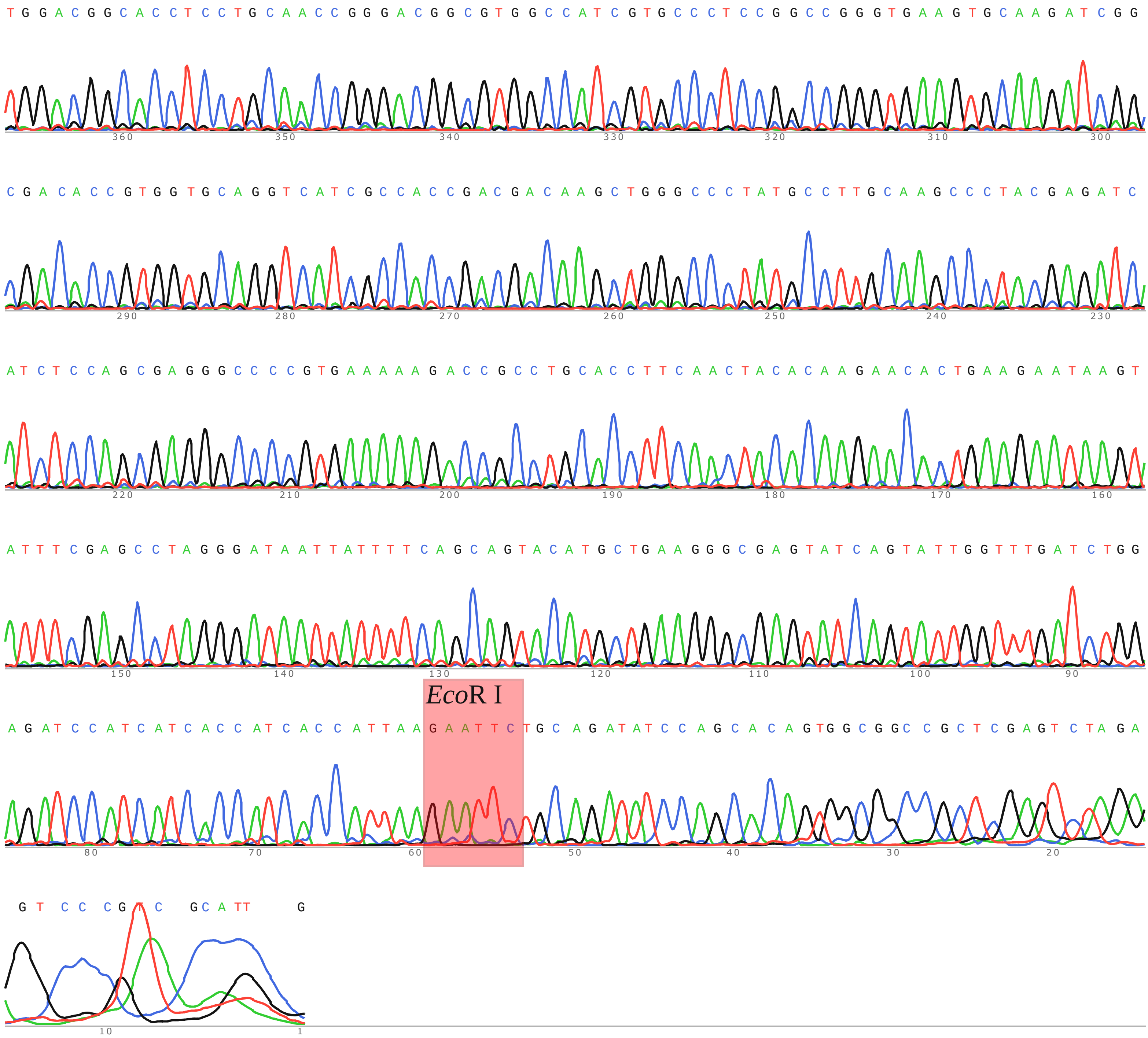

4

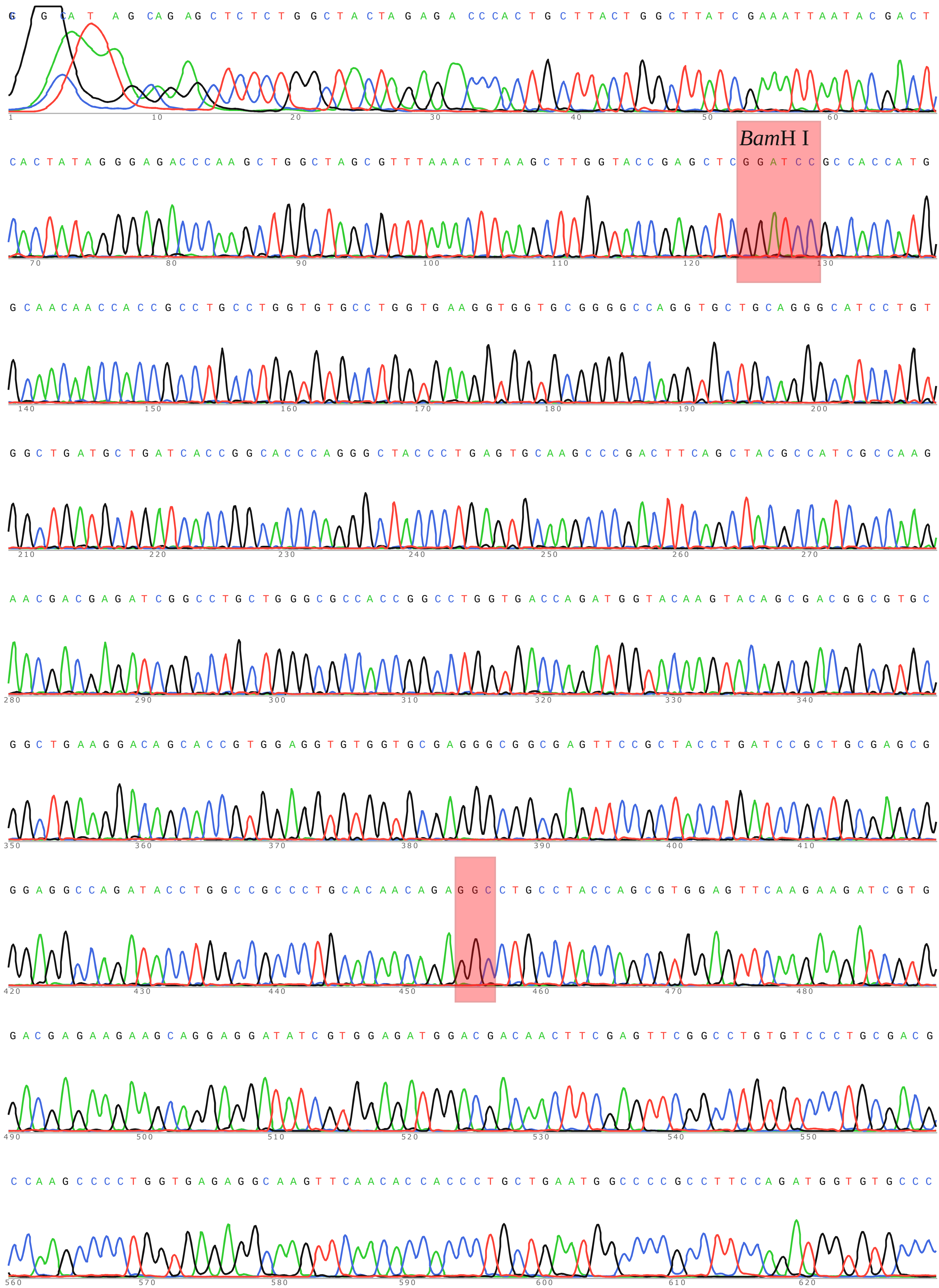
GGCATAGCAGAGCTCTCTGGCTACTAGAGACCCACTGCTTACTGGCTTATCGAAATTAATACGACT
CACTATAGGGAGACCCAAGCTGGCTAGCGTTTAAACTTAAGCTTGGTACCGAGCTCGGATCCGCCACCATG
BamH I
GCAACAACCACCGCCTGCCTGGTGTGCCTGGTGAAGGTGGTGCGGGGCCAGGTGCTGCAGGGCATCCTGT
GGCTGATGCTGATCACCGGCACCCAGGGCTACCCTGAGTGCAAGCCCGACTTCAGCTACGCCATCGCCAAG
AACGACGAGATCGGCCTGCTGGGCGCCACCGGCCTGGTGACCAGATGGTACAAGTACAGCGACGGCGTGC
GGCTGAAGGACAGCACCGTGGAGGTGTGGTGCGAGGGCGGCGAGTTCCGCTACCTGATCCGCTGCGAGCG
GGAGGCCAGATACCTGGCCGCCCTGCACAACAGAGGCCTGCCTACCAGCGTGGAGTTCAAGAAGATCGTG
GACGAGAAGAAGCAGGAGGATATCGTGGAGATGGACGACAACTTCGAGTTCGGCCTGTGTCCCTGCGACG
CCAAGCCCCTGGTGAGAGGCAAGTTCAACACCACCCTGCTGAATGGCCCCGCCTTCCAGATGGTGTGCCC

CATCGGCTGGACCGGCACCGTGAGCTGCACCCTGGCCAACGAGGATACCCTGGCCACCACCGTGGTGCGC

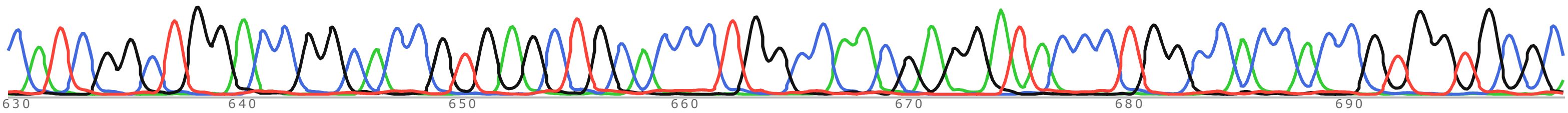

ACCTACAAGAGGGTGAGACCCTTCCCCTACCGGCAGGACTGCGTGACCCAGAAAACCATCGGCGAGGACC

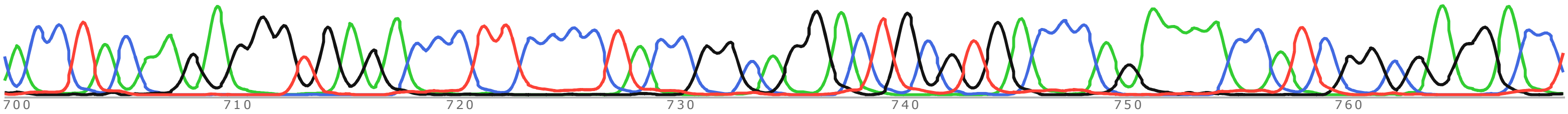

TGTACGATTGCGCCCTGGGCGGCAACTGGACCTGCGTGCTGGGCGACATCCTGAGGTACGTGGCCGGCCC

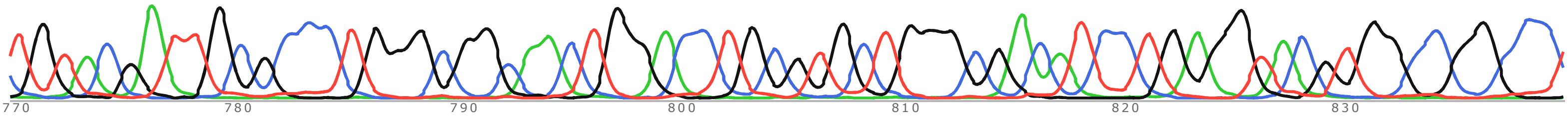

TGTGGAGTCCTGCAAGTGGTGCGG CCAC AAGTTCC TGAAGTCCGAGGGCCTGCCCCACTTCCCCATCGGCA

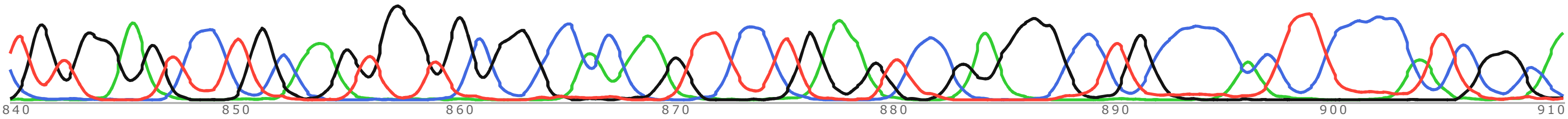

AGTGCC GCCTGAA GAACGAGAGCGG CTACAGACTGG TGG AC GGCACCTCCTGCAACCGGGAC GGCGTGG C

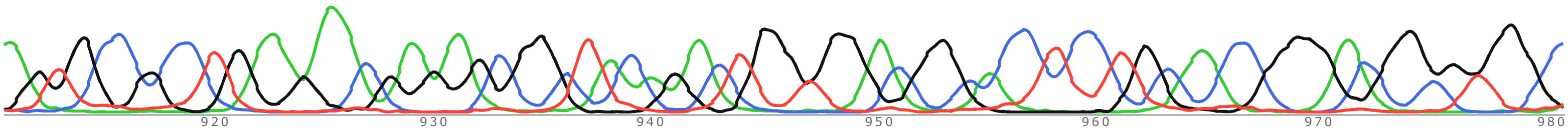

CATCGTG CCCTCC GG CC GGG

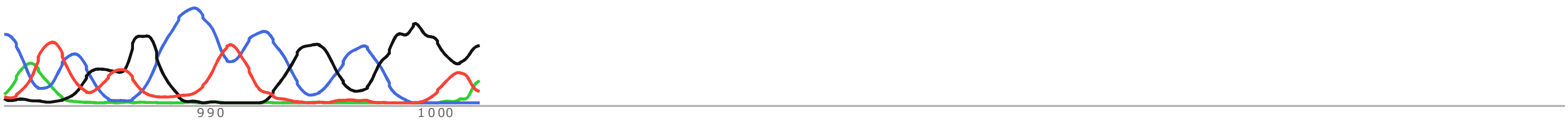

MEP-018-pcDNA3.1-BVDV-XH32-E2 (A73G)_BGH-r_2024-01-19_C08.ab1 (1000 bases)

GGTGACCAGATGGTACAAGTACAGCGACGGCGTGCGGCTGAAGGACAGCACCGTGGAGGTGTGGTGCGAGGGC

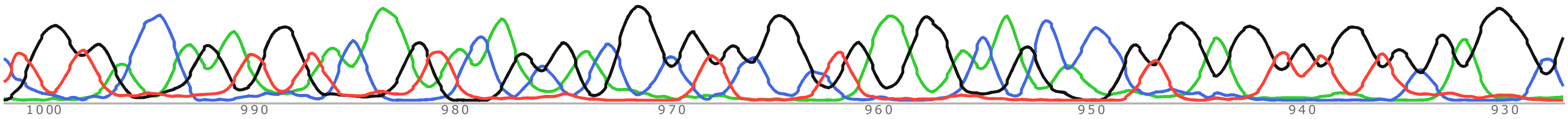

GGCGAGTTCCGCTACCTGATCCGCTGCGAGCGGGAGGCCAGATACCTGGCCGCCCTGCACAACAGAGGCCTGCC

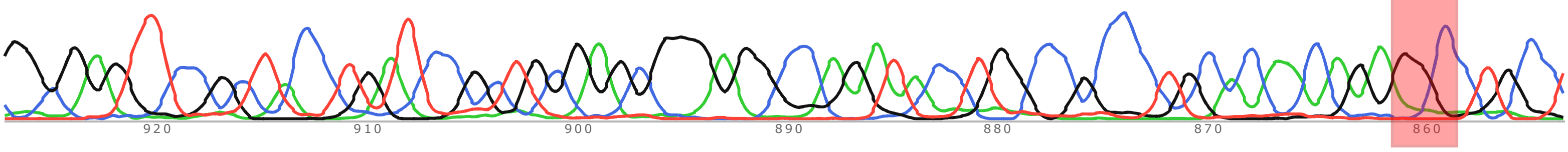

TACCAGCGTGGAGTTCAAGAAGATCGTGGACGAGAAGAAGCAGGAGGATATCGTGGAGATGGACGACAACTTCGA

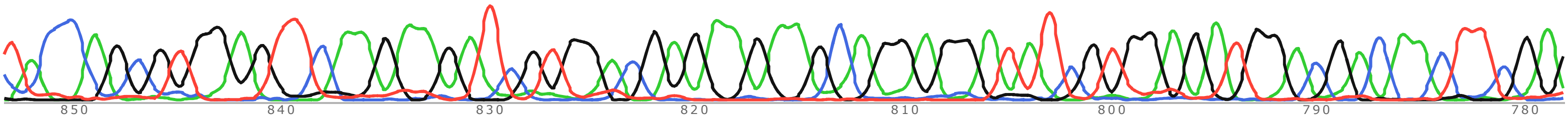

GTTCGGCCTGTGTCCCTGCGACGCCAAGCCCCTGGTGAGAGGCAAGTTCAACACCACCCTGCTGAATGGCCCCG

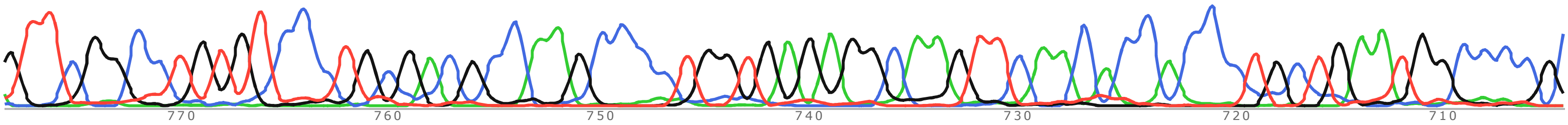

CCTTCCAGATGGTGTGCCCCATCGGCTGGACCGGCACCGTGAGCTGCACCCTGGCCAACGAGGATACCCTGGCC

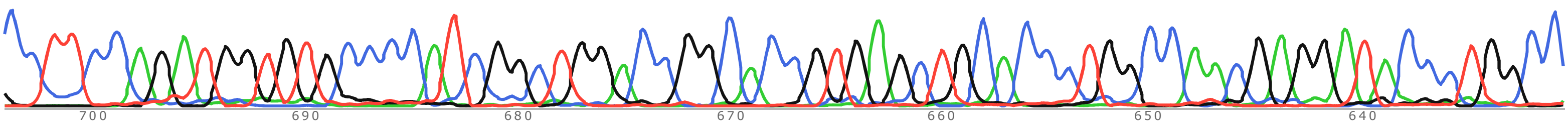

ACCACCGTGGTGCGCACCTACAAGAGGGTGAGACCCTTCCCCTACCGGCAGGACTGCGTGACCCAGAAAACCA

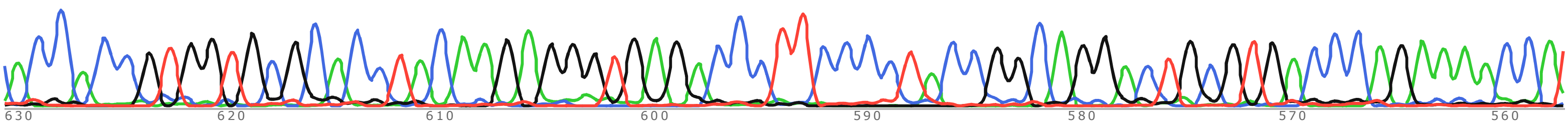

TCGGCGAGGACCTGTACGATTGCGCCCTGGGCGGCAACTGGACCTGCGTGCTGGGCGACATCCTGAGGTACGTG

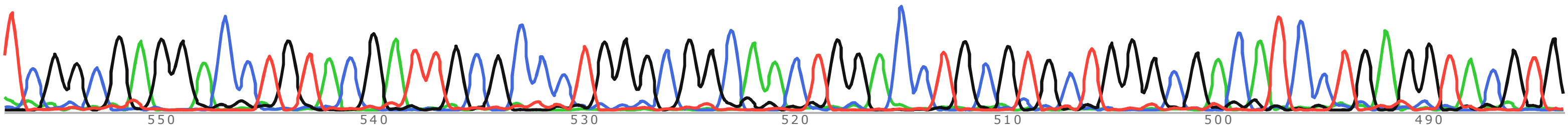

GCCGGCCCTGTGGAGTCCTGCAAGTGGTGCGGCCACAAGTTCCTGAAGTCCGAGGGCCTGCCCCACTTCCCCAT

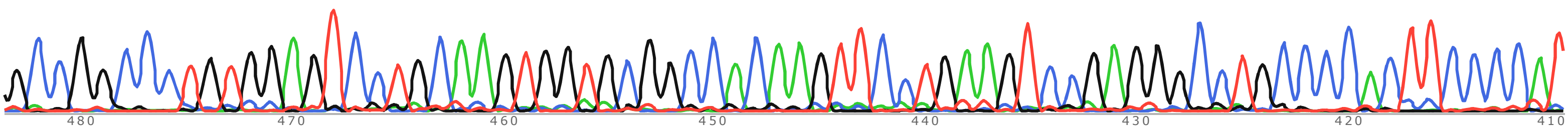

CGGCAAGTGCCGCCTGAAGAACGAGAGCGGCTACAGACTGGTGGACGGCACCTCCTGCAACCGGGACGGCGTGG

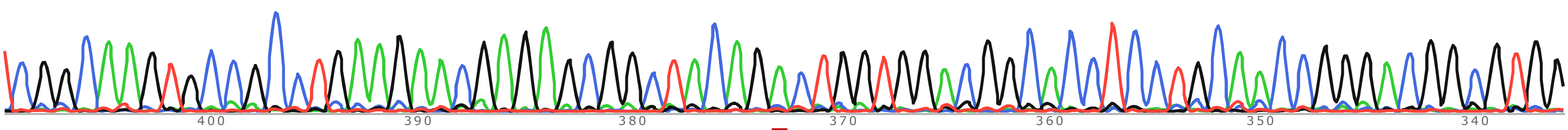

7

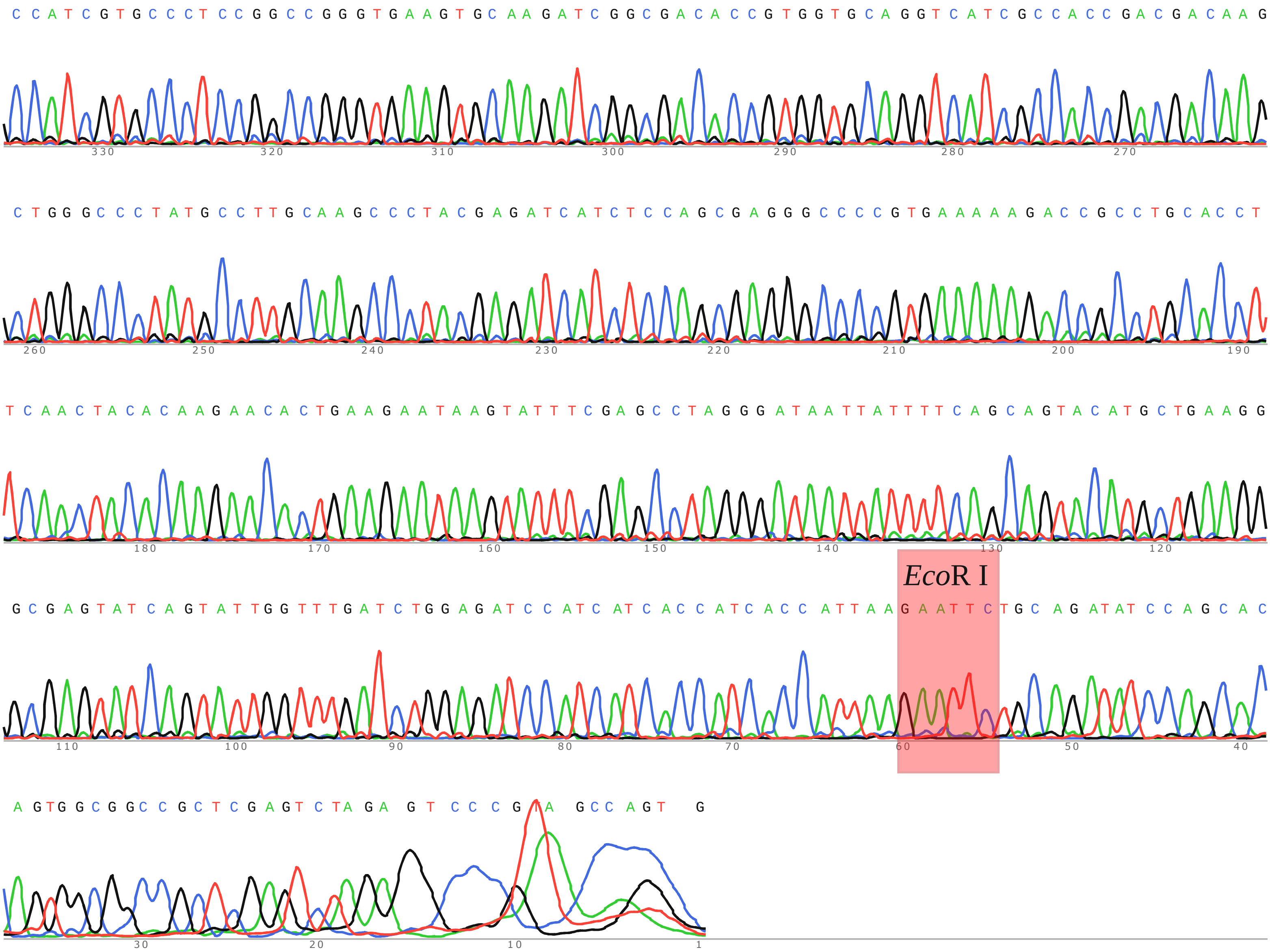
CCATCGTGCCCTCCGGCCGGGTGAAGTGCAAGATCGGCGACACCGTGGTGCAGGTCATCGCCACCGACAAG
CTGGGCCCTATGCCTTGCAAGCCCTACGAGATCATCTCCAGCGAGGGCCCCGTGAAAAAGACCGCCTGCACCT
TCAACTACACAAGAACACTGAAGAATAAGTATTTCGAGCCTAGGGATAATTATTTTCAGCAGTACATGCTGAAGG
EcoR I
GCGAGTATCAGTATTGGTTTGATCTGGAGATCCATCATCACCATCACCATTAAGAATTCTGCAGATATCCAGCAC
AGTGGCGGCCGCTCGAGTCTAGAGTCCCGTAGCCAGTG

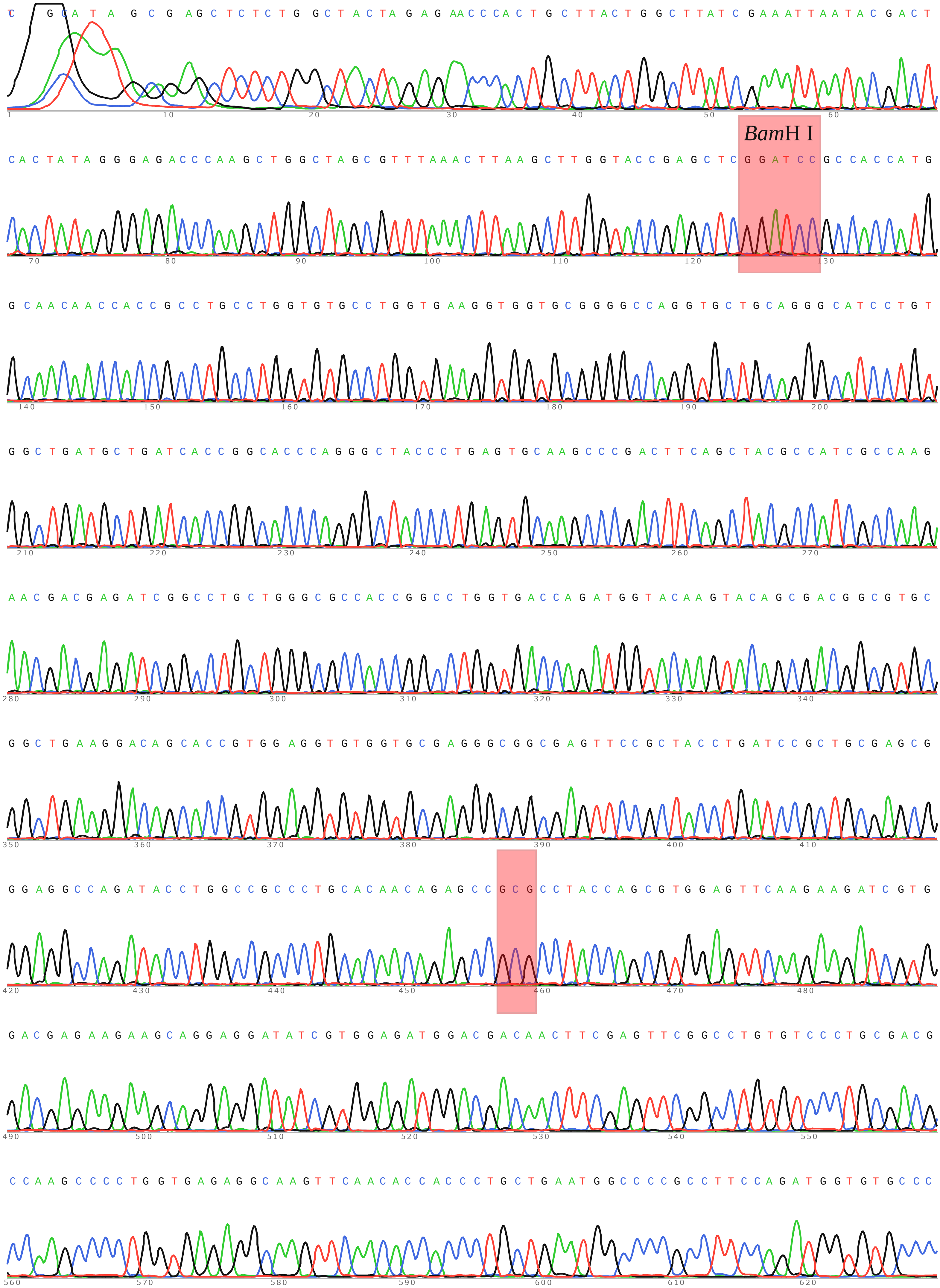
TGCATAGCGAGCTCTCTGGCTACTAGAGAACCCACTGCTTACTGGCTTATCGAAATTAATACGACT
BamH I
CACTATAGGGAGACCCAAGCTGGCTAGCGTTTAAACTTAAGCTTGGTACCGAGCTCGGATCCGCCACCATG
GCAACAACCACCGCCTGCCTGGTGTGCCTGGTGAAGGTGGTGCGGGGCCAGGTGCTGCAGGGCATCCTGT
GGCTGATGCTGATCACCGGCACCCAGGGCTACCCTGAGTGCAAGCCCGACTTCAGCTACGCCATCGCCAAG
AACGACGAGATCGGCCTGCTGGGCGCCACCGGCCTGGTGACCAGATGGTACAAGTACAGCGACGGCGTGC
GGCTGAAGGACAGCACCGTGGAGGTGTGGTGCGAGGGCGGCGAGTTCCGCTACCTGATCCGCTGCGAGCG
GGAGGCCAGATACCTGGCCGCCCTGCACAACAGAGCCGCGCCTACCAGCGTGGAGTTCAAGAAGATCGTG
GACGAGAAGAAGCAGGAGGATATCGTGGAGATGGACGACAACTTCGAGTTCGGCCTGTGTCCCTGCGACG
CCAAGCCCCTGGTGAGAGGCAAGTTCAACACCACCCTGCTGAATGGCCCCGCCTTCCAGATGGTGTGCCC

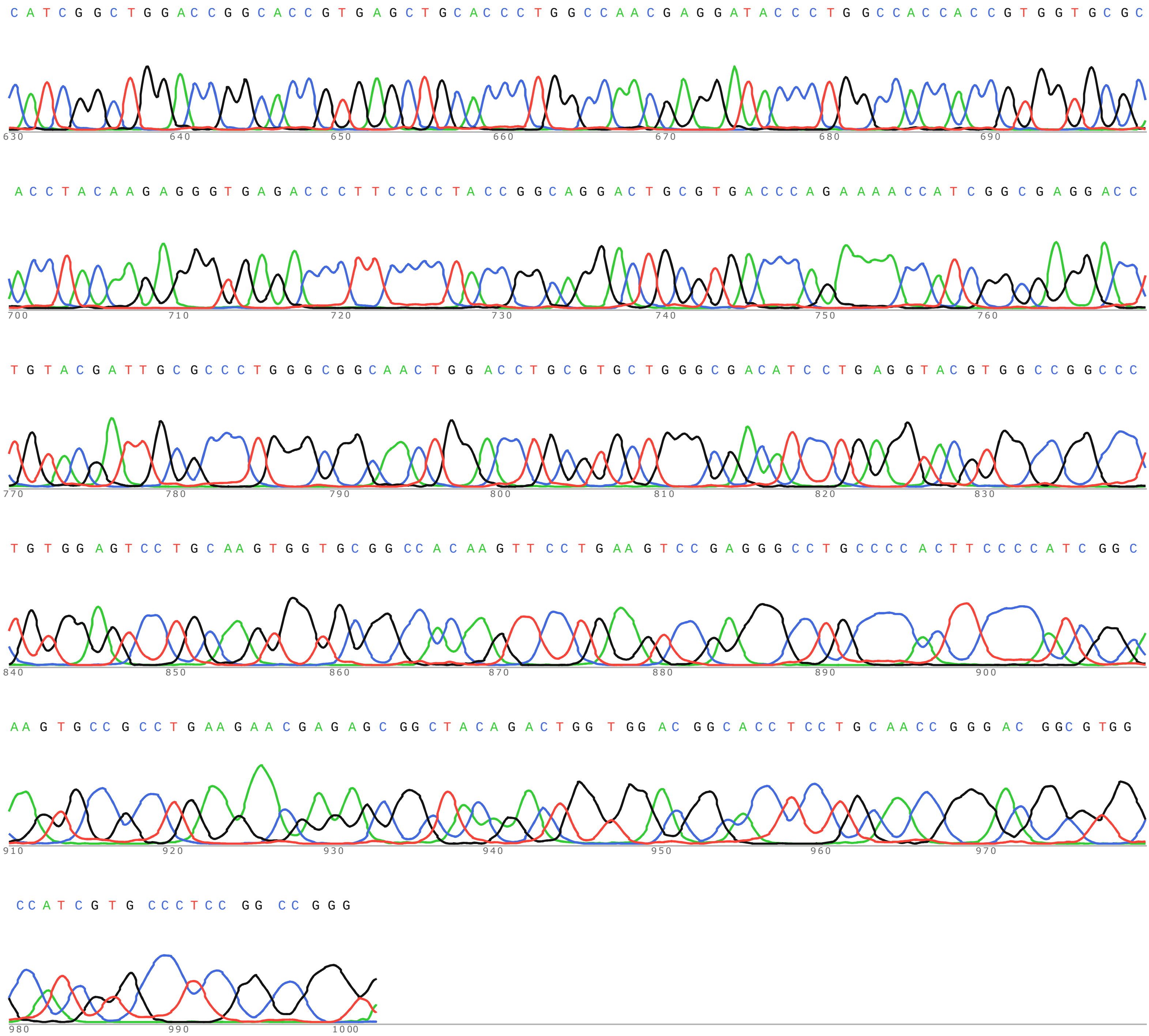

C A T C G G C T G G A C C G G C A C C G T G A G C T G C A C C C T G G C C A A C G A G G A T A C C C T G G C C A C C A C C G T G G T G C G C
630 640 650 660 670 680 690
A C C T A C A A G A G G G T G A G A C C C T T C C C C T A C C G G C A G G A C T G C G T G A C C C A G A A A A C C A T C G G C G A G G A C C
700 710 720 730 740 750 760
T G T A C G A T T G C G C C C T G G G C G G C A A C T G G A C C T G C G T G C T G G G C G A C A T C C T G A G G T A C G T G G C C G G C C C
770 780 790 800 810 820 830
T G T G G A G T C C T G C A A G T G G T G C G G C C A C A A G T T C C T G A A G T C C G A G G G C C T G C C C C A C T T C C C C A T C G G C
840 850 860 870 880 890 900
A A G T G C C G C C T G A A G A A C G A G A G C G G C T A C A G A C T G G T G G A C G G C A C C T C C T G C A A C C G G G A C G G C G T G G
910 920 930 940 950 960 970
C C A T C G T G C C C T C C G G C C G G G
980 990 1000

GGTGACCAGATGGTACAAGTACAGCGACGGCGTGCGGCTGAAGGACAGCACCGTGGAGGTGTGGTGCGAG

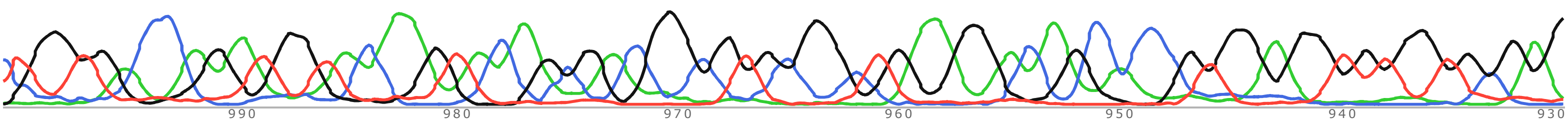

GGCGGCGAGTTCCGCTACCTGATCCGCTGCGAGCGGGAGGCCAGATACCTGGCCGCCCTGCACAACAGAG

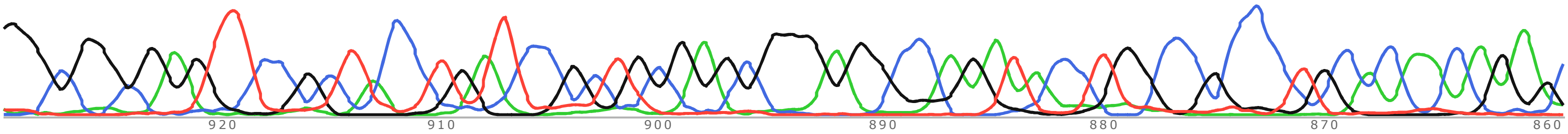

CCGCGCCTACCAGCGTGGAGTTCAAGAAGATCGTGGACGAGAAGCAGGAGGATATCGTGGAGATGGACG

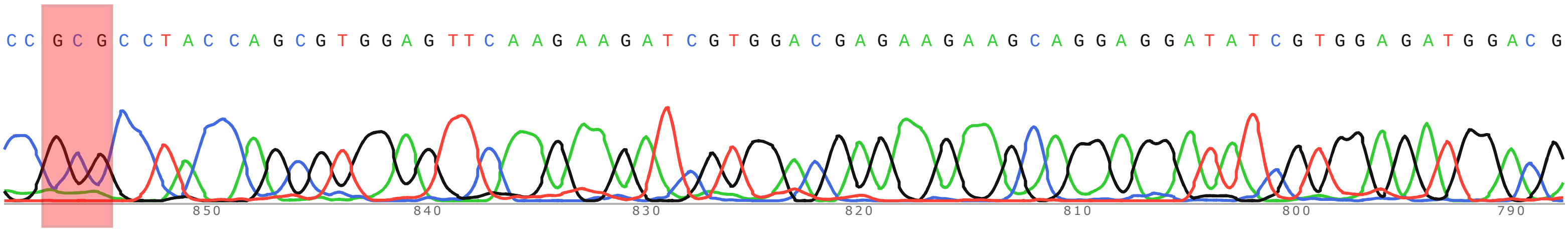

ACAACTTCGAGTTCGGCCTGTGTCCCTGCGACGCCAAGCCCCTGGTGAGAGGCAAGTTCAACACCACCCTG

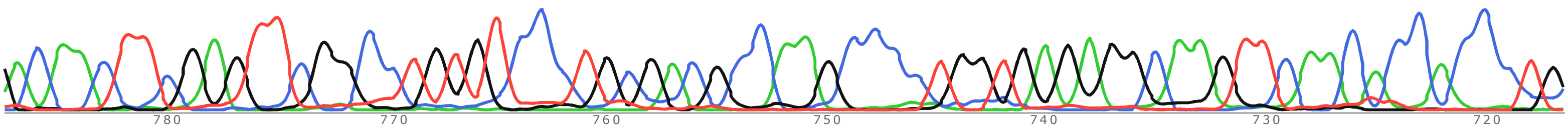

CTGAATGGCCCCGCCTTCCAGATGGTGTGCCCCATCGGCTGGACCGGCACCGTGAGCTGCACCCTGGCCAA

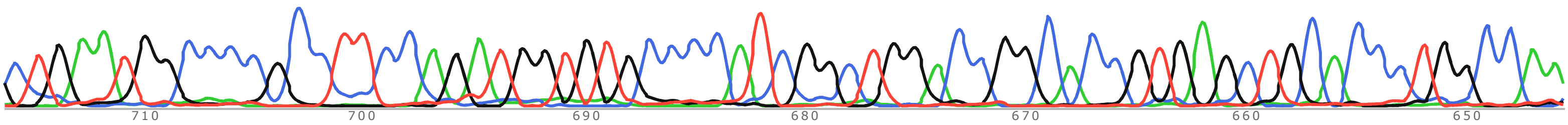

CGAGGATACCCTGGCCACCACCGTGGTGCGCACCTACAAGAGGGTGAGACCCTTCCCCTACCGGCAGGAC

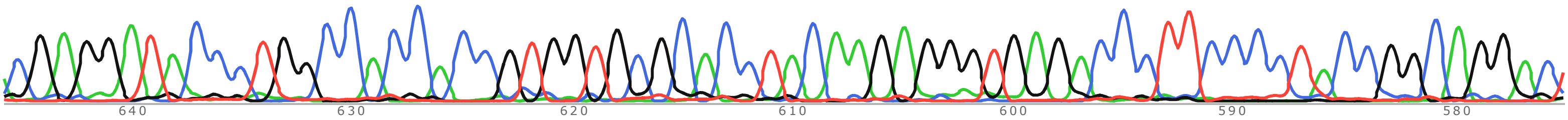

TGCGTGACCCAGAAAACCATCGGCGAGGACCTGTACGATTGCGCCCTGGGCGGCAACTGGACCTGCGTGCT

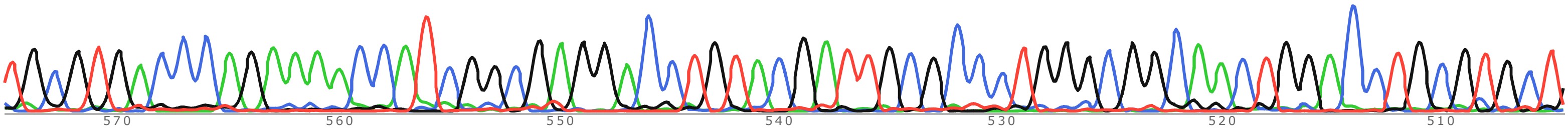

GGGCGACATCCTGAGGTACGTGGCCGGCCCTGTGGAGTCCTGCAAGTGGTGCGGCCACAAGTTCCTGAAGT

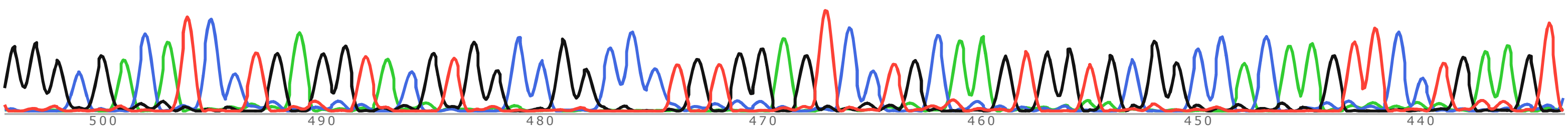

CCGAGGGCCTGCCCCACTTCCCCATCGGCAAGTGCCGCCTGAAGAACGAGAGCGGCTACAGACTGGTGGAC

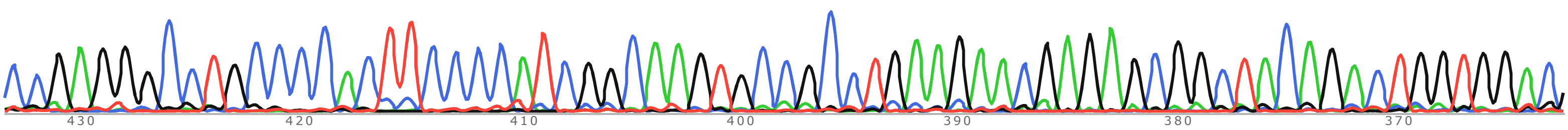

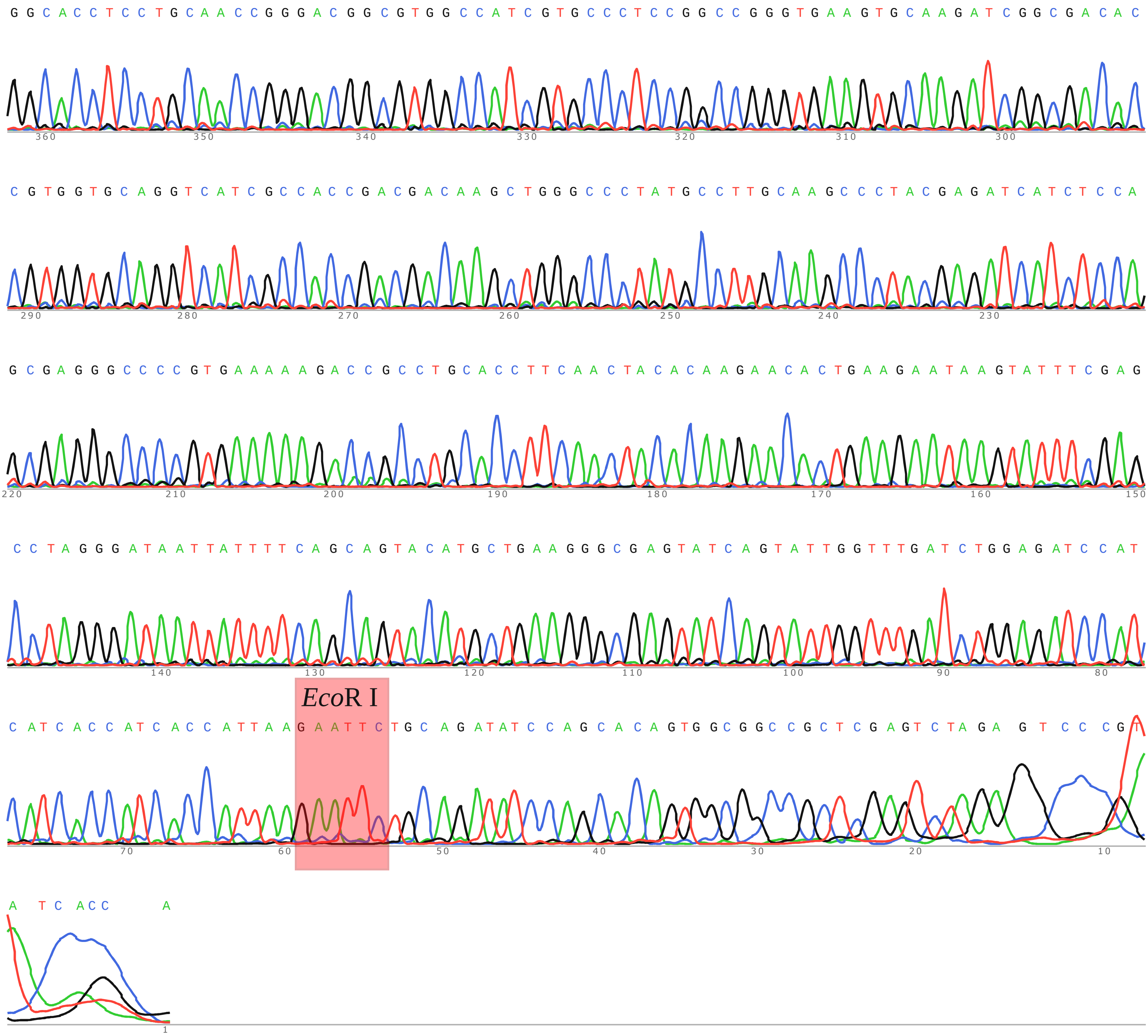
GGCACCTCCTGCAACCGGGACGGCGTGGCCATCGTGCCCTCCGGCCGGGTGAAGTGCAAGATCGGCGACAC
360 350 340 330 320 310 300
CGTGGTGCAGGTCATCGCCACCGACGACAAGCTGGGCCCTATGCCTTGCAAGCCCTACGAGATCATCTCCA
290 280 270 260 250 240 230
GCGAGGGCCCCGTGAAAAAGACCGCCTGCACCTTCAACTACACAAGAACACTGAAGAATAAGTATTTCGAG
220 210 200 190 180 170 160 150
CCTAGGGATAATTATTTTCAGCAGTACATGCTGAAGGGCGAGTATCAGTATTGGTTTGATCTGGAGATCCAT
140 130 120 110 100 90 80
*Eco*R I
CATCACCATCACCATTAAGAATTCTGCAGATATCCAGCACAGTGGCGGCCGCTCGAGTCTAGA GTCC CGT
70 60 50 40 30 20 10
A TC ACC A
1

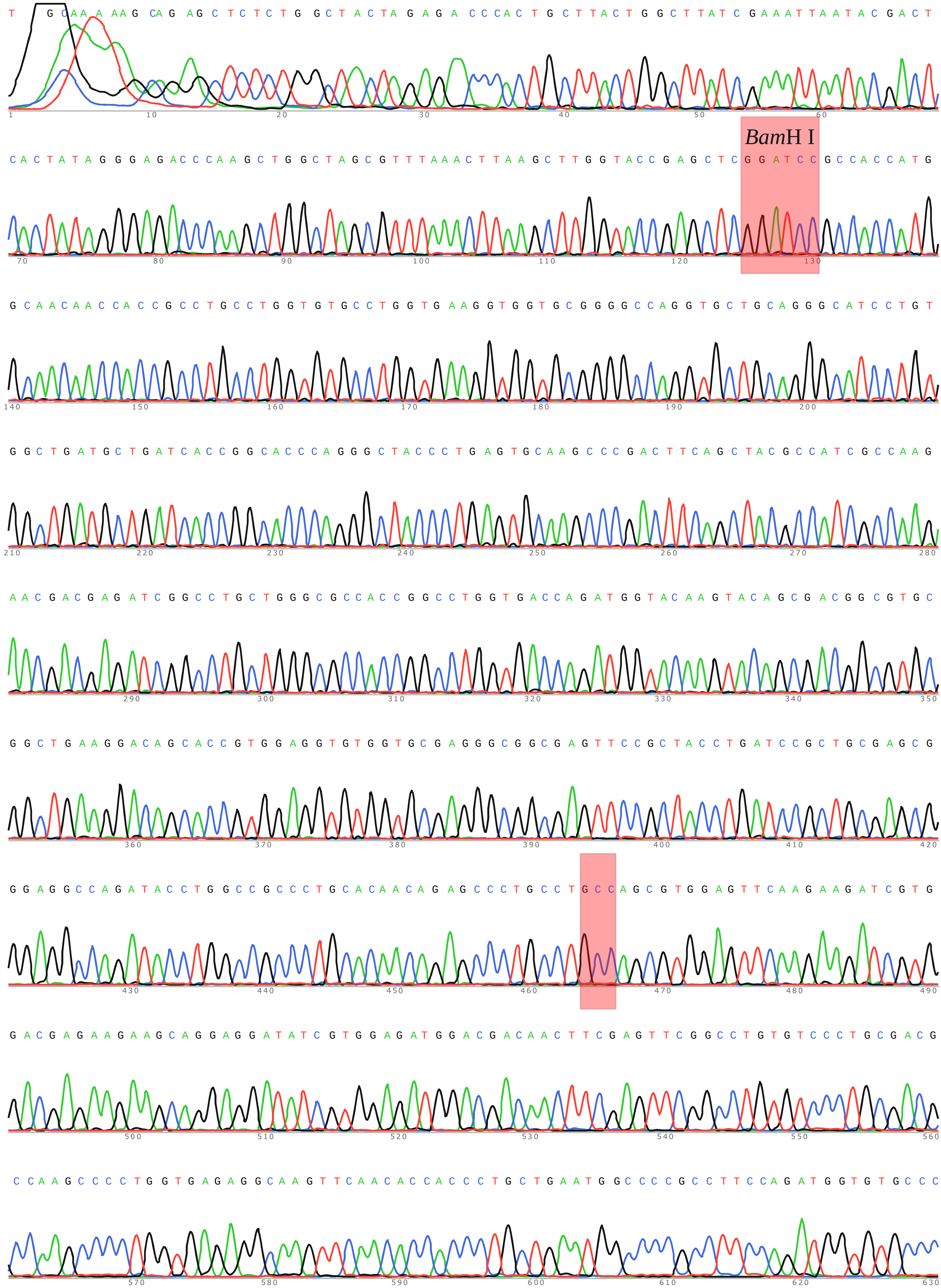
TGCAAAAAGCAGAGCTCTCTGGCTACTAGAGACCCACTGCTTACTGGCTTATCGAAATTAATACGACT
1 10 20 30 40 50 60
BamH I
CACTATAGGGAGACCCAAGCTGGCTAGCGTTTAAACTTAAGCTTGGTACCGAGCTCGGATCCGCCACCATG
70 80 90 100 110 120 130
GCAACAACCACCGCCTGCCTGGTGTGCCTGGTGAAGGTGGTGCGGGGCCAGGTGCTGCAGGGCATCCTGT
140 150 160 170 180 190 200
GGCTGATGCTGATCACCGGCACCCAGGGCTACCCTGAGTGCAAGCCCGACTTCAGCTACGCCATCGCCAAG
210 220 230 240 250 260 270 280
AACGACGAGATCGGCCTGCTGGGCGCCACCGGCCTGGTGACCAGATGGTACAAGTACAGCGACGGCGTGC
290 300 310 320 330 340 350
GGCTGAAGGACAGCACCGTGGAGGTGTGGTGCGAGGGCGGCGAGTTCCGCTACCTGATCCGCTGCGAGCG
360 370 380 390 400 410 420
GGAGGCCAGATACCTGGCCGCCCTGCACAACAGAGCCCTGCCTGCCAGCGTGGAGTTCAAGAAGATCGTG
430 440 450 460 470 480 490
GACGAGAAGAAGCAGGAGGATATCGTGGAGATGGACGACAACTTCGAGTTCGGCCTGTGTCCCTGCGACG
500 510 520 530 540 550 560
CCAAGCCCCTGGTGAGAGGCAAGTTCAACACCACCCTGCTGAATGGCCCCGCCTTCCAGATGGTGTGCCC
570 580 590 600 610 620 630

CATCGGCTGGACCGGCACCGTGAGCTGCACCCTGGCCAACGAGGATACCCTGGCCACCACCGTGGTGCG

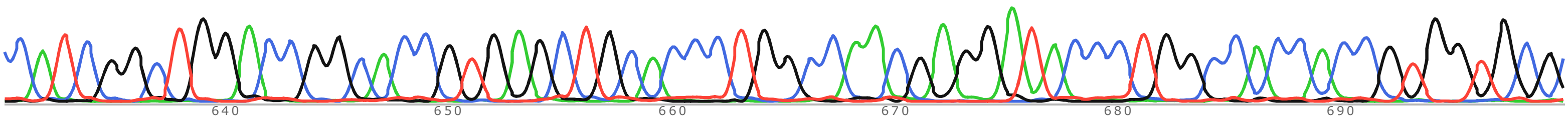

CACCTACAAGAGGGTGAGACCCTTCCCCTACCGGCAGGACTGCGTGACCCAGAAAACCATCGGCGAGGAC

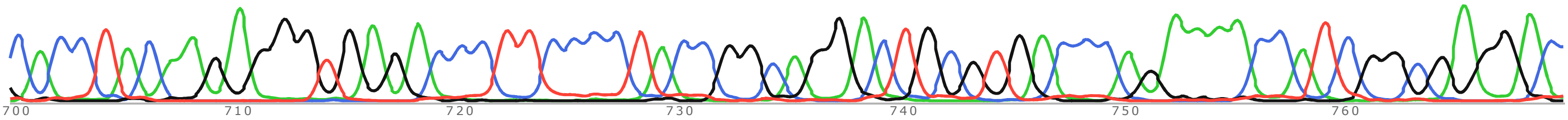

CTGTACGATTGCGCCCTGGGCGGCAACTGGACCTGCGTGCTGGGCGACATCCTGAGGTACGTGGCCGGCC

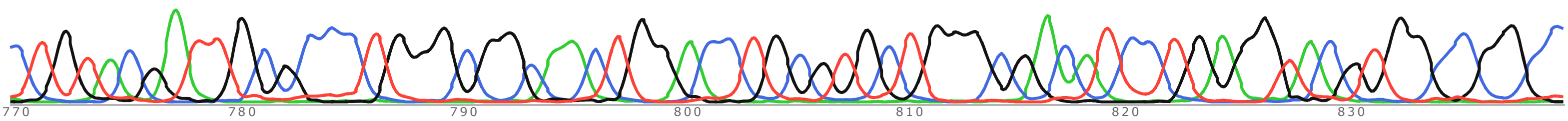

CTGTGGAGTCCTGCAAGTGGTGCGGCCACAAGTTCCTGAAGTCCGAGGGCCTGCCCCACTTCCCCATCGG

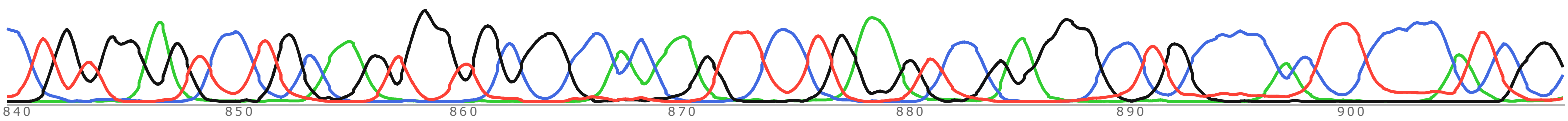

CAAGTGCCGCCTGAAGAACGAGAGCGGCTACAGACTGGTGGACGGCACCTCCTGCAACCGGGACGCGTG

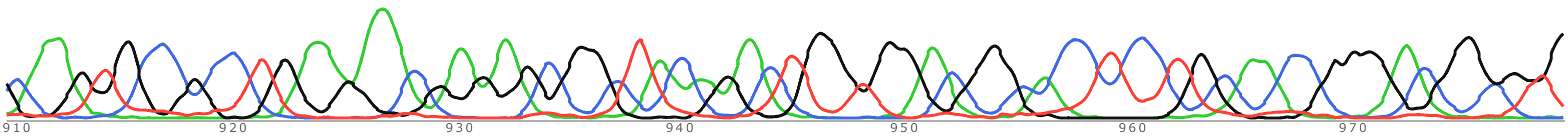

GCCATCGTGCCCTCCGGCCGGG

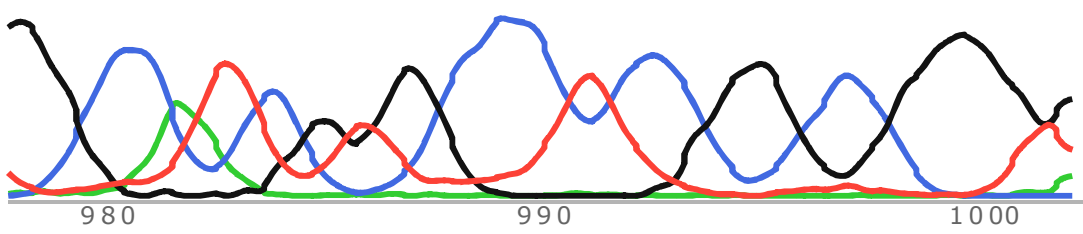

14

MEP-020-pcDNA3.1-BVDV-XH32-E2(T76A)_BGH-r_2024-01-19_G08.ab1 (1000 bases)

GGTGACCAGATGGTACAAGTACAGCGACGGCGTGCGGCTGAAGGACAGCACCGTGGAGGTGTGGTGCGAG

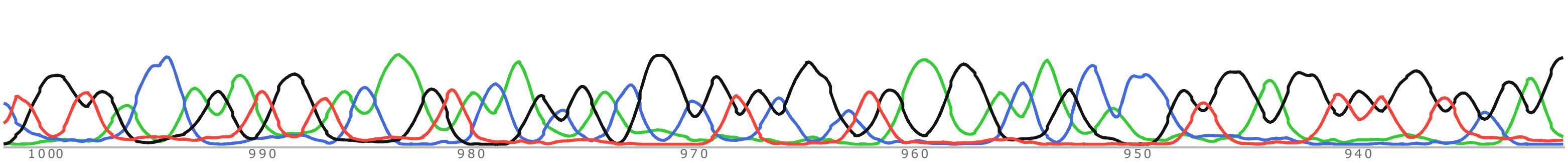

GGCGGCGAGTTCCGCTACCTGATCCGCTGCGAGCGGGAGGCCAGATACCTGGCCGCCCTGCACAACAGAG

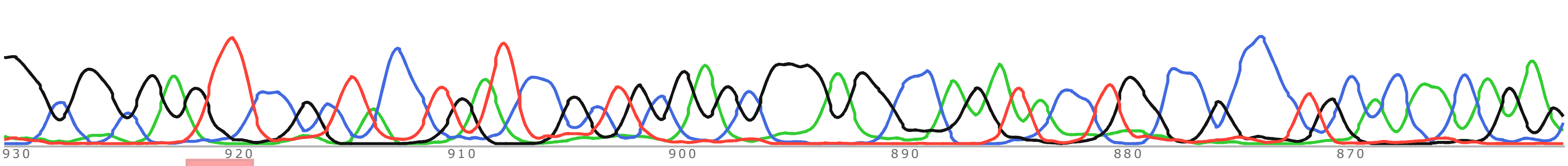

CCCTGCCTGCCAGCGTGGAGTTCAAGAAGATCGTGGACGAGAAGAAGCAGGAGGATATCGTGGAGATGGAC

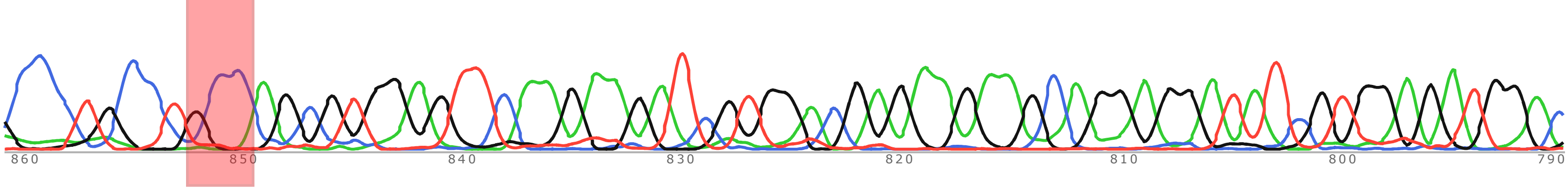

GACAACTTCGAGTTCGGCCTGTGTCCCTGCGACGCCAAGCCCCTGGTGAGAGGCAAGTTCAACACCACC

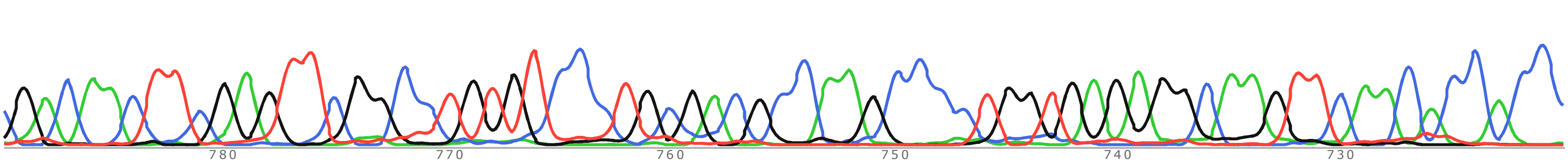

CTGCTGAATGGCCCCGCCTTCCAGATGGTGTGCCCCATCGGCTGGACCGGCACCGTGAGCTGCACCCTGG

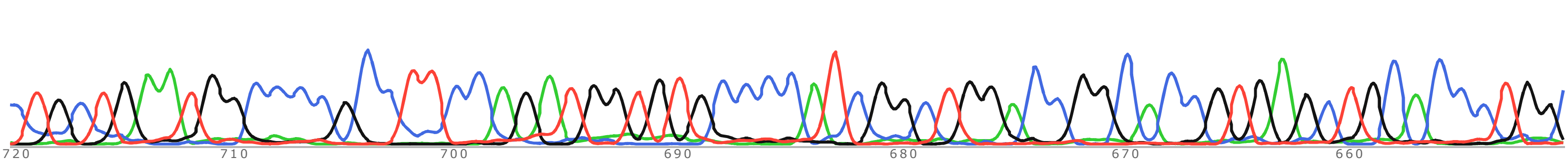

CCAACGAGGATACCCTGGCCACCACCGTGGTGCGCACCTACAAGAGGGTGAGACCCTTCCCCTACCGGCA

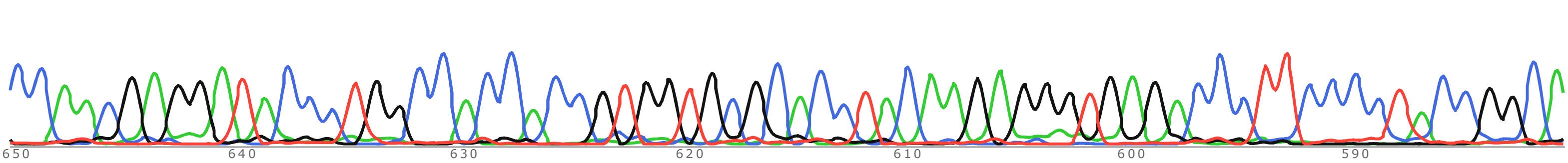

GGACTGCGTGACCCAGAAAACCATCGGCGAGGACCTGTACGATTGCGCCCTGGGCGGCAACTGGACCTGC

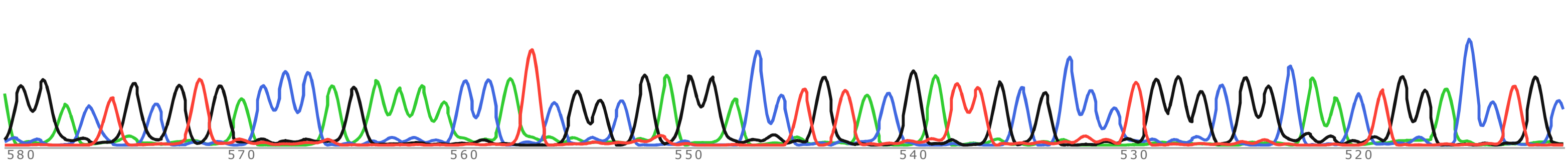

GTGCTGGGCGACATCCTGAGGTACGTGGCCGGCCCTGTGGAGTCCTGCAAGTGGTGCGGCCACAAGTTCC

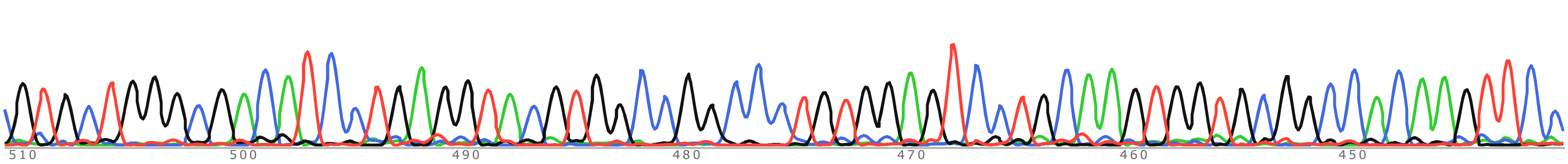

TGAAGTCCGAGGGCCTGCCCCACTTCCCCATCGGCAAGTGCCGCCTGAAGAACGAGAGCGGCTACAGACT

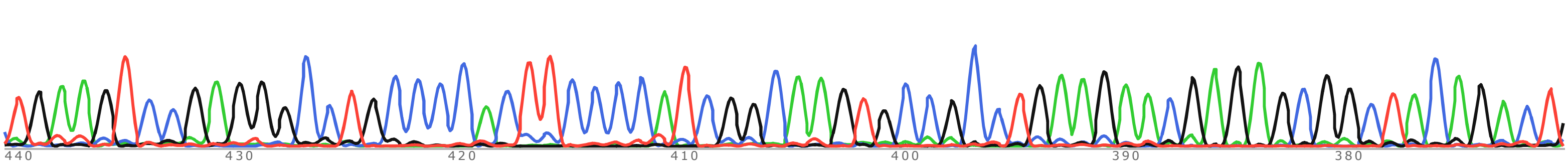

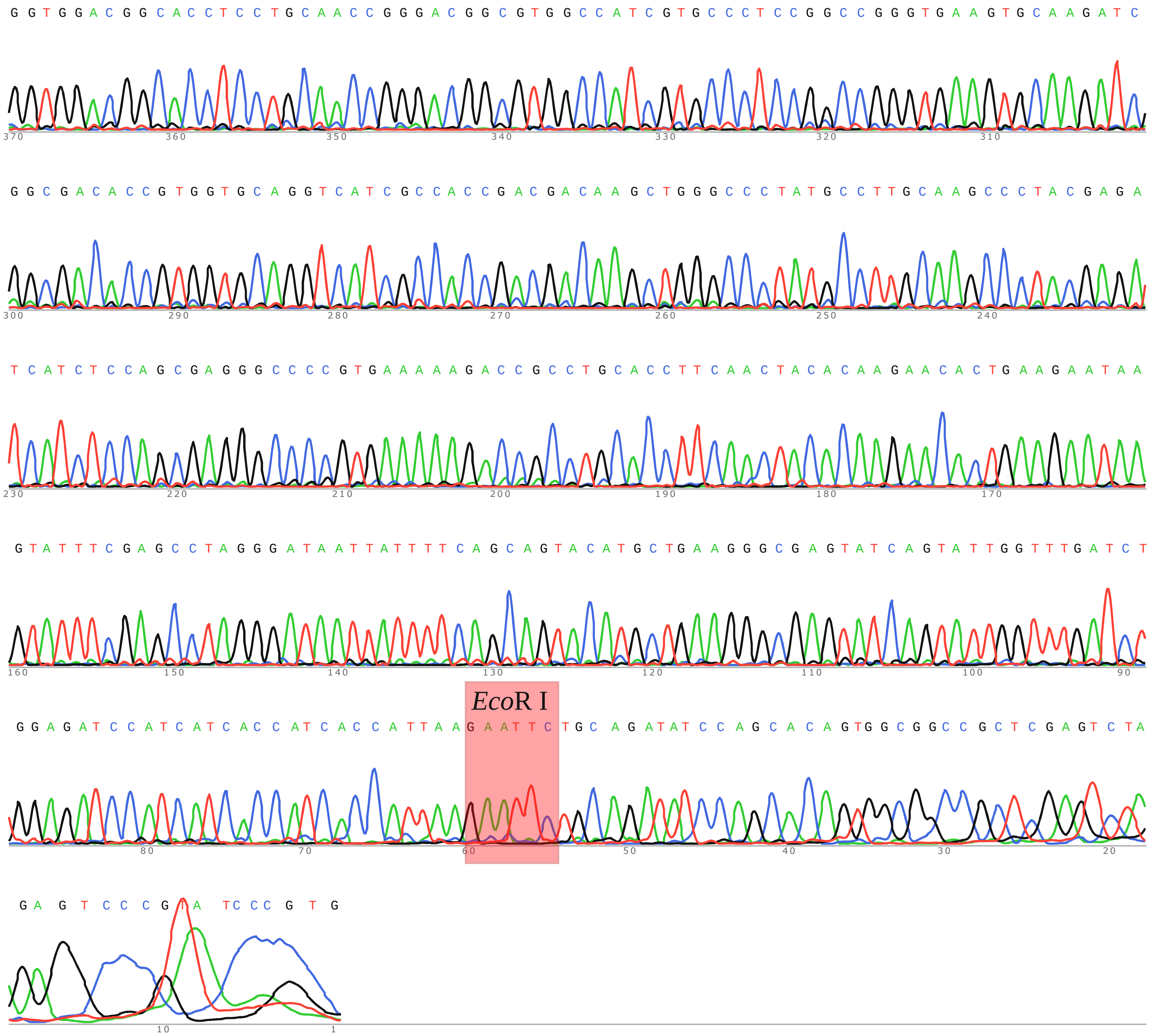
G G T G G A C G G C A C C T C C T G C A A C C G G G A C G G C G T G G C C A T C G T G C C C T C C G G C C G G G T G A A G T G C A A G A T C
370 360 350 340 330 320 310
G G C G A C A C C G T G G T G C A G G T C A T C G C C A C C G A C G A C A A G C T G G G C C C T A T G C C T T G C A A G C C C T A C G A G A
300 290 280 270 260 250 240
T C A T C T C C A G C G A G G G C C C C G T G A A A A A G A C C G C C T G C A C C T T C A A C T A C A C A A G A A C A C T G A A G A A T A A
230 220 210 200 190 180 170
G T A T T T C G A G C C T A G G G A T A A T T A T T T T C A G C A G T A C A T G C T G A A G G G C G A G T A T C A G T A T T G G T T T G A T C T
160 150 140 130 120 110 100 90
EcoR I
G G A G A T C C A T C A T C A C C A T C A C C A T T A A G A A T T C T G C A G A T A T C C A G C A C A G T G G C G G C C G C T C G A G T C T A
80 70 60 50 40 30 20
G A G T C C C G T A T C C C G T G
10 1

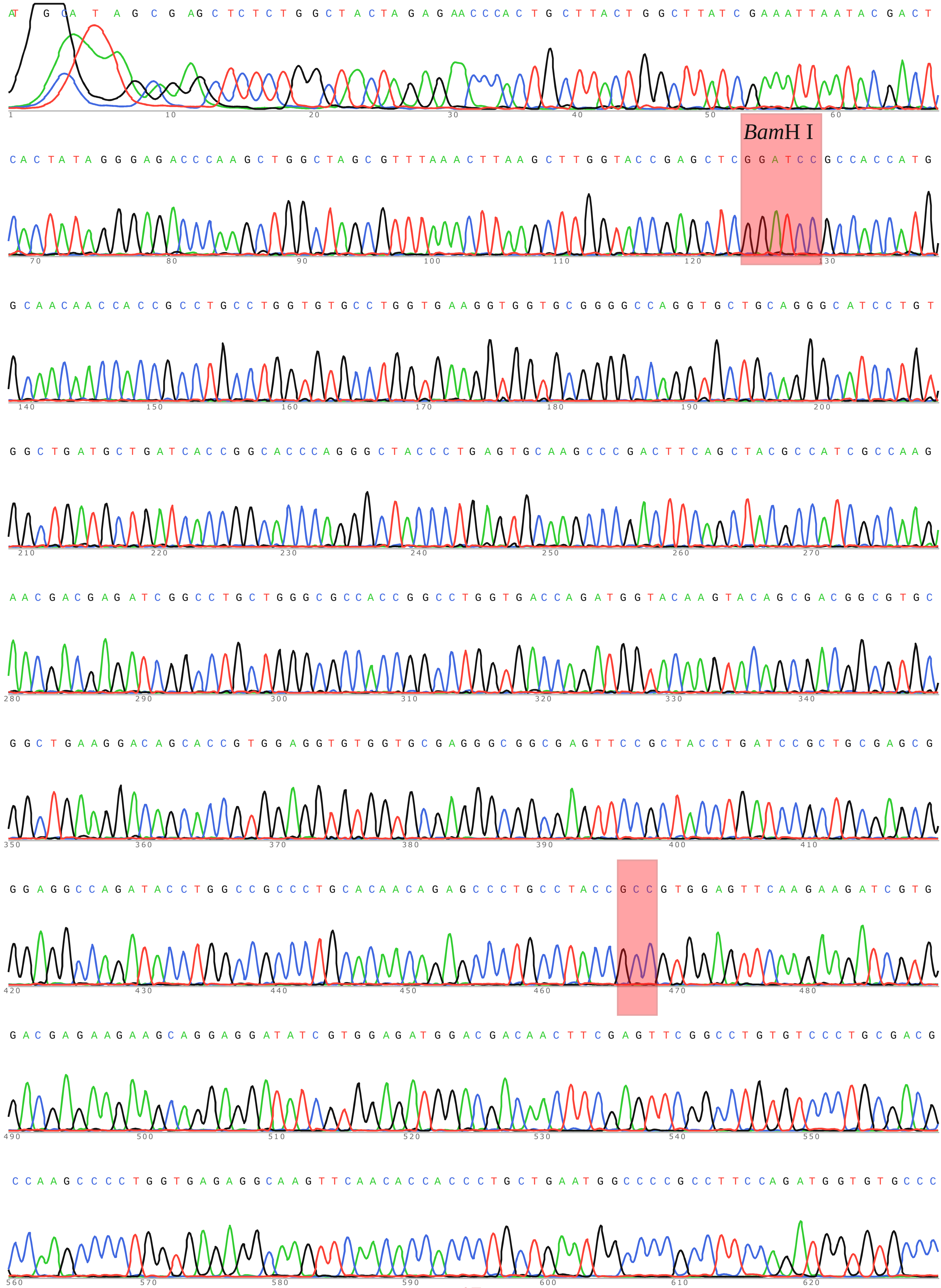
A T G C A T A G C G A G C T C T C T G G C T A C T A G A G A A C C C A C T G C T T A C T G G C T T A T C G A A A T T A A T A C G A C T
BamH I
C A C T A T A G G G A G A C C C A A G C T G G C T A G C G T T T A A A C T T A A G C T T G G T A C C G A G C T C G G A T C C G C C A C C A T G
G C A A C A A C C A C C G C C T G C C T G G T G T G C C T G G T G A A G G T G G T G C G G G G C C A G G T G C T G C A G G G C A T C C T G T
G G C T G A T G C T G A T C A C C G G C A C C C A G G G C T A C C C T G A G T G C A A G C C C G A C T T C A G C T A C G C C A T C G C C A A G
A A C G A C G A G A T C G G C C T G C T G G G C G C C A C C G G C C T G G T G A C C A G A T G G T A C A A G T A C A G C G A C G G C G T G C
G G C T G A A G G A C A G C A C C G T G G A G G T G T G G T G C G A G G G C G G C G A G T T C C G C T A C C T G A T C C G C T G C G A G C G
G G A G G C C A G A T A C C T G G C C G C C C T G C A C A A C A G A G C C C T G C C T A C C G C C G T G G A G T T C A A G A A G A T C G T G
G A C G A G A A G A A G C A G G A G G A T A T C G T G G A G A T G G A C G A C A A C T T C G A G T T C G G C C T G T G T C C C T G C G A C G
C C A A G C C C C T G G T G A G A G G C A A G T T C A A C A C C A C C C T G C T G A A T G G C C C C G C C T T C C A G A T G G T G T G C C C

CATCGGCTGGACCGGCACCGTGAGCTGCACCCTGGCCAACGAGGATACCCTGGCCACCACCGTGGTGCG

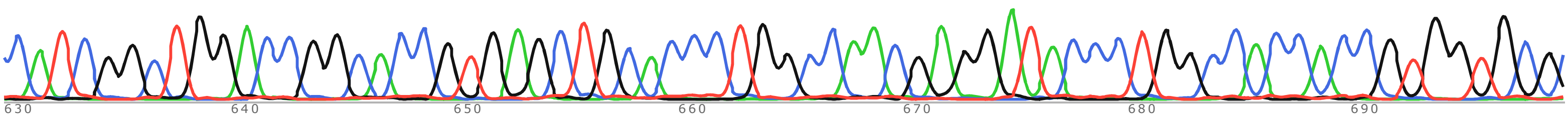

CACCTACAAGAGGGTGAGACCCTTCCCCTACCGGCAGGACTGCGTGACCCAGAAAACCATCGGCGAGGAC

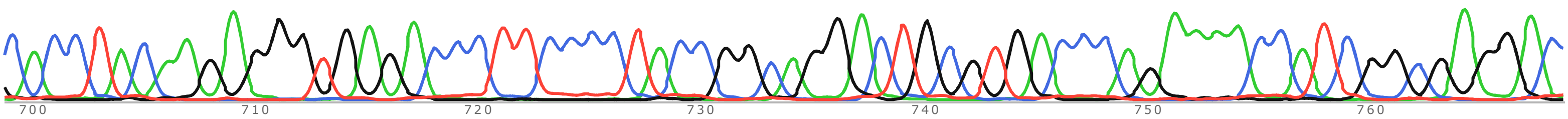

CTGTACGATTGCGCCCTGGGCGGCAACTGGACCTGCGTGCTGGGCGACATCCTGAGGTACGTGGCCGGCC

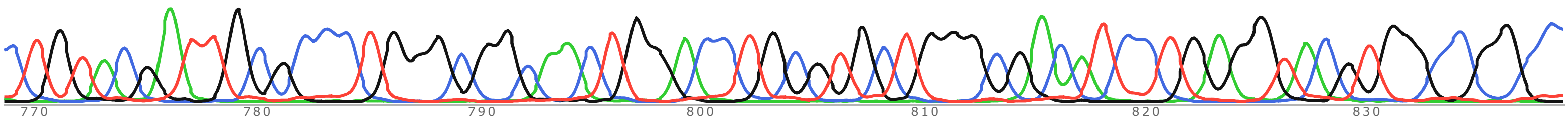

CTGTGGAGTCCTGCAAGTGGTGCGGCCACAAGTTCCTGAAGTCCGAGGGCCTGCCCCACTTCCCCATCGGC

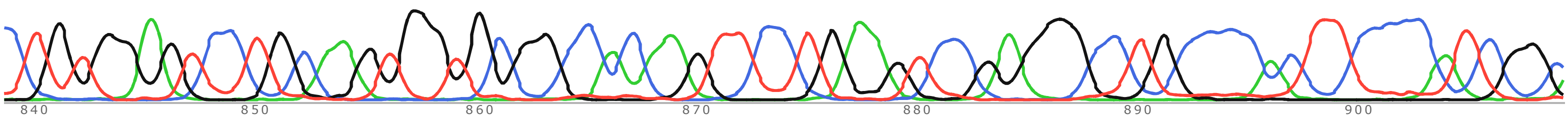

AAGTGCCGCCTGAAGAACGAGAGCGGCTACAGACTGGTGGACGGCACCTCCTGCAACCGGGACGGCGTGG

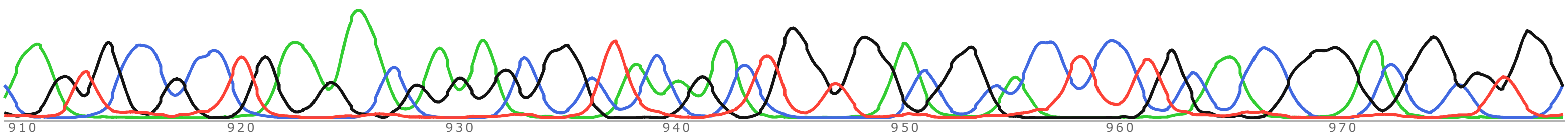

CCATCGTGCCCTCCGGGCCGG

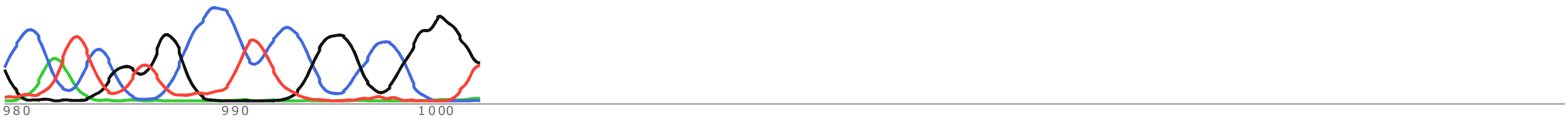

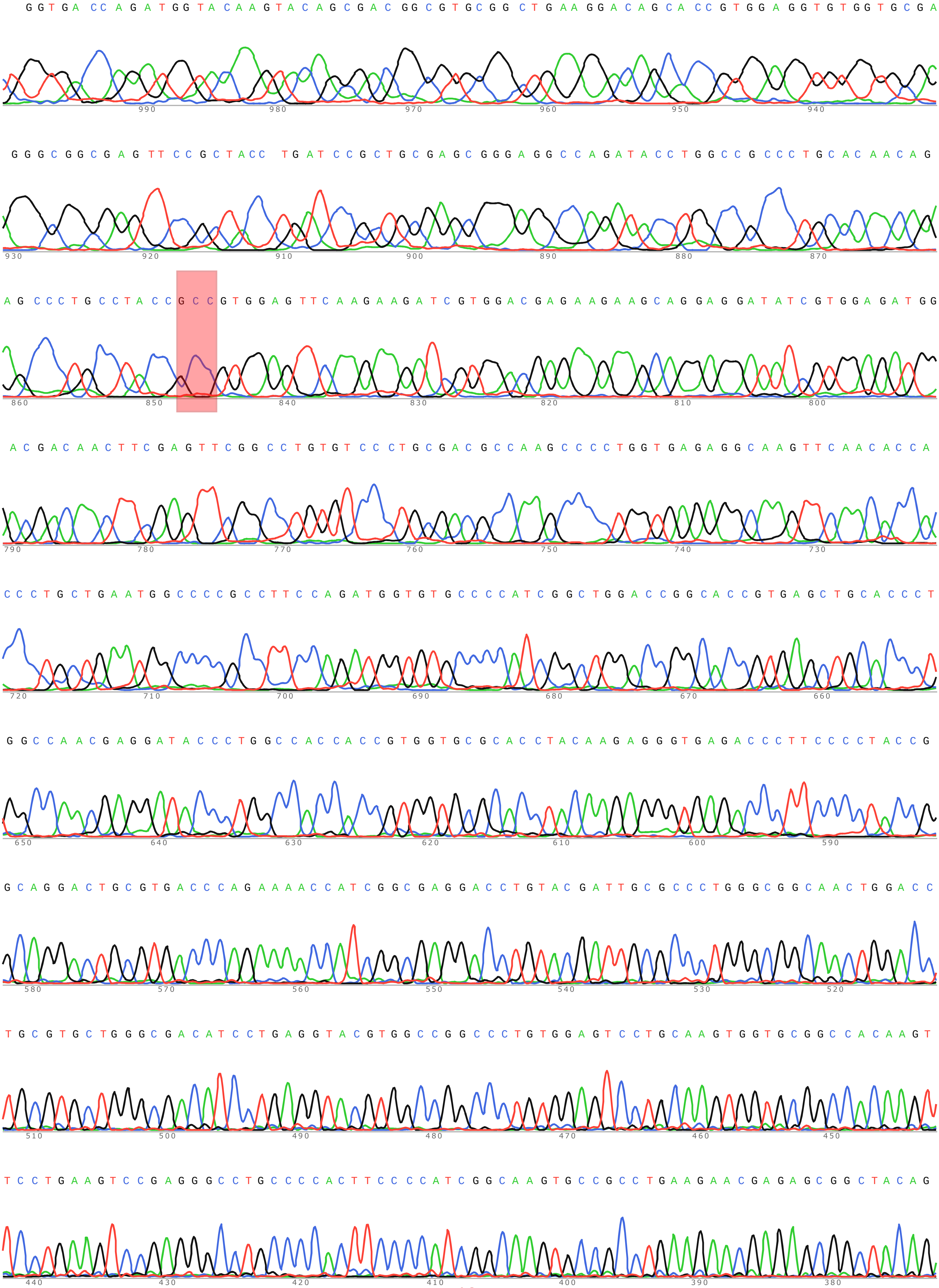
G G T G A C C A G A T G G T A C A A G T A C A G C G A C G G C G T G C G G C T G A A G G A C A G C A C C G T G G A G G T G T G G T G C G A
990 980 970 960 950 940
G G G C G G C G A G T T C C G C T A C C T G A T C C G C T G C G A G C G G G A G G C C A G A T A C C T G G C C G C C C T G C A C A A C A G
930 920 910 900 890 880 870
A G C C C T G C C T A C C G C C G T G G A G T T C A A G A A G A T C G T G G A C G A G A A G A A G C A G G A G G A T A T C G T G G A G A T G G
860 850 840 830 820 810 800
A C G A C A A C T T C G A G T T C G G C C T G T G T C C C T G C G A C G C C A A G C C C C T G G T G A G A G G C A A G T T C A A C A C C A
790 780 770 760 750 740 730
C C C T G C T G A A T G G C C C C G C C T T C C A G A T G G T G T G C C C C A T C G G C T G G A C C G G C A C C G T G A G C T G C A C C C T
720 710 700 690 680 670 660
G G C C A A C G A G G A T A C C C T G G C C A C C A C C G T G G T G C G C A C C T A C A A G A G G G T G A G A C C C T T C C C C T A C C G
650 640 630 620 610 600 590
G C A G G A C T G C G T G A C C C A G A A A A C C A T C G G C G A G G A C C T G T A C G A T T G C G C C C T G G G C G G C A A C T G G A C C
580 570 560 550 540 530 520
T G C G T G C T G G G C G A C A T C C T G A G G T A C G T G G C C G G C C C T G T G G A G T C C T G C A A G T G G T G C G G C C A C A A G T
510 500 490 480 470 460 450
T C C T G A A G T C C G A G G G C C T G C C C C A C T T C C C C A T C G G C A A G T G C C G C C T G A A G A A C G A G A G C G G C T A C A G
440 430 420 410 400 390 380

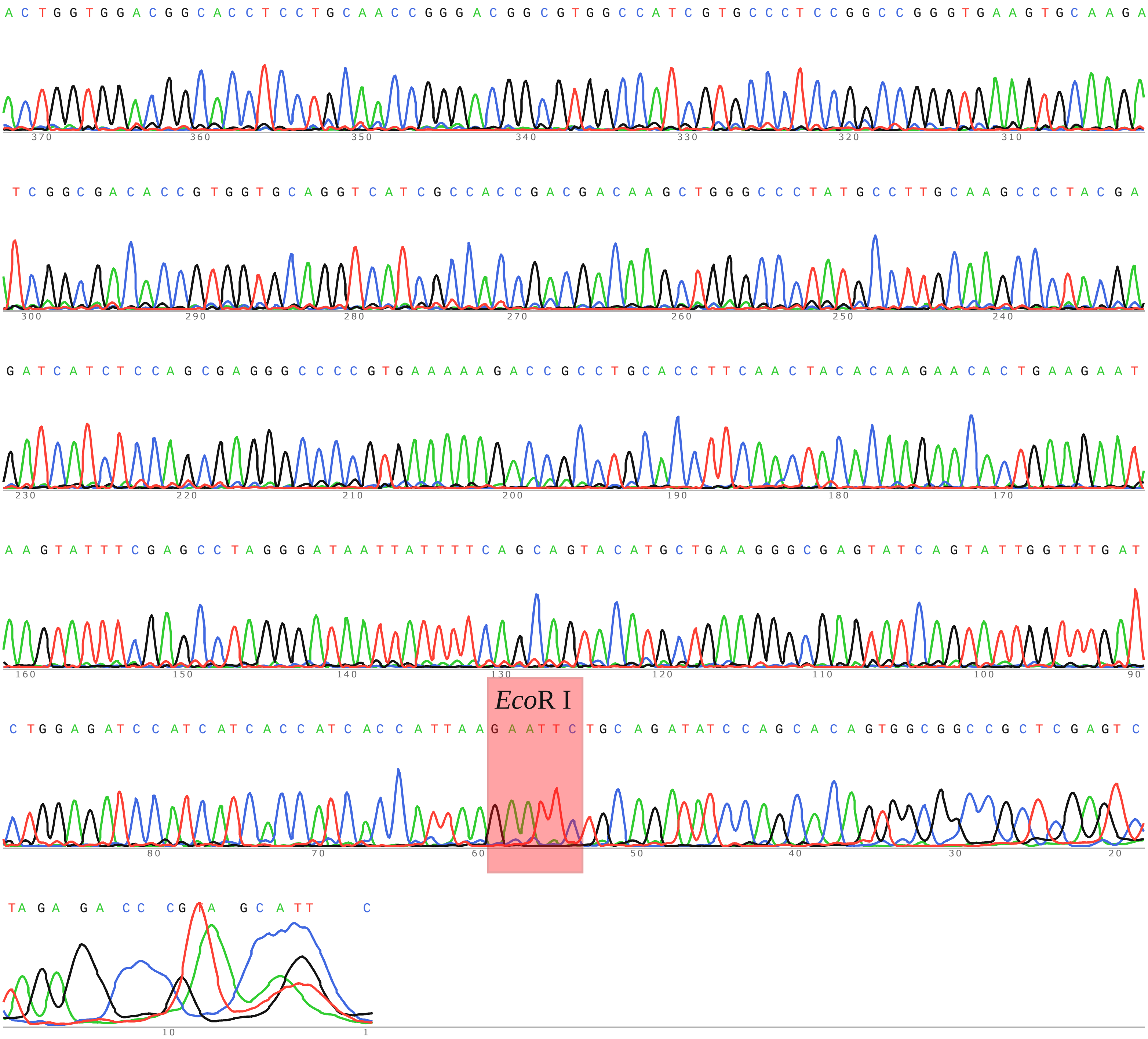
ACTGGTGGACGGCACCTCCTGCAACCGGGACGGCGTGGCCATCGTGCCCTCCGGCCGGGTGAAGTGCAAGA
370 360 350 340 330 320 310
TCGGCGACACCGTGGTGCAGGTCATCGCCACCGACGACAAGCTGGGCCCTATGCCTTGCAAGCCCTACGA
300 290 280 270 260 250 240
GATCATCTCCAGCGAGGGCCCCGTGAAAAAGACCGCCTGCACCTTCAACTACACAAGAACACTGAAGAAT
230 220 210 200 190 180 170
AAGTATTTCGAGCCTAGGGATAATTATTTTCAGCAGTACATGCTGAAGGGCGAGTATCAGTATTGGTTTGAT
160 150 140 130 120 110 100 90
EcoR I
CTGGAGATCCATCATCACCATCACCATTAAGAATTCTGCAGATATCCAGCACAGTGGCGGCCGCTCGAGTC
80 70 60 50 40 30 20
TAGAGACCCGTAGCATTC
10 1

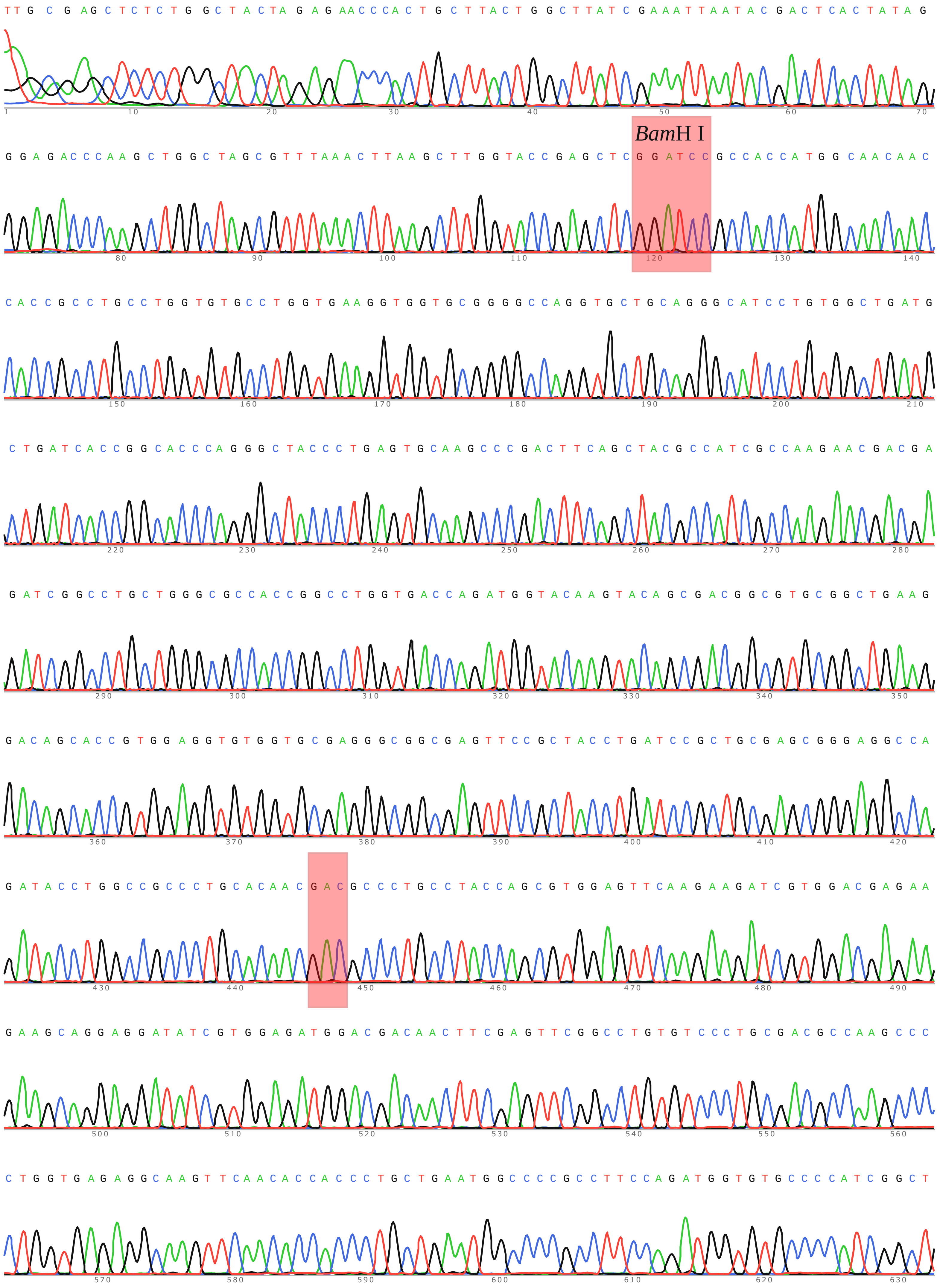
TTGCGAGCTCTCTGGCTACTAGAGAACCCACTGCTTACTGGCTTATCGAAATTAATACGACTCACTATAG
BamH I
GGAGACCCAAGCTGGCTAGCGTTTAAACTTAAGCTTGGTACCGAGCTCGGATCCGCCACCATGGCAACAAC
CACCGCCTGCCTGGTGTGCCTGGTGAAGGTGGTGCGGGGCCAGGTGCTGCAGGGCATCCTGTGGCTGATG
CTGATCACCGGCACCCAGGGCTACCCTGAGTGCAAGCCCGACTTCAGCTACGCCATCGCCAAGAACGACGA
GATCGGCCTGCTGGGCGCCACCGGCCTGGTGACCAGATGGTACAAGTACAGCGACGGCGTGCGGCTGAAG
GACAGCACCGTGGAGGTGTGGTGCGAGGGCGGCGAGTTCCGCTACCTGATCCGCTGCGAGCGGGAGGCCA
GATACCTGGCCGCCCTGCACAACGACGCCCTGCCTACCAGCGTGGAGTTCAAGAAGATCGTGGACGAGAA
GAAGCAGGAGGATATCGTGGAGATGGACGACAACTTCGAGTTCGGCCTGTGTCCCTGCGACGCCAAGCCC
CTGGTGAGAGGCAAGTTCAACACCACCCTGCTGAATGGCCCCGCCTTCCAGATGGTGTGCCCCATCGGCT

G G A C C G G C A C C G T G A G C T G C A C C C T G G C C A A C G A G G A T A C C C T G G C C A C C A C C G T G G T G C G C A C C T A C A A

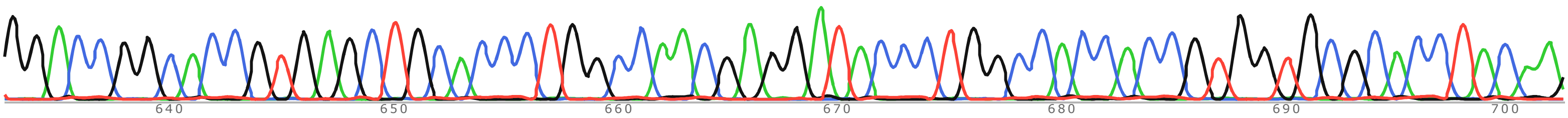

G A G G G T G A G A C C C T T C C C C T A C C G G C A G G A C T G C G T G A C C C A G A A A A C C A T C G G C G A G G A C C T G T A C G A T T

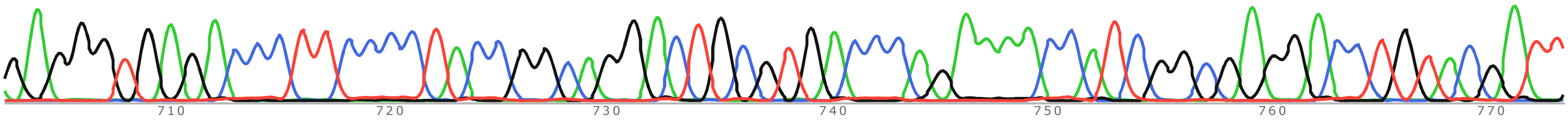

G C G C C C T G G G C G G C A A C T G G A C C T G C G T G C T G G G C G A C A T C C T G A G G T A C G T G G C C G G C C C T G T G G A G T C

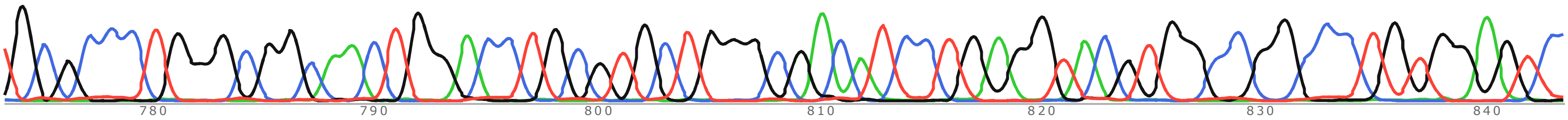

C T G C A A G T G G T G C G G C C A C A A G T T C C T G A A G T C C G A G G G C C T G C C C C A C T T C C C C A T C G G C A A G T G C C G C C

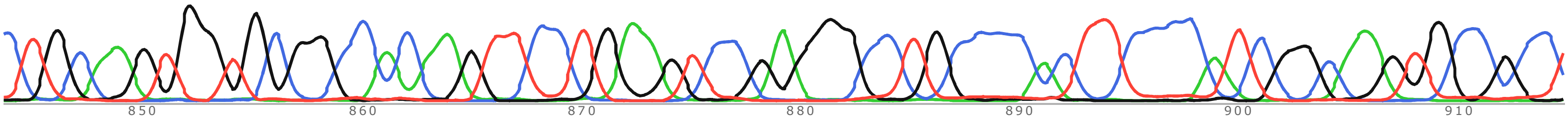

T G A A G A A C G A G A G C G G C T A C A G A C T G G T G G A C G G C A C C T C C T G C A A C C G G G A C G G C G T G G C C A T C G T G C C C T

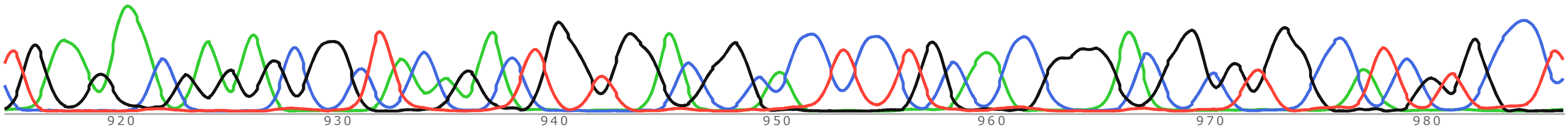

C C G G C C G G G T G A A G T G C A G A T C G G

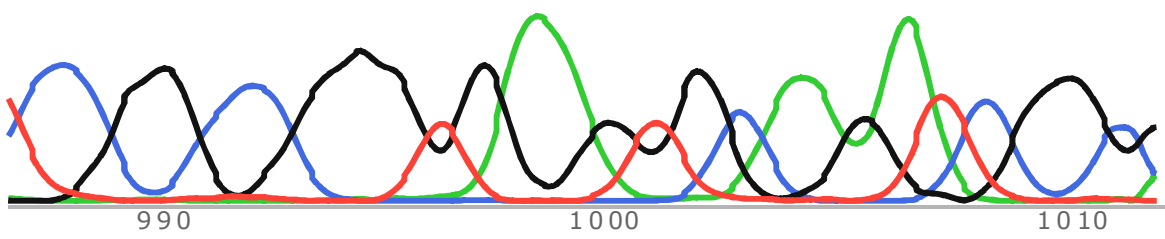

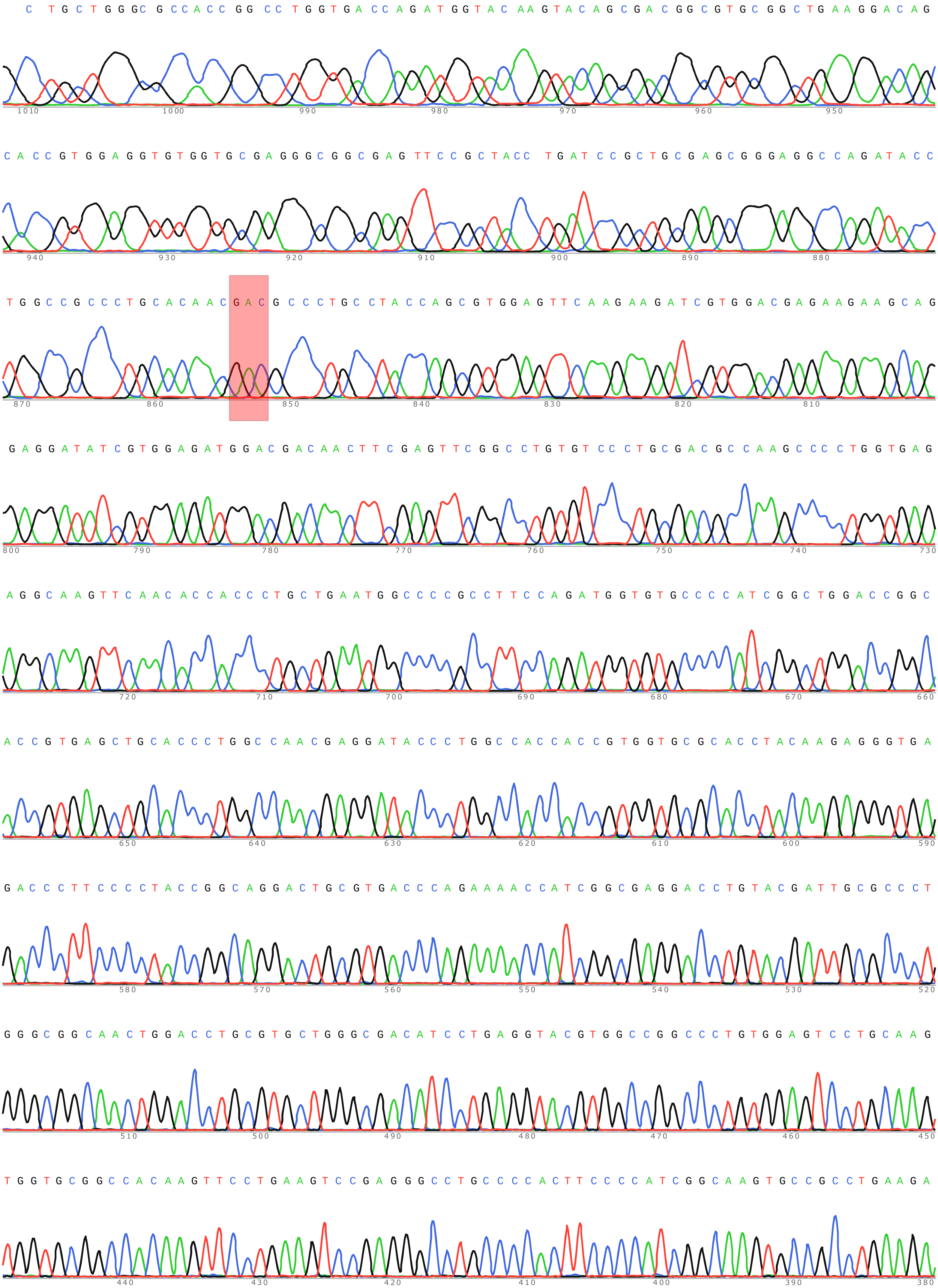
CTGCTGGGCGCCACCGGCCTGGTGACCAGATGGTACAAGTACAGCGACGGCGTGCGGCTGAAGGACAG
1010 1000 990 980 970 960 950
CACCGTGGAGGTGTGGTGCGAGGGCGGCGAGTTCCGCTACCTGATCCGCTGCGAGCGGGAGGCCAGATACC
940 930 920 910 900 890 880
TGGCCGCCCTGCACAACGACGCCCTGCCTACCAGCGTGGAGTTCAAGAAGATCGTGGACGAGAAGAAGCAG
870 860 850 840 830 820 810
GAGGATATCGTGGAGATGGACGACAACTTCGAGTTCGGCCTGTGTCCCTGCGACGCCAAGCCCCTGGTGAG
800 790 780 770 760 750 740 730
AGGCAAGTTCAACACCACCCTGCTGAATGGCCCCGCCTTCCAGATGGTGTGCCCCATCGGCTGGACCGGC
720 710 700 690 680 670 660
ACCGTGAGCTGCACCCTGGCCAACGAGGATACCCTGGCCACCACCGTGGTGCGCACCTACAAGAGGGTGA
650 640 630 620 610 600 590
GACCCTTCCCCTACCGGCAGGACTGCGTGACCCAGAAAACCATCGGCGAGGACCTGTACGATTGCGCCCT
580 570 560 550 540 530 520
GGGCGGCAACTGGACCTGCGTGCTGGGCGACATCCTGAGGTACGTGGCCGGCCCTGTGGAGTCCTGCAAG
510 500 490 480 470 460 450
TGGTGCGGCCACAAGTTCCTGAAGTCCGAGGGCCTGCCCCACTTCCCCATCGGCAAGTGCCGCCTGAAGA
440 430 420 410 400 390 380

A C G A G A G C G G C T A C A G A C T G G T G G A C G G C A C C T C C T G C A A C C G G G A C G G C G T G G C C A T C G T G C C C T C C G G

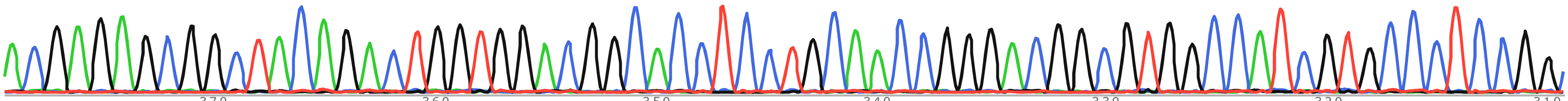

C C G G G T G A A G T G C A A G A T C G G C G A C A C C G T G G T G C A G G T C A T C G C C A C C G A C G A C A A G C T G G G C C C T A T G

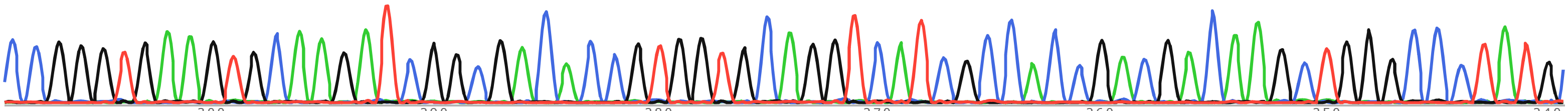

C C T T G C A A G C C C T A C G A G A T C A T C T C C A G C G A G G G C C C C G T G A A A A A G A C C G C C T G C A C C T T C A A C T A C A

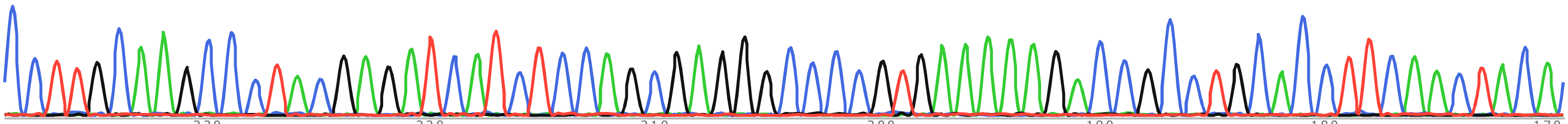

C A A G A A C A C T G A A G A A T A A G T A T T T C G A G C C T A G G G A T A A T T A T T T T C A G C A G T A C A T G C T G A A G G G C G A G

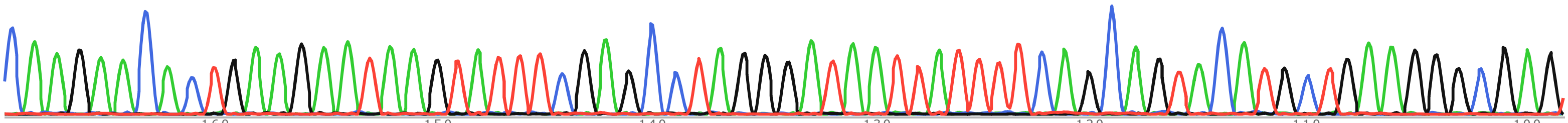

T A T C A G T A T T G G T T T G A T C T G G A G A T C C A T C A T C A C C A T C A C C A T T A A G A A T T C T G C A G A T A T C C A G C A C A

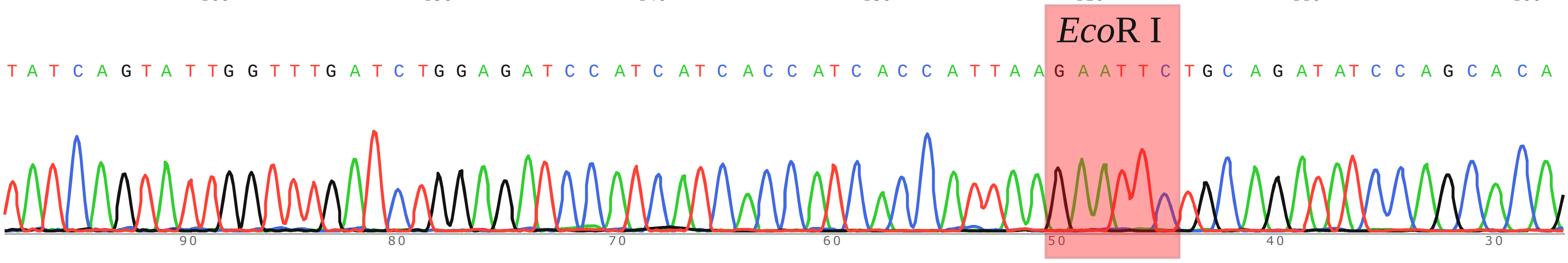

G T G G C G G C C G C T C G A G T C T A G A G T C C T

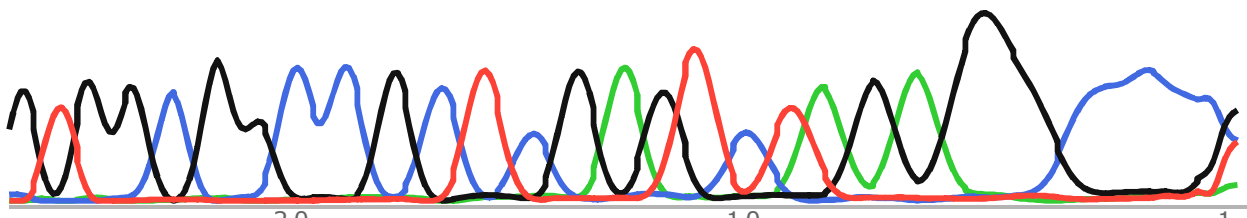

MEP-029-pcDNA3.1-BVDV-XH32-E2 (R72K)_pRluc-MCS-F_2024-02-01_C04.ab1 (1010 bases)

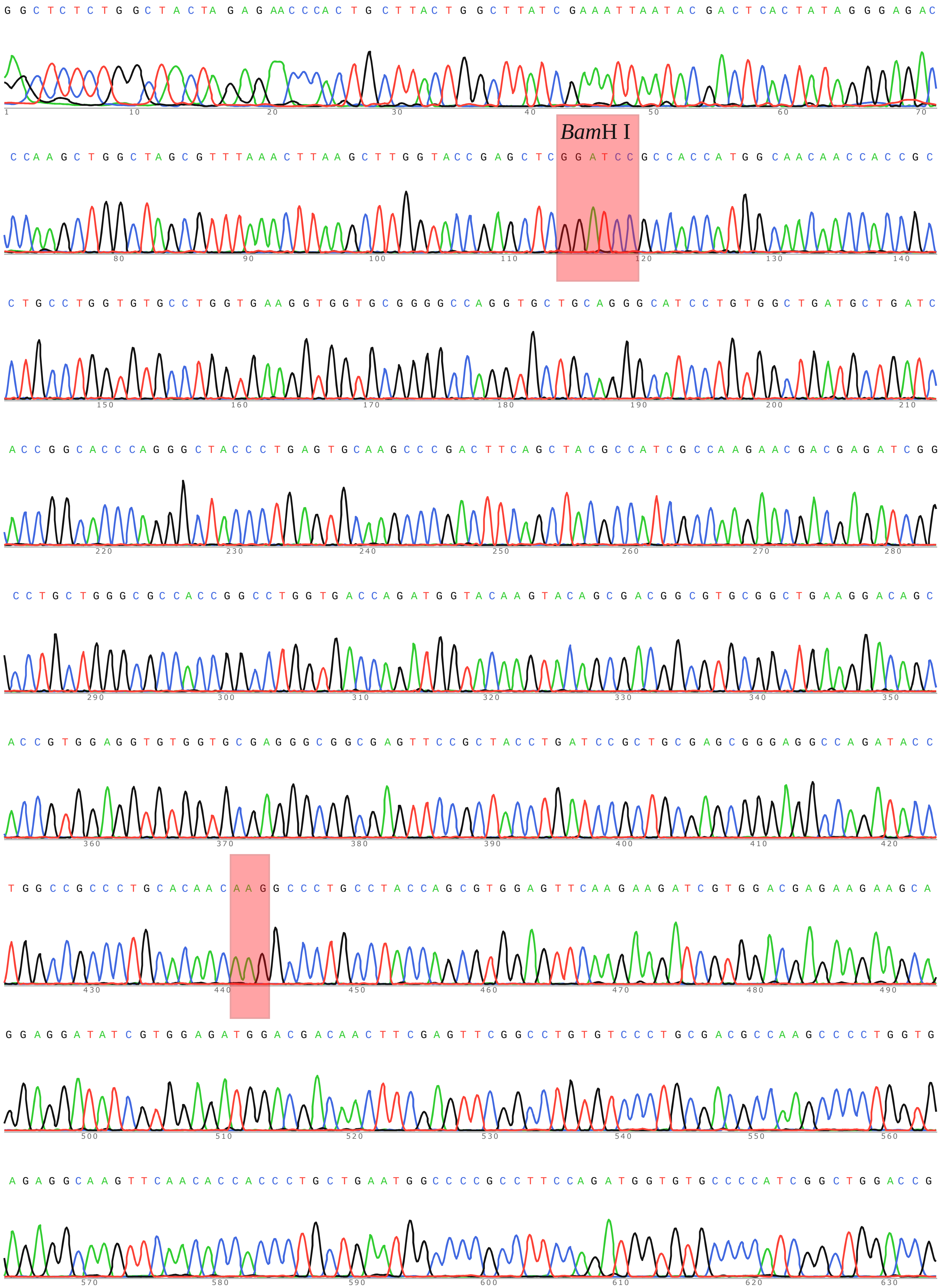

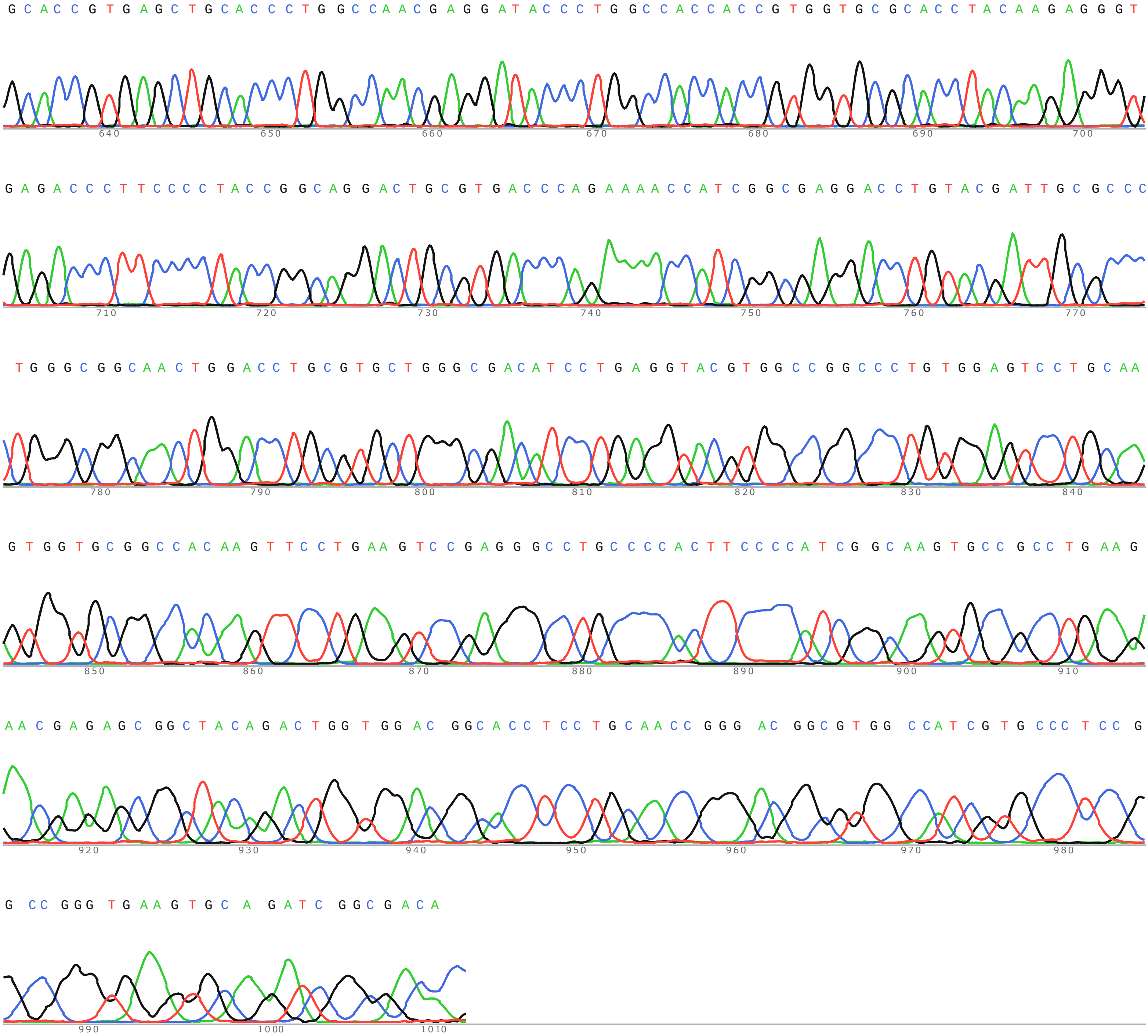
G C A C C G T G A G C T G C A C C C T G G C C A A C G A G G A T A C C C T G G C C A C C A C C G T G G T G C G C A C C T A C A A G A G G G T
640 650 660 670 680 690 700
G A G A C C C T T C C C C T A C C G G C A G G A C T G C G T G A C C C A G A A A A C C A T C G G C G A G G A C C T G T A C G A T T G C G C C C
710 720 730 740 750 760 770
T G G G C G G C A A C T G G A C C T G C G T G C T G G G C G A C A T C C T G A G G T A C G T G G C C G G C C C T G T G G A G T C C T G C A A
780 790 800 810 820 830 840
G T G G T G C G G C C A C A A G T T C C T G A A G T C C G A G G G C C T G C C C C A C T T C C C C A T C G G C A A G T G C C G C C T G A A G
850 860 870 880 890 900 910
A A C G A G A G C G G C T A C A G A C T G G T G G A C G G C A C C T C C T G C A A C C G G G A C G G C G T G G C C A T C G T G C C C T C C G
920 930 940 950 960 970 980
G C C G G G T G A A G T G C A G A T C G G C G A C A
990 1000 1010

GG C TGCT GGGCG CCACC GG CC T GGTGA CCAGATGGTACAAGTACAGCGACGGCGTGCGGCTG AA GGACAG

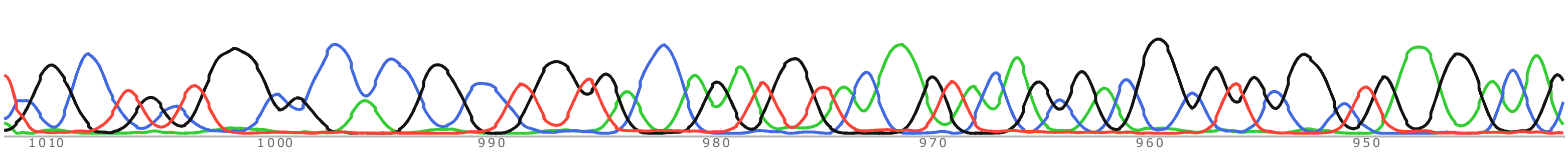

CACCGTGGAGGTGTGGTGCGAGGGCGGCGAGTTCCGCTACC TGATCCGCTGCGAGCGGGAGGC CAGATACC

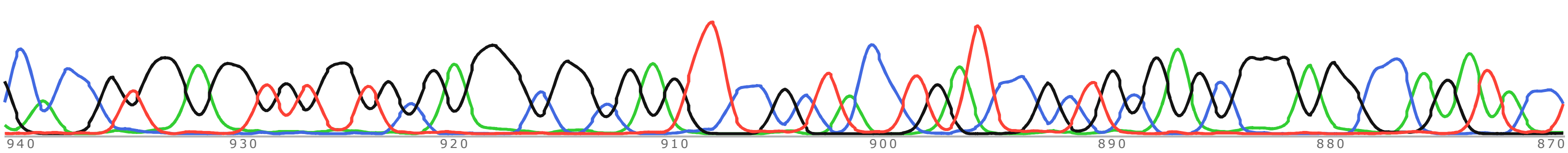

TGGCCGCCCTGCACAACAAGGCCCTGCCTACCAGCGTGGAGTTCAAGAAGATCGTGGACGAGAAGAAGCAG

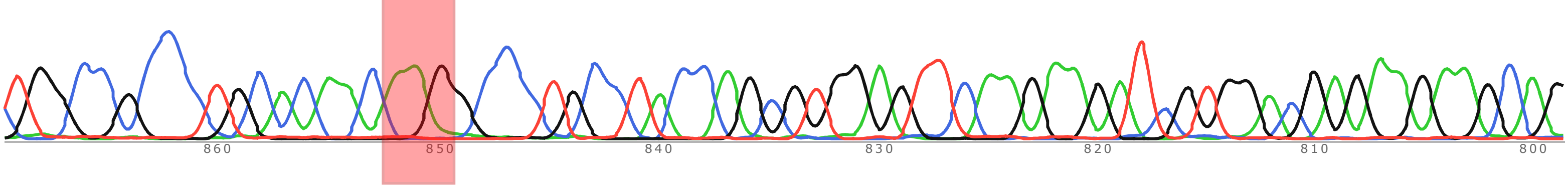

GAGGATATCGTGGAGATGGACGACAACTTCGAGTTCGGCCTGTGTCCCTGCGACGCCAAGCCCCTGGTGAG

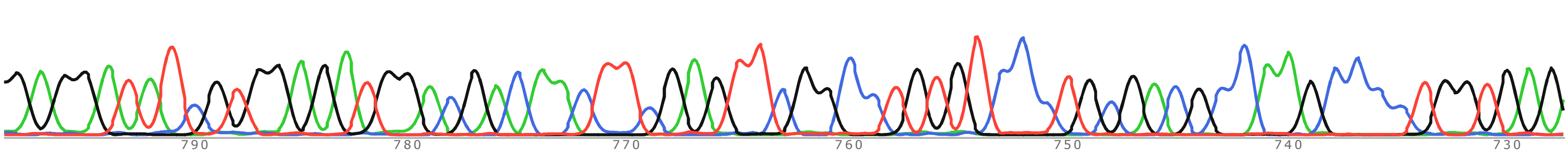

AGGCAAGTTCAACACCACCCTGCTGAATGGCCCCGCCTTCCAGATGGTGTGCCCCATCGGCTGGACCGGC

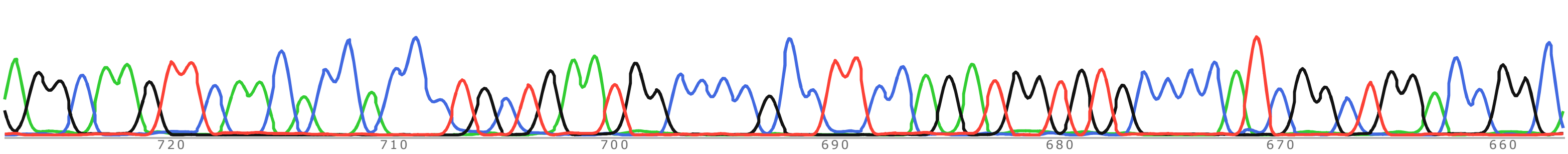

ACCGTGAGCTGCACCCTGGCCAACGAGGATACCCTGGCCACCACCGTGGTGCGCACCTACAAGAGGGTGA

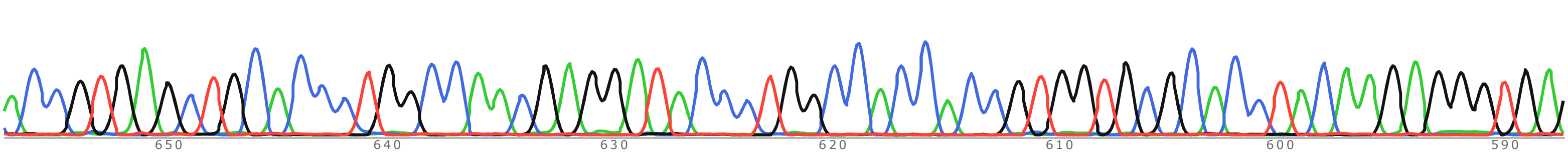

GACCCTTCCCCTACCGGCAGGACTGCGTGACCCAGAAAACCATCGGCGAGGACCTGTACGATTGCGCCCT

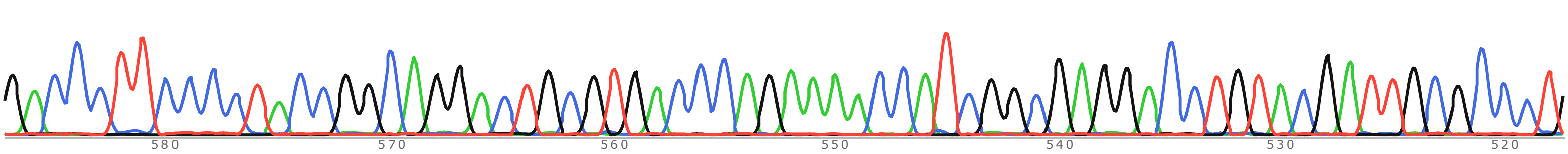

GGGCGGCAACTGGACCTGCGTGCTGGGCGACATCCTGAGGTACGTGGCCGGCCCTGTGGAGTCCTGCAAG

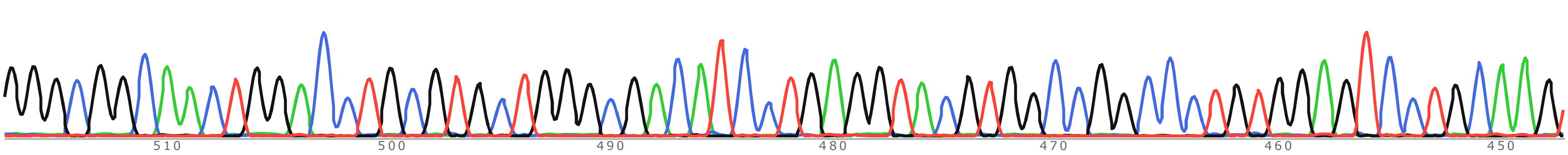

TGGTGCGGCCACAAGTTCCTGAAGTCCGAGGGCCTGCCCCACTTCCCCATCGGCAAGTGCCGCCTGAAGA

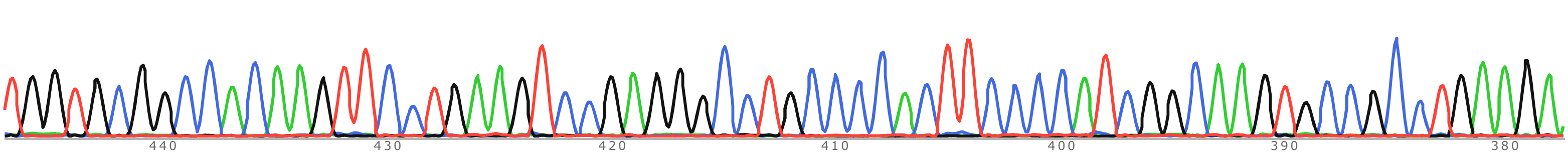

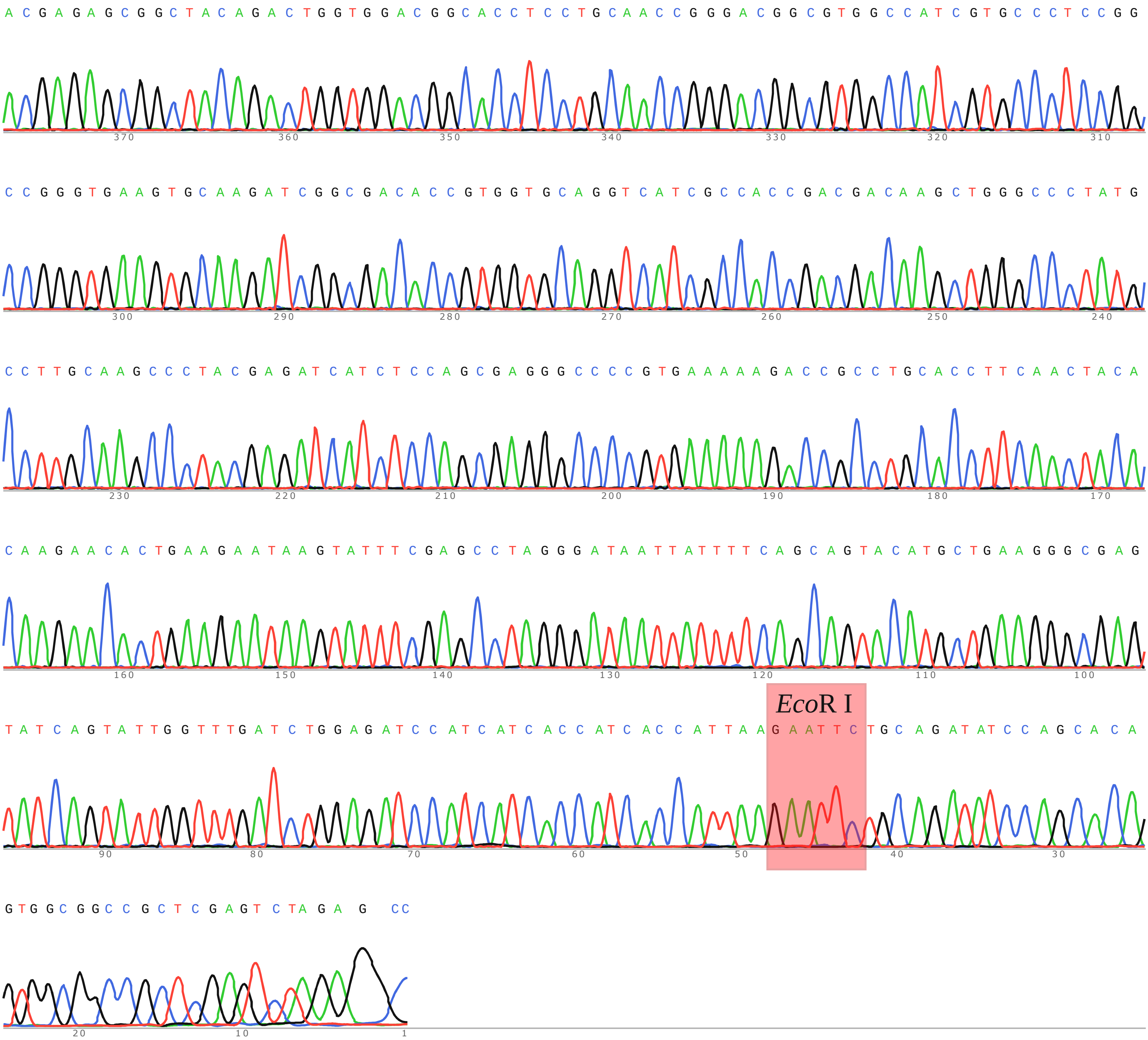

ACGAGAGCGGCTACAGACTGGTGGACGGCACCTCCTGCAACCGGGACGGCGTGGCCATCGTGCCCTCCGG
370 360 350 340 330 320 310
CCGGGTGAAGTGCAAGATCGGCGACACCGTGGTGCAGGTCATCGCCACCGACGACAAGCTGGGCCCTATG
300 290 280 270 260 250 240
CCTTGCAAGCCCTACGAGATCATCTCCAGCGAGGGCCCCGTGAAAAAGACCGCCTGCACCTTCAACTACA
230 220 210 200 190 180 170
CAAGAACACTGAAGAATAAGTATTTCGAGCCTAGGGATAATTATTTTCAGCAGTACATGCTGAAGGGCGAG
160 150 140 130 120 110 100
EcoR I
TATCAGTATTGGTTTGATCTGGAGATCCATCATCACCATCACCATTAAGAATTCTGCAGATATCCAGCACA
90 80 70 60 50 40 30
GTGGCGGCCGCTCGAGTCTAGAGCC
20 10 1

BEP-505-pFsat Bac1-BVDV-M31182-E2 (D72R)_poly-F_2024-06-15_A05.ab1 (1000 bases)

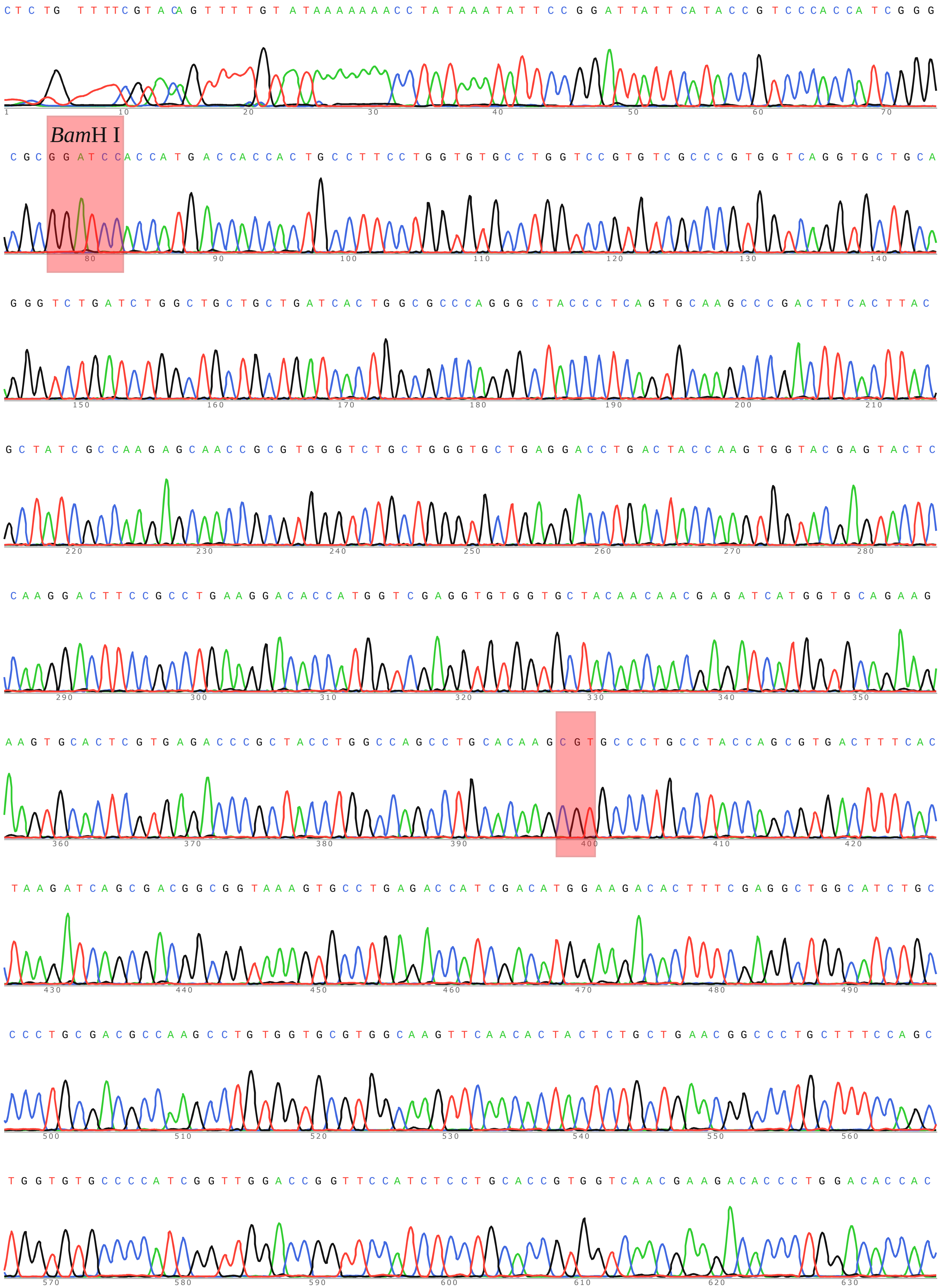

CGTCATCAAGACTTTCCGTCGTGCCAAGCCCTTCCCCTACCGCCAGGACTGCACTACCCAGAAGCTGGTG

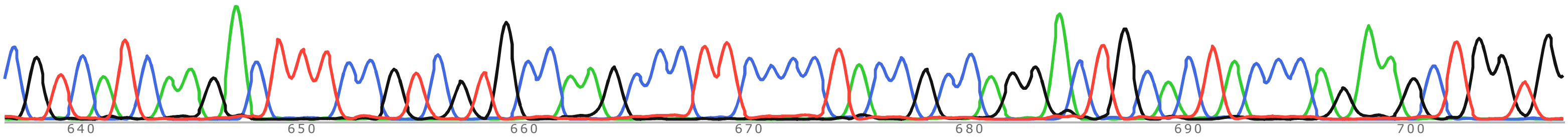

GGCGAAGACCTGCACGAGTGCATGCTGGGCGGCAACTGGACTTGCATCGTGGGTGAGCCTCTGAAGTACGG

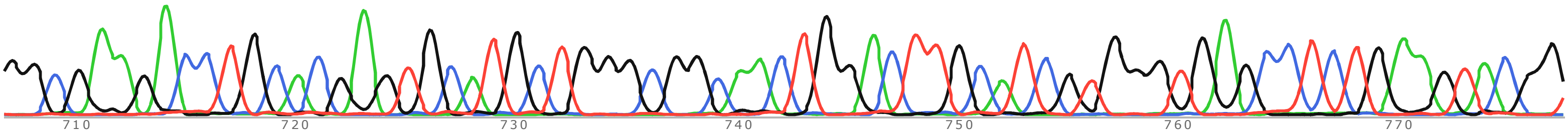

TGGCGGCCGCGTGTCCTCCTGCAAGTGGTGCGGTTACAAGTTCGAGAAGTCCGACGGCCTGCCCCACTACC

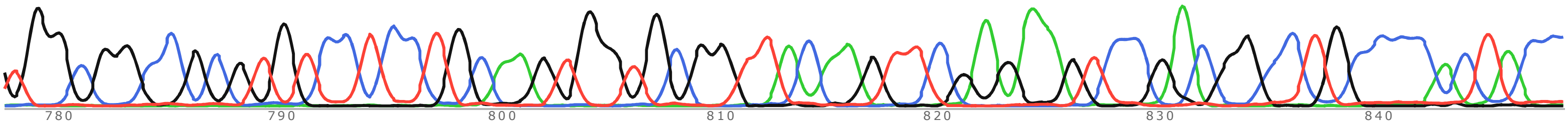

CCATCGGCAAGTGCGAACTGGCTAACGAGACTGGTGACCGCATCGTGGACGAAACCCCCTGCGACCGTGGTG

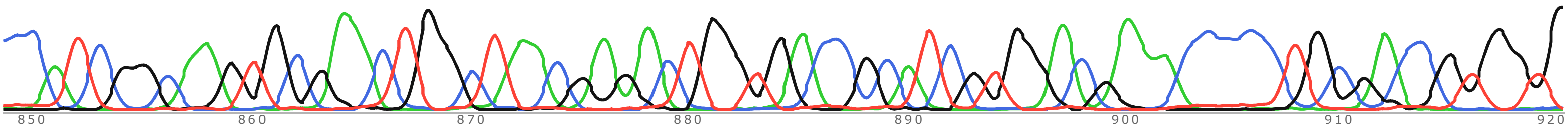

GCGTCGCCATCGTGAGCACTGGTACCATCAAGTGCAAGATCGGTGACACTGTGGTGCAGGTCATTGCCACC

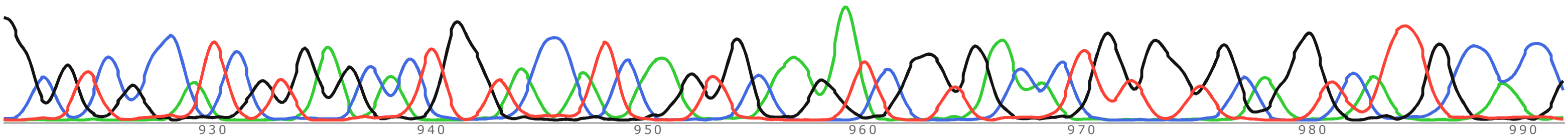

GACACCTCC

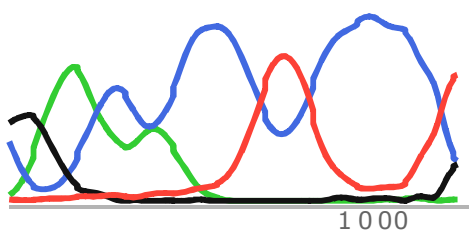

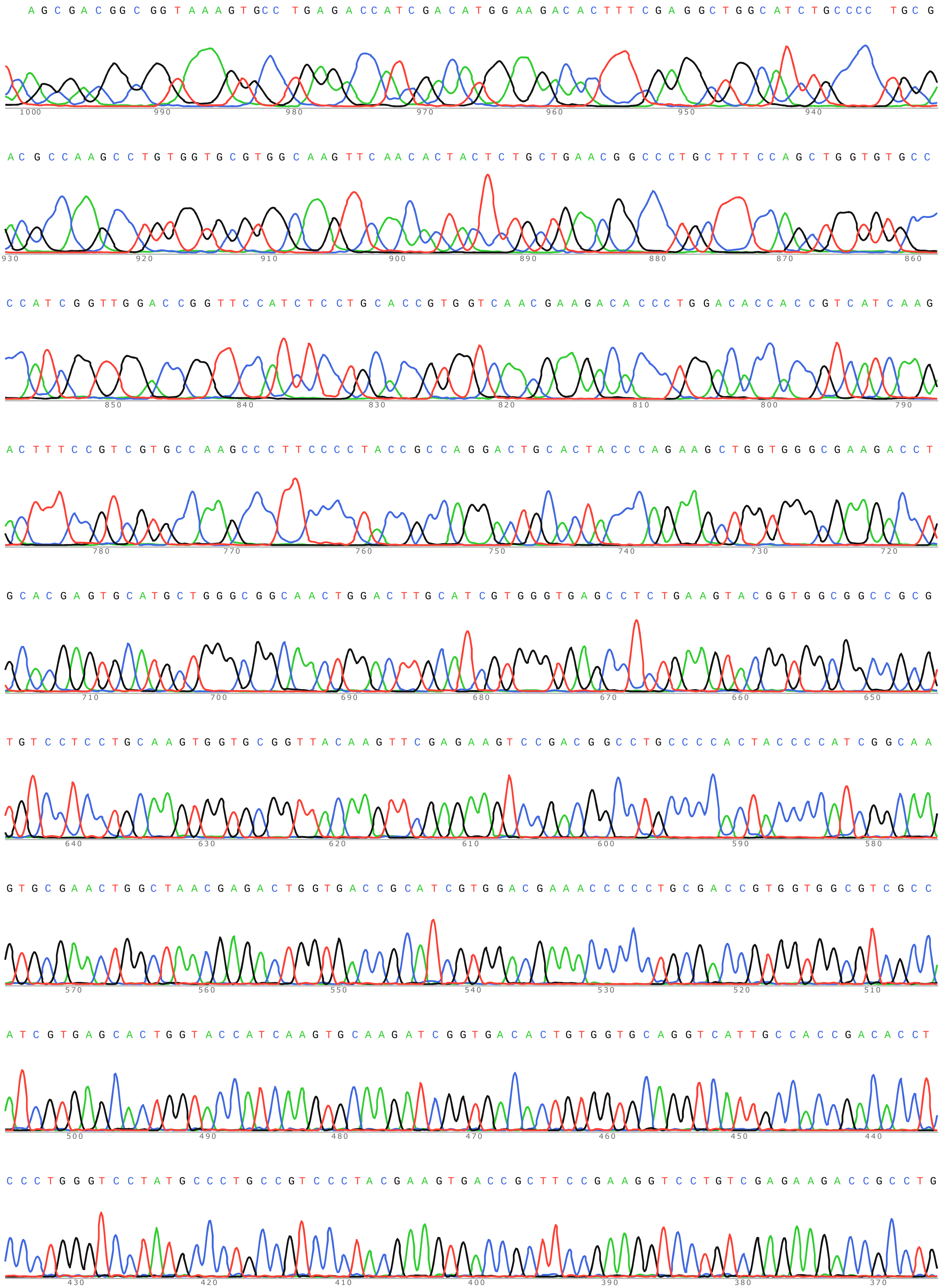
AGCGACGGCGGTAAAGTGCCTGAGACCATCGACATGGAAGACACTTTCGAGGCTGGCATCTGCCCCTGCG
ACGCCAAGCCTGTGGTGCGTGGCAAGTTCAACACTACTCTGCTGAACGGCCCTGCTTTCCAGCTGGTGTGCC
CCATCGGTTGGACCGGTTCCATCTCCTGCACCGTGGTCAACGAAGACACCCTGGACACCACCGTCATCAAG
ACTTTCCGTCGTGCCAAGCCCTTCCCCTACCGCCAGGACTGCACTACCCAGAAGCTGGTGGGCGAAGACCT
GCACGAGTGCATGCTGGGCGGCAACTGGACTTGCATCGTGGGTGAGCCTCTGAAGTACGGTGGCGGCCGCG
TGTCCTCCTGCAAGTGGTGCGGTTACAAGTTCGAGAAGTCCGACGGCCTGCCCCACTACCCCATCGGCAA
GTGCGAACTGGCTAACGAGACTGGTGACCGCATCGTGGACGAAACCCCCTGCGACCGTGGTGGCGTCGCC
ATCGTGAGCACTGGTACCATCAAGTGCAAGATCGGTGACACTGTGGTGCAGGTCATTGCCACCGACACCT
CCCTGGGTCCTATGCCCTGCCGTCCCTACGAAGTGACCGCTTCCGAAGGTCCTGTCGAGAAGACCGCCTG

31

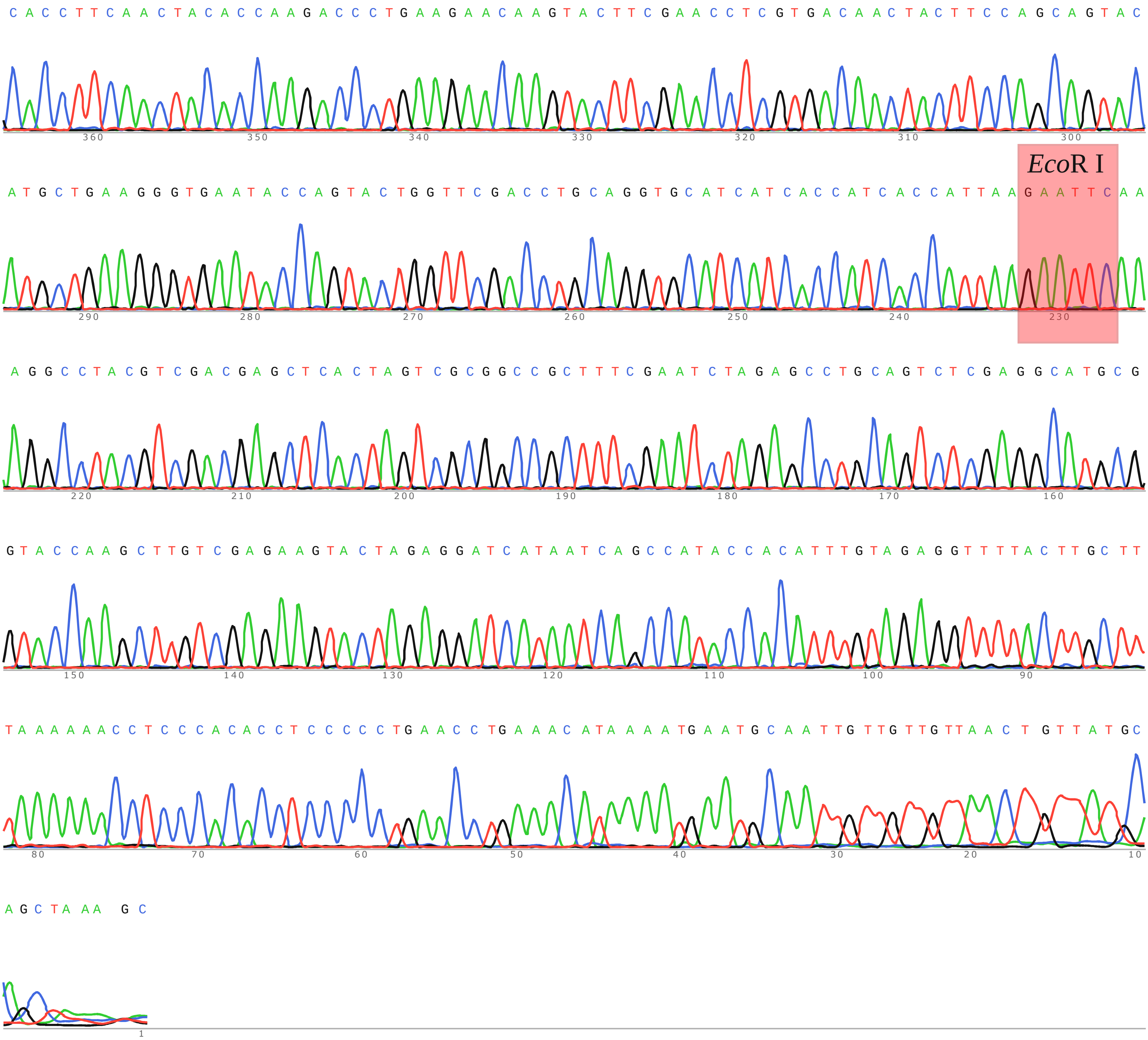
CACCTTCAACTACACCAAGACCCTGAAGAACAAGTACTTCGAACCTCGTGACAACTACTTCCAGCAGTAC
ATGCTGAAGGGTGAATACCAGTACTGGTTCGACCTGCAGGTGCATCATCACCATCACCATTAAGAATTCAA
*Eco*R I
AGGCCTACGTCGACGAGCTCACTAGTCGCGGCCGCTTTCGAATCTAGAGCCTGCAGTCTCGAGGCATGCG
GTACCAAGCTTGTCGAGAAGTACTAGAGGATCATAATCAGCCATACCACATTTGTAGAGGTTTTACTTGCTT
TAAAAAACCTCCCACACCTCCCCCTGAACCTGAAACATAAAATGAATGCAATTGTTGTTGTTAACTGTTATGC
AGCTAAAGC

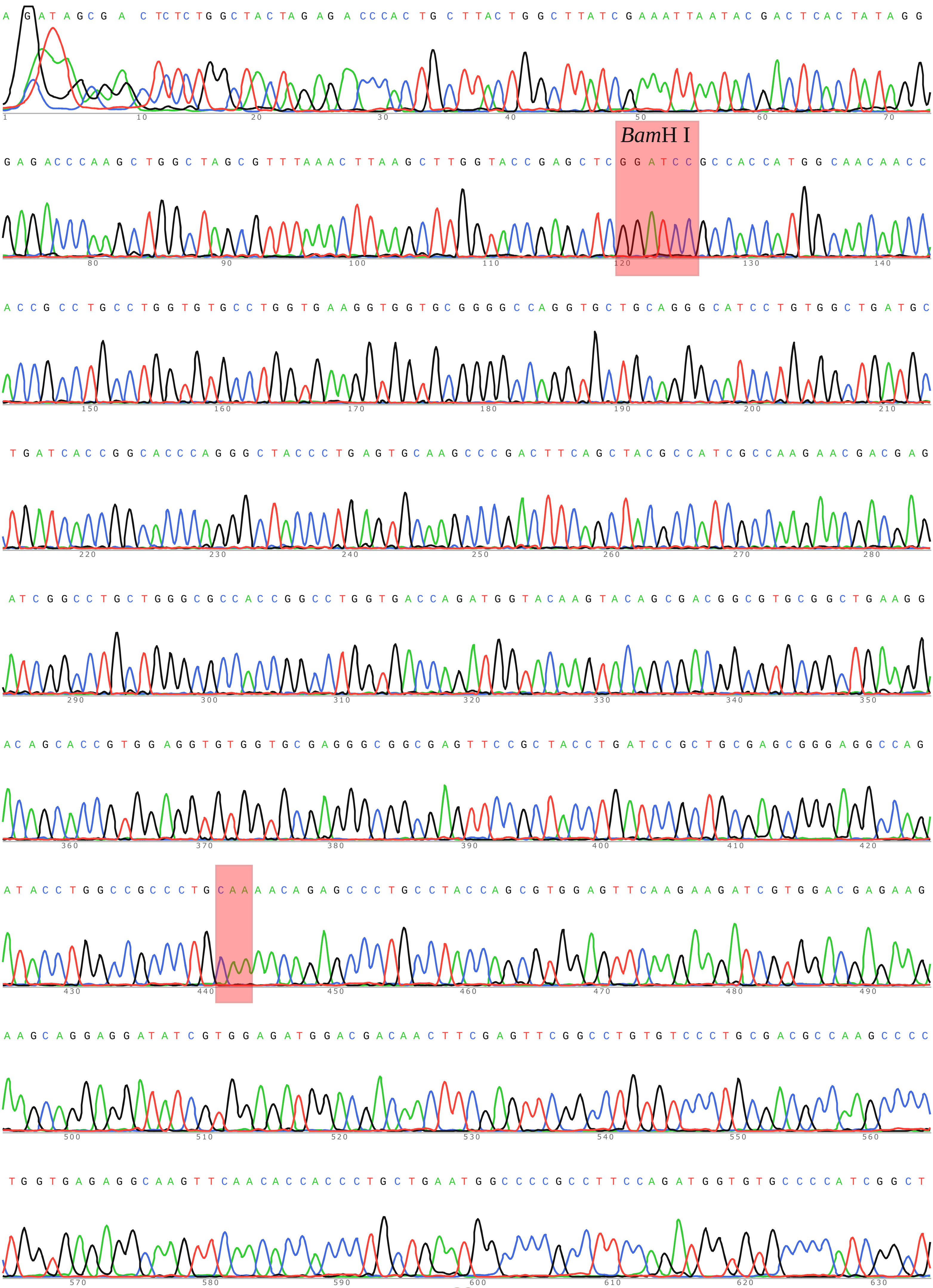
A G A T A G C G A C T C T C T G G C T A C T A G A G A C C C A C T G C T T A C T G G C T T A T C G A A A T T A A T A C G A C T C A C T A T A G G
BamH I
G A G A C C C A A G C T G G C T A G C G T T T A A A C T T A A G C T T G G T A C C G A G C T C G G A T C C G C C A C C A T G G C A A C A A C C
A C C G C C T G C C T G G T G T G C C T G G T G A A G G T G G T G C G G G G C C A G G T G C T G C A G G G C A T C C T G T G G C T G A T G C
T G A T C A C C G G C A C C C A G G G C T A C C C T G A G T G C A A G C C C G A C T T C A G C T A C G C C A T C G C C A A G A A C G A C G A G
A T C G G C C T G C T G G G C G C C A C C G G C C T G G T G A C C A G A T G G T A C A A G T A C A G C G A C G G C G T G C G G C T G A A G G
A C A G C A C C G T G G A G G T G T G G T G C G A G G G C G G C G A G T T C C G C T A C C T G A T C C G C T G C G A G C G G G A G G C C A G
A T A C C T G G C C G C C C T G C A A A A C A G A G C C C T G C C T A C C A G C G T G G A G T T C A A G A A G A T C G T G G A C G A G A A G
A A G C A G G A G G A T A T C G T G G A G A T G G A C G A C A A C T T C G A G T T C G G C C T G T G T C C C T G C G A C G C C A A G C C C C
T G G T G A G A G G C A A G T T C A A C A C C A C C C T G C T G A A T G G C C C C G C C T T C C A G A T G G T G T G C C C C A T C G G C T

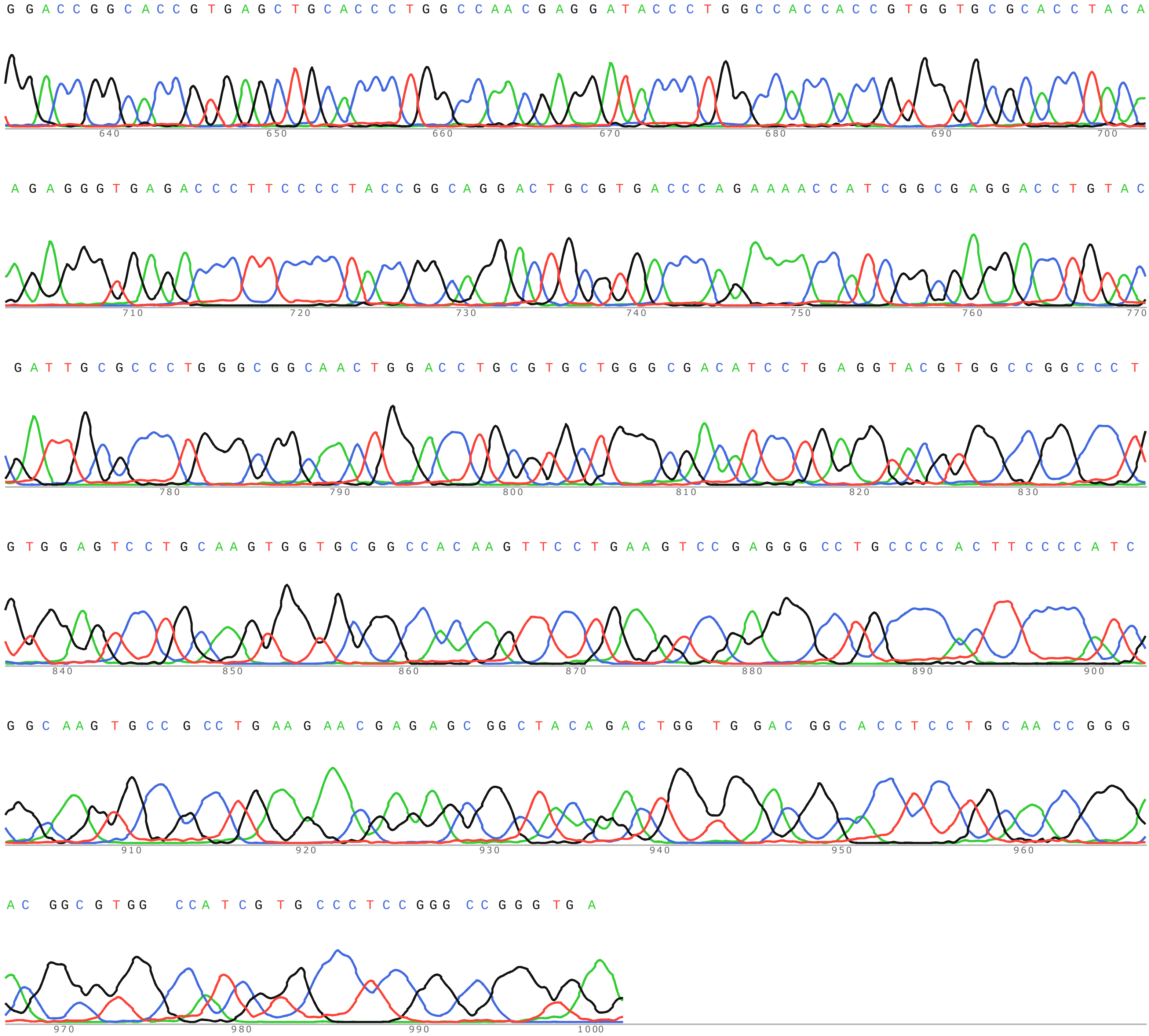
GGACCGGCACCGTGAGCTGCACCCTGGCCAACGAGGATACCCTGGCCACCACCGTGGTGCGCACCTACA
AGAGGGTGAGACCCTTCCCCTACCGGCAGGACTGCGTGACCCAGAAAACCATCGGCGAGGACCTGTAC
GATTGCGCCCTGGGCGGCAACTGGACCTGCGTGCTGGGCGACATCCTGAGGTACGTGGCCGGCCCT
GTGGAGTCCTGCAAGTGGTGCGGCCACAAGTTCCTGAAGTCCGAGGGCCTGCCCCACTTCCCCATC
GGCAAGTGCCGCCTGAAGAACGAGAGCGGCTACAGACTGGTGGACGGCACCTCCTGCAACCGGG
ACGGCGTGGCCATCGTGCCCTCCGGGCCGGGTGA

MEP-048-pcDNA3.1-BVDV-XH32-E2 (H70Q)_BGH-r_2024-05-10_A03.ab1 (1000 bases)

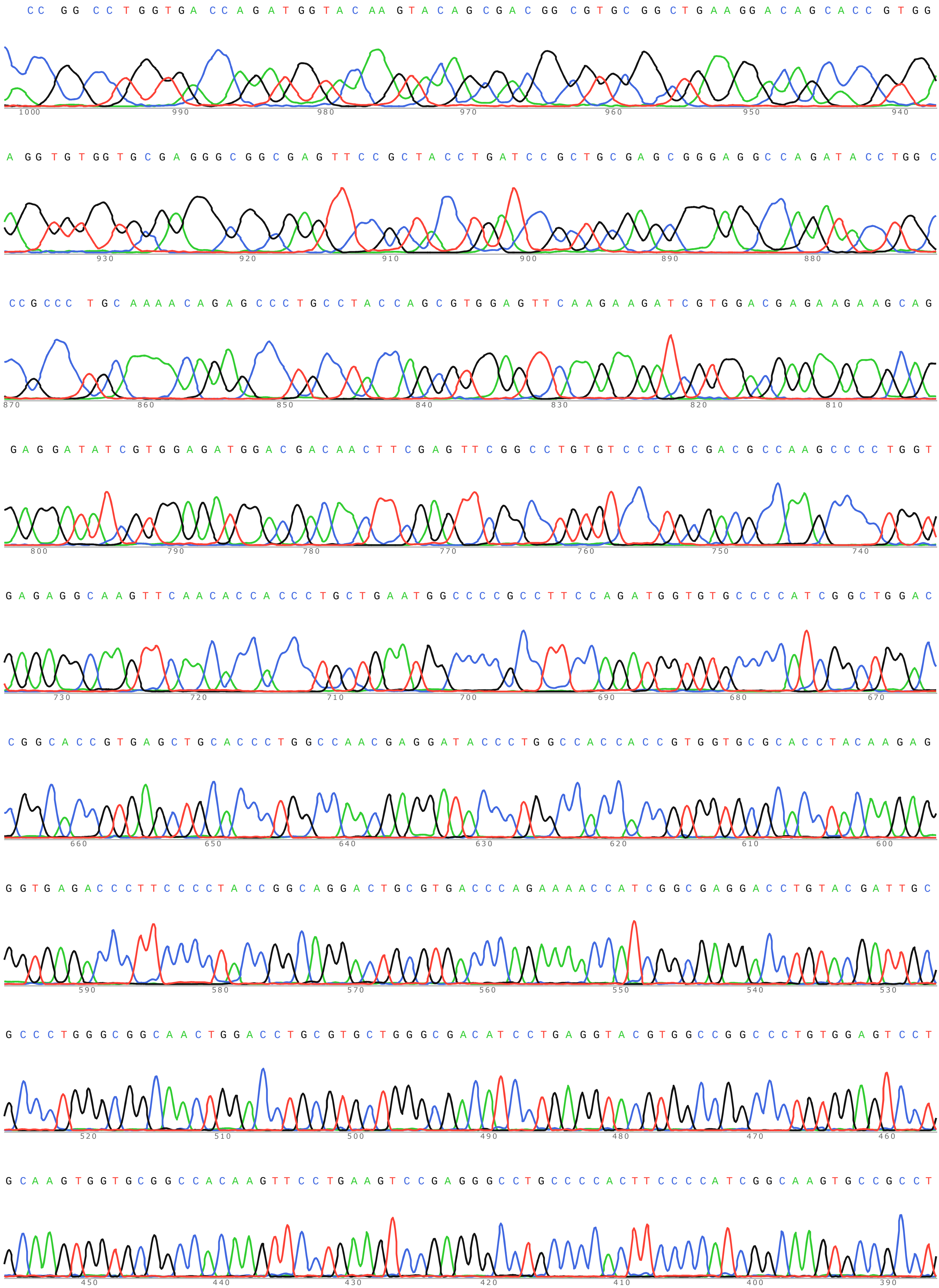

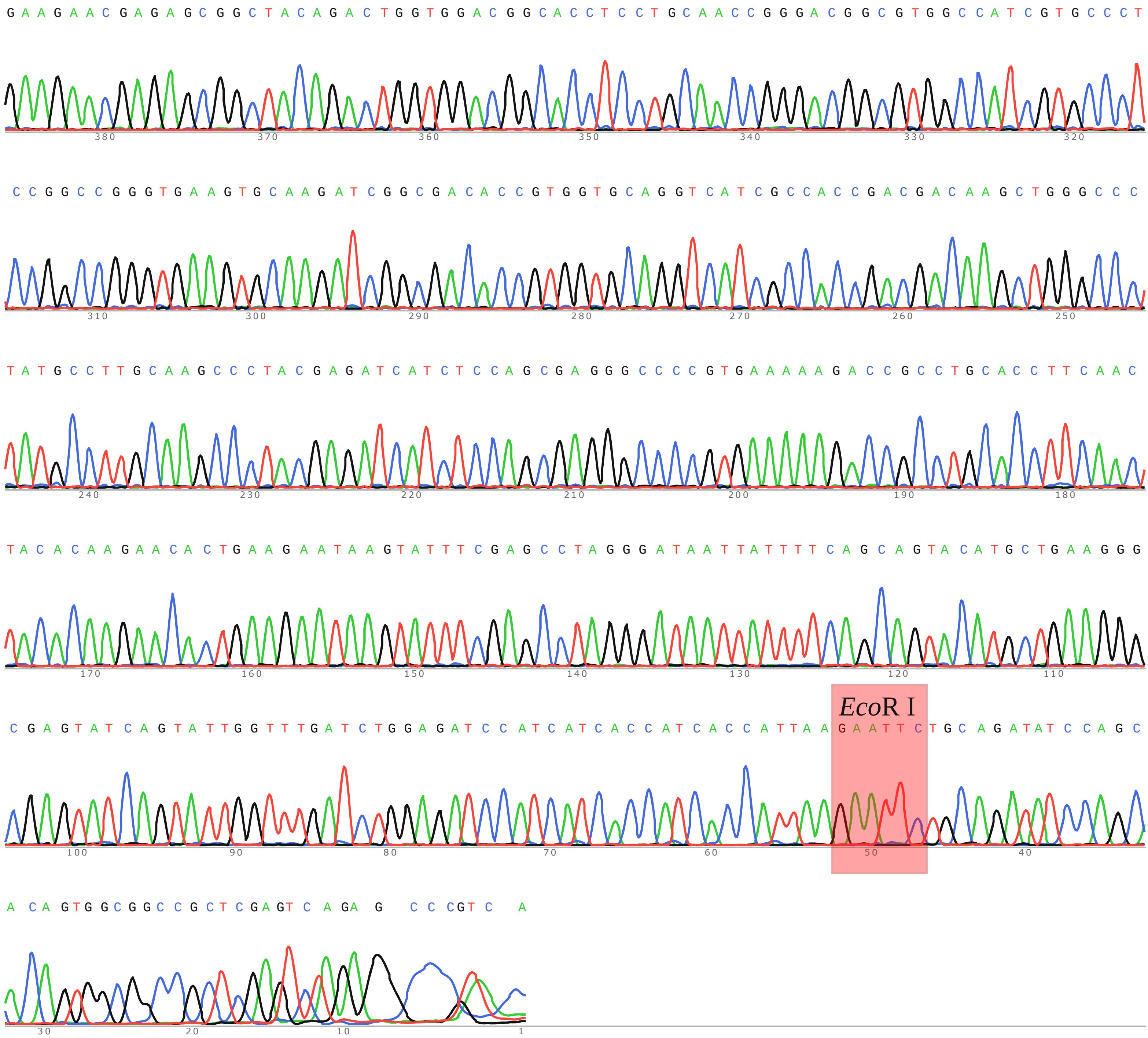
GAAGAACGAGAGCGGCTACAGACTGGTGGACGGCACCTCCTGCAACCGGGACGGCGTGGCCATCGTGCCCT
CCGGCCGGGTGAAGTGCAAGATCGGCGACACCGTGGTGCAGGTCATCGCCACCGACGACAAGCTGGGCCC
TATGCCTTGCAAGCCCTACGAGATCATCTCCAGCGAGGGCCCCGTGAAAAAGACCGCCTGCACCTTCAAC
TACACAAGAACACTGAAGAATAAGTATTTCGAGCCTAGGGATAATTATTTTCAGCAGTACATGCTGAAGGG
CGAGTATCAGTATTGGTTTGATCTGGAGATCCATCATCACCATCACCATTAAGAATTCTGCAGATATCCAGC
*Eco*R I
ACAGTGGCGGCCGCTCGAGTCAGAGCCCGTCA

MEP-049-pcDNA3.1-CSFV-CN033-E2_pRLuc-MCS-F_2024-05-10_C02.ab1 (1000 bases)

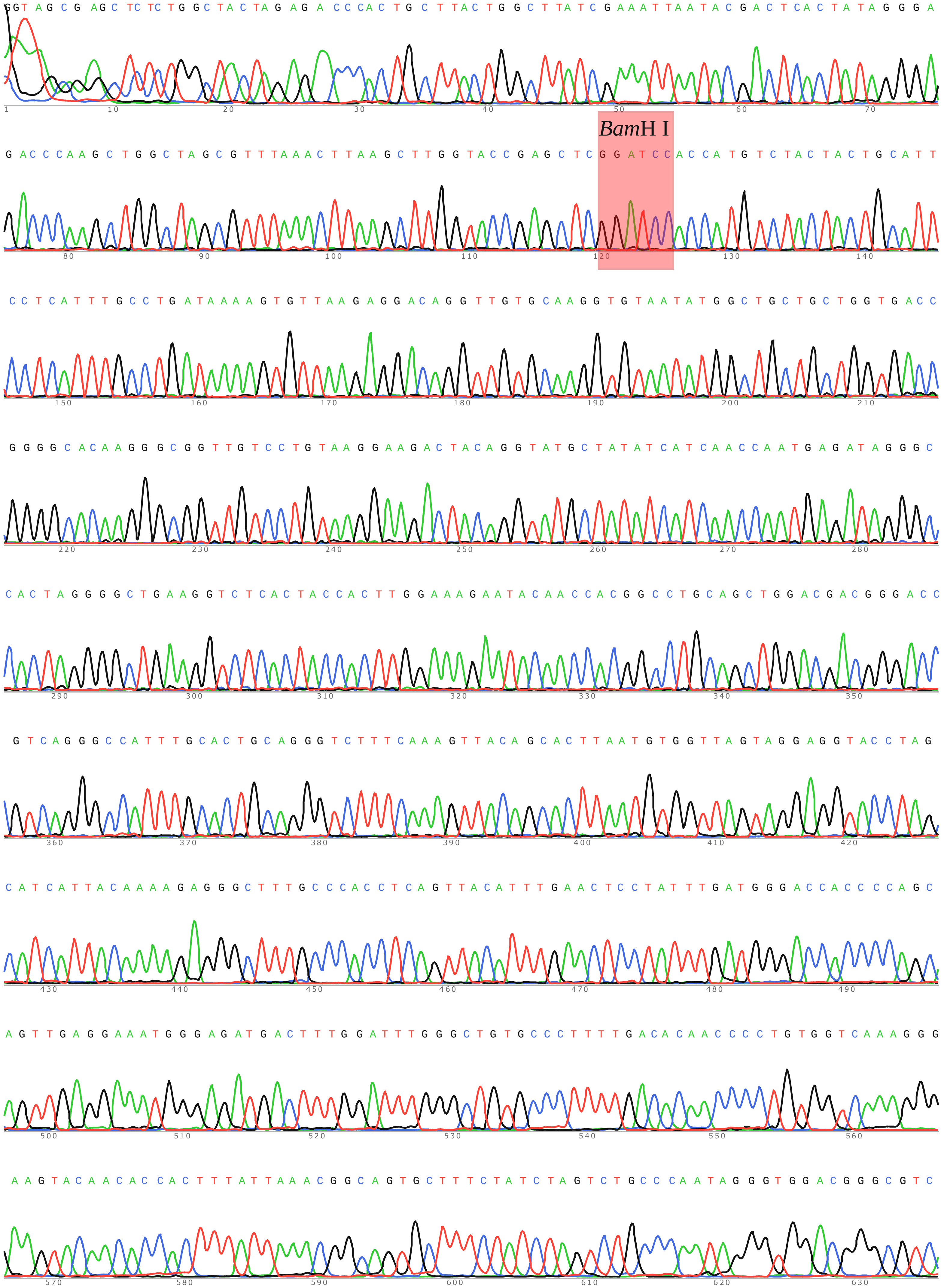

A T A G A G T G C A C G G C A G T G A G C C C C A C T A C C T T G A G A A C A G A A G T G G T A A A A A C C T T T A A G A G A G A A G C

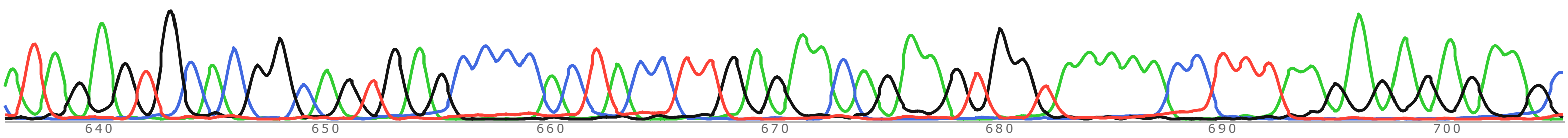

C C T T C C C G C A C A G A G T G G A T T G C G T G A C C A C T A T A G T A G A A A A A G A G G A C C T G T T C T A C T G C A A G T T G G

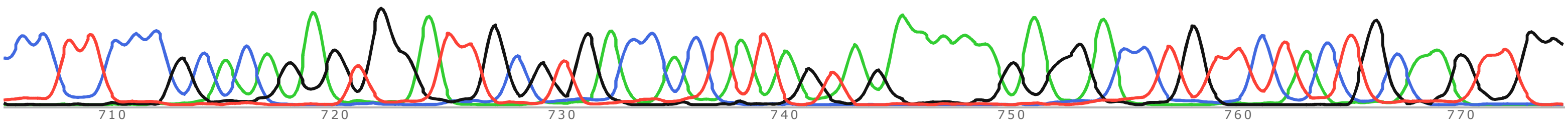

G G G G C A A T T G G A C A T G T G T G A A A G G C A A C C C G G T G A C C T A C A C A G G A G G G C A A G T A A A G C A A T G C A G G

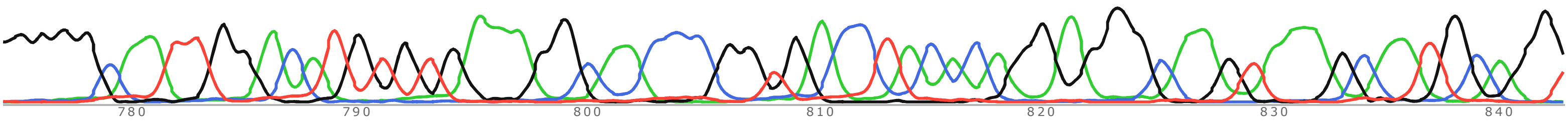

T G G T G C G G G T T T G A C T T C A A A G A A C C C G A T G G G C T C C C G C A C T A C C C C A T A G G C A A G T G C A T C C T A G T

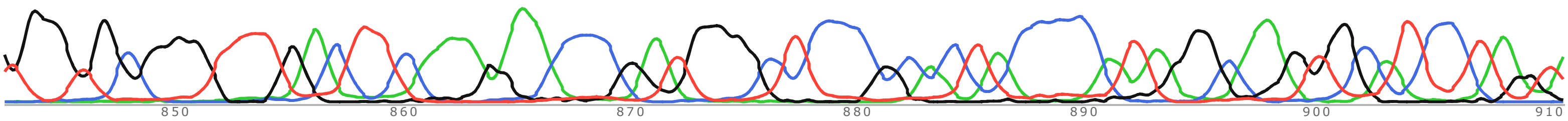

A A A T G A G A C G G G T T A C A G G G T G G T G G A T T C C A C A G A C T G C A A C A G A G A T G G T G T T G T A T C A G T A C T

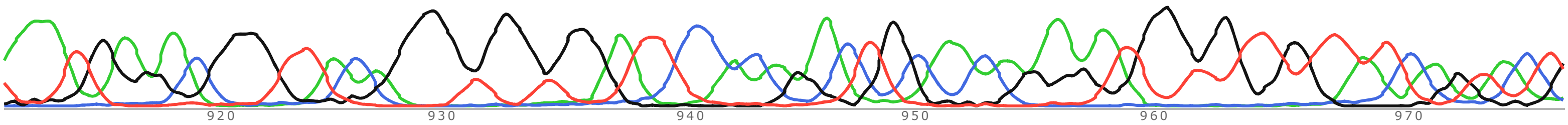

G A G A G A C A T G A G T G C T T G A T C G G C

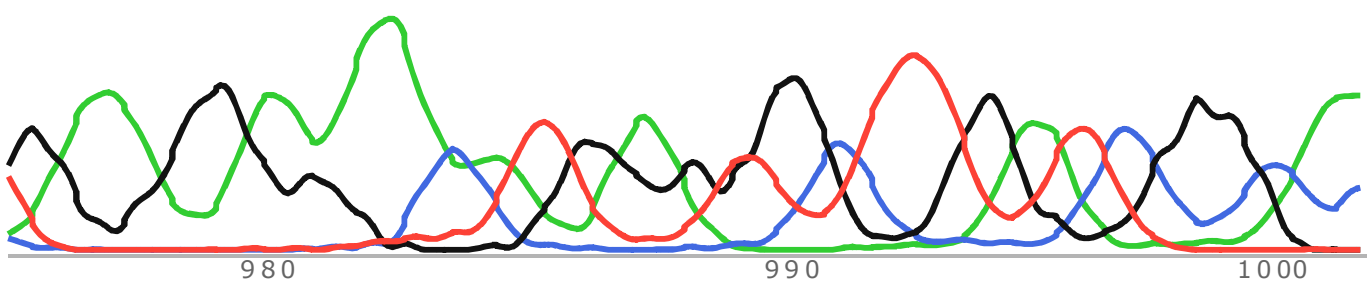

CTGAAGGTCTCACTACCACTTGGAAAGAATACAATCACGGCCTGCAGCTGGACGACGGGACCGTC

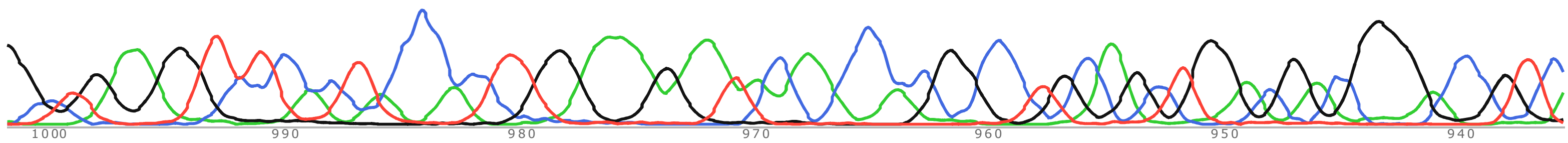

AGGGCCCATTTGCACTGCAGGGTCTTTCAAAGTTACAGCACTTAATGTGGTTAGTAGGAGGTACCTAGC

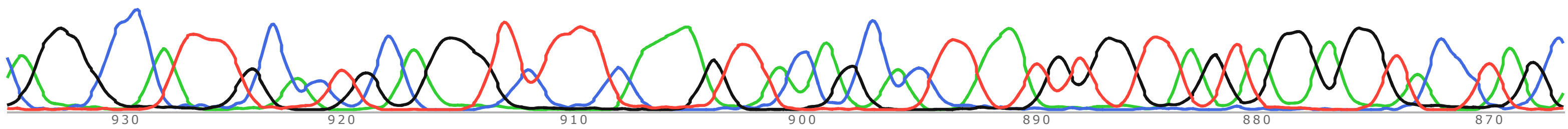

ATCATTACAAAAGAGGGCTTTGCCCACCTCAGTTACATTTGAACTCCTATTTGATGGGACCACCCCAG

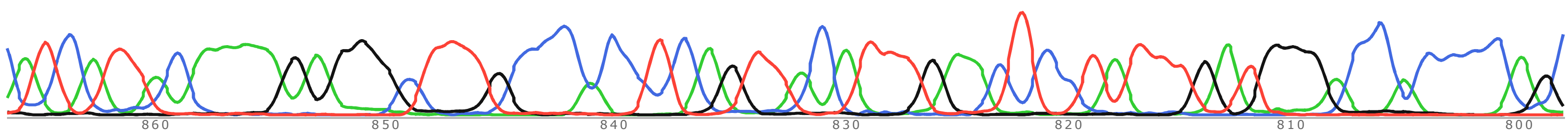

CAGTTGAGGAAATGGGAGATGACTTTGGATTTGGGCTGTGCCCTTTTGACACAACCCCTGTGGTCAAAGG

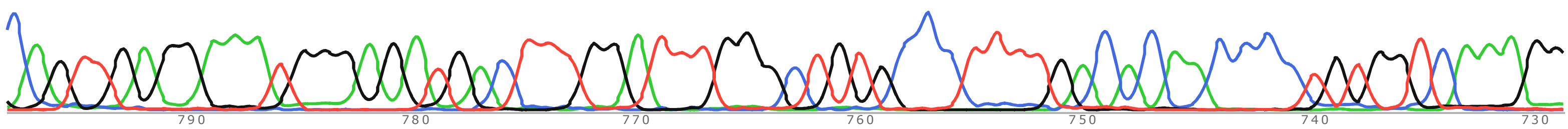

GAAGTACAACACCACTTTATTAAACGGCAGTGCTTTCTATCTAGTCTGCCCAATAGGGTGGACGGGCGT

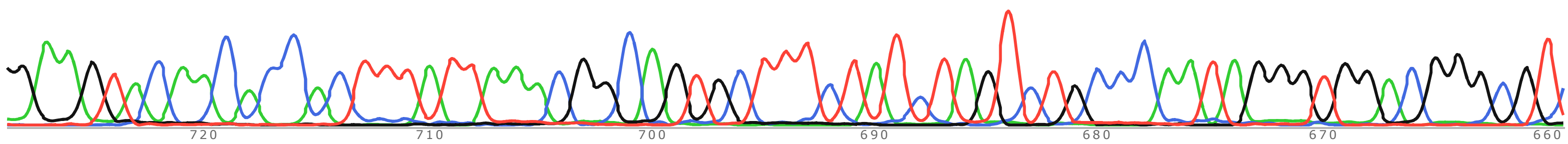

CATAGAGTGCACGGCAGTGAGCCCCACTACCTTGAGAACAGAAGTGGTAAAAACCTTTAAGAGAGAGAAG

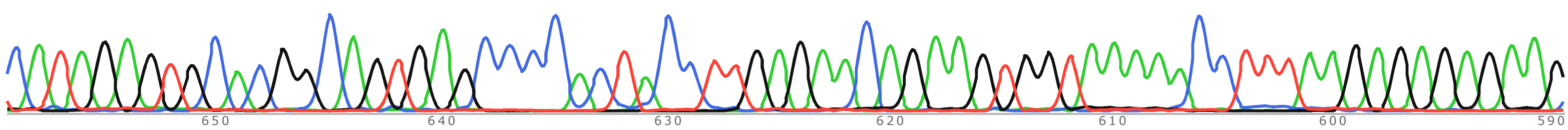

CCCTTCCCGCACAGAGTGGATTGCGTGACCACTATAGTAGAAAAAGAGGACCTGTTCTACTGCAAGTTG

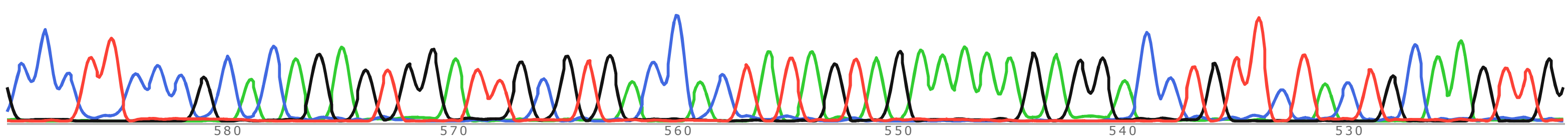

GGGGGCAATTGGACATGTGTGAAAGGCAACCCGGTGACCTACACAGGAGGGCAAGTAAAGCAATGCAGGT

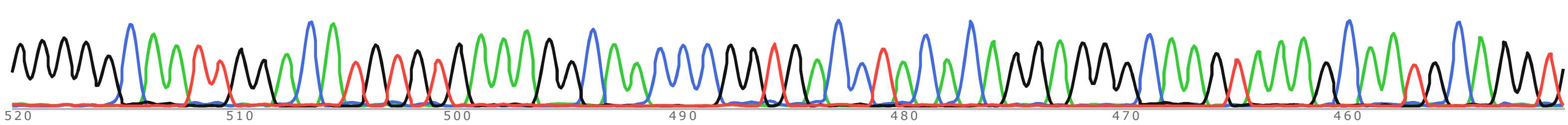

GGTGCGGGTTTGACTTCAAAGAACCCGATGGGCTCCCGCACTACCCCATAGGCAAGTGCATCCTAGTAAA

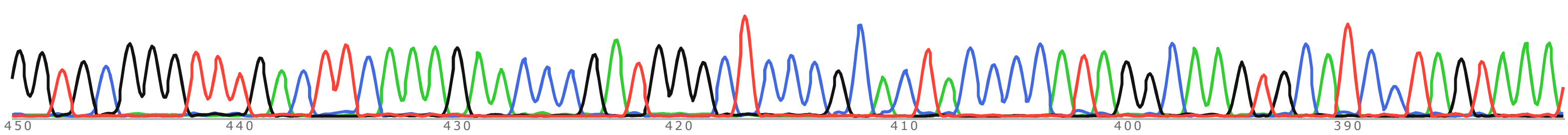

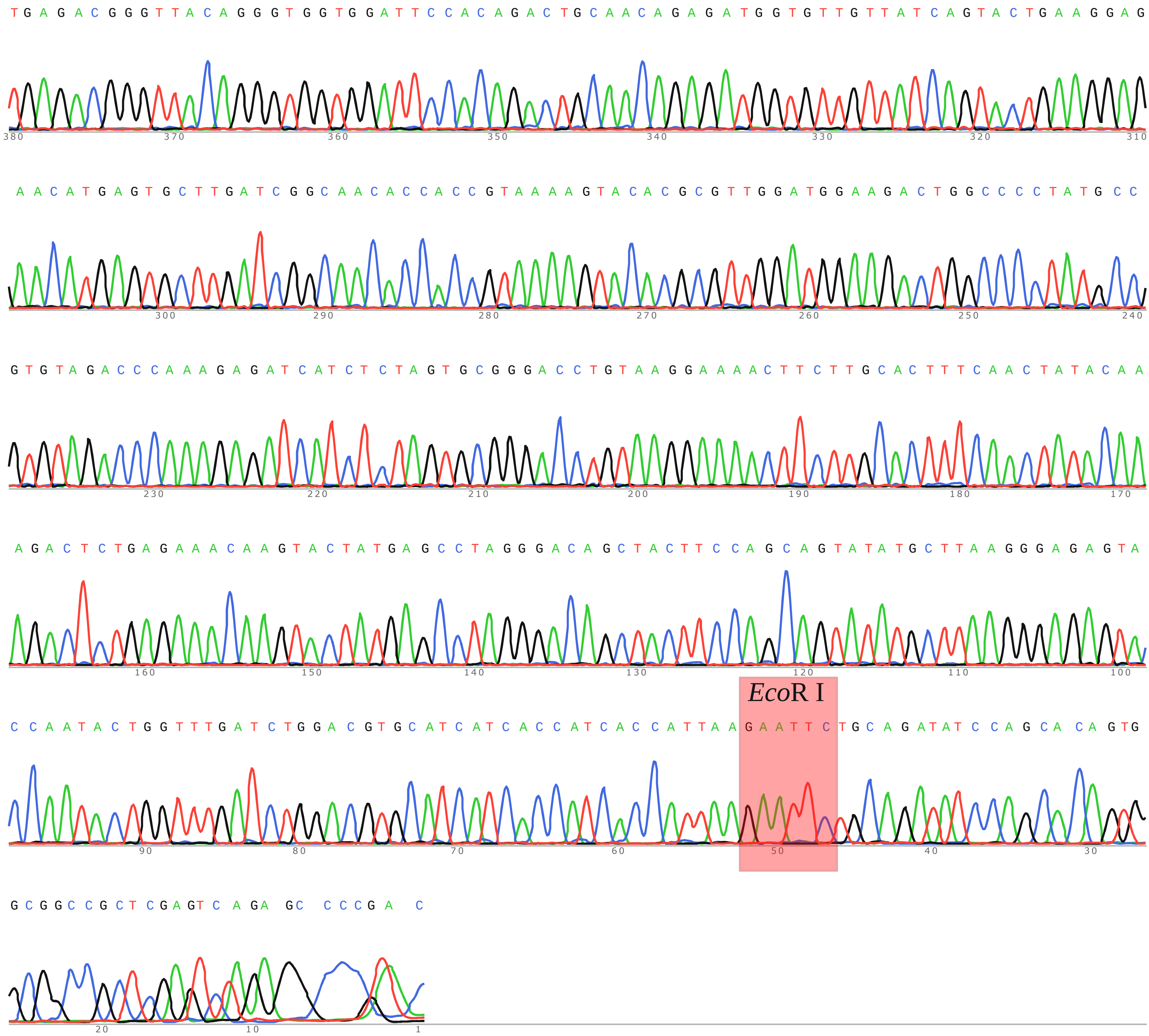
TGAGACGGGTTACAGGGTGGTGGATTCCACAGACTGCAACAGAGATGGTGTTGTTATCAGTACTGAAGGAG
AACATGAGTGCTTGATCGGCAACACCACCGTAAAAGTACACGCGTTGGATGGAAGACTGGCCCCTATGCC
GTGTAGACCCAAAGAGATCATCTCTAGTGCGGGACCTGTAAGGAAAACTTCTTGCACTTTCAACTATACAA
AGACTCTGAGAAACAAGTACTATGAGCCTAGGGACAGCTACTTCCAGCAGTATATGCTTAAGGGAGAGTA
EcoR I
CCAATACTGGTTTGATCTGGACGTGCATCATCACCATCACCATTAAGAATTCTGCAGATATCCAGCACAGTG
GCGGCCGCTCGAGTCAGAGCCCGAC

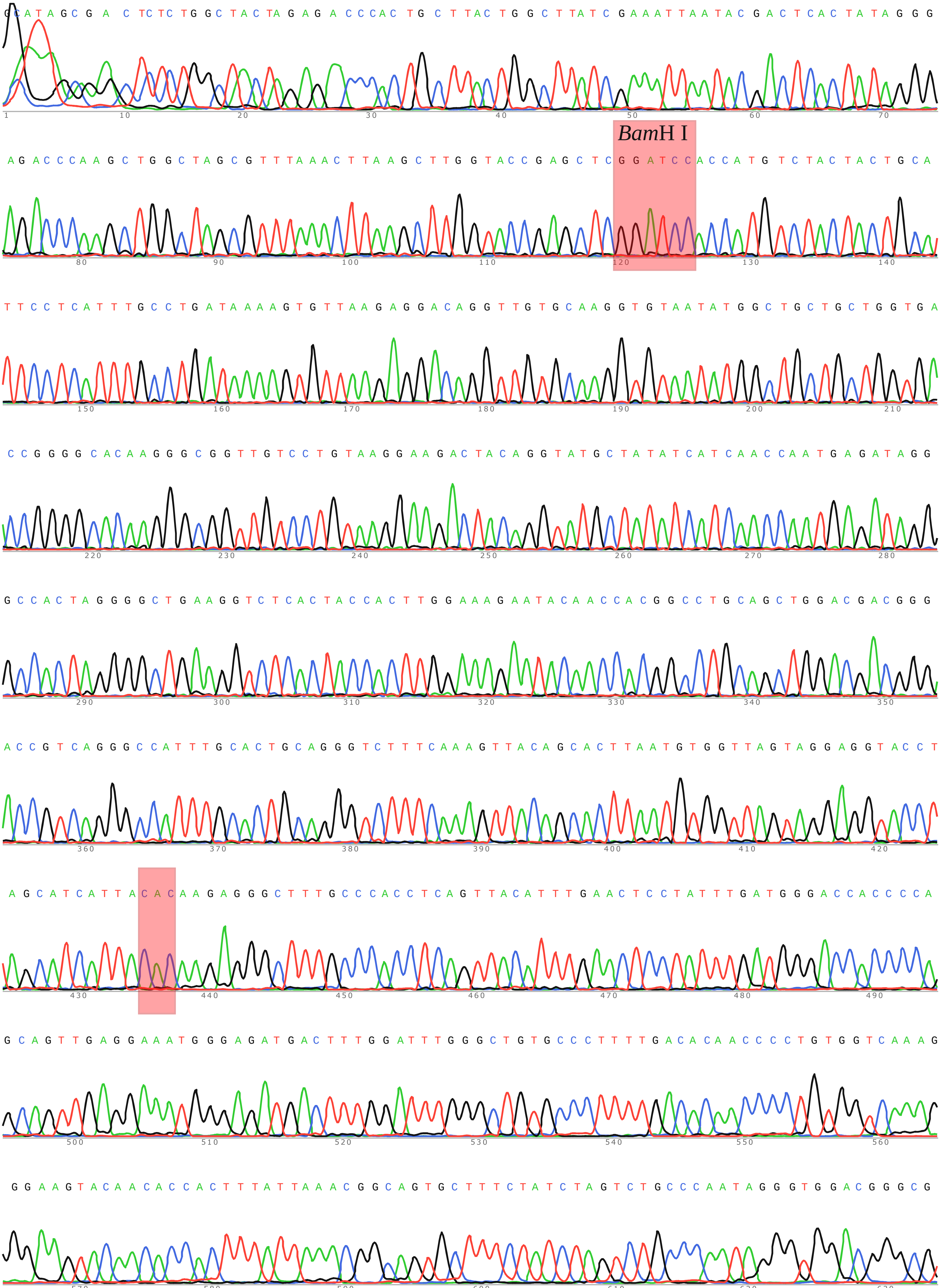
GCATAGCGACTCTCTGGCTACTAGAGACCCACTGCTTACTGGCTTATCGAAATTAATACGACTCACTATAGGG
BamH I
AGACCCAAGCTGGCTAGCGTTTAAACTTAAGCTTGGTACCGAGCTCGGATCCACCATGTCTACTACTGCA
TTCCTCATTTGCCTGATAAAAGTGTTAAGAGGACAGGTTGTGCAAGGTGTAATATGGCTGCTGCTGGTGA
CCGGGGCACAAGGGCGGTTGTCCTGTAAGGAAGACTACAGGTATGCTATATCATCAACCAATGAGATAGG
GCCACTAGGGGCTGAAGGTCTCACTACCACTTGGAAAGAATACAACCACGGCCTGCAGCTGGACGACGGG
ACCGTCAGGGCCATTTGCACTGCAGGGTCTTTCAAAGTTACAGCACTTAATGTGGTTAGTAGGAGGTACCT
AGCATCATTACACAAGAGGGCTTTGCCCACCTCAGTTACATTTGAACTCCTATTTGATGGGACCACCCA
GCAGTTGAGGAAATGGGAGATGACTTTGGATTTGGGCTGTGCCCTTTTGACACAACCCCTGTGGTCAAAG
GGAAGTACAACACCACTTTATTAAACGGCAGTGCTTTCTATCTAGTCTGCCCAATAGGGTGGACGGGCG

TCATAGAGTGCACGGCAGTGAGCCCCACTACCTTGAGAACAGAAGTGGTAAAAACCTTTAAGAGAGAGA

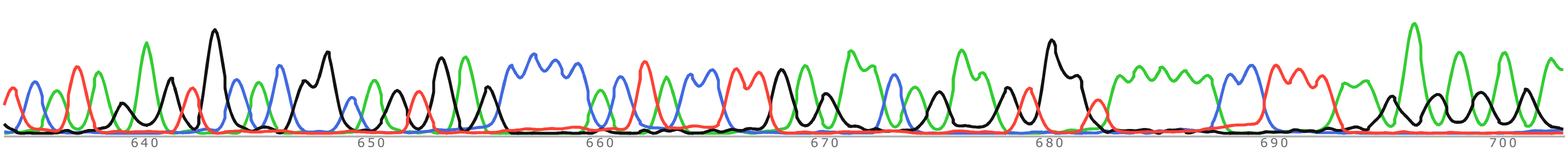

AGCCCTTCCCGCACAGAGTGGATTGCGTGACCACTATAGTAGAAAAAGAGGACCTGTTCTACTGCAAGT

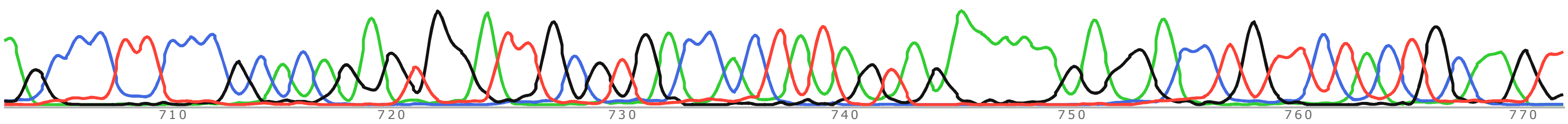

TGGGGGCAATTGGACATGTGTGAAAGGCAACCCGGTGACCTACACAGGAGGGCAAGTAAAGCAATGCA

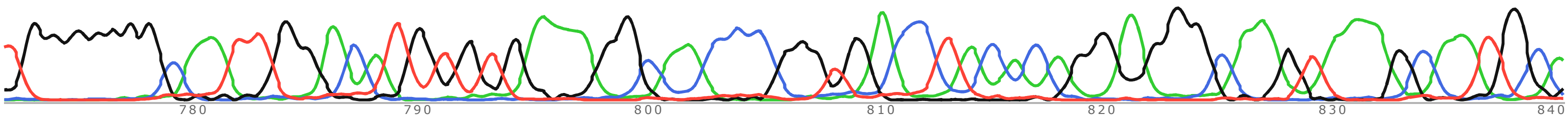

CGTGGTGCGGGTTTGACTTCAAAGAACCCGATGGGCTCCCGCACTACCCCATAGGCAAGTGCATCCT

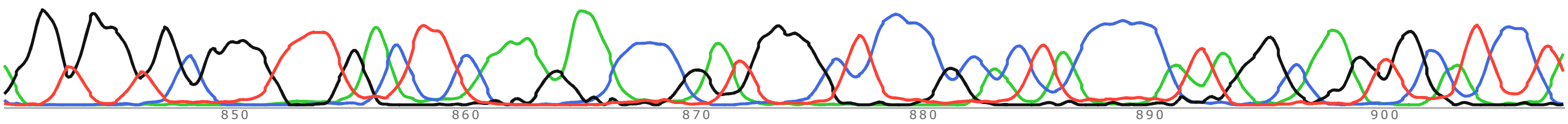

AGTAAATGAGACGGGTTACAGGGTGGTGGATTCCACAGACTGCAACAGAGATGGTGTTGTTATCAG

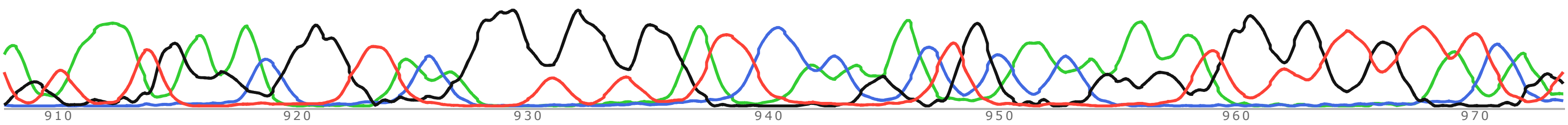

TACTGAAGGAGACATGAGTGCTTGATC

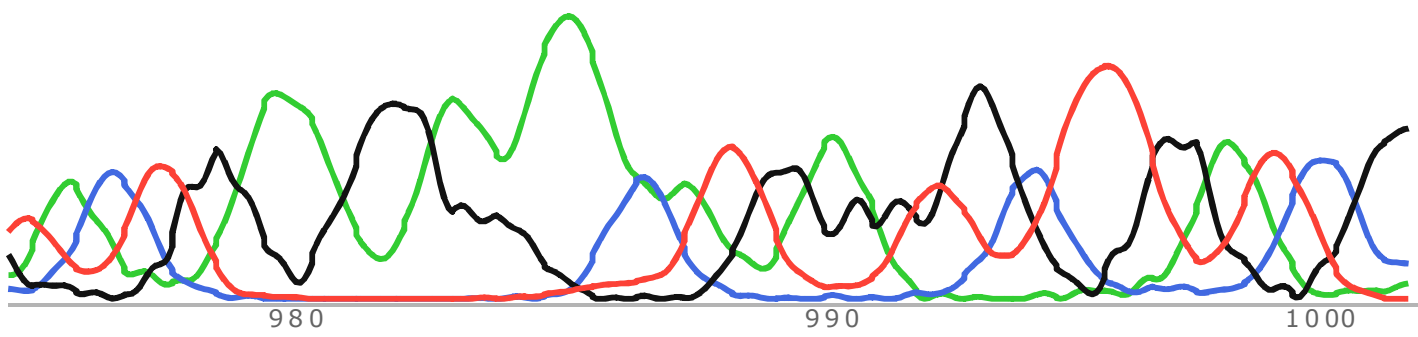

MEP-050-pcDNA3.1-CSFV-CN033-E2 (Q70H)_BGH-r_2024-05-10_E03.ab1 (1000 bases)

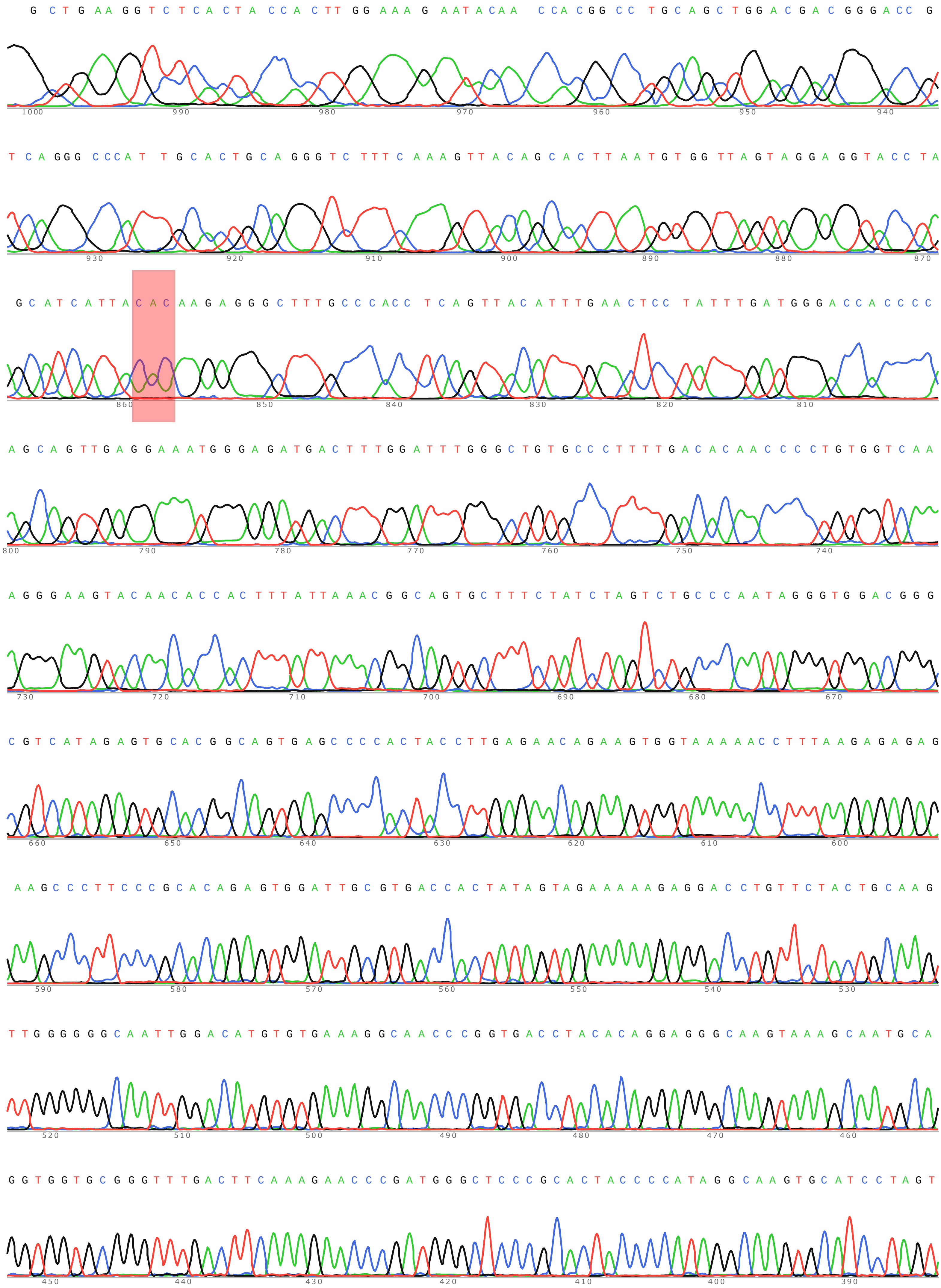

A A A T G A G A C G G G T T A C A G G G T G G T G G A T T C C A C A G A C T G C A A C A G A G A T G G T G T T G T T A T C A G T A C T G A A G

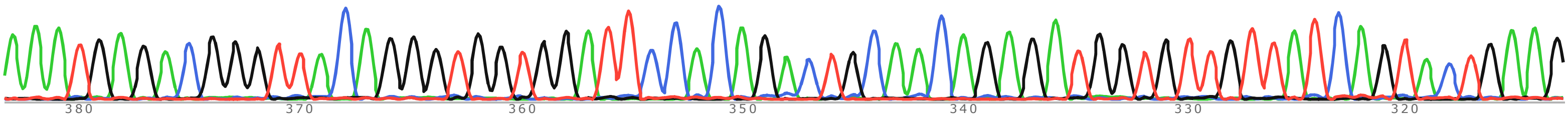

G A G A A C A T G A G T G C T T G A T C G G C A A C A C C A C C G T A A A A G T A C A C G C G T T G G A T G G A A G A C T G G C C C C T A T

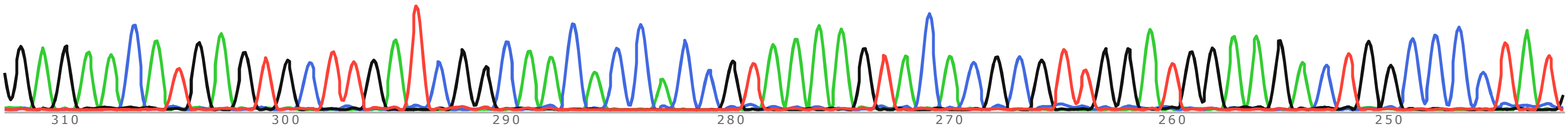

G C C G T G T A G A C C C A A A G A G A T C A T C T C T A G T G C G G G A C C T G T A A G G A A A A C T T C T T G C A C T T T C A A C T A T A

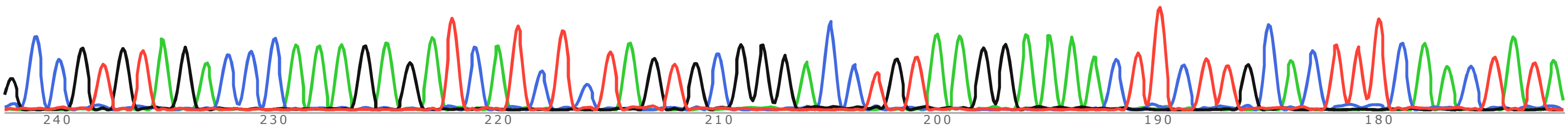

C A A A G A C T C T G A G A A A C A A G T A C T A T G A G C C T A G G G A C A G C T A C T T C C A G C A G T A T A T G C T T A A G G G A G A

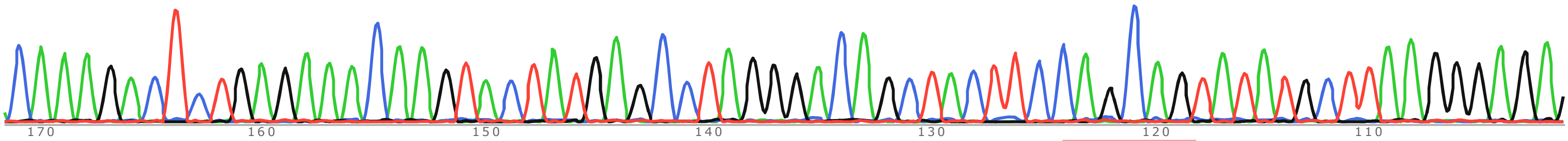

G T A C C A A T A C T G G T T T G A T C T G G A C G T G C A T C A T C A C C A T C A C C A T T A A G A A T T C T G C A G A T A T C C A G C A C A

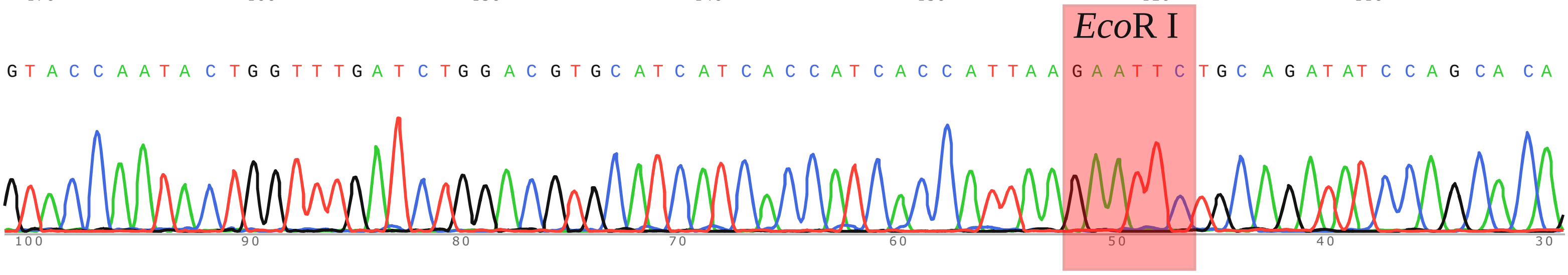

G T G G C G G C C G C T C G A G T C A G A G A C C C G A C

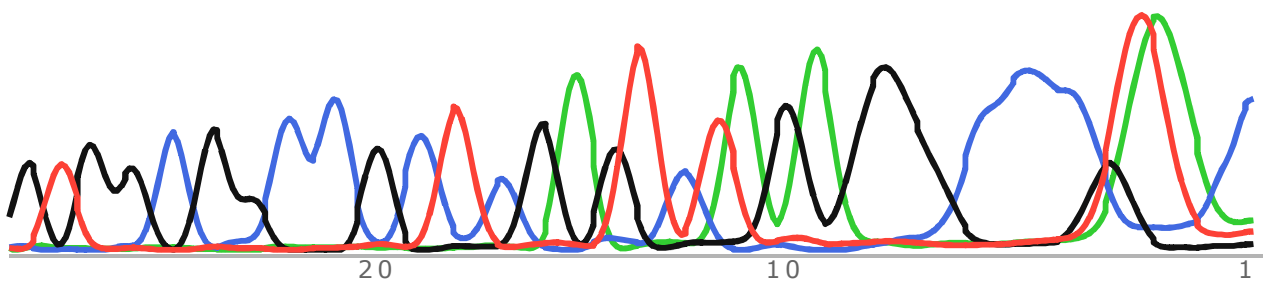

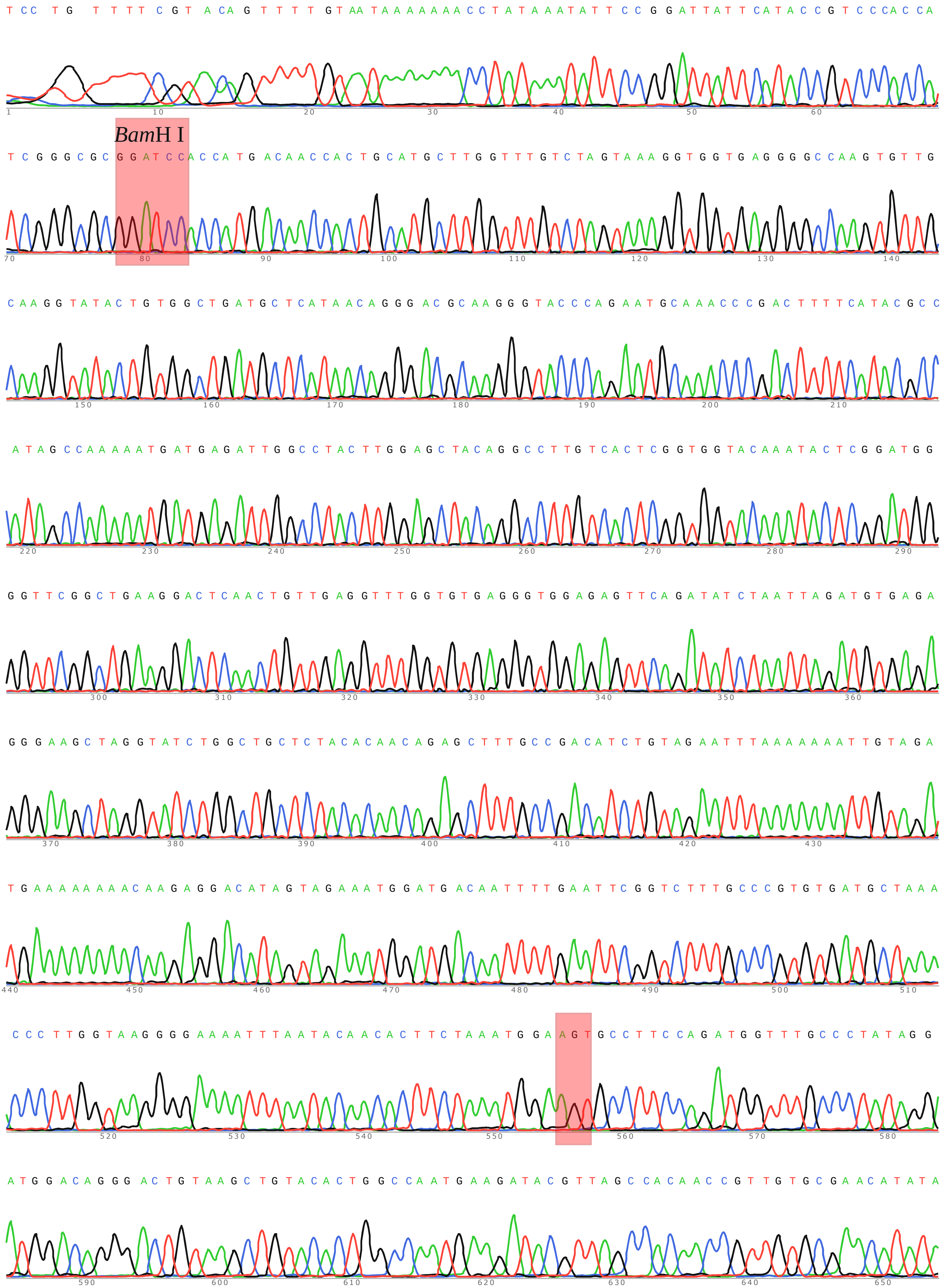
TCC TG TTTT CGT ACA GTTTT GTAA TAAAAAAACCTATAAATATTCCGGATTATTCATACCGTCCCACCA
BamH I
TCGGGCGCGGATCCACCATGACAACCACTGCATGCTTGGTTTGTCTAGTAAAGGTGGTGAGGGGCCAAGTGTTG
CAAGGTATACTGTGGCTGATGCTCATAACAGGGACGCAAGGGTACCCAGAATGCAAACCCGACTTTTCATACGCC
ATAGCCAAAAATGATGAGATTGGCCTACTTGGAGCTACAGGCCTTGTCACTCGGTGGTACAAATACTCGGATGG
GGTTCGGCTGAAGGACTCAACTGTTGAGGTTTGGTGTGAGGGTGGAGAGTTCAGATATCTAATTAGATGTGAGA
GGGAAGCTAGGTATCTGGCTGCTCTACACAACAGAGCTTTGCCGACATCTGTAGAATTTAAAAAAATTGTAGA
TGAAAAAAAACAAGAGGACATAGTAGAAATGGATGACAATTTTGAATTCGGTCTTTGCCCGTGTGATGCTAAA
CCCTTGGTAAGGGGAAAATTTAATACAACACTTCTAAATGGAAGTGCCTTCCAGATGGTTTGCCCTATAGG
ATGGACAGGGACTGTAAGCTGTACACTGGCCAATGAAGATACGTTAGCCACAACCGTTGTGCGAACATATA

A G A G G G T C A G G C C T T T T C C A T A T A G G C A G G A C T G T G T C A C A C A G A A A A C C A T T G G G G A A G A C C T C T A T G

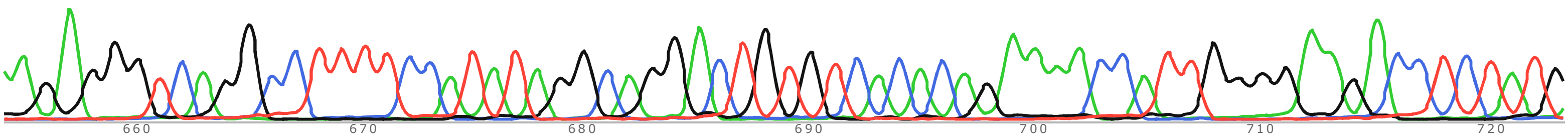

A C T G T G C C C T A G G A G G G A A T T G G A C C T G T G T G T T G G G G G G A T A T A C T A C G A T A T G T A G C T G G G C C T G T

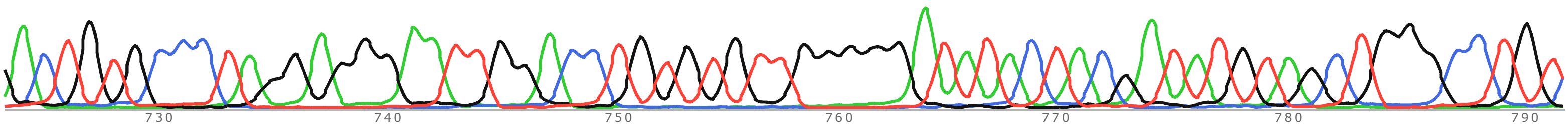

C G A G T C T T G T A A G T G G T G T G G T C A C A A A T T T C T T A A A A G T G A G G G T C T G C C G C A T T T C C C A A T T G G

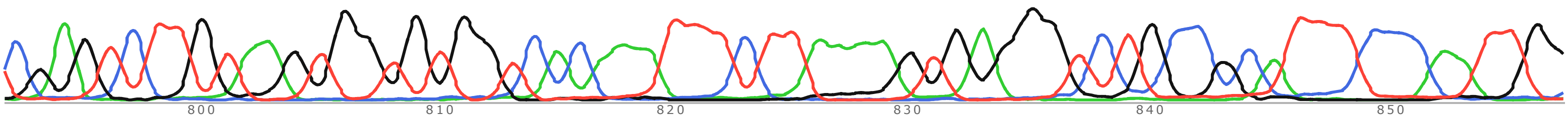

C A A A T G C A G G C T G A A G A A T G A G A G T G G C T A T A G A C T A G T G G A T G G G A C T T C T T G C A A C A G A G

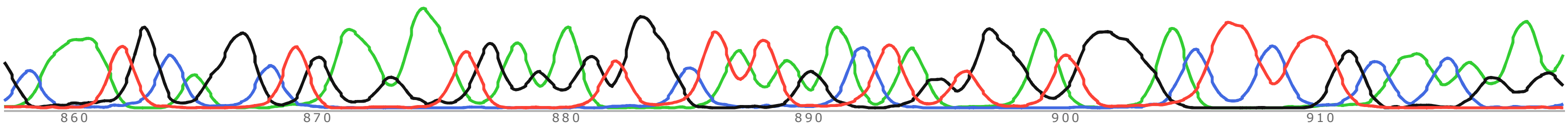

A C G G C G T G G C T A T A G T G C C A T C T G G G C C G G G T C A A A T G C A A A A T A G G A G A C A C A G T G G T G

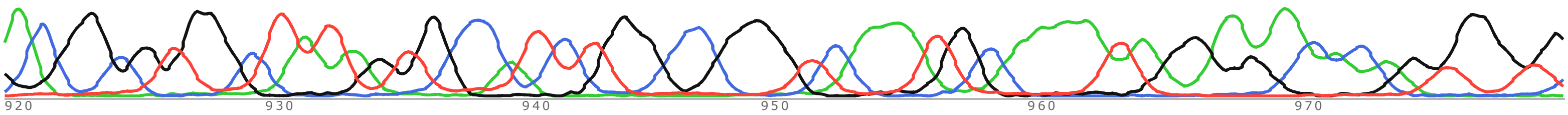

C A G G T C A T T G C A A C G G A T G A T A A A C T A G G C C

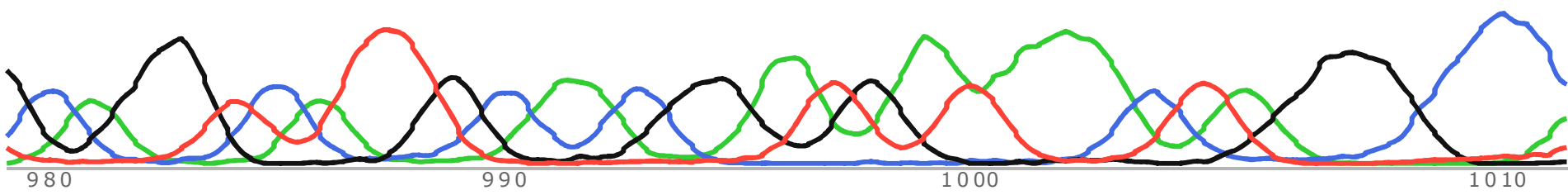

A T G T A G A T G A A A A A A A A C A G A G G A C A T A G T A G A A A T G G A T G A C A A T T T T G A A T T C G G T C T T

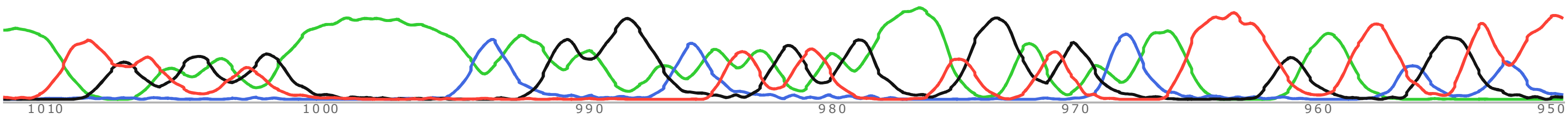

T G C C C G T G T G A T G C T A A A C C C T T G G T A A G G G G A A A A T T T A A T A C A A C A C T T C T A A A T G G A A G T G C C

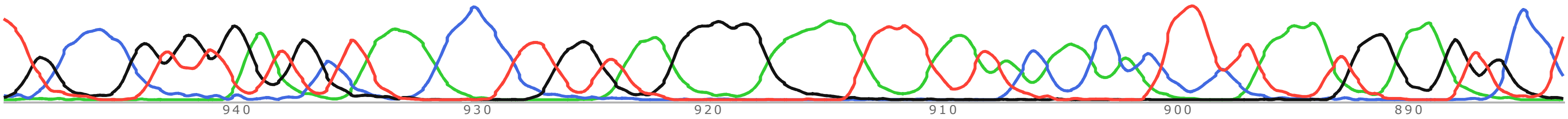

T T C C A G A T G G T T T G C C C T A T A G G A T G G A C A G G G A C T G T A A G C T G T A C A C T G G C C A A T G A A G A T A C G T T

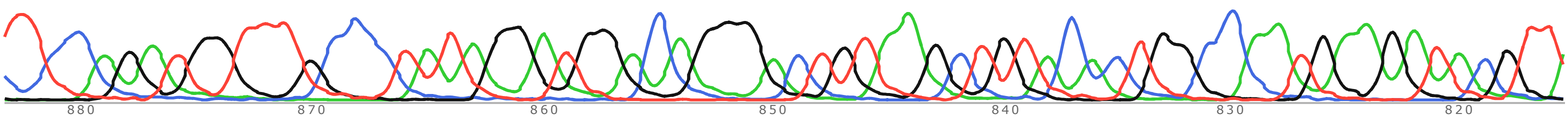

A G C C A C A A C C G T T G T G C G A A C A T A T A A G A G G G T C A G G C C T T T T C C A T A T A G G C A G G A C T G T G T C A C A C A

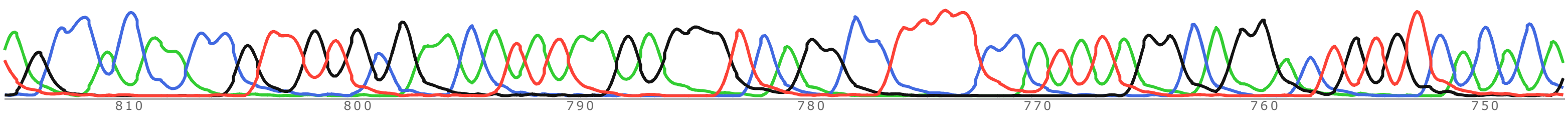

G A A A C C A T T G G G G A A G A C C T C T A T G A C T G T G C C C T A G G A G G G A A T T G G A C C T G T G T G T T G G G G A T A T A

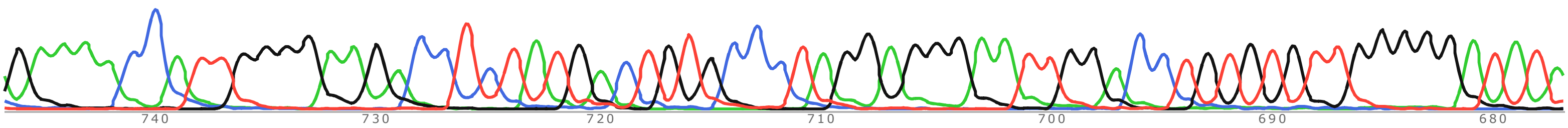

C T A C G A T A T G T A G C T G G G C C T G T C G A G T C T T G T A A G T G G T G T G G T C A C A A A T T T C T T A A A A G T G A G G G T

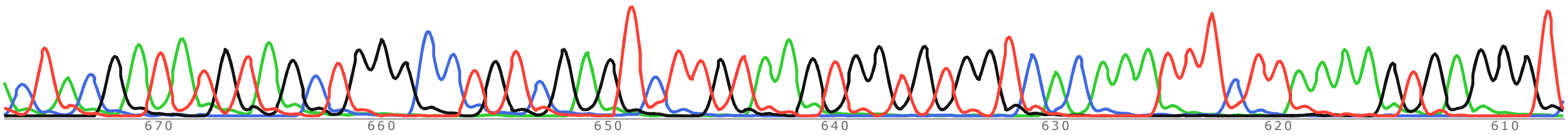

C T G C C G C A T T T C C C A A T T G G C A A A T G C A G G C T G A A G A A T G A G A G T G G C T A T A G A C T A G T G G A T G G G A C T T

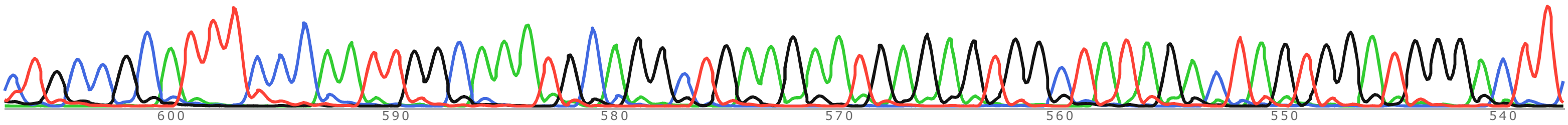

C T T G C A A C A G A G A C G G C G T G G C T A T A G T G C C A T C T G G C C G G G T C A A A T G C A A A A T A G G A G A C A C A G T G G T

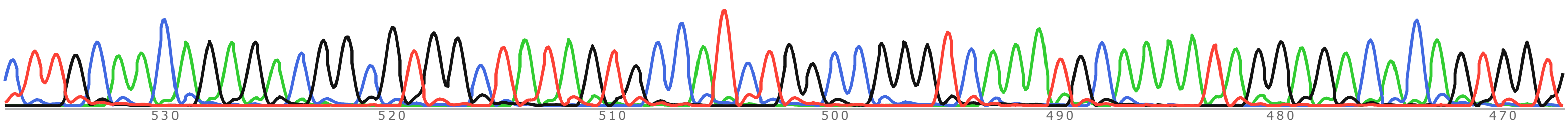

G C A G G T C A T T G C A A C G G A T G A T A A A C T A G G G C C C A T G C C T T G C A A A C C A T A T G A A A T C A T T T C C A G T G A G

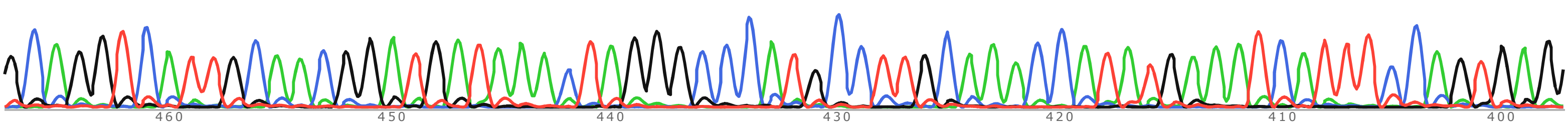

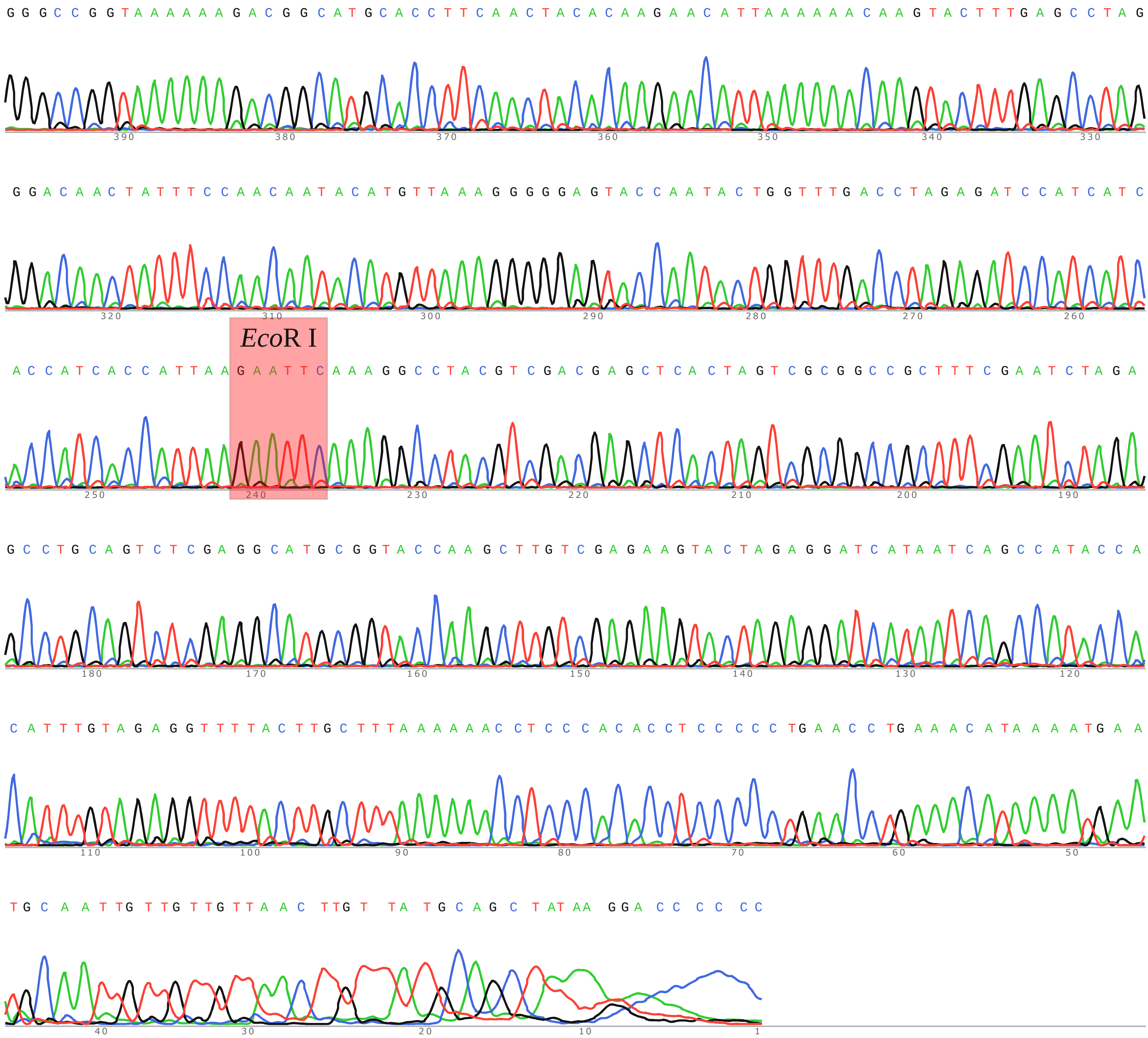
GGGCCGGTAAAAAAGACGGCATGCACCTTCAACTACACAAGAACATTAAAAAACAAGTACTTTGAGCCTAG
GGACAACTATTTCCAACAATACATGTTAAAGGGGGAGTACCAATACTGGTTTGACCTAGAGATCCATCATC
*Eco*R I
ACCATCACCATTAAGAATTCAAAGGCCTACGTCGACGAGCTCACTAGTCGCGGCCGCTTTCGAATCTAGA
GCCTGCAGTCTCGAGGCATGCGGTACCAAGCTTGTCGAGAAGTACTAGAGGATCATAATCAGCCATACCA
CATTTGTAGAGGTTTTACTTGCTTTAAAAACCTCCCACACCTCCCCCTGAACCTGAAACATAAAATGAA
TGCAATTGTTGTTGTTAACTTGTTATGCAGCTATAAGGACCCCCC

BEP-486-pFsat Bac1-BVDV-XH32-E2 (Q127Y)_poly-F_2023-07-04_E03.ab1 (1010 bases)

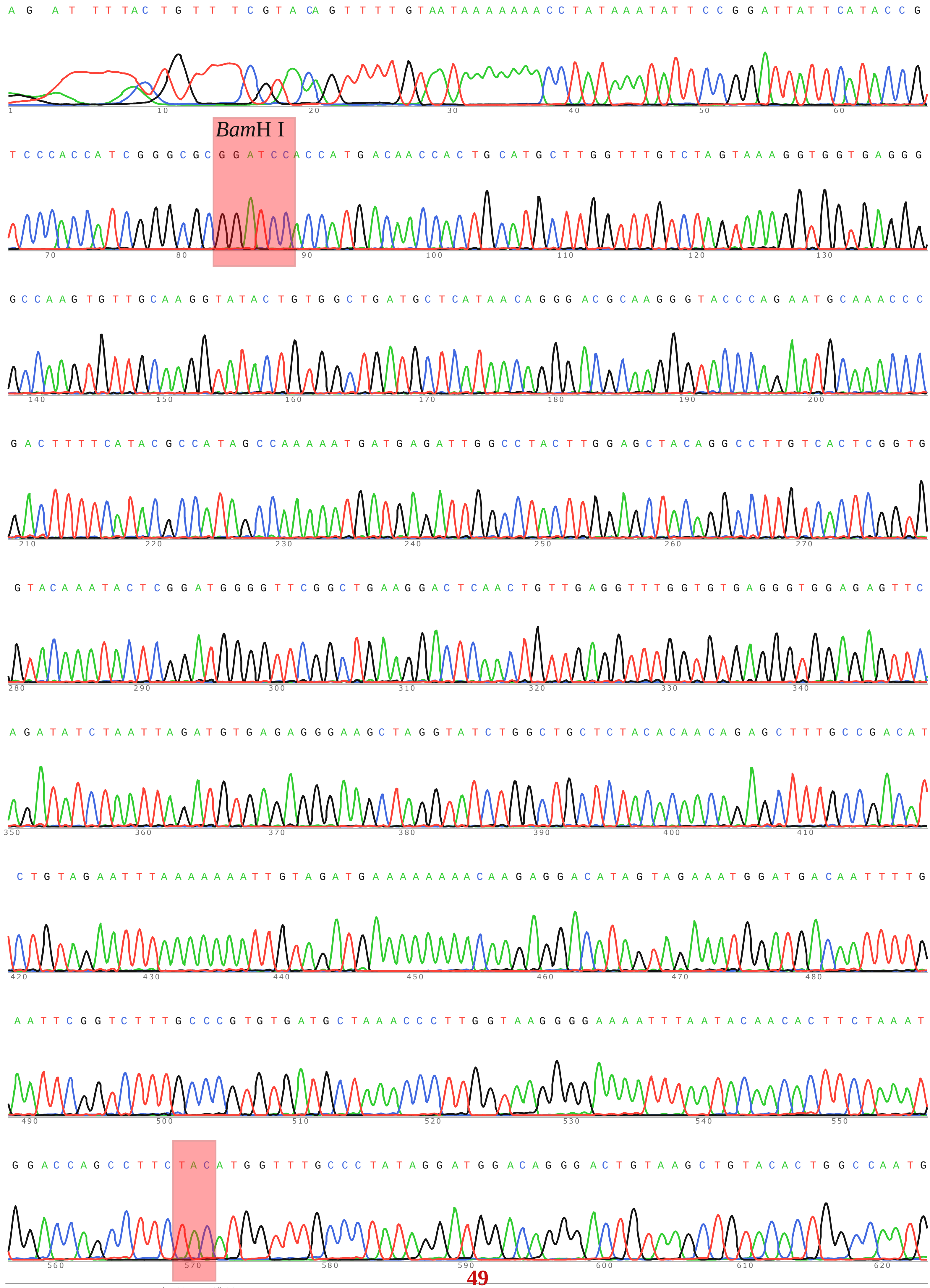

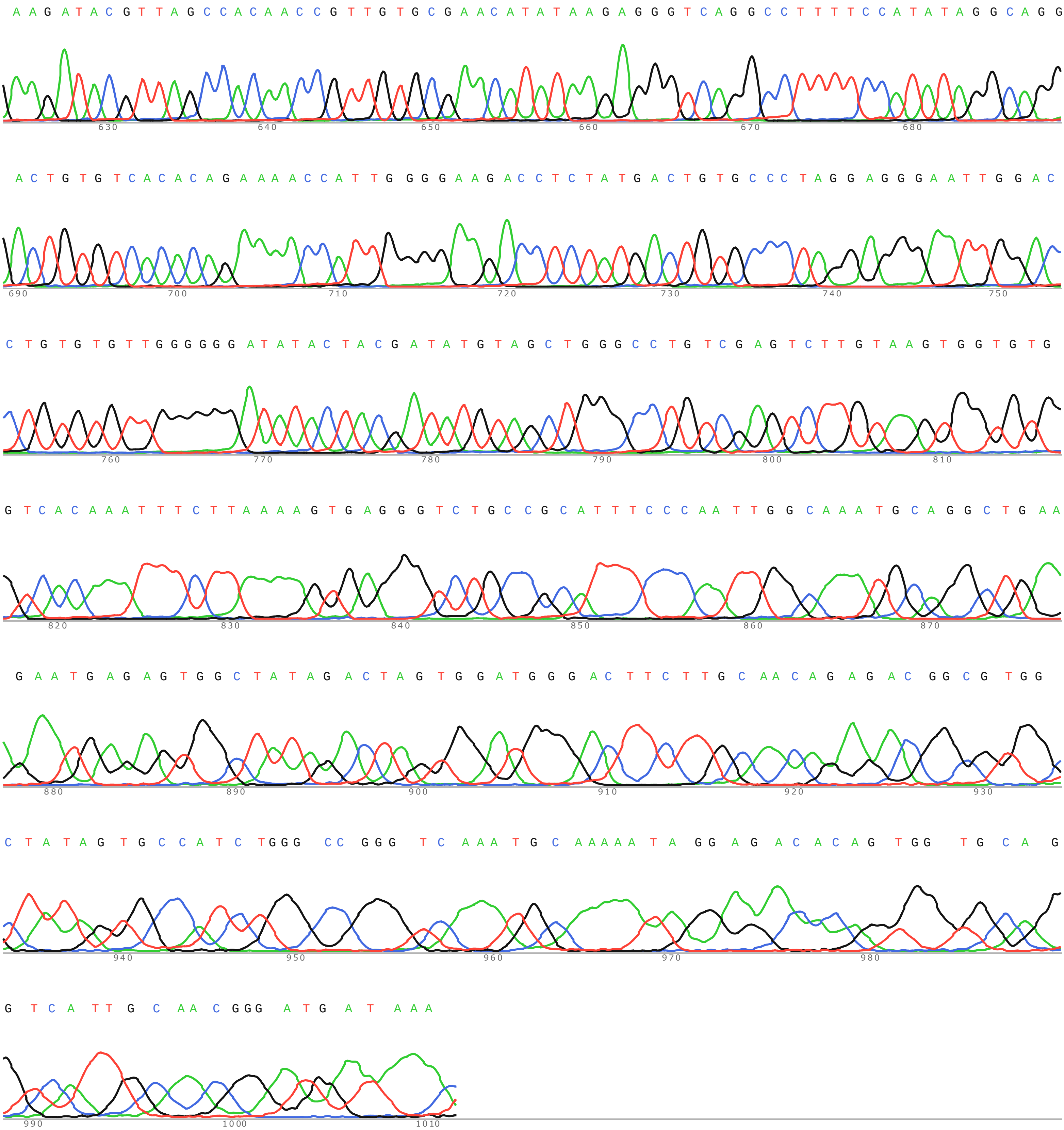
A A G A T A C G T T A G C C A C A A C C G T T G T G C G A A C A T A T A A G A G G G T C A G G C C T T T T C C A T A T A G G C A G G
630 640 650 660 670 680
A C T G T G T C A C A C A G A A A A C C A T T G G G G A A G A C C T C T A T G A C T G T G C C C T A G G A G G G A A T T G G A C
690 700 710 720 730 740 750
C T G T G T G T T G G G G G G A T A T A C T A C G A T A T G T A G C T G G G C C T G T C G A G T C T T G T A A G T G G T G T G
760 770 780 790 800 810
G T C A C A A A T T T C T T A A A A G T G A G G G T C T G C C G C A T T T C C C A A T T G G C A A A T G C A G G C T G A A
820 830 840 850 860 870
G A A T G A G A G T G G C T A T A G A C T A G T G G A T G G G A C T T C T T G C A A C A G A G A C G G C G T G G
880 890 900 910 920 930
C T A T A G T G C C A T C T G G G C C G G G T C A A A T G C A A A A A T A G G A G A C A C A G T G G T G C A G
940 950 960 970 980
G T C A T T G C A A C G G G A T G A T A A A
990 1000 1010

BEP-486-pFsat Bac1-BVDV-XH32-E2 (Q127Y)_SV40pA-R_2023-07-04_E04.ab1 (1010 bases)

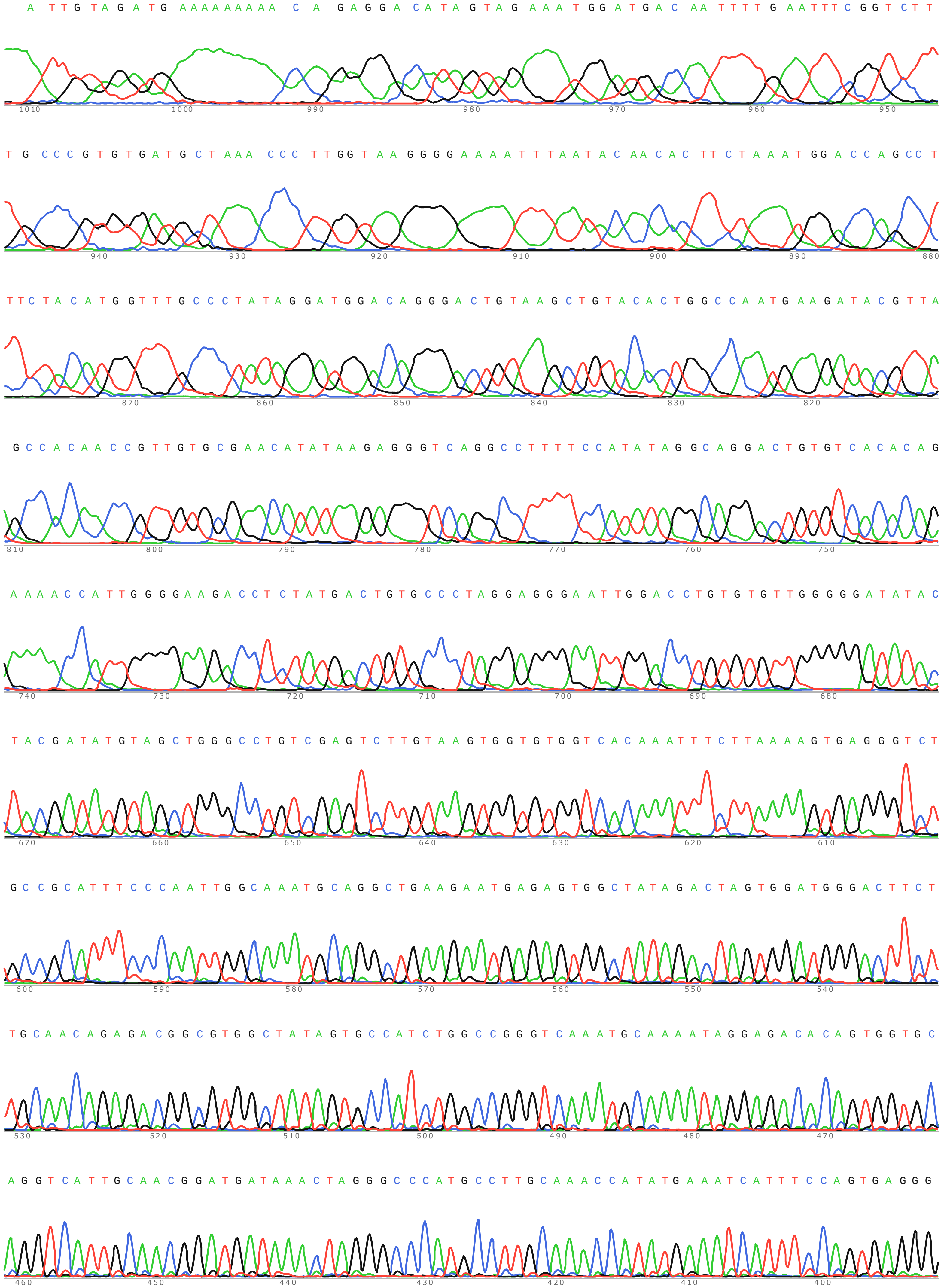

G C C G G T A A A A A A G A C G G C A T G C A C C T T C A A C T A C A C A A G A A C A T T A A A A A A C A A G T A C T T T G A G C C T A G G G

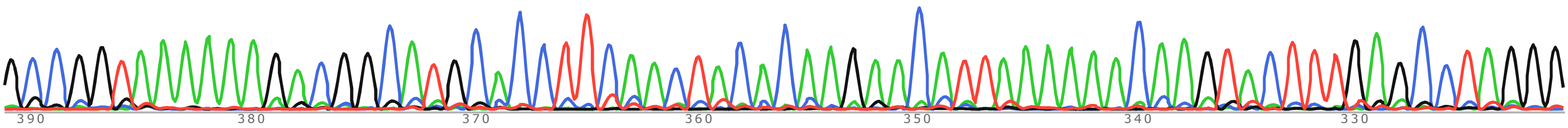

A C A A C T A T T T C C A A C A A T A C A T G T T A A A G G G G G A G T A C C A A T A C T G G T T T G A C C T A G A G A T C C A T C A T C A C

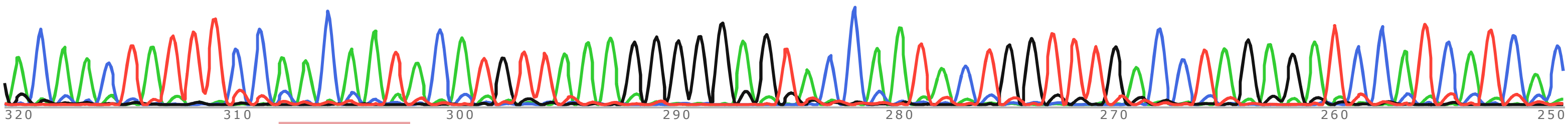

*Eco*R I

C A T C A C C A T T A A G A A T T C A A A G G C C T A C G T C G A C G A G C T C A C T A G T C G C G G C C G C T T T C G A A T C T A G A G C

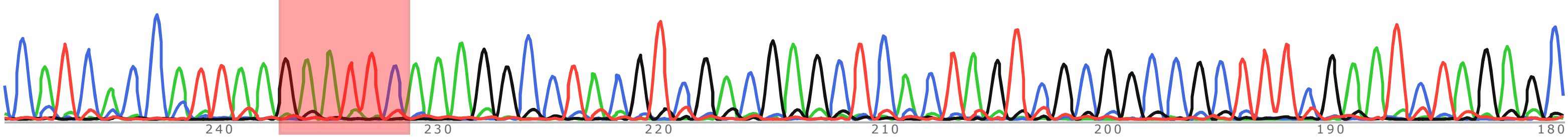

C T G C A G T C T C G A G G C A T G C G G T A C C A A G C T T G T C G A G A A G T A C T A G A G G A T C A T A A T C A G C C A T A C C A C

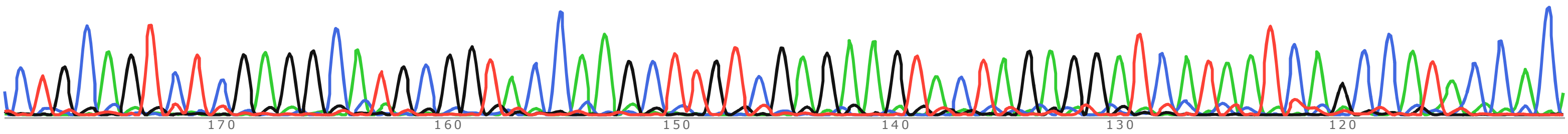

A T T T G T A G A G G T T T T A C T T G C T T T A A A A A C C T C C C A C A C C T C C C C C T G A A C C T G A A A C A T A A A A T G A A T

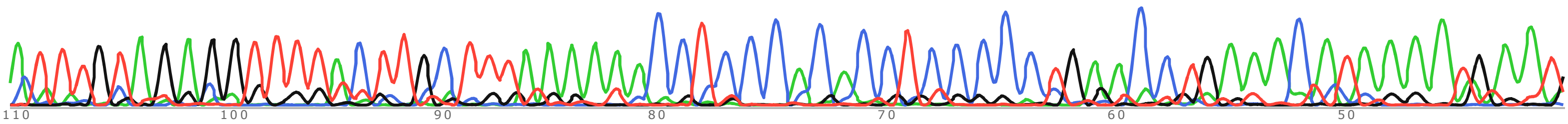

G C A A T T G T T G T T G T T A A C T G T T A T G C A G C T A T A A T T A A A C

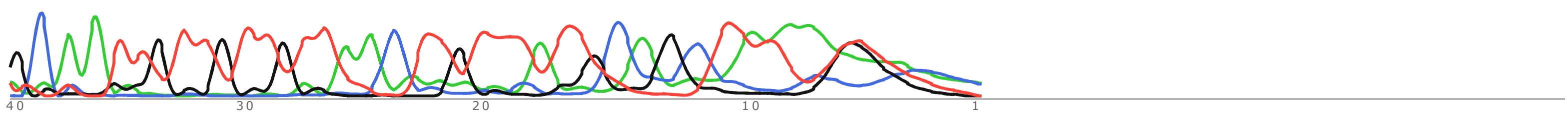

BEP-487-pFsat Bac1-BVDV-XH32-E2 (M128L)_poly-F_2023-07-04_G03.ab1 (1010 bases)

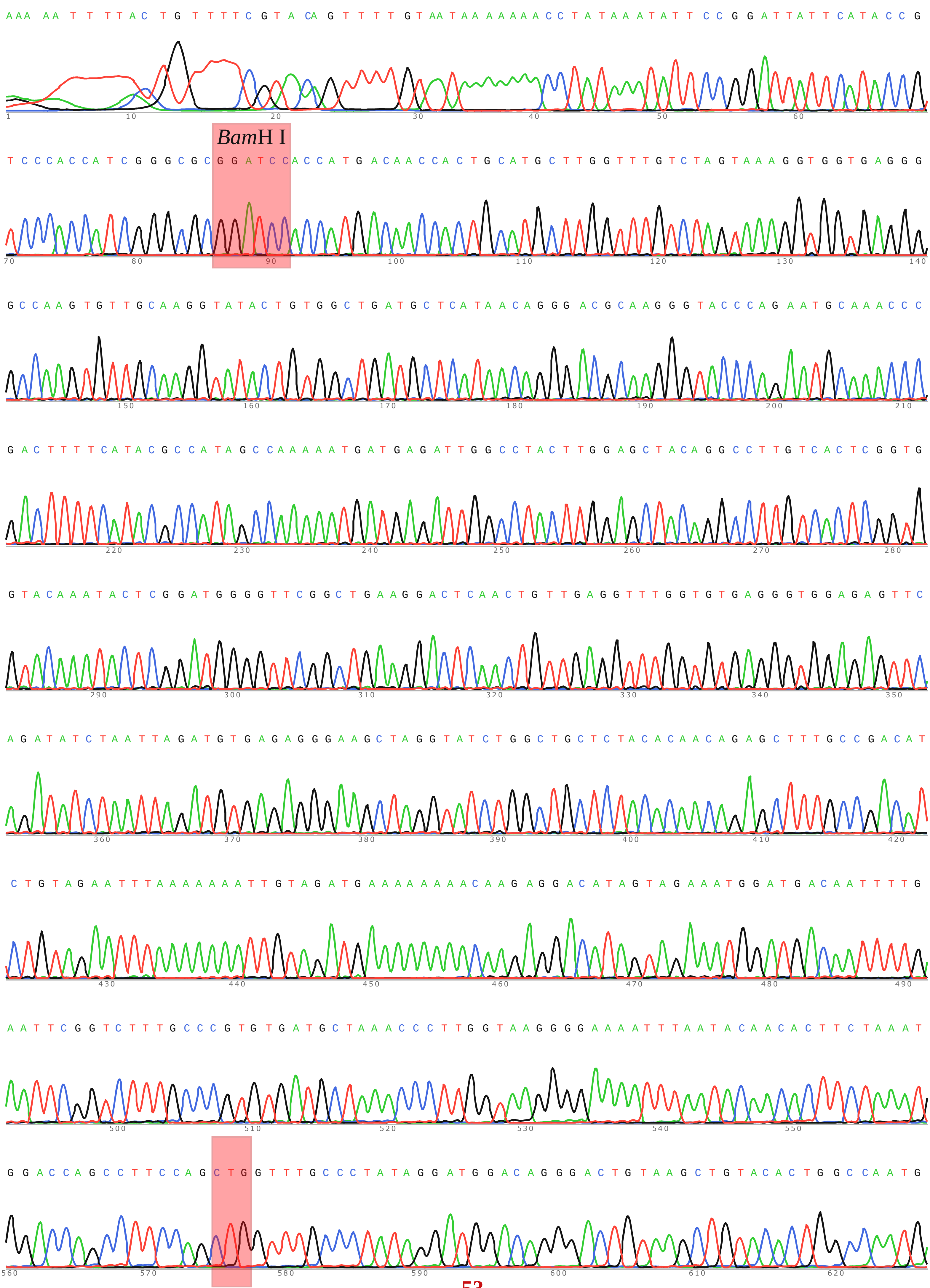

A A G A T A C G T T A G C C A C A A C C G T T G T G C G A A C A T A T A A G A G G G T C A G G C C T T T T C C A T A T A G G C A G G

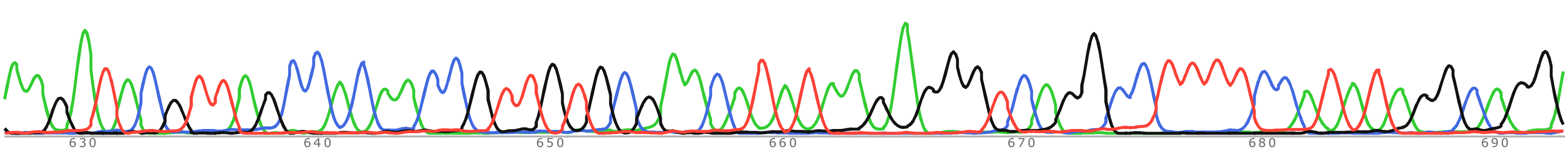

A C T G T G T C A C A C A G A A A A C C A T T G G G G A A G A C C T C T A T G A C T G T G C C C T A G G A G G G A A T T G G A C C

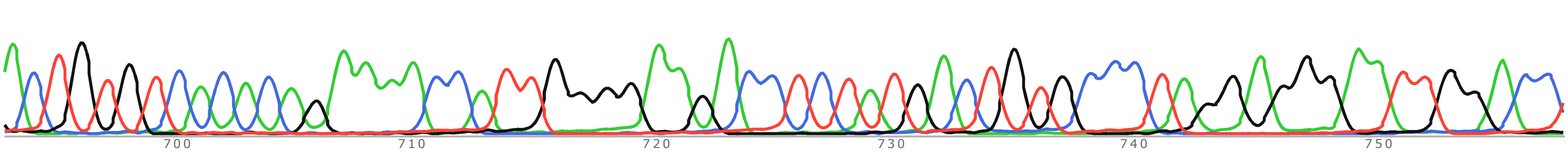

T G T G T G T T G G G G G A T A T A C T A C G A T A T G T A G C T G G G C C T G T C G A G T C T T G T A A G T G G T G T G G T

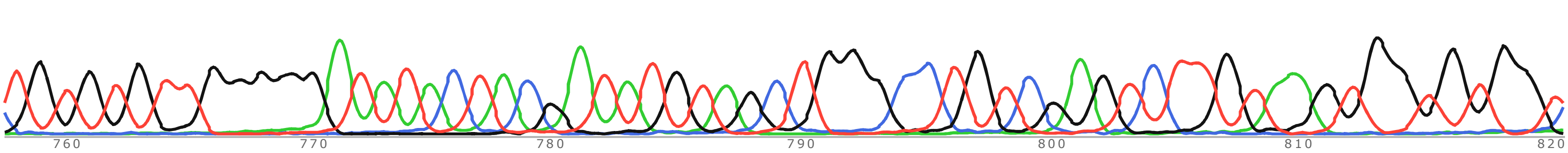

C A C A A A T T T C T T A A A A G T G A G G G T C T G C C G C A T T T C C C A A T T G G C A A A T G C A G G C T G A A G A

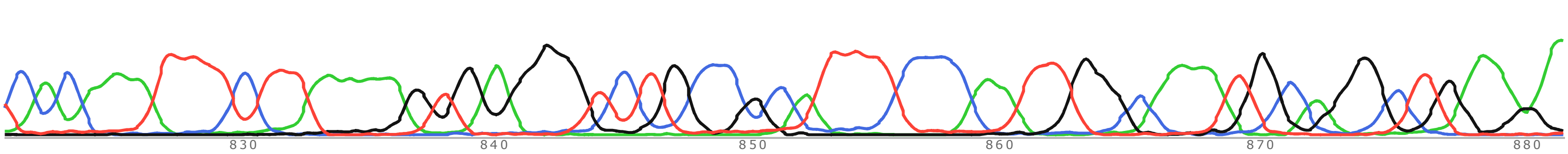

A T G A G A G T G G C T A T A G A C T A G T G G A T G G G A C T T C T T G C A A C A G A G A C G G C G T G G C T A

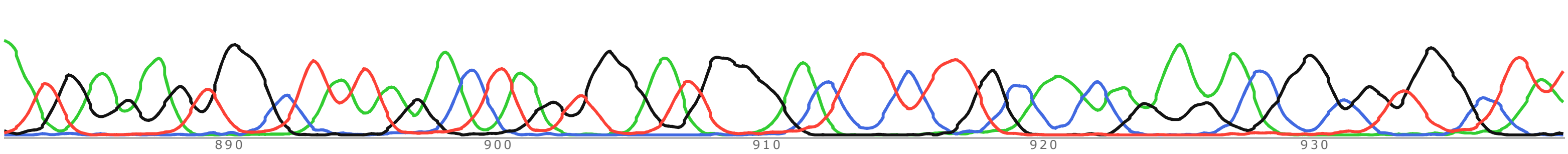

T A G T G C C A T C T G G C C C G G G G T C A A A T G C A A A A T A G G A G A C A C A G T G G T G C A G G T C A

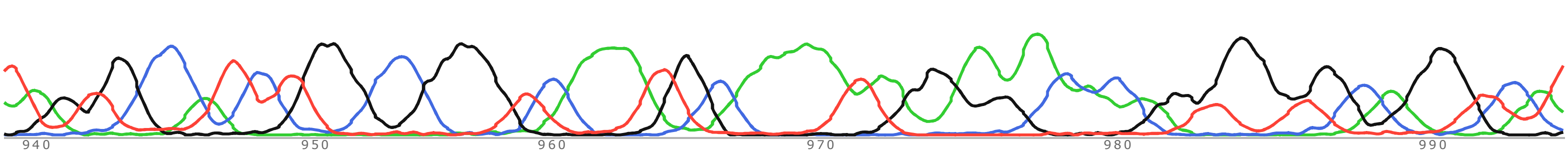

T T G C A A C G G A T G A T A A

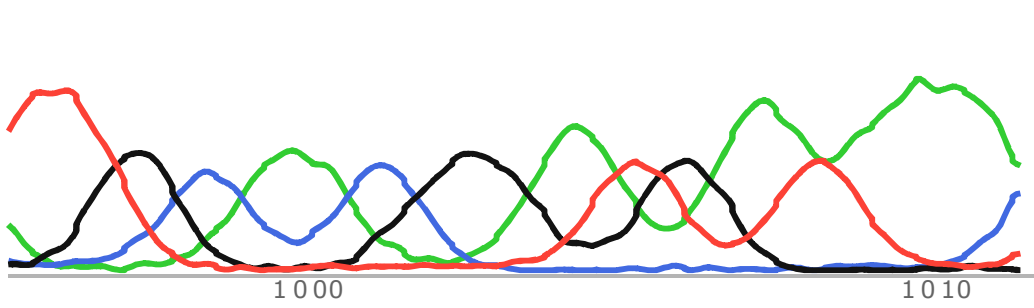

54

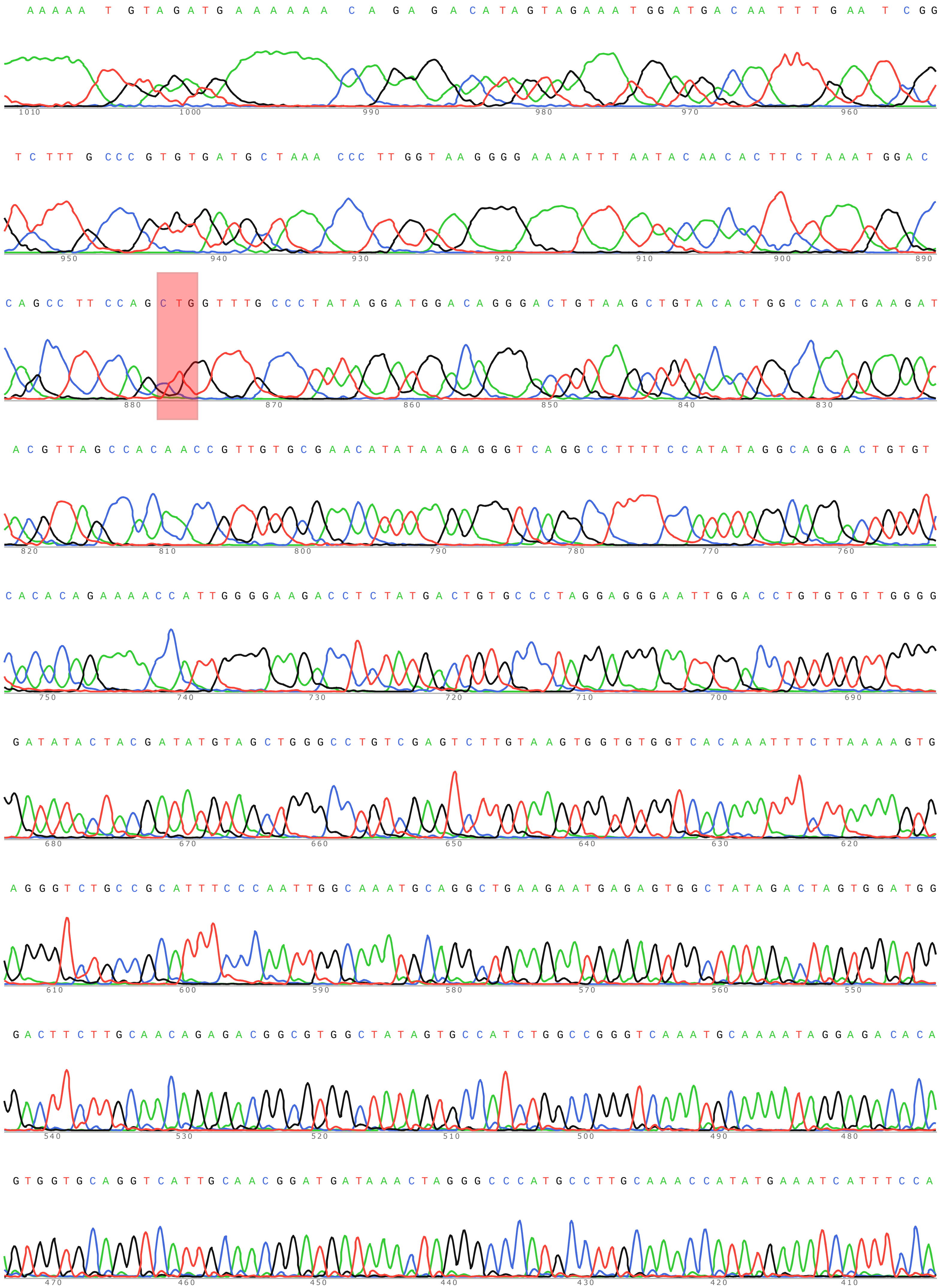
AAAAATGTAGATGAAAAAACAGAGACATAGTAGAAATGGATGACAATTTGAATCGG
TCTTTGCCCGTGTGATGCTAAACCCTTGGTAAGGGGAAAATTTAATACAACACTTCTAAATGGAC
CAGCCTTCCAGCTGGTTTGCCCTATAGGATGGACAGGGACTGTAAGCTGTACACTGGCCAATGAAGAT
ACGTTAGCCACAACCGTTGTGCGAACATATAAGAGGGTCAGGCCTTTTCCATATAGGCAGGACTGTGT
CACACAGAAAACCATTGGGGAAGACCTCTATGACTGTGCCCTAGGAGGGAATTGGACCTGTGTGTTGGGG
GATATACTACGATATGTAGCTGGGCCTGTCGAGTCTTGTAAGTGGTGTGGTCACAAATTTCTTAAAAGTG
AGGGTCTGCCGCATTTCCCAATTGGCAAATGCAGGCTGAAGAATGAGAGTGGCTATAGACTAGTGGATGG
GACTTCTTGCAACAGAGACGGCGTGGCTATAGTGCCATCTGGCCGGGTCAAATGCAAAATAGGAGACACA
GTGGTGCAGGTCATTGCAACGGATGATAAACTAGGGCCCATGCCTTGCAAACCATATGAAATCATTTCCA

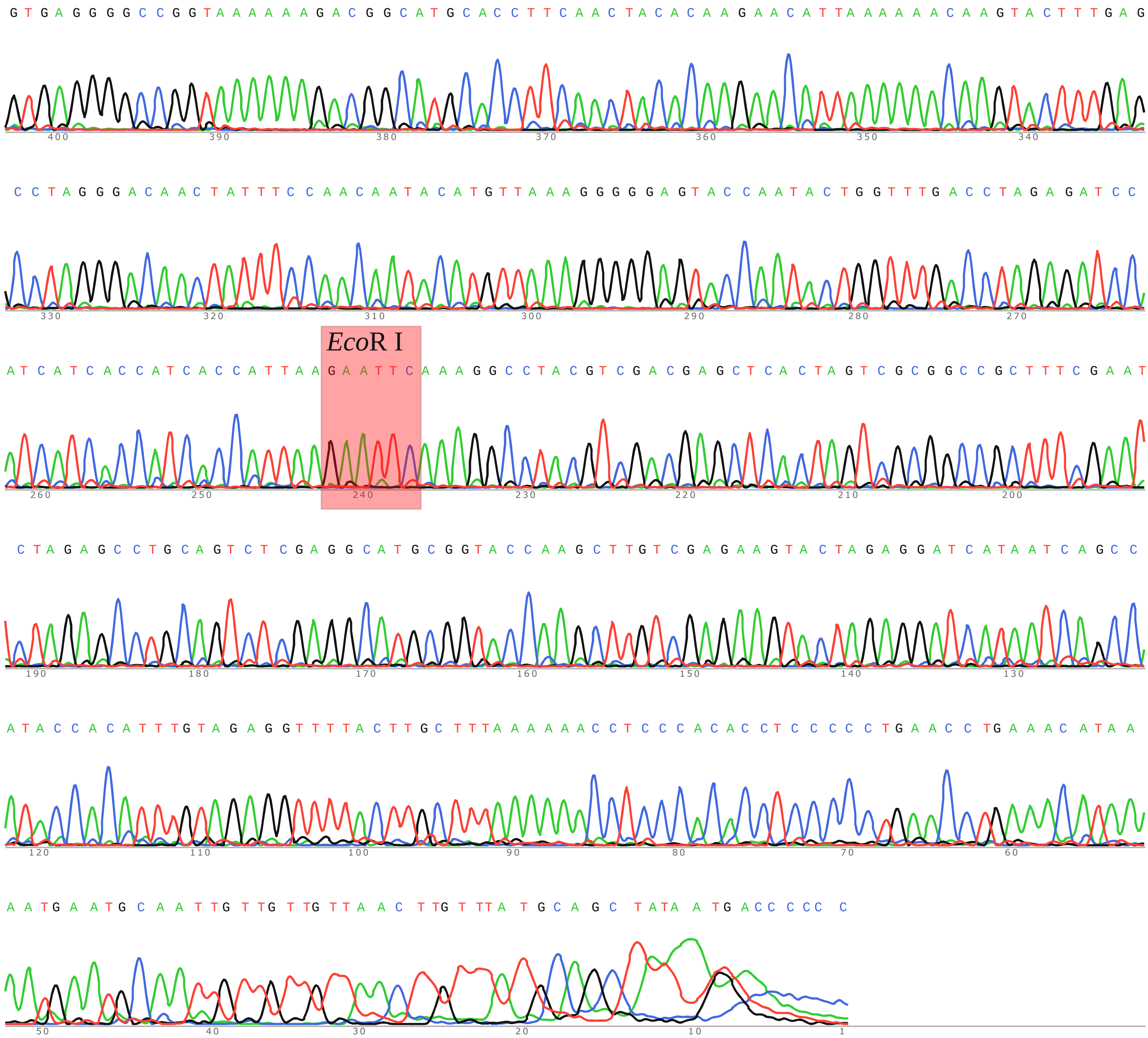
GTGAGGGGCCGGTAAAAAAGACGGCATGCACCTTCAACTACACAAGAACATTAAAAAACAAGTACTTTGAG
CCTAGGGACAACTATTTCCAACAATACATGTTAAAGGGGGAGTACCAATACTGGTTTGACCTAGAGATCC
*Eco*R I
ATCATCACCATCACCATTAAGAATTCAAAGGCCTACGTCGACGAGCTCACTAGTCGCGGCCGCTTTCGAAT
CTAGAGCCTGCAGTCTCGAGGCATGCGGTACCAAGCTTGTCGAGGAAGTACTAGAGGATCATAATCAGCC
ATACCACATTTGTAGAGGTTTTACTTGCTTTAAAAAACCTCCCACACCTCCCCCTGAACCTGAAACATAA
AATGAATGCAATTGTTGTTGTTAACTTGTTTATGCAGCTATAATGACCCCCC

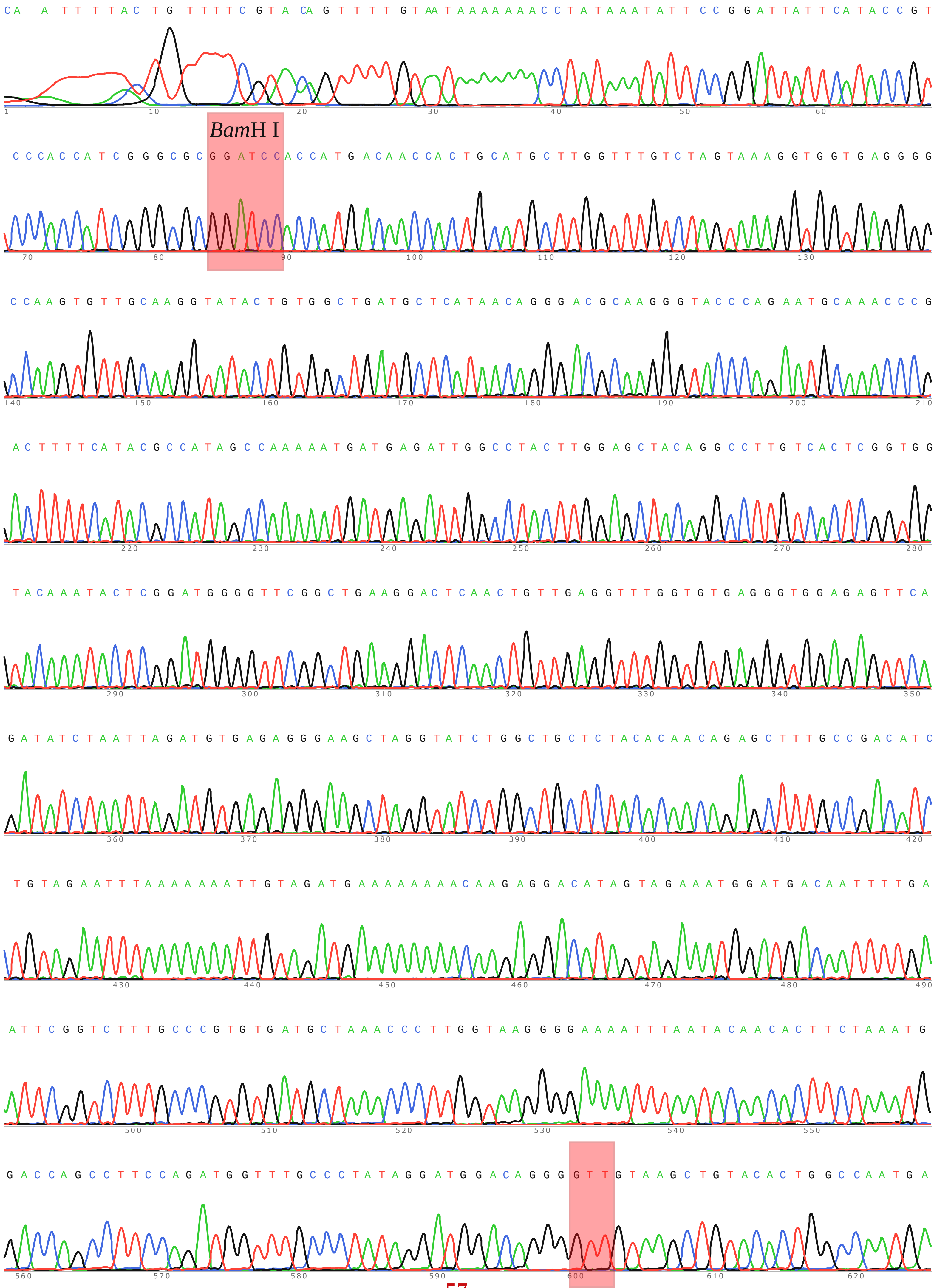
CAATTTTACTGTTTTCGTACAGTTTTGTAATAAAAAAACCTATAAATATTCCGGATTATTCATACCGT
*Bam*H I
CCCACCATCGGGCGCGGATCCACCATGACAACCACTGCATGCTTGGTTTGTCTAGTAAAGGTGGTGAGGGG
CCAAGTGTTGCAAGGTATACTGTGGCTGATGCTCATAACAGGGACGCAAGGGTACCCAGAATGCAAACCCG
ACTTTTCATACGCCATAGCCAAAAATGATGAGATTGGCCTACTTGGAGCTACAGGCCTTGTCACTCGGTGG
TACAAATACTCGGATGGGGTTCGGCTGAAGGACTCAACTGTTGAGGTTTGGTGTGAGGGTGGAGAGTTCA
GATATCTAATTAGATGTGAGAGGGAAGCTAGGTATCTGGCTGCTCTACACAACAGAGCTTTGCCGACATC
TGTAGAATTTAAAAAAATTGTAGATGAAAAAAAACAAGAGGACATAGTAGAAATGGATGACAATTTTGA
ATTCGGTCTTTGCCCGTGTGATGCTAAACCCTTGGTAAGGGGAAAATTTAATACAACACTTCTAAATG
GACCAGCCTTCCAGATGGTTTGCCCTATAGGATGGACAGGGGTTGTAAGCTGTACACTGGCCAATGA

A G A T A C G T T A G C C A C A A C C G T T G T G C G A A C A T A T A A G A G G G T C A G G C C T T T T C C A T A T A G G C A G G A

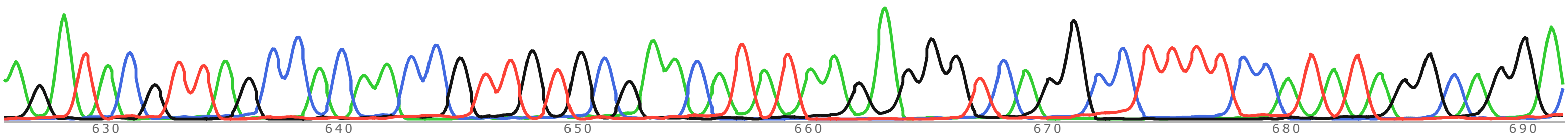

C T G T G T C A C A C A G A A A A C C A T T G G G G A A G A C C T C T A T G A C T G T G C C C T A G G A G G G A A T T G G A C C T

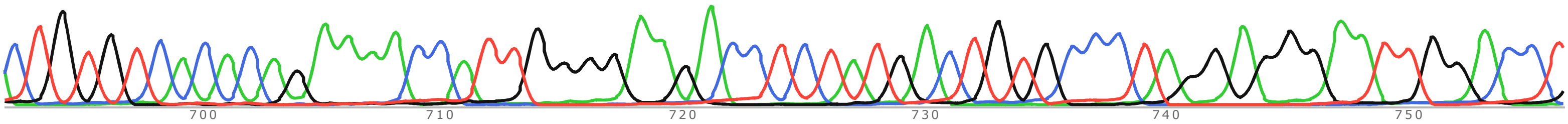

G T G T G T T G G G G A T A T A C T A C G A T A T G T A G C T G G G C C T G T C G A G T C T T G T A A G T G G T G T G G T

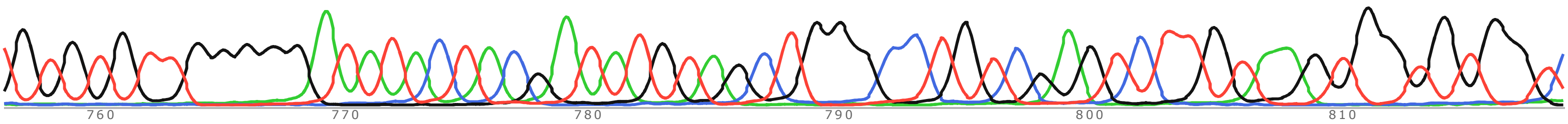

C A C A A A T T T C T T A A A A G T G A G G G T C T G C C G C A T T T C C C A A T T G G C A A A T G C A G G C T G A A G A A

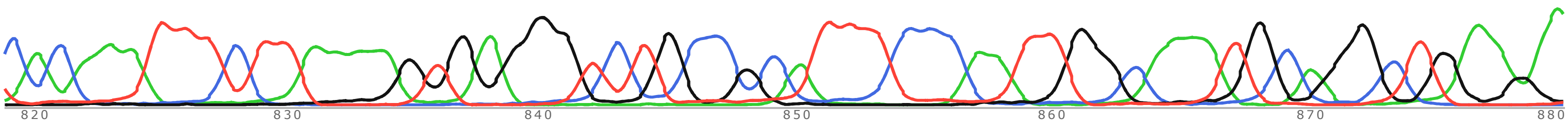

A T G A G A G T G G C T A T A G A C T A G T G G A T G G G A C T T C T T G C AA C A G A G A C GG C G T GG C T A

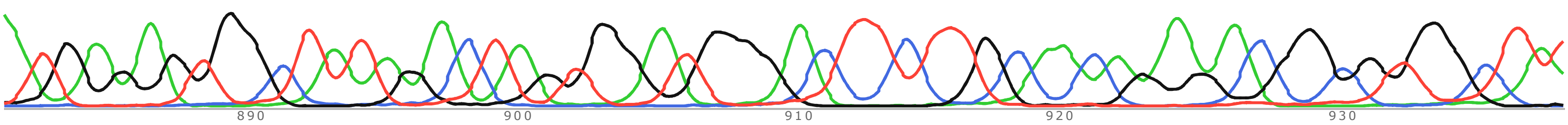

T A G T G C C A T C T G G C C G G G T C A A A T G C A A A A T A GG A G A C A C A G T GG T G C A GG T C A

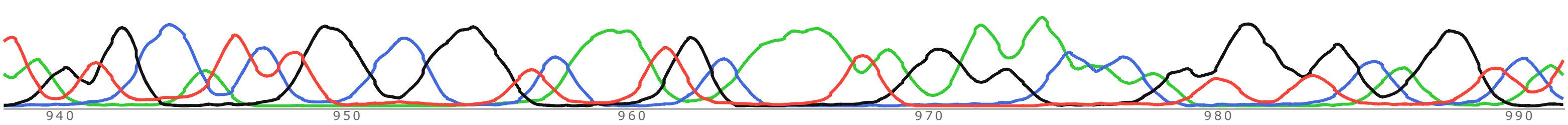

TT G C AA CC GG A T G A T A A A C

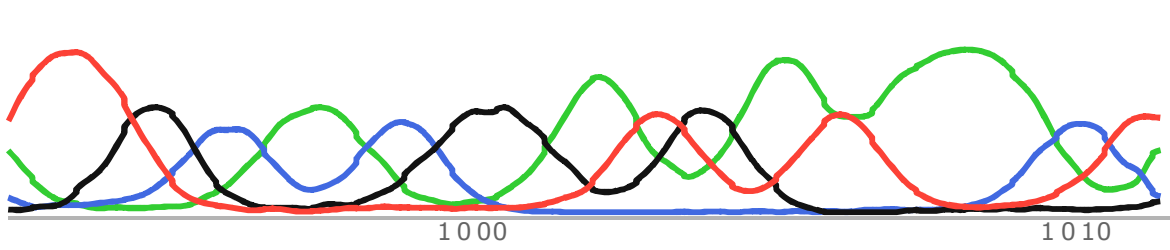

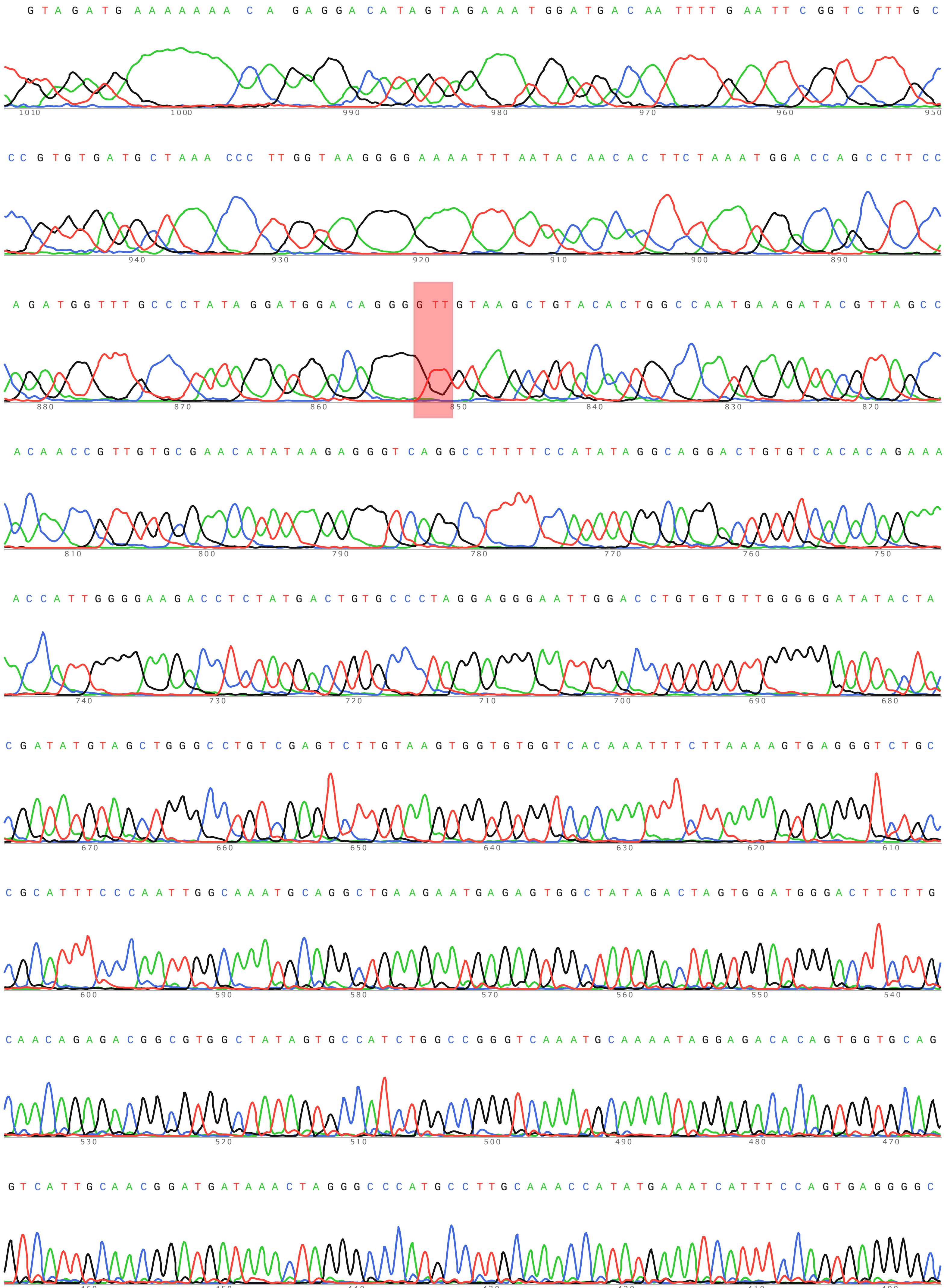
GTAGATGAAAAAAACAGAGGACATAGTAGAAATGGATGACAATTTTGAATTCGGTCTTTGC
1010 1000 990 980 970 960 950
CCGTGTGATGCTAAACCCTTGGTAAGGGGAAAATTTAATACAACACTTCTAAATGGACCAGCCTTCC
940 930 920 910 900 890
AGATGGTTTGCCCTATAGGATGGACAGGGGTTGTAAGCTGTACACTGGCCAATGAAGATACGTTAGCC
880 870 860 850 840 830 820
ACAACCGTTGTGCGAACATATAAGAGGGTCAGGCCTTTTCCATATAGGCAGGACTGTGTCACACAGAAA
810 800 790 780 770 760 750
ACCATTGGGGAAGACCTCTATGACTGTGCCCTAGGAGGGAATTGGACCTGTGTGTTGGGGATATACTA
740 730 720 710 700 690 680
CGATATGTAGCTGGGCCTGTCGAGTCTTGTAAGTGGTGTGGTCACAAATTTCTTAAAAGTGAGGGTCTGC
670 660 650 640 630 620 610
CGCATTTCCCAATTGGCAAATGCAGGCTGAAGAATGAGAGTGGCTATAGACTAGTGGATGGGACTTCTTG
600 590 580 570 560 550 540
CAACAGAGACGGCGTGGCTATAGTGCCATCTGGCCGGGTCAAATGCAAAATAGGAGACACAGTGGTGCAG
530 520 510 500 490 480 470
GTCATTGCAACGGATGATAAACTAGGGCCCATGCCTTGCAAACCATATGAAATCATTTCCAGTGAGGGGC
460 450 440 430 420 410 400

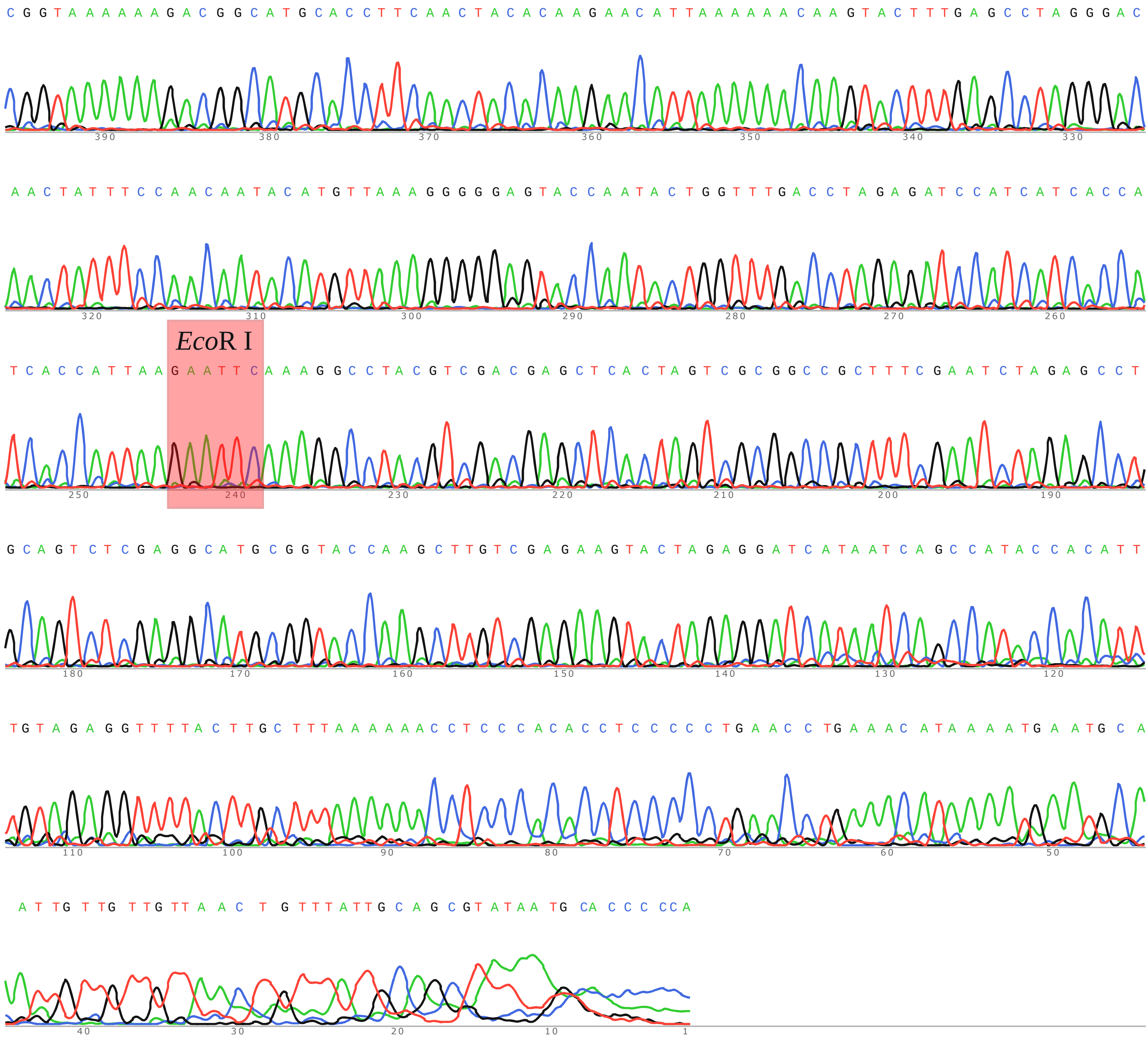
CGGTAAAAAAGACGGCATGCACCTTCAACTACACAAGAACATTAAAAAACAAGTACTTTGAGCCTAGGGAC
390 380 370 360 350 340 330
AACTATTTCCAACAATACATGTTAAAGGGGGAGTACCAATACTGGTTTGACCTAGAGATCCATCATCACCA
320 310 300 290 280 270 260
EcoR I
TCACCATTAAGAATTCAAAGGCCTACGTCGACGAGCTCACTAGTCGCGGCCGCTTTCGAATCTAGAGCCT
250 240 230 220 210 200 190
GCAGTCTCGAGGCATGCGGTACCAAGCTTGTCGAGGAAGTACTAGAGGATCATAATCAGCCATACCACATT
180 170 160 150 140 130 120
TGTAGAGGTTTTACTTGCTTTAAAAACCTCCCACACCTCCCCCTGAACCTGAAACATAAAATGAATGCA
110 100 90 80 70 60 50
ATTGTTGTTGTTAACTGTTTATTGCAGCGTATAATGCACCCCCA
40 30 20 10 1

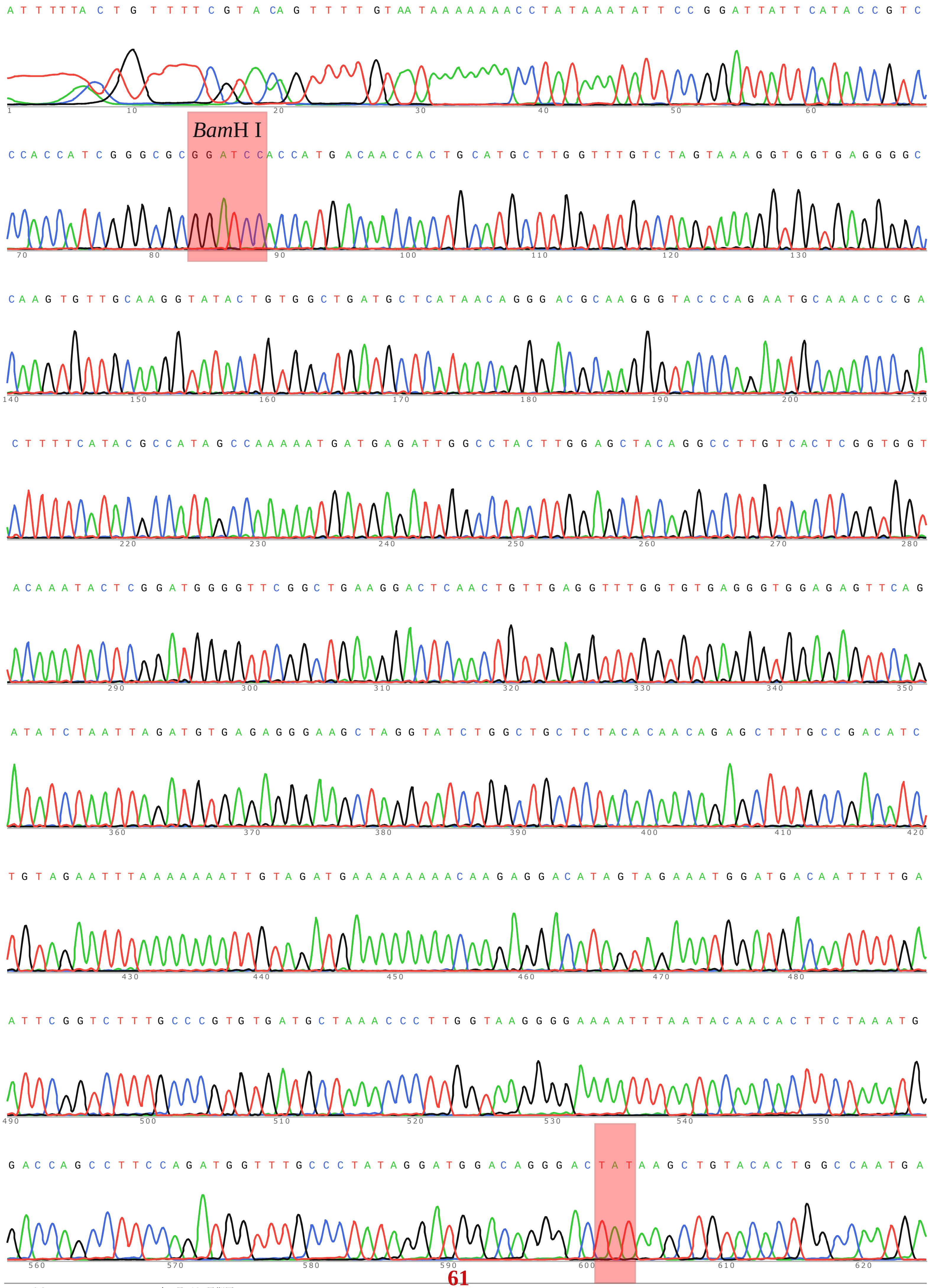
ATTTTTACTGTTTTCGTACAGTTTTGTAATAAAAAAACCTATAAATATTCCGGATTATTCATACCGTC
CCACCATCGGGCGCGGATCCACCATGACAACCACTGCATGCTTGGTTTGTCTAGTAAAGGTGGTGAGGGGC
*Bam*H I
CAAGTGTTGCAAGGTATACTGTGGCTGATGCTCATAACAGGGACGCAAGGGTACCCAGAATGCAAACCCGA
CTTTTCATACGCCATAGCCAAAAATGATGAGATTGGCCTACTTGGAGCTACAGGCCTTGTCACTCGGTGGT
ACAAATACTCGGATGGGGTTCGGCTGAAGGACTCAACTGTTGAGGTTTGGTGTGAGGGTGGAGAGTTCAG
ATATCTAATTAGATGTGAGAGGGAAGCTAGGTATCTGGCTGCTCTACACAACAGAGCTTTGCCGACATC
TGTAGAATTTAAAAAAATTGTAGATGAAAAAAAACAAGAGGACATAGTAGAAATGGATGACAATTTTGA
ATTCGGTCTTTGCCCGTGTGATGCTAAACCCTTGGTAAGGGGAAAATTTAATACAACACTTCTAAATG
GACCAGCCTTCCAGATGGTTTGCCCTATAGGATGGACAGGGACTATAAGCTGTACACTGGCCAATGA

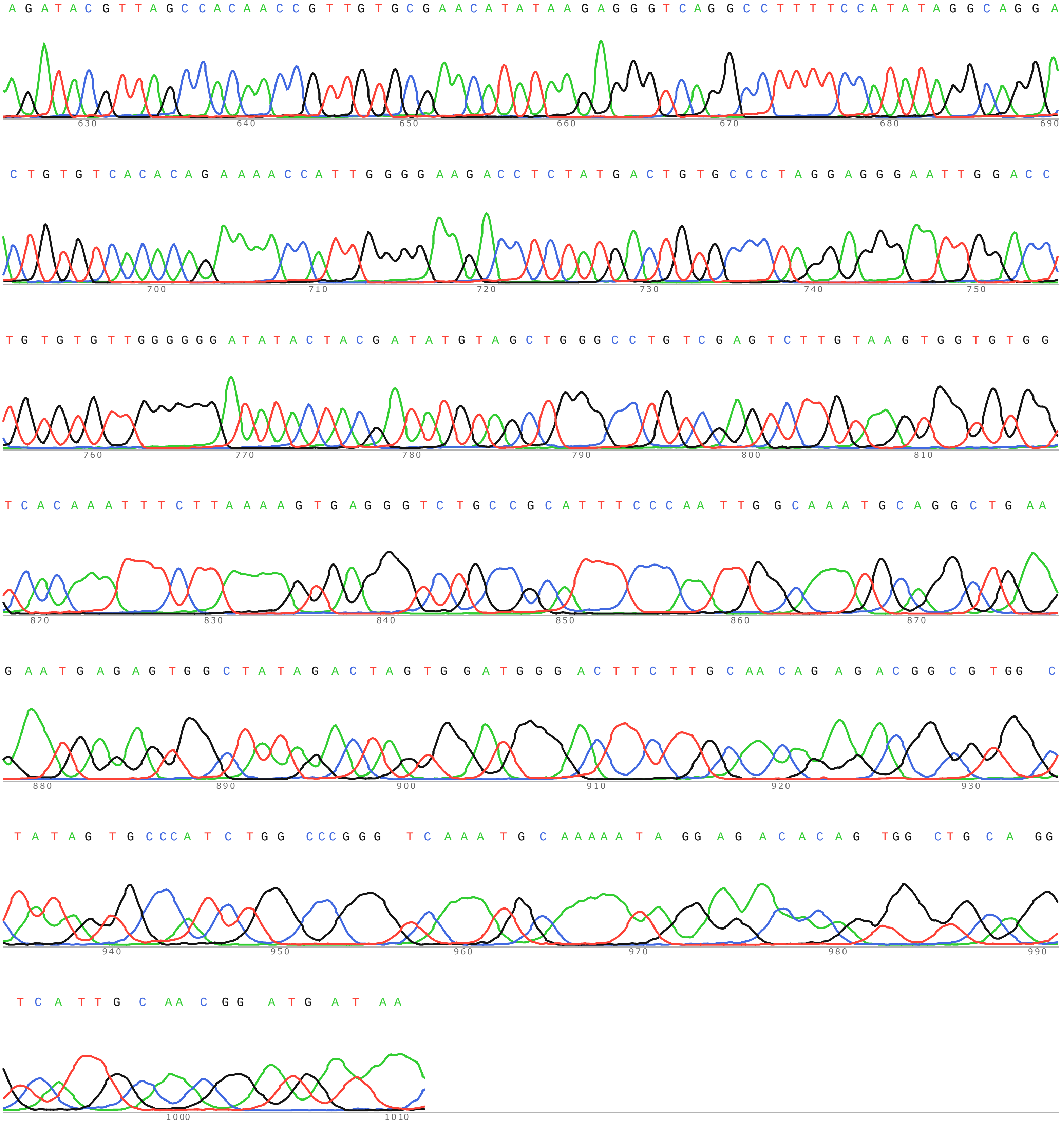
AGATACGTTAGCCACAACCGTTGTGCGAACATATAAGAGGGTCAGGCCTTTTCCATATAGGCAGGA
630 640 650 660 670 680 690
CTGTGTCACACAGAAAACCATTGGGGAAGACCTCTATGACTGTGCCCTAGGAGGGAATTGGACC
700 710 720 730 740 750
TGTGTGTTGGGGGGATATACTACGATATGTAGCTGGGCCTGTCGAGTCTTGTAAGTGGTGTGG
760 770 780 790 800 810
TCACAAATTTCTTAAAAGTGAGGGTCTGCCGCATTTCCCAATTGGCAAATGCAGGCTGAA
820 830 840 850 860 870
GAATGAGAGTGGCTATAGACTAGTGGATGGGACTTCTTGCAACAGAGACGGCGTGGC
880 890 900 910 920 930
TATAGTGCCCATCTGGCCCGGGTCAAATGCAAAAATAGGAGACACAGTGGCTGCAGG
940 950 960 970 980 990
TCATTGCAACGGATGATAA
1000 1010

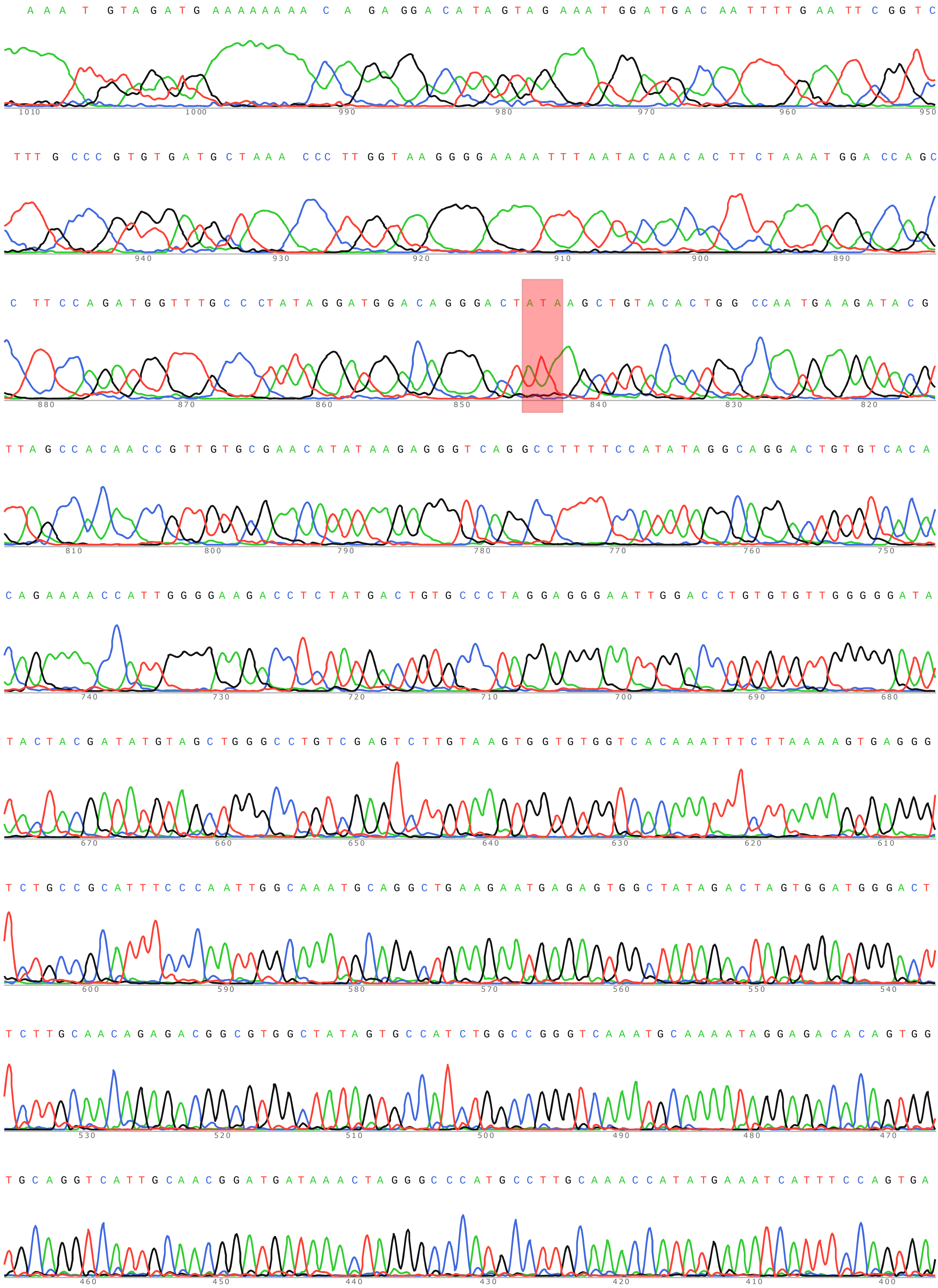
AAATGTAGATGAAAAAAAACAGAGGACATAGTAGAAATGGATGACAATTTTGAATTCGGTC
1010 1000 990 980 970 960 950
TTTGCCCGTGTGATGCTAAACCCTTGGTAAGGGGAAAATTTAATACAACACTTCTAAATGGACCAGC
940 930 920 910 900 890
CTTCCAGATGGTTTGCCCTATAGGATGGACAGGGACTATAAGCTGTACACTGGCCAATGAAGATACG
880 870 860 850 840 830 820
TTAGCCACAACCGTTGTGCGAACATATAAGAGGGTCAGGCCTTTTCCATATAGGCAGGACTGTGTCACA
810 800 790 780 770 760 750
CAGAAAACCATTGGGGAAGACCTCTATGACTGTGCCCTAGGAGGGAATTGGACCTGTGTGTTGGGGGATA
740 730 720 710 700 690 680
TACTACGATATGTAGCTGGGCCTGTCGAGTCTTGTAAGTGGTGTGGTCACAAATTTCTTAAAAGTGAGGG
670 660 650 640 630 620 610
TCTGCCGCATTTCCCAATTGGCAAATGCAGGCTGAAGAATGAGAGTGGCTATAGACTAGTGGATGGGACT
600 590 580 570 560 550 540
TCTTGCAACAGAGACGGCGTGGCTATAGTGCCATCTGGCCGGGTCAAATGCAAAATAGGAGACACAGTGG
530 520 510 500 490 480 470
TGCAGGTCATTGCAACGGATGATAAACTAGGGCCCATGCCTTGCAAACCATATGAAATCATTTCCAGTGA
460 450 440 430 420 410 400

G G G G C C G G T A A A A A A G A C G G C A T G C A C C T T C A A C T A C A C A A G A A C A T T A A A A A A C A A G T A C T T T G A G C C T A

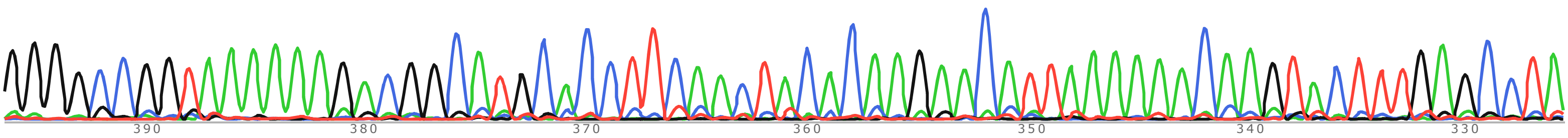

G G G A C A A C T A T T T C C A A C A A T A C A T G T T A A A G G G G G A G T A C C A A T A C T G G T T T G A C C T A G A G A T C C A T C A T

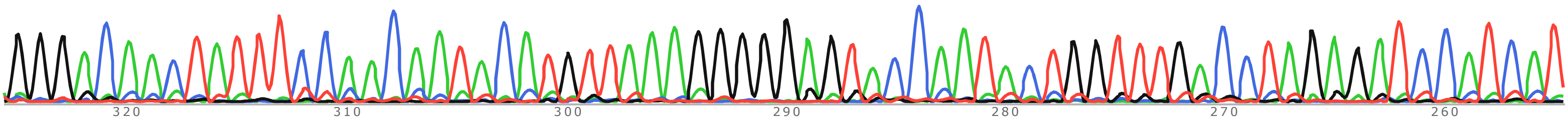

*Bam*H I

C A C C A T C A C C A T T A A G A A T T C A A A G G C C T A C G T C G A C G A G C T C A C T A G T C G C G G C C G C T T T C G A A T C T A G

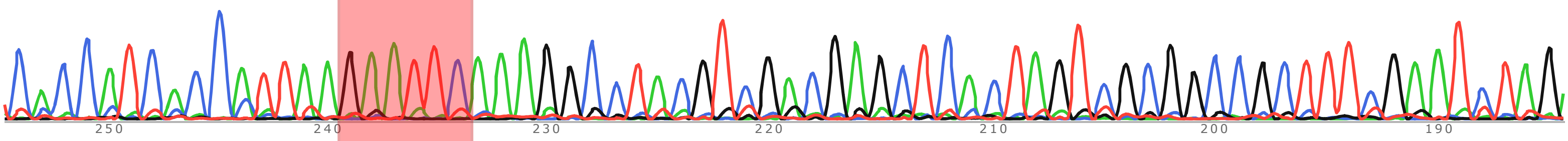

A G C C T G C A G T C T C G A G G C A T G C G G T A C C A A G C T T G T C G A G A A G T A C T A G A G G A T C A T A A T C A G C C A T A C C

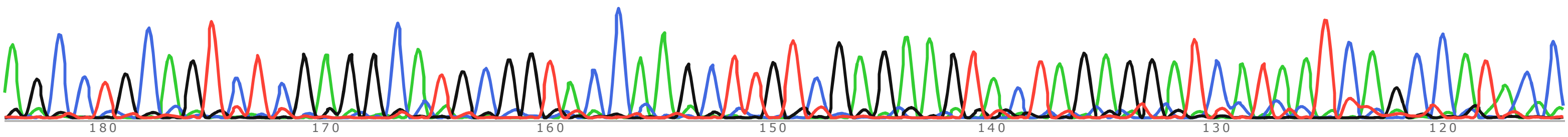

A C A T T T G T A G A G G T T T T A C T T G C T T T A A A A A C C T C C C A C A C C T C C C C C T G A A C C T G A A A C A T A A A A T G A

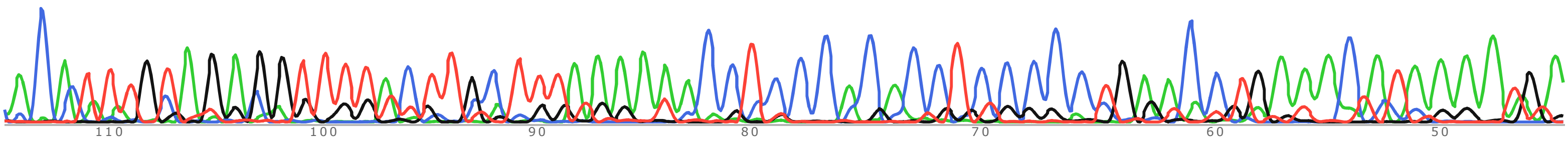

A T G C A A T T G T T G T T G T T A A C T T G T T A T G C A G C T A T A C T A C C A A A

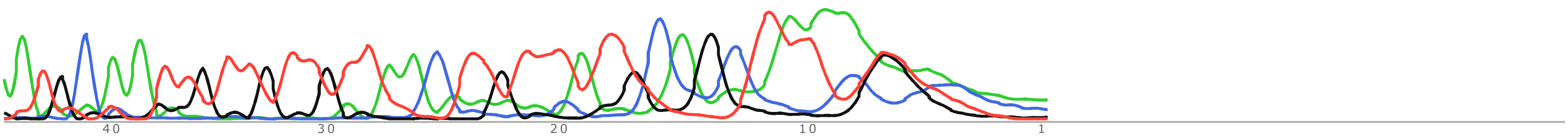

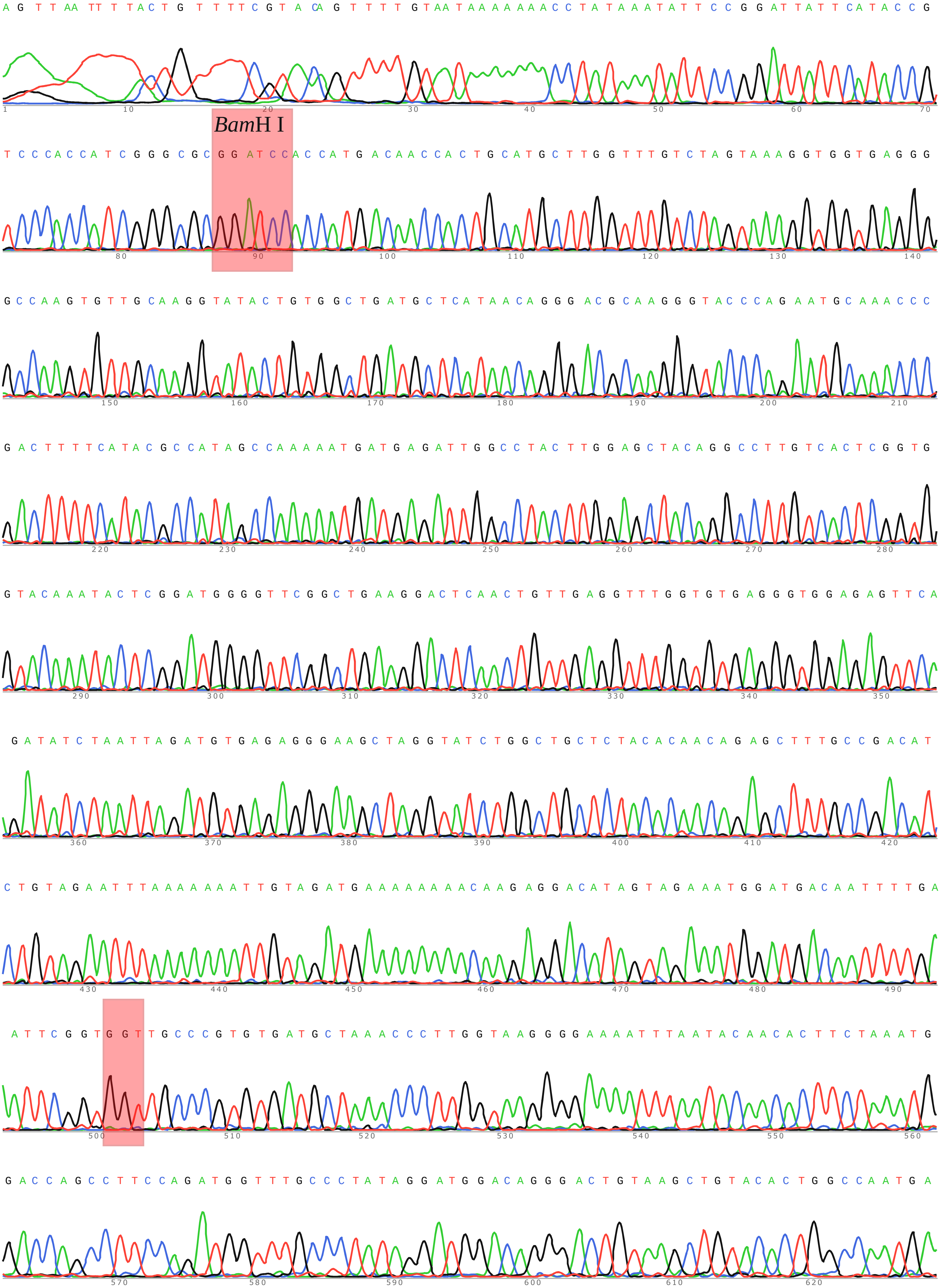
*Bam*H I

A G A T A C G T T A G C C A C A A C C G T T G T G C G A A C A T A T A A G A G G G T C A G G C C T T T T C C A T A T A G G C A G G A C

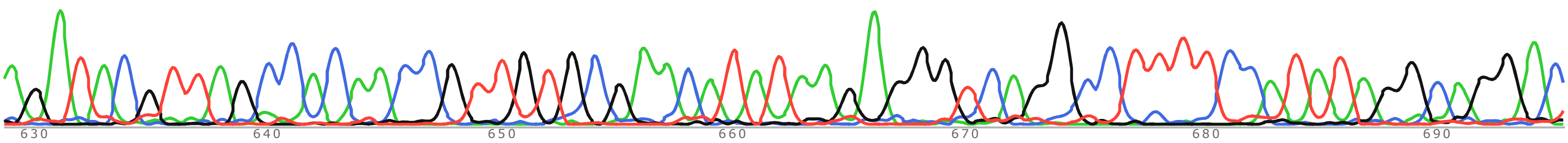

T G T G T C A C A C A G A A A A C C A T T G G G G A A G A C C T C T A T G A C T G T G C C C T A G G A G G G A A T T G G A C C T G

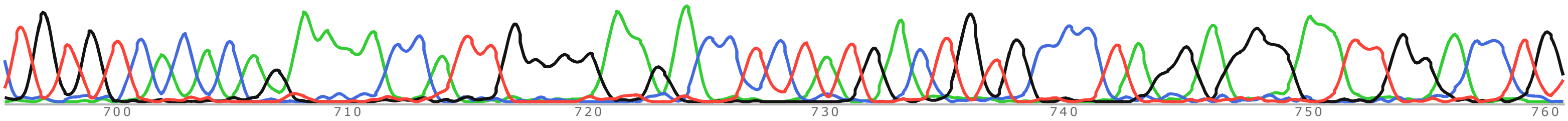

T G T G T T G G G G G A T A T A C T A C G A T A T G T A G C T G G G C C T G T C G A G T C T T G T A A G T G G T G T G G T C A C

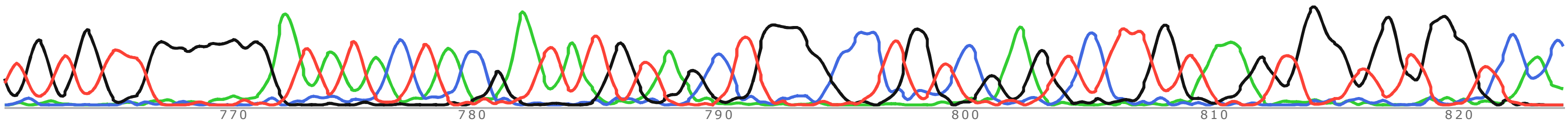

A A A T T T C T T A A A A G T G A G G G T C T G C C G C A T T T C C C A A T T G G C A A A T G C A G G C T G A A G A A T G

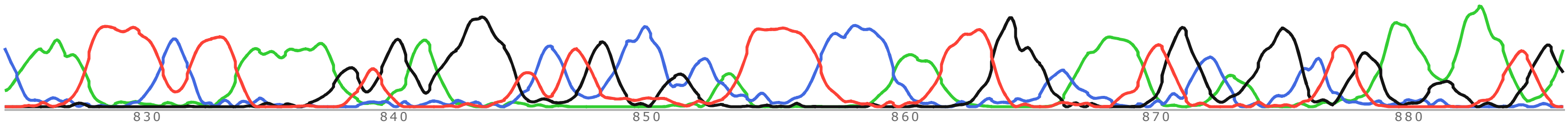

A G A G T G G C T A T A G A C T A G T G G A T G G G A C T T C T T G C A C A G A G A C G G C G T G G C T A T A G T

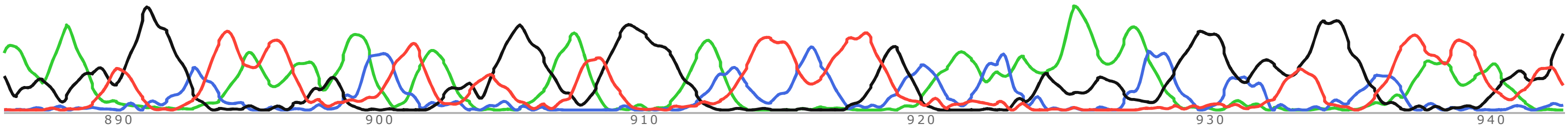

G C C A T C T G G C C G G G T C A A A T G C A A A A A T A G G A G A C A C A G T G G T G C A G G T C A T T G C

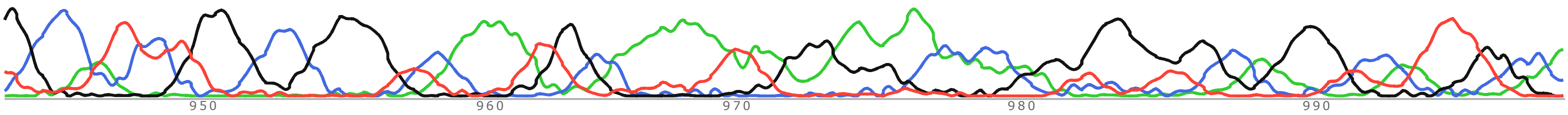

A C G G A T G A T A A A C

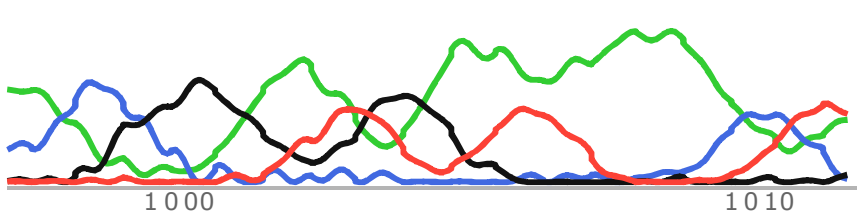

AAAAA T GTAGATGAAAAAAA CA GAGGACATAGTAGAAAT GGATGAC AAT T T G AA TT C GGT

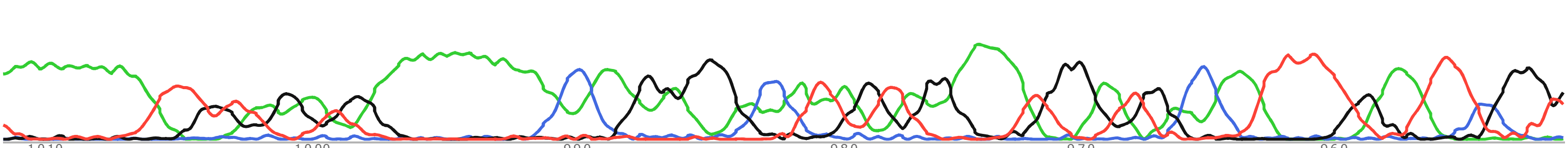

1010 1000 990 980 970 960

GG TT G CCCGTGTGATGCTAAA CC TTGGT AA GGGG AAAATTT AATAC AAC AC TT C TAAAT GG A CC AG

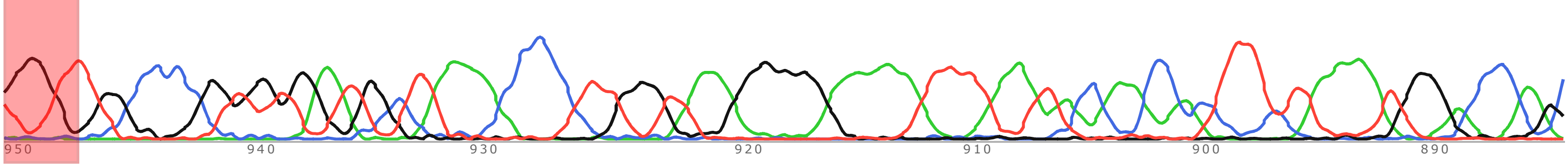

950 940 930 920 910 900 890

CC TTCCAGATGG TTTGCCCTATA GGATGG ACA GGGACTGTAAGCTGTACACTGG CCAATGAAGATACG

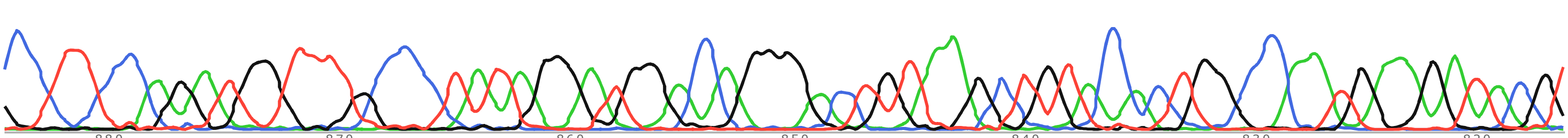

880 870 860 850 840 830 820

TTAGC CACAACCG TTGTGCGAACATATAA GA GGGTCAGGCCTTTTCCATATAGGCAGG ACTGTGTCACAC

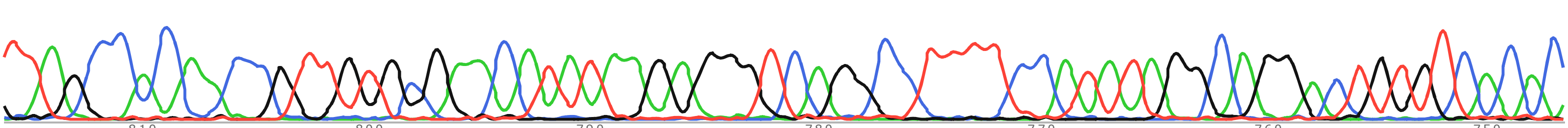

810 800 790 780 770 760 750

AGAAAACCATTGGGGAAGACCTCTATGACTGTGCCCTAGGAGGGAATTGGACCTGTGTGTTGGGGGATAT

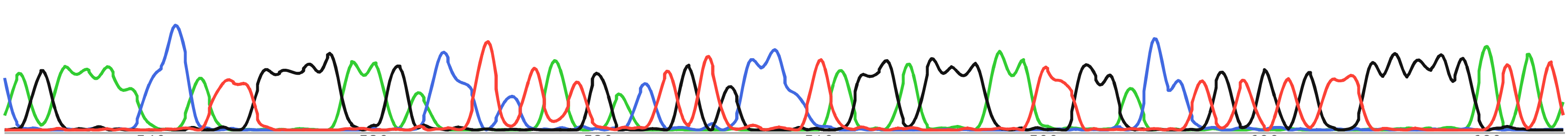

740 730 720 710 700 690 680

ACTACGATATGTAGCTGGGCCTGTCGAGTCTTGTAAGTGGTGTGGTCACAAATTTCTTAAAAGTGAGGGT

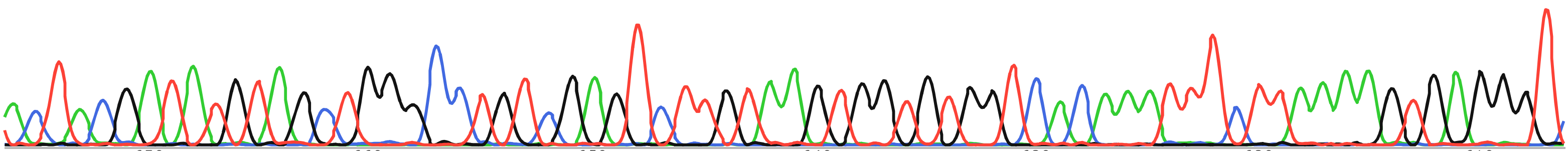

670 660 650 640 630 620 610

CTGCCGCATTTCCCAATTGGCAAATGCAGGCTGAAGAATGAGAGTGGCTATAGACTAGTGGATGGGACTT

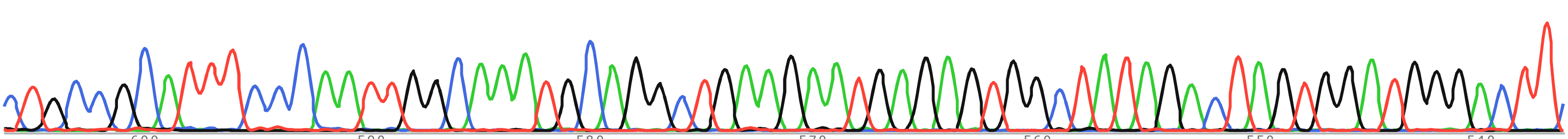

600 590 580 570 560 550 540

CTTGCAACAGAGACGGCGTGGCTATAGTGCCATCTGGCCGGGTCAAATGCAAAATAGGAGACACAGTGGT

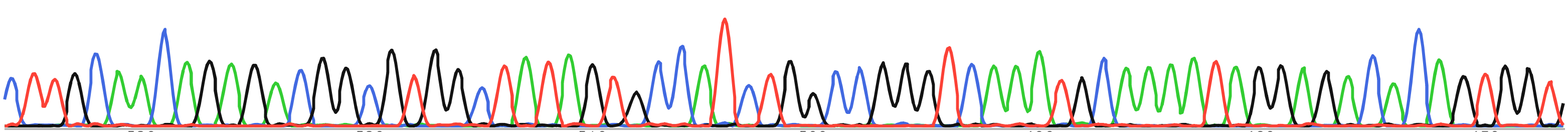

530 520 510 500 490 480 470

GCAGGTCATTGCAACGGATGATAAACTAGGGCCCATGCCTTGCAAACCATATGAAATCATTTCCAGTGAG

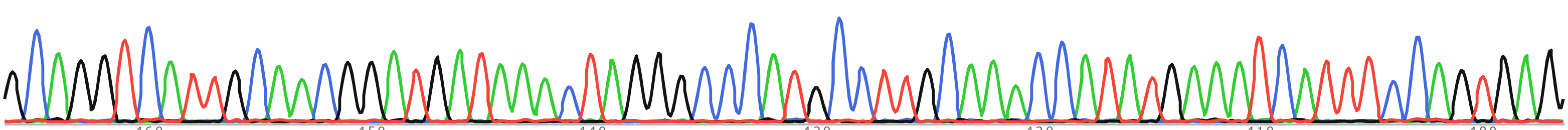

460 450 440 430 420 410 400

GGGCCGGTAAAAAAGACGGCATGCACCTTCAACTACACAAGAACATTAAAAAACAAGTACTTTGAGCCTA

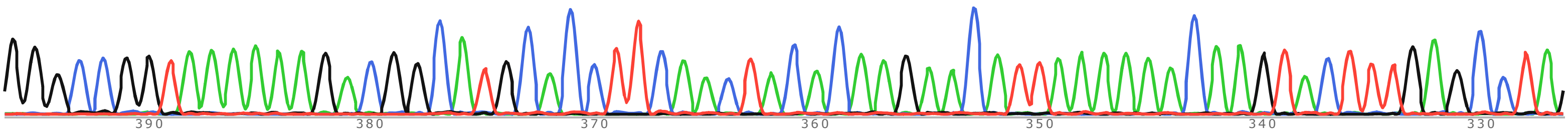

GGGACAACTATTTCCAACAATACATGTTAAAGGGGGAGTACCAATACTGGTTTGACCTAGAGATCCATCAT

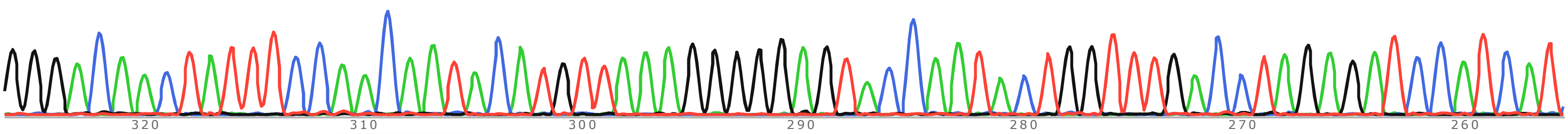

*Eco*R I

CACCATCACCATTAAGAATTCAAAGGCCTACGTCGACGAGCTCACTAGTCGCGGCCGCTTTCGAATCTAG

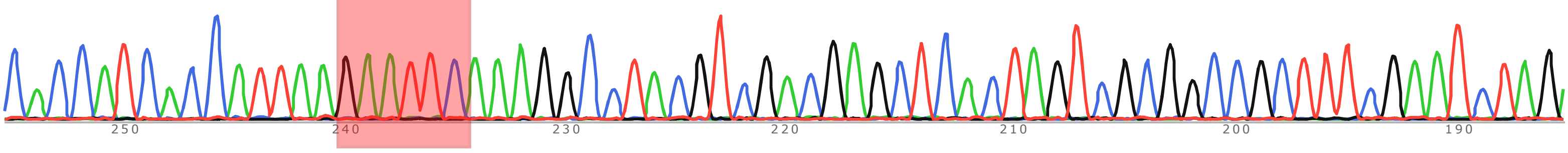

AGCCTGCAGTCTCGAGGCATGCGGTACCAAGCTTGTCGAGAAGTACTAGAGGATCATAATCAGCCATACC

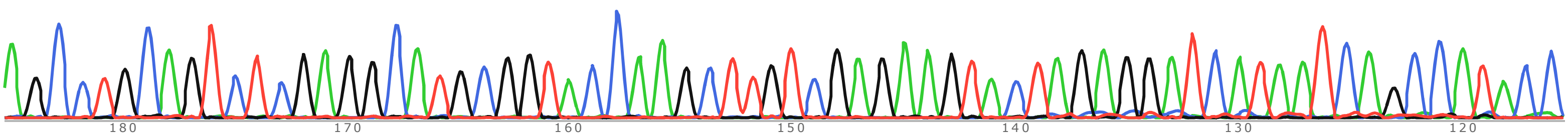

ACATTTGTAGAGGTTTTACTTGCTTTAAAAACCTCCCACACCTCCCCCTGAACCTGAAACATAAAATGA

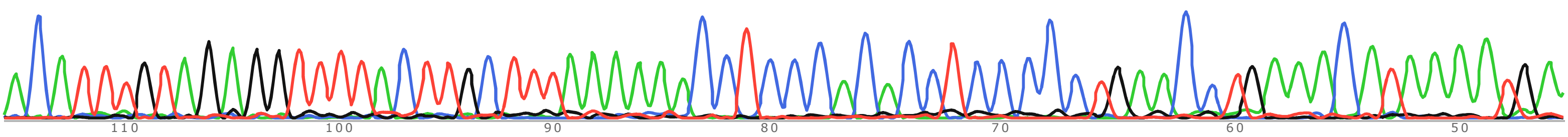

ATGCAATTGTTGTTGTTAACTTGTTATGCAGCTATTAGTACCATG

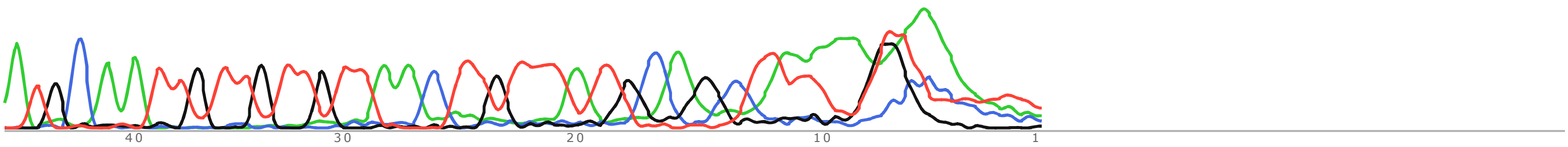

68

BEP-491-pFsat Bac1-BVDV-XH32-E2 (L105A)_poly-F_2023-07-28_D02.ab1 (1010 bases)

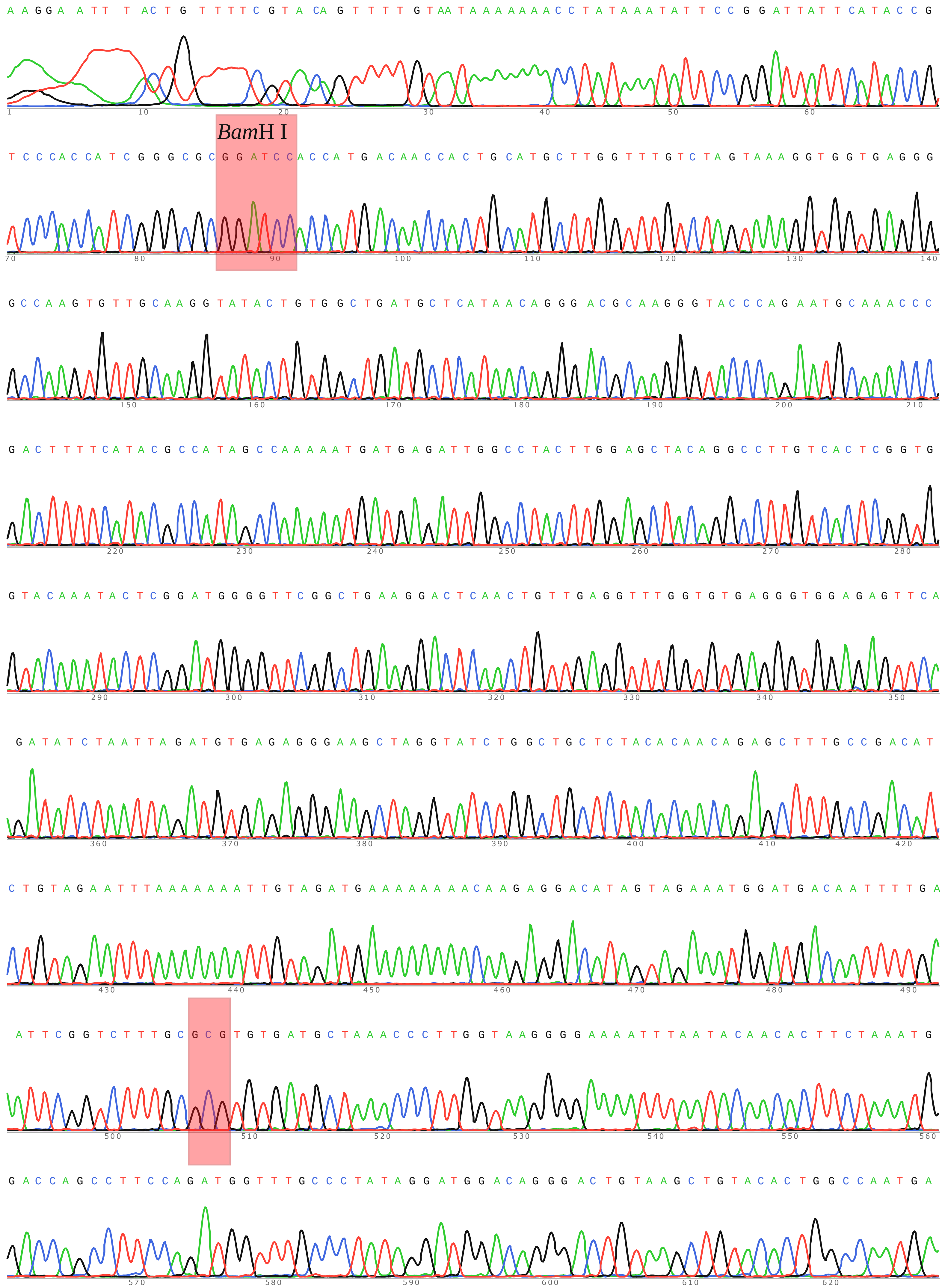

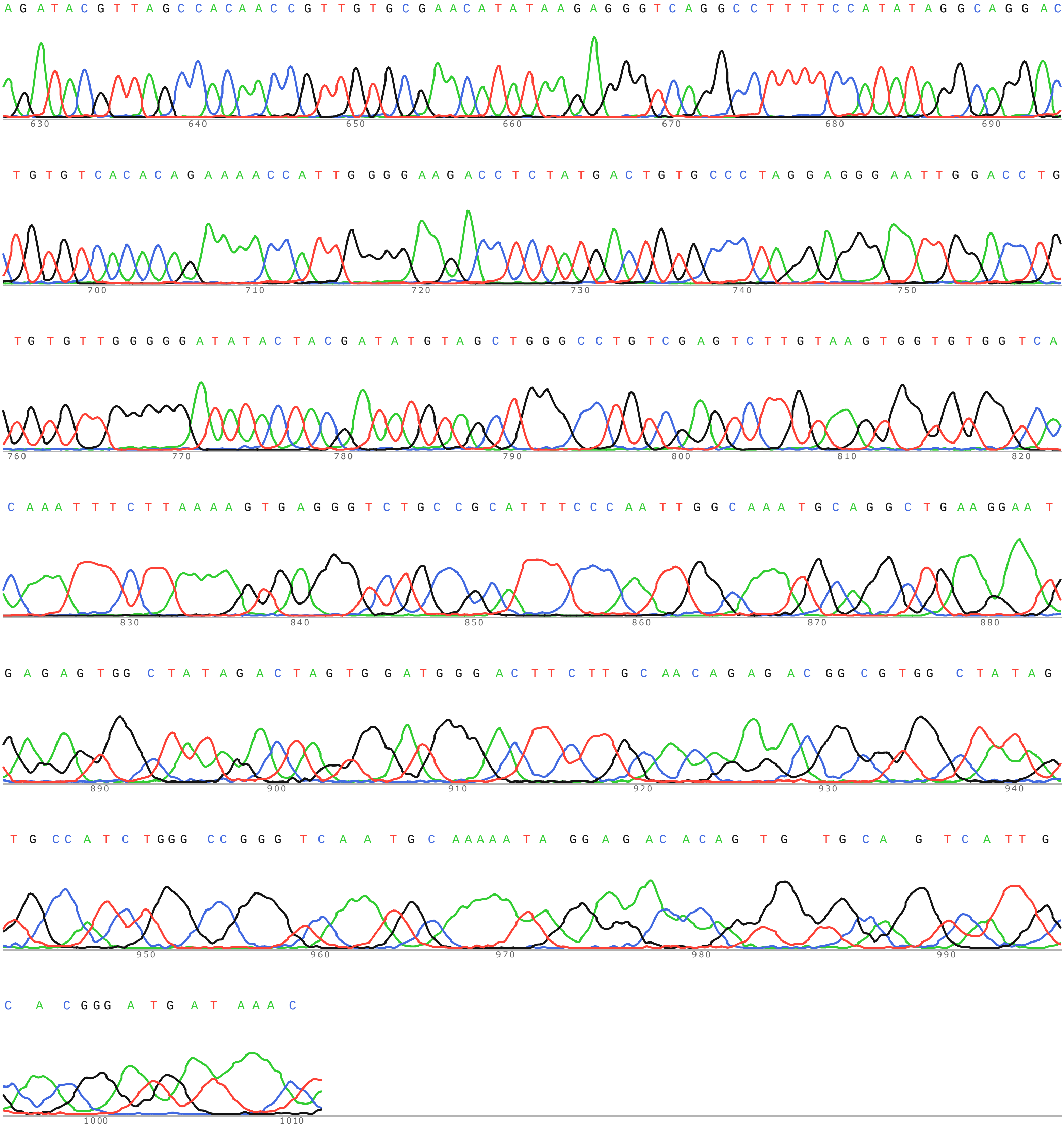
AGATACGTTAGCCACAACCGTTGTGCGAACATATAAGAGGGTCAGGCCTTTTCCATATAGGCAGGAC
630 640 650 660 670 680 690
TGTGTCACACAGAAAACCATTGGGGAAGACCTCTATGACTGTGCCCTAGGAGGGAATTGGACCTG
700 710 720 730 740 750
TGTGTTGGGGGATATACTACGATATGTAGCTGGGCCTGTCGAGTCTTGTAAGTGGTGTGGTCA
760 770 780 790 800 810 820
CAAATTTCTTAAAAGTGAGGGTCTGCCGCATTTCCCAATTGGCAAATGCAGGCTGAAGGAAT
830 840 850 860 870 880
GAGAGTGGCTATAGACTAGTGGATGGGACTTCTTGCAACAGAGACGGCGTGGCTATAG
890 900 910 920 930 940
TGCCATCTGGGCCGGGTCAATGCAAAAATAGGAGACACAGTGTGCAGTCATTG
950 960 970 980 990
CACGGGATGATAAAC
1000 1010

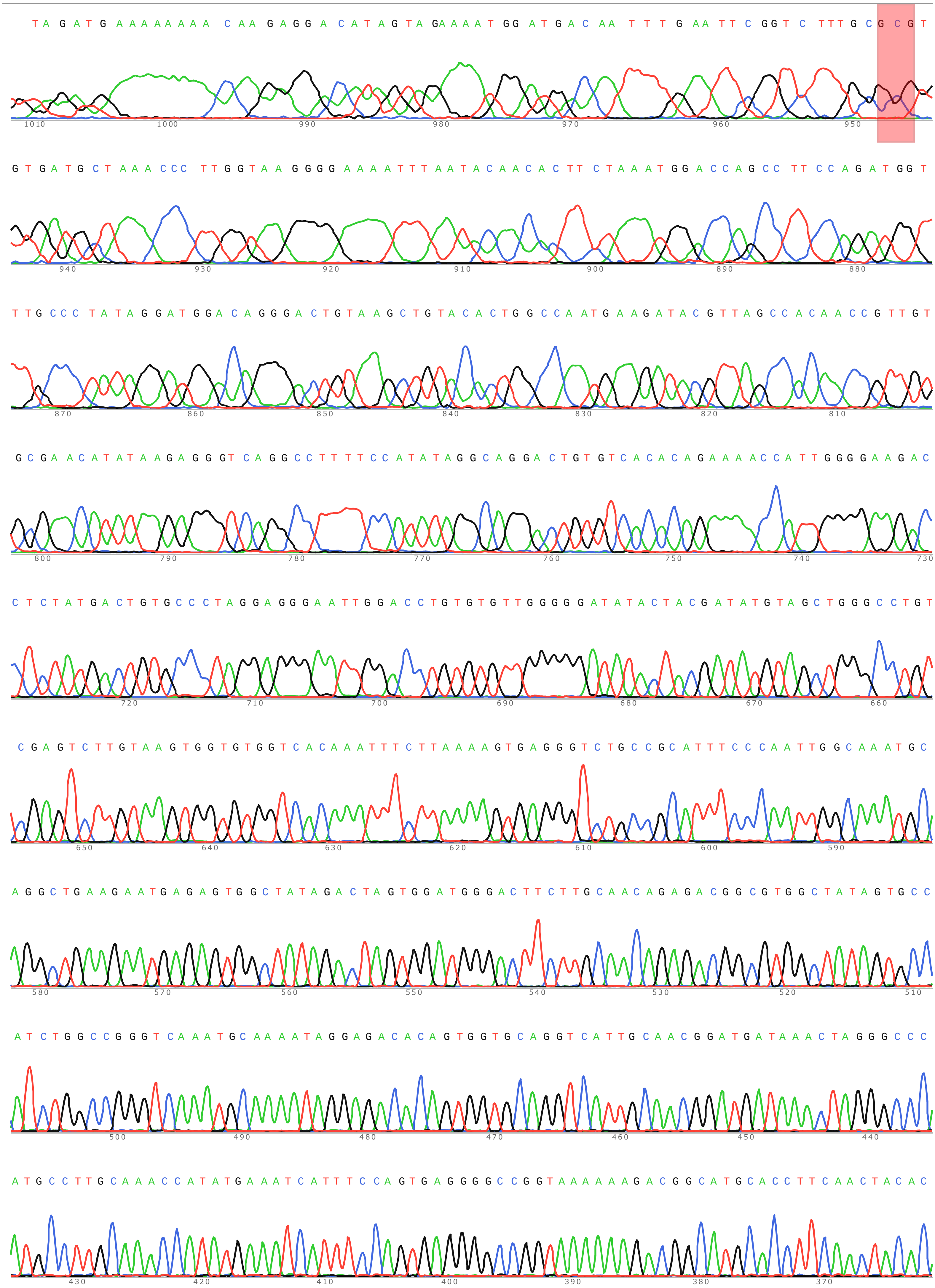
TAGATGAAAAAAAACAAGAGGACATAGTAGAAAATGGATGACAATTTGAATTCGGTCTTTGCGCGT
1010 1000 990 980 970 960 950
GTGATGCTAAACCCTTGGTAAGGGGAAAATTTAATACAACACTTCTAAATGGACCAGCCTTCCAGATGGT
940 930 920 910 900 890 880
TTGCCCTATAGGATGGACAGGGACTGTAAGCTGTACACTGGCCAATGAAGATACGTTAGCCACAACCGTTGT
870 860 850 840 830 820 810
GCGAACATATAAGAGGGTCAGGCCTTTTCCATATAGGCAGGACTGTGTCACACAGAAAACCATTGGGGAAGAC
800 790 780 770 760 750 740 730
CTCTATGACTGTGCCCTAGGAGGGAATTGGACCTGTGTGTTGGGGGATATACTACGATATGTAGCTGGGCCTGT
720 710 700 690 680 670 660
CGAGTCTTGTAAGTGGTGTGGTCACAAATTTCTTAAAAGTGAGGGTCTGCCGCATTTCCCAATTGGCAAATGC
650 640 630 620 610 600 590
AGGCTGAAGAATGAGAGTGGCTATAGACTAGTGGATGGGACTTCTTGCAACAGAGACGGCGTGGCTATAGTGCC
580 570 560 550 540 530 520 510
ATCTGGCCGGGTCAAATGCAAAATAGGAGACACAGTGGTGCAGGTCATTGCAACGGATGATAAACTAGGGCCC
500 490 480 470 460 450 440
ATGCCTTGCAAACCATATGAAATCATTTCCAGTGAGGGGCCGGTAAAAAAGACGGCATGCACCTTCAACTACAC
430 420 410 400 390 380 370

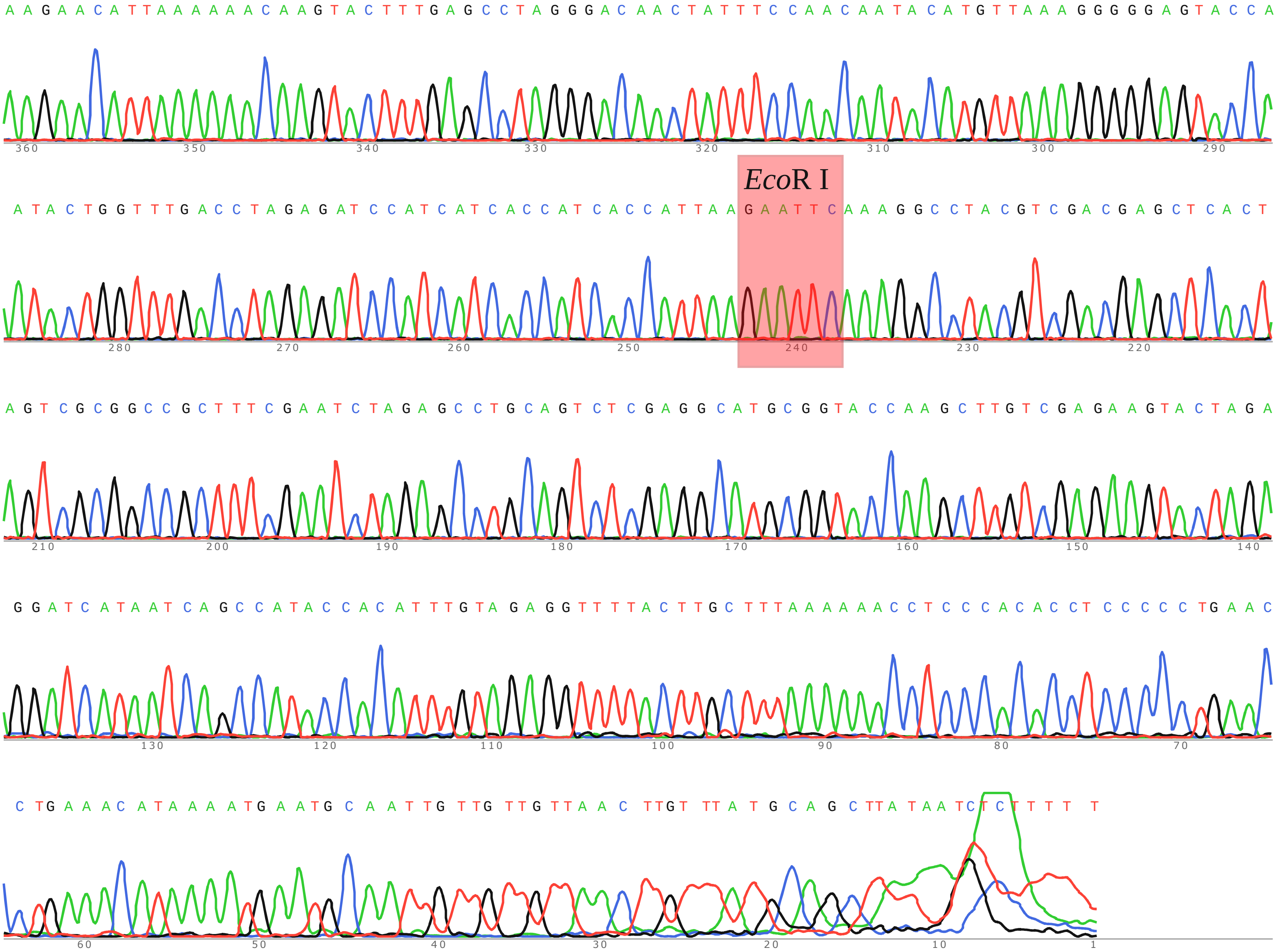
A A G A A C A T T A A A A A C A A G T A C T T T G A G C C T A G G G A C A A C T A T T T C C A A C A A T A C A T G T T A A A G G G G G A G T A C C A
EcoR I
A T A C T G G T T T G A C C T A G A G A T C C A T C A T C A C C A T C A C C A T T A A G A A T T C A A A G G C C T A C G T C G A C G A G C T C A C T
A G T C G C G G C C G C T T T C G A A T C T A G A G C C T G C A G T C T C G A G G C A T G C G G T A C C A A G C T T G T C G A G A A G T A C T A G A
G G A T C A T A A T C A G C C A T A C C A C A T T T G T A G A G G T T T T A C T T G C T T T A A A A A C C T C C C A C A C C T C C C C C T G A A C
C T G A A A C A T A A A A T G A A T G C A A T T G T T G T T G T T A A C T T G T T T A T G C A G C T T A T A A T C T C T T T T T

BEP-492-pFsat Bac1-BVDV-XH32-E2 (L107A)_poly-F_2023-07-28_H02.ab1 (1010 bases)

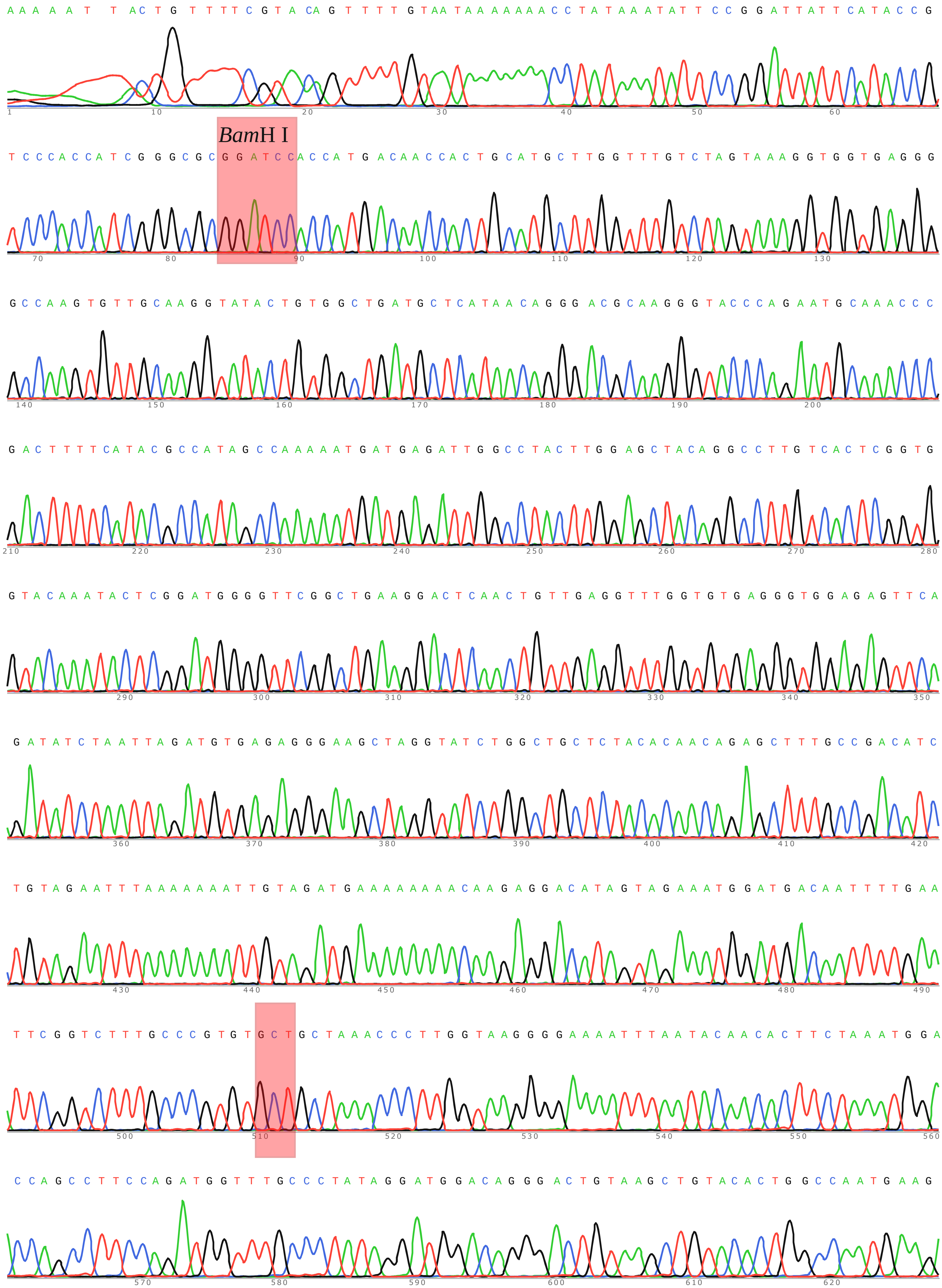

A T A C G T T A G C C A C A A C C G T T G T G C G A A C A T A T A A G A G G G T C A G G C C T T T T C C A T A T A G G C A G G A C T G

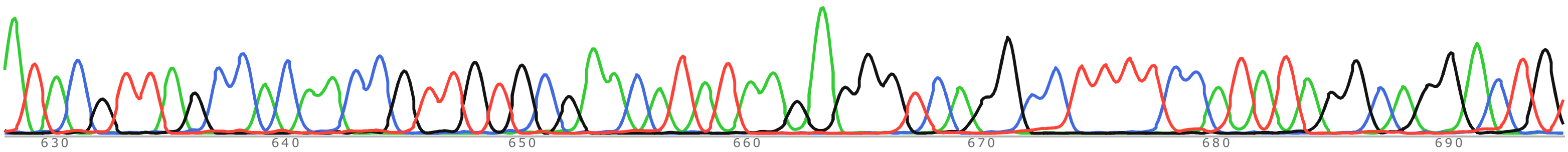

T G T C A C A C A G A A A A C C A T T G G G G A A G A C C T C T A T G A C T G T G C C C T A G G A G G G A A T T G G A C C T G T G T

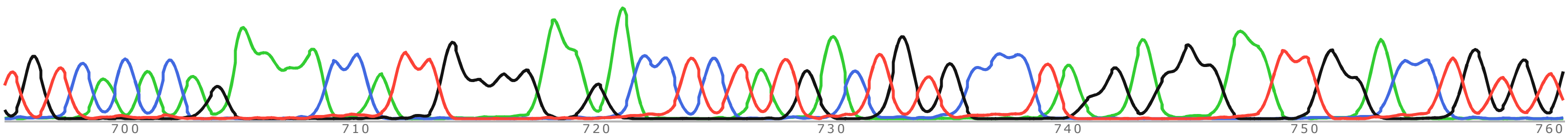

G T T G G G G G A T A T A C T A C G A T A T G T A G C T G G G C C T G T C G A G T C T T G T A A G T G G T G T G G T C A C A A A T

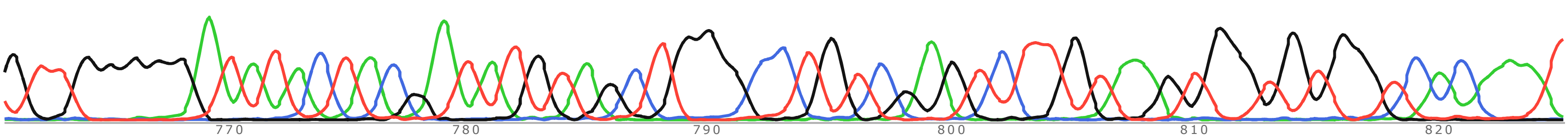

T T C T T A A A A G T G A G G G T C T G C C G C A T T T C C C A A T T G G C A A A T G C A G G C T G A A G A A T G A G A G T

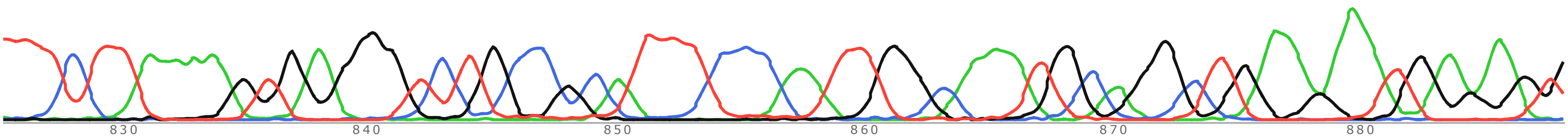

G G C T A T A G A C T A G T G G A T G G G A C T T C T T G C A A C A G A G A C G G C G T G G C T A T A G T G C C A T C

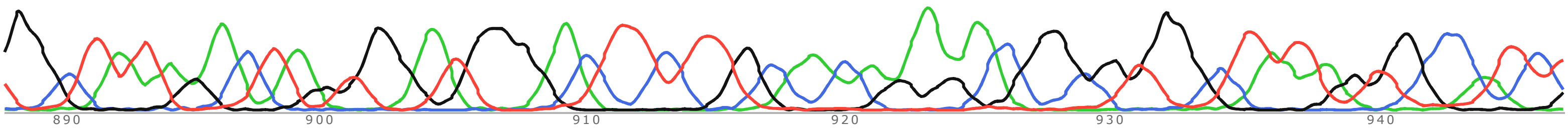

T G G G C C G G G T C A A A T G C A A A T A G G A G A C A C A G T G G T G C A G G T C A T T G C A C G G A T G A

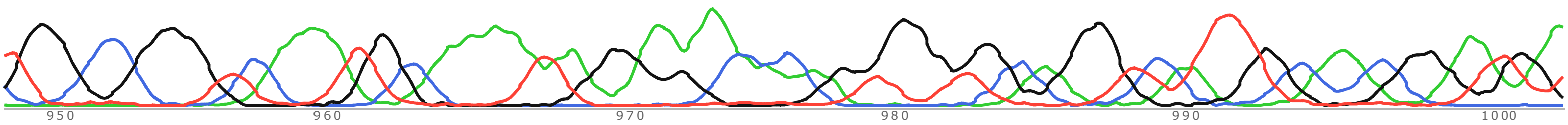

T A A A C T A G

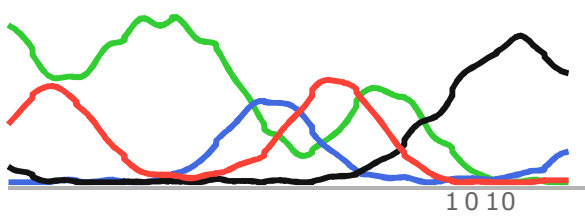

AAAATGTAGATGAAAAAAACAGAGGACATAGTAGAAATGGATGACAATTTGAATTCGGT

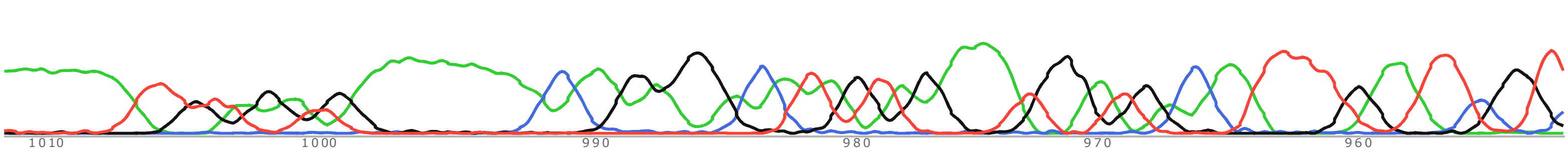

CTTTGCCCGTGTGCTGCTAAACCCTTGGTAAGGGGAAAATTTAATACAACACTTCTAAATGGACCA

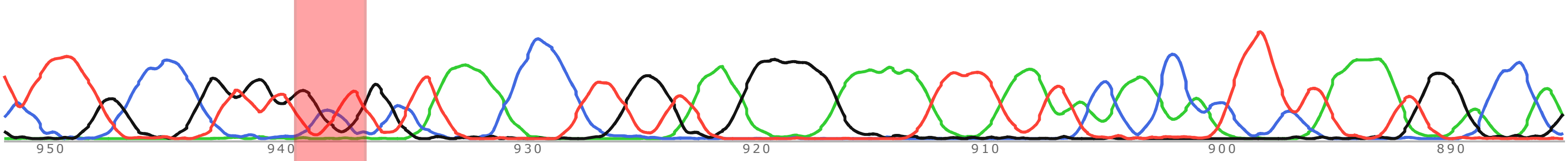

GCCTTCCAGATGGTTTGCCCTATAGGATGGACAGGGACTGTAAGCTGTACACTGGCCAATGAAGATAC

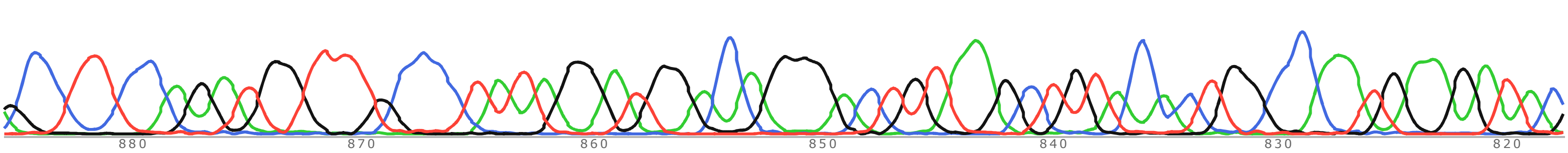

GTTAGCCACAACCGTTGTGCGAACATATAAGAGGGTCAGGCCTTTTCCATATAGGCAGGACTGTGTCAC

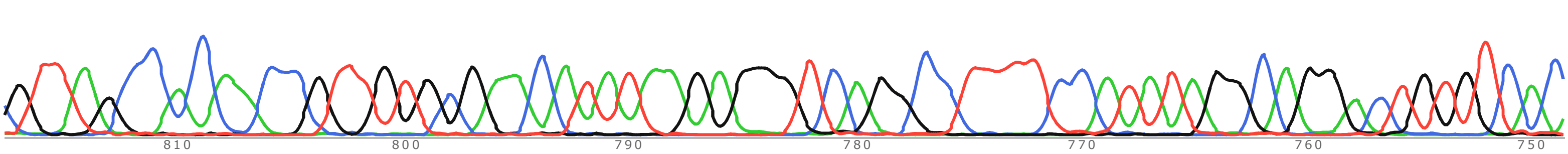

ACAGAAAACCATTGGGGAAGACCTCTATGACTGTGCCCTAGGAGGGAATTGGACCTGTGTGTTGGGGGA

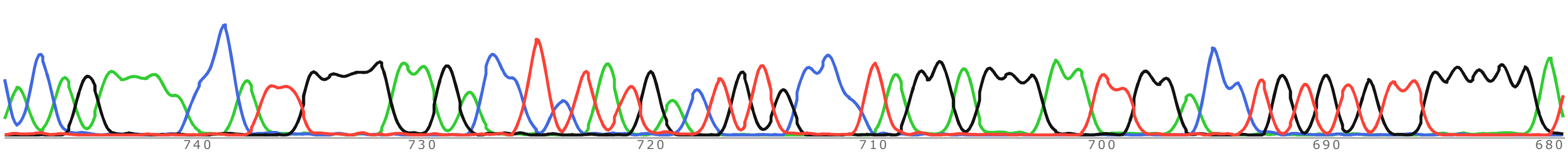

TATACTACGATATGTAGCTGGGCCTGTCGAGTCTTGTAAGTGGTGTGGTCACAAATTTCTTAAAAGTGAG

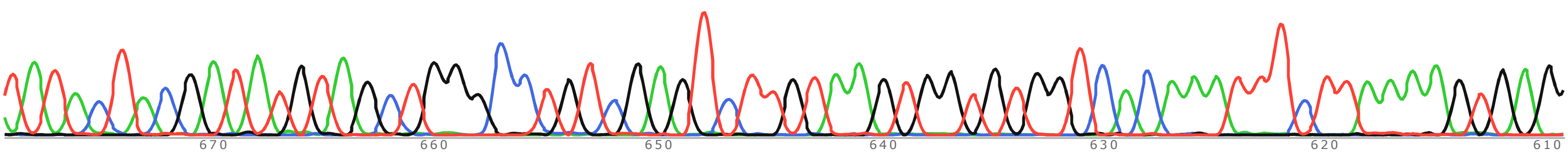

GGTCTGCCGCATTTCCCAATTGGCAAATGCAGGCTGAAGAATGAGAGTGGCTATAGACTAGTGGATGGGA

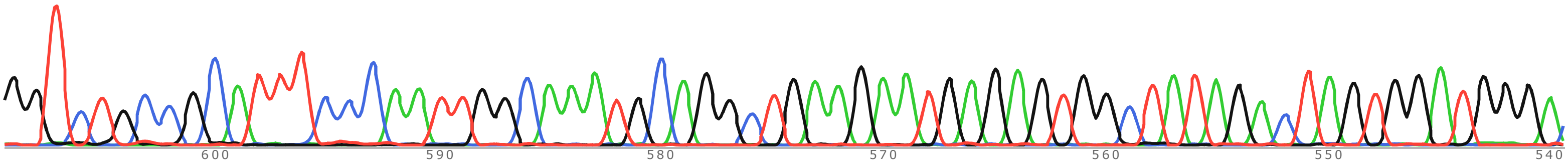

CTTCTTGCAACAGAGACGGCGTGGCTATAGTGCCATCTGGCCGGGTCAAATGCAAAATAGGAGACACAGT

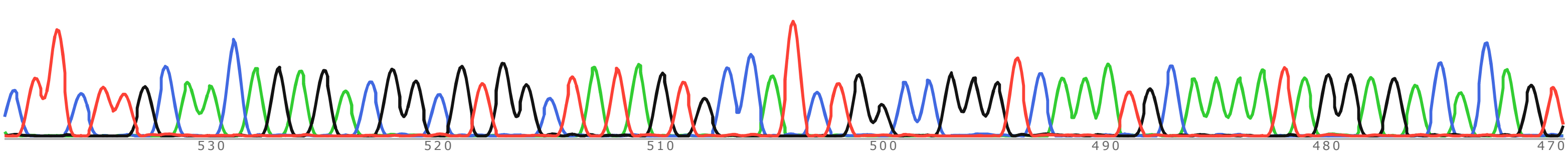

GGTGCAGGTCATTGCAACGGATGATAAACTAGGGCCCATGCCTTGCAAACCATATGAAATCATTTCCAGT

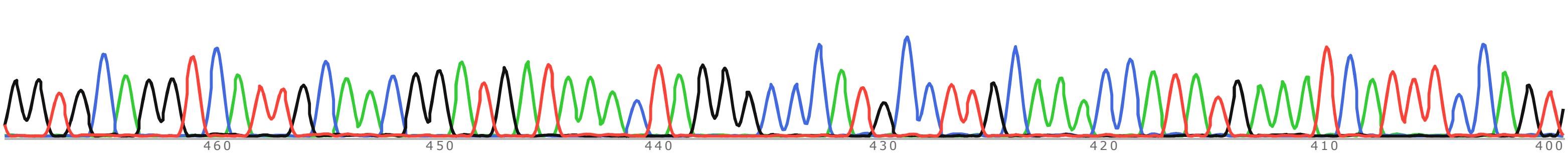

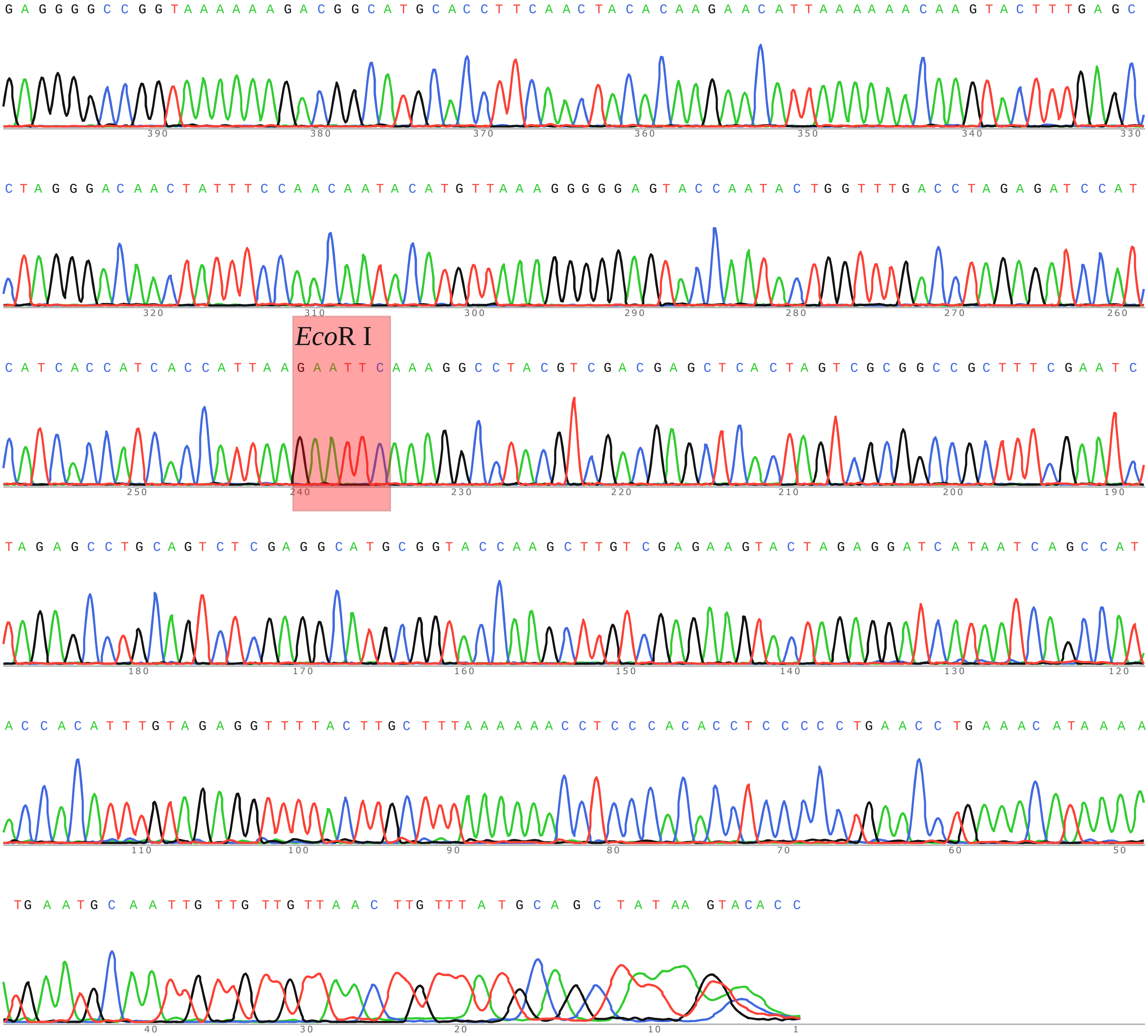
G A G G G G C C G G T A A A A A A G A C G G C A T G C A C C T T C A A C T A C A C A A G A A C A T T A A A A A A C A A G T A C T T T G A G C
C T A G G G A C A A C T A T T T C C A A C A A T A C A T G T T A A A G G G G G A G T A C C A A T A C T G G T T T G A C C T A G A G A T C C A T
EcoR I
C A T C A C C A T C A C C A T T A A G A A T T C A A A G G C C T A C G T C G A C G A G C T C A C T A G T C G C G G C C G C T T T C G A A T C
T A G A G C C T G C A G T C T C G A G G C A T G C G G T A C C A A G C T T G T C G A G A A G T A C T A G A G G A T C A T A A T C A G C C A T
A C C A C A T T T G T A G A G G T T T T A C T T G C T T T A A A A A C C T C C C A C A C C T C C C C C T G A A C C T G A A A C A T A A A A
TG A A T G C A A T T G T T G T T G T T A A C T T G T T T A T G C A G C T A T AA G T A C A C C

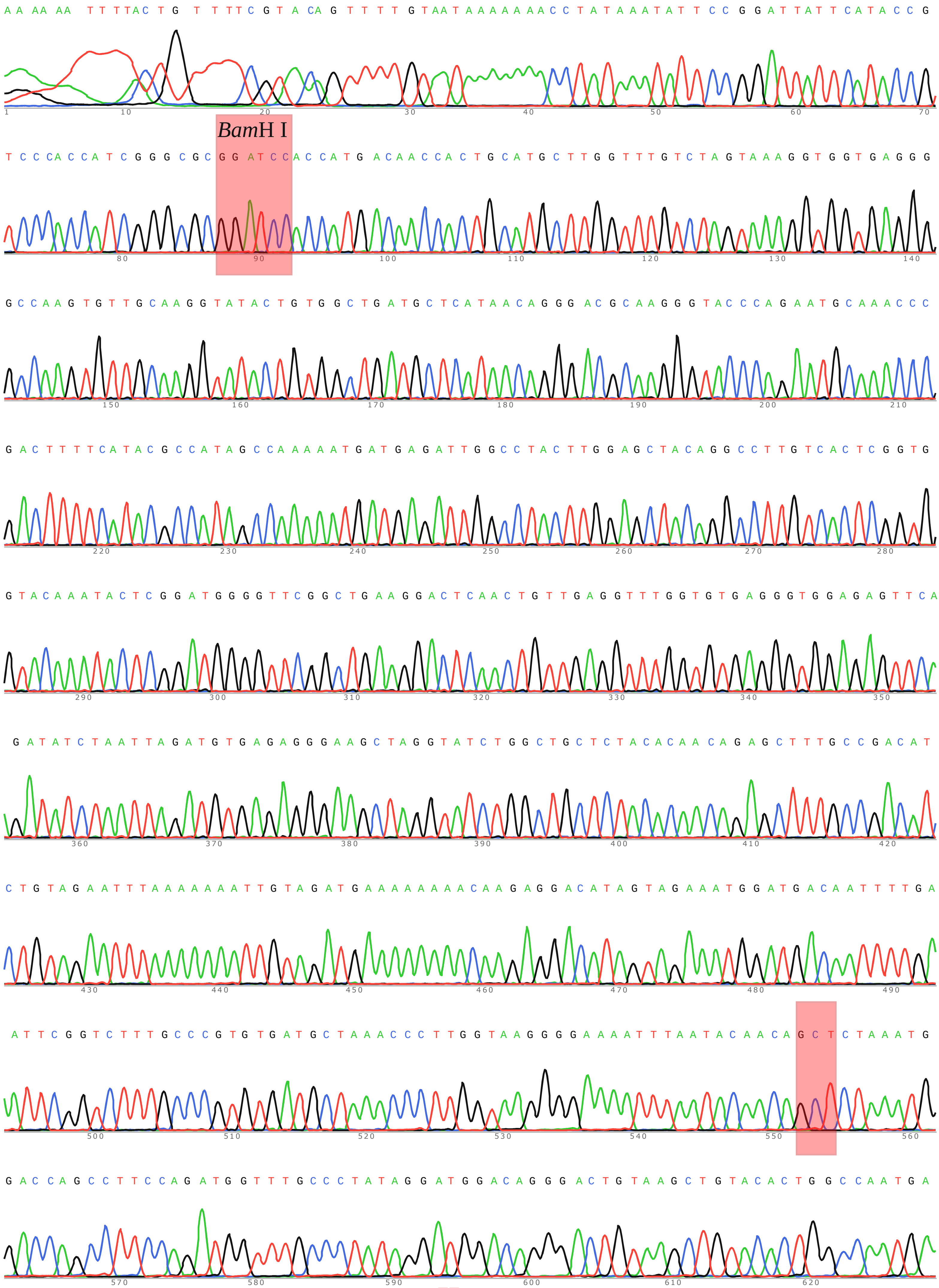
A A AA AA T T T T A C T G T T T T C G T A CA G T T T T G T A A T A A A A A A A C C T A T A A A T A T T C C G G A T T A T T C A T A C C G
*Bam*H I
T C C CA C C A T C G G G C G C G G A T C C A C C A T G A C A A C C A C T G C A T G C T T G G T T T G T C T A G T A A A G G T G G T G A G G G
G C C A A G T G T T G C A A G G T A T A C T G T G G C T G A T G C T C A T A A C A G G G A C G C A A G G G T A C C C A G A A T G C A A A C C C
G A C T T T T C A T A C G C C A T A G C C A A A A A T G A T G A G A T T G G C C T A C T T G G A G C T A C A G G C C T T G T C A C T C G G T G
G T A C A A A T A C T C G G A T G G G G T T C G G C T G A A G G A C T C A A C T G T T G A G G T T T G G T G T G A G G G T G G A G A G T T C A
G A T A T C T A A T T A G A T G T G A G A G G G A A G C T A G G T A T C T G G C T G C T C T A C A C A A C A G A G C T T T G C C G A C A T
C T G T A G A A T T T A A A A A A A T T G T A G A T G A A A A A A A A C A A G A G G A C A T A G T A G A A A T G G A T G A C A A T T T T G A
A T T C G G T C T T T G C C C G T G T G A T G C T A A A C C C T T G G T A A G G G G A A A A T T T A A T A C A A C A G C T C T A A A T G
G A C C A G C C T T C C A G A T G G T T T G C C C T A T A G G A T G G A C A G G G A C T G T A A G C T G T A C A C T G G C C A A T G A

A G A T A C G T T A G C C A C A A C C G T T G T G C G A A C A T A T A A G A G G G T C A G G C C T T T T C C A T A T A G G C A G G A C

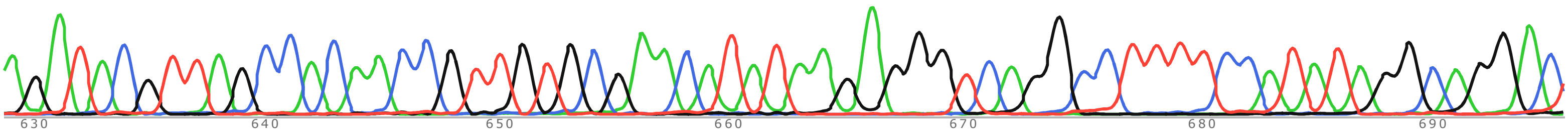

T G T G T C A C A C A G A A A A C C A T T G G G G A A G A C C T C T A T G A C T G T G C C C T A G G A G G G A A T T G G A C C T G

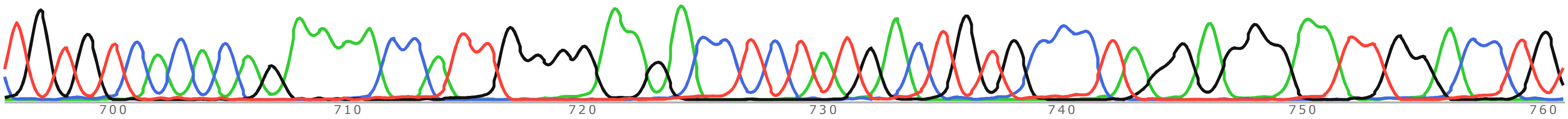

T G T G T T G G G G G A T A T A C T A C G A T A T G T A G C T G G G C C T G T C G A G T C T T G T A A G T G G T G T G G T C A C

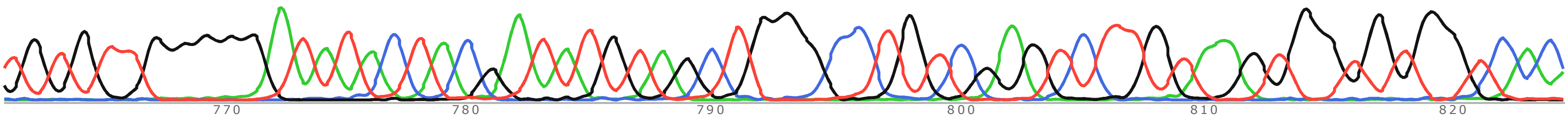

A A A T T T C T T A A A A G T G A G G G T C T G C C G C A T T T C C C A A T T G G C A A A T G C A G G C T G A A G A A T G A

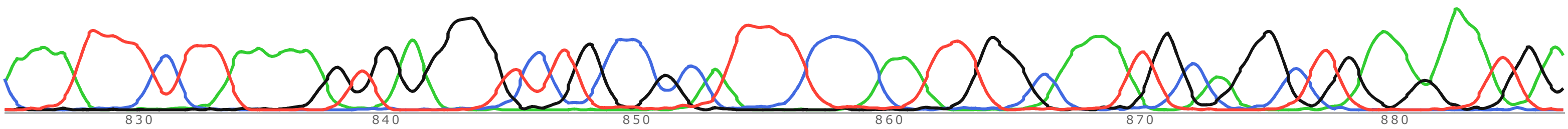

G A G T G G C T A T A G A C T A G T G G A T G G G A C T T C T T G C A A C A G A G A C G G C G T G G C T A T A G T G

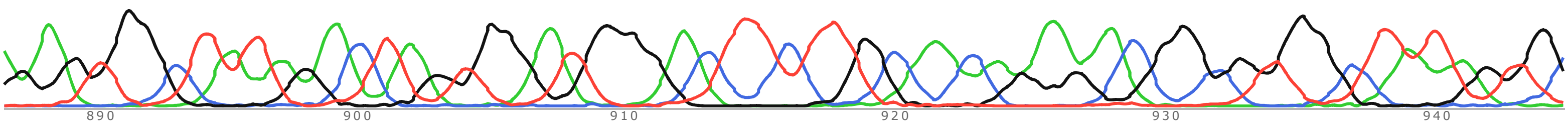

C C C A T C T G G G C C G G G T C A A A T G C A A A T A G G A G A C A C A G T G G T G C A G G T C A T T G C A

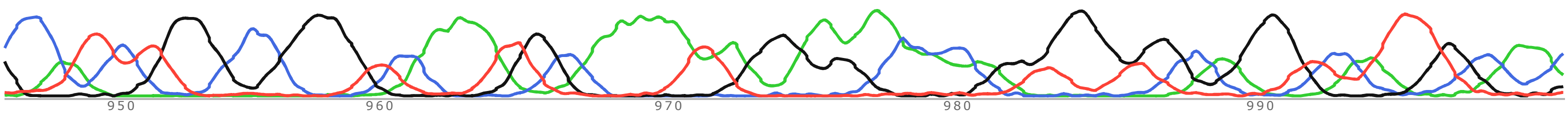

C G G A T G A T A A C

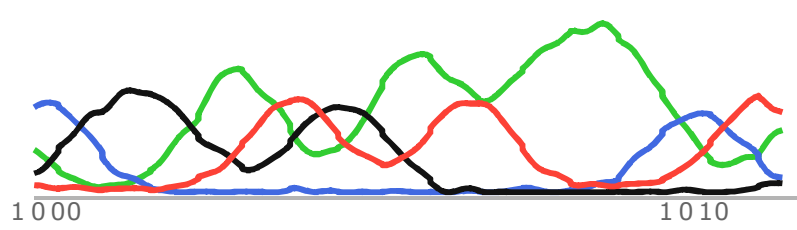

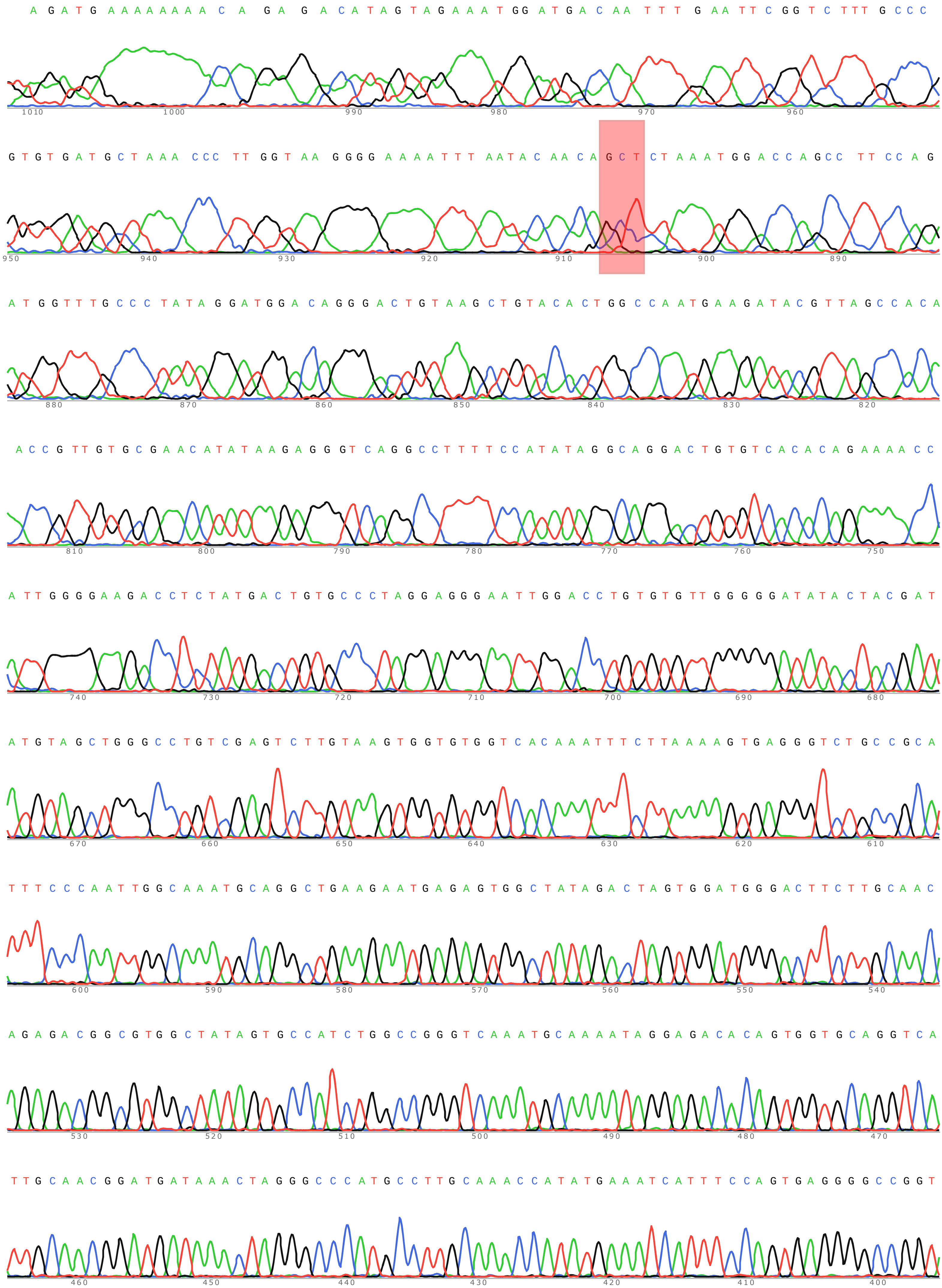
AGATGAAAAAAAACAGAGACATAGTAGAAATGGATGACAATTTGAATTCGGTCTTTGCCC
1010 1000 990 980 970 960
GTGTGATGCTAAACCCTTGGTAAGGGGAAAATTTAATACAACAGCTCTAAATGGACCAGCCTTCCAG
950 940 930 920 910 900 890
ATGGTTTGCCCTATAGGATGGACAGGGACTGTAAGCTGTACACTGGCCAATGAAGATACGTTAGCCACA
880 870 860 850 840 830 820
ACCGTTGTGCGAACATATAAGAGGGTCAGGCCTTTTCCATATAGGCAGGACTGTGTCACACAGAAAACC
810 800 790 780 770 760 750
ATTGGGGAAGACCTCTATGACTGTGCCCTAGGAGGGAATTGGACCTGTGTGTTGGGGGATATACTACGAT
740 730 720 710 700 690 680
ATGTAGCTGGGCCTGTCGAGTCTTGTAAGTGGTGTGGTCACAAATTTCTTAAAAGTGAGGGTCTGCCGCA
670 660 650 640 630 620 610
TTTCCCAATTGGCAAATGCAGGCTGAAGAATGAGAGTGGCTATAGACTAGTGGATGGGACTTCTTGCAAC
600 590 580 570 560 550 540
AGAGACGGCGTGGCTATAGTGCCATCTGGCCGGGTCAAATGCAAAATAGGAGACACAGTGGTGCAGGTCA
530 520 510 500 490 480 470
TTGCAACGGATGATAAACTAGGGCCCATGCCTTGCAAACCATATGAAATCATTTCCAGTGAGGGGCCGGT
460 450 440 430 420 410 400

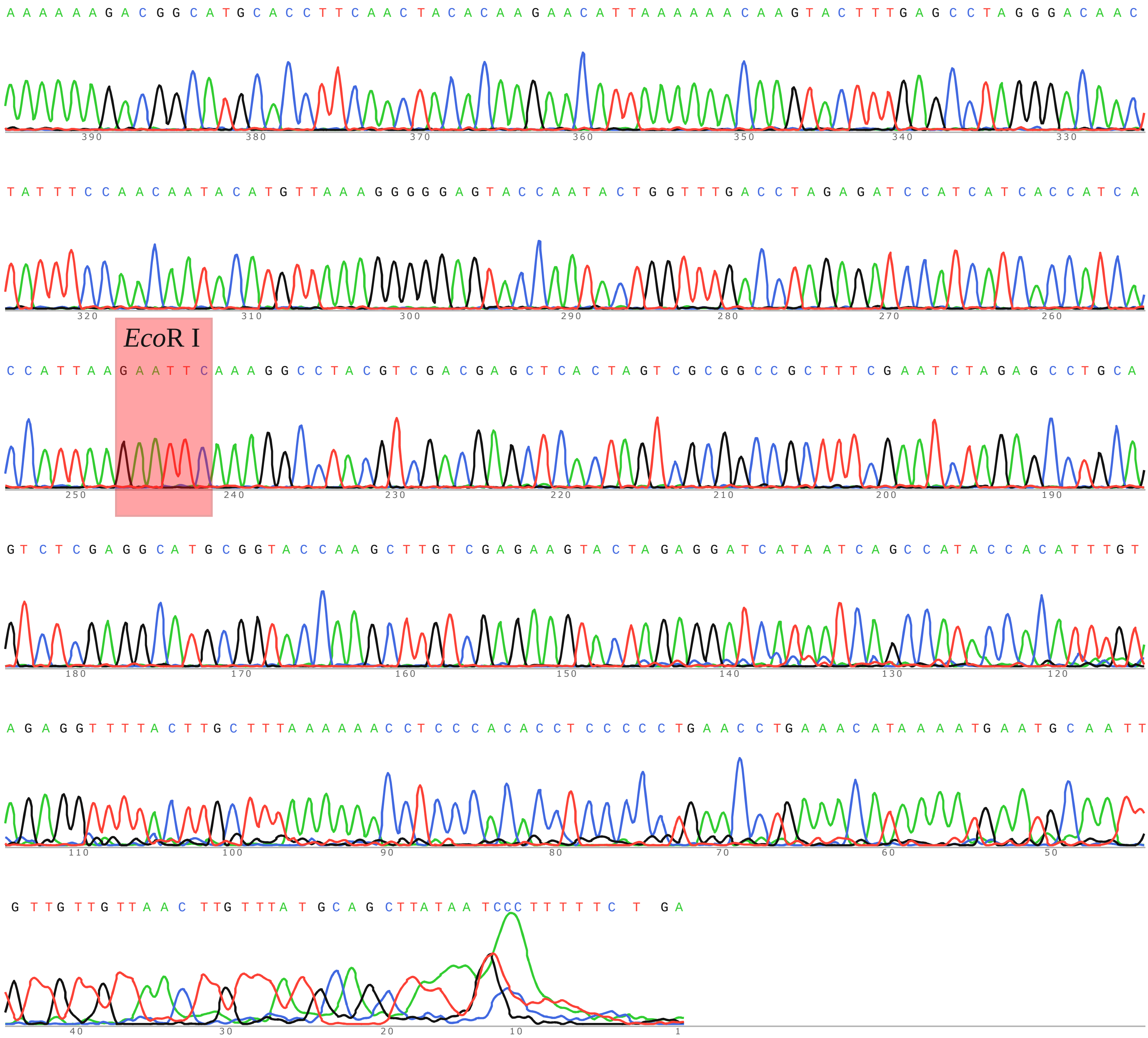
A A A A A A G A C G G C A T G C A C C T T C A A C T A C A C A A G A A C A T T A A A A A A C A A G T A C T T T G A G C C T A G G G A C A A C
390 380 370 360 350 340 330
T A T T T C C A A C A A T A C A T G T T A A A G G G G G A G T A C C A A T A C T G G T T T G A C C T A G A G A T C C A T C A T C A C C A T C A
320 310 300 290 280 270 260
*Eco*R I
C C A T T A A G A A T T C A A A G G C C T A C G T C G A C G A G C T C A C T A G T C G C G G C C G C T T T C G A A T C T A G A G C C T G C A
250 240 230 220 210 200 190
G T C T C G A G G C A T G C G G T A C C A A G C T T G T C G A G A A G T A C T A G A G G A T C A T A A T C A G C C A T A C C A C A T T T G T
180 170 160 150 140 130 120
A G A G G T T T T A C T T G C T T T A A A A A C C T C C C A C A C C T C C C C C T G A A C C T G A A A C A T A A A A T G A A T G C A A T T
110 100 90 80 70 60 50
G T T G T T G T T A A C T T G T T T A T G C A G C T T A T A A T C C C T T T T T C T G A
40 30 20 10 1

BEP-494-pFsat Bac1-BVDV-XH32-E2 (L121A)_poly-F_2023-07-28_E04.ab1 (1010 bases)

AAGTTAATTTACTGTTTTCGTACAGTTTTGTAATAAAAAAACCTATAAATATTCCGGATTATTCATACCG

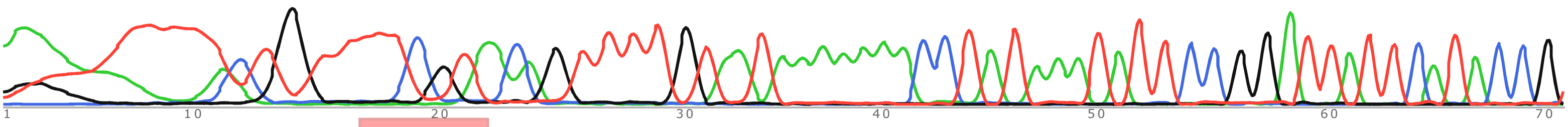

TCCCACCATCGGGCGCGGATCCACCATGACAACCACTGCATGCTTGGTTTGTCTAGTAAAGGTGGTGAGGG

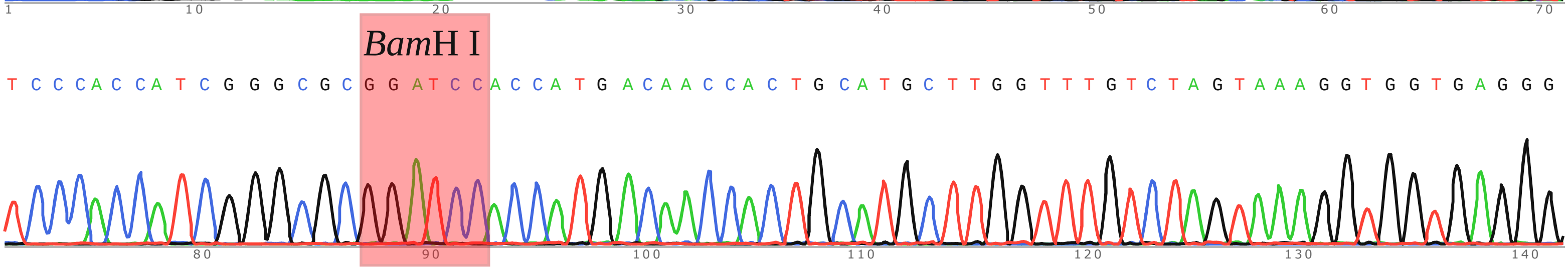

GCCAAGTGTTGCAAGGTATACTGTGGCTGATGCTCATAACAGGGACGCAAGGGTACCCAGAATGCAAACCC

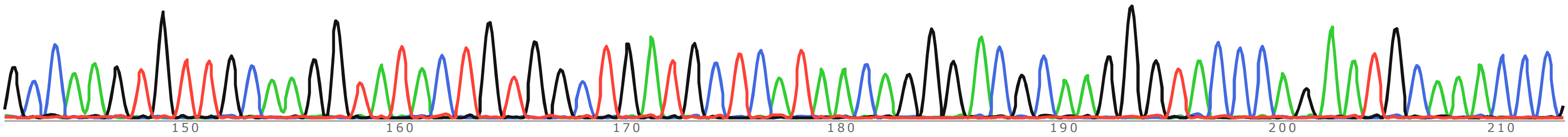

GACTTTTCATACGCCATAGCCAAAAATGATGAGATTGGCCTACTTGGAGCTACAGGCCTTGTCACTCGGTG

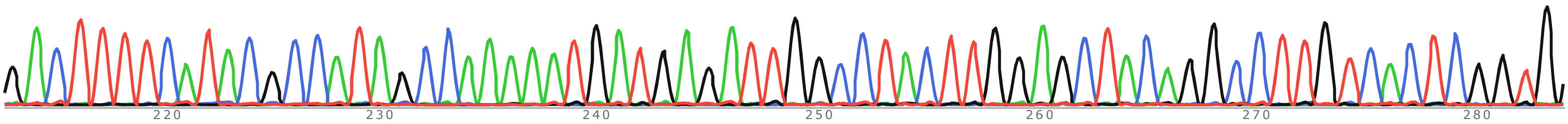

GTACAAATACTCGGATGGGGTTCGGCTGAAGGACTCAACTGTTGAGGTTTGGTGTGAGGGTGGAGAGTTC

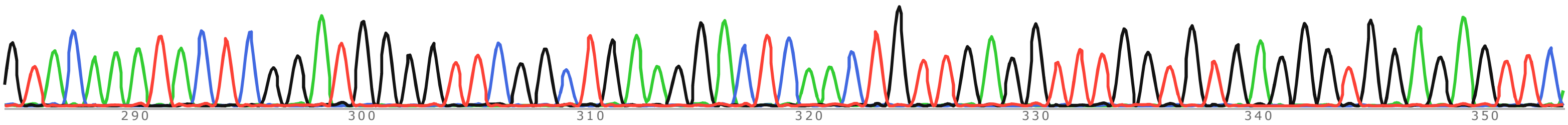

AGATATCTAATTAGATGTGAGAGGGAAGCTAGGTATCTGGCTGCTCTACACAACAGAGCTTTGCCGACAT

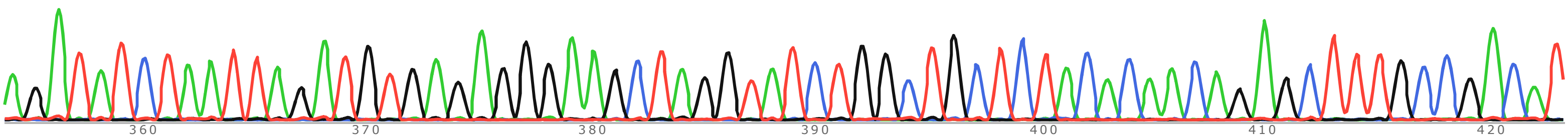

CTGTAGAATTTAAAAAAATTGTAGATGAAAAAAAACAAGAGGACATAGTAGAAATGGATGACAATTTTG

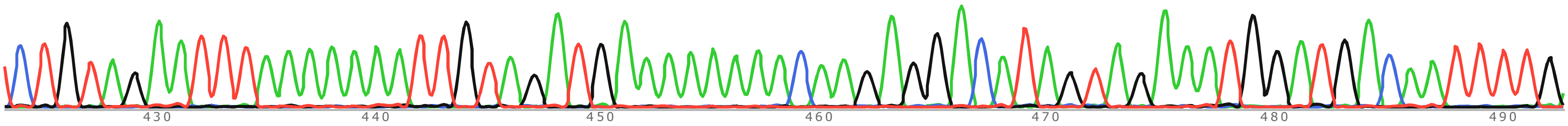

AATTCGGTCTTTGCCCGTGTGATGCTAAACCCTTGGTAAGGGGAAAATTTAATACAACACTTGCAAAT

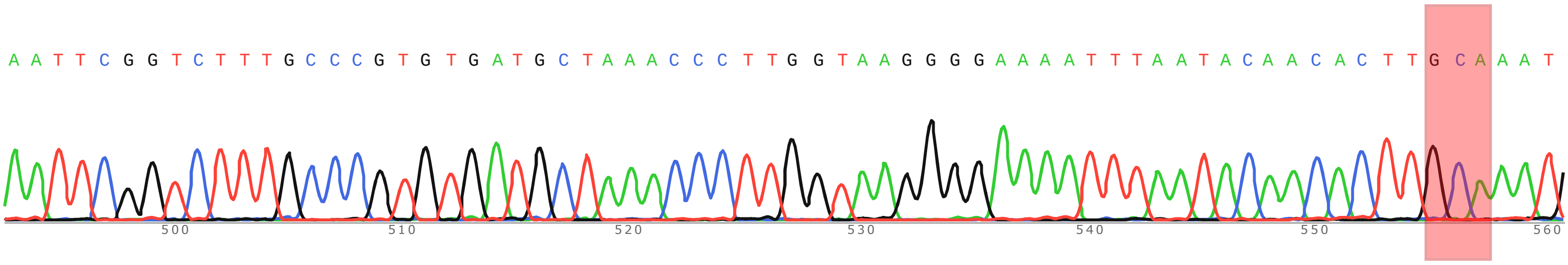

GGACCAGCCTTCCAGATGGTTTGCCCTATAGGATGGACAGGGACTGTAAGCTGTACACTGGCCAATG

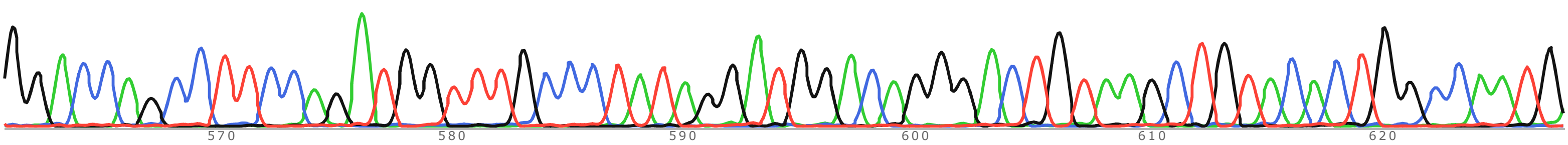

A A G A T A C G T T A G C C A C A A C C G T T G T G C G A A C A T A T A A G A G G G T C A G G C C T T T T C C A T A T A G G C A G G

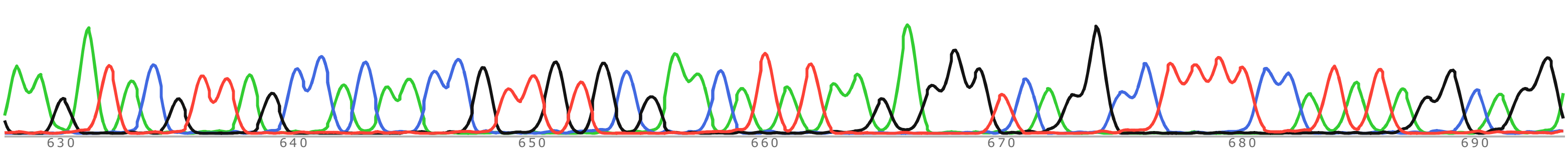

A C T G T G T C A C A C A G A A A A C C A T T G G G G A A G A C C T C T A T G A C T G T G C C C T A G G A G G G A A T T G G A C C

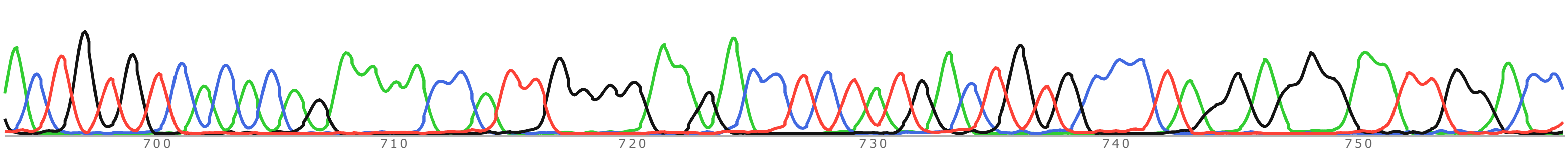

T G T G T G T T G G G G G A T A T A C T A C G A T A T G T A G C T G G G C C T G T C G A G T C T T G T A A G T G G T G T G G

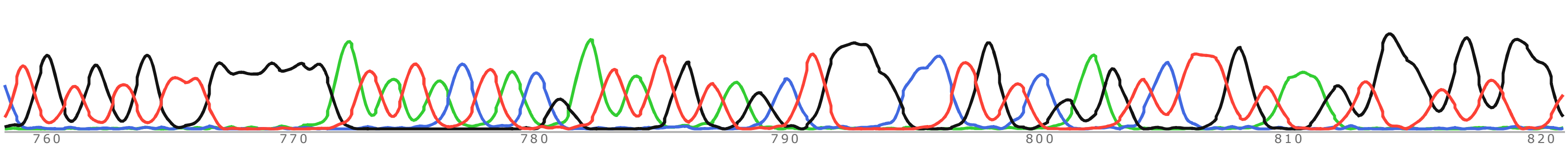

T C A C A A A T T T C T T A A A A G T G A G G G T C T G C C G C A T T T C C C A A T T G G C A A A T G C A G G C T G A A G

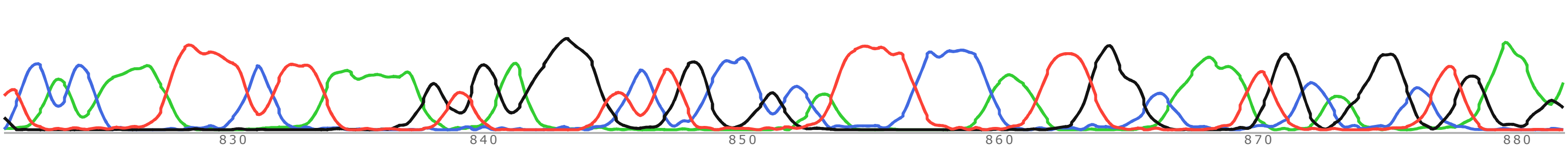

A A T G A G A G T G G C T A T A G A C T A G T G G A T G G G A C T T C T T G C A A C A G A G A C G G C G T G G C T

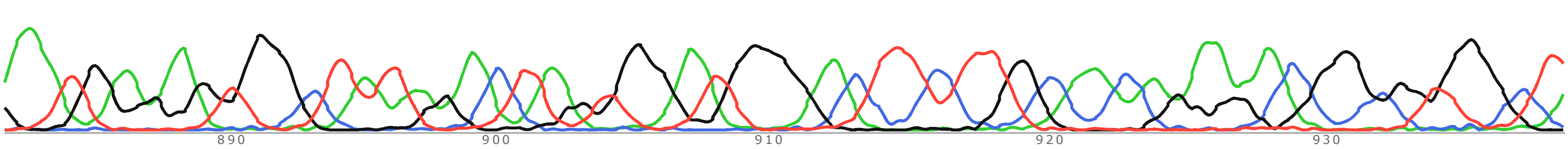

A T A G T G C C A T C T G G G C C G G G T C A A A T G C A A A A T A G G A G A C A C A G T G G T G C A G G T

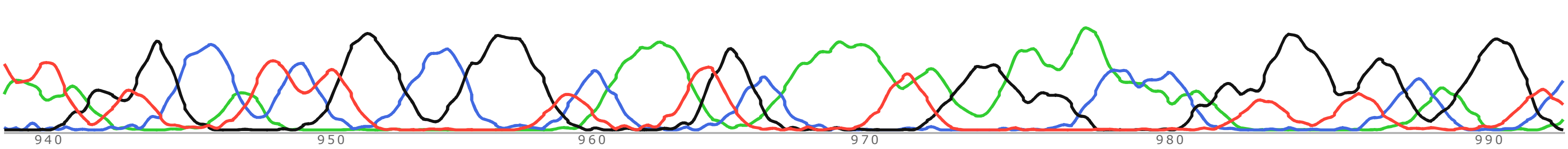

C A T T G C A C G G A T G A T A A C

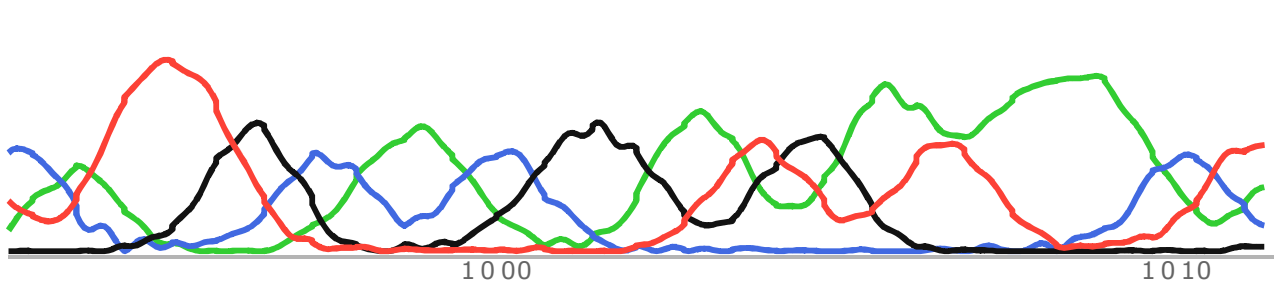

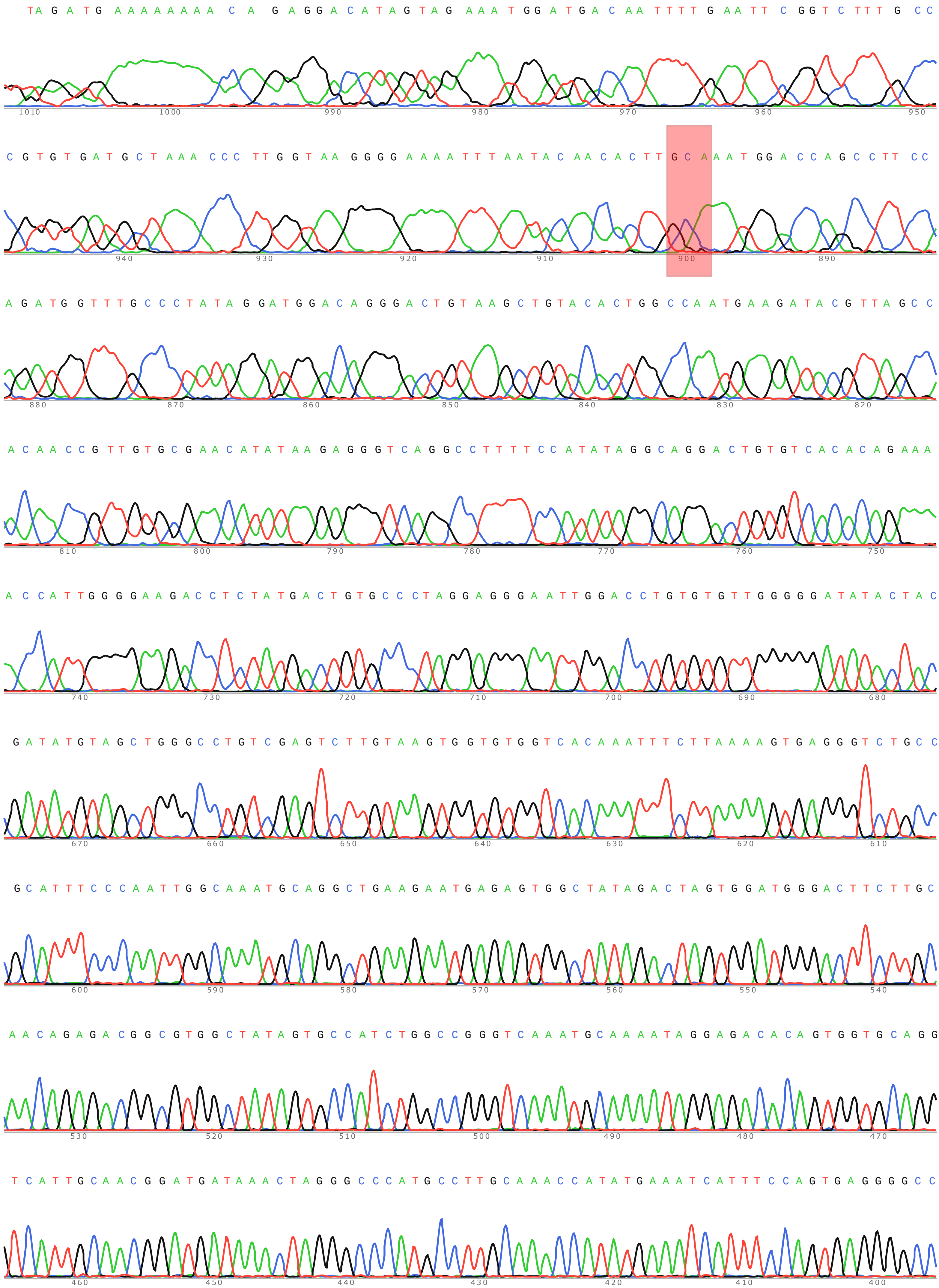
TAGATGAAAAAAAACAGAGGACATAGTAGAAATGGATGACAATTTTGAATTCGGTCTTTGCC
1010 1000 990 980 970 960 950
CGTGTGATGCTAAACCCTTGGTAAGGGGAAAATTTAATACAACACTTGCAAATGGACCAGCCTTCC
940 930 920 910 900 890
AGATGGTTTGCCCTATAGGATGGACAGGGACTGTAAGCTGTACACTGGCCAATGAAGATACGTTAGCC
880 870 860 850 840 830 820
ACAACCGTTGTGCGAACATATAAGAGGGTCAGGCCTTTTCCATATAGGCAGGACTGTGTCACACAGAAA
810 800 790 780 770 760 750
ACCATTGGGGAAGACCTCTATGACTGTGCCCTAGGAGGGAATTGGACCTGTGTGTTGGGGGATATACTAC
740 730 720 710 700 690 680
GATATGTAGCTGGGCCTGTCGAGTCTTGTAAGTGGTGTGGTCACAAATTTCTTAAAAGTGAGGGTCTGCC
670 660 650 640 630 620 610
GCATTTCCCAATTGGCAAATGCAGGCTGAAGAATGAGAGTGGCTATAGACTAGTGGATGGGACTTCTTGC
600 590 580 570 560 550 540
AACAGAGACGGCGTGGCTATAGTGCCATCTGGCCGGGTCAAATGCAAAATAGGAGACACAGTGGTGCAGG
530 520 510 500 490 480 470
TCATTGCAACGGATGATAAACTAGGGCCCATGCCTTGCAAACCATATGAAATCATTTCCAGTGAGGGGCC
460 450 440 430 420 410 400

G G T A A A A A A G A C G G C A T G C A C C T T C A A C T A C A C A A G A A C A T T A A A A A A C A A G T A C T T T G A G C C T A G G G A C

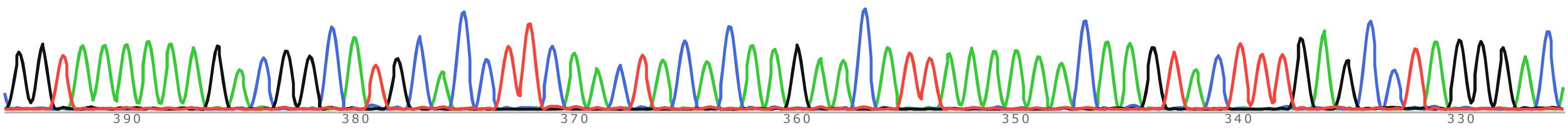

A A C T A T T T C C A A C A A T A C A T G T T A A A G G G G G A G T A C C A A T A C T G G T T T G A C C T A G A G A T C C A T C A T C A C C A

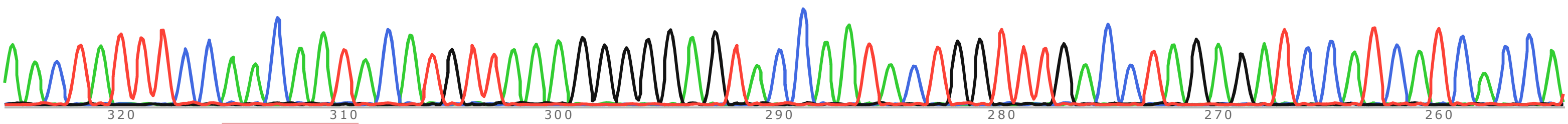

T C A C C A T T A A G A A T T C A A A G G C C T A C G T C G A C G A G C T C A C T A G T C G C G G C C G C T T T C G A A T C T A G A G C C T

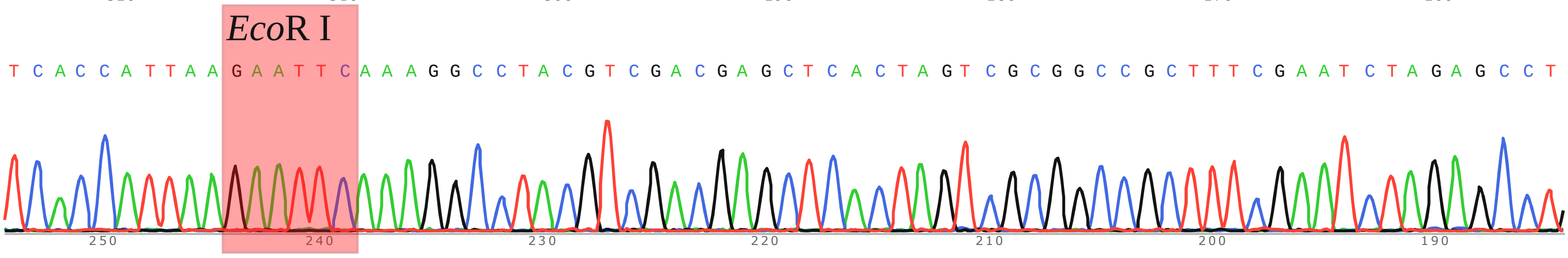

G C A G T C T C G A G G C A T G C G G T A C C A A G C T T G T C G A G A A G T A C T A G A G G A T C A T A A T C A G C C A T A C C A C A T T

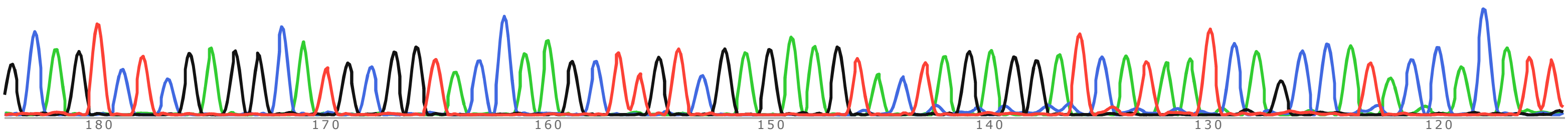

T G T A G A G G T T T T A C T T G C T T T A A A A A A C C T C C C A C A C C T C C C C C T G A A C C T G A A A C A T A A A A T G A A T G C A

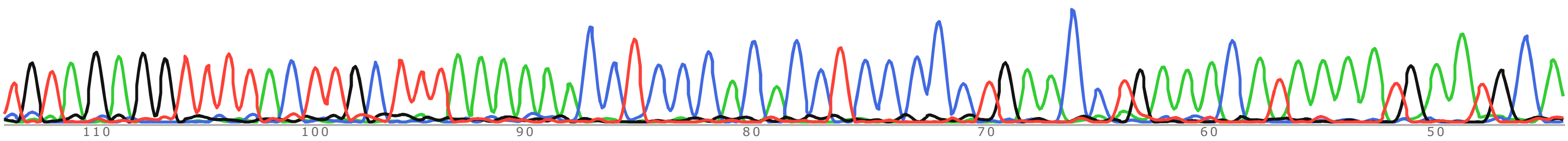

A T T G T T G T T G T T A A C T T G T T T A T T G C A G C T T A T A A T C C C T T C C T

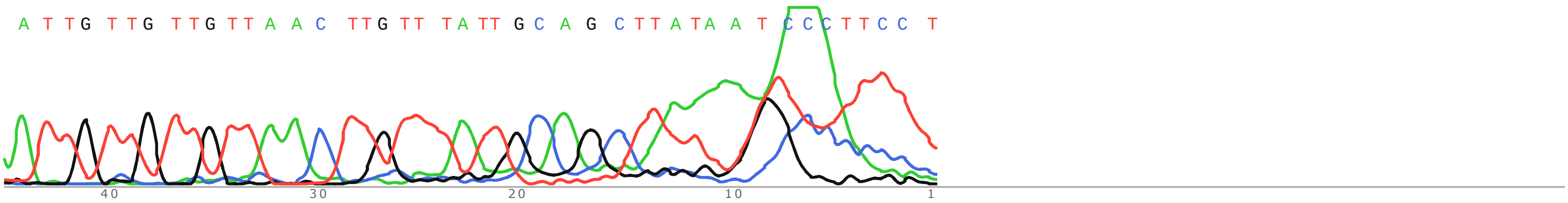

84

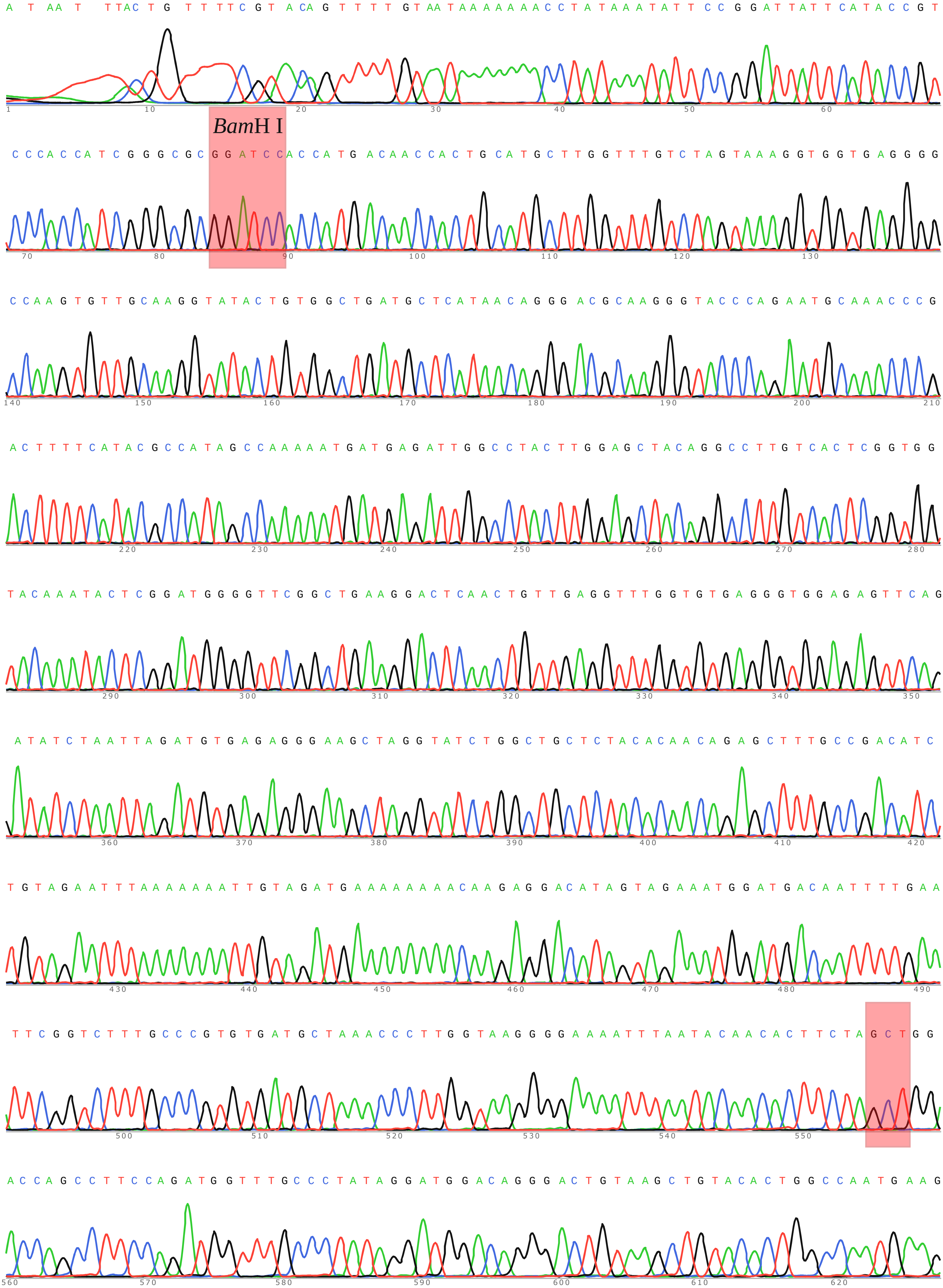
ATAATTTACTGTTTTCGTACAGTTTTGTAATAAAAAAACCTATAAATATTCCGGATTATTCATACCGT
BamH I
CCCACCATCGGGCGCGGATCCACCATGACAACCACTGCATGCTTGGTTTGTCTAGTAAAGGTGGTGAGGGG
CCAAGTGTTGCAAGGTATACTGTGGCTGATGCTCATAACAGGGACGCAAGGGTACCCAGAATGCAAACCCG
ACTTTTCATACGCCATAGCCAAAAATGATGAGATTGGCCTACTTGGAGCTACAGGCCTTGTCACTCGGTGG
TACAAATACTCGGATGGGGTTCGGCTGAAGGACTCAACTGTTGAGGTTTGGTGTGAGGGTGGAGAGTTCAG
ATATCTAATTAGATGTGAGAGGGAAGCTAGGTATCTGGCTGCTCTACACAACAGAGCTTTGCCGACATC
TGTAGAATTTAAAAAAATTGTAGATGAAAAAAAACAAGAGGACATAGTAGAAATGGATGACAATTTTGAA
TTCGGTCTTTTGCCCGTGTGATGCTAAACCCTTGGTAAGGGGAAAATTTAATACAACACTTCTAGCTGG
ACCAGCCTTCCAGATGGTTTGCCCTATAGGATGGACAGGGACTGTAAGCTGTACACTGGCCAATGAAG

A T A C G T T A G C C A C A A C C G T T G T G C G A A C A T A T A A G A G G G T C A G G C C T T T T C C A T A T A G G C A G G A C T

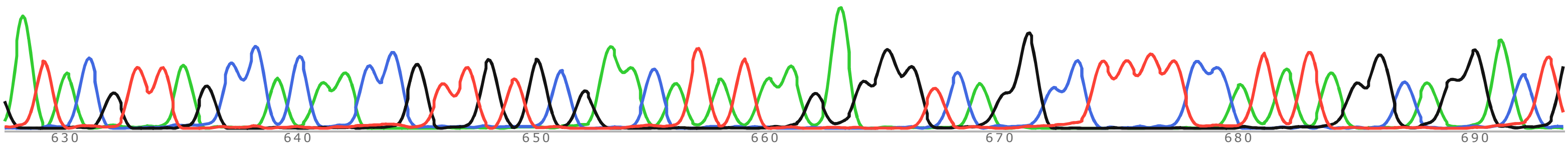

G T G T C A C A C A G A A A A C C A T T G G G G A A G A C C T C T A T G A C T G T G C C C T A G G A G G G A A T T G G A C C T G T G

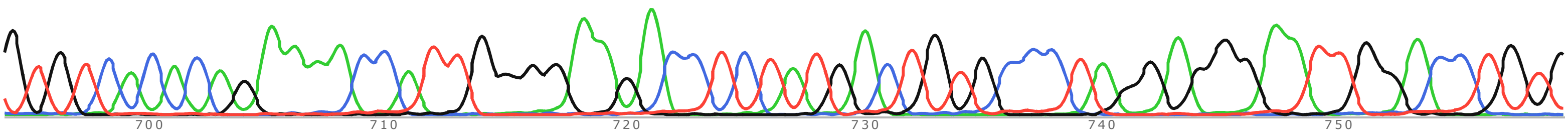

T G T T G G G G G A T A T A C T A C G A T A T G T A G C T G G G C C T G T C G A G T C T T G T A A G T G G T G T G G T C A C A

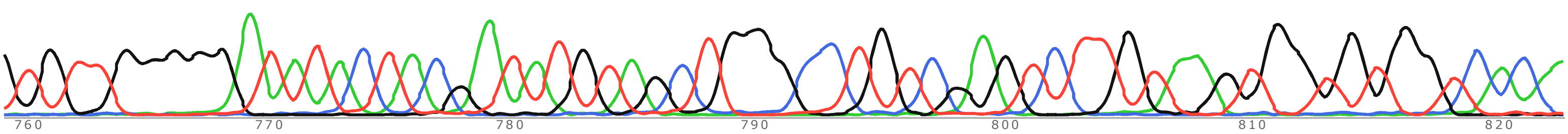

A A T T T C T T A A A A G T G A G G G T C T G C C G C A T T T C C C A A T TGGG C A A A T G C A GG C T G A A G AA T G A G

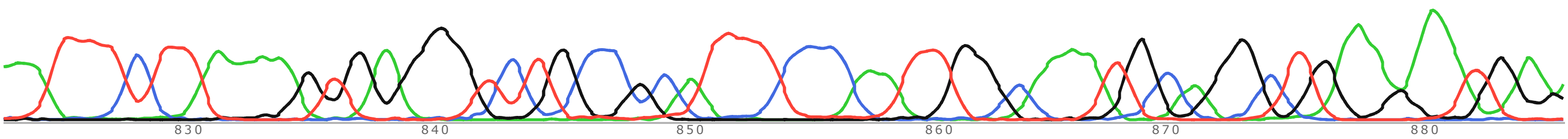

A G T GG C T A T A G A C T A G TGGG A T G G G A C TT C TTT G C A A C A G A G A C G G C G T GG C T A T A G T G C

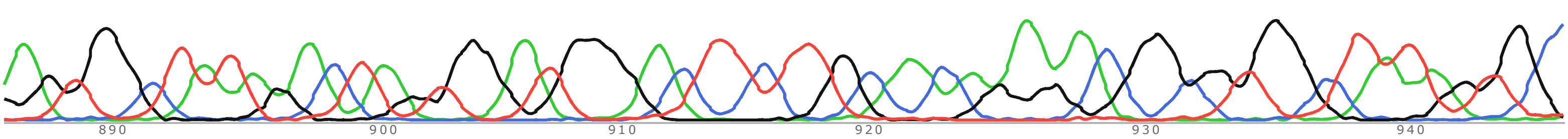

C A T C TGGG CC G G G T C A A A T G C AA AA T A GG A G A C A C A G TGG T G C A GG T C A TT G C A C

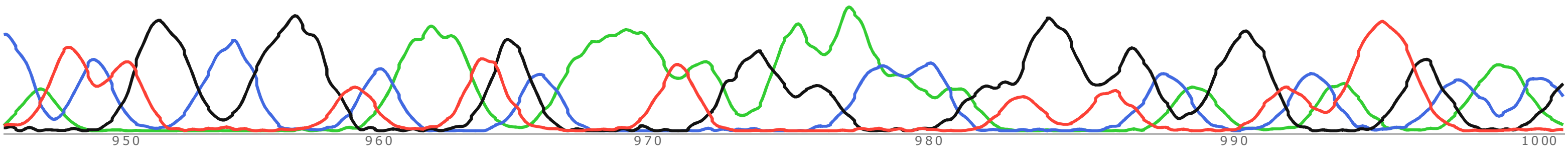

GG A T G A T A A C

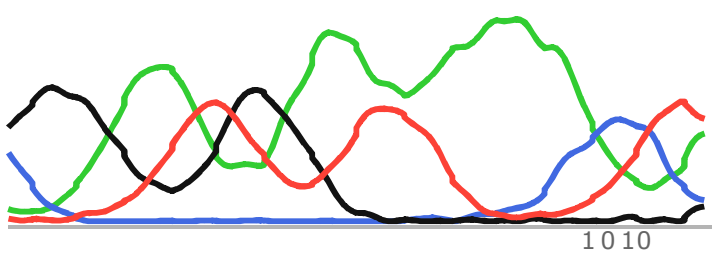

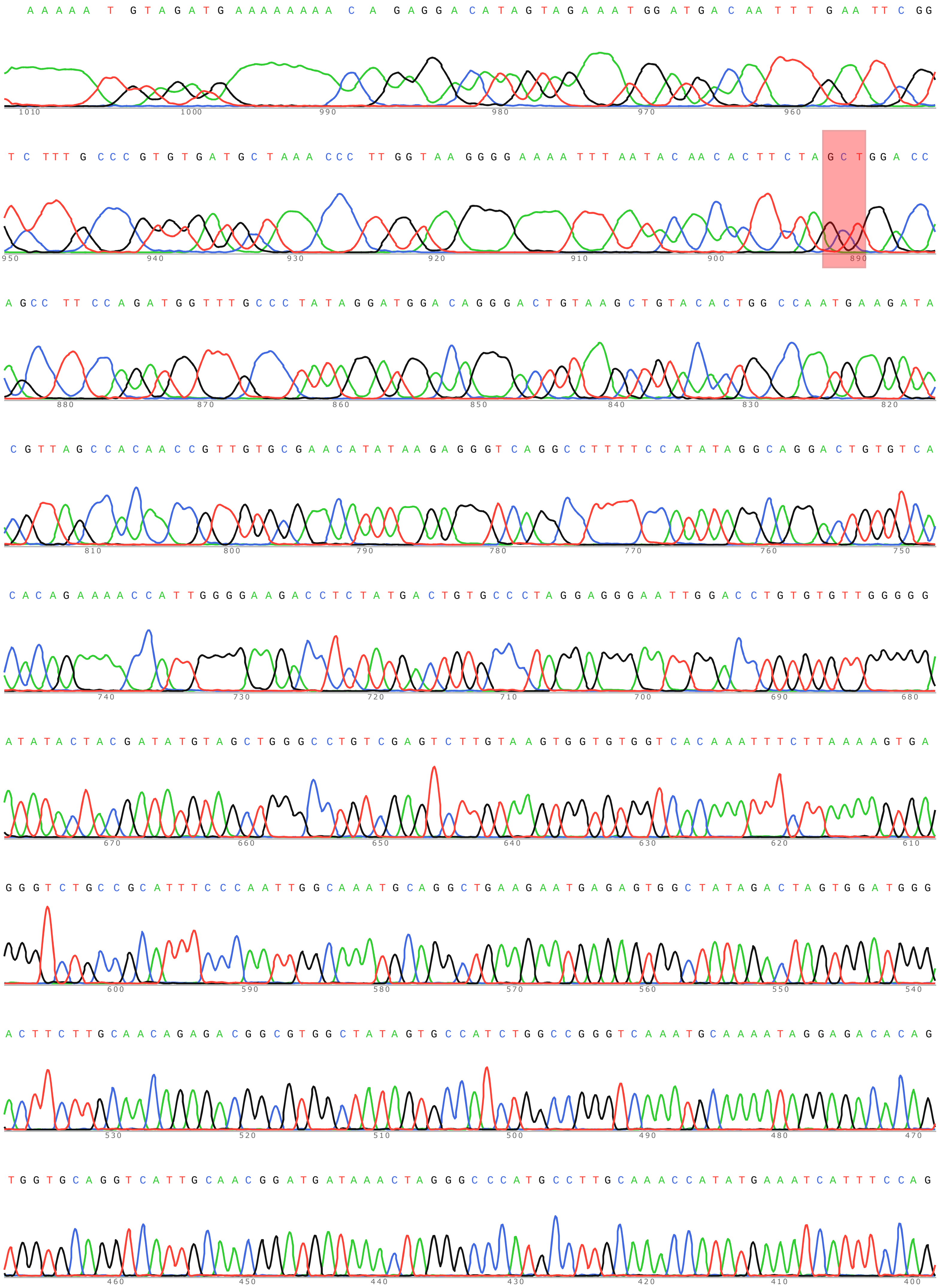
AAAAA T GTAGATG AAAAAAAA C A GAGGACATAGTAGAAAT GG ATGAC AA TTT GAA TT C GG
T C TTT G CCC GTGTGATGC TAAA CCC TT GGTAA GGGG AAAA TTT AATAC AA CACTT CTAGCTGGA CC
AGCC TT CCAGATGGTTTGCCC TATAGGATGGA CAGGGACTGTAAGCTGTACACTGG CCAATGAAGATA
CGTTAGCCACAACCGTTGTGCGAACATATAAGAGGGTCAGGCCTTTTCCATATAGGCAGGACTGTGTCA
CACAGAAAACCATTGGGGAAGACCTCTATGACTGTGCCCTAGGAGGGAATTGGACCTGTGTGTTGGGGG
ATATACTACGATATGTAGCTGGGCCTGTCGAGTCTTGTAAGTGGTGTGGTCACAAATTTCTTAAAAGTGA
GGGTCTGCCGCATTTCCCAATTGGCAAATGCAGGCTGAAGAATGAGAGTGGCTATAGACTAGTGGATGGG
ACTTCTTGCAACAGAGACGGCGTGGCTATAGTGCCATCTGGCCGGGTCAAATGCAAAATAGGAGACACAG
TGGTGCAGGTCATTGCAACGGATGATAAACTAGGGCCCATGCCTTGCAAACCATATGAAATCATTTCCAG

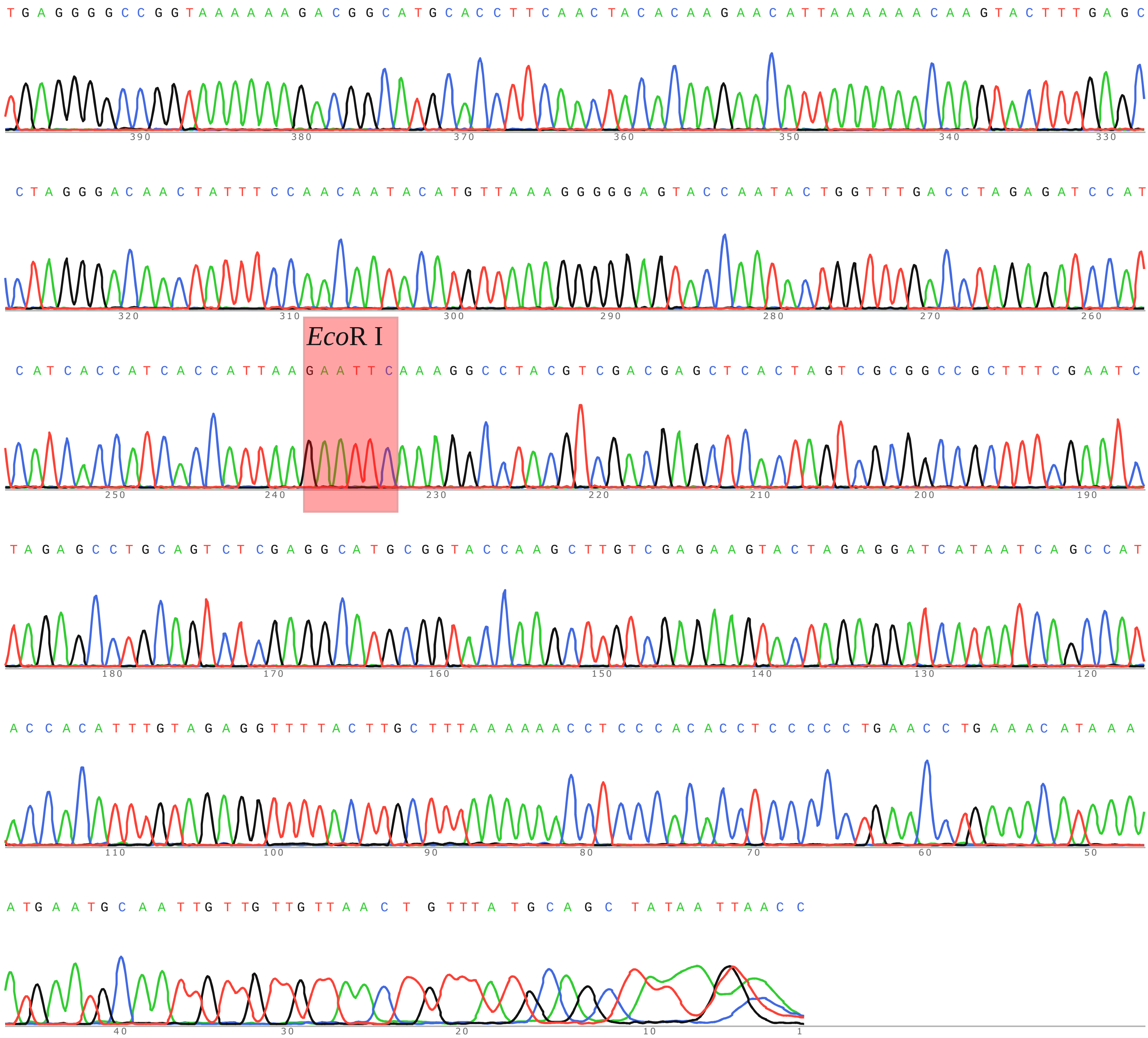
T G A G G G G C C G G T A A A A A A G A C G G C A T G C A C C T T C A A C T A C A C A A G A A C A T T A A A A A A C A A G T A C T T T G A G C
390 380 370 360 350 340 330
C T A G G G A C A A C T A T T T C C A A C A A T A C A T G T T A A A G G G G G A G T A C C A A T A C T G G T T T G A C C T A G A G A T C C A T
320 310 300 290 280 270 260
EcoR I
C A T C A C C A T C A C C A T T A A G A A T T C A A A G G C C T A C G T C G A C G A G C T C A C T A G T C G C G G C C G C T T T C G A A T C
250 240 230 220 210 200 190
T A G A G C C T G C A G T C T C G A G G C A T G C G G T A C C A A G C T T G T C G A G A A G T A C T A G A G G A T C A T A A T C A G C C A T
180 170 160 150 140 130 120
A C C A C A T T T G T A G A G G T T T T A C T T G C T T T A A A A A C C T C C C A C A C C T C C C C C T G A A C C T G A A A C A T A A A
110 100 90 80 70 60 50
A T G A A T G C A A T T G T T G T T G T T A A C T G T T T A T G C A G C T A T A A T T A A C C
40 30 20 10 1

BEP-496-pFsat Bac1-BVDV-XH32-E2 (G123L)_poly-F_2023-07-28_C06.ab1 (1010 bases)

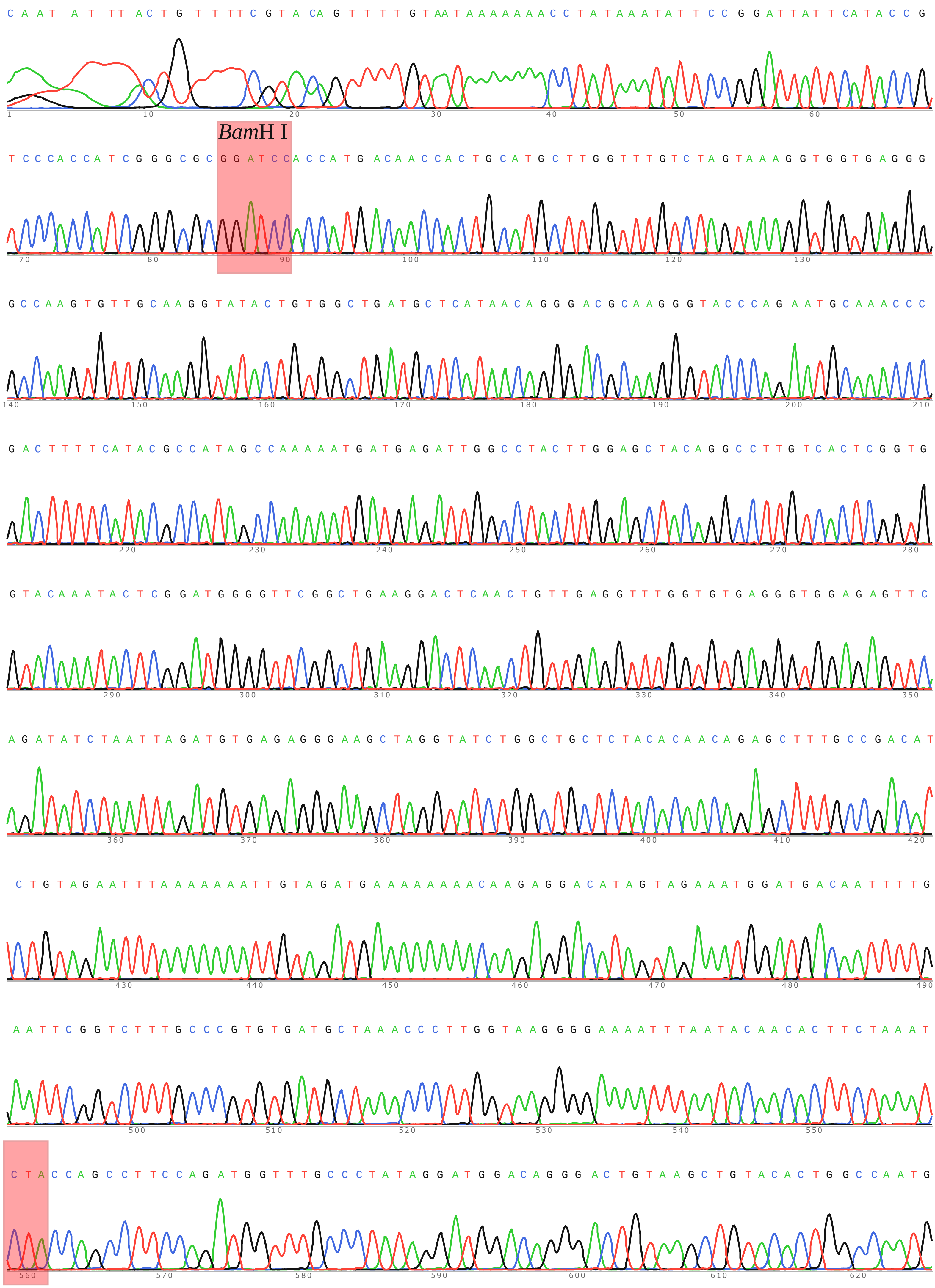

A A G A T A C G T T A G C C A C A A C C G T T G T G C G A A C A T A T A A G A G G G T C A G G C C T T T T C C A T A T A G G C A G G

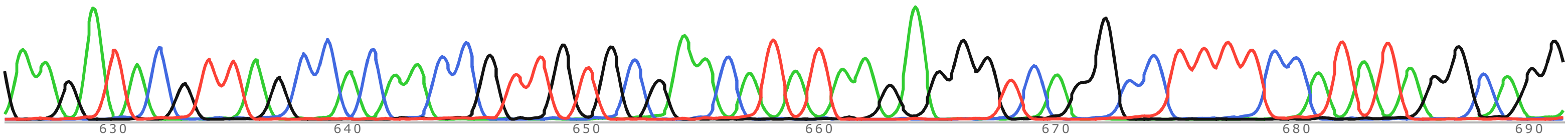

A C T G T G T C A C A C A G A A A A C C A T T G G G G A A G A C C T C T A T G A C T G T G C C C T A G G A G G G A A T T G G A C

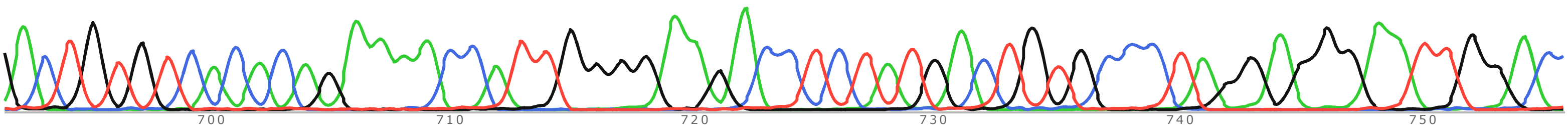

C T G T G T G T T G G G G G A T A T A C T A C G A T A T G T A G C T G G G C C T G T C G A G T C T T G T A A G T G G T G T G G

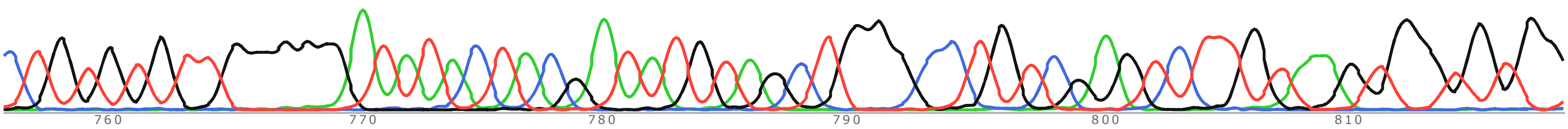

T C A C A A A T T T C T T A A A A G T G A G G G T C T G C C G C A T T T C C C A A T T G G C A A A T G C A GG C T G AA

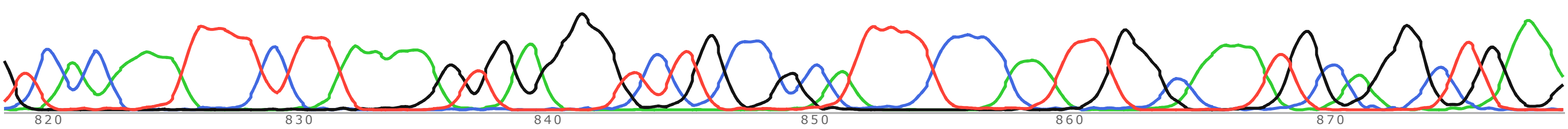

G AA T G A G A G T GG C T A T A G A C T A G TGG AT G G G AC TT C TT G C AA C A G A G A C GG C G TGG C

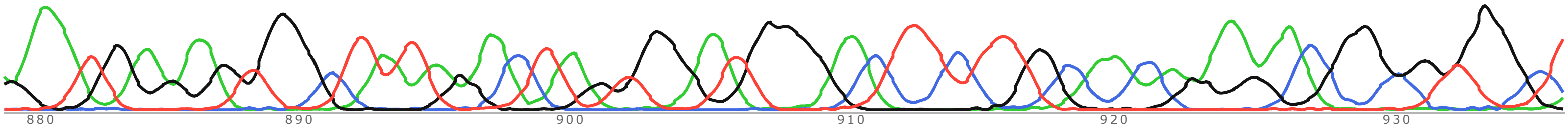

T A T A G T G CC A T C TGGG CC G G G T C A A A T G C AAAAA T A GG A G AC AC A G TGG C T G C A GG T

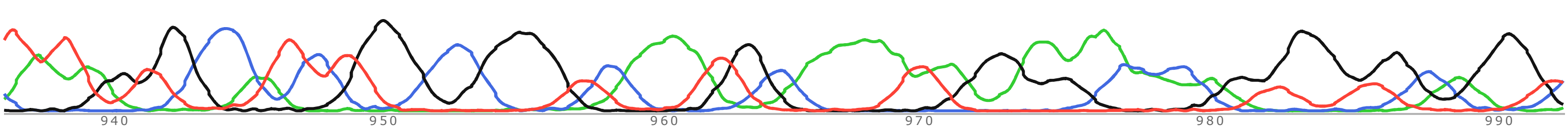

C A TTT G C A C GGG A T G A T A

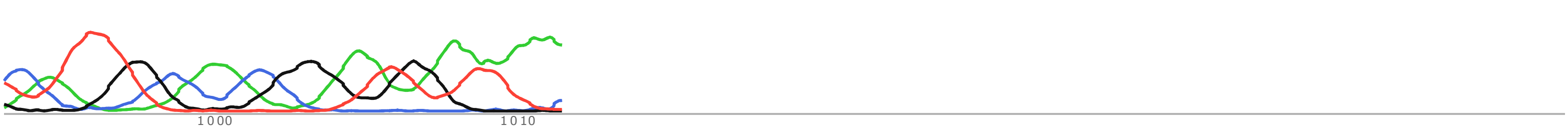

BEP-496-pFsat Bac1-BVDV-XH32-E2 (G123L)_SV40pA-R_2023-07-28_C07.ab1 (1010 bases)

AGATGAAAAAAAACAGAGGACATAGTAGAAATGGATGACAATTTTGAATTCGGTCTTTGCCC

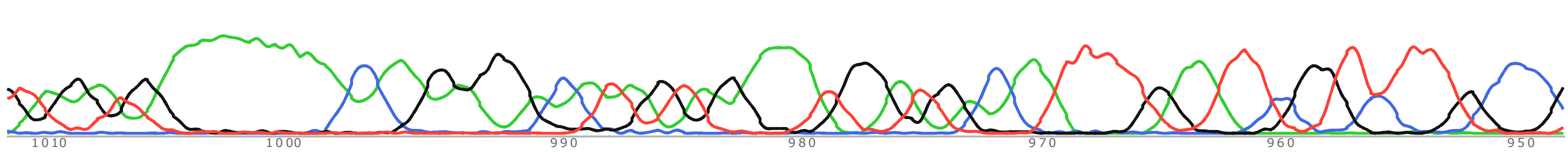

GTGTGATGCTAAACCCTTGGTAAGGGGAAAATTTAATACAACACTTCTAAATCTACCAGCCTTTCCAG

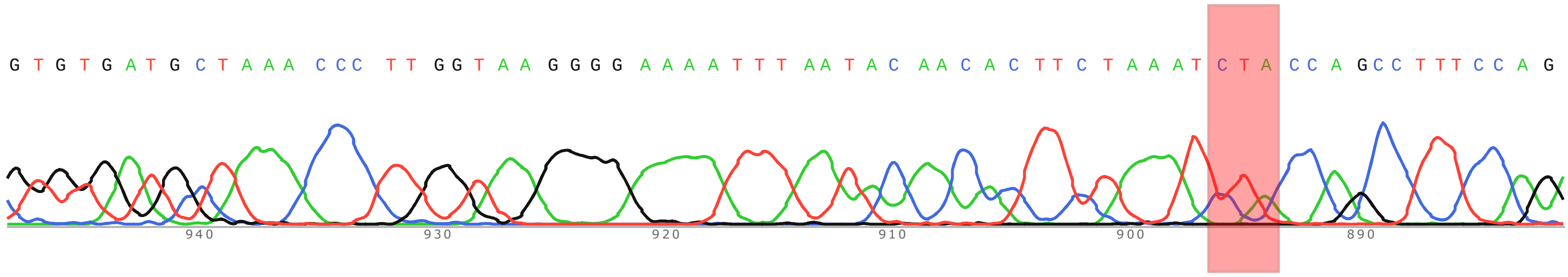

ATGGTTTGCCCTATAGGATGGACAGGGACTGTAAGCTGTACACTGGCCAATGAAGATACGTTAGCCAC

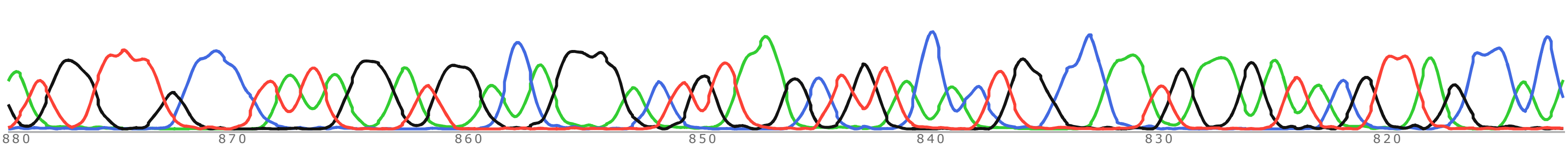

AACCGTTGTGCGAACATATAAGAGGGTCAGGCCTTTTCCATATAGGCAGGACTGTGTCACACAGAAAAC

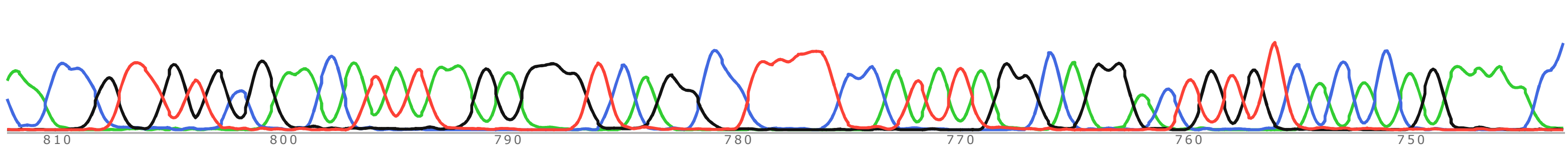

CATTGGGGAAGACCTCTATGACTGTGCCCTAGGAGGGAATTGGACCTGTGTGTTGGGGGATATACTACGA

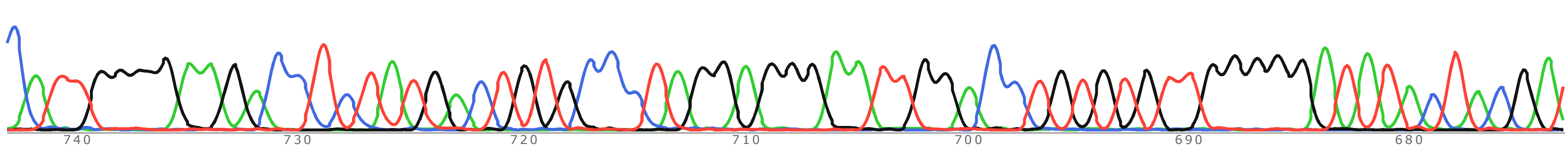

TATGTAGCTGGGCCTGTCGAGTCTTGTAAGTGGTGTGGTCACAAATTTCTTAAAAGTGAGGGTCTGCCGC

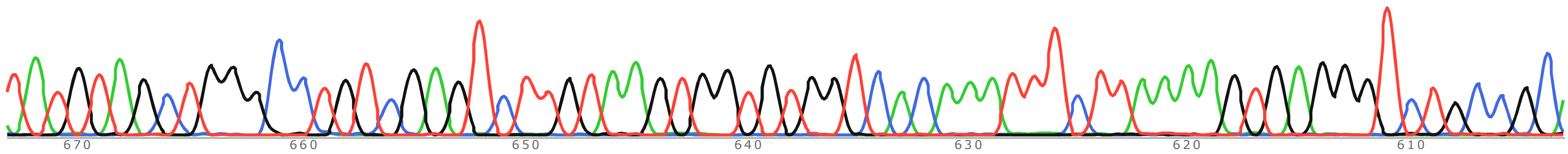

ATTTCCCAATTGGCAAATGCAGGCTGAAGAATGAGAGTGGCTATAGACTAGTGGATGGGACTTCTTGCAA

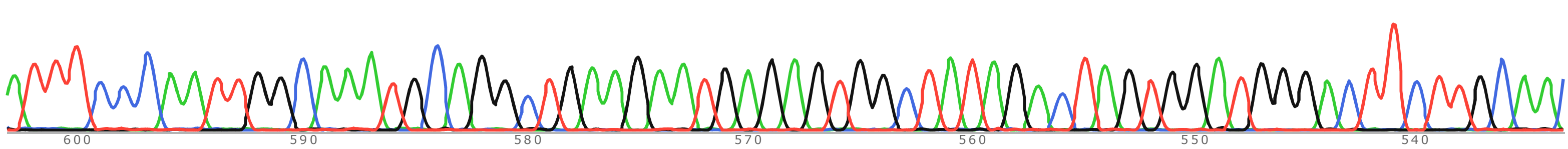

CAGAGACGGCGTGGCTATAGTGCCATCTGGCCGGGTCAAATGCAAAATAGGAGACACAGTGGTGCAGGTC

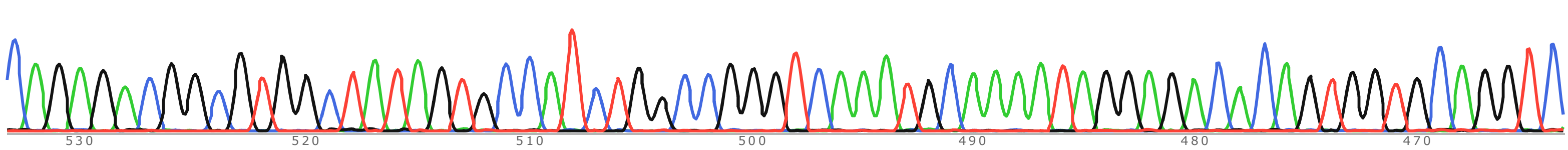

ATTGCAACGGATGATAAACTAGGGCCCATGCCTTGCAAACCATATGAAATCATTTCCAGTGAGGGGCCGG

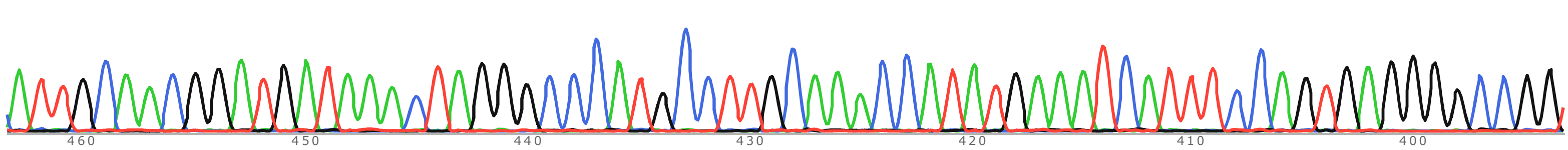

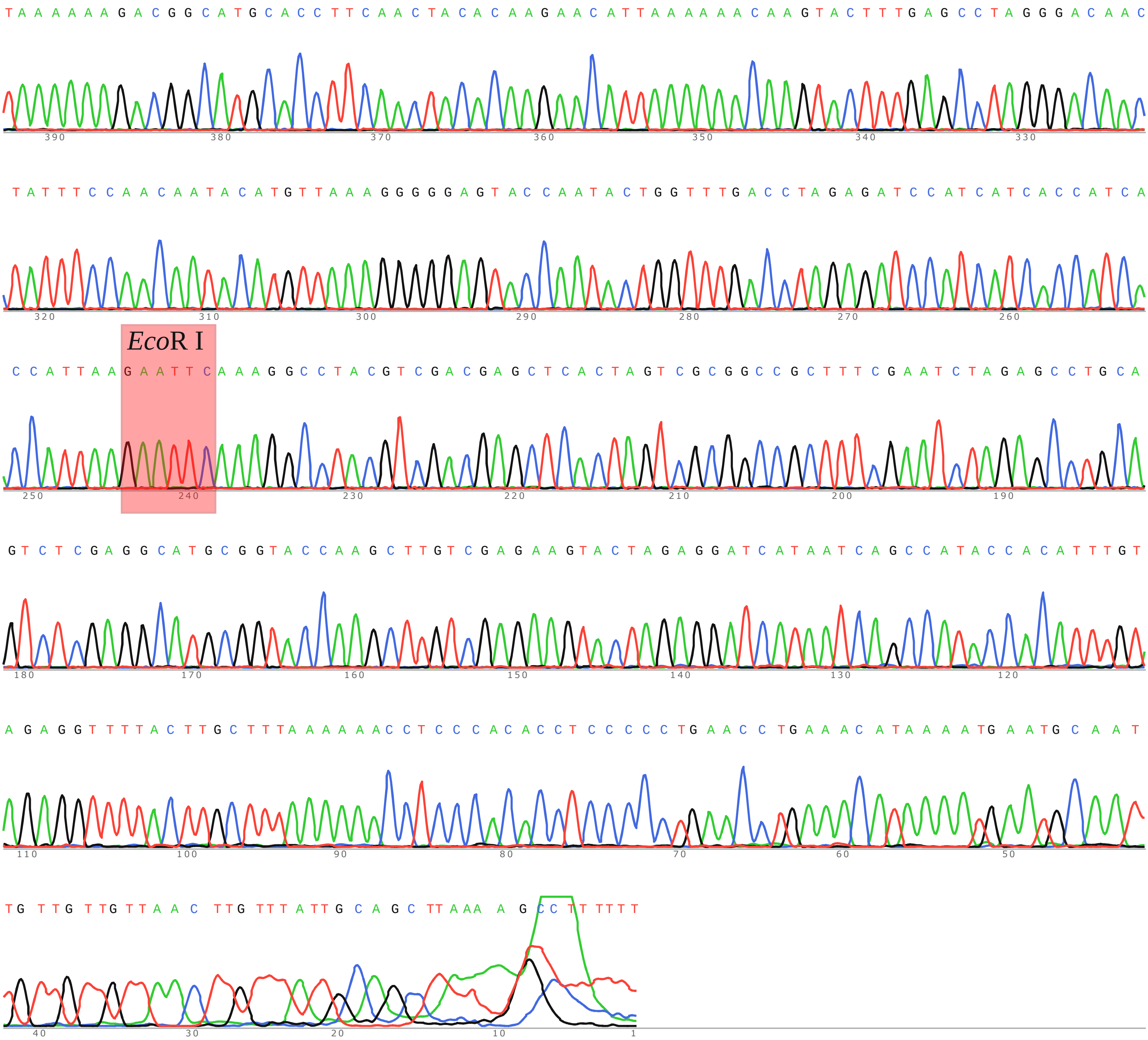
T A A A A A A G A C G G C A T G C A C C T T C A A C T A C A C A A G A A C A T T A A A A A A C A A G T A C T T T G A G C C T A G G G A C A A C
390 380 370 360 350 340 330
T A T T T C C A A C A A T A C A T G T T A A A G G G G G A G T A C C A A T A C T G G T T T G A C C T A G A G A T C C A T C A T C A C C A T C A
320 310 300 290 280 270 260
EcoR I
C C A T T A A G A A T T C A A A G G C C T A C G T C G A C G A G C T C A C T A G T C G C G G C C G C T T T C G A A T C T A G A G C C T G C A
250 240 230 220 210 200 190
G T C T C G A G G C A T G C G G T A C C A A G C T T G T C G A G A A G T A C T A G A G G A T C A T A A T C A G C C A T A C C A C A T T T G T
180 170 160 150 140 130 120
A G A G G T T T T A C T T G C T T T A A A A A C C T C C C A C A C C T C C C C C T G A A C C T G A A A C A T A A A A T G A A T G C A A T
110 100 90 80 70 60 50
T G T T G T T G T T A A C T T G T T T A T T G C A G C T T A A A A G C C T T T T T
40 30 20 10 1

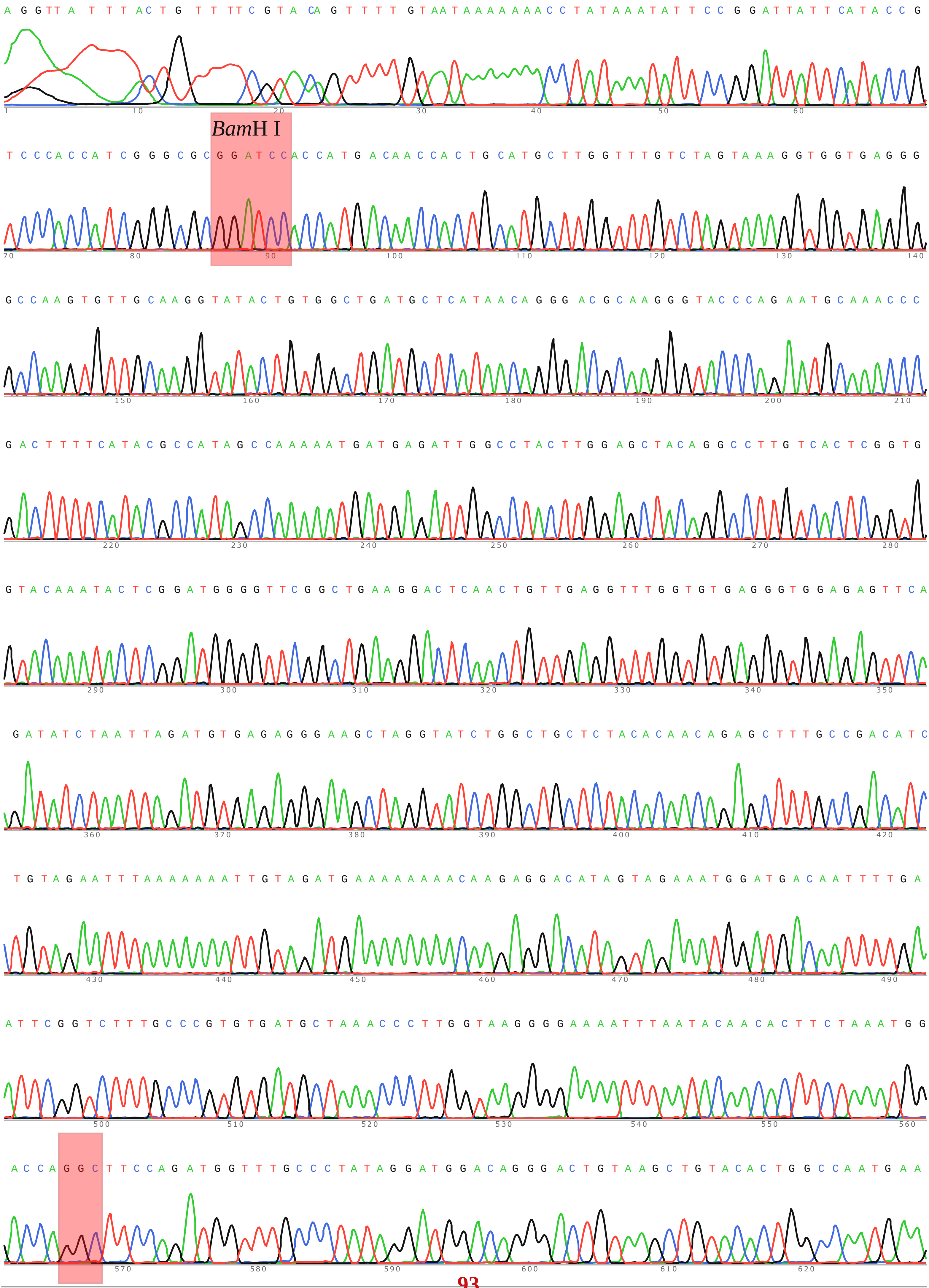
AGGTTATTTACTGTTTTCGTACAGTTTTGTAATAAAAAAACCTATAAATATTCCGGATTATTCATACCG
BamH I
TCCCACCATCGGGCGCGGATCCACCATGACAACCACTGCATGCTTGGTTTGTCTAGTAAAGGTGGTGAGGG
GCCAAGTGTTGCAAGGTATACTGTGGCTGATGCTCATAACAGGGACGCAAGGGTACCCAGAATGCAAACCC
GACTTTTCATACGCCATAGCCAAAAATGATGAGATTGGCCTACTTGGAGCTACAGGCCTTGTCACTCGGTG
GTACAAATACTCGGATGGGGTTCGGCTGAAGGACTCAACTGTTGAGGTTTGGTGTGAGGGTGGAGAGTTCA
GATATCTAATTAGATGTGAGAGGGAAGCTAGGTATCTGGCTGCTCTACACAACAGAGCTTTGCCGACATC
TGTAGAATTTAAAAAAATTGTAGATGAAAAAAAACAAGAGGACATAGTAGAAATGGATGACAATTTTGA
ATTCGGTCTTTGCCCGTGTGATGCTAAACCCTTGGTAAGGGGAAAATTTAATACAACACTTCTAAATGG
ACCAGGCTTCCAGATGGTTTGCCCTATAGGATGGACAGGGACTGTAAGCTGTACACTGGCCAATGAA

G A T A C G T T A G C C A C A A C C G T T G T G C G A A C A T A T A A G A G G G T C A G G C C T T T T C C A T A T A G G C A G G A C T

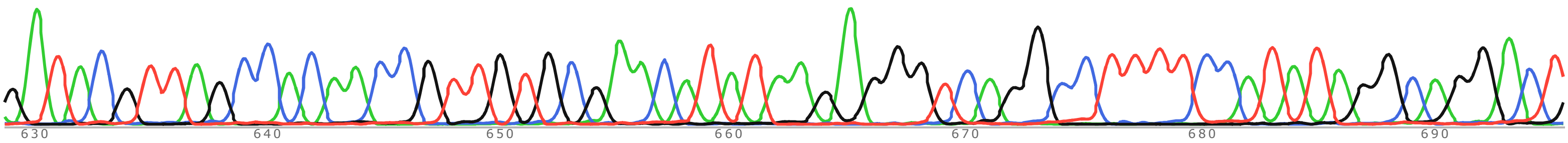

G T G T C A C A C A G A A A A C C A T T G G G G A A G A C C T C T A T G A C T G T G C C C T A G G A G G G A A T T G G A C C T G T

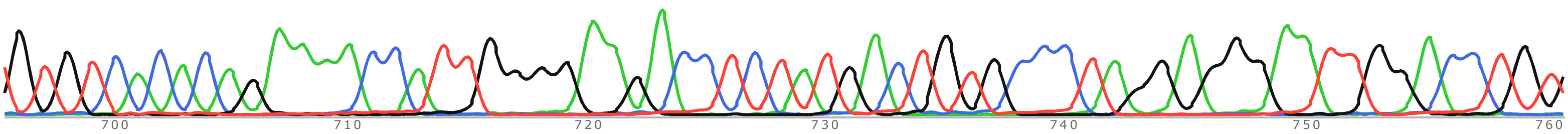

G T G T T G G G G G A T A T A C T A C G A T A T G T A G C T G G G C C T G T C G A G T C T T G T A A G T G G T G T G G T C A C

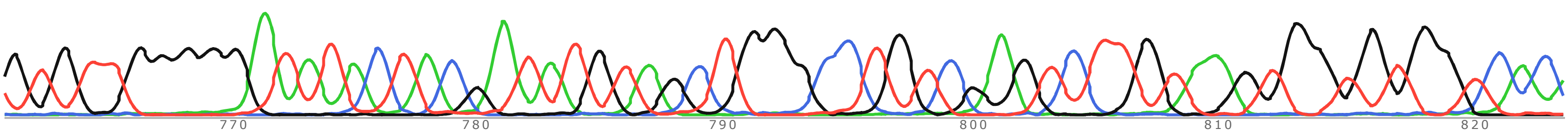

A A A T T T C T T A A A A G T G A G G G T C T G C C G C A T T T C C C A A T T G G C A A A T G C A G G C T G A A G A A T G A

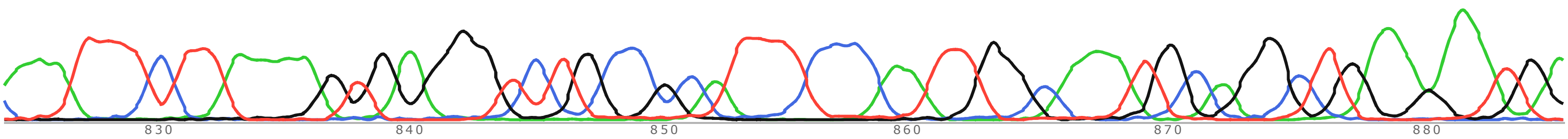

G A G T G G C T A T A G A C T A G T G G A T G G G A C T T C T T G C A A C A G A G A C G G C G T G G C T A T A G T G

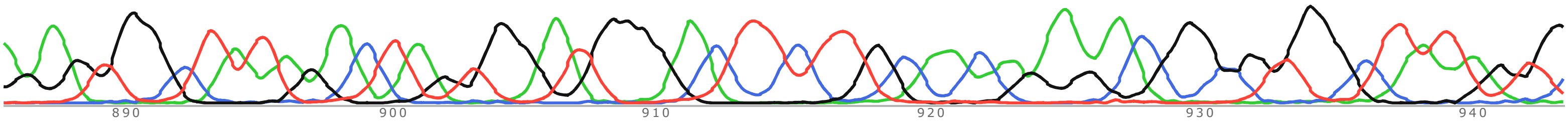

C C A T C T G G G C C G G G T C A A A T G C A A A A T A G G A G A C A C A G T G G C T G C A G G T C A T T T G C A

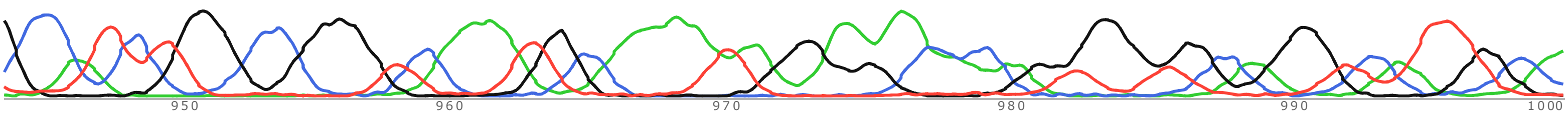

A C G G A T G A T A

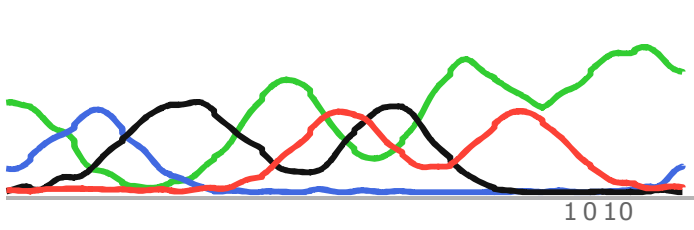

AAAAAACAAGAGGACATAGTAGAAATGGATGACAATTTTGAATTCGGTCTTTGCCCGTGTG

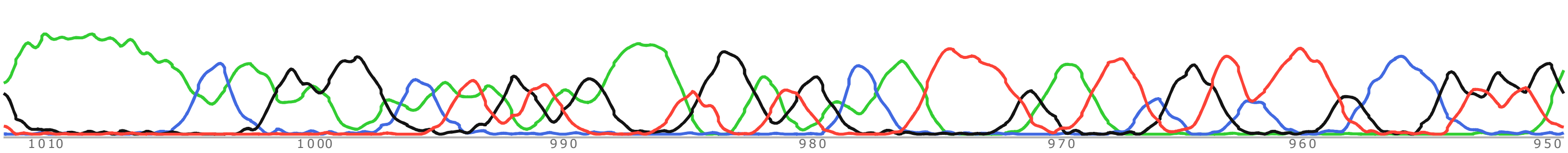

ATGCTAAACCCTTGGTAAGGGGAAAATTTAATACAACACTTCTAAATGGACCAGGCTTCCAGATGG

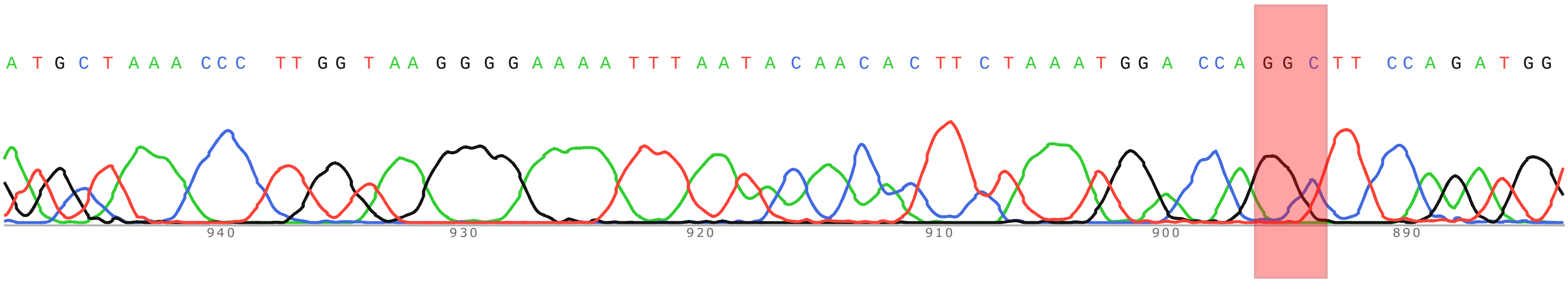

TTTGCCCTATAGGATGGACAGGGACTGTAAGCTGTACACTGGCCAATGAAGATACGTTAGCCACAAC

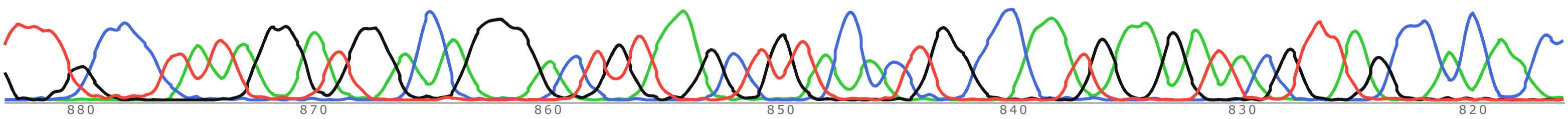

CGTTGTGCGAACATATAAGAGGGTCAGGCCTTTTCCATATAGGCAGGACTGTGTCACACAGAAAACCAT

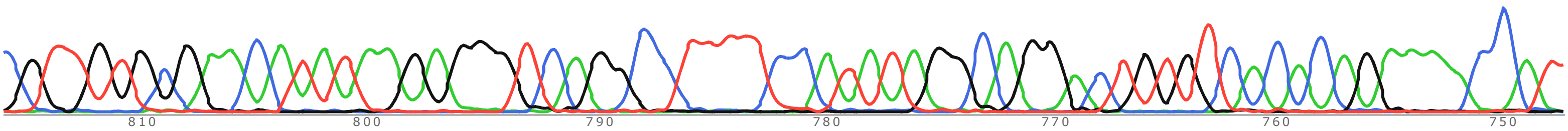

TGGGGAAGACCTCTATGACTGTGCCCTAGGAGGGAATTGGACCTGTGTGTTGGGGGATATACTACGATAT

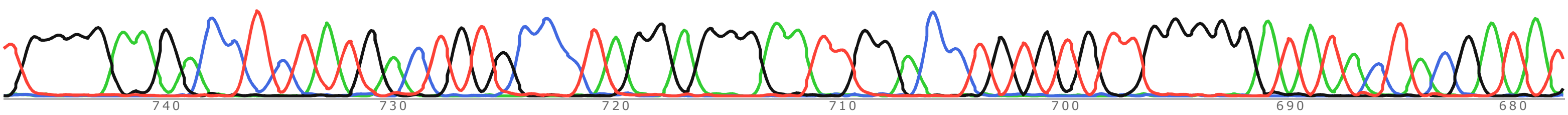

GTAGCTGGGCCTGTCGAGTCTTGTAAGTGGTGTGGTCACAAATTTCTTAAAAGTGAGGGTCTGCCGCAT

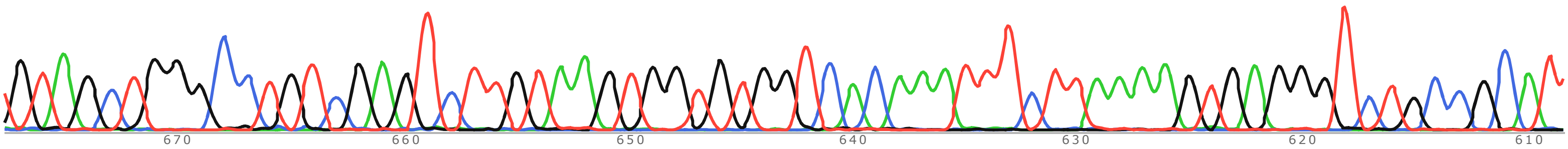

TTCCCAATTGGCAAATGCAGGCTGAAGAATGAGAGTGGCTATAGACTAGTGGATGGGACTTCTTGCAACA

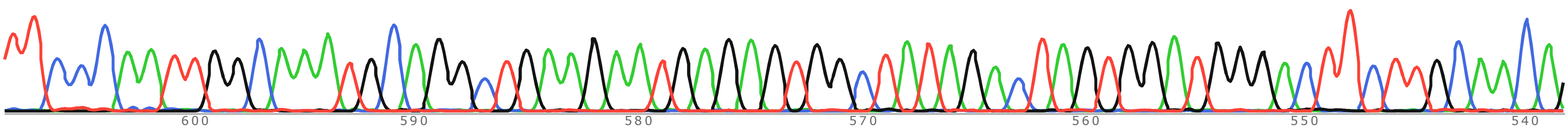

GAGACGGCGTGGCTATAGTGCCATCTGGCCGGGTCAAATGCAAAATAGGAGACACAGTGGTGCAGGTCAT

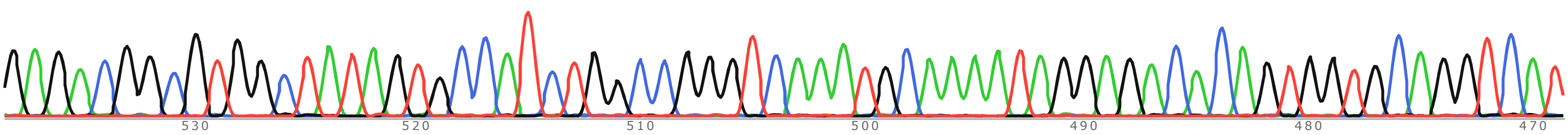

TGCAACGGATGATAAACTAGGGCCCATGCCTTGCAAACCATATGAAATCATTTCCAGTGAGGGGCCGGTA

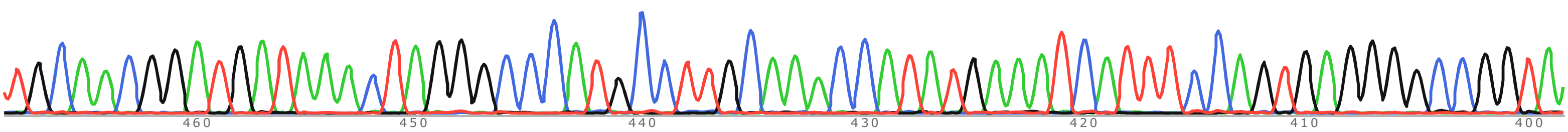

AAAAAGACGGCATGCACCTTCAACTACACAAGAACATTAAAAAACAAGTACTTTGAGCCTAGGGACAACTA

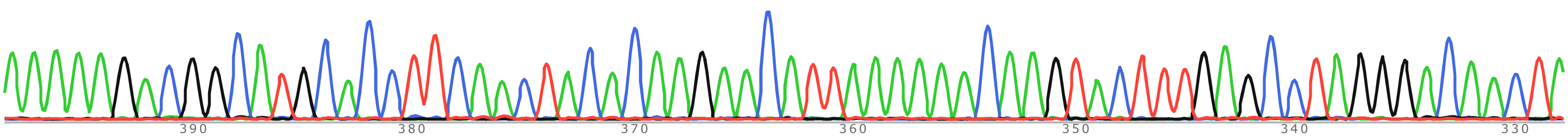

TTTCCAACAATACATGTTAAAGGGGGAGTACCAATACTGGTTTGACCTAGAGATCCATCATCACCATCAC

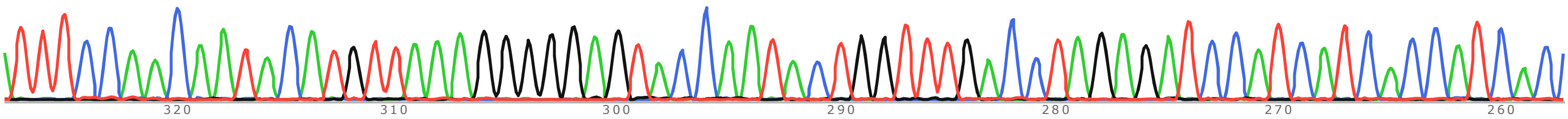

CATTAAGAATTCAAAGGCCTACGTCGACGAGCTCACTAGTCGCGGCCGCTTTCGAATCTAGAGCCTGCAGT

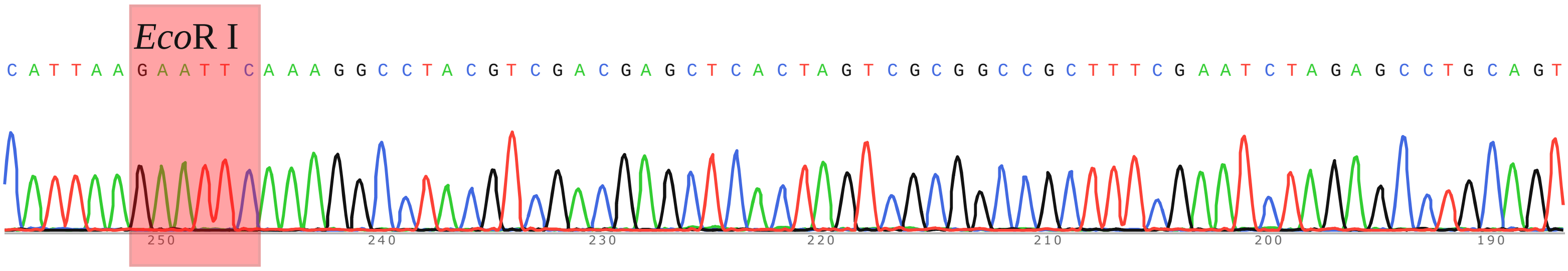

CTCGAGGCATGCGGTACCAAGCTTGTCGAGAAGTACTAGAGGATCATAATCAGCCATACCACATTTGTAG

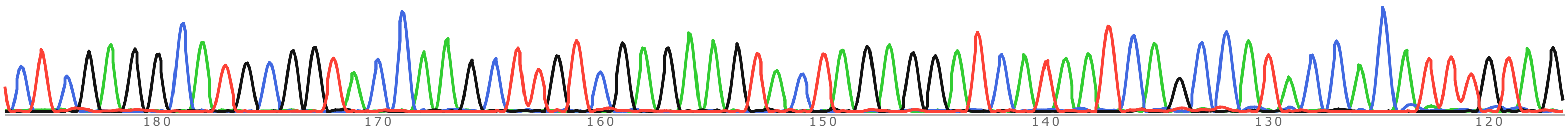

AGGTTTTACTTGCTTTAAAAAACCTCCCACACCTCCCCCTGAACCTGAAACATAAAATGAATGCAATTGTT

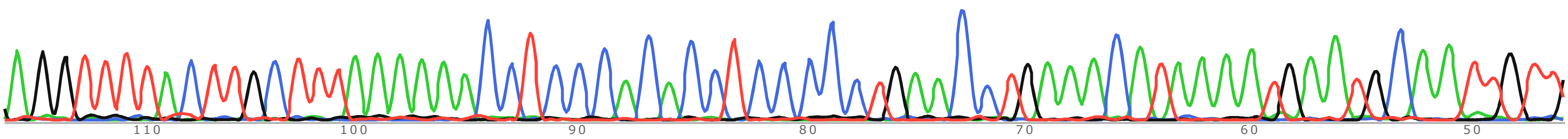

GTTGTAACTGTTATGCAGCTATAGCCTGAGGGCTCGGAAAGGGGC

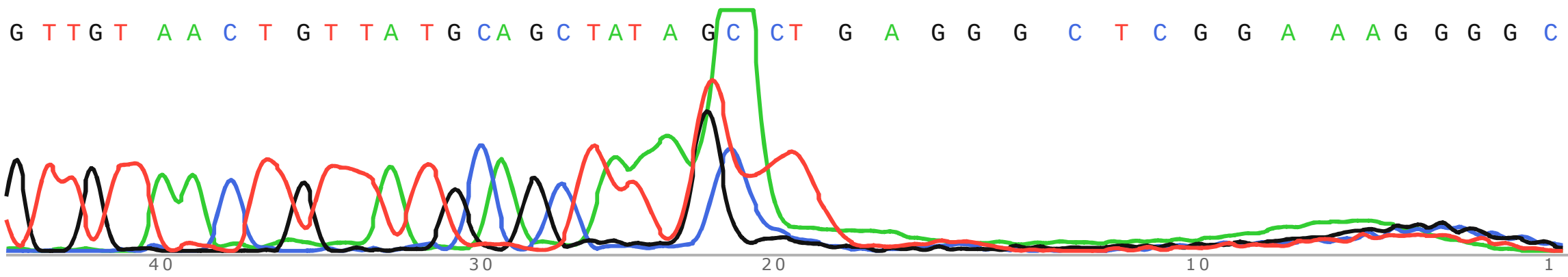

BEP-499-pFsat Bac1-CSFV-HCLV-E2 (S124P)_poly-F_2023-07-28_D08.ab1 (1010 bases)

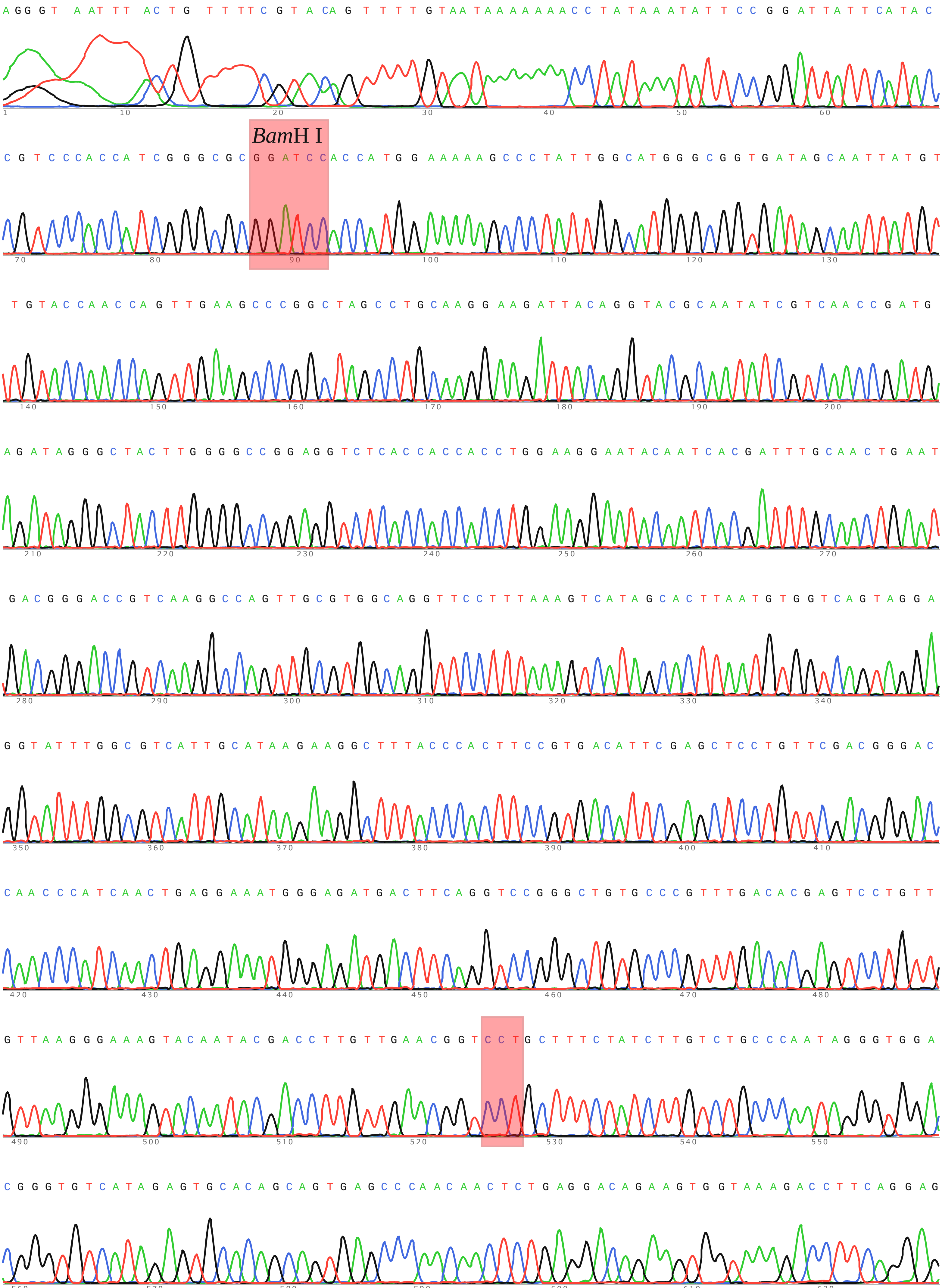

AGACAAGCCCTTTCCGCACAGAATGGATTGTGTGACCACCACAGTGGAAAATGAAGATTTATTCTATTGT

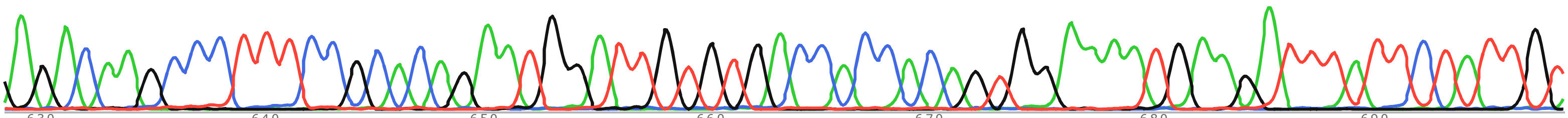

AAGTTGGGGGGCAACTGGACATGTGTGAAAGGCGAGCCAGTGGTCTACACAGGGGGGGGTAGTAAAACAA

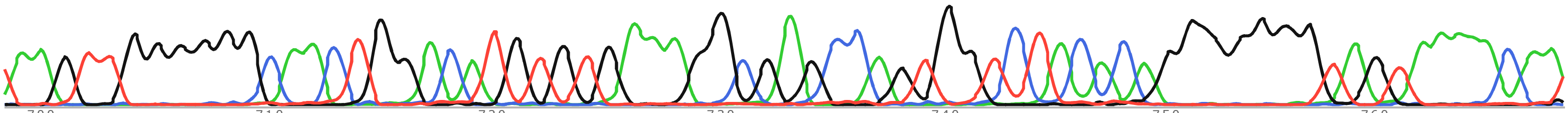

TGTAGATGGTGTGGCTTCGACTTCGATGGGCCTGACGGACTCCCGCATTACCCCATAGGTAAGTGCAT

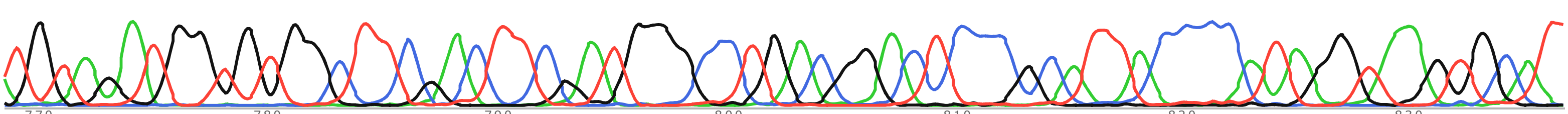

TTTGGCAAATGAGACAGGTTACAGAATAGTAGATTCAACGG ACTGTAACAGAGAT GGCGTTGTAATCA

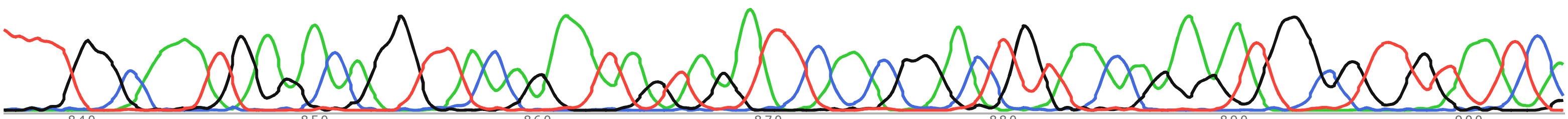

GCACAGA GGGG AGTCATGAGTGC TTGATC GGTAACACGACTGTCAA GGTGCATGCATCAGATGAAA

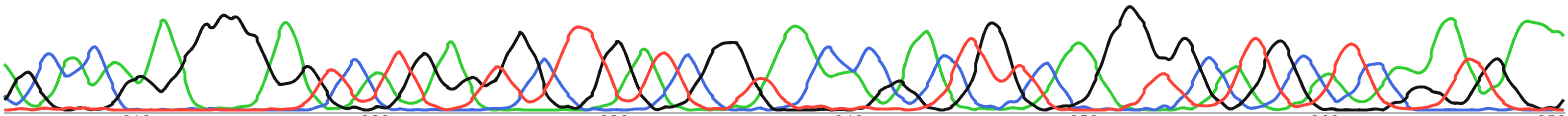

GACTGGG CCC TATG CCATGCAGACCT AAAGAGATTGTCTC

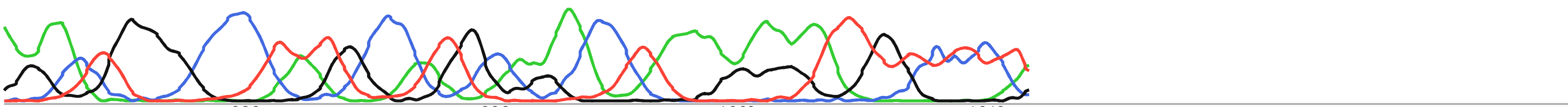

98

TGTTCGACGGGACCAACCCATCAACTGAGGAAATGGGAGATGACTTCAGGTCCGGGCTGTGCC

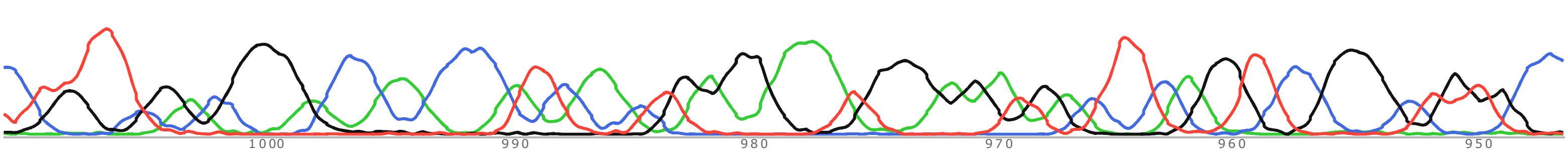

CGTTTGACACGAGTCCTGTTGTTAAGGGAAAGTACAATACGACCTTTGTTGAACGGTCCTGCTTTCT

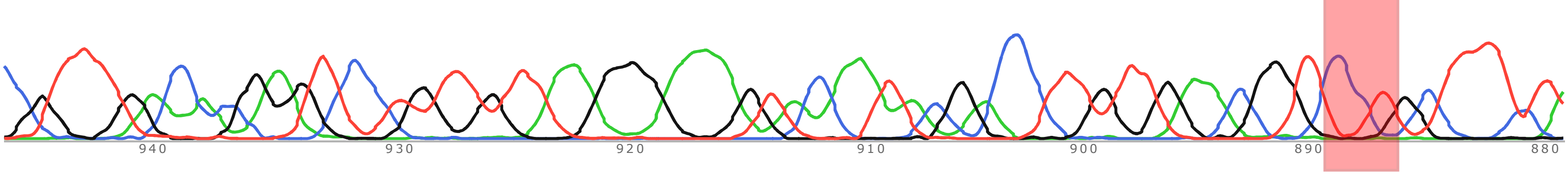

ATCTTGTCTGCCCAATAGGGTGGACGGGTGTCATAGAGTGCACAGCAGTGAGCCCAACAACTCTGAGG

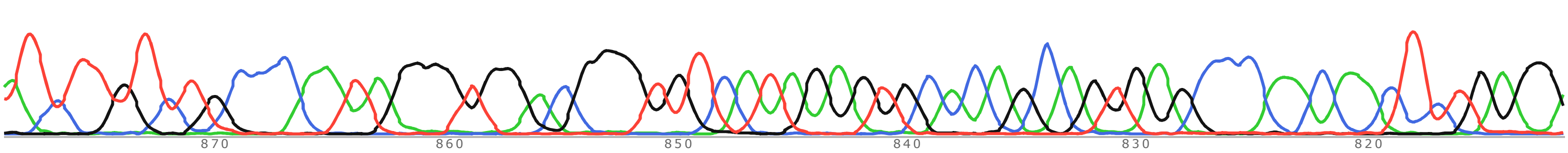

ACAGAAGTGGTAAAGACCttCAGGAGAGACAAGCCCTTTCCGCACAGAATGGATTGTGTGACCACCACA

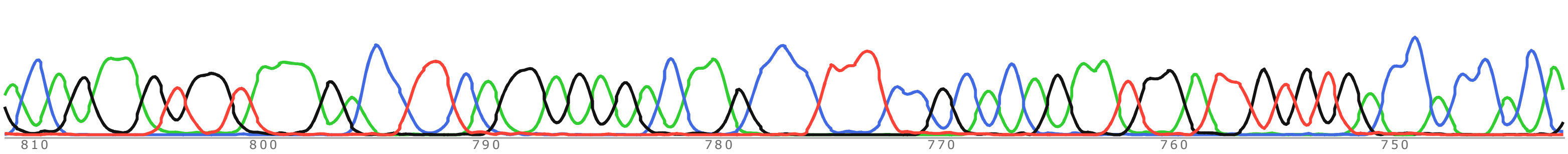

GTGGAAAATGAAGATTTATTCTATTGTAAGTTGGGGGGCAACTGGACATGTGTGAAAGGCGAGCCAGTG

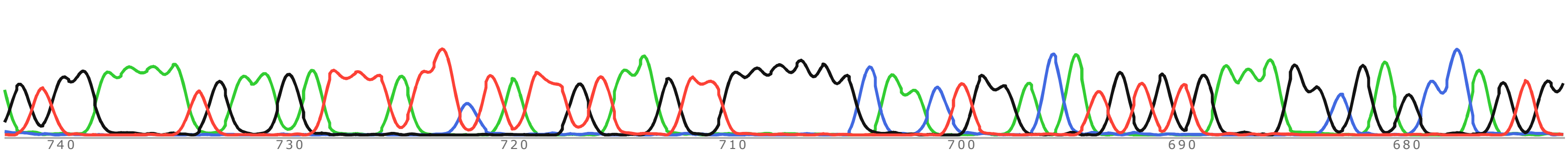

GTCTACACAGGGGGGGTAGTAAAACAATGTAGATGGTGTGGCTTCGACTTCGATGGGCCTGACGGACTCC

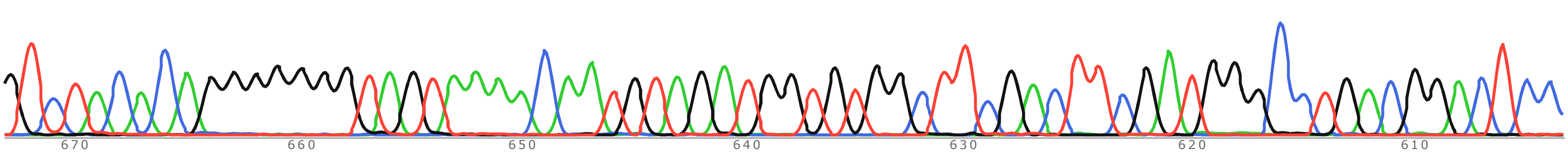

CGCATTACCCCATAGGTAAGTGCATTTTGGCAAATGAGACAGGTTACAGAATAGTAGATTCAACGGACTG

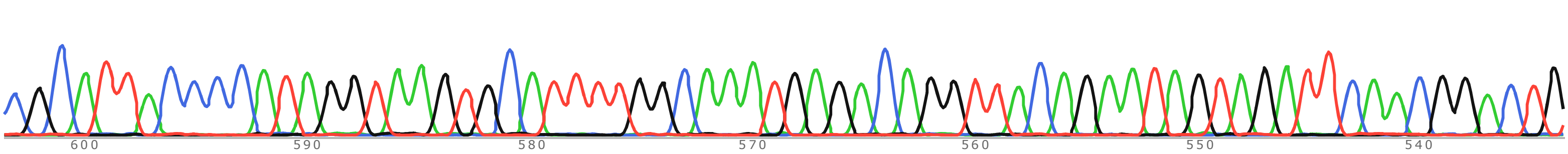

TAACAGAGATGGCGTTGTAATCAGCACAGAGGGGAGTCATGAGTGCTTGATCGGTAACACGACTGTCAAG

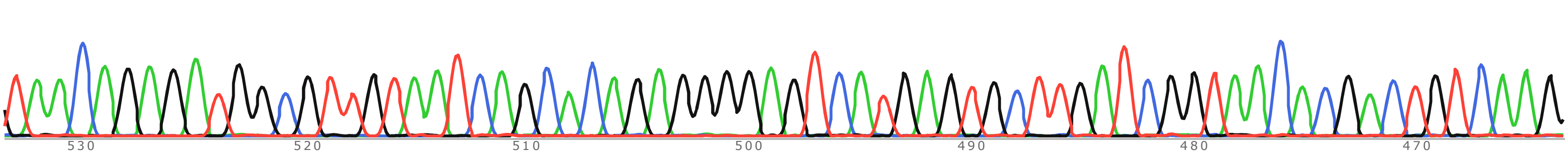

GTGCATGCATCAGATGAAAGACTGGGCCCCTATGCCATGCAGACCTAAAGAGATTGTCTCTAGTGCTGGTC

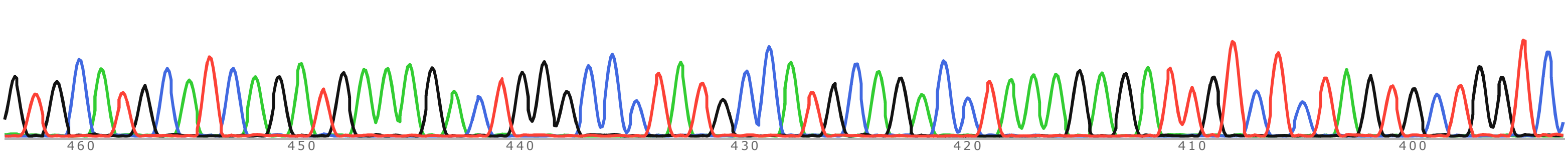

C T G T A A A G A A A A C C T C C T G T A C A T T C A A C T A C A C A A A A A C T T T G A A G A A C A G G T A C T A T G A G C C C A G G G A C

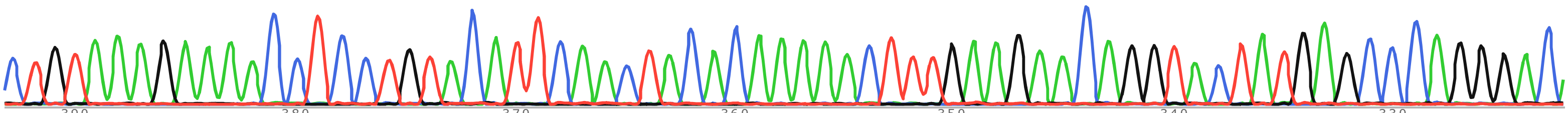

390 380 370 360 350 340 330

A G C T A C T T C C A G C A A T A T A T G C T T A A G G G T G A G T A T C A G T A C T G G T T T G A C C T G G A T G C G C A T C A T C A C C A

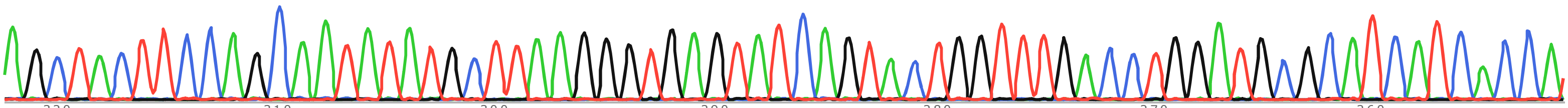

320 310 300 290 280 270 260

*Eco*R I

T C A C C A T T A A G A A T T C A A A G G C C T A C G T C G A C G A G C T C A C T A G T C G C G G C C G C T T T C G A A T C T A G A G C C T

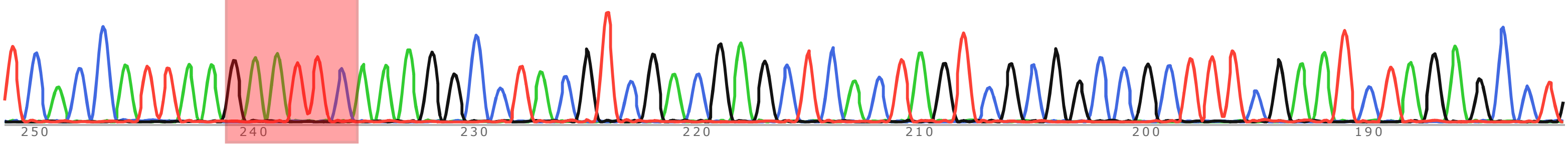

250 240 230 220 210 200 190

G C A G T C T C G A G G C A T G C G G T A C C A A G C T T G T C G A G A A G T A C T A G A G G A T C A T A A T C A G C C A T A C C A C A T T

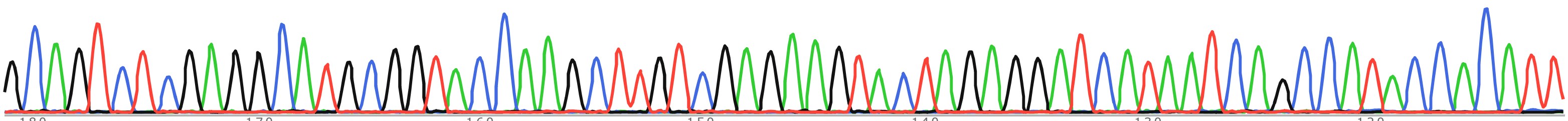

180 170 160 150 140 130 120

T G T A G A G G T T T T A C T T G C T T T A A A A A A C C T C C C A C A C C T C C C C C T G A A C C T G A A A C A T A A A A T G A A T G C

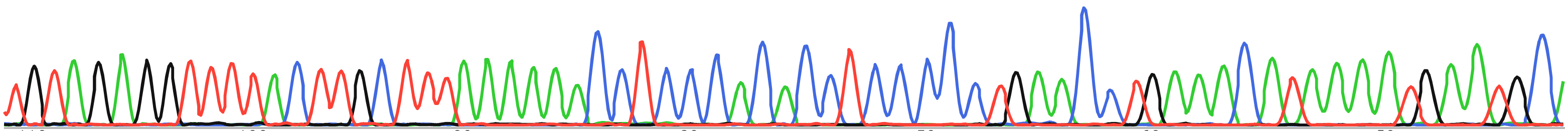

110 100 90 80 70 60 50

A A T T G T T G T T G T T A A C T G T T T A T G C A G C T A T A A G T C T G T T T T

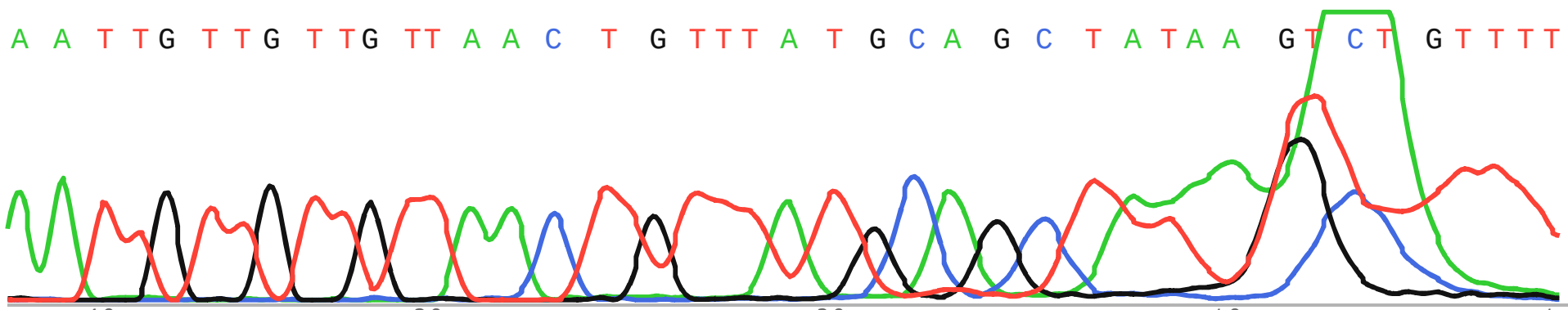

40 30 20 10 1

*Bam*H I

TTTTGTATAAAAAAACCTATAAATATTCCGGATTATTCATACCGTCCCACCATCGGGCGCGGATCCACCA

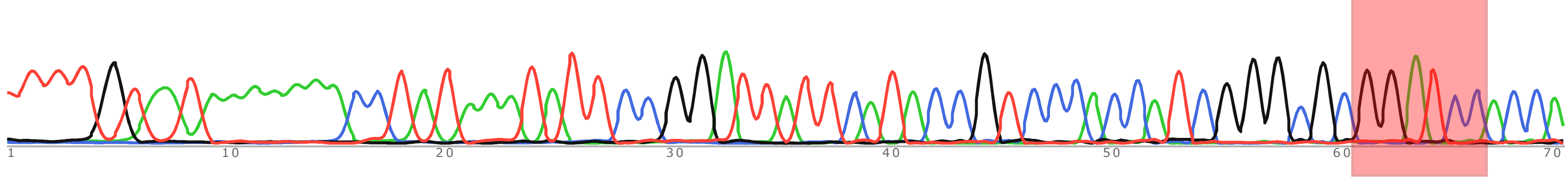

TGACCACCACTGCCTTCCTGGTGTGCCTGGTGAAGGTGGTGCGTGGTCAGGTCGTGCAGGGTGTCATCTG

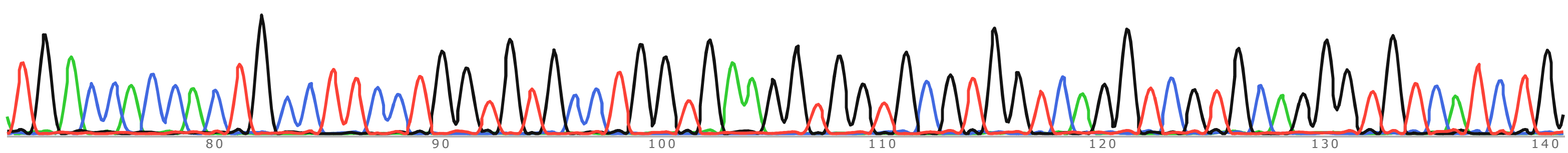

GCTGCTGCTGATCACCGGTGTCCAGGGCCACCTGGACTGCAAGCCTGACTTCTCCTACGCTATCGCTAAGG

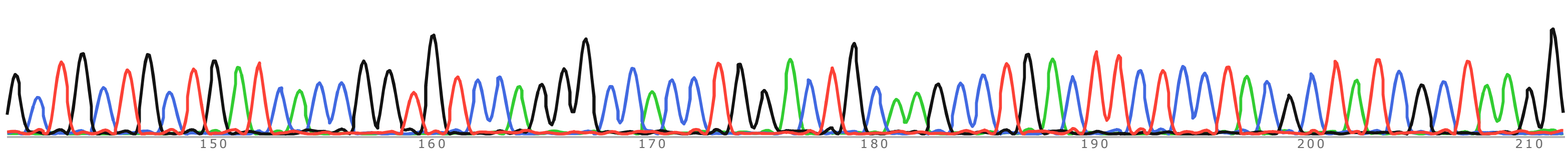

ACAAGCGTATCGGTCTGCTGGGTGCCGAGGGTCTGACCACTACTTGGTACGACTACAGCGACGGCATGCAG

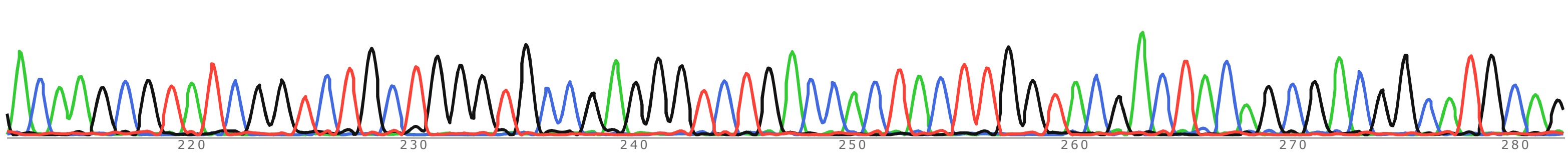

ATCTCCGACACCATGGTCGAGGTGCGTTGCAAGGGCGGTGAGTTCACTTTCGTGAAGAAGTGCAAGACCG

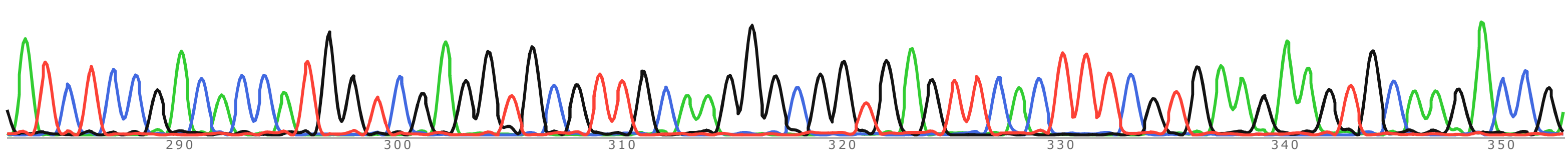

AAACCCGTTACCTGGCTACCCTGCACCAGCGTGCCCTGCCTACCAGCGTGGTCTTCGAAAAGATCTTCGA

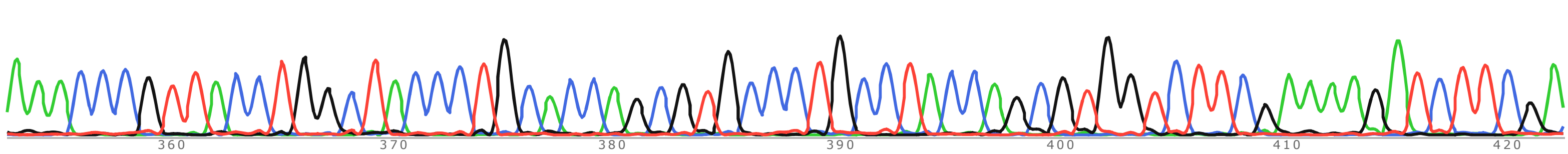

CGGCCAGAAGAGCGCCGACACTATCGAAATGGACGACAACTTCGAGTTCGCTCTCTGCCCCTGCGACGC

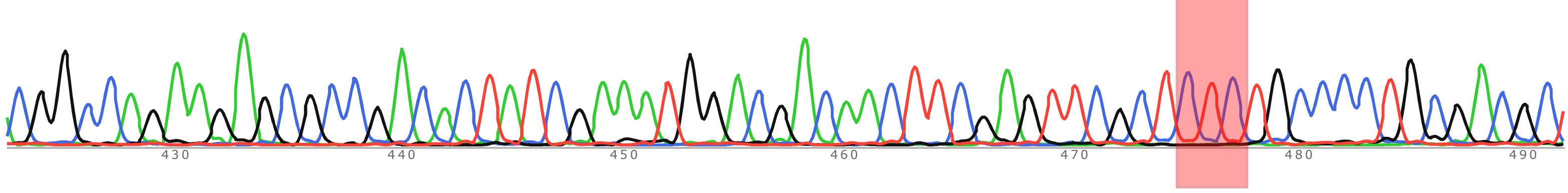

TAAGCCTGTGGTGCGCGGTAAATTCAACACCACTCTGCTGAACGGCCCCGCTTTCCAGATGGTCTGCCC

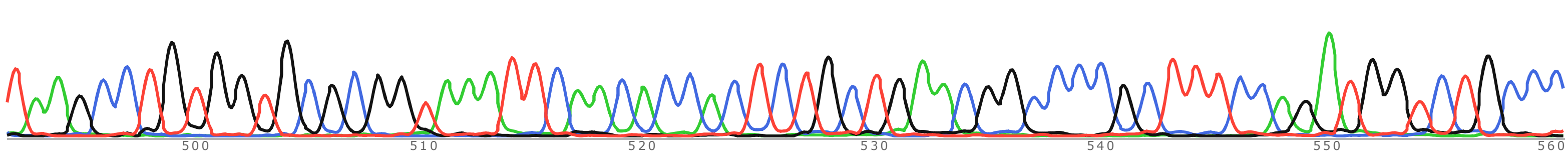

TATCGGTTGGACTGGCACCGTCAACTGCGTGCTGACTAACAAGGACACCCTGGACATGGTCGTGGTC

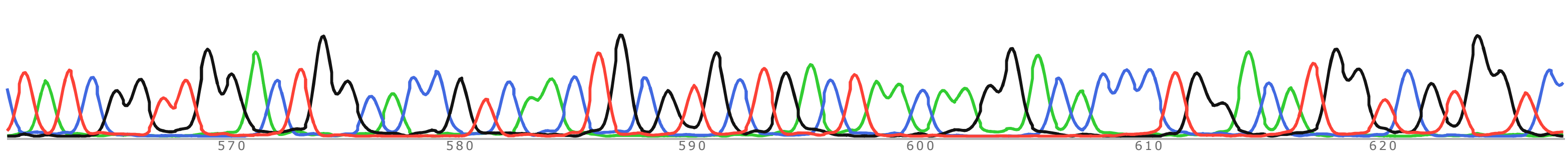

C A G A C T T A C A A G C G C G G C A G C C C T T T C C C C A A C C G T C A G G G C T G C G T C A C T C A G A A G C T G C T G G G C G

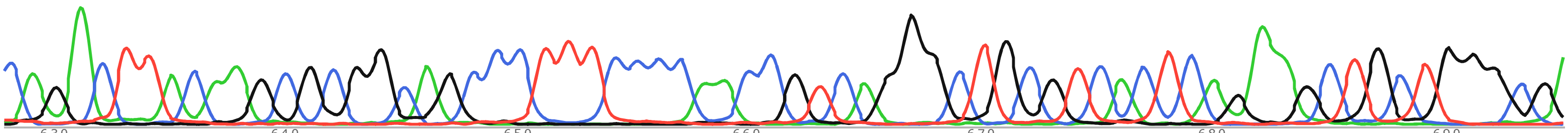

A A G A C C T G T A C G A C T G C A C T C T G G G C G G T A A C T G G A C C T G C A T C G C T G G T G A A C A G C T G C A C T A C A C

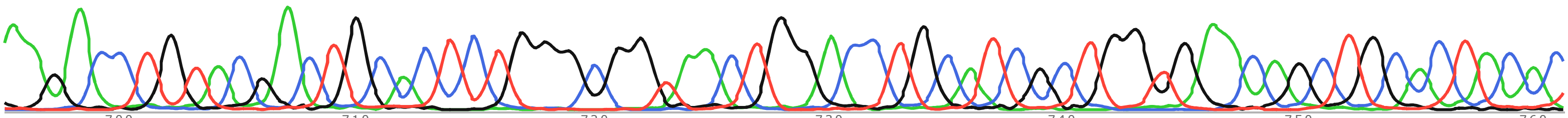

T G G T G G T A C C A T C G A A A G C T G C A A G T G G T G C G G C T A C C G T T T C C T G A A G A G C G A G G G C C T G C C T

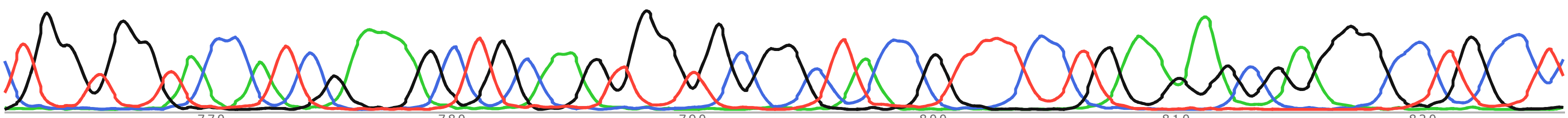

C A C T A C C C T A T C G G T A A A T G C C G T C T G A A G A A C G A G A C T G G C T A C C G T C T G G T C G A C G A C A C T

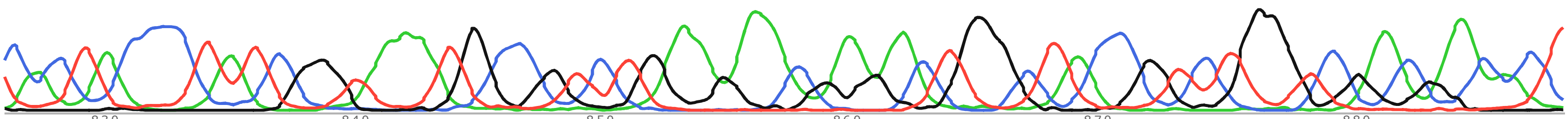

T C C T G C AA CC G C G A C GG C G T G G CC A T C G T G C C T C A GGG T A C C G T G AAA G T G C C G C A T C G

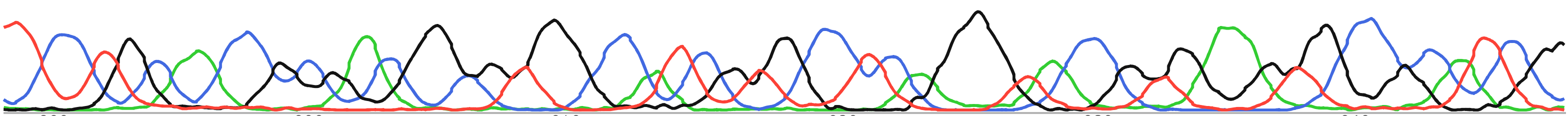

G C G A C A C T G T C G T G C A G T C A TTT G CC A T GG A C A C C AAA G C T G G G T CC T A T G C C C T G

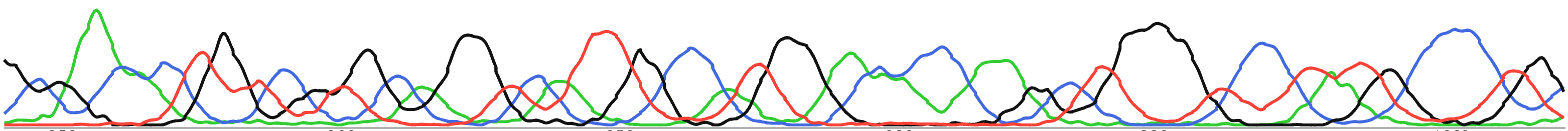

C A G C C T C

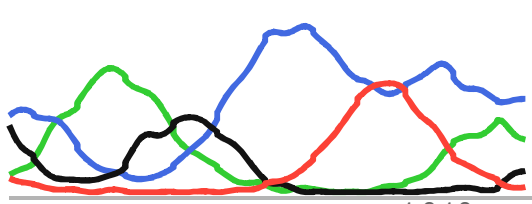

GCGTGGTCTTCGAAAAAGATCTTCGACGGCCCAGAAGAGCGCCGACACTATCGAAATGGACGACAA

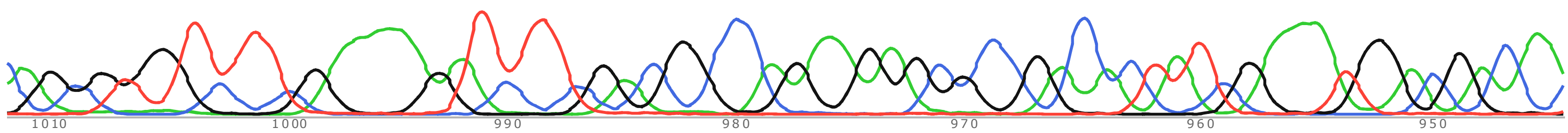

CTTCGAGTTCGCTCTCTGCCCGTGCGACGCTAAGCCTGTGGTGCGCGGTAAATTCAACACCACTCT

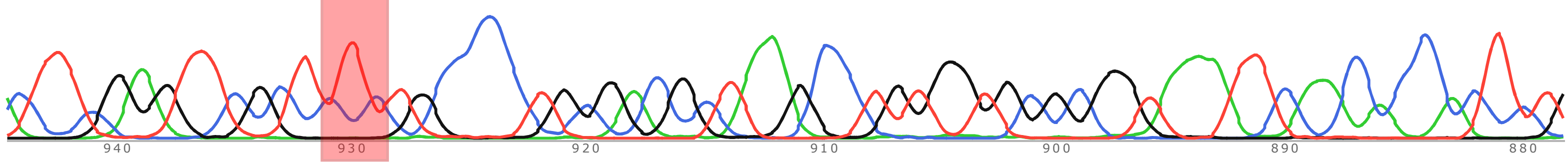

GCTGAACGGCCCCGCTTTCCAGATGGTCTGCCCTATCGGTTGGACTGGCACCGTCAACTGCGTGCTGAC

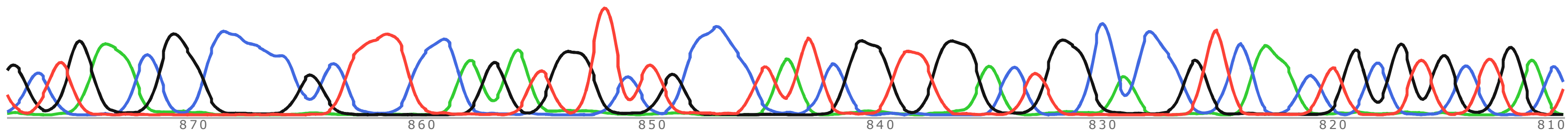

TAACAAGGACACCCTGGACATGGTCGTGGTCCAGACTTACAAGCGCGGCAGCCCTTTCCCCAACCGTCA

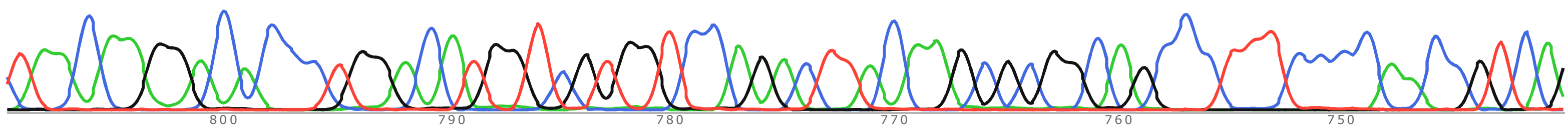

GGGCTGCGTCACTCAGAAGCTGCTGGGCGAAGACCTGTACGACTGCACTCTGGGCGGTAACTGGACCTGC

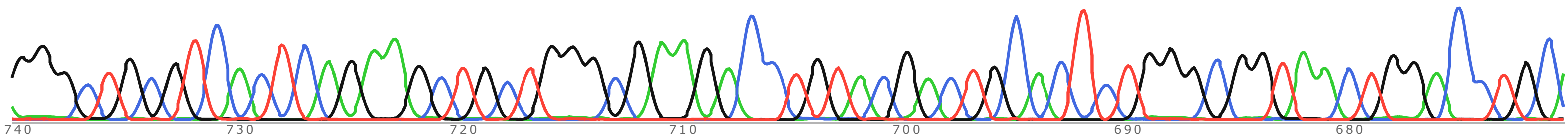

ATCGCTGGTGAACAGCTGCACTACACTGGTGGTACCATCGAAAGCTGCAAGTGGTGCGGCTACCGTTTCC

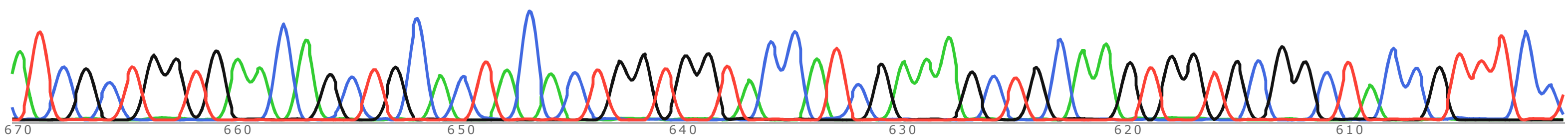

TGAAGAGCGAGGGCCTGCCTCACTACCCTATCGGTAAATGCCGTCTGAAGAACGAGACTGGCTACCGTCT

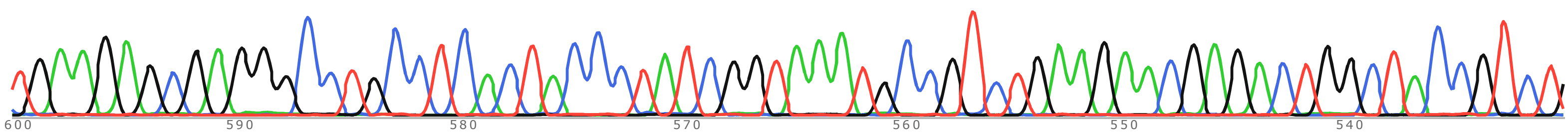

GGTCGACGACACTTCCTGCAACCGCGACGGCGTGGCCATCGTGCCTCAGGGTACCGTGAAGTGCCGCATC

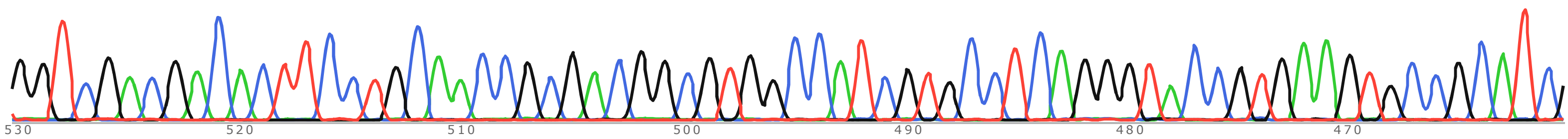

GGCGACACTGTCGTGCAGGTCATTGCCATGGACACCAAGCTGGGTCCTATGCCCTGCAAGCCTCACGAAA

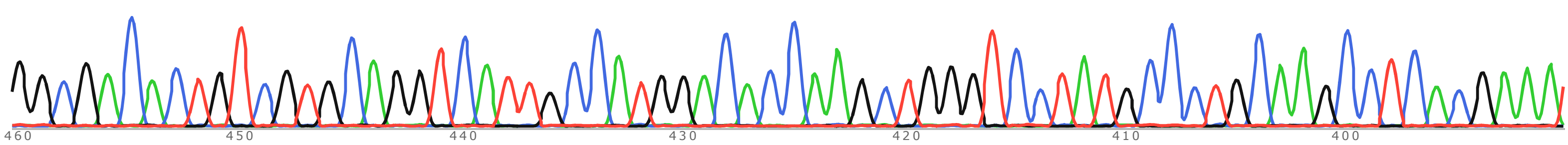

BEP-500-pFsat Bac1-BVDV-BJ1201-E2 (I103L)_SV40-pA-R_2023-08-13_A07.ab1 (1010 bases)

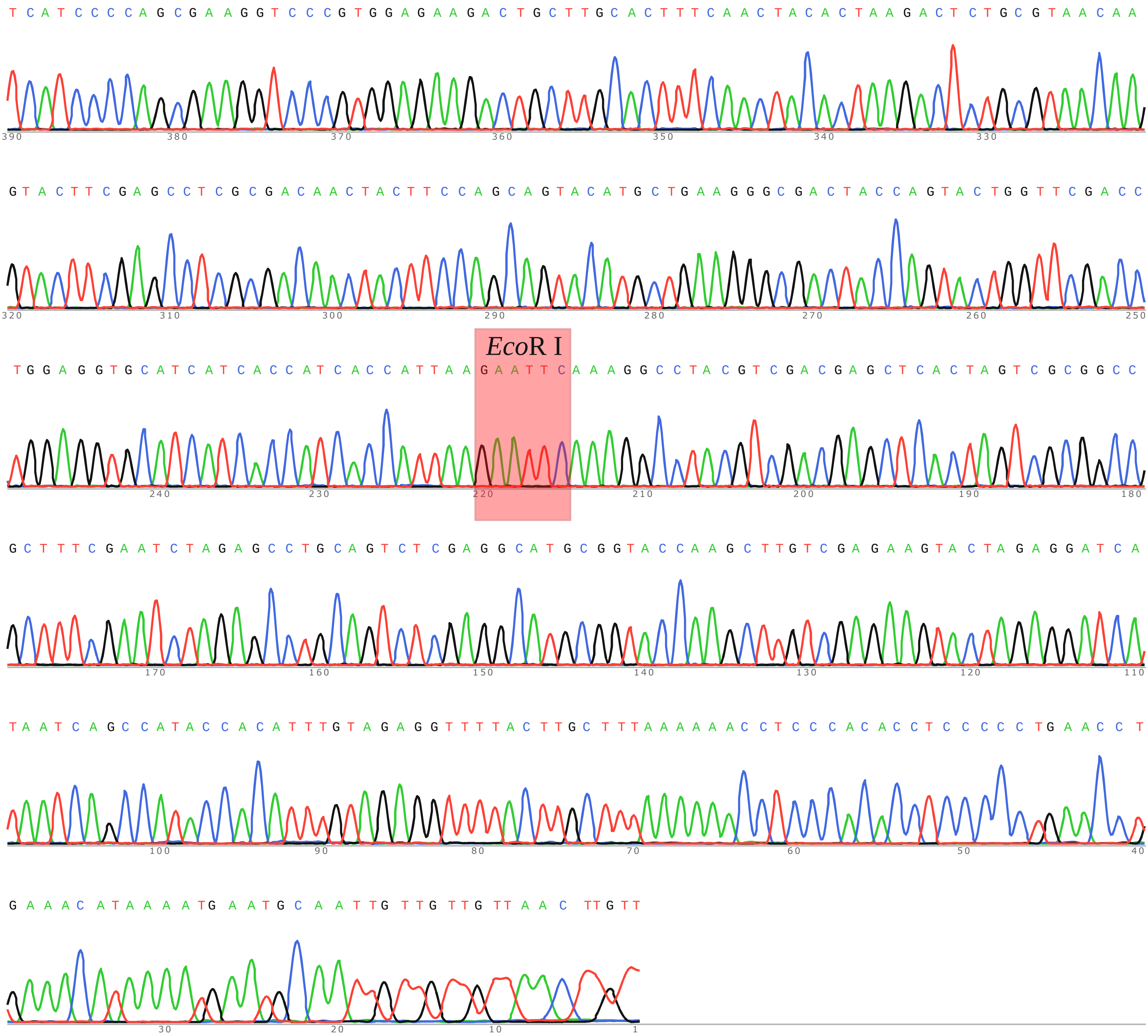

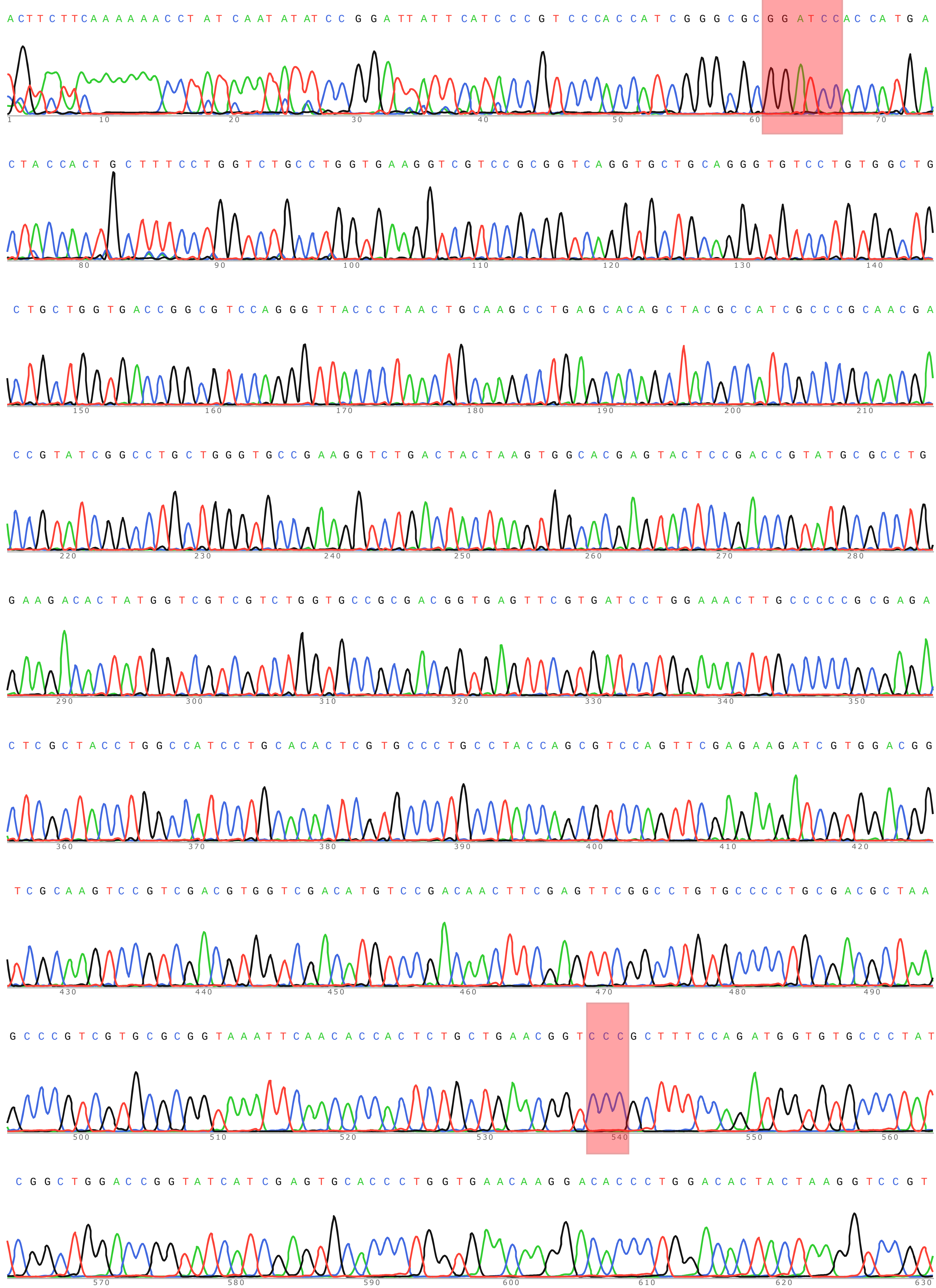
BamH I
ACTTCTTCAAAAAACCTATCAATATATCCGGATTATTCATCCCGTCCCACCATCGGGCGCGGATCCACCATGA
CTACCACTGCTTTCCTGGTCTGCCTGGTGAAGGTCGTCCGCGGTCAGGTGCTGCAGGGTGTCCTGTGGCTG
CTGCTGGTGACCGGCGTCCAGGGTTACCCTAACTGCAAGCCTGAGCACAGCTACGCCATCGCCCGCAACGA
CCGTATCGGCCTGCTGGGTGCCGAAGGTCTGACTACTAAGTGGCACGAGTACTCCGACCGTATGCGCCTG
GAAGACACTATGGTCGTCGTCTGGTGCCGCGACGGTGAGTTCGTGATCCTGGAAACTTGCCCCCGCGAGA
CTCGCTACCTGGCCATCCTGCACACTCGTGCCCTGCCTACCAGCGTCCAGTTCGAGAAGATCGTGGACGG
TCGCAAGTCCGTCGACGTGGTCGACATGTCCGACAACTTCGAGTTCGGCCTGTGCCCCTGCGACGCTAA
GCCCGTCGTGCGCGGTAAATTCAACACCACTCTGCTGAACGGTCCCGCTTTCCAGATGGTGTGCCCTAT
CGGCTGGACCGGTATCATCGAGTGCACCCTGGTGAACAAGGACACCCTGGACACTACTAAGGTCCGT

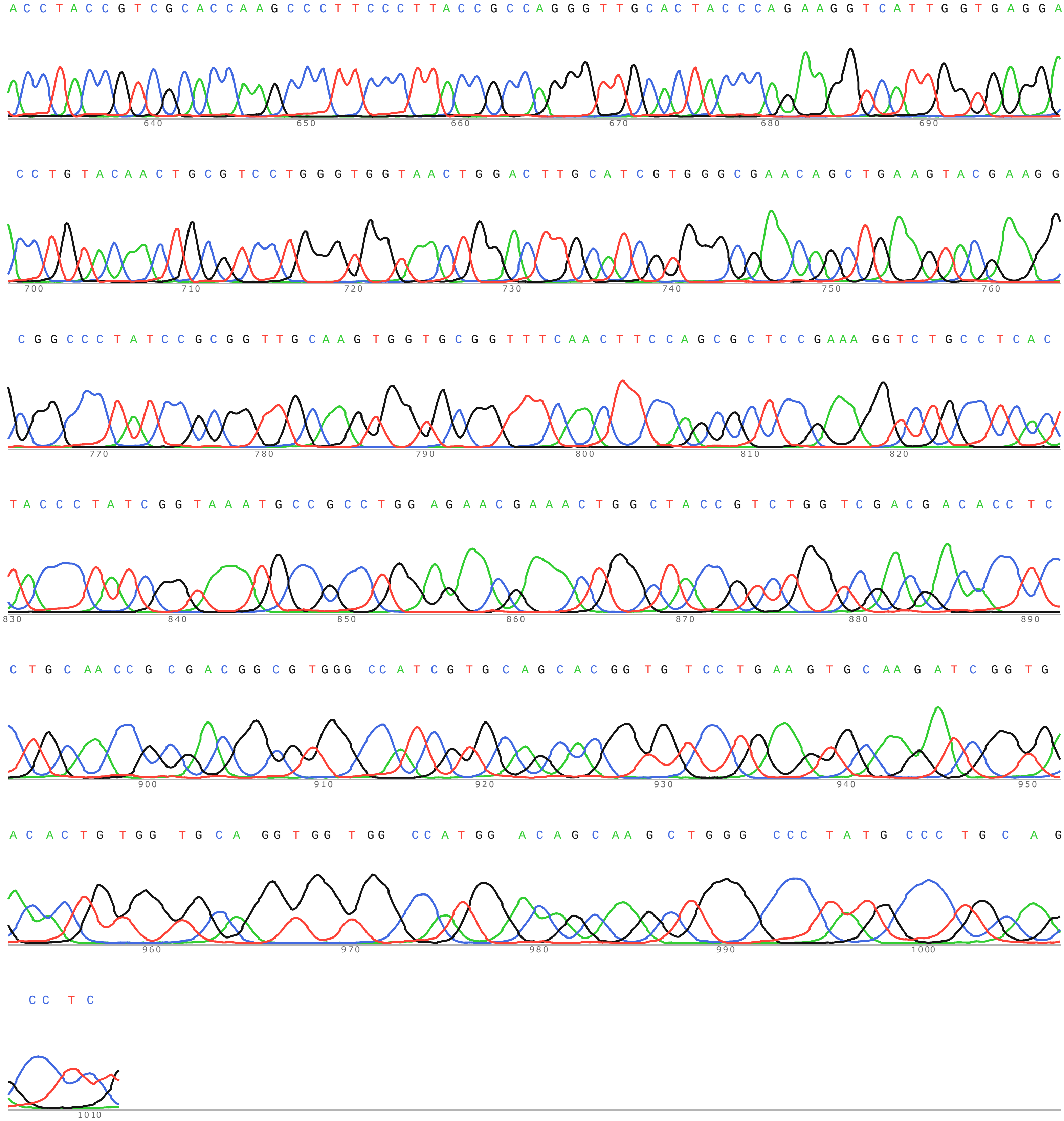
A C C T A C C G T C G C A C C A A G C C C T T C C C T T A C C G C C A G G G T T G C A C T A C C C A G A A G G T C A T T G G T G A G G A
C C T G T A C A A C T G C G T C C T G G G T G G T A A C T G G A C T T G C A T C G T G G G C G A A C A G C T G A A G T A C G A A G G
C G G C C C T A T C C G C G G T T G C A A G T G G T G C G G T T T C A A C T T C C A G C G C T C C G A A A G G T C T G C C T C A C
T A C C C T A T C G G T A A A T G C C G C C T G G A G A A C G A A A C T G G C T A C C G T C T G G T C G A C G A C A C C T C
C T G C A A C C G C G A C G G C G T G G G C C A T C G T G C A G C A C G G T G T C C T G A A G T G C A A G A T C G G T G
A C A C T G T G G T G C A G G T G G T G G C C A T G G A C A G C A A G C T G G G C C C T A T G C C C T G C A G
C C T C

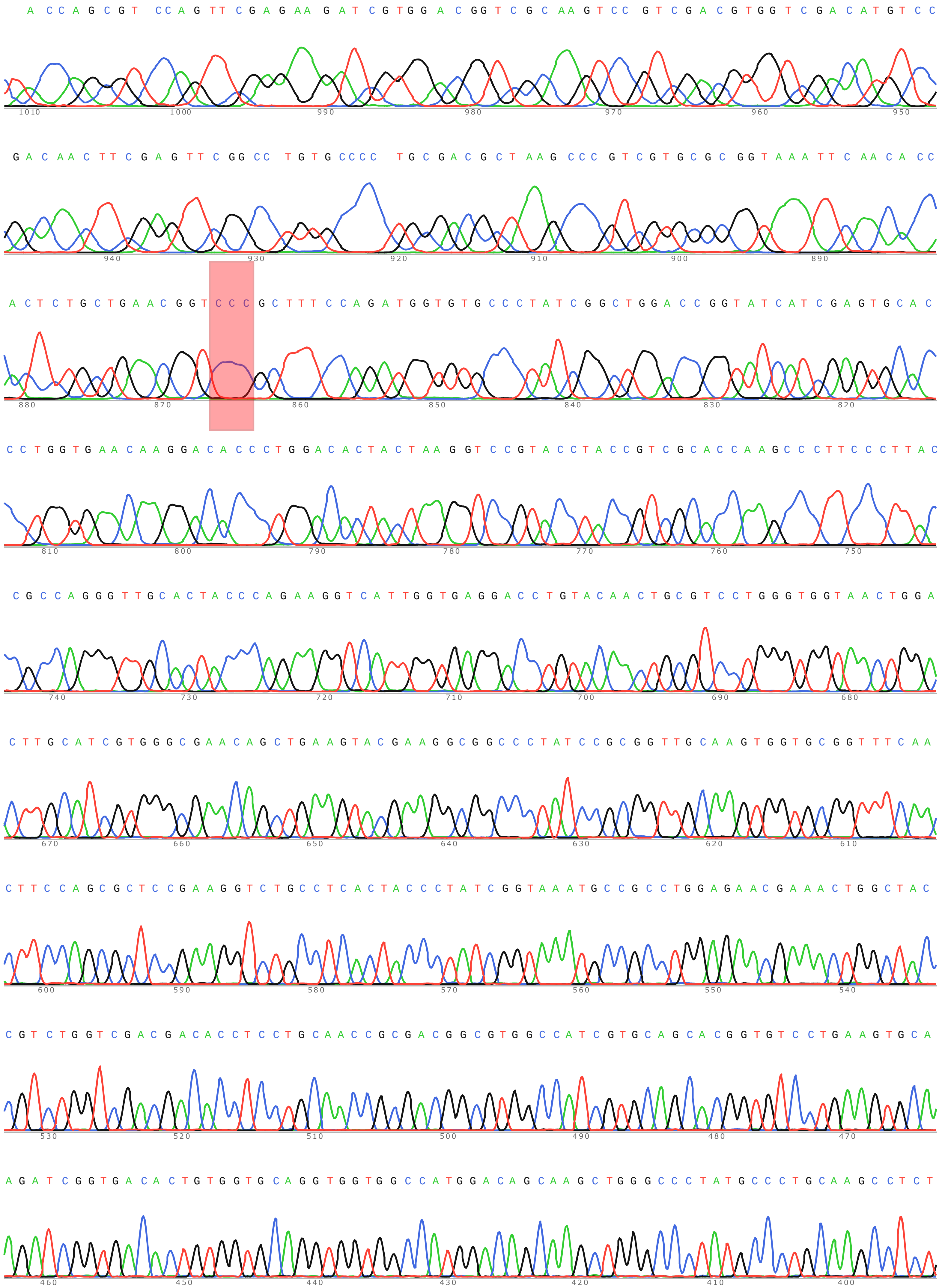
ACCAGCGTCCAGTTCGAGAAGATCGTGGACGGTCGCAAGTCCGTCGACGTGGTCGACATGTCC
1010 1000 990 980 970 960 950
GACAACTTCGAGTTCGGCCTGTGCCCCTGCGACGCTAAGCCCGTCGTGCGCGGTAAATTCAACACC
940 930 920 910 900 890
ACTCTGCTGAACGGTCCCGCTTTCCAGATGGTGTGCCCTATCGGCTGGACCGGTATCATCGAGTGCAC
880 870 860 850 840 830 820
CCTGGTGAACAAGGACACCCTGGACACTACTAAGGTCCGTACCTACCGTCGCACCAAGCCCTTCCCTTAC
810 800 790 780 770 760 750
CGCCAGGGTTGCACTACCCAGAAGGTCATTGGTGAGGACCTGTACAACTGCGTCCTGGGTGGTAACTGGA
740 730 720 710 700 690 680
CTTGCATCGTGGGCGAACAGCTGAAGTACGAAGGCGGCCCTATCCGCGGTTGCAAGTGGTGCGGTTTCAA
670 660 650 640 630 620 610
CTTCCAGCGCTCCGAAGGTCTGCCTCACTACCCTATCGGTAAATGCCGCCTGGAGAACGAAACTGGCTAC
600 590 580 570 560 550 540
CGTCTGGTCGACGACACCTCCTGCAACCGCGACGGCGTGGCCATCGTGCAGCACGGTGTCCTGAAGTGCA
530 520 510 500 490 480 470
AGATCGGTGACACTGTGGTGCAGGTGGTGGCCATGGACAGCAAGCTGGGCCCTATGCCCTGCAAGCCTCT
460 450 440 430 420 410 400

G G A G A T C A C T C C T T C C G A G G G C C C T G T G G A A A A G A C T G C T T G C A C T T T C A A C T A C A C C A A G A C T C T G A A G

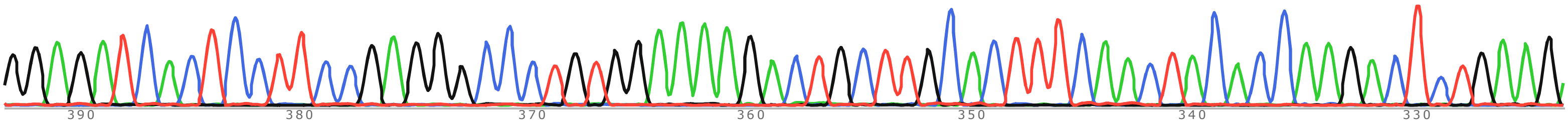

A A C A A G T A C T T C G A A C C T C G C G A C A G C T A C T T C C A G C A G T A C A T G C T G A A G G G C G G T T A C C A G T A C T G G T T

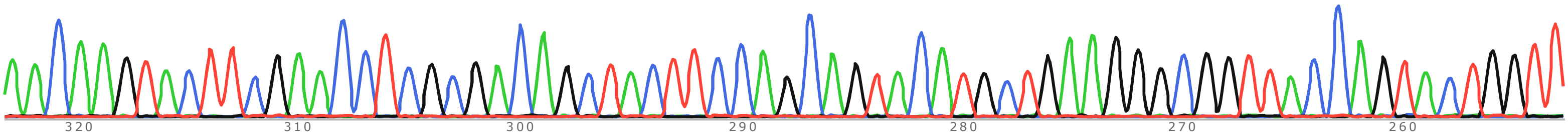

C G A C C T G G A C G C T C A T C A T C A C C A T C A C C A T T A A G A A T T C A A A G G C C T A C G T C G A C G A G C T C A C T A G T C G

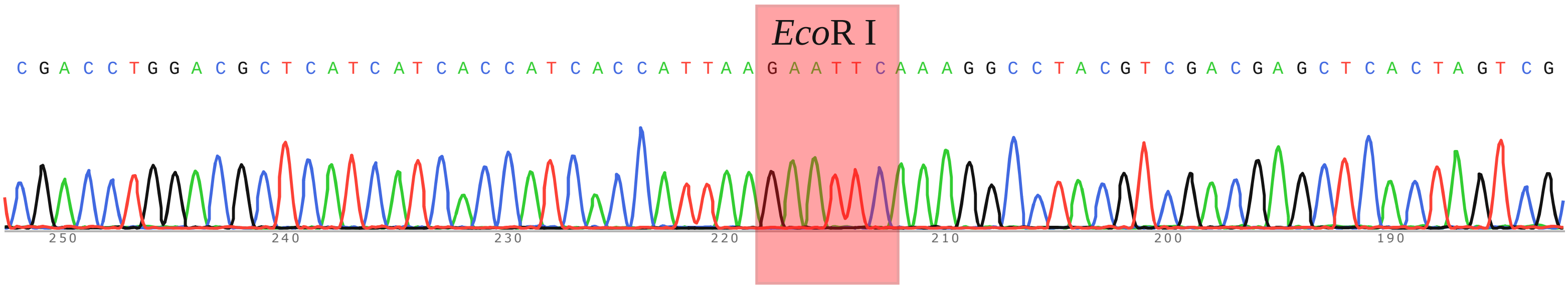

C G G C C G C T T T C G A A T C T A G A G C C T G C A G T C T C G A G G C A T G C G G T A C C A A G C T T G T C G A G A A G T A C T A G A G

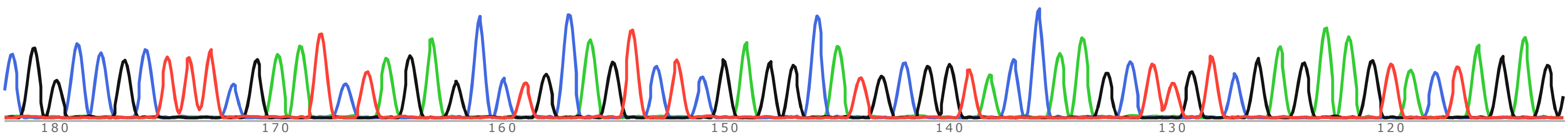

G A T C A T A A T C A G C C A T A C C A C A T T T G T A G A G G T T T T A C T T G C T T T A A A A A C C T C C C A C A C C T C C C C C T G

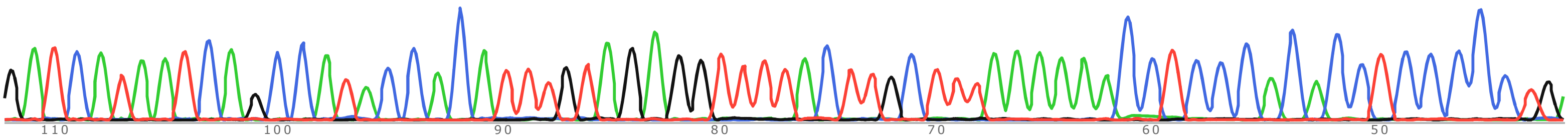

A A C C T G A A A C A T A A A A T G A A T G C A A T T G T T G T T G T T A A C T T T

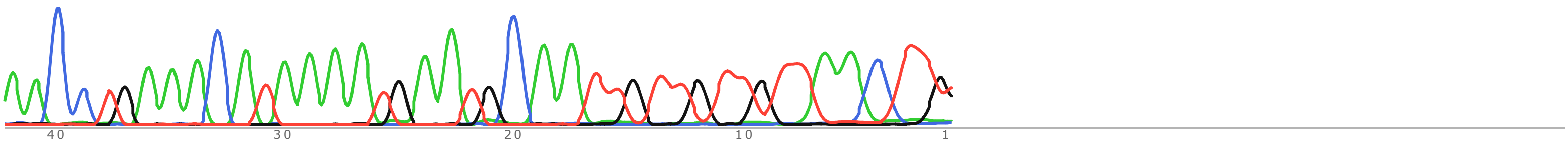

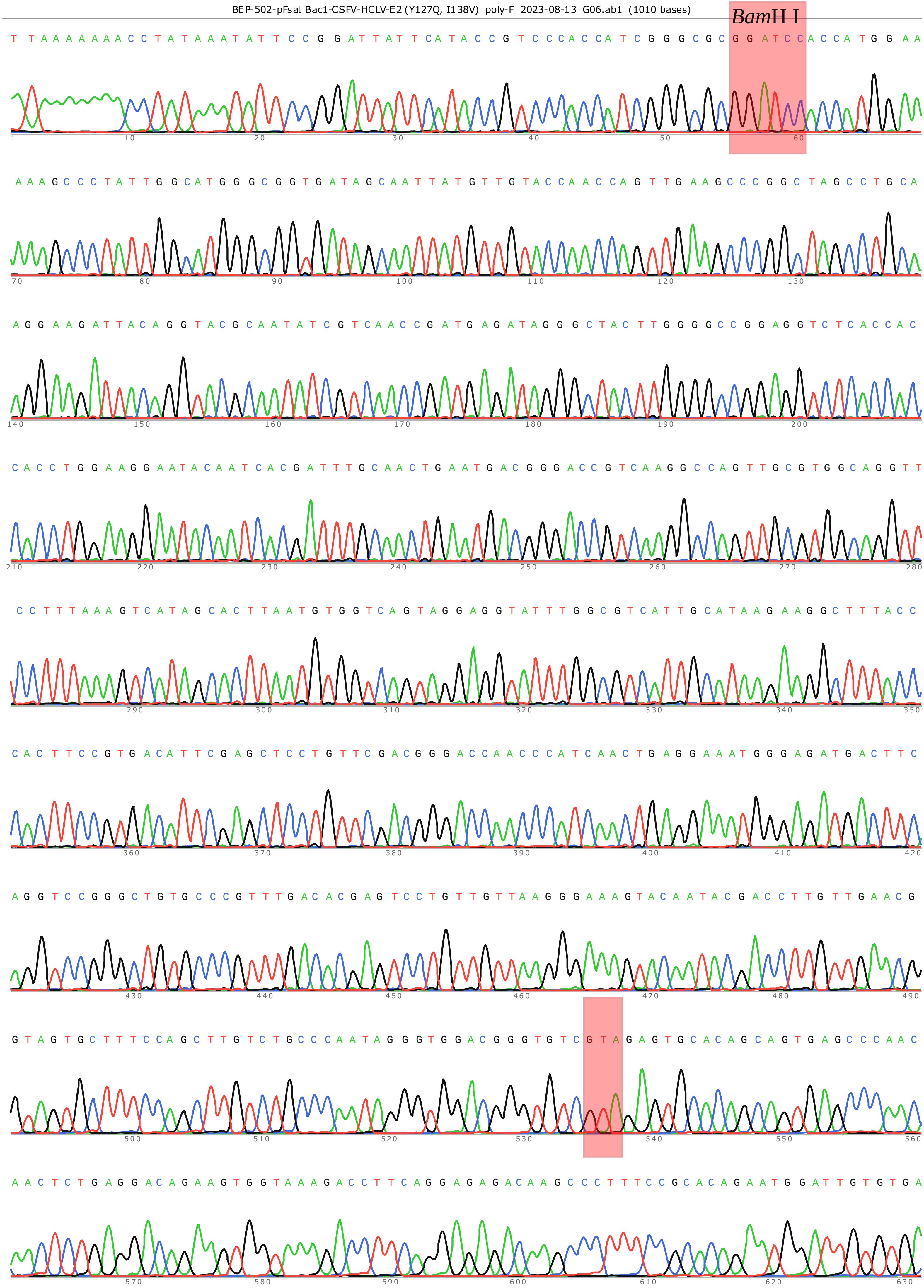
*Bam*H I
T T A A A A A A A C C T A T A A A T A T T C C G G A T T A T T C A T A C C G T C C C A C C A T C G G G C G C G G A T C C A C C A T G G A A
A A A G C C C T A T T G G C A T G G G C G G T G A T A G C A A T T A T G T T G T A C C A A C C A G T T G A A G C C C G G C T A G C C T G C A
A G G A A G A T T A C A G G T A C G C A A T A T C G T C A A C C G A T G A G A T A G G G C T A C T T G G G G C C G G A G G T C T C A C C A C
C A C C T G G A A G G A A T A C A A T C A C G A T T T G C A A C T G A A T G A C G G G A C C G T C A A G G C C A G T T G C G T G G C A G G T T
C C T T T A A A G T C A T A G C A C T T A A T G T G G T C A G T A G G A G G T A T T T G G C G T C A T T G C A T A A G A A G G C T T T A C C
C A C T T C C G T G A C A T T C G A G C T C C T G T T C G A C G G G A C C A A C C C A T C A A C T G A G G A A A T G G G A G A T G A C T T C
A G G T C C G G G C T G T G C C C G T T T G A C A C G A G T C C T G T T G T T A A G G G A A A G T A C A A T A C G A C C T T G T T G A A C G
G T A G T G C T T T C C A G C T T G T C T G C C C A A T A G G G T G G A C G G G T G T C G T A G A G T G C A C A G C A G T G A G C C C A A C
A A C T C T G A G G A C A G A A G T G G T A A A G A C C T T C A G G A G A G A C A A G C C C T T T C C G C A C A G A A T G G A T T G T G T G A

CCACCACAGTGGAAAATGAAGATTTATTCTATTGTAAGTTGGGGGGCAACTGGACATGTGTGAAAGGCGAG

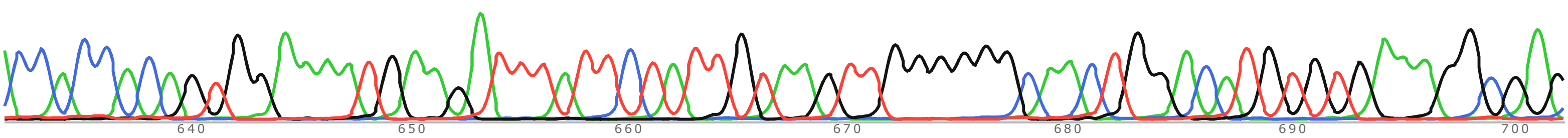

CCAGTGGTCTACACAGGGGGGGTAGTAAAACAATGTAGATGGTGTGGCTTCGACTTCGATGGGCCTGACG

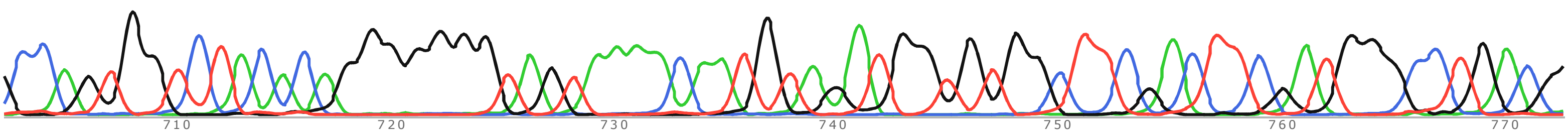

GACTCCCGCATTACCCCATAGGTAAGTGCATTTTTGGCAAATGAGACAGGTTACAGAATAGTAGATTCAAC

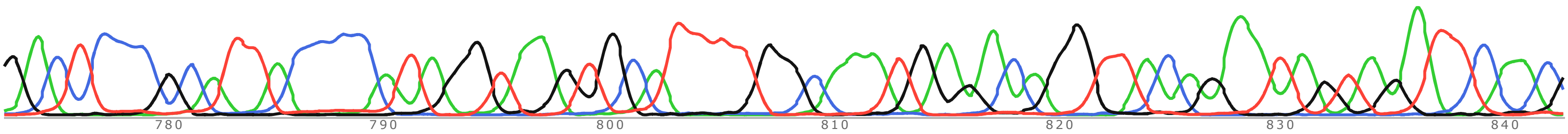

GGACTGTAACAGAGATGGCGTTGTAATCAGCACAGAGGGGAGTCATGAGTGCTTGATCGGTAACACGA

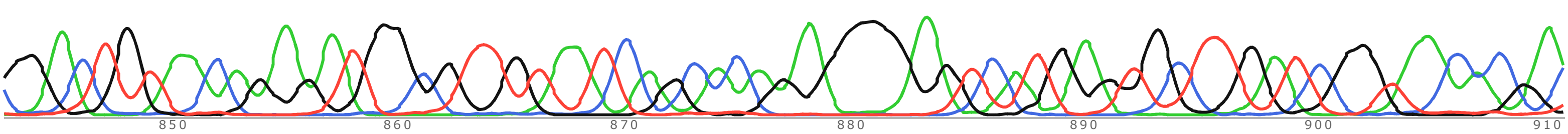

CTGTCAA GGTGCATGCATCAGATGAA GACTGGG CCC TATG CCATGCAGACC TAAAGAGATT GTCTC

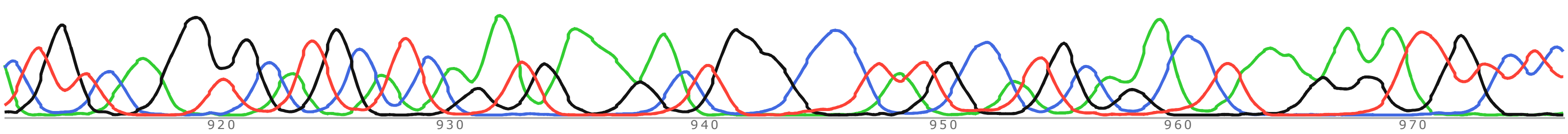

TAG TGCTGG TCCTG TAAAGAAAA CCTCC TGTACA

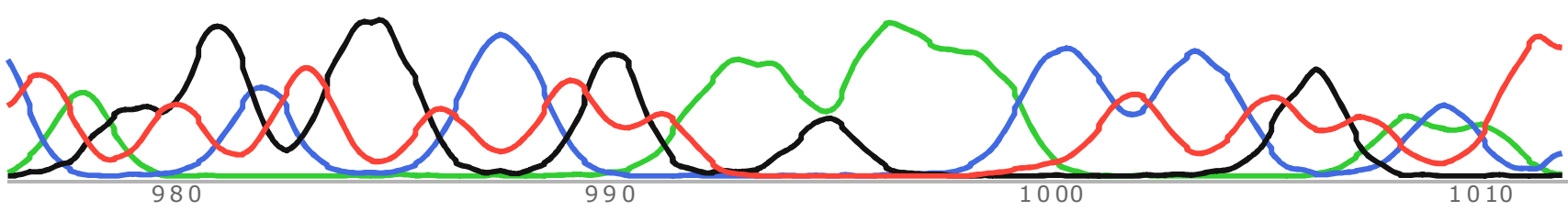

BEP-502-pFsat Bac1-CSFV-HCLV-E2 (Y127Q, I138V)_SV40-pA-R_2023-08-13_G07.ab1 (1010 bases)

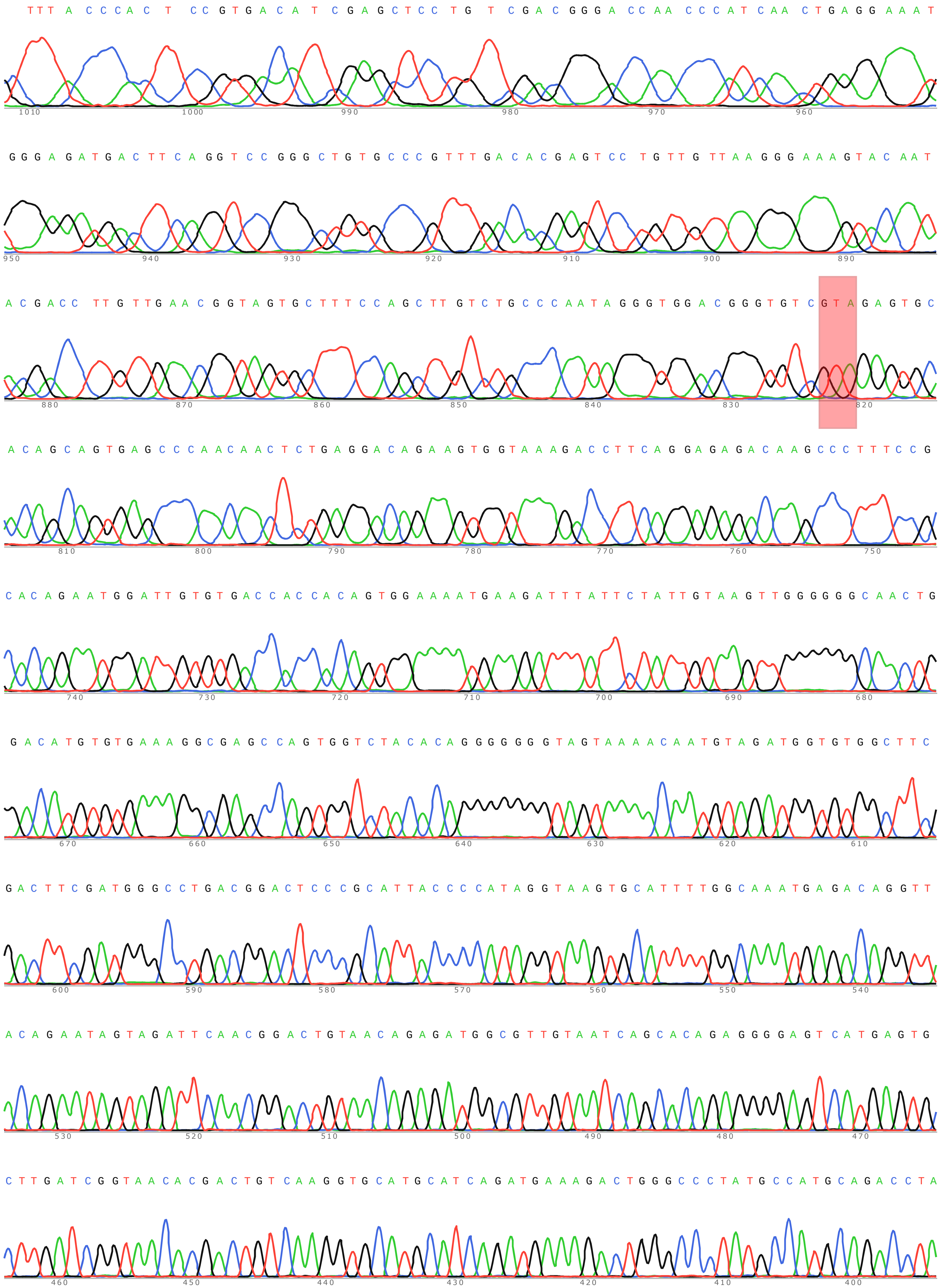

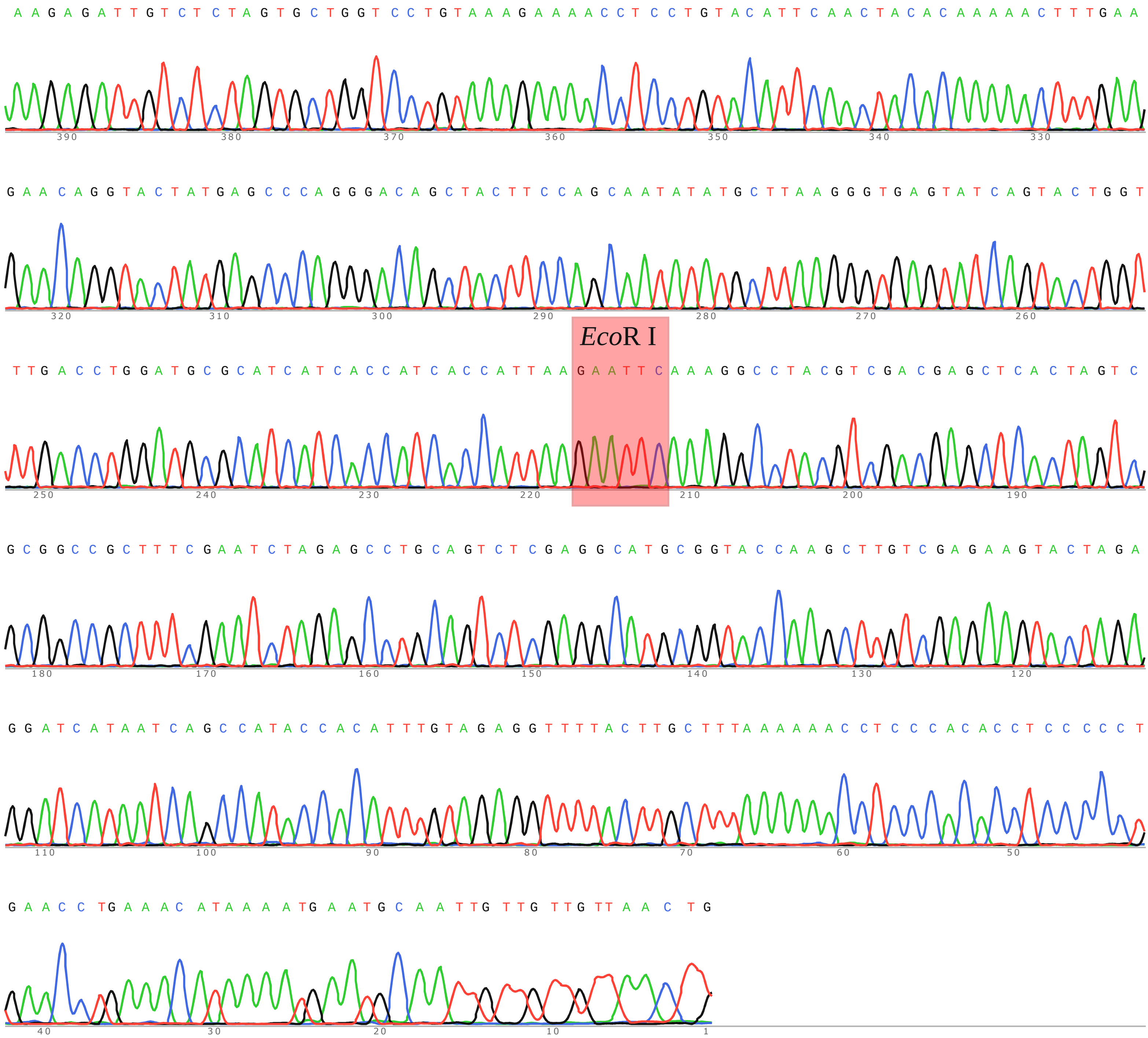
AAGAGATTGTCTCTAGTGCTGGTCCTGTAAAGAAAACCTCCTGTACATTCAACTACACAAAAACTTTGAA
GAACAGGTACTATGAGCCCAGGGACAGCTACTTCCAGCAATATATGCTTAAGGGTGAGTATCAGTACTGGT
EcoR I
TTGACCTGGATGCGCATCATCACCATCACCATTAAGAATTCAAAGGCCTACGTCGACGAGCTCACTAGTC
GCGGCCGCTTTCGAATCTAGAGCCTGCAGTCTCGAGGCATGCGGTACCAAGCTTGTCGAGAAGTACTAGA
GGATCATAATCAGCCATACCACATTTGTAGAGGTTTTACTTGCTTTAAAAACCTCCCACACCTCCCCCT
GAACCTGAAACATAAAATGAATGCAATTGTTGTTGTTAACTG

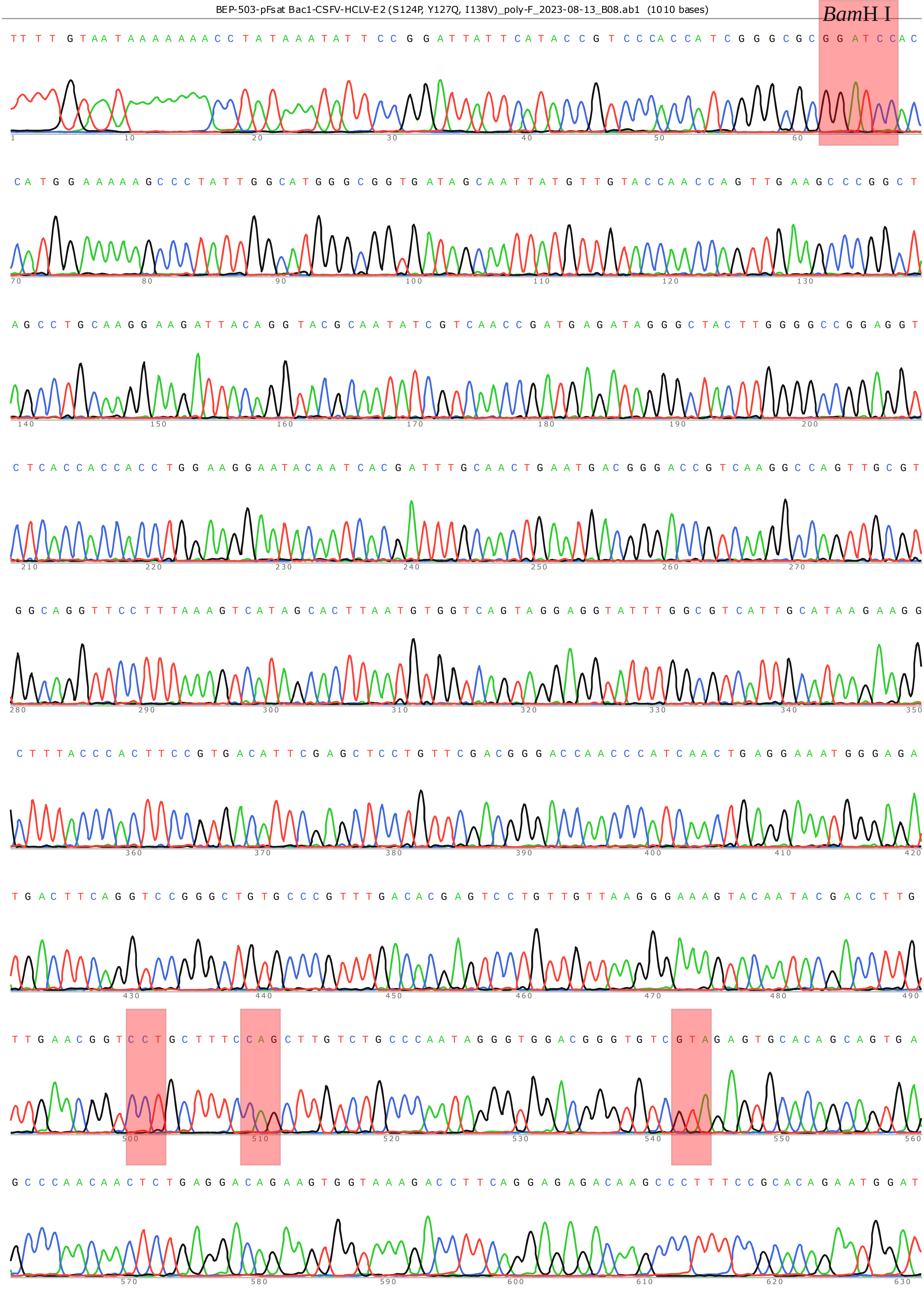
BamH I
TTTTGTAATAAAAAAACCTATAAATATTCCGGATTATTCATACCGTCCCACCATCGGGCGCGGATCCAC
1 10 20 30 40 50 60
CATGGAAAAAGCCCTATTGGCATGGGCGGTGATAGCAATTATGTTGTACCAACCAGTTGAAGCCCGGCT
70 80 90 100 110 120 130
AGCCTGCAAGGAAGATTACAGGTACGCAATATCGTCAACCGATGAGATAGGGCTACTTGGGGCCGGAGGT
140 150 160 170 180 190 200
CTCACCACCACCTGGAAGGAATACAATCACGATTTGCAACTGAATGACGGGACCGTCAAGGCCAGTTGCGT
210 220 230 240 250 260 270
GGCAGGTTCCTTTAAAGTCATAGCACTTAATGTGGTCAGTAGGAGGTATTTGGCGTCATTGCATAAGAAGG
280 290 300 310 320 330 340 350
CTTTACCCACTTCCGTGACATTCGAGCTCCTGTTCGACGGGACCAACCCATCAACTGAGGAAATGGGAGA
360 370 380 390 400 410 420
TGACTTCAGGTCCGGGCTGTGCCCGTTTGACACGAGTCCTGTTGTTAAGGGAAAGTACAATACGACCTTG
430 440 450 460 470 480 490
TTGAACGGTCCTGCTTTCCAGCTTGTCTGCCCAATAGGGTGGACGGGTGTCGTAGAGTGCACAGCAGTGA
500 510 520 530 540 550 560
GCCCAACAACTCTGAGGACAGAAGTGGTAAAGACCTTCAGGAGAGACAAGCCCTTTCCGCACAGAATGGAT
570 580 590 600 610 620 630

T G T G T G A C C A C C A C A G T G G A A A A T G A A G A T T T A T T T C T A T T G T A A G T T G G G G G G C A A C T G G A C A T G T G T G A A

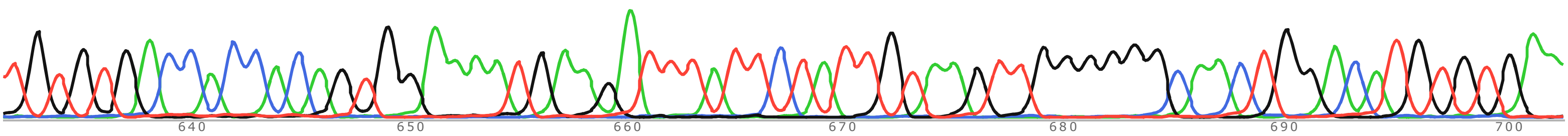

A G G C G A G C C A G T G G T C T A C A C A G G G G G G G T A G T A A A A C A A T G T A G A T G G T G T G G C T T C G A C T T C G A T G G G C

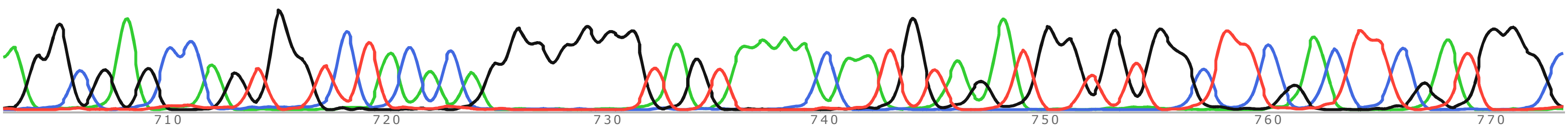

C T G A C G G A C T C C C G C A T T A C C C C A T A G G T A A G T G C A T T T T G G C A A A T G A G A C A G G T T A C A G A A T A G T A G A

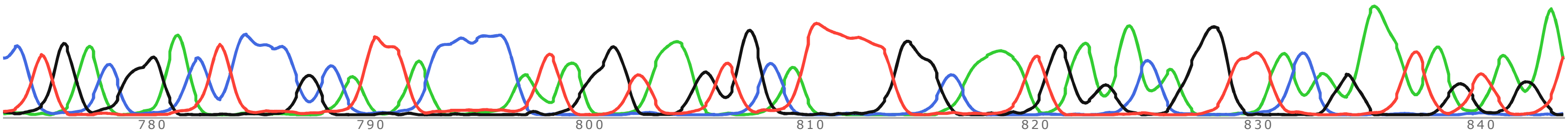

T T C A A C G G A C T G T A A C A G A G A T G G C G T T G T A A T C A G C A C A G A G G G G A G T C A T G A G T G C T T G A T C G G T A A

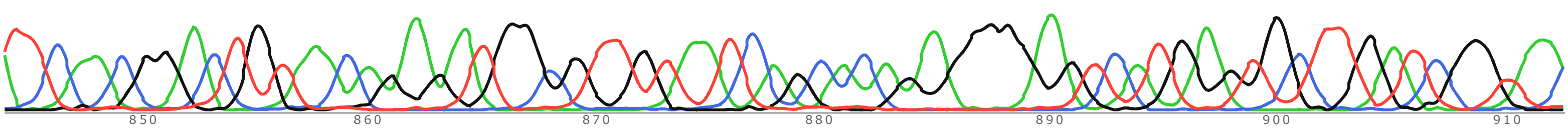

C A C G A C T G T C A A G G T G C A T G C A T C A G A T G A A A G A C T G G G C C C T A T G C C A T G C A G A C C T A A A G A G A T T G

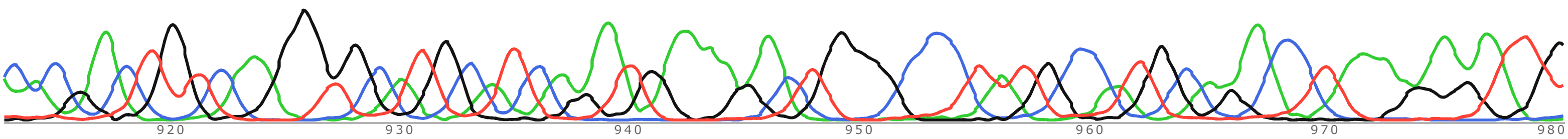

T C T C T A G T G C T G G T C C T G T A A A G A A A A C C T

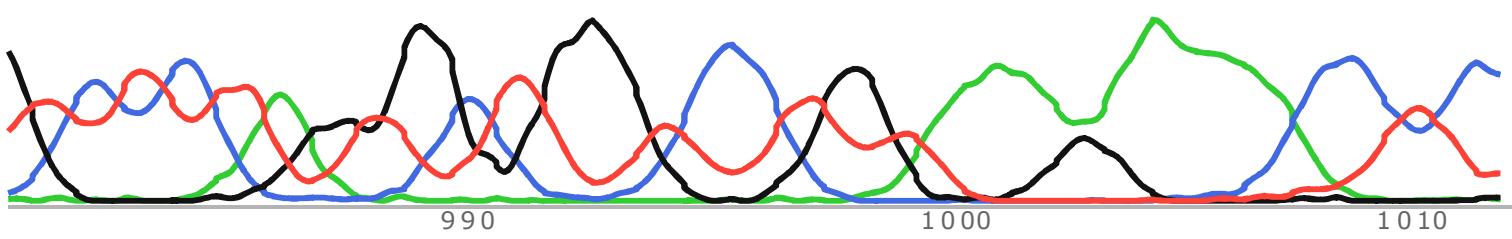

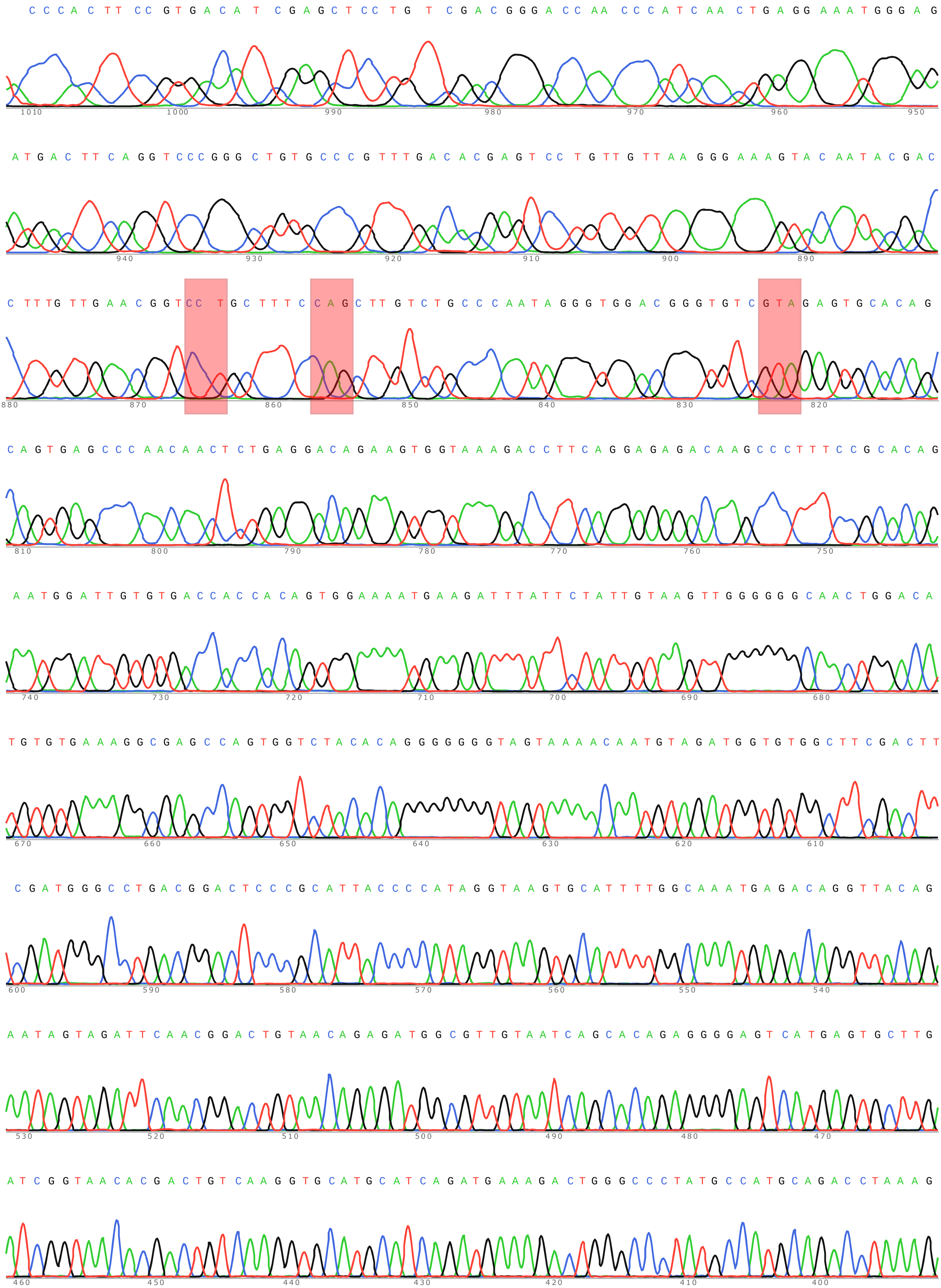
CCCACTTCCGTGACATCGAGCTCCTGTCGACGGGACCAACCCATCAACTGAGGAAATGGGAG
ATGACTTCAGGTCCCGGGCTGTGCCCGTTTGACACGAGTCCTGTTGTTAAGGGAAAGTACAATACGAC
CTTTGTTGAACGGTCCTGCTTTCCAGCTTGTCTGCCCAATAGGGTGGACGGGTGTCGTAGAGTGCACAG
CAGTGAGCCCAACAACTCTGAGGACAGAAGTGGTAAAGACCTTCAGGAGAGACAAGCCCTTTCCGCACAG
AATGGATTGTGTGACCACCACAGTGGAAAATGAAGATTTATTCTATTGTAAGTTGGGGGGCAACTGGACA
TGTGTGAAAGGCGAGCCAGTGGTCTACACAGGGGGGTAGTAAAACAATGTAGATGGTGTGGCTTCGACTT
CGATGGGCCTGACGGACTCCCGCATTACCCCATAGGTAAGTGCATTTTGGCAAATGAGACAGGTTACAG
AATAGTAGATTCAACGGACTGTAACAGAGATGGCGTTGTAATCAGCACAGAGGGGAGTCATGAGTGCTTG
ATCGGTAACACGACTGTCAAGGTGCATGCATCAGATGAAAGACTGGGCCCTATGCCATGCAGACCTAAAG

A G A T T G T C T C T A G T G C T G G T C C T G T A A A G A A A A C C T C C T G T A C A T T C A A C T A C A C A A A A A C T T T G A A G A A C

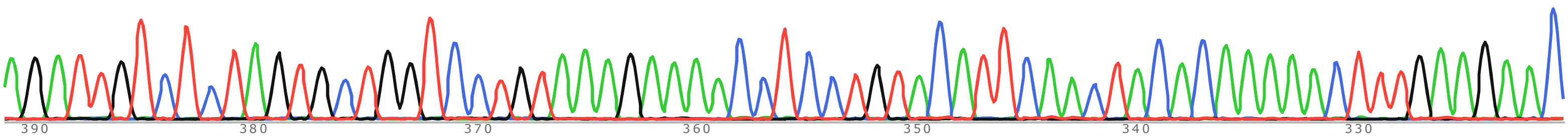

A G G T A C T A T G A G C C C A G G G A C A G C T A C T T C C A G C A A T A T A T G C T T A A G G G T G A G T A T C A G T A C T G G T T T G A

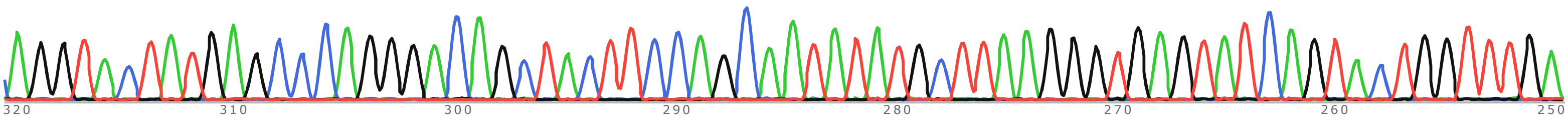

*Eco*R I

C C T G G A T G C G C A T C A T C A C C A T C A C C A T T A A G A A T T C A A A G G C C T A C G T C G A C G A G C T C A C T A G T C G C G G

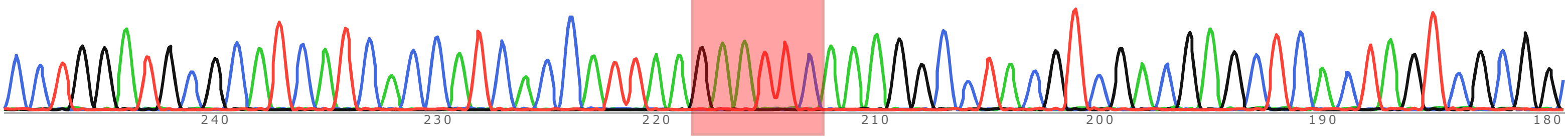

C C G C T T T C G A A T C T A G A G C C T G C A G T C T C G A G G C A T G C G G T A C C A A G C T T G T C G A G A A G T A C T A G A G G A T

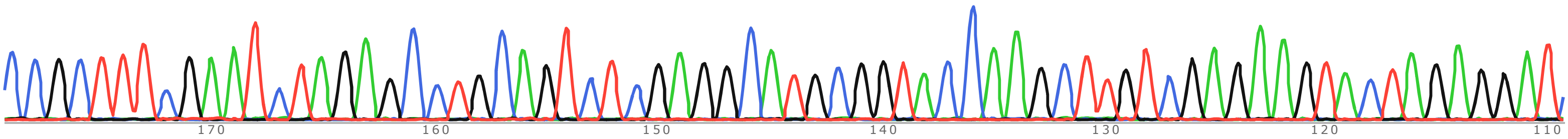

C A T A A T C A G C C A T A C C A C A T T T G T A G A G G T T T T A C T T G C T T T A A A A A C C T C C C A C A C C T C C C C T G A A C

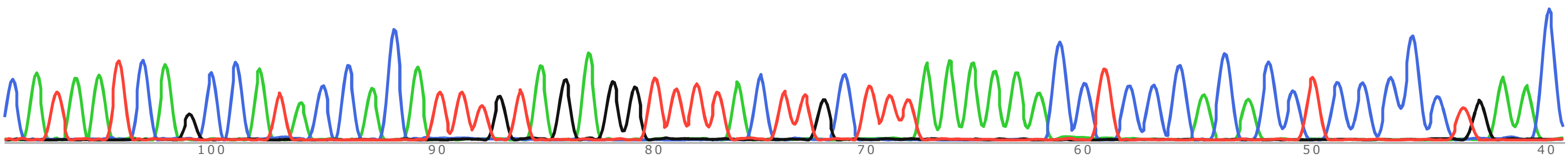

C T G A A A C A T A A A A T G A A T G C A A T T G T T G T T G T T A A C T T T

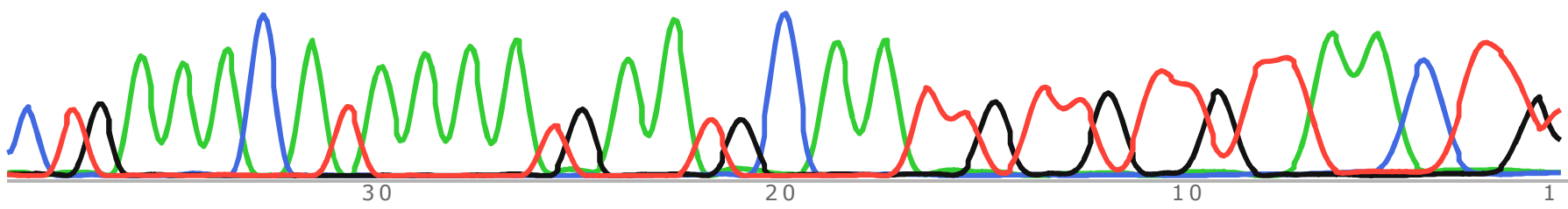

MEP-044-pcDNA3.1-BVDV-XH32-E2 (F126L)_pRLuc-MCS-F_2024-03-30_A04.ab1 (1030 bases)

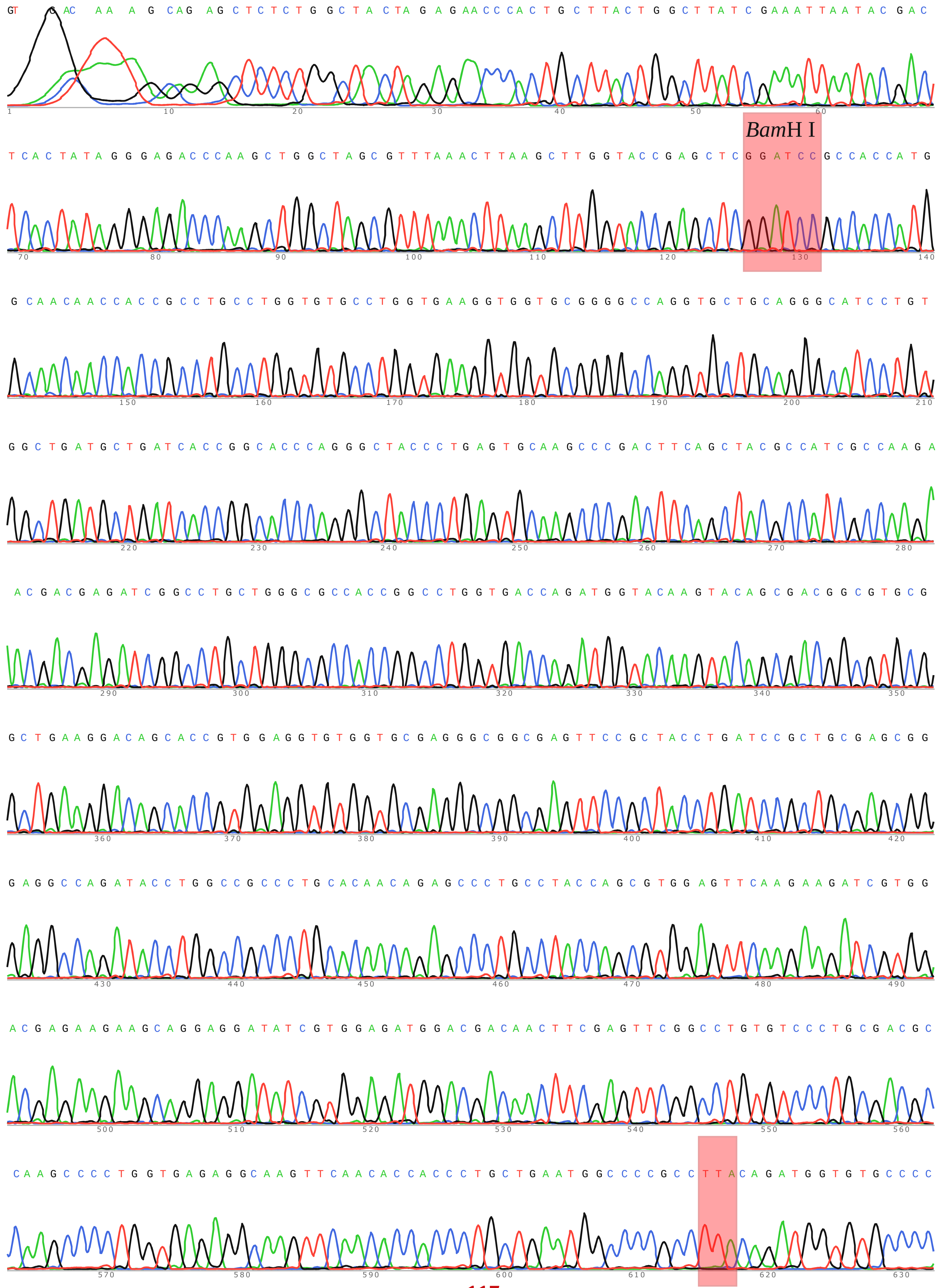

A T C G G C T G G A C C G G C A C C G T G A G C T G C A C C C T G G C C A A C G A G G A T A C C C T G G C C A C C A C C G T G G T G C G C A

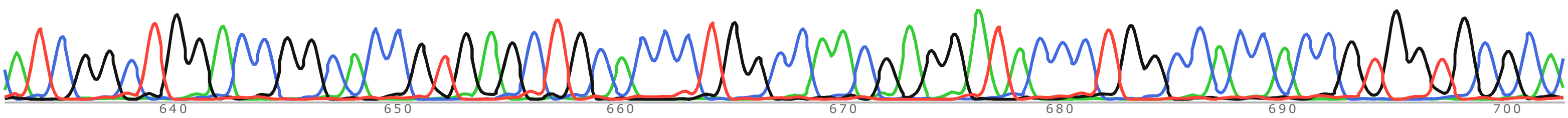

C C T A C A A G A G G G T G A G A C C C T T C C C C T A C C G G C A G G A C T G C G T G A C C C A G A A A A C C A T C G G C G A G G A C C T G

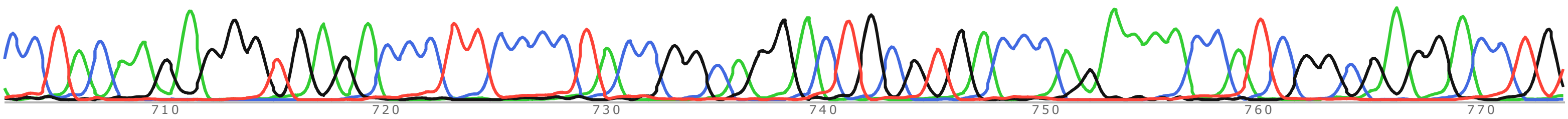

T A C G A T T G C G C C C T G G G C G G C A A C T G G A C C T G C G T G C T G G G C G A C A T C C T G A G G T A C G T G G C C G G C C C T G T G

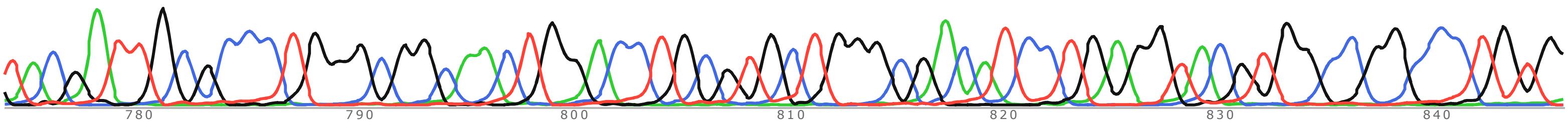

G A G T C C T G C A A G T G G T G C G G C C A C A A G T T C C T G A A G T C C G A G G G C C T G C C C C A C T T C C C C A T C G G C A A G T G C C

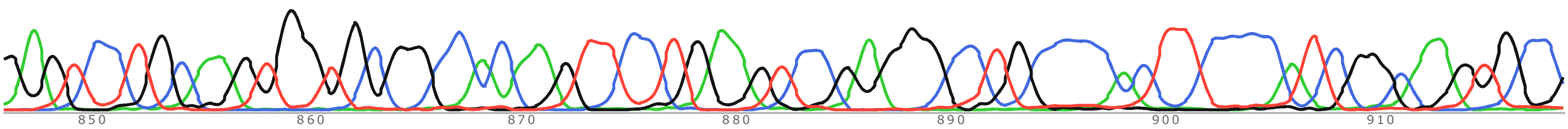

G C C T G A A G A A C G A G A G C G G C T A C A G A C T G G T G G A C G G C A C C T C C T G C A A C C G G G A C G G C G T G G C C A T C G T G C C C

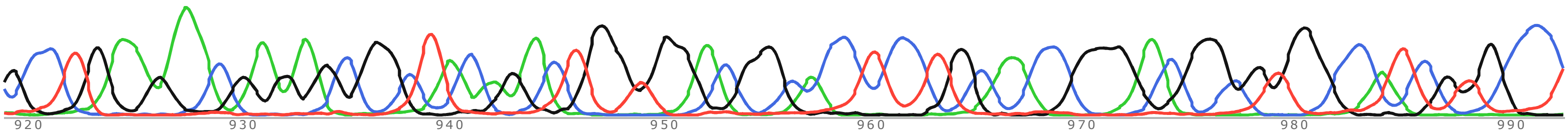

T C C G G C C G G G T G A A G T G C A A G A T C G G C G A C A C C G T G G T

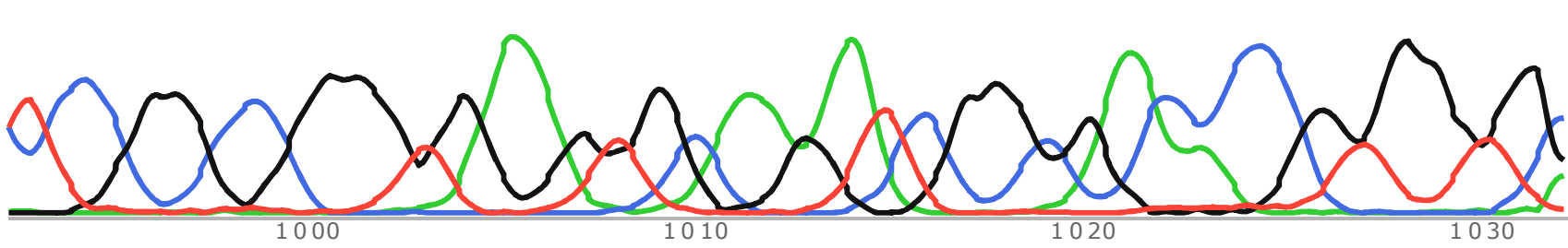

MEP-044-pcDNA3.1-BVDV-XH32-E2 (F126L)_BGH-r_2024-03-30_A05.ab1 (1030 bases)

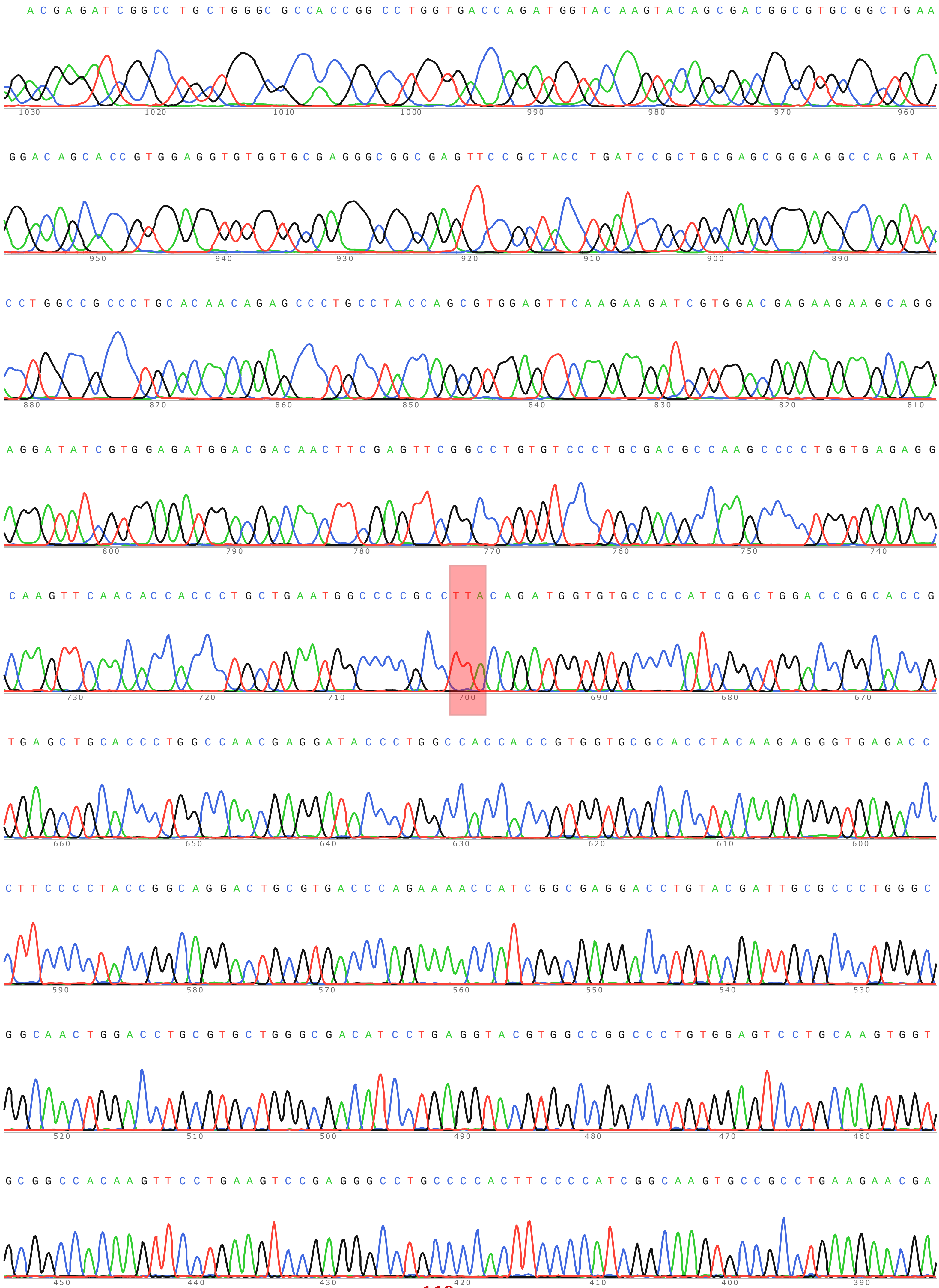

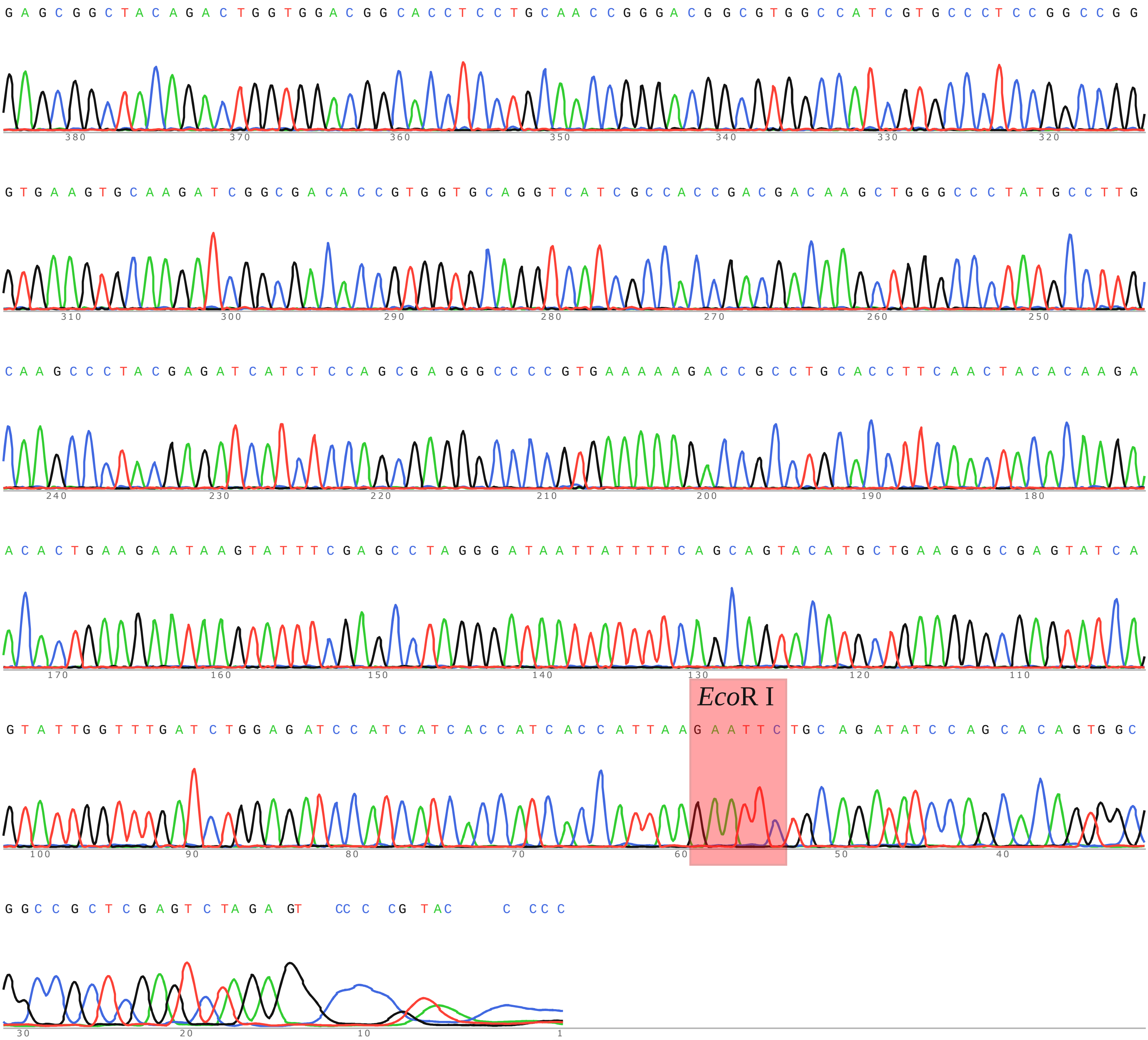
G A G C G G C T A C A G A C T G G T G G A C G G C A C C T C C T G C A A C C G G G A C G G C G T G G C C A T C G T G C C C T C C G G C C G G
380 370 360 350 340 330 320
G T G A A G T G C A A G A T C G G C G A C A C C G T G G T G C A G G T C A T C G C C A C C G A C G A C A A G C T G G G C C C T A T G C C T T G
310 300 290 280 270 260 250
C A A G C C C T A C G A G A T C A T C T C C A G C G A G G G C C C C G T G A A A A A G A C C G C C T G C A C C T T C A A C T A C A C A A G A
240 230 220 210 200 190 180
A C A C T G A A G A A T A A G T A T T T C G A G C C T A G G G A T A A T T A T T T T C A G C A G T A C A T G C T G A A G G G C G A G T A T C A
170 160 150 140 130 120 110
G T A T T G G T T T G A T C T G G A G A T C C A T C A T C A C C A T C A C C A T T A A G A A T T C T G C A G A T A T C C A G C A C A G T G G C
EcoR I
100 90 80 70 60 50 40
G G C C G C T C G A G T C T A G A G T CC C C G T A C C C C C
30 20 10 1

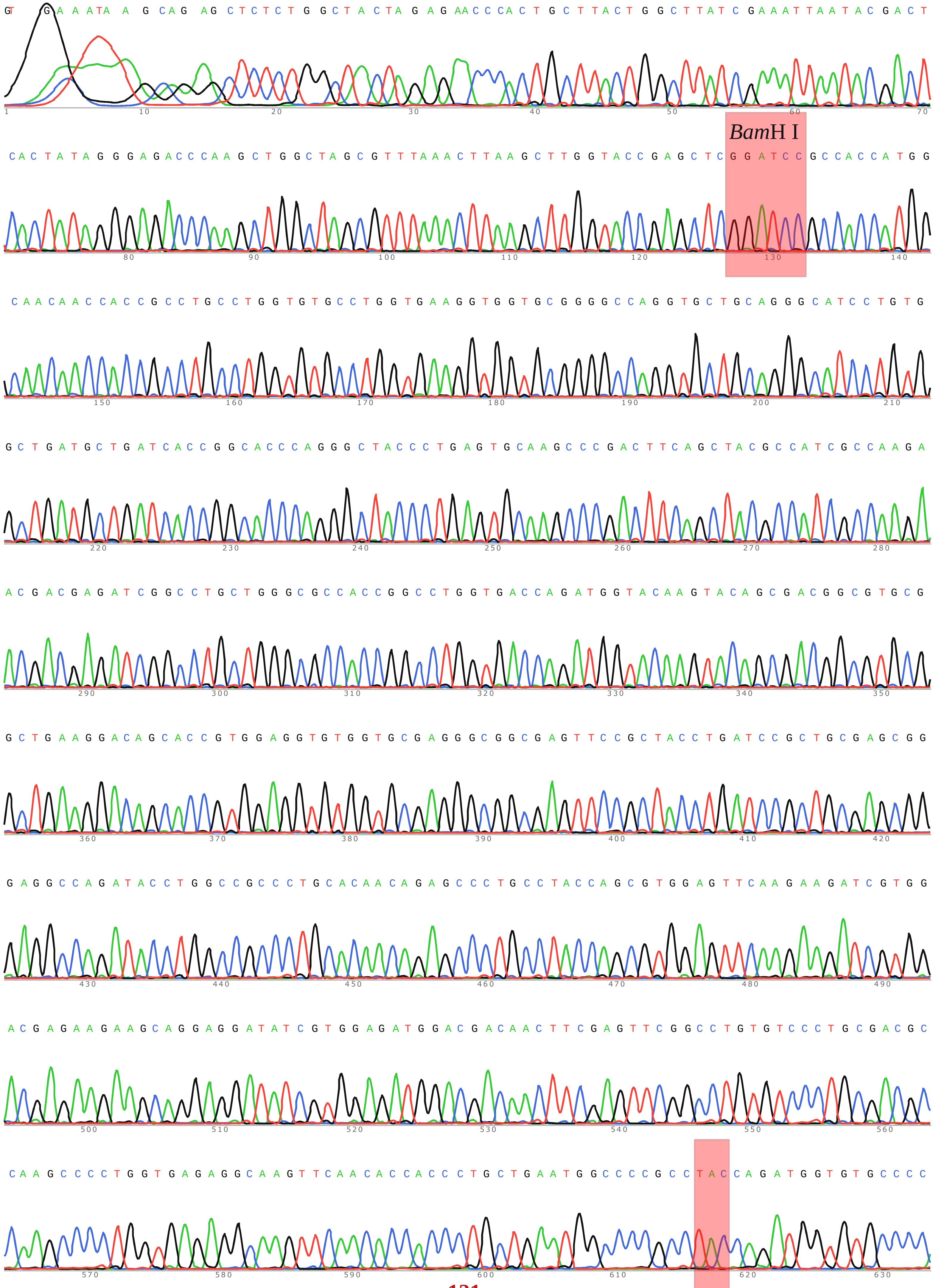
GT G A A ATA A G CAG AG C T C T C T G G C T A C T A G A G A A C C C A C T G C T T A C T G G C T T A T C G A A A T T A A T A C G A C T
*Bam*H I
C A C T A T A G G G A G A C C C A A G C T G G C T A G C G T T T A A A C T T A A G C T T G G T A C C G A G C T C G G A T C C G C C A C C A T G G
C A A C A A C C A C C G C C T G C C T G G T G T G C C T G G T G A A G G T G G T G C G G G G C C A G G T G C T G C A G G G C A T C C T G T G
G C T G A T G C T G A T C A C C G G C A C C C A G G G C T A C C C T G A G T G C A A G C C C G A C T T C A G C T A C G C C A T C G C C A A G A
A C G A C G A G A T C G G C C T G C T G G G C G C C A C C G G C C T G G T G A C C A G A T G G T A C A A G T A C A G C G A C G G C G T G C G
G C T G A A G G A C A G C A C C G T G G A G G T G T G G T G C G A G G G C G G C G A G T T C C G C T A C C T G A T C C G C T G C G A G C G G
G A G G C C A G A T A C C T G G C C G C C C T G C A C A A C A G A G C C C T G C C T A C C A G C G T G G A G T T C A A G A A G A T C G T G G
A C G A G A A G A A G C A G G A G G A T A T C G T G G A G A T G G A C G A C A A C T T C G A G T T C G G C C T G T G T C C C T G C G A C G C
C A A G C C C C T G G T G A G A G G C A A G T T C A A C A C C A C C C T G C T G A A T G G C C C C G C C T A C C A G A T G G T G T G C C C C

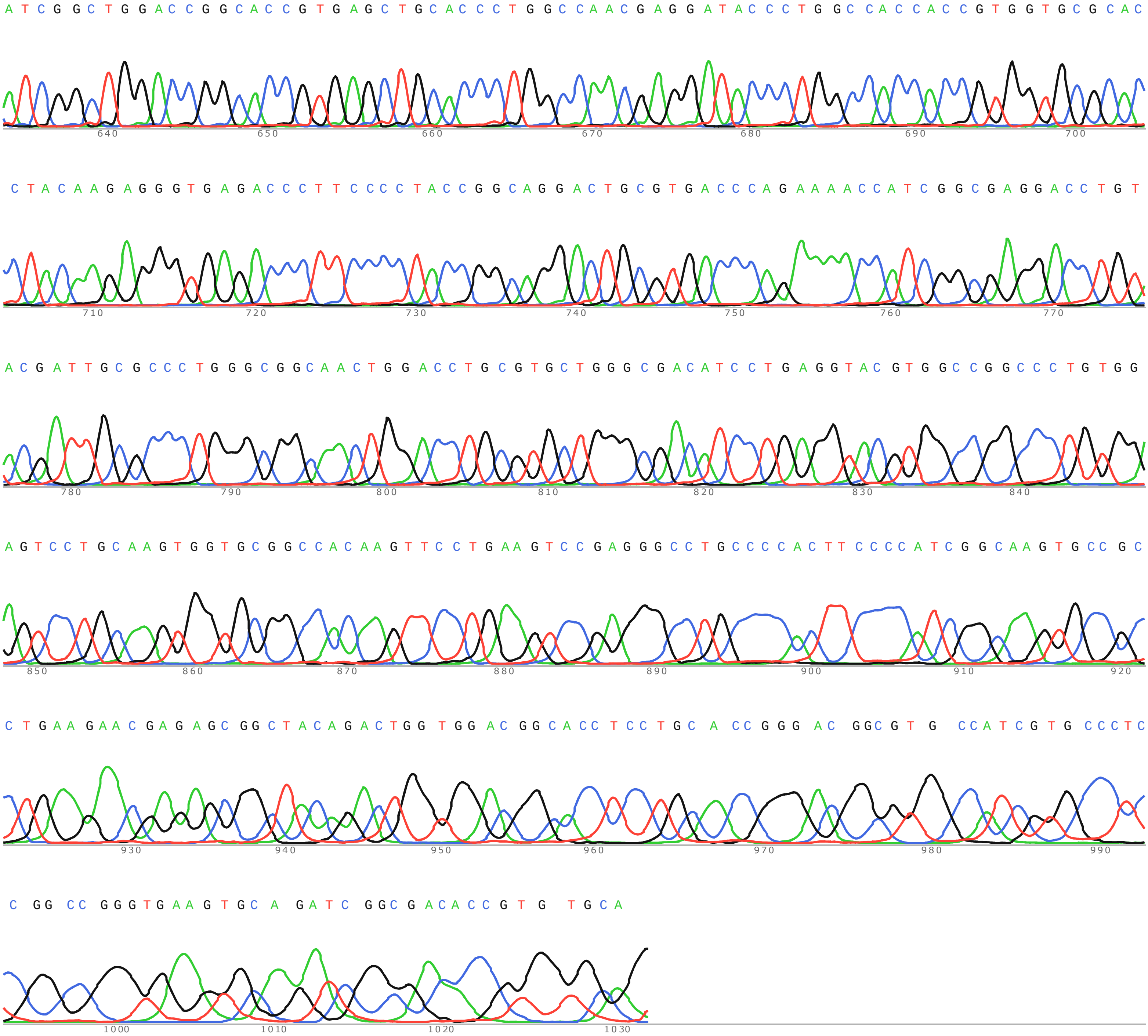
ATCGGCTGGACCGGCACCGTGAGCTGCACCCTGGCCAACGAGGATACCCTGGCCACCACCGTGGTGCGCAC
640 650 660 670 680 690 700
CTACAAGAGGGTGAGACCCTTCCCCTACCGGCAGGACTGCGTGACCCAGAAAACCATCGGCGAGGACCTGT
710 720 730 740 750 760 770
ACGATTGCGCCCTGGGCGGCAACTGGACCTGCGTGCTGGGCGACATCCTGAGGTACGTGGCCGGCCCTGTGG
780 790 800 810 820 830 840
AGTCCTGCAAGTGGTGCGGCCACAAGTTCCTGAAGTCCGAGGGCCTGCCCCACTTCCCCATCGGCAAGTGCCGC
850 860 870 880 890 900 910 920
CTGAAGAACGAGAGCGGCTACAGACTGGTGGACGGCACCTCCTGCACCGGGACGGCGTGCCATCGTGCCCTC
930 940 950 960 970 980 990
CGGCCGGGTGAAGTGCAGATCGGCGACACCGTGTGCA
1000 1010 1020 1030

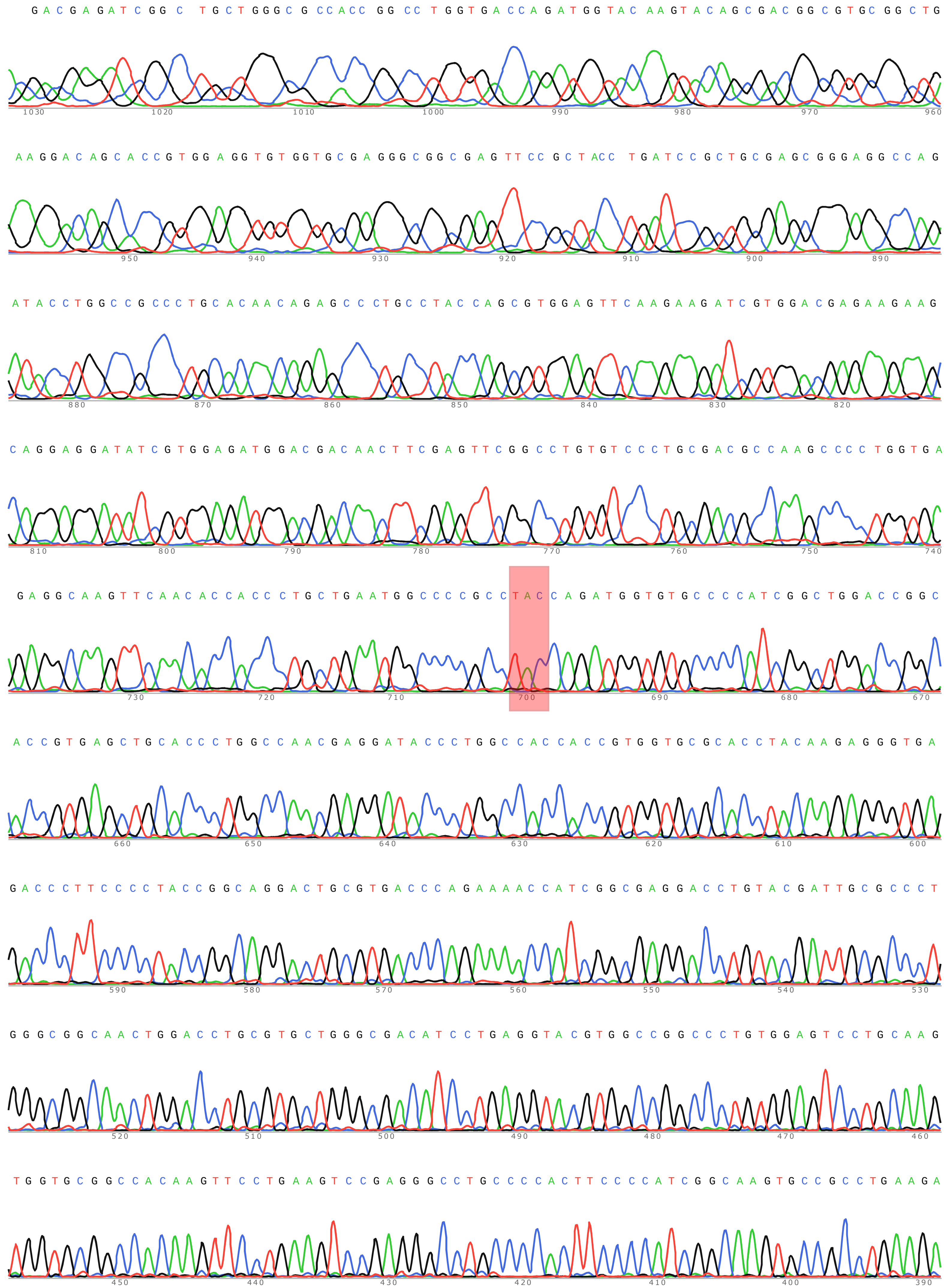
GACGAGATCGGC TGCTGGGCG CCACC GG CC TGGTGACCAGATGGTAC AAGTACAGCGACGGCGTGC GGCTG
AAGGACAGCACCGTGGAGGTGTGGTGCGAGGGCGGCGAGTTCCGCTACC TGATCCGCTGCGAGCGGGAGGCCAG
ATACCTGGCCGCCCTGCACAACAGAGCC CTGCCTAC CAGCGTGGAGTTCAAGAAGATCGTGGACGAGAAGAAG
CAGGAGGATATCGTGGAGATGGACGACAACTTCGAGTTCGGCCTGTGTCCCTGCGACGCCAAGCCCCTGGTGA
GAGGCAAGTTCAACACCACCCTGCTGAATGGCCCCGCCTACCAGATGGTGTGCCCCATCGGCTGGACCGGC
ACCGTGAGCTGCACCCTGGCCAACGAGGATACCCTGGCCACCACCGTGGTGCGCACCTACAAGAGGGTGA
GACCCTTCCCCTACCGGCAGGACTGCGTGACCCAGAAAACCATCGGCGAGGACCTGTACGATTGCGCCCT
GGGCGGCAACTGGACCTGCGTGCTGGGCGACATCCTGAGGTACGTGGCCGGCCCTGTGGAGTCCTGCAAG
TGGTGCGGCCACAAGTTCCTGAAGTCCGAGGGCCTGCCCCACTTCCCCATCGGCAAGTGCCGCCTGAAGA

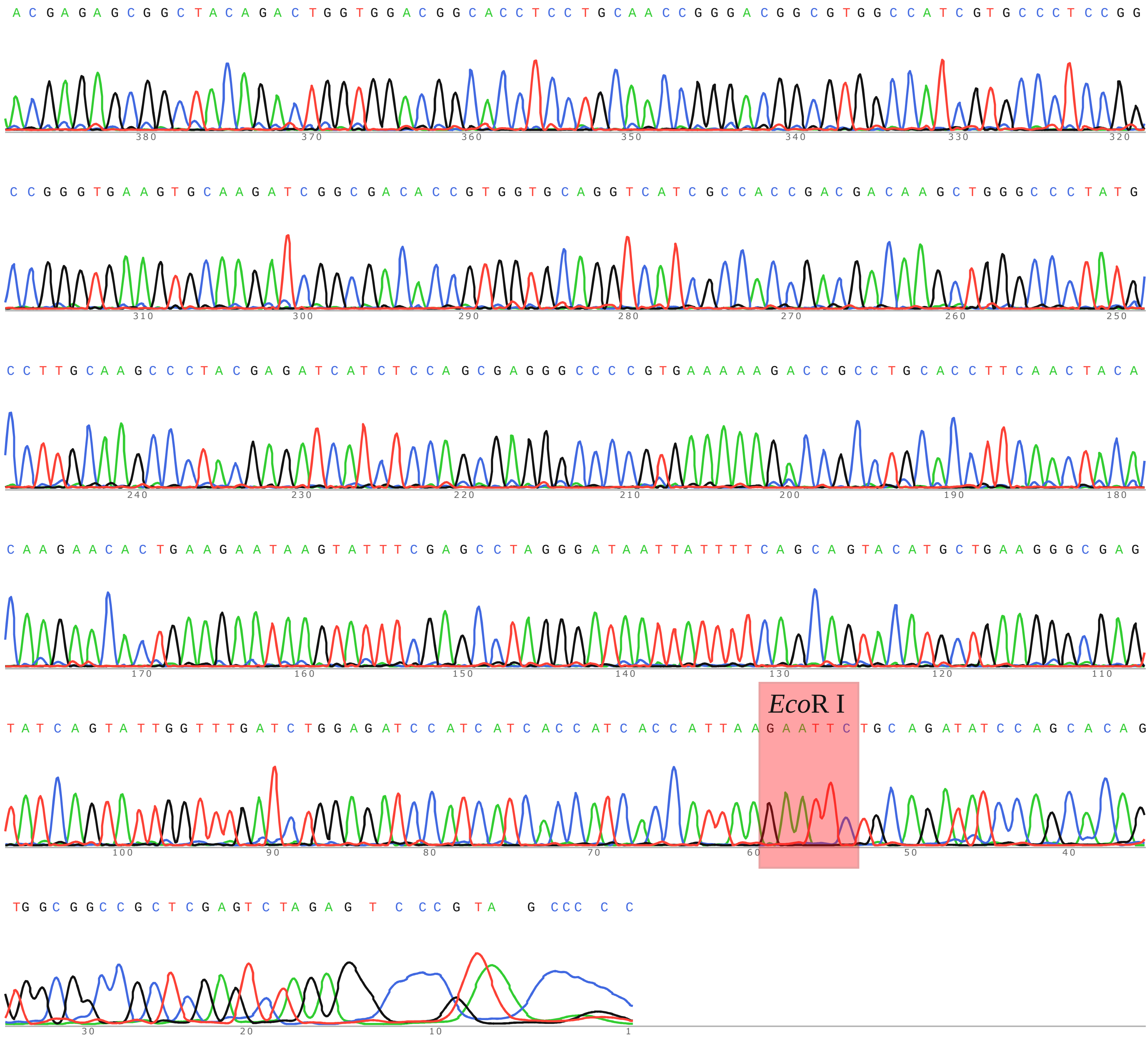
ACGAGAGCGGCTACAGACTGGTGGACGGCACCTCCTGCAACCGGGACGGCGTGGCCATCGTGCCCTCCGG
380 370 360 350 340 330 320
CCGGGTGAAGTGCAAGATCGGCGACACCGTGGTGCAGGTCATCGCCACCGACGACAAGCTGGGCCCTATG
310 300 290 280 270 260 250
CCTTGCAAGCCCTACGAGATCATCTCCAGCGAGGGCCCCGTGAAAAAGACCGCCTGCACCTTCAACTACA
240 230 220 210 200 190 180
CAAGAACACTGAAGAATAAGTATTTCGAGCCTAGGGATAATTATTTTCAGCAGTACATGCTGAAGGGCGAG
170 160 150 140 130 120 110
EcoR I
TATCAGTATTGGTTTGATCTGGAGATCCATCATCACCATCACCATTAAGAATTCTGCAGATATCCAGCACAG
100 90 80 70 60 50 40
TG GC GGCCGCTCGAGTCTAGAG T C CCG TA G CCC C C
30 20 10 1

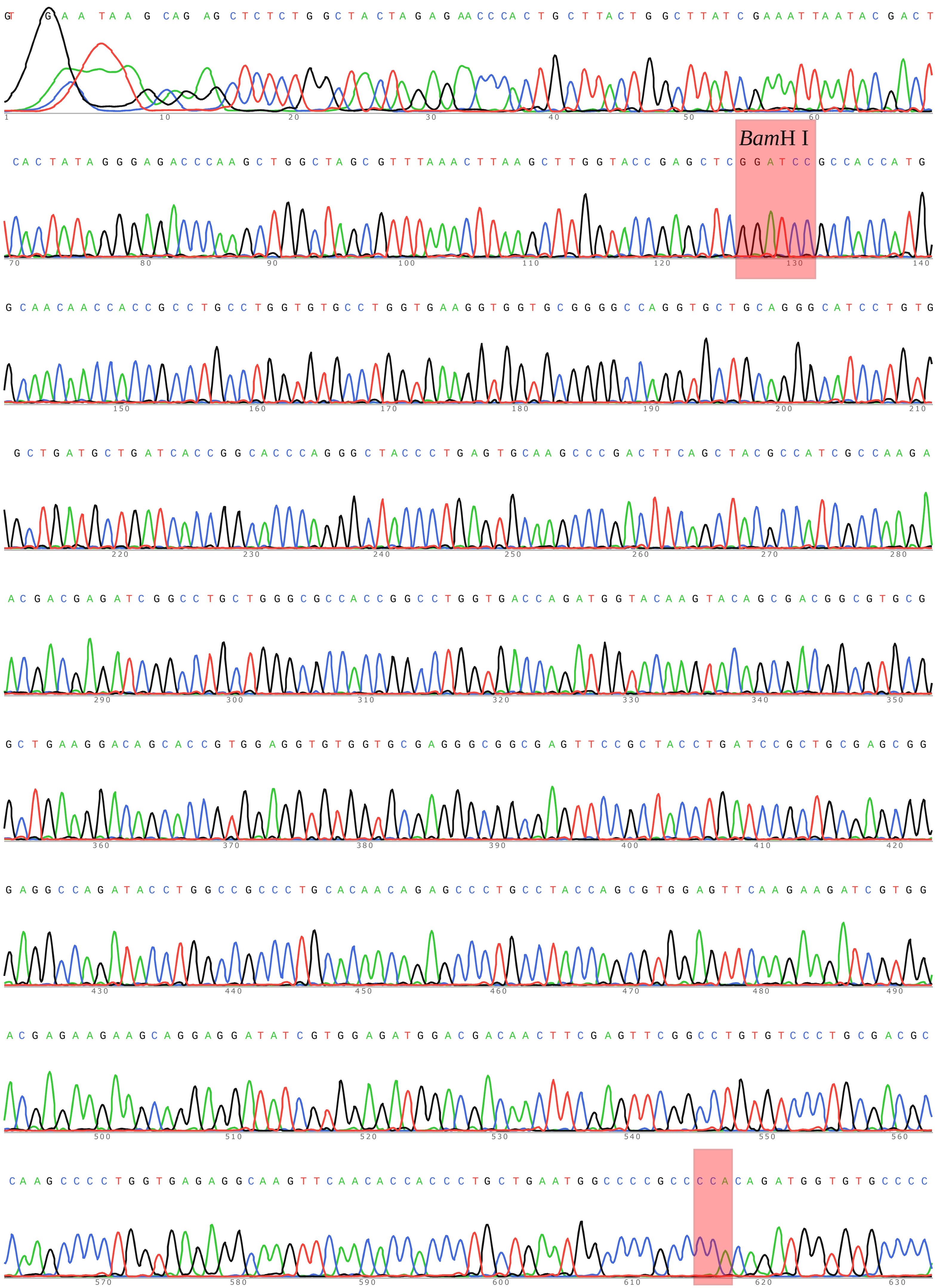
GTGAATAAGCAGAGCTCTCTGGCTACTAGAGAACCCACTGCTTACTGGCTTATCGAAATTAATACGACT
CACTATAGGGAGACCCAAGCTGGCTAGCGTTTAAACTTAAGCTTGGTACCGAGCTCGGATCCGCCACCATG
BamH I
GCAACAACCACCGCCTGCCTGGTGTGCCTGGTGAAGGTGGTGCGGGGCCAGGTGCTGCAGGGCATCCTGTG
GCTGATGCTGATCACCGGCACCCAGGGCTACCCTGAGTGCAAGCCCGACTTCAGCTACGCCATCGCCAAGA
ACGACGAGATCGGCCTGCTGGGCGCCACCGGCCTGGTGACCAGATGGTACAAGTACAGCGACGGCGTGCG
GCTGAAGGACAGCACCGTGGAGGTGTGGTGCGAGGGCGGCGAGTTCCGCTACCTGATCCGCTGCGAGCGG
GAGGCCAGATACCTGGCCGCCCTGCACAACAGAGCCCTGCCTACCAGCGTGGAGTTCAAGAAGATCGTGG
ACGAGAAGAAGCAGGAGGATATCGTGGAGATGGACGACAACTTCGAGTTCGGCCTGTGTCCCTGCGACGC
CAAGCCCCTGGTGAGAGGCAAGTTCAACACCACCCTGCTGAATGGCCCCGCCCCACAGATGGTGTGCCCC

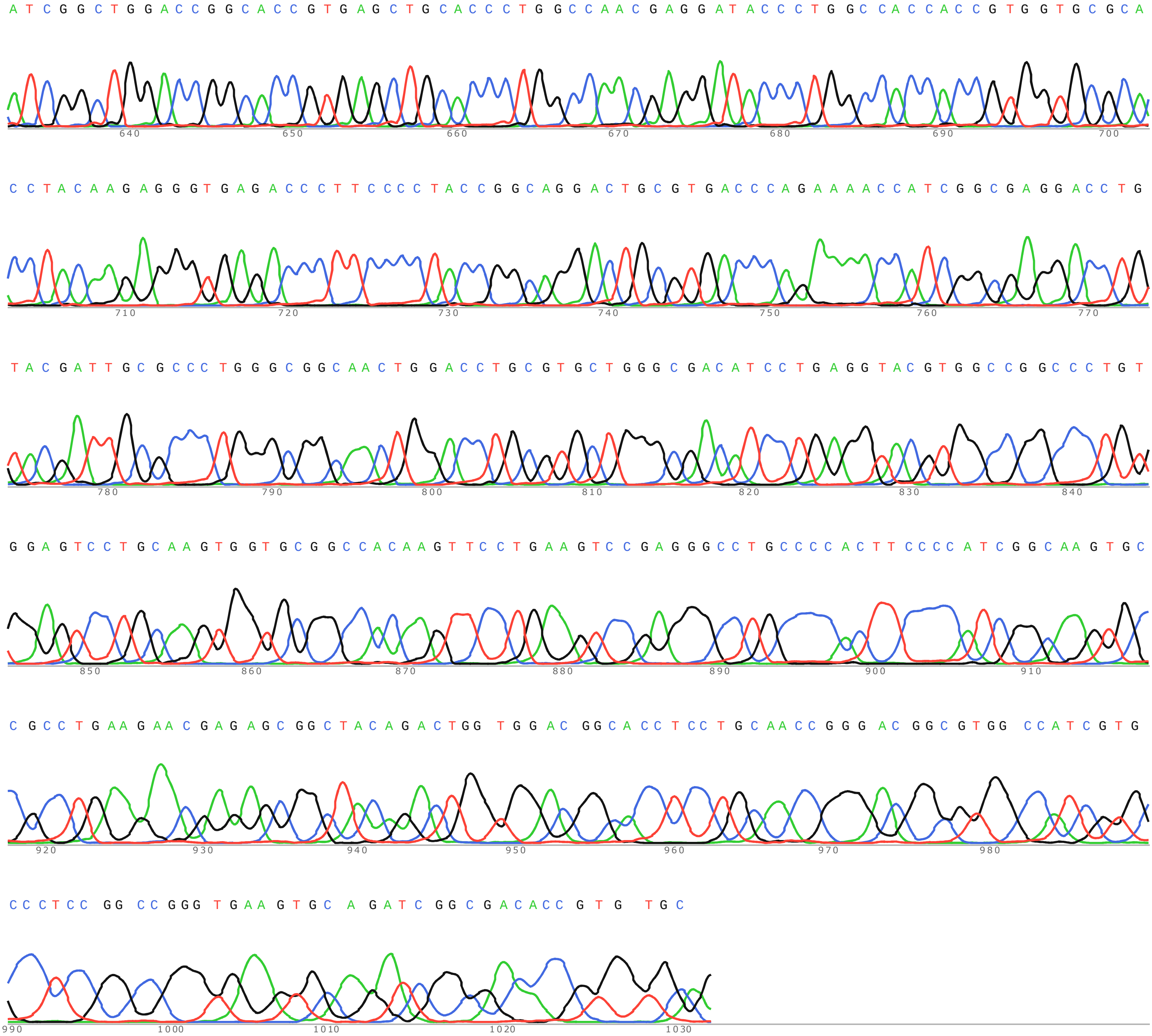
A T C G G C T G G A C C G G C A C C G T G A G C T G C A C C C T G G C C A A C G A G G A T A C C C T G G C C A C C A C C G T G G T G C G C A
640 650 660 670 680 690 700
C C T A C A A G A G G G T G A G A C C C T T C C C C T A C C G G C A G G A C T G C G T G A C C C A G A A A A C C A T C G G C G A G G A C C T G
710 720 730 740 750 760 770
T A C G A T T G C G C C C T G G G C G G C A A C T G G A C C T G C G T G C T G G G C G A C A T C C T G A G G T A C G T G G C C G G C C C T G T
780 790 800 810 820 830 840
G G A G T C C T G C A A G T G G T G C G G C C A C A A G T T C C T G A A G T C C G A G G G C C T G C C C C A C T T C C C C A T C G G C A A G T G C
850 860 870 880 890 900 910
C G C C T G A A G A A C G A G A G C G G C T A C A G A C T G G T G G A C G G C A C C T C C T G C A A C C G G G A C G G C G T G G C C A T C G T G
920 930 940 950 960 970 980
C C C T C C G G C C G G G T G A A G T G C A G A T C G G C G A C A C C G T G T G C
990 1000 1010 1020 1030

126

CGAGATCGGCCTGCTGGGCGCCACCGGCCTGGTGACCAGATGGTACAAGTACAGCGACGGCGTGCGGCTG

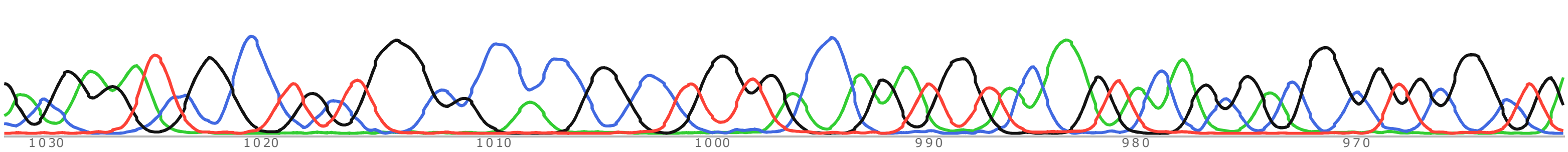

AAGGACAGCACCGTGGAGGTGTGGTGCGAGGGCGGCGAGTTCCGCTACCTGATCCGCTGCGAGCGGGAGGCCA

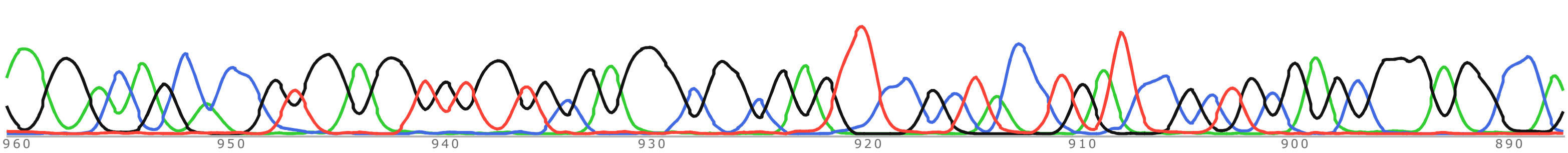

GATACCTGGCCGCCCTGCACAACAGAGCCCTGCCTACCAGCGTGGAGTTCAAGAAGATCGTGGACGAGAAGA

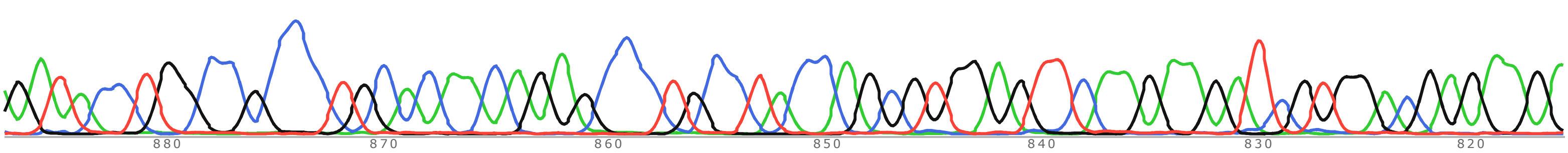

AGCAGGAGGATATCGTGGAGATGGACGACAACTTCGAGTTCGGCCTGTGTCCCTGCGACGCCAAGCCCCTG

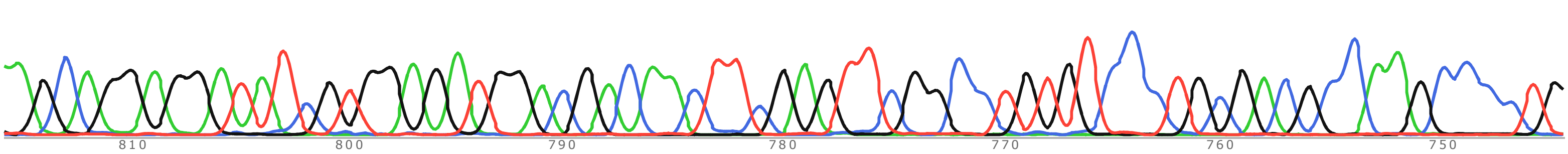

GTGAGAGGCAAGTTCAACACCACCCTGCTGAATGGCCCCGCCCCACAGATGGTGTGCCCCATCGGCTGGA

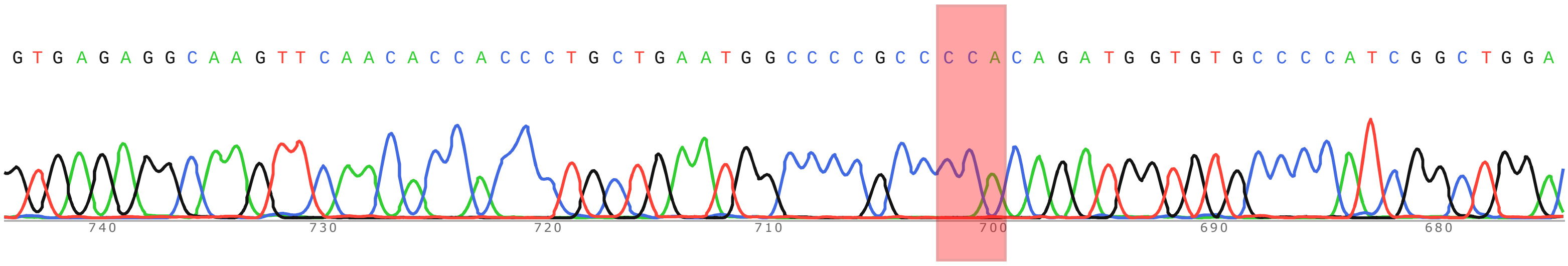

CCGGCACCGTGAGCTGCACCCTGGCCAACGAGGATACCCTGGCCACCACCGTGGTGCGCACCTACAAGAG

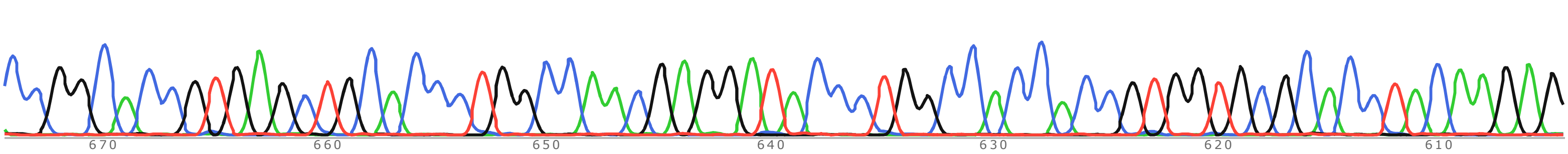

GGTGAGACCCTTCCCCTACCGGCAGGACTGCGTGACCCAGAAAACCATCGGCGAGGACCTGTACGATTGC

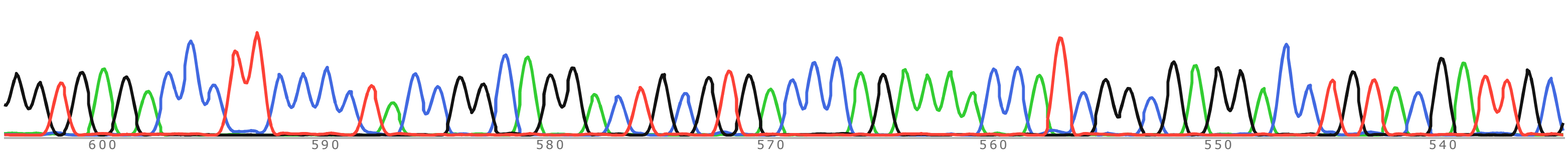

GCCCTGGGCGGCAACTGGACCTGCGTGCTGGGCGACATCCTGAGGTACGTGGCCGGCCCCTGTGGAGTCCT

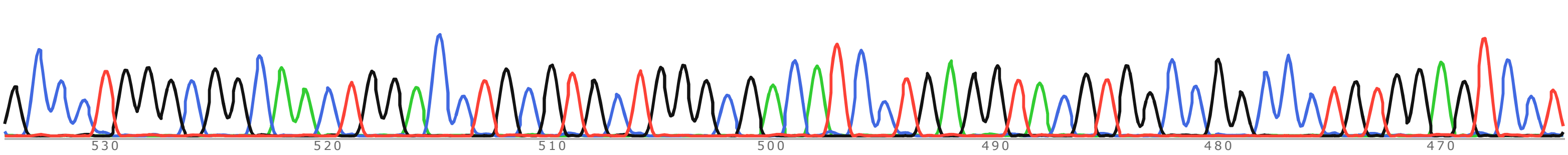

GCAAGTGGTGCGGCCACAAGTTCCTGAAGTCCGAGGGCCTGCCCCACTTCCCCATCGGCAAGTGCCGCCT

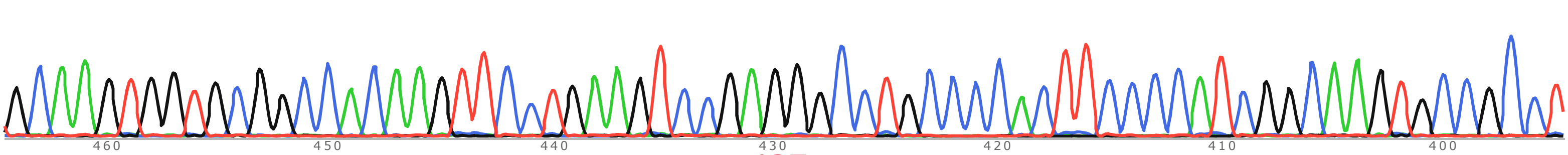

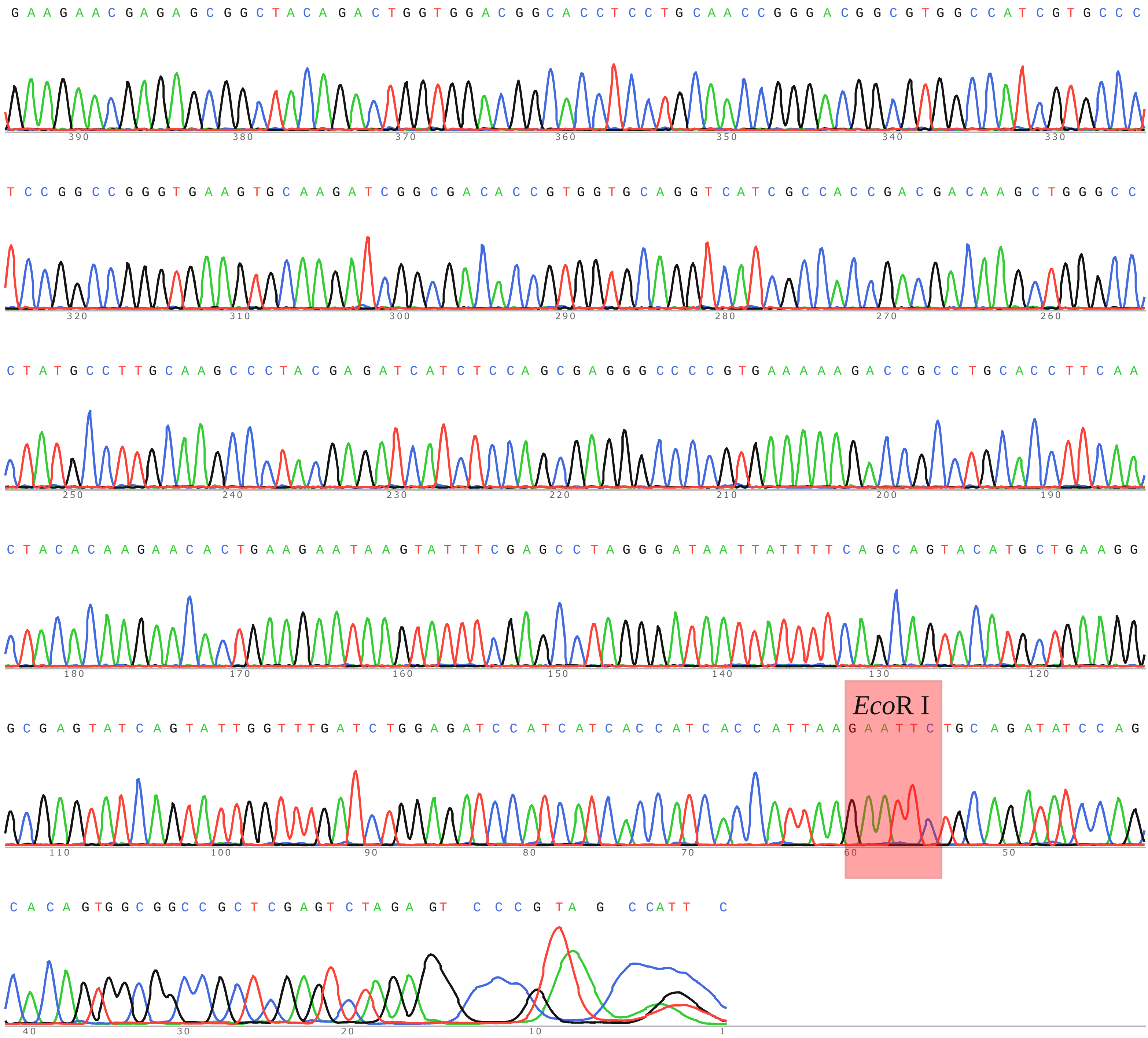
G A A G A A C G A G A G C G G C T A C A G A C T G G T G G A C G G C A C C T C C T G C A A C C G G G A C G G C G T G G C C A T C G T G C C C
390 380 370 360 350 340 330
T C C G G C C G G G T G A A G T G C A A G A T C G G C G A C A C C G T G G T G C A G G T C A T C G C C A C C G A C G A C A A G C T G G G C C
320 310 300 290 280 270 260
C T A T G C C T T G C A A G C C C T A C G A G A T C A T C T C C A G C G A G G G C C C C G T G A A A A A G A C C G C C T G C A C C T T C A A
250 240 230 220 210 200 190
C T A C A C A A G A A C A C T G A A G A A T A A G T A T T T C G A G C C T A G G G A T A A T T A T T T T C A G C A G T A C A T G C T G A A G G
180 170 160 150 140 130 120
EcoR I
G C G A G T A T C A G T A T T G G T T T G A T C T G G A G A T C C A T C A T C A C C A T C A C C A T T A A G A A T T C T G C A G A T A T C C A G
110 100 90 80 70 60 50
C A C A G T G G C G G C C G C T C G A G T C T A G A G T C C C G T A G C C A T T C
40 30 20 10 1

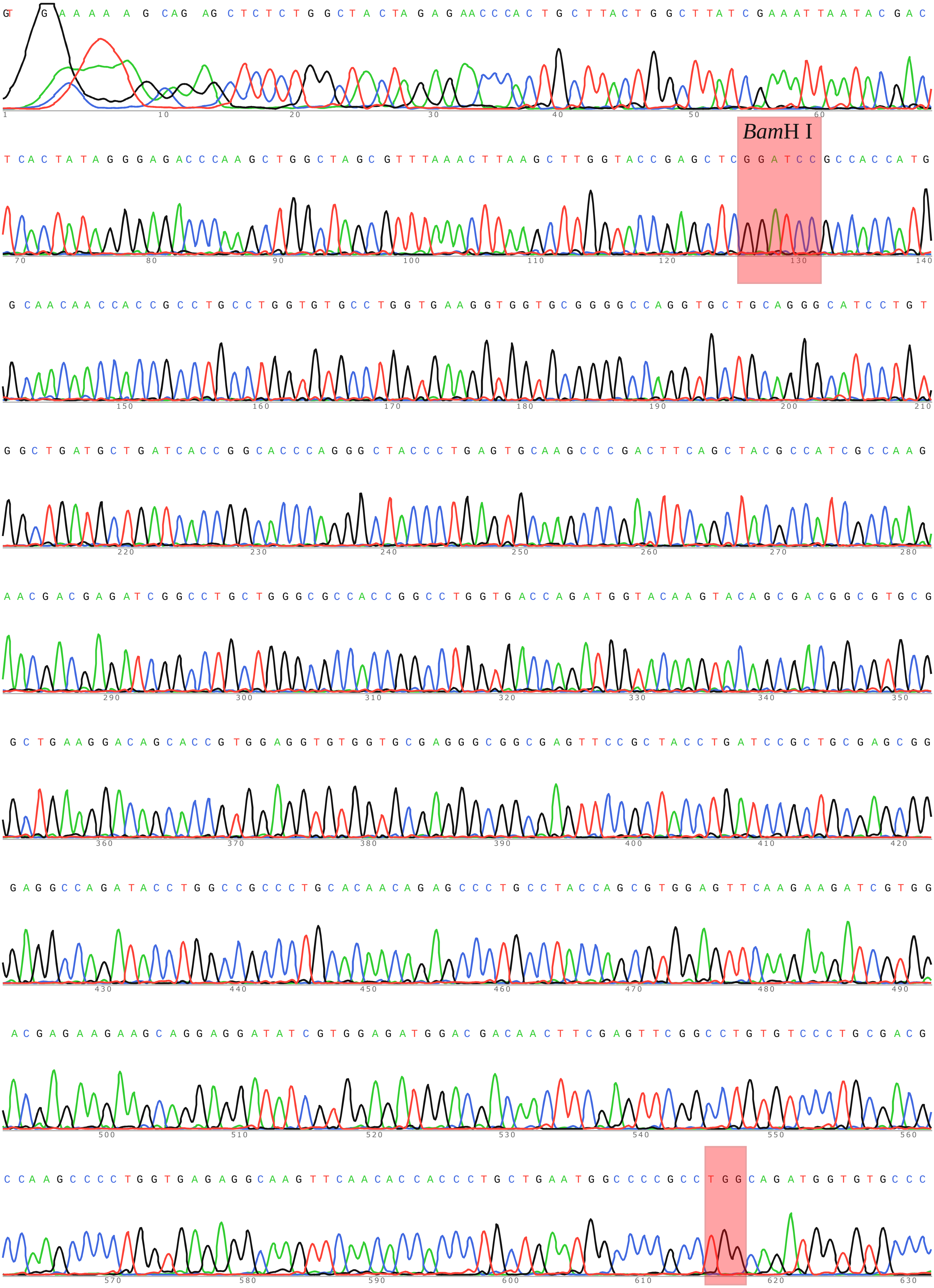
GTGAAAAAGCAGAGCTCTCTGGCTACTAGAGAACCCACTGCTTACTGGCTTATCGAAATTAATACGAC
BamH I
TCACTATAGGGAGACCCAAGCTGGCTAGCGTTTAAACTTAAGCTTGGTACCGAGCTCGGATCCGCCACCATG
GCAACAACCACCGCCTGCCTGGTGTGCCTGGTGAAGGTGGTGCGGGGCCAGGTGCTGCAGGGCATCCTGT
GGCTGATGCTGATCACCGGCACCCAGGGCTACCCTGAGTGCAAGCCCGACTTCAGCTACGCCATCGCCAAG
AACGACGAGATCGGCCTGCTGGGCGCCACCGGCCTGGTGACCAGATGGTACAAGTACAGCGACGGCGTGCG
GCTGAAGGACAGCACCGTGGAGGTGTGGTGCGAGGGCGGCGAGTTCCGCTACCTGATCCGCTGCGAGCGG
GAGGCCAGATACCTGGCCGCCCTGCACAACAGAGCCCTGCCTACCAGCGTGGAGTTCAAGAAGATCGTGG
ACGAGAAGAAGCAGGAGGATATCGTGGAGATGGACGACAACTTCGAGTTCGGCCTGTGTCCCTGCGACG
CCAAGCCCCTGGTGAGAGGCAAGTTCAACACCACCCTGCTGAATGGCCCCGCCTGGCAGATGGTGTGCCC

CATCGGCTGGACCGGCACCGTGAGCTGCACCCTGGCCAACGAGGATACCCTGGCCACCACCGTGGTGCGCA

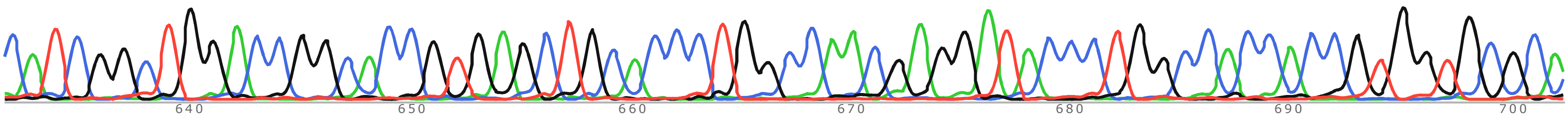

CCTACAAGAGGGTGAGACCCTTCCCCTACCGGCAGGACTGCGTGACCCAGAAAACCATCGGCGAGGACCTGT

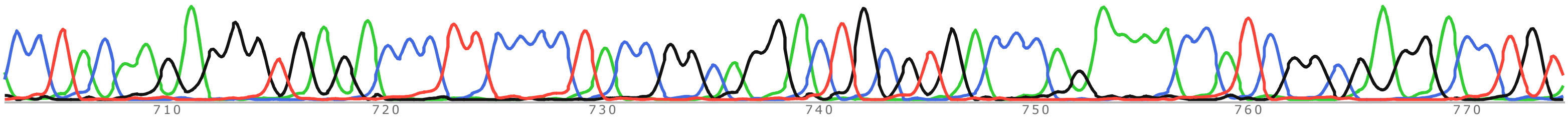

ACGATTGCGCCCTGGGCGGCAACTGGACCTGCGTGCTGGGCGACATCCTGAGGTACGTGGCCGGCCCTGTGGA

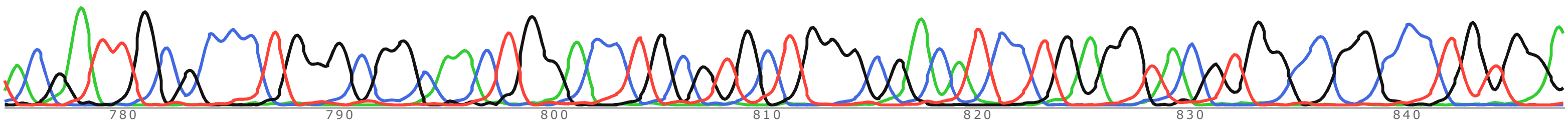

GTCCTGCAAGTGGTGCGGCCACAAGTTCCTGAAGTCCGAGGGCCTGCCCCACTTCCCCATCGGCAAGTGCCGCC

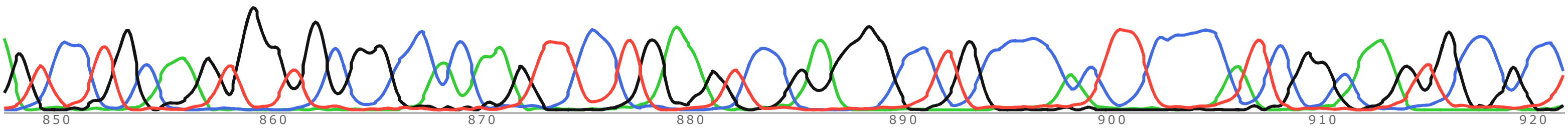

TGAAGAACGAGAGCGGCTACAGACTGGTGGACGGCACCTCCTGCAACCGGGACGGCGTGGCCATCGTGCCCTCC

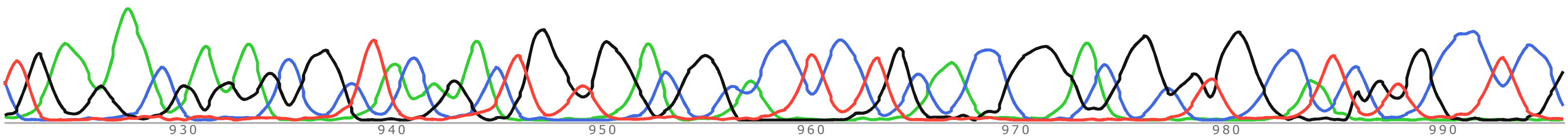

GGCCGGGTGAAGTGCAAGATCGGCGACACCGTGTG

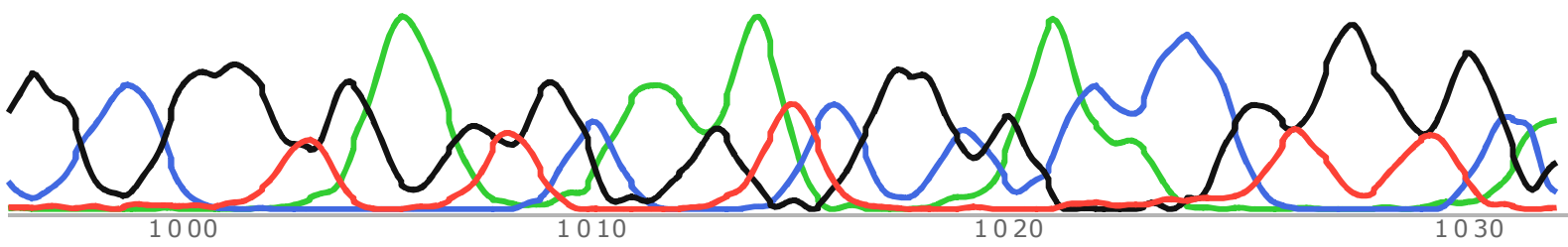

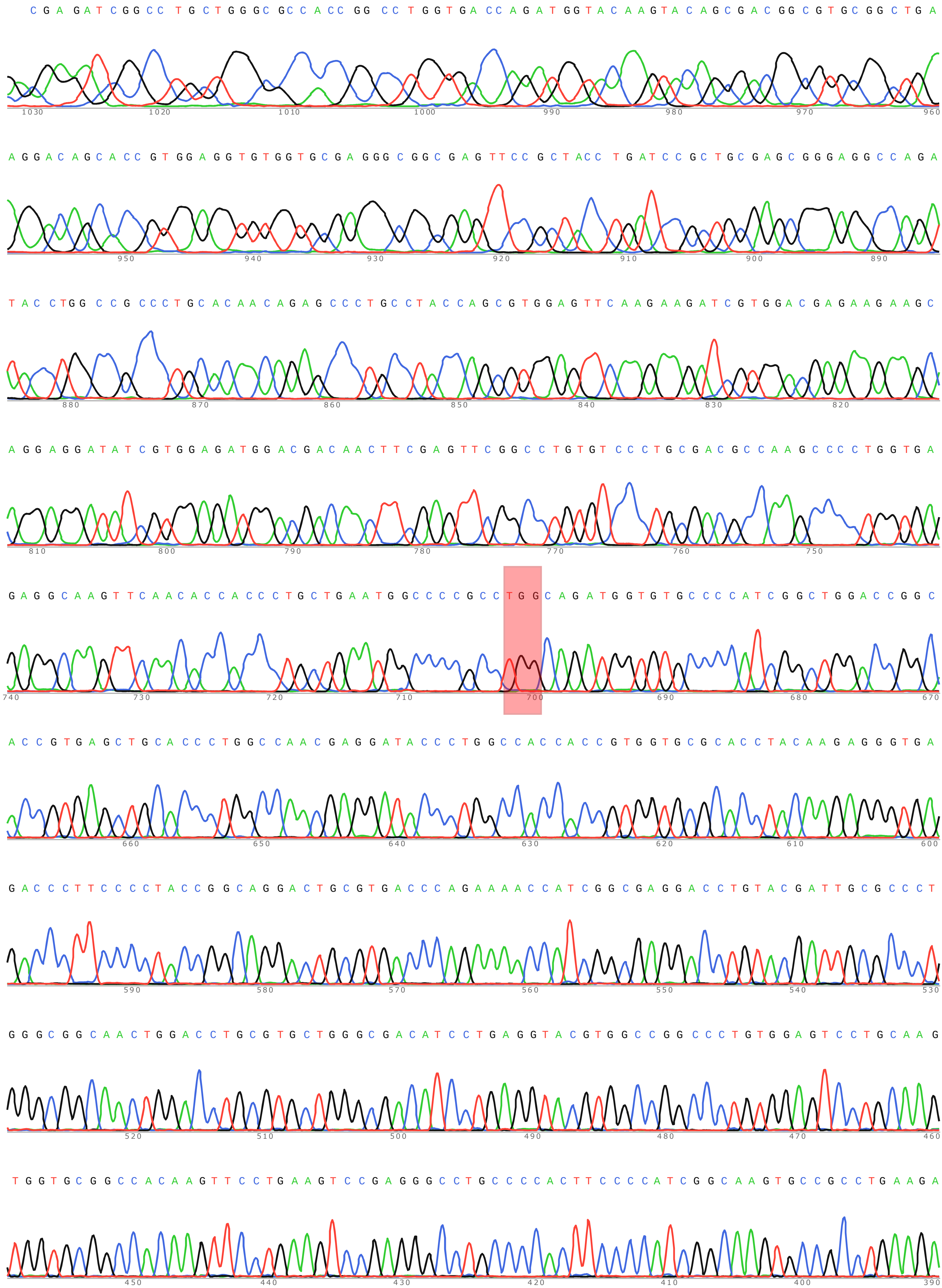
CGAGATCGGCCTGCTGGGCGCCACCGGCCTGGTGACCAGATGGTACAAGTACAGCGACGGCGTGCGGCTGA
1030 1020 1010 1000 990 980 970 960
AGGACAGCACCGTGGAGGTGTGGTGCGAGGGCGGCGAGTTCCGCTACCTGATCCGCTGCGAGCGGGAGGCCAGA
950 940 930 920 910 900 890
TACCTGGCCGCCCTGCACAACAGAGCCCTGCCTACCAGCGTGGAGTTCAAGAAGATCGTGGACGAGAAGAAGC
880 870 860 850 840 830 820
AGGAGGATATCGTGGAGATGGACGACAACTTCGAGTTCGGCCTGTGTCCCTGCGACGCCAAGCCCCTGGTGA
810 800 790 780 770 760 750
GAGGCAAGTTCAACACCACCCTGCTGAATGGCCCCGCCTGGCAGATGGTGTGCCCCATCGGCTGGACCGGC
740 730 720 710 700 690 680 670
ACCGTGAGCTGCACCCTGGCCAACGAGGATACCCTGGCCACCACCGTGGTGCGCACCTACAAGAGGGTGA
660 650 640 630 620 610 600
GACCCTTCCCCTACCGGCAGGACTGCGTGACCCAGAAAACCATCGGCGAGGACCTGTACGATTGCGCCCT
590 580 570 560 550 540 530
GGGCGGCAACTGGACCTGCGTGCTGGGCGACATCCTGAGGTACGTGGCCGGCCCTGTGGAGTCCTGCAAG
520 510 500 490 480 470 460
TGGTGCGGCCACAAGTTCCTGAAGTCCGAGGGCCTGCCCCACTTCCCCATCGGCAAGTGCCGCCTGAAGA
450 440 430 420 410 400 390

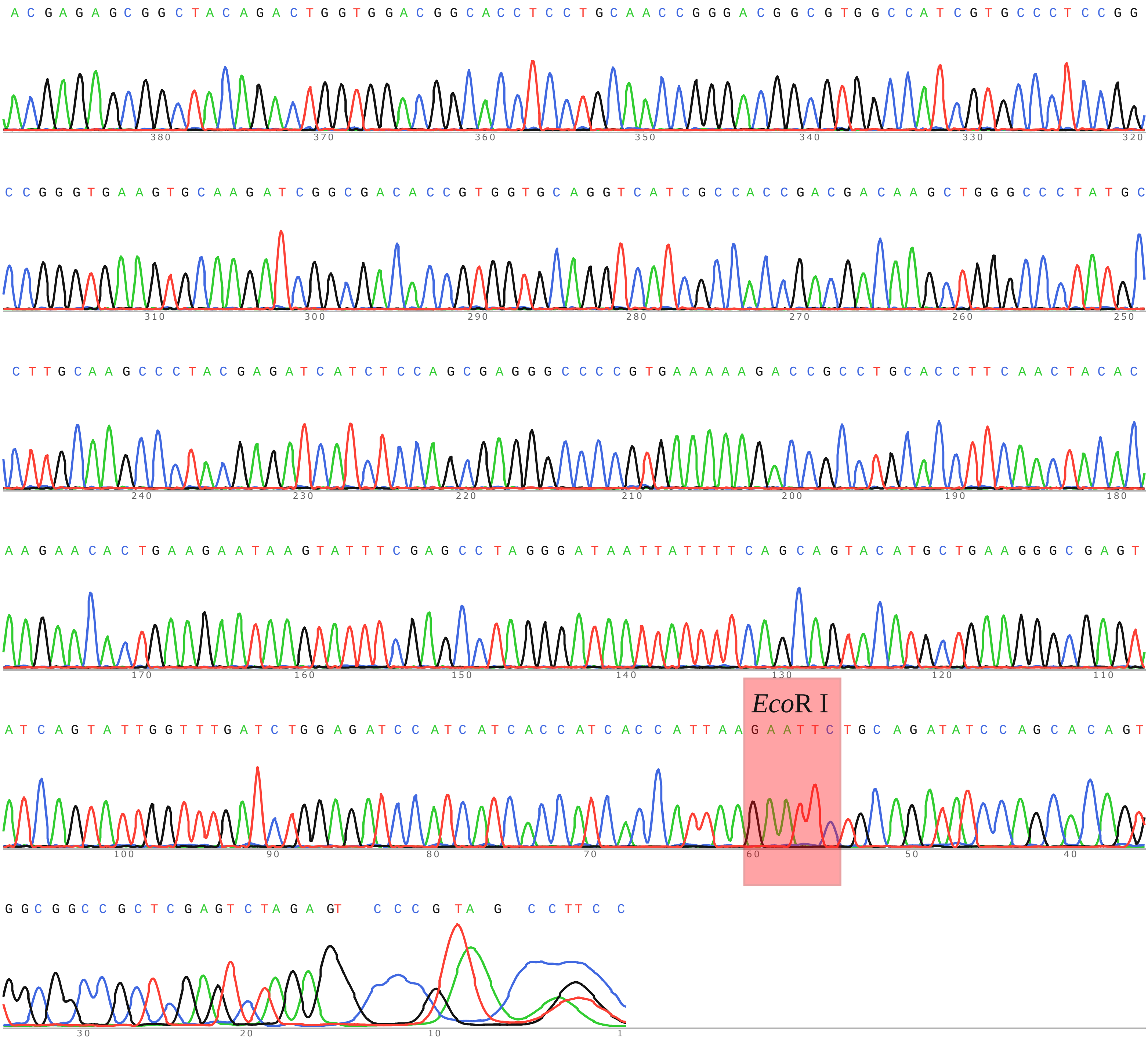
ACGAGAGCGGCTACAGACTGGTGGACGGCACCTCCTGCAACCGGGACGGCGTGGCCATCGTGCCCTCCGG
CCGGGTGAAGTGCAAGATCGGCGACACCGTGGTGCAGGTCATCGCCACCGACGACAAGCTGGGCCCTATGC
CTTGCAAGCCCTACGAGATCATCTCCAGCGAGGGCCCCGTGAAAAAGACCGCCTGCACCTTCAACTACAC
AAGAACACTGAAGAATAAGTATTTCGAGCCTAGGGATAATTATTTTCAGCAGTACATGCTGAAGGGCGAGT
ATCAGTATTGGTTTGATCTGGAGATCCATCATCACCATCACCATTAAGAATTCTGCAGATATCCAGCACAGT
EcoR I
GGCGGCCGCTCGAGTCTAGAGTCCCGTAGCCTTCC
