# Supplementary material for: Generation and epitope mapping of two distinct neutralizing mAb subsets targeting BVDV E2: a pan-pestivirus cross-reactive linear epitope and a BVDV-specific conformational epitope
Source: Vet Res. 2026 Oct 7;57:176. doi: 10.1186/s13567-026-01852-5 (PMC13644284; doi:10.1186/s13567-026-01852-5)
Supplement: Supplementary file 5 [file 13567_2026_1852_MOESM6_ESM.pdf]

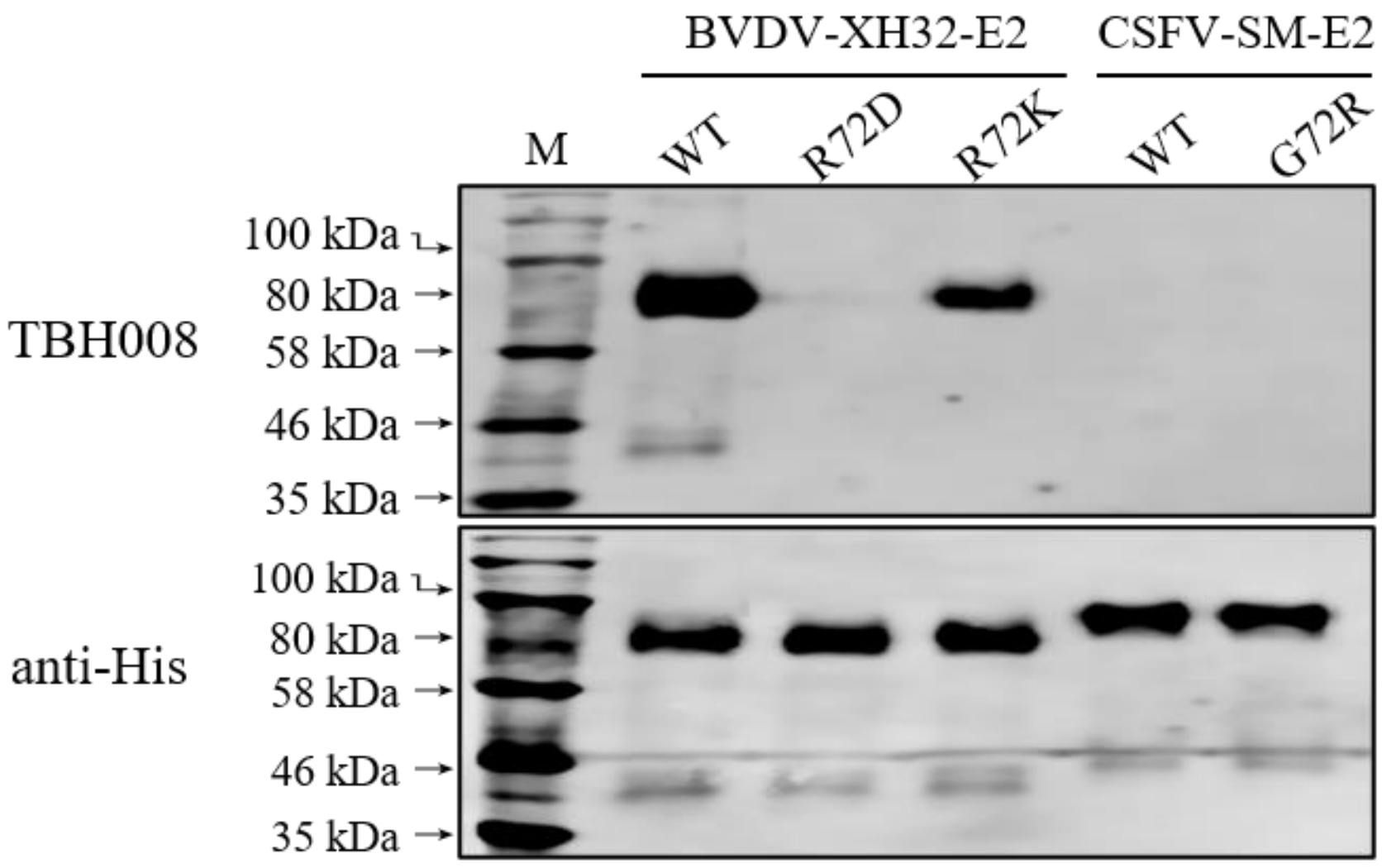

Reactivity of mAb TBH008 with E2 proteins bearing mutation at aa position 72 of BVDV XH32 and CSFV SM. The residue ^{72}R of BVDV-XH32-E2 was mutated to D and K, respectively, while residue ^{72}G of CSFV-SM-E2 was mutated to R. Position-72 substituted E2 variants were transiently expressed in HEK-293 cells, and recombinant proteins harvested from culture supernatants were analyzed by Western blot. As a result, mAb TBH008 reacted with wild-type BVDV-XH32-E2 but not wild-type CSFV-SM-E2. The R72D mutation in BVDV-XH32-E2 completely abrogated TBH008 binding, whereas the R72K mutation had no effect on TBH008 recognition. In parallel, the G72R mutation in CSFV-SM-E2 failed to restore binding reactivity to TBH008.
